# A Bacillales-specific tubular scaffold essential for NADH dehydrogenase activity

**DOI:** 10.1038/s41467-026-74385-2

**Published:** 2026-06-18

**Authors:** Farida Seduk, Rayan Osman, Pierre Simon Garcia, Lilou Bizien-Jaglin, Pauline Juyoux, Artemis Kosta, Salomé Sauvage, Maria J. Maté, Fabien Pierrel, Régine Lebrun, Guy Schoehn, Yoshiki Yamaryo-Botté, Cyrille Y. Botté, Yvain Nicolet, Mickael V. Cherrier, Anne Walburger, Axel Magalon

**Affiliations:** 1 Aix Marseille Univ, CNRS, LCB, IM2B Marseille, France; 2 Univ. Grenoble-Alpes, CNRS, CEA, IBS, Metalloproteins Unit, Grenoble, France; 3 Université Grenoble Alpes, CNRS, CEA, EMBL, ISBG, Grenoble, France; 4 Aix Marseille Univ, CNRS, IMM, Microscopy Core Facility, IM2B Marseille, France; 5 Aix Marseille Univ, CNRS, IMM, Marseille Protéomique, IM2B Marseille, France; 6 Aix Marseille Univ, CNRS, AFMB, IM2B Marseille, France; 7 Univ. Grenoble-Alpes, CNRS, UMR5525, VetAgro Sup, Grenoble INP, TIMC, Grenoble, France; 8 Univ Grenoble-Alpes, CNRS, CEA, IBS, Electron Microscopy and Methods Unit, Grenoble, France; 9 Apicolipid Team & Gemeli Platform, Institut pour l’Avancée des Biosciences, CNRS UMR5309, INSERM U1209, Université Grenoble Alpes, La Tronche, France

**Keywords:** Cryoelectron microscopy, Microbiology, Structural biology, Biophysics, Biochemistry

## Abstract

Respiratory type II NADH:quinone oxidoreductases (NDH-II) are typically monotopic flavoproteins that make direct contact with the membrane to access the quinone pool. Here, we show that in *Bacillus subtilis*, one NDH-II, termed Ndh, assembles with the helical membrane plugin (HMP) protein YjlC and forms supramolecular fibers. Genetic and biochemical analyses demonstrate that Ndh and YjlC proteins are essential for NADH oxidation. Cryo-EM analysis reveals that YjlC forms a tubular scaffold onto which multiple Ndh subunits are regularly docked via their C-terminal domain, repurposed from its classical role in direct membrane binding. These fibers can extend up to ~1000 Å, creating a continuous hydrophobic tunnel filled with lipids and quinones, thereby mimicking the membrane environment. Comparative genomics unveils that this partnership arose exclusively within *Bacillales* through the recruitment of an ancestral HMP originally associated with sulfide:quinone reductases. Together, our findings uncover a lineage-specific structural adaptation in which NDH-II enzymes depend on an HMP scaffold, expanding their functional diversity beyond the classical monotopic paradigm.

## Abstract (web summary)

Quinone-reducing respiratory enzymes are usually described as interacting directly with membranes. Here, authors show that Bacillales use a tubular protein scaffold to organize NADH dehydrogenase and connect it to quinones.

## Introduction

Electron transfer chains, located in the cytoplasmic membrane of prokaryotes and the mitochondrial membrane of eukaryotes, are essential energy hubs for organisms that depend on respiration. They channel electrons from reduced molecules to terminal electron acceptors such as oxygen, fumarate or nitrate, while simultaneously driving ion translocation or pumping, thereby creating electrochemical gradients essential for life. NADH, produced by central metabolism, serves as a primary electron donor in both prokaryotes and eukaryotes. Three evolutionarily distinct enzyme families utilize NADH to feed electrons into the respiratory chain. The first two families are multi-subunit membrane complexes that couple electron transfer to the translocation of protons or sodium ions across the membrane. The first includes proton-pumping enzymes, exemplified by Complex I (also known as type-I NADH dehydrogenase or NDH-I), that are broadly conserved across the tree of life^1^. The second comprises sodium-translocating NADH:quinone oxidoreductases (NQR or RNF in related systems), which are restricted to a few bacterial phyla^2–4^. The third family consists of the alternative NAD(P)H:quinone oxidoreductases (also known as NDH-II), which, unlike the other two, are non-electrogenic and generally simpler enzymes^5,6^. In many pathogenic bacteria, NQR and NDH-II enzymes serve as the primary gateways for electron entry into the respiratory chain, making them long-standing targets for antibacterial drug development^7–10^. While NQRs are restricted to prokaryotes, NDH-IIs are widespread across all domains of life. In humans, proteins such as AIF (Apoptosis-Inducing Factor) and FSP1 (Ferroptosis Suppressor Protein 1) are NDH-II homologs with confirmed NADH:quinone oxidoreductase activity^11–13^.

NDH-II enzymes are currently described primarily as monomeric peripheral membrane flavoproteins, belonging to a broad superfamily and characterized by two conserved domains for binding FAD and NADH^12,14^. Their C-terminal region, composed of amphipathic helices, is the most variable region of the protein and plays a dual role in quinone binding and direct membrane association^15–18^. This organization integrates NADH oxidation, quinone reduction, and membrane interaction within a single polypeptide chain, unlike other NADH dehydrogenases which typically require multiple subunits. However, emerging evidence suggests that, in some NDH-II, membrane attachment would involve additional protein partners, indicating broader structural and functional diversity than previously recognized^19,20^.

Here, we combine comparative genomics, biochemistry, and structural biology to reveal the structural adaptation of NDH-II in *Bacillales*. In *Bacillus subtilis*, one NDH-II, Ndh, forms a complex with the helical membrane plugin (HMP) protein YjlC, with both proteins being essential for NADH oxidation. Cryo-EM reveals that YjlC assembles into a tetrameric superhelical tubular scaffold that recruits four Ndh molecules via their C-terminal domains, and further polymerizes into supramolecular fibers. Together, these findings reveal an alternative strategy for NDH-II organization and membrane interaction, highlighting how *Bacillales* expanded the functional landscape of this enzyme family.

## Results

### Genomic association between NDH-II and HMP to *Bacillales*

**Fig. 1:**
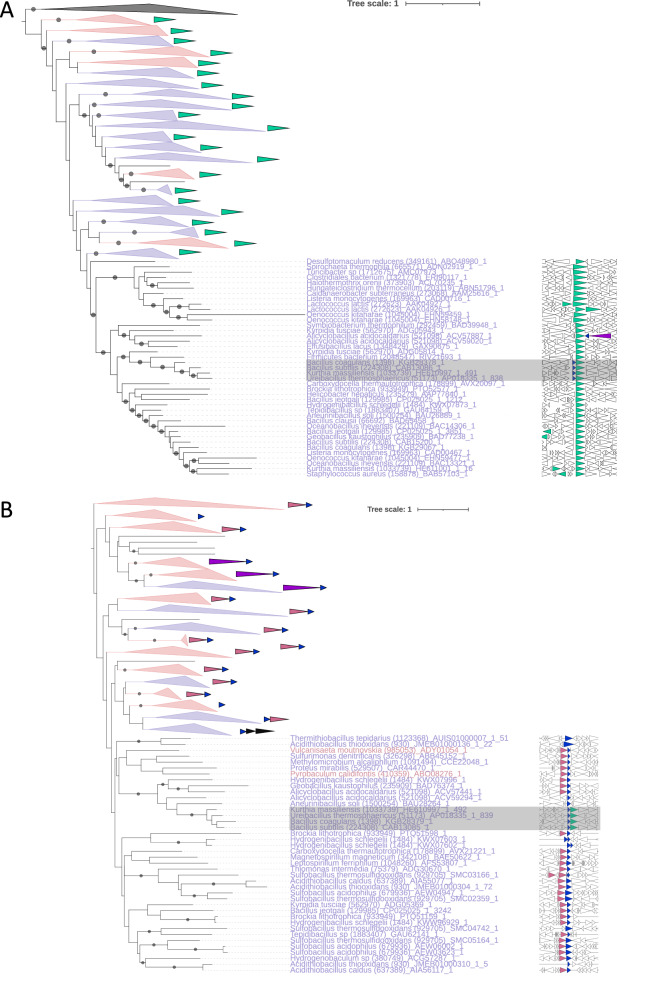
Phylogenetic trees of NDH-II and HMP proteins highlight their specific association in *Bacillales.* Phylogeny of NDH-II (**A**) and HMP (**B**). The phylogenetic trees have been inferred using IQ-TREE (NDH-II: 318 sequences, 359 amino-acid sites, Q.Pfam+I + R10, HMP: 200 sequences, 132 amino-acid sites, LG + F + R5). The NDH-II phylogeny has been rooted using 26 SQR sequences as an outgroup (gray triangle). The scale bars represent the average number of substitutions per site. The circles on branches represent ultra-fast bootstrap values >= 0.95. Most clades were collapsed except those containing HMP-associated NDH-II. Bacteria are indicated in blue and Archaea in red. For uncollapsed clades, the taxonomic ID is indicated in parentheses, and the accession number of protein sequences is provided. The genomic vicinity between NDH-II (green), HMP (blue), FDH (violet), SQR (red), and [Ni-Fe] hydrogenase (black) is mapped on the phylogeny, on a genomic window of 5000 bp. Specific association between NDH-II and HMP is highlighted on both phylogenies by a black rectangle. For collapsed clades, the predominant genomic organization is shown. The corresponding uncollapsed phylogenies are provided in Fig. S2. Source data are provided as a Source Data file.

To explore the sequence diversity of NDH-II and identify additional protein partners likely to mediate membrane association and thus provide NDH-II access to the quinone pool, we used a phylogenetic approach combined with comparative genomics. Because such accessory components are expected to functionally cooperate with NDH-II, we reasoned that they would most likely be encoded by genes located in close proximity to *ndh*. First, we identified homologs of NDH-II and related sulfide:quinone oxidoreductases (SQR)^21^ across a representative genomic database of Bacteria and Archaea, which includes over 10,000 genomes (Tables S1 and S2). We then mapped the distribution of NDH-II and SQR genes onto reference phylogenies of Bacteria and Archaea (Fig. S1), revealing a scattered occurrence across prokaryotic lineages, with a higher prevalence in Bacteria. A rooted tree of NDH-II, using SQR as an outgroup, does not show a distinct split between bacterial and archaeal sequences, preventing a confident inference of NDH-II presence in the last common ancestor (Fig. 1A and Fig. S2a). The higher prevalence in Bacteria compared to Archaea could favor a bacterial emergence.

Comparative genomic analysis using genomic neighborhoods identified three protein families frequently co-localized with NDH-II: polyprenyl synthetases, S8 peptidases, and HMP proteins. Among these, HMP stood out, because such proteins form tubular membrane-associated structures connecting oxidoreductases to the quinone pool. Recent examples include formate dehydrogenase in *B. subtilis*^19^ and [Ni-Fe] hydrogenase in *Mycobacterium smegmatis*^22^. These observations suggest that HMP might also interact with NDH-II, prompting us to investigate their diversity, distribution, and genomic associations in more detail.

HMP homologs exhibit a scattered taxonomic distribution in a few bacterial and archaeal clades (Fig. S1). The number of HMP copies per genome ranges from 1 to 8, with marked enrichment in *Crenarchaeota* and *Firmicutes* (Table S2). This variability points to a history shaped by multiple gene duplication events and/or horizontal gene transfers. Mapping the genomic contexts of HMP genes onto their phylogeny further revealed that they are frequently located near genes encoding four types of oxidoreductases recently proposed to be HMP-associated: formate dehydrogenases (FDH), SQR, [Ni-Fe] hydrogenases, and NDH-II^19^ (Fig. 1B, Fig. S2b, Table S2). Most HMP genes are located adjacent to SQR genes, whereas those near FDH genes cluster into a distinct and more restricted clade comprising mainly *Firmicutes* and *Crenarchaeota*. Likewise, HMP genes found near [Ni-Fe] hydrogenase genes form a coherent group containing *Firmicutes* and *Actinobacteria*. Finally, HMP genes located near NDH-II genes form a monophyletic group represented only by *Bacillales*. Although the root of the HMP phylogeny cannot be confidently determined, the predominance of SQR-associated HMP suggests that the ancestral partner of this protein family was SQR, with subsequent, lineage-specific recruitments by FDH, [Ni-Fe] hydrogenases, and NDH-II (Fig. 1B). Considering both the NDH-II and HMP phylogenies, the reciprocal monophyly of NDH-II sequences with adjacent HMP (Fig. 1A), and of HMP associated with NDH-II (Fig. 1B), points to a single, lineage-specific event of mutual recruitment during the diversification of *Bacillales*.

### HMP is essential for NDH-II activity in *Bacillus subtilis*

The specific association of NDH-II with HMP proteins in *Bacillales* makes *B. subtilis* an ideal model to investigate whether this genomic association reflects a functional interaction. The genome of *B. subtilis* encodes three putative NDH-II enzymes (Ndh, YumB, YutJ), each featuring conserved FAD-, NADH-, and quinone-binding motifs^6^. Notably, only *ndh* is adjacent to an HMP gene (*yjlC*), suggesting a dedicated partnership (Table S2).

**Fig. 2:**
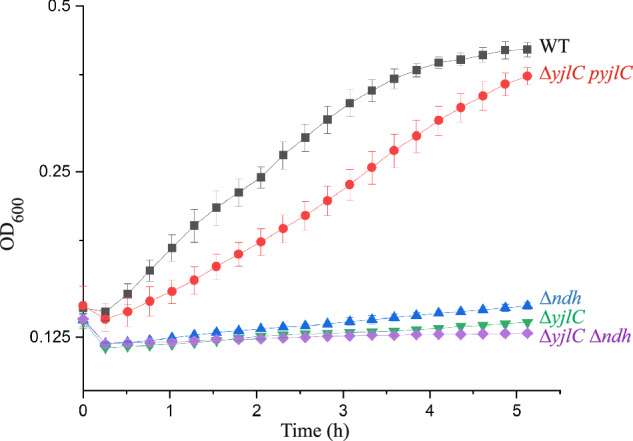
The HMP-containing protein YjlC and Ndh are essential for *B. subtilis* growth on malate. Growth of *B. subtilis* strains in chemically defined CE medium with malate as the sole carbon source. Data represent the mean of three independent experiments (*n* = 3), and error bars indicate the standard deviation. Source data are provided as a Source Data file.

To assess their functional link, we devised a growth assay using malate as the sole carbon and energy source, a condition in which growth strictly depends on efficient NADH re-oxidation by NADH dehydrogenases^23^. Under these conditions, the Δ*ndh* mutant strain failed to grow, establishing Ndh as the essential NDH-II in *B. subtilis* (Fig. 2). Strikingly, the Δ*yjlC* mutant displayed a similar growth defect, and introducing *yjlC* in *trans* markedly improved growth, although it did not fully reach wild-type levels (Fig. 2). These results indicate that both *ndh* and *yjlC*, the gene encoding the HMP protein, are required for NADH oxidation.

### Interdependent assembly of YjlC and Ndh into a high-affinity NADH dehydrogenase

To understand how Ndh and YjlC cooperate to sustain NADH oxidation, we characterized their molecular association. A 6×His tag was introduced at the N-terminus of YjlC, and the *yjlC–ndh* operon was expressed under the control of an inducible promoter. This construct enabled the co-purification of both proteins, as confirmed by SDS-PAGE (Fig. S3a). In parallel, an N-terminally His-tagged version of Ndh was expressed alone in a Δ*yjlC* mutant. In this context, Ndh could not be purified, suggesting a structural and functional interdependence between the two proteins.

Spectroscopic and biochemical analyses revealed that the purified complex contains both flavin and quinone cofactors. UV–visible spectra showed characteristic flavoprotein absorption peaks at 375 and 450 nm, corresponding to ~0.5 FAD per complex (Fig. S3b). In addition, HPLC coupled to electrochemical detection identified 0.54 molecules of menaquinone-7 (MK-7) per complex, the major quinone in *B. subtilis* membranes (Table S3)^24^. When expressed in *Escherichia coli*, the two proteins also form a complex and co-purified with 0.35 ubiquinone-8 (UQ-8) and 0.1 MK-8 molecules per complex (Table S3), indicating its ability to bind both benzoquinones and naphthoquinones.

Steady-state kinetics of NADH oxidation using DCPIP as an artificial electron acceptor showed Michaelis–Menten behavior with a low turnover number (*k*_cat_ = 27 s⁻¹) but high substrate affinity (*K*_M_^NADH^ = 1.4 µM), yielding a catalytic efficiency (*k*_cat_/*K*_M_ = 19,3 µM⁻¹ s⁻¹) (Fig. S3c). The very low *K*_M_^NADH^ suggests that the YjlC/Ndh complex is adapted to operate efficiently at low NADH concentrations. In contrast, menadione reduction, used as a quinone analog, displayed a *K*_M_ of 62 µM, similar to values reported for NDH-II enzymes from *Caldalkalibacillus thermarum, Staphylococcus aureus* and *Mycobacterium tuberculosis*^17,25,26^ (Fig. S3d).

Strikingly, biophysical analyses revealed that the YjlC/Ndh complex corresponds to unexpected large assemblies. Size-exclusion chromatography coupled with multi-angle light scattering (SEC-MALS) analysis detected oligomeric species around 800 kDa, along with higher-order assemblies reaching up to ~2 MDa (Fig. S3e). Consistently, negative-stain electron microscopy showed that a large fraction of the purified complex self-assembles into fibers of variable length, an intrinsic property also observed upon heterologous expression in *E. coli* (Fig. S3f). These fibers display regularly spaced protuberances along their central axis, consistent with a modular and symmetrical tubular architecture. Taken together, these findings highlight an exceptionally large supramolecular assembly in which multiple NDH-II enzymes are probably bound around an HMP-based tubular scaffold.

### Ndh docks onto a tubular YjlC scaffold, forming supramolecular fibers

**Fig. 3:**
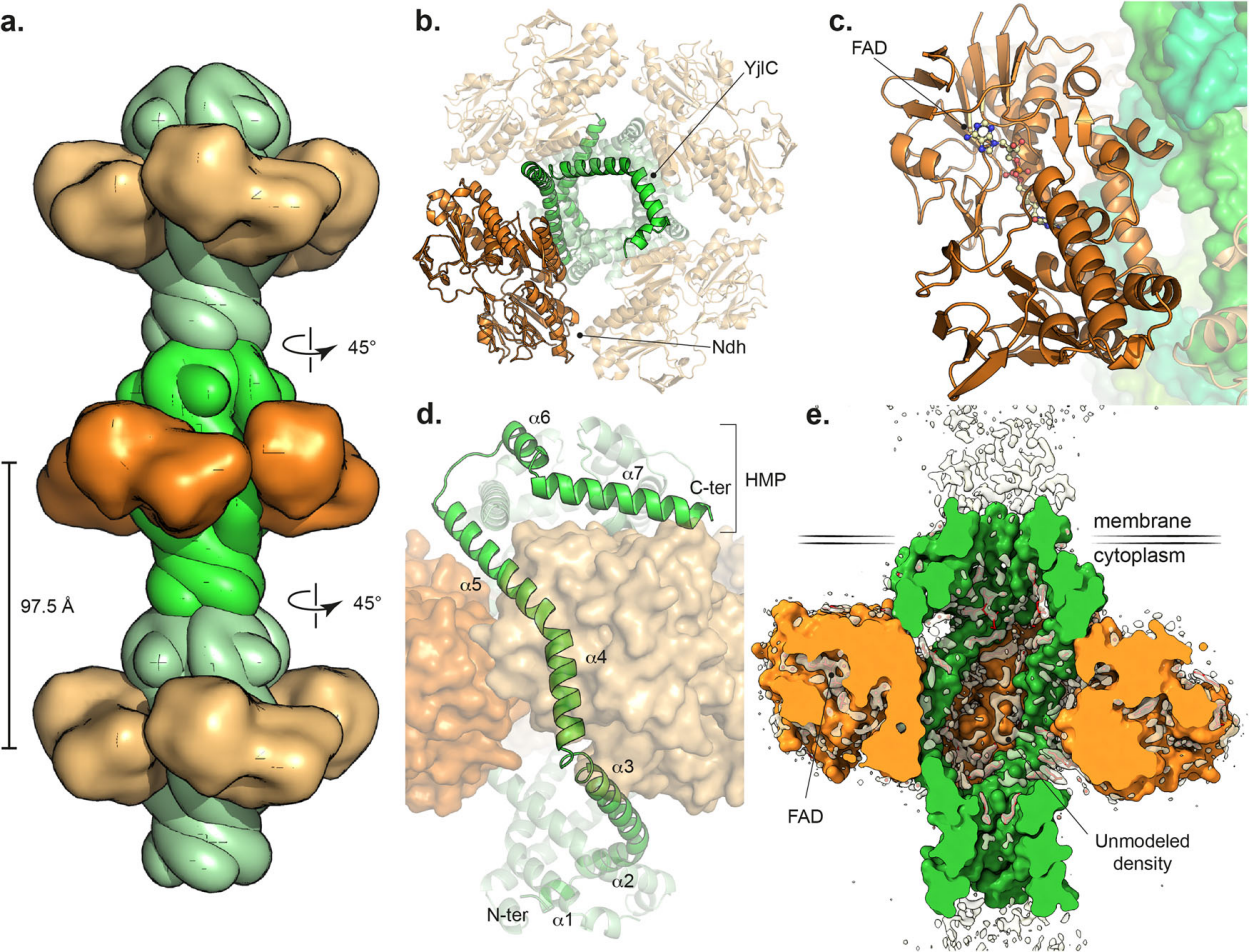
YjlC-Ndh structure. **a** Schematic representation of YjlC-Ndh fiber showing three YjlC_4_–Ndh_4_ units. The low-resolution surface corresponds to a surface representation of the fiber protein model generated with a 10 Å Gaussian filter. **b** Cartoon representation of the YjlC_4_–Ndh_4_. One YjlC monomer is highlighted in green, and one Ndh monomer is highlighted in orange. The other YjlC and Ndh proteins of the complex are colored in light green and light orange, respectively. **c** Close view of one Ndh monomer in cartoon representation. FAD is shown in ball-and-stick representation with carbon, oxygen, nitrogen, and phosphorus atoms colored in light yellow, red, blue, and orange, respectively. **d** Close view of one YjlC monomer in cartoon representation. **e** Sagittal section of YjlC-Ndh cryo-EM reconstruction highlighting the presence of densities inside the tube formed by 4 YjlC and mostly interpreted as lipids. YjlC and Ndh cryo-EM models are displayed in surface and colored in green and orange, respectively. The remaining cryo-EM densities that do not correspond to protein atoms are displayed in semitransparent beige surfaces contoured at 4σ. FAD, modeled lipids, and water molecules are displayed as red sticks.

The structure of the YjlC/Ndh assembly was determined by cryo-electron microscopy at a resolution of 2.6 Å (Fig. 3). The fundamental building block of the fiber is an α₄β₄ hetero-octamer, consisting of four YjlC and four Ndh subunits (232 kDa). These units stack head-to-tail with a translation of ~97.5 Å and a 45° rotation, creating a polarized fiber that can extend to as many as ten α₄β₄ units, as observed in micrograph images (Fig. 3a). At its core, four YjlC subunits assemble into a tetrameric superhelical tube, onto which the four Ndh catalytic subunits are symmetrically arranged in a square array (Fig. 3b–d), an architecture reminiscent of the ForCE and HucSLM complexes^19,22^.

**Fig. 4:**
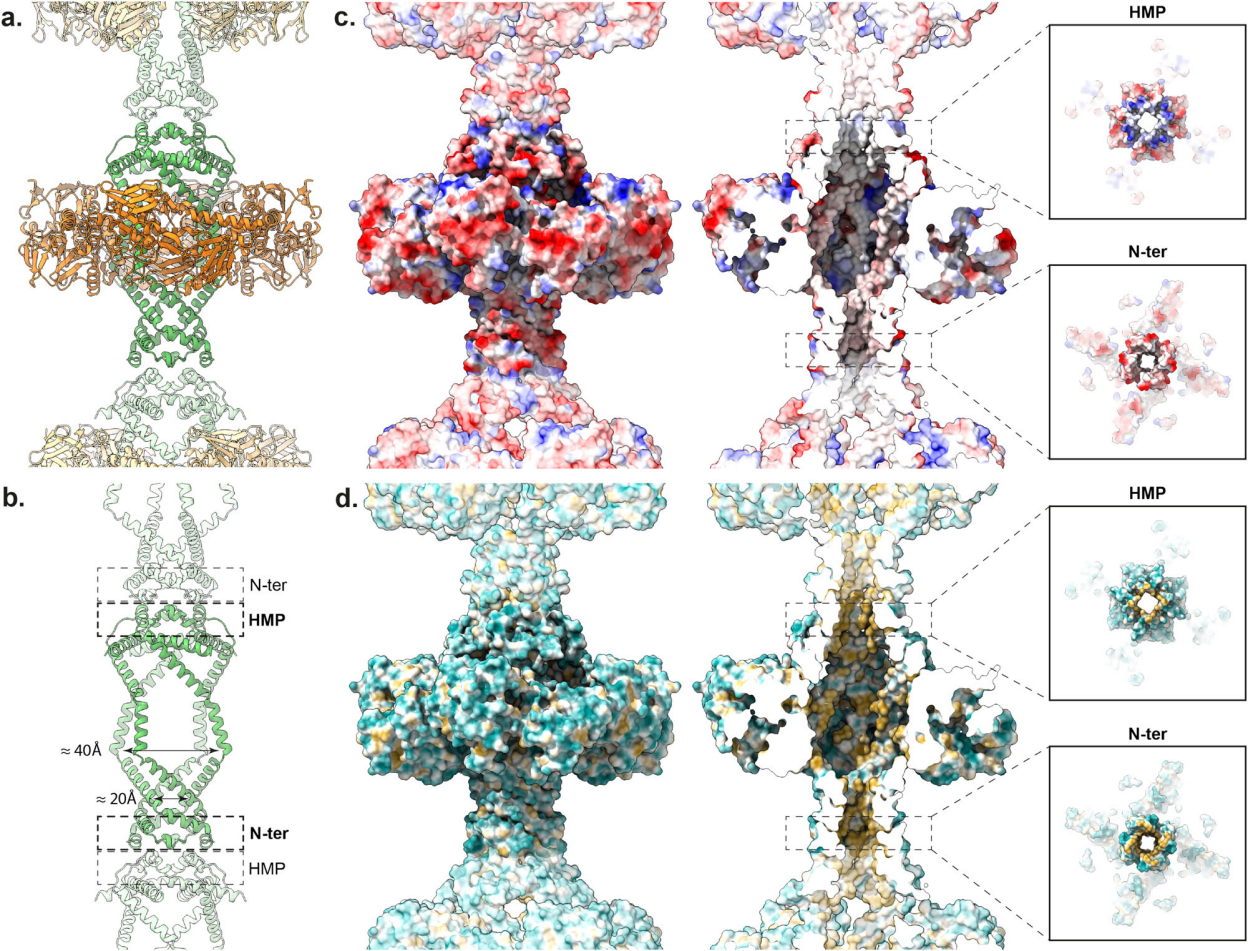
YjlC-Ndh fiber. **a** Cartoon representation of YjlC-Ndh fiber with YjlC and Ndh colored in green and orange, respectively. Unlike the central YjlC_4_–Ndh_4_ octamer, the two others shown here, on the HMP side and the C-terminal side, are colored with lighter hues. **b** Same representation as (**a**) but with only YjlC displayed. The approximate minimum and maximum size of the inner part of the tube created by the YjlC tetramer are indicated. HMP and C-terminal extremities are located with dashed rectangles. **c** Electrostatic surface representation of the YjlC-Ndh proteins. Positively charged amino acids are colored in blue, negatively charged amino acids in red, and neutral residues in white. The cutaway shows the internal surface of the YjlC tube lined with hydrophobic residues. The inserts show the HMP (top view) and N-terminal (bottom view) ends of the YjlC tube. **d** Same representation as (**c**) but showing the hydrophobic surface. Hydrophobic and hydrophilic residues are colored in light orange and light blue, respectively.

The C-terminal helices of YjlC define a membrane-interacting domain corresponding to the HMP module that mediates head-to-tail stacking between octamers (Figs. 3d, e, and 4a, b). This interface is strengthened by charge complementarity, where negatively charged residues on the N-terminal side interact with positively charged residues on the adjacent HMP domain (Fig. 4 c, d). The resulting continuous tunnel extends along the fiber axis, narrowing at ~20 Å wide at the YjlC tetramer core and widening to ~40 Å at the Ndh-binding region (Fig. 4b). The inner surface is predominantly hydrophobic and provides a conduit for molecules such as lipids and quinones (Fig. 4c, d). Features in the cryo-EM map at YjlC-Ndh interfaces suggest the presence of lipid molecules as observed in ForCE^19^ (Fig. 3e). To further investigate this association, mass spectrometry-based lipidomic analyses were conducted on the purified complex. Remarkably, the YjlC/Ndh complex was associated with a large number of lipid molecules, with ~18 moles of lipids per mole of complex, an unusually high level for a membrane-associated protein assembly (Fig. S4a). Comparison with the *B. subtilis* lipidome revealed a striking enrichment in cardiolipin (CL) and its biosynthetic precursor phosphatidylglycerol (PG), which together accounted for ~45% of bound lipids (Fig. S4a). In contrast, CL represents <5% of total lipids in vegetative membranes^27,28^. Both PG and CL fractions were dominated by saturated fatty acid species, primarily C14:0, C15:0, and C16:0, forming specific molecular species such as PG(30:0), PG(31:0), PG(32:0), and CL(60:0,30:0), CL(61:0, 30:0), CL(62:0, 30:0), CL(62:0,31:0), CL(63:0, 31:0), and CL (64:0, 32:0) (Fig. S4b-c). This enrichment in anionic, saturated lipids likely enhances the interaction of the complex with the membrane and may promote quinone diffusion within the hydrophobic tunnel. Consistent with this, the architecture and physicochemical properties of the HMP domain support transient, peripheral membrane contacts, similar to those previously observed in ForCE^19^.

**Fig. 5:**
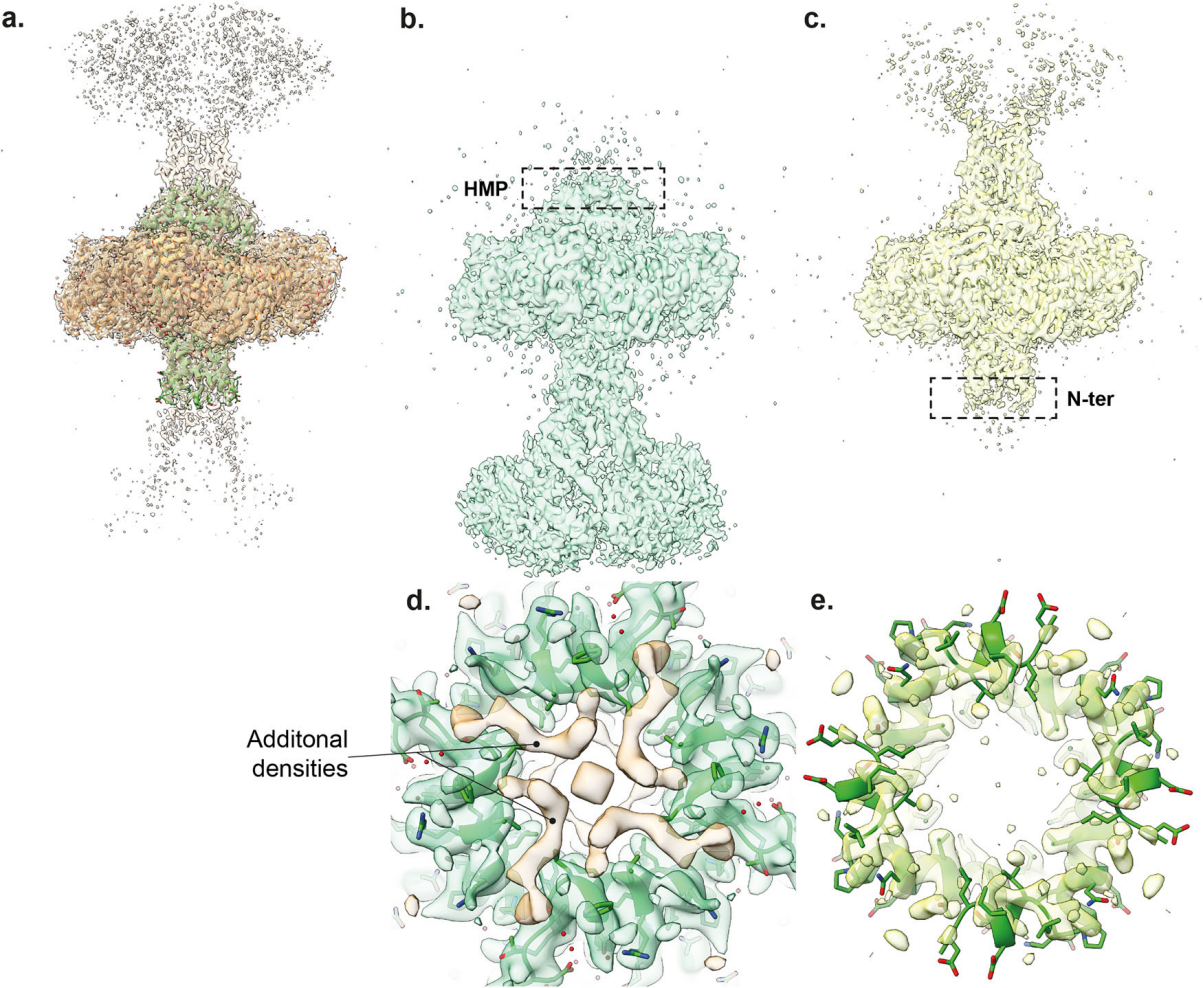
Cryo-EM reconstruction of the fiber extremities. **a** Global view of YjlC-Ndh cryo-EM reconstruction corresponding to an intermediate octamer of YjlC-Ndh fiber. Cryo-EM reconstruction, contoured at 4σ, is displayed as a wheat surface. YjlC and Ndh protein models are displayed as cartoons for the main chain and as sticks for the side chains, with carbons colored green and orange, respectively. **b** Cryo-EM reconstruction of the YjlC-Ndh octamer at the HMP extremity of the fiber (3.61 Å resolution), contoured at 4σ. **c** Cryo-EM reconstruction of the YjlC-Ndh octamer at the N-terminal extremity of the fiber (3.04 Å resolution), contoured at 4σ. **d** Close view of the HMP extremity of the fiber with the protein model of YjlC. Additional densities, by comparison to the reconstruction of the intermediate octamer, are colored in semitransparent orange and are visible on top of the fiber’s HMP extremity, which could correspond to lipids. **e** Close view of the N-terminal extremity of the fiber with the protein model of YjlC.

We next examined whether fiber extremities structurally differ from internal regions. Reconstructions of N- or C-terminal α₄β₄ octamers yielded maps at 3.0 and 3.6 Å resolution respectively, revealing no structural differences compared to octamers within the fiber (Fig. 5a–c). This indicates that the YjlC tube is preorganized for polymerization and fiber formation. At the C-terminal end, an additional density capping the HMP domain is observed, consistent with lipid or hydrophobic molecules, as reported for ForCE^19^ (Fig. 5d). However, such a feature is not observed along the fiber at the interface between each α₄β₄ octamer (Fig. 3e) and at the N-terminal extremity of the fiber (Fig. 5e).

**Fig. 6:**
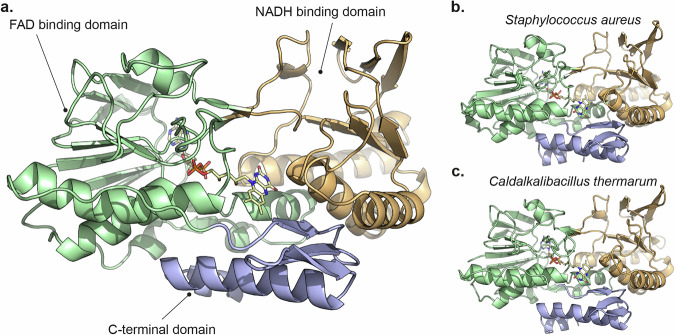
Canonical three-domain architecture of NDH-II enzymes. **a** Cartoon representation of YjlC-Ndh from *B. subtilis* with FAD-binding domain (residues 1-109 and 259-340) colored in green, NADH-binding domain (residues 110-258) colored in beige, and C-terminal domain (residues 341–392) in blue. FAD is represented by sticks, with the carbon, oxygen, nitrogen, and phosphorus atoms colored light yellow, red, blue, and orange, respectively. **b** Same representation as (**a**) for NDH-II from *Staphylococcus aureus* (PDB code 5NA1). **c** Same representation as (**a**) for NDH-II from *Caldalkalibacillus thermarum* (PDB code 4NWZ).

**Fig. 7:**
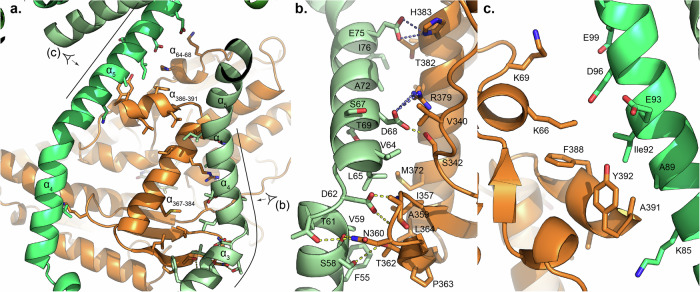
Two distinct interfaces mediate Ndh docking onto the YjlC scaffold. **a** Global view of Ndh interacting with two YjlC proteins. Ndh and YjlC are depicted as cartoons and colored orange and green, respectively. The side chains of the residues involved in the interaction between the Ndh and YjlC proteins, identified with PISA^29^, are shown as sticks. **b,** **c** Focused views of the two main zones of interaction. Hydrogen bonds and ionic interactions are represented by yellow and blue dashed lines, respectively.

The Ndh subunit adopts the canonical three-domain architecture of NDH-II enzymes, as confirmed by structural comparison with NDH-II homologs from *C. thermarum* (PDB 4NWZ)^17^ and *S. aureus* (PDB 5NA1)^25^, with RMSDs of 0.74–0.95 Å (Fig. 6). The first Rossmann-like domain (residues 1-109 and 259-340) binds FAD (Fig. S5a), while the second Rossmann-like fold (residues 110-258) contains the NADH-binding site (Fig. S5b). The C-terminal domain (residues 341-392) consists of an antiparallel β-sheet followed by two amphipathic helices forming a helix–turn–helix motif, which mediates inter-protomer interactions within the tube (Fig. 6, Fig. 3b, c, Fig. 7).

Although MK7 co-purified with the complex (Table S3), no discrete density attributable to quinone was observed, likely due to conformational heterogeneity or multiple transient binding sites. Molecular docking calculations consistently positioned MK7 within an internal Ndh cavity, with the headgroup near the FAD isoalloxazine ring and the isoprenyl tail extending into the YjlC-defined tunnel (Fig. S5c, d). This cavity corresponds to quinone-binding sites of monomeric NDH-II enzymes^15,18^. The predicted insertion of the isoprenyl tail into the tunnel parallels ForCE and HucSLM, where quinones bridge the catalytic center to the membrane via the HMP scaffold.

We next analyzed the interface between Ndh and the YjlC scaffold using PISA^29^. The contact area (~850 Å², ΔG_diss ~9 kcal/mol) is primarily hydrophobic with additional polar interactions (Fig. 7). Although its molecular composition resembles that of ForCE and HucSLM^19,22^, the smaller buried surface likely reflects differences in size and oligomeric state of the associated oxidoreductases: a single monomer in Ndh and ForC versus a HucS₂L₂ hetero-tetramer in HucSLM. In YjlC/Ndh, two distinct regions of Ndh mediate the interaction with the tubular scaffold (Fig. 7). The primary interface (ΔG_diss ~6 kcal/mol) involves one α-helix and two β-strands from the Ndh C-terminal domain contacting helices α_3_ and α_4_ of a single YjlC monomer, and is stabilized by a network of hydrophobic contacts, hydrogen bonds and ionic interactions (Fig. 7b). A secondary, weaker interface (ΔG_diss ~3 kcal/mol) occurs between helices α_64-68_ and α_386-391_ of the Ndh C- and N-terminal domains and helix α_5_ of another YjlC monomer, relying exclusively on hydrophobic contacts (Fig. 7c). Together, these two interfaces likely enhance both the specificity and stability of the complex. These interactions are in full agreement with previous DSSO cross-linking data from the *B. subtilis* proteome that detected multiple YjlC-Ndh contacts^30^. Strikingly, the C-terminal region involved in this interaction coincides with the domain that monotopic NDH-II enzymes use to anchor to membranes, as seen in the *M. smegmatis* cryo-EM structure^31^. This suggests that, in *Bacillales*, the C-terminal region has been repurposed from direct membrane binding to mediate docking onto the tubular HMP scaffold, which now serves as an adaptor between the enzyme and the membrane.

### Evolutionary signatures reveal how Ndh recognizes its YjlC partner

**Fig. 8:**
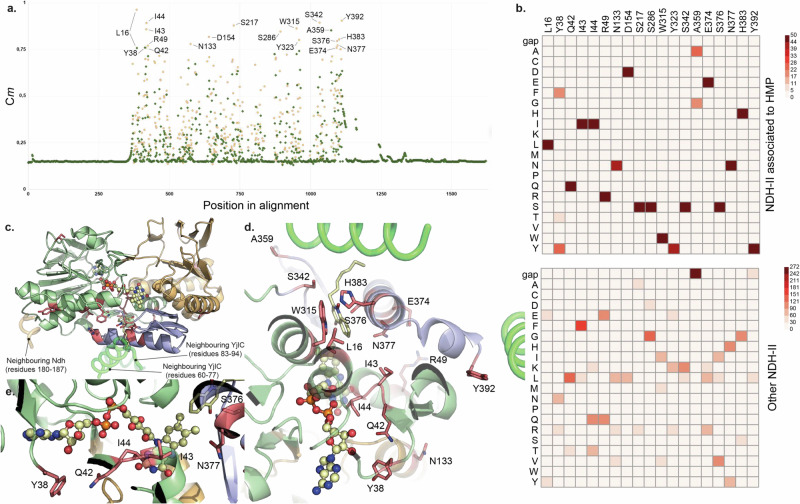
Sequence signatures distinguishing HMP-associated NDH-II highlight regions for scaffold docking and cofactor binding. **a** Plot of Cm (maximal Jaccard Coefficient) values along the alignment. Orange circle: Cm values computed using 20 amino-acids; green square: Cm values computed using the Dayhoff 6 categories (AGPST, C, FWY, HRK, MILV, NDEQ). Sites with a Cm value higher than 0.75 are indicated, with the numbering of *B. subtilis* NDH-II sequence.** b** heatmap of occurrence of amino-acids in sites highlighted in (a). **c** Mapping of specific sites highlighted in (a) on the structure of *B. subtilis* NDH-II (red and stick). **d**,** e** Focus on residues located near the contact interface between HMP and Ndh and located in the pocket containing quinone and flavine.

To identify the molecular features underlying the specific recruitment of HMP protein by NDH-II in *Bacillales*, we compared NDH-II sequences located next to HMP genes (“HMP-associated NDH-II”) with those found in genomes lacking an HMP neighbor. For each position in the multiple sequence alignment, we computed a residue-specificity score (Cm; see Methods) that integrates both amino acid identity and physicochemical class (Fig. 8a). Nineteen high-Cm sites were strongly conserved among HMP-associated NDH-II while varying substantially in other NDH-II enzymes (Fig. 8b). When mapped onto the *B. subtilis* Ndh structure, these positions only grouped into two distinct regions (Fig. 8c–e). The first group of positions (11/19 sites) lies within the two C-terminal α-helices and the adjacent region, consistent with their expected role in mediating the interaction with the YjlC scaffold (Fig. 8d). Notably, four of these sites are located precisely at the YjlC-Ndh interface in the *B. subtilis* complex, directly contributing to HMP scaffold binding (Ser342, Ala359, His383 and Tyr392) (Fig. 7). The second group (4/19 sites) lines the predicted quinone channel near the FAD site (Fig. 8d, e). Together, these two sets of residues define a minimal signature for docking onto the HMP tube.

To test whether these sequence features specify interaction with YjlC, we co-expressed YjlC with NDH-II from *S. aureus* (*Sa*Ndh), an enzyme encoded in a genome that lacks any HMP protein. Surprisingly, *Sa*Ndh co-purified with YjlC when produced in *E. coli*, as shown by SDS-PAGE (Fig. S6a). However, unlike the native *B. subtilis* complex where YjlC and Ndh co-purify in near-equimolar amounts, the *Sa*Ndh–YjlC complex displayed a lower relative intensity of the *Sa*Ndh band compared to YjlC. This imbalance persisted after size-exclusion chromatography (SEC), indicating that the association is weaker and more labile (Fig. S6b, c). Despite this reduced stability, SEC profiles revealed that *Sa*Ndh and YjlC can still assemble into high-molecular-weight species, similar to the Ndh/YjlC complex (Fig. S6b). Mass spectrometry confirmed the presence of both proteins, alongside minor *E. coli* contaminants with the *Sa*Ndh/YjlC complex (Fig. S6d, e). Together, these data indicate that the sequence determinants identified through evolutionary analysis are sufficient to permit cross-species association.

## Discussion

Our findings highlight a lineage-specific trait in *Bacillales*, where NDH-II recruited an HMP partner via a single evolutionary event. In *B. subtilis*, both Ndh and the HMP protein YjlC are essential for NADH oxidation. The cryo-EM structure reveals that Ndh docks onto the HMP tube via its C-terminal region, repurposing the domain that monotopic NDH-II typically uses for direct membrane anchoring, and assembles into supramolecular fibers of up to ten α₄β₄ units. Structural analysis identified the residues forming this interface, some of them being specifically found in HMP-associated NDH-II. Consistent with this, experiments using a non-cognate NDH-II from *S. aureus* showed that interaction with an HMP protein can still occur, albeit weaker. This indicates that sequence modifications in *Bacillales* NDH-II primarily enhance, rather than strictly permit, binding to the HMP scaffold. Such flexibility raises the possibility that other oxidoreductases could also interact with HMP tubes through promiscuous binding. Overall, *Bacillales* NDH-II evolved an alternative strategy for membrane association and quinone pool connectivity, diverging from the classical monotopic paradigm.

The evolutionary origin of this system likely involved the recruitment of an HMP protein previously associated with SQR. Given the close structural similarity between SQR and NDH-II (RMSDs of 2.5–3.8 Å)^18^, only limited adaptations would have been needed for the HMP scaffold to accommodate NDH-II as a partner. This scenario explains how a single event gave rise to the distinctive *Bacillales* association between NDH-II and an HMP scaffold.

Our study expands the repertoire of HMP-based assemblies by introducing a third type of oxidoreductase organized around a tubular scaffold. Strikingly, this conservation extends beyond enzyme docking and also includes quinone positioning (Fig. S7). In ForCE, MK7 binds within a V-shaped pocket formed by two amphipathic helices of ForC (residues 223–257)^19^. In HucSLM, MK9 occupies a related site at the interface between the catalytic subunit HucS (residues 298–311) and the scaffold-forming HMP protein HucM (residues 63–78)^22^. In YjlC/Ndh, MK7 is predicted to bind in an analogous V-shaped pocket formed by helix 367–383 of Ndh and helix 62–78 of YjlC (Fig. S7). Despite sequence divergence, these systems converge on a shared structural solution, precisely positioning the quinone at the interface between the catalytic enzyme and the membrane-anchoring scaffold.

These findings highlight a broad evolutionary theme in which hydrophobic tunnels serve as versatile platforms for membrane-associated processes. In HMP assemblies, such conduits mediate the reversible exchange of quinones between the membrane pool and cytoplasmic enzymes^19,22^. Bridge-like lipid transport proteins (BLTPs) such as LPD-3 use hydrophobic channels to mediate directional transfer of bulk lipids between membranes, likely guided by ionizable residues that line one side of the tunnel^32^. Thus, evolution has repeatedly converged on comparable structural designs, confined hydrophobic tunnels, while adapting them to distinct biological functions: reversible quinone circulation in HMP-based oxidoreductases versus vectorial lipid transfer in BLTPs.

Beyond channeling quinones, the tubular architecture may also offer functional advantages. By clustering several catalytic units around a single HMP scaffold, the assembly drastically reduces membrane surface occupancy, from ~84 nm² for four individual Ndh enzymes to ~16 nm² for the corresponding hetero-octamer unit. For comparison, Complex I, which catalyzes the same NADH oxidation reaction, occupies ~72 nm² of membrane surface^1,33^. In addition, the dimensions and physicochemical properties of the lumen, which behaves as an extension of the membrane, confine quinone turnover, thereby enabling efficient electron transfer while limiting unwanted reactions with molecular oxygen. These functional implications remain hypothetical and warrant future investigation.

In conclusion, our work reveals how a tubular scaffold was co-opted within *Bacillales* to expand the organizational and functional landscape of NDH-II enzymes. These findings suggest that similar architectural principles may underlie yet undiscovered systems, providing a framework to explore how hydrophobic tunnels have shaped the evolution of membrane-associated electron transfer pathways.

## Methods

### Strain construction

The *B. subtilis* 168 (*trpC2*) Δ*yjlC* Δ*ndh* mutant was engineered through allelic replacement using a kanamycin resistance cassette. Complemented strains were constructed by integrating either p*ndh* or p*yjlC* at the *amyE* locus. Strain 4355 was generated by integrating, at the native *yjlC* locus, a spectinomycin resistance cassette along with a fragment carrying the 6×CAT-*yjlC* gene placed under the control of a xylose-inducible promoter. For *E. coli*, the ∆*ndh::kan* allele from strain BW25113 *∆ndh::kan* was transferred into Shuffle® T7 by P1 transduction, followed by the excision of the kanamycin cassette using plasmid pCP20. The resulting Shuffle® T7 ∆*ndh* strain was subsequently transformed with either pET28a–*yjlC*–6×CAT–*ndh* or pLB6. All strains are verified by PCR and are listed in Table S4.

### Plasmid construction

Plasmids employed in this study were generated by PCR amplification of the specified fragments, followed by restriction digestion and assembly using either a ligation-free cloning kit (abm) or T4 DNA ligase (NEB). Inserts were cloned into pSPH1, pET28a, or pET14a backbones under the control of either xylose or T7 promoters. Primer numbers, DNA templates, restriction sites, and assembly strategies are detailed in Table S4. All plasmids were verified by sequencing.

### Media and culture conditions

*B. subtilis* 168 and its derivatives were cultured overnight at 37 °C in Luria-Bertani (LB) medium. The precultures were diluted to an OD₆₀₀ of 0.05 in CE medium^34^, supplemented with 0.1 mM tryptophan, 30 mM malate, and 0.1% (w/v) xylose, when specified. The cultures were incubated for 6 h, after which they were diluted to an OD₆₀₀ of 0.1 in CE medium containing 2% (w/v) xylose, as indicated. For growth monitoring, 100 µL aliquots were incubated in a microplate reader at 37 °C with shaking at 180 rpm for 6 h.

For recombinant protein production, *B. subtilis* 168, *E. coli*, and their derivatives were cultured overnight in LB at 37 °C. The precultures were diluted to OD₆₀₀ of 0.05 in LB and grown to mid-exponential phase (OD₆₀₀ = 0.4–0.6). Protein expression was induced with 0.8% (w/v) xylose (*B. subtilis*) and 1 mM IPTG (*E. coli*) for 4 h. The cells were harvested, washed once with 50 mM Tris-HCl (pH 8.0), and stored at -80 °C.

### Purification procedures

All steps were performed at 4 °C using an ÄKTA FPLC system (Cytiva). Frozen cells (*B. subtilis* and *E. coli*) were thawed and resuspended in 15 mL 50 mM of Tris-HCl (pH 8), supplemented with a protease inhibitor cocktail (Roche). For *B. subtilis*, lysozyme (1 mg/mL) and DNase I were added, and the suspension was incubated for 30 min at 37 °C, followed by quenching with 0.5 mM EDTA. Cell disruption was achieved using a French press, and cell debris was removed by centrifugation at 11,000 × *g* for 30 min. The resulting supernatant was adjusted to contain 150 mM NaCl and 20 mM imidazole, and loaded onto a 5 mL Ni-NTA column (Cytiva). After washing with a buffer containing 50 mM Tris-HCl, 150 mM NaCl, and 20 mM imidazole, a second washing step was performed with 50 mM imidazole, and the protein was eluted using 200 mM imidazole. Imidazole was removed by buffer exchange using a 100 kDa Amicon (Millipore). The purified protein was supplemented with 5% glycerol, flash-frozen in liquid nitrogen, and stored at -80 °C. For size-exclusion chromatography (SEC) purification, 25 µL of YjlC/Ndh (3 mg/mL) and 25 µL of YjlC/*Sa*Ndh (4.8 mg/mL) were loaded onto a Superdex 200 5/150 GL (Cytiva) column equilibrated with Tris 50 mM (pH 8) NaCl 150 mM at a flow rate of 0.15 mL.min^-1^. Fractions 1 to 6 were analyzed by SDS-PAGE.

### Activity assays and kinetic analysis

NADH dehydrogenase activity was measured at room temperature in an anaerobic chamber using an AvaSpec-2048L spectrophotometer. For NADH-DCPIP assays, reactions were carried out in 50 mM Tris-HCl (pH 8), 150 mM NaCl, containing 1 mM DCPIP and varying concentrations of NADH (0.2-50 µM). DCPIP reduction was monitored at 600 nm (ℇ_600_ = 21.000 M^-1^ cm^-1^). For NADH:menadione assays, reactions were performed in the same buffer with 50 µM NADH and varying menadione concentrations (5–250 µM). NADH oxidation was monitored at 340 nm (ℇ_340_ = 6.220 M⁻¹cm⁻¹). One unit of activity was defined as the amount of enzyme catalyzing the oxidation of 1 µmole of NADH or the reduction of 1 µmole of menadione per minute per mg of enzyme. Kinetic parameters were determined by fitting the data to the Michaelis-Menten equation using OriginPro.

### Quinone extraction and analysis

Quinones were extracted from solutions of purified NDH-II and analyzed as described^35^.

#### FAD quantification

The FAD content of the purified complex was determined by UV-visible spectroscopy. Absorbance at 450 nm was converted to FAD concentration using the molar extinction coefficient of oxidized FAD (ℇ_450_ = 11,300 M⁻¹ cm⁻¹)^36^, and the FAD-to-protein ratio was calculated by comparison with independently measured protein concentrations.

#### SEC-MALS analysis

50 µL at 8.5 mg/mL of YjlC/Ndh was applied to a Superdex 200 10/300 Increase column (Cytiva) equilibrated with 50 mM Tris-HCl (pH 8), 150 mM NaCl at a flow rate of 0.6 mL.min^-1^ and analyzed as described^19^.

### Mass spectrometry based-Lipidomic analyses on purified protein

Total lipid was extracted from the 10 µL of a 6.25 mg/mL purified protein sample or from two negative control proteins that do not bind lipids (namely, catalase 1 and catalase 2). The extraction process utilized 350 µL chloroform/methanol (4:3 v/v) containing 1 nmol each of internal standards (as listed in Table S6), followed by the addition of 140 µL of water to induce biphasic separation. The lower organic phase was collected and subsequently dried in a vial insert. The recovered lipids were dissolved in methanol (50 µL) and aliquot (10 µL) was injected to a Agilent 1290 infinity/Infinity II LCMS system equipped with ZORBAX Eclipse Plus C18, 100 ×2.1 mm, 1.8 um reversed-phase column (Agilent) with Infinity II inline filter, 0.3 um (Agilent) maintained at 45 C. Samples were separated by the change in gradient of solvent A (water:acetonitrile:isopropanol, 5:3:2 (v/v/v), 10 mM ammonium formate) and solvent B (isopropanol:acetonitrile:water, 90:9:1 (v/v/v), 10 mM ammonium formate). The gradient was as follows, starting with a flow rate of 0.4 mL/min at 15% B and increasing to 50% over 2.5 min then to 57% at 2.6 min, then to 70% over 9 min and to 93% at 9.1 min, then to 96% over 11 min and to 100% at 11.1 min and hold to 12 min then back to 15% at 12.2 min to 16 min (total of 16 min). MS (Agilent 6495c triple quadrupole) was operated in for targeted analysis with DMRM (dynamic multiple reaction monitoring) with 650 ms of cycle time with following setting: Agilent Jet stream ion source with positive and negative switching with gas temperature at 150 °C, drying gas (nitrogen) 17 L/min, Nebulizer gas 20 psi, Sheath gas temperature at 200 °C with flow at 10 L/min, capillary voltage 3500 V for positive and -3000 V for negative mode, nozzle voltage at 1000 V for positive and -1500 V for negative mode. As described in ref. ^37^, for targeted lipidomic analysis in *B. subtilis*, first, collision energy (CE) and characteristic fragmentation for Lysil-phosphatidylglycerol (LyPG), was obtained by Mass Hunter Optimizer software (Agilent) using authentic standard LyPG C18:1 (Sigma). Then, the fatty acid combination for each lipid species was confirmed by *B. subtilis* total lipid using neutral loss ion scan (NL m/z 35 for DAG with CE 35 V; m/z 141 for LPE and PE with CE 19 and 17 V, respectively; m/z 300 for LyPG with CE 41 V; m/z 189 for PG with CE 19 V, m/z 115 for PA with CE 13 V in positive mode). For CL, PG species detected as above was further used to determine CL species in *B. subtilis*. The resulted LCMS data was combined to pre-existing dynamic multiple reaction monitoring (DMRM) method for optimized targeted lipidomic analysis for *B. subtilis* (as shown in Table S6).

### Negative-stain electron microscopy

5 μl drops of the sample were placed directly on glow-discharged carbon-coated grids (EMS) for 3 minutes. The grids were then washed quickly with two drops of 2% aqueous uranyl acetate and stained with a third drop for 1 min. Grids were dried on filter paper, and the samples were analyzed using a Tecnai 200 kV electron microscope (Thermo Fisher Scientific), and digital acquisitions were made with a numeric camera (Oneview, Gatan) at a nominal magnification of 50,000, yielding a 2.1 Å pixel size. Images were recorded automatically using the Latitude S software (Gatan).

### Mass spectrometry

Purified proteins were subjected to SDS-PAGE, and the stacking gel band was analyzed as described^35^ with the following modifications. Spectra were processed by Proteome Discoverer software (Thermo Scientific, version 3.0) and searched against the *E. coli* K12 database (Taxonomy ID: 83333, downloaded from the NCBI by Protein Center, including 4516 entries). Proteins were filtered to retain only those with a minimum of five peptide spectral matches. For His-YjlC, the sequence used corresponded to YjlC with an N-terminal extension consisting of Met-Ala followed by a hexahistidine tag (Met-Ala-His_6_). The mass spectrometry proteomics data have been deposited to the ProteomeXchange Consortium via the PRIDE partner repository with the dataset identifier PXD069025.

### Phylogenetic and genomic analysis

The protein database assembled in Garcia et al. 2022^38^ has been used to perform the analysis and covers the diversity of prokaryotes. Briefly, the genomes have been first downloaded from the FTP of the NCBI and several steps of sampling have been performed using taxonomy, whole genome comparison, and similarity-based clustering. The quality of genomes has been assessed to select a set of genomes that represent each cluster. For this, the number of proteins annotated as reviewed in Uniprot, the NCBI completeness status, the NCBI representative status, and the availability of annotation files have been used. The database contains 9596 bacterial proteomes and 1268 archaeal genomes (Table S1).

We identified the homologs of NDH-II using a collection of representative NDH-II sequences collected from^21^. This dataset has been aligned using MAFFT v7.419^39^ (auto option) and has been used to build an HMM profile using HMMER v3.2.1^40^ (HMMbuild). From this profile, we searched for homolog sequences by using HMMsearch. Sequences presenting an e-value < 1e-20 were considered as homologs (5240 sequences, Source data). The sequences were aligned using MAFFT v7.419^39^ (auto option), and the alignment has been trimmed using BMGE v1.12 (entropy threshold=0.95, minimum length=1, matrix=BLOSUM30)^41^. A preliminary phylogeny has been inferred using FASTTREE v2.1.10 (LG + G4)^42^. NDH-II and SQR families have been delineated using previous literature^21^, mapping of already characterized sequences, tree topology, and phylogenetic distance.

For HMP, we first performed a BLASTP v2.8.1 + ^43^, using 9 sequences of HMP sequences representing 9 major groups that have been previously described^19^, with an e-value threshold of 0.01. From this method, we gathered 531 homologous sequences. We then aligned these sequences, curated manually the alignment and used it to build a HMM profile. Using HMMsearch, we gathered 1080 sequences. We realigned these sequences, manually curated it and selected the C-terminal domain that is conserved. We reinterrogated the database using HMMsearch. We reiterated this procedure 3 times, resulting in a dataset of 1105 sequences. The sequences have then been clustered using MMSEQS2 v14-7e284 (e-value threshold = 0.001, coverage = 0.05, mode = greedy incremental). The sequences of the 30 more represented out of 70 families have been independently aligned, used to build HMM profiles and have been again used to interrogate the database using HMMsearch (e-value < 0.01). The 1122 resulting sequences have then been pooled with the 1105 sequences. This dataset has been aligned and manually curated, providing a final dataset of 1136 sequences (Source data).

For FDH, we used a simple BLASTP v2.8.1 + ^43^, using *Methanobacterium formicicum* FDH as a seed, with an e-value threshold of 1e-20. For [Ni-Fe] hydrogenases, we used several HMM profiles available on NCBI and TIGRFAMs databases, covering several hydrogenases and homologs (large subunit: EchE, EhaO, EhbN, FrhA, MbhL, MbxL, MvhA, NuoD, VhtA and small subunit: EchC, EhaN, EhbM, FrhG, MbhJ, MbxJ, MvhG, NuoB, VhtG). These profiles have been used to perform HMMsearch, with an e-value < 1e-20. The datasets have then been pooled for the small and for the large subunit.

To identify the gene families that present a moderate conservation in terms of genomic vicinity with NDH-II, we performed a comparative genomic analysis. First, we build homologous protein families using MMSEQS2 v14-7e284 (e-value threshold = 0.001, coverage = 0.8, mode = greedy set cover) on the full database, to get the pangenome of prokaryotes. We then searched for families that could be associated with NDH-II, using the genomic proximity and the gene orientation as proxies. We thus searched for the families with the highest number of sequences located close to NDH-II genes (50 bp maximum) and with the same orientation. The 3 families with the highest number were a polyprenyl synthetase (62 occurrences), HMP (46 occurrences), and a peptidase S8 (43 occurrences).

We then mapped the taxonomic distribution of NDH-II, SQR, and HMP on the species phylogenies of Bacteria and Archaea. The phylogenetic trees correspond to the database of Garcia et al. 2022^38^. Both trees have been inferred using IQTREE v.1.6.10^44^ with mixture model (LG + C60 + PMSF) and the bacterial tree is based on a concatenation of RpoB, RpoC and IF2 while the archaeal tree on concatenation of 30 ribosomal proteins. For the phylogenetic analysis of NDH-II and HMP, a taxonomic subsampling has been performed. We first selected the subsample described in Garcia et al. 2022 of 551 representative genomes, covering the diversity of bacterial phyla and major archaeal clades. In addition and to increase the diversity in the gene phylogenies, we performed a clustering using TREECLUSTER^45^ (distance threshold = 3.5 for NDH-II and 4 for HMP). For each cluster, a representative sequence has been chosen, using several criteria based on the quality of the genome from the sequence originated: the number of proteins annotated as reviewed in Uniprot, the completeness status of the genome (Complete > Chromosome > Scaffold > Contig) and the presence of native GFF annotation files on the NCBI FTP. For each selected sequence, the associated genome has been added to the subsampling list, if not already present, with a final list of 630 genomes in total (Table S1). The sequences from the datasets of NDH-II (292 sequences) and HMP (200 sequences) that correspond to this list have been extracted, realigned using MAFFT-LINSI (Source data). For NDH-II, 26 sequences of SQR have been added in the dataset to use as an out-group and the sequence of *Thermus thermophilus* has been manually added (it is absent from.faa file of the genome). The corresponding alignments have been manually curated (removal of incomplete and divergent sequences) and trimmed using BMGE v1.12 (entropy threshold=0.95, minimum length=1, matrix=BLOSUM30)^41^. The trimmed alignments have been used to infer phylogenies using IQ-TREE v2.2.2.6^44^ (Source data). The best-suited homogeneous model has been selected using TESTNEW, based on the Bayesian Information Criteria. The robustness of branches has been assessed using 1000 ultra-fast bootstrap replicates. The figures have been generated using ITOL^46^. The genomic context has been mapped on the phylogenies using GENESPY v1.2^47^.

For the identification of specific residues present in the NDH-II associated to HMP and different in other NDH-II, we used the alignment of NDH-II (292 sequences), with 46 additional HMP-associated NDH-II to enrich the diversity (Source data). The sequences have been aligned using MAFFT-LINSI. We used a method used in ref. ^48^. Briefly, for each position of the alignment *p* and for each possible amino-acid *i*, we computed the Jaccard coefficient *C* of the HMP-associated sequences and the set of sequences having the amino-acid *i*. The maximal value among all amino-acid corresponds to the maximal coefficient *Cm* as:

$${{Cm}}_{p}=\left\{\frac{|G\cap {S}_{i}|}{|G\cup {S}_{i}|}\right\}=\left\{\frac{|{G}_{i}|}{|{G}_{i}+{G}_{i}+{G}_{i}|}\right\}$$

With p the position in the alignment, i the amino-acid, G the set of all amino acids at position p from the group of tested sequences, G_i_ the set of amino acid i at position p from the group of tested sequences, S_i_ the full set of amino acids i. The score has also been computed using the 6 Dayhoff categories. The values higher than 0.75 have been retained.

### Cryo-EM grid preparation

Procedure derived from ref. ^49^. QUANTIFOIL Cryo-EM grids Au 300-mesh, R 2/1 were glow-discharged for 45 s at 30 mA under low vacuum. Then, 4 μl of the protein sample at 6.25 mg ml^−1^ were applied to the grid, which was subsequently blotted and flash-cooled in liquid propane. The Vitrobot parameters were set at 22 °C, 100% humidity, 2 s blotting time and blot force 1.

### Cryo-EM data acquisition and image processing

YjlC-Ndh data were collected using the ESRF CM02 Titan Krios G4 electron microscope equipped with cold FEG, a Selectris X energy filter and a Falcon 4i electron detector (Thermo Fisher Scientific), and operated at 300 kV with a nominal magnification of 215,000x (corresponding to 0.58 Å/pixel at the specimen level) and a defocus range of −0.5 to −2.9 μm. A series of 43,216 movies, each containing 1008 frames, were collected with a total dose of 40 e^-^/Å^2^ (Table S5). YjlC-Ndh reconstruction was performed using cryoSPARC (version 4.5.1)^50^ (Fig. S8). Each movie was first drift-corrected for full-frame motion and sample deformation before the contrast transfer function (CTF) was estimated. A blob picking job was first used on 1000 micrographs to select 156,294 particles, which were then sorted using 2D classification jobs. The two best 2D classes, corresponding to 11,276 particles, were used as templates for a template-picking job on 2000 micrographs, resulting in the selection of 426,514 particles. Next, 2D classification jobs selected 18,036 particles corresponding to the seven best classes. These particles were then used as templates for a final template picking step on the full dataset. A total of 4,070,805 particles were extracted and 2D sorted. The best 2D-classes obtained from 1,064,325 particles were selected and used to calculate three ab initio three-dimensional models, which were subsequently used for the first step of particle curation via a hetero refinement job with C1 symmetry. The particles corresponding to the two best reconstructions (835,105 particles) were used for a second round of particle curation. Five ab initio three-dimensional models were calculated and used in a hetero refinement job using C1 symmetry. The particles corresponding to the two best reconstructions (691,400 particles) were kept and used to calculate an initial 3D reconstruction using a homogeneous refinement procedure applying C4 symmetry. This led to a 2.68 Å resolution map, which was sharpened with a B factor of −127.1 Å². Next, reference-based motion correction was performed, leading to the 2.59Å resolution final map (C4 symmetry),using the Fourier shell correlation (FSC) criterion of 0.143, and was sharpened with a B factor of −130.1Å². The local resolution of the reconstruction was estimated using the cryoSPARC procedure (Fig. S9).

### Cryo-EM image processing of fiber’s extremities

HMP and N-terminal extremities reconstructions were performed using CryoSPARC version 4.7.1^50^ and Topaz version 0.2.5a^51^. The same cryo-EM dataset was used for this analysis as for the initial global reconstruction. To identify and visualize particles corresponding to Ndh filament extremities, 3588 terminal-end particles were manually picked from 1025 curated micrographs, without prior knowledge of whether they represented the N-terminal or HMP extremities (Fig. S10). They were then used to train a Topaz deep-learning model to recognize and select fiber extremities. The trained model was then applied to the full dataset for automated particle picking. The resulting picked particles were visually inspected to confirm that the model was accurately identifying only fiber extremities. This procedure allows the selection of 42,508 particles that were subsequently subjected to 2D classification. All resulting 2D classes were retained for further processing and then used to calculate an ab initio three-dimensional model with C1 symmetry. This was used as a starting model of a non-uniform refinement with C4 symmetry, resulting in an initial map with a resolution of 3.54 Å. In order to sort the particles corresponding to the 2 fibers extremities a heterogeneous refinement with C4 symmetry was carried out. As input, the non-uniform refinement reconstruction volume was provided along with two additional modified versions of the previously obtained reconstruction: one simulating a N-terminal extremity and another simulating a HMP extremity of the fiber. The particles corresponding to the two best reconstructions were kept and used to calculate two final non-uniform reconstructions. The first one, corresponding to the N-terminal extremity (37,373 particles) resulted in a map with a final resolution of 3.04 Å (FSC = 0.143), that was sharpened with a B factor value of -104.1 Å². The second one, corresponding to the HMP extremity (4133 particles), yielded a map with a final resolution of 3.61 Å (FSC = 0.143), which was sharpened with a B factor value of −61.8 Å². The local resolution of the reconstruction was estimated using the cryoSPARC procedure.

### Model building in the cryo-EM maps

An Alphafold3^52^ model of the *Bacillus subtilis* YjlC-Ndh hetero-octamer was used as the initial model and was fitted into the cryo-EM density map as a rigid body. Several iterative cycles of manual modification using Coot^53^ and refinement with PHENIX^54^ were necessary to fit the complete structure into the reconstructed cryo-EM density map.

In the first cycle of model refinement, only one monomer each of YjlC and Ndh was reconstructed. Then, the full protein was refined using C4 symmetry. Residues that did not match the reconstructed cryo-EM density map were removed from the model. Water molecules that were visible in the cryo-EM map were added to the model (Table S5). Density corresponding to the FAD was clearly visible and modeled. Some of the remaining unexplained densities could be interpreted as lipids. Nevertheless, other densities remained uninterpretable. Figures of structures were generated using ChimeraX^55^ and PyMol (The PyMOL Molecular Graphics System, Version 3.1 Schrödinger, LLC).

## Supplementary information

Supplementary information

Transparent Peer Review file

## Source data

Source data

## Data Availability

The atomic coordinates were deposited in the Protein Data Bank (PDB) under accession code 9SNK (for the YjlC-Ndh hetero-octamer). The cryo-EM maps were deposited in the Electron Microscopy Data Bank (EMDB) under accession codes EMD-55048 (for the YjlC-Ndh hetero-octamer), EMD-55049 (for the HMP extremity) and EMD-55050 (for the N-terminal extremity). The mass spectrometry proteomics data generated in this study have been deposited in the ProteomeXchange Consortium (http://proteomecentral.proteomexchange.org) via the PRIDE partner repository with the dataset identifier PXD069025. Source data underlying Figs. 1, 2, Supplementary Figs. S1-S4 and S6, cryo-EM data, structural maps and models files together with their corresponding validation reports are provided with this paper. Source data are provided with this paper.
