# Supplementary material for: A Bacillales-specific tubular scaffold essential for NADH dehydrogenase activity
Source: Nat Commun. 2026 Jun 18;17:7717. doi: 10.1038/s41467-026-74385-2 (PMC13433803; doi:10.1038/s41467-026-74385-2)
Supplement: Supplementary file 1 [file 41467_2026_74385_MOESM1_ESM.pdf]

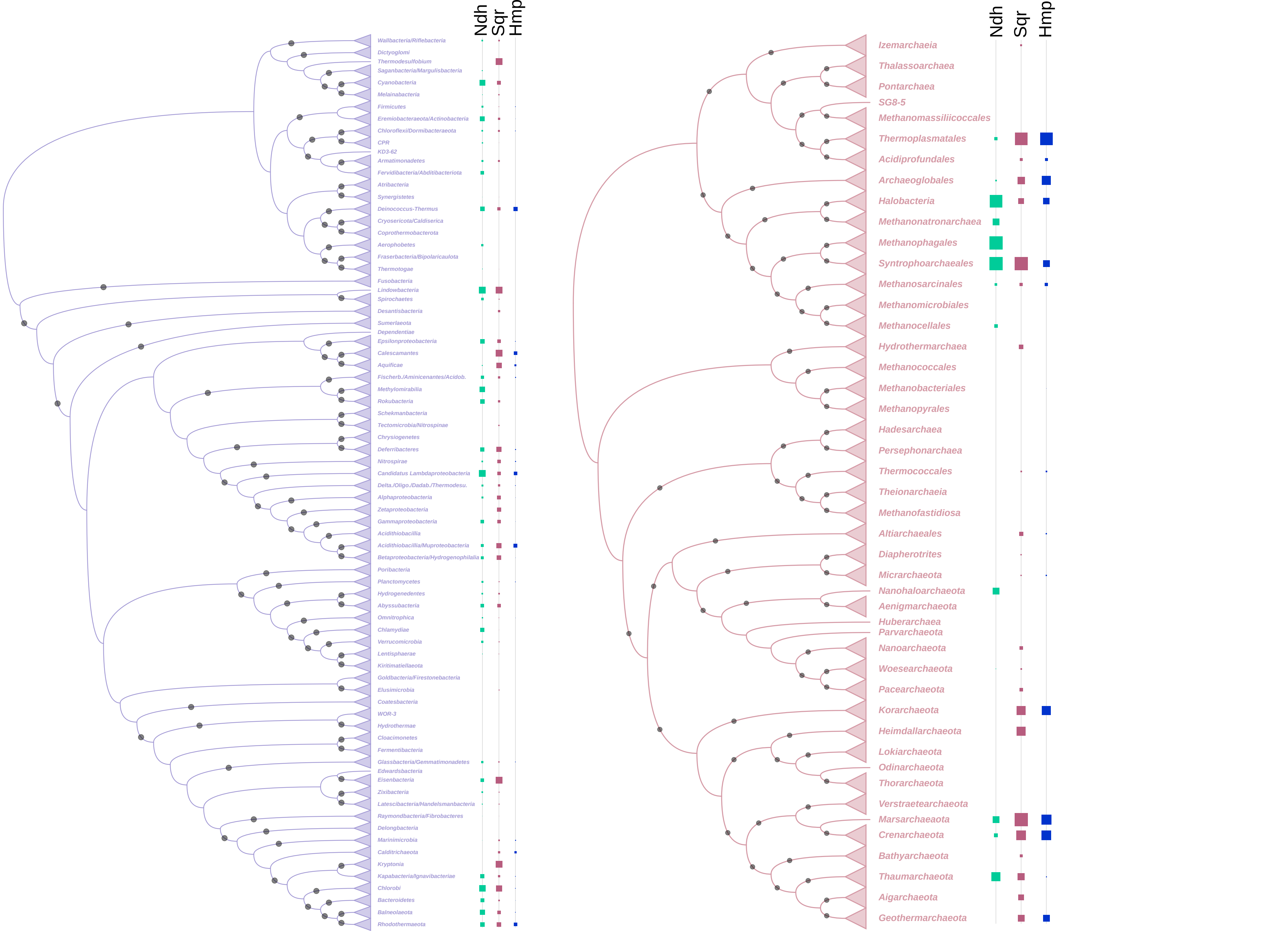

Ndh
Sqr
Hmp
Wallbacteria/Riflebacteria
Dictyoglomi
Thermodesulfobium
Saganbacteria/Margulisbacteria
Cyanobacteria
Melainabacteria
Firmicutes
Eremiobacteraeota/Actinobacteria
Chloroflexi/Dormibacteraeota
CPR
KD3-62
Armatimonadetes
Fervidibacteria/Abditibacteriota
Atribacteria
Synergistetes
Deinococcus-Thermus
Cryosericota/Caldiserica
Coprothermobacterota
Aerophobetes
Fraserbacteria/Bipolaricaulota
Thermotogae
Fusobacteria
Lindowbacteria
Spirochaetes
Desantisbacteria
Sumerlaeota
Dependentiae
Epsilonproteobacteria
Calescamantes
Aquificae
Fischerb./Aminicenantes/Acidob.
Methylomirabilia
Rokubacteria
Schekmanbacteria
Tectomicrobia/Nitrospinae
Chrysiogenetes
Deferribacteres
Nitrospirae
Candidatus Lambdaproteobacteria
Delta./Oligo./Dadab./Thermodesu.
Alphaproteobacteria
Zetaproteobacteria
Gammaproteobacteria
Acidithiobacillia
Acidithiobacillia/Muproteobacteria
Betaproteobacteria/Hydrogenophilalia
Poribacteria
Planctomycetes
Hydrogenedentes
Abyssubacteria
Omnitrophica
Chlamydiae
Verrucomicrobia
Lentisphaerae
Kiritimatiellaeota
Goldbacteria/Firestonebacteria
Elusimicrobia
Coatesbacteria
WOR-3
Hydrothermae
Cloacimonetes
Fermentibacteria
Glassbacteria/Gemmatimonadetes
Edwardsbacteria
Eisenbacteria
Zixibacteria
Latescibacteria/Handelsmanbacteria
Raymondbacteria/Fibrobacteres
Delongbacteria
Marinimicrobia
Calditrichaeota
Kryptonia
Kapabacteria/Ignavibacteriae
Chlorobi
Bacteroidetes
Balneolaeota
Rhodothermaeota
Ndh
Sqr
Hmp
Izemarchaeia
Thalassoarchaea
Pontarchaea
SG8-5
Methanomassiliicoccales
Thermoplasmatales
Acidiprofundales
Archaeoglobales
Halobacteria
Methanonatronarchaea
Methanophagales
Syntrophoarchaeales
Methanosarcinales
Methanomicrobiales
Methanocellales
Hydrothermarchaea
Methanococcales
Methanobacteriales
Methanopyrales
Hadesarchaea
Persephonarchaea
Thermococcales
Theionarchaeia
Methanofastidiosa
Altiarchaeales
Diapherotrites
Micrarchaeota
Nanohaloarchaeota
Aenigmarchaeota
Huberarchaea
Parvarchaeota
Nanoarchaeota
Woesearchaeota
Pacearchaeota
Korarchaeota
Heimdallarchaeota
Lokiarchaeota
Odinarchaeota
Thorarchaeota
Verstraetearchaeota
Marsarchaeota
Crenarchaeota
Bathyarchaeota
Thaumarchaeota
Aigarchaeota
Geothermarchaeota

Figure S1. **Taxonomic distribution of NDH-II, SQR and HMP in prokaryotes**. Left panel; bacteria. Right panel: archaea. The circles on branches represent an ultra-fast bootstrap >= 95%. The presence/absence of NDH-II (green), SQR (red) and HMP (blue) is represented by squares. For each phyla/clade, the size of the square is proportional to the ratio of the number of genomes having at least one copy on the total number of genomes. Source data are provided as a Source Data file.

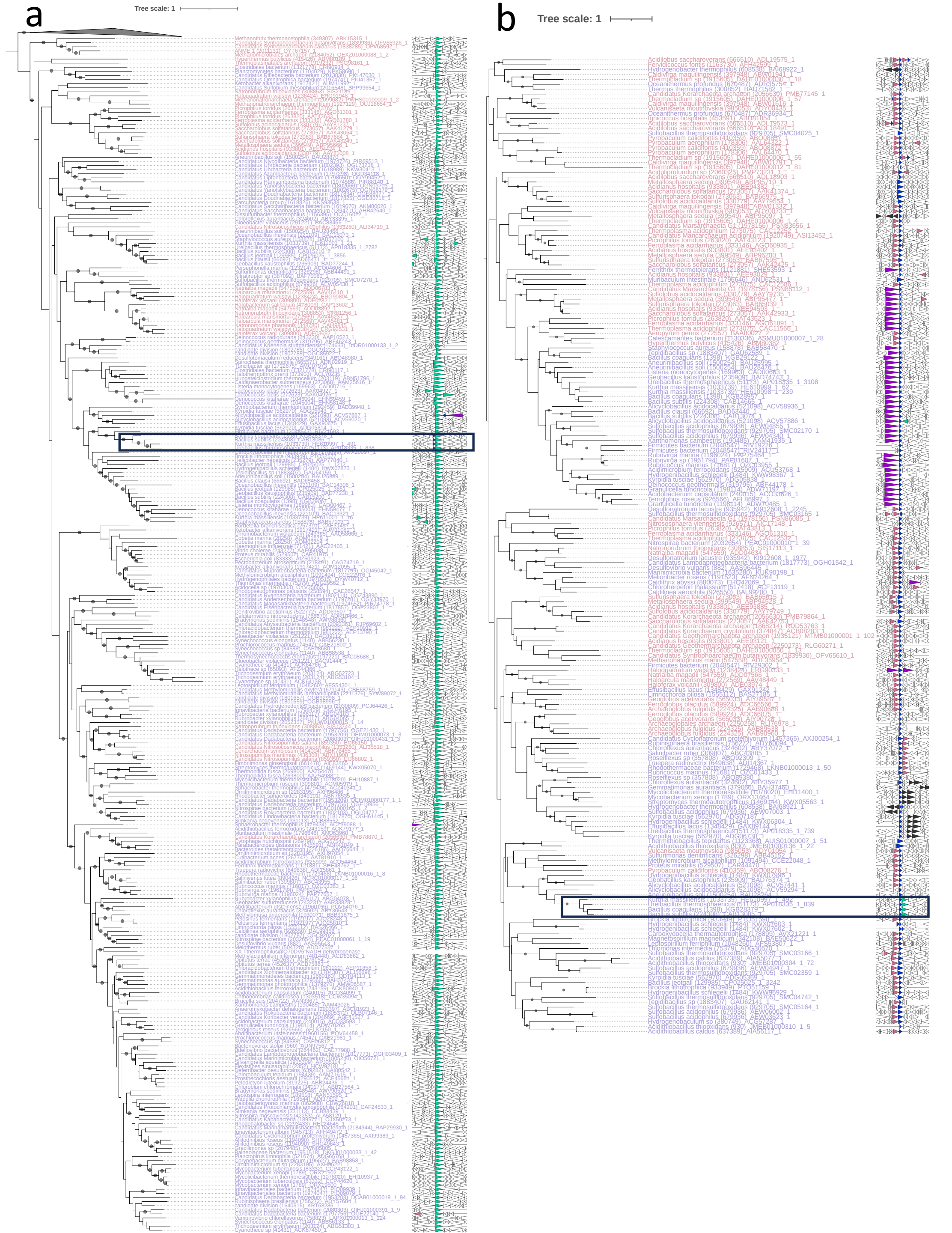

a
Tree scale: 1
b
Tree scale: 1

Figure S2. **Uncollapsed phylogenies of NDH-II and HMP.** Phylogenetic tree of NDH-II **(a)** and HMP **(b)**. For details, see Figure 1. Trees were inferred using IQ-TREE (NDH-II: 318 sequences, 359 amino-acid sites, Q.Pfam+I+R10, HMP: 200 sequences, 132 amino-acid sites, LG+F+R5). The NDH-II phylogeny was rooted using 26 SQR sequences as an outgroup. Scale bars indicate the average number of substitutions per site. Circles on branches denote ultra-fast bootstrap values >= 0.95. Bacterial sequences are shown in blue and archaeal sequences in red. Taxonomic IDs are indicated in parenthesis and protein accession numbers are provided. The genomic vicinity of NDH-II (green), HMP (blue), FDH (violet), SQR (red), and [Ni-Fe] hydrogenase (black) genes is mapped onto the phylogenies within a 5,000 bp genomic window. The specific association between NDH-II and HMP is highlighted on both phylogenies by a black rectangle. Source data are provided as a Source Data file.

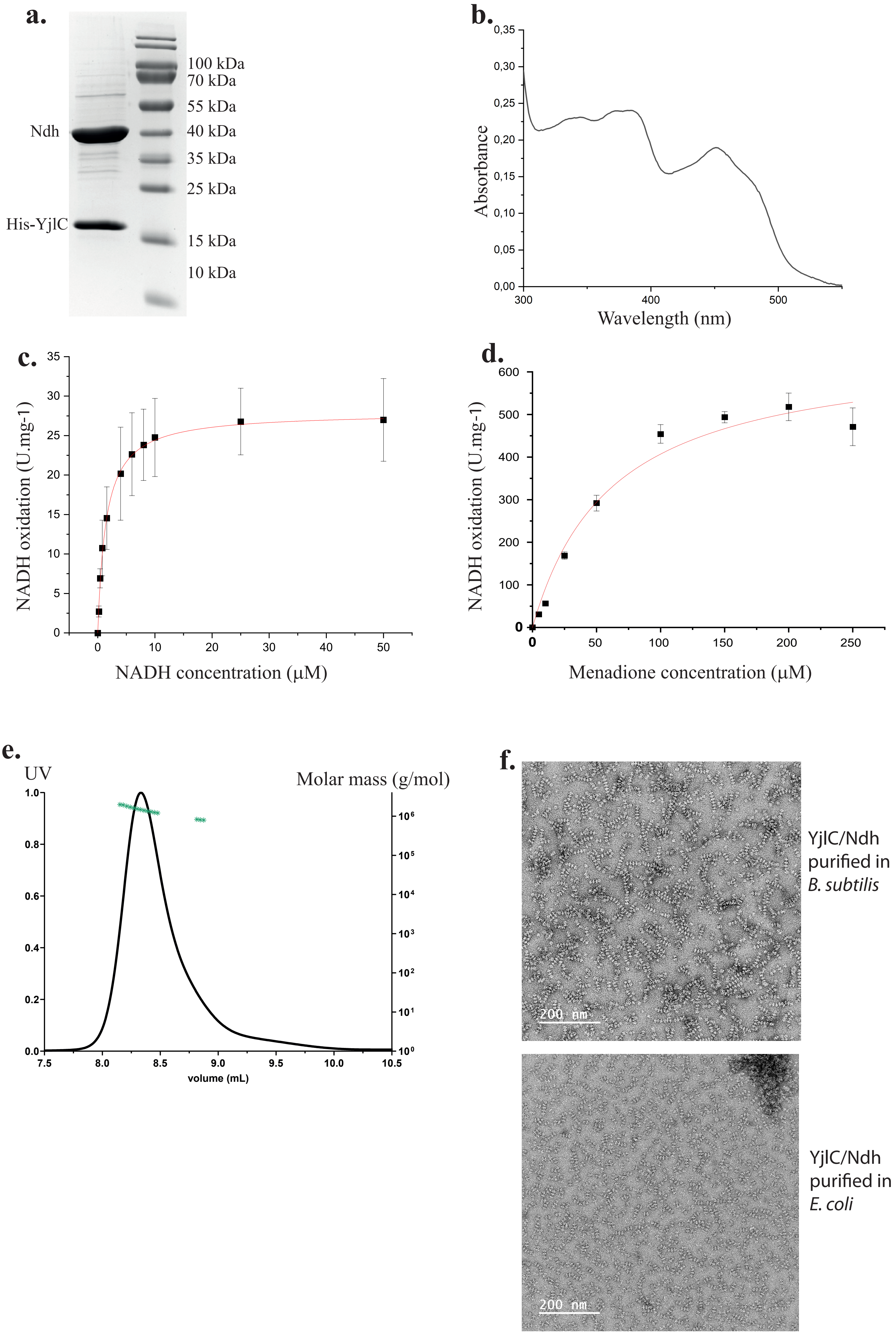

a.
Ndh
His-YjlC
100 kDa
70 kDa
55 kDa
40 kDa
35 kDa
25 kDa
15 kDa
10 kDa
b.
Absorbance
Wavelength (nm)
c.
NADH oxidation (U.mg-1)
NADH concentration (µM)
d.
NADH oxidation (U.mg-1)
Menadione concentration (µM)
e.
UV
Molar mass (g/mol)
volume (mL)
f.
YjlC/Ndh purified in *B. subtilis*
YjlC/Ndh purified in *E. coli*
200 nm
200 nm

Figure S3. Purification and biochemical characterization of the YjlC/Ndh complex. a. SDS–PAGE of the purified complex. **b**. UV–visible absorption spectrum of the oxidized complex. **c**. Michaelis–Menten kinetics of NADH oxidation using DCPIP as electron acceptor. Data from n = 3 biological replicates are presented as mean values +/− SD. **d**. Michaelis–Menten kinetics of NADH oxidation using menadione as electron acceptor. Data from n = 3 biological replicates are presented as mean values +/− SD. **e**. SEC–MALS analysis of the purified complex. Left y-axis, normalized UV chromatograms; right y-axis, molar mass with measured molar mass in green symbols. **f**. Negative-stain electron micrographs of YjlC/Ndh purified from *B. subtilis* or *E. coli*, respectively. Source data are provided as a Source Data file.

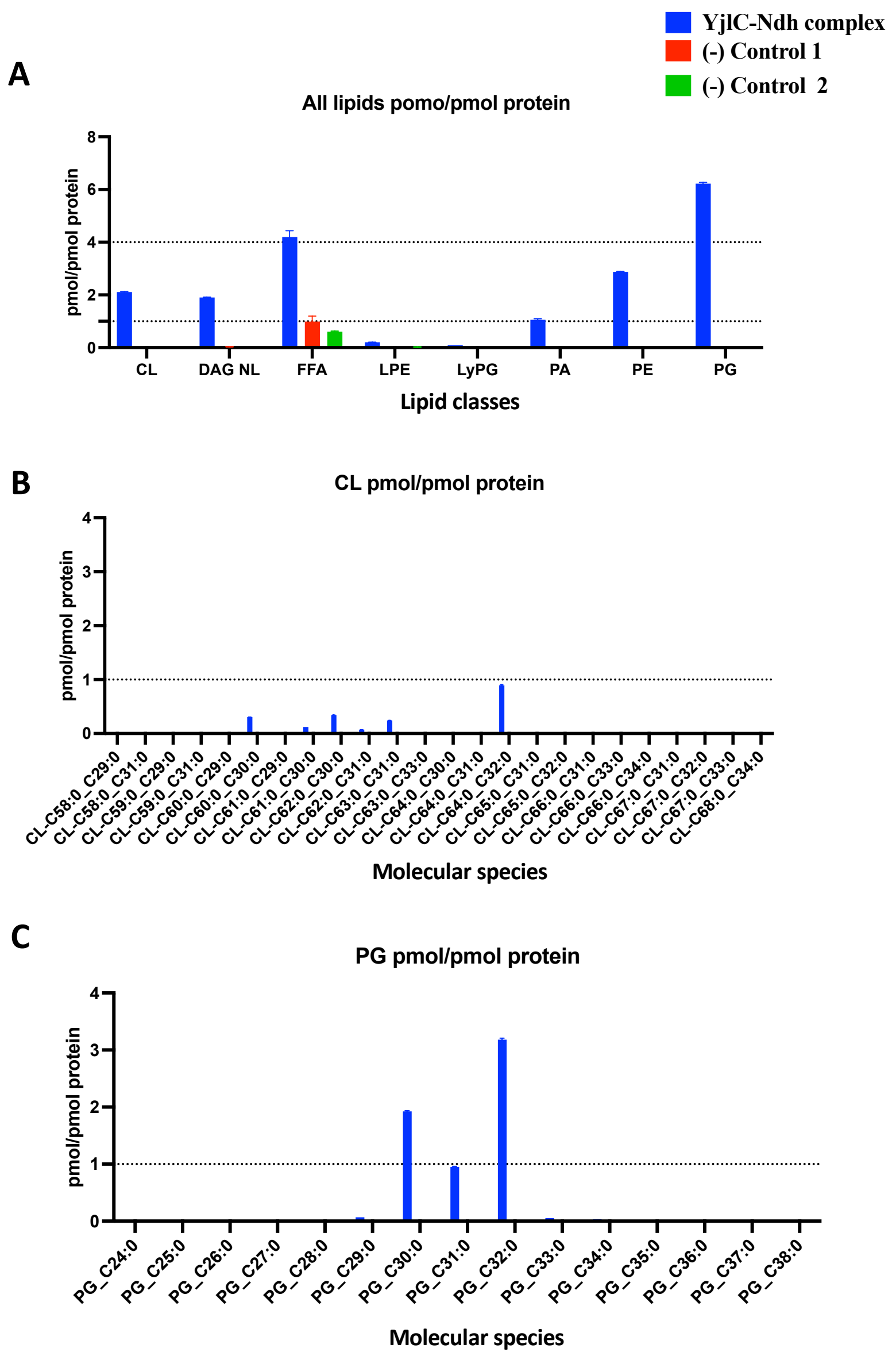

YjlC-Ndh complex
(-) Control 1
(-) Control 2
A
All lipids pomo/pmol protein
pmol/pmol protein
0
2
4
6
8
CL
DAG NL
FFA
LPE
LyPG
PA
PE
PG
Lipid classes
B
CL pmol/pmol protein
pmol/pmol protein
0
1
2
3
4
CL-C58:0_C29:0
CL-C58:0_C31:0
CL-C59:0_C29:0
CL-C59:0_C31:0
CL-C60:0_C29:0
CL-C60:0_C30:0
CL-C61:0_C29:0
CL-C61:0_C30:0
CL-C62:0_C30:0
CL-C62:0_C31:0
CL-C63:0_C31:0
CL-C63:0_C33:0
CL-C64:0_C30:0
CL-C64:0_C31:0
CL-C64:0_C32:0
CL-C65:0_C31:0
CL-C65:0_C32:0
CL-C66:0_C31:0
CL-C66:0_C33:0
CL-C66:0_C34:0
CL-C67:0_C31:0
CL-C67:0_C32:0
CL-C67:0_C33:0
CL-C68:0_C34:0
Molecular species
C
PG pmol/pmol protein
pmol/pmol protein
0
1
2
3
4
PG_C24:0
PG_C25:0
PG_C26:0
PG_C27:0
PG_C28:0
PG_C29:0
PG_C30:0
PG_C31:0
PG_C32:0
PG_C33:0
PG_C34:0
PG_C35:0
PG_C36:0
PG_C37:0
PG_C38:0
Molecular species

Figure S4. Lipidomic analysis reveals enrichment of cardiolipin and phosphatidylglycerol in the purified YjlC-Ndh complex. a. Mass spectrometry-based lipidomic analyses of the purified YjlC-Ndh complex (blue bar) and two negative controls unable to specifically bind lipids (catalase 1, red bars, and catalase 2, green bars) (see Methods). MSMS analyses reveal the molecular profiles of the CL (**b**), and PG (**c**) species associated with the complex. Source data are provided as a Source Data file.

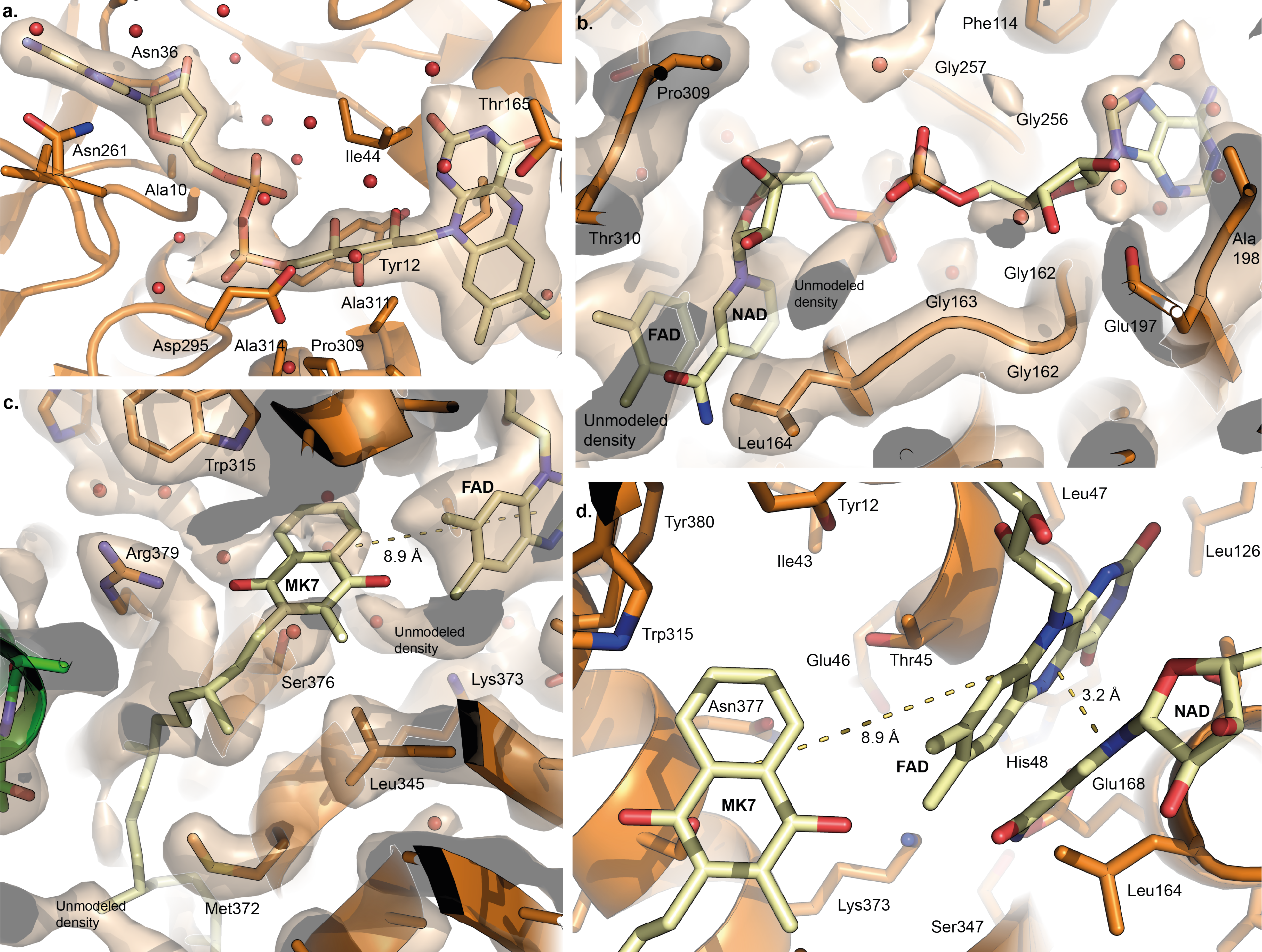
a.
Asn36
Thr165
Ile44
Asn261
Ala10
Tyr12
Ala311
Asp295
Ala314
Pro309
b.
Phe114
Gly257
Pro309
Gly256
Thr310
Ala
198
Gly162
Unmodeled
density
Gly163
NAD
FAD
Glu197
Gly162
Unmodeled
density
Leu164
c.
Trp315
FAD
Arg379
8.9 Å
MK7
Unmodeled
density
Ser376
Lys373
Leu345
Unmodeled
density
Met372
d.
Tyr380
Tyr12
Leu47
Ile43
Leu126
Trp315
Glu46
Thr45
3.2 Å
Asn377
NAD
8.9 Å
FAD
His48
Glu168
MK7
Lys373
Leu164
Ser347

Figure S5. Cryo-EM density of FAD and proposed docking sites for NAD^+ and MK7 in Ndh. a. FAD inside the Ndh active site. Coulomb potential density is displayed only around the FAD molecule as a beige semi-transparent surface and contoured at 6σ. **b.** Comparison of the proposed NAD^+ binding site and the cryo-EM reconstruction of YjlC-Ndh. The Coulomb potential density, contoured at 4σ, does not indicate the presence of NAD^+ in its expected binding site. Ndh protein and FAD atoms correspond to the model built inside the YjlC-Ndh cryo-EM reconstruction. NAD^+ corresponds to an Alphafold3 model of Ndh with NAD^+ calculated and aligned to the Ndh Cryo-EM model. **c.** Comparison of the proposed menaquinone binding site and the cryo-EM reconstruction of YjlC-Ndh. The Coulomb potential density, contoured at 4σ, does not indicate the presence of MK7 in the expected location for electron transfer with the FAD molecule in the active site. Ndh-YjlC proteins and FAD atoms correspond to the model built inside the YjlC-Ndh cryo-EM reconstruction. MK7 corresponds to an Alphafold3 model of Ndh with MK7 calculated and aligned to the Ndh Cryo-EM model. **d.** Model of the Ndh active site. Ndh protein and FAD atoms correspond to the model built inside the YjlC-Ndh cryo-EM reconstruction. NAD^+ and MK7 correspond to an Alphafold3 model of Ndh with NAD^+ or MK7 calculated and aligned to the Ndh Cryo-EM model. Ndh protein main chain and side chains are represented as cartoon and sticks respectively with carbon colored in orange. MK7, NAD^+ and FAD are represented by sticks, with the carbon, oxygen, nitrogen, and phosphorus atoms colored light yellow, red, blue, and orange, respectively. The Ndh and YjlC main chains are displayed as an orange and green, semi- transparent cartoon, respectively. Side chain atoms are displayed as sticks, and water molecules are displayed as spheres.

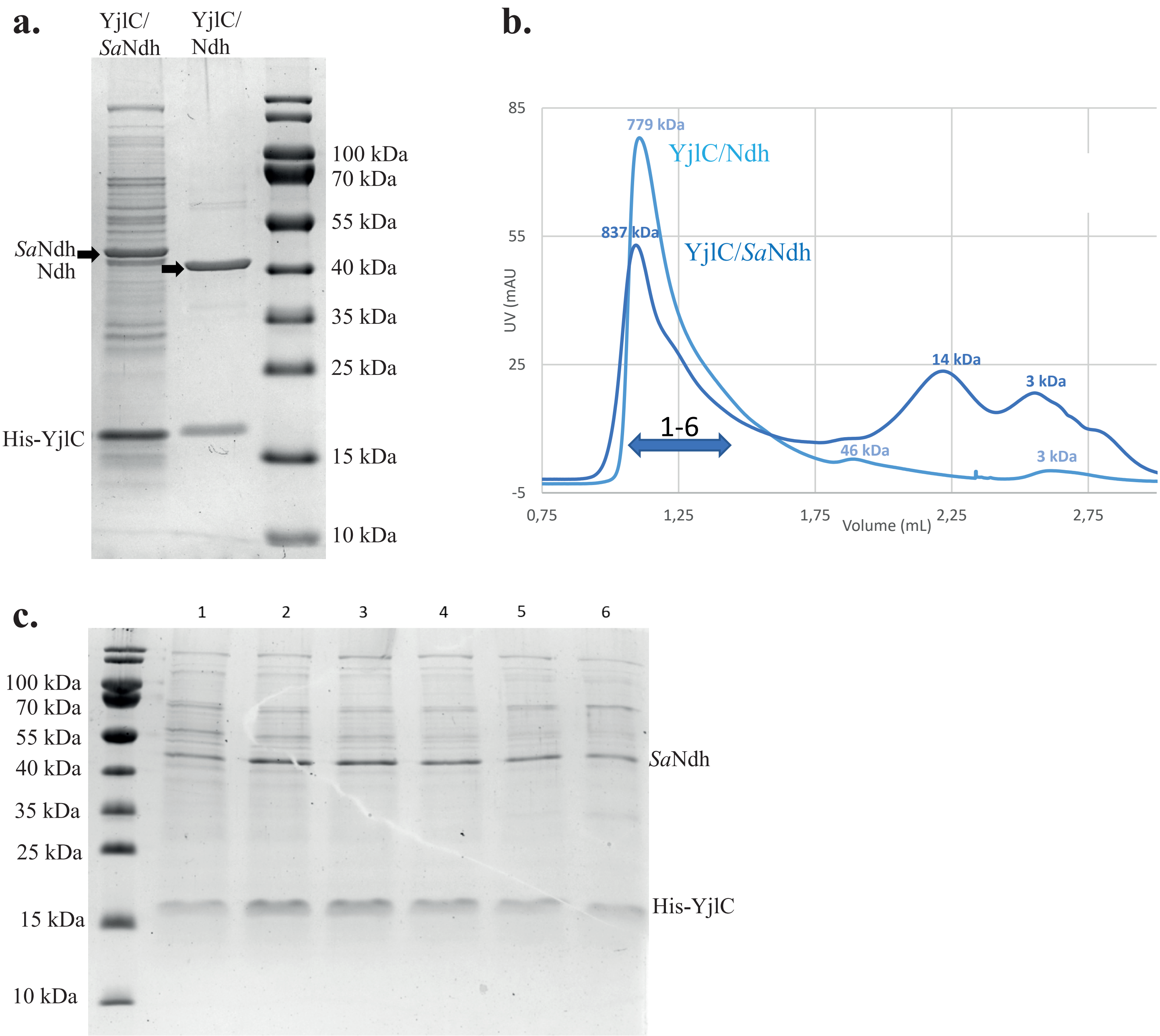

d.

Accession number	Protein name	Coverage [%]	Peptide Spectral Match	Unique Peptides	MW [kDa]
A0A199WG74	Ndh [B. subtilis]	44	105	18	41.9
NA	6his-YjlC [B.subtilis]	55	26	7	16.6

e.

Accession number	Protein Name	Coverage [%]	Peptide Spectral Match	Unique Peptides	MW [kDa]
A0A0H3KFB6	*Sa* NDH [S. aureus]	29	97	13	44.1
P04949	Flagellin [E. coli]	23	34	6	51.3
P09546	Bifunctional protein PutA [E. coli]	14	29	13	143.7
P0AC41	Succinate dehydrogenase FAD subunit [E. coli]	11	22	5	64.4
P0ABB4	ATP synthase subunit beta [E. coli]	27	13	8	50.3
P0ABB0	ATP synthase subunit alpha [E. coli]	18	11	8	55.2
P32176	FdoG [E. coli]	6	9	4	112.5
NA	6his-YjlC [B. subtilis]	48	9	6	16.6
P0A7G6	Protein RecA [E. coli]	12	7	3	38
P0A6J5	D-amino acid dehydrogenase [E. coli]	16	6	4	47.6
P0ABJ9	bd-I ubiquinol oxidase subunit 1 [E. coli]	7	5	3	58.2
P13035	Aerobic G3P dehydrogenase [E. coli]	13	5	4	56.7

Figure S6. **Cross-species interaction between YjlC and *S. aureus* Ndh shows reduced stability. a**. SDS-PAGE of His-tagged YjlC co-purified with *S. aureus* Ndh (*Sa*Ndh, lane 1) or *B. subtilis* Ndh (Ndh, lane 2). **b**. SEC chromatograms of YjlC/Ndh and YjlC/*Sa*Ndh. **c**. SDS-PAGE of peak fractions (1–6) of YjlC/*Sa*Ndh. **d**. Mass spectrometry analysis of SEC fraction 2 of YjlC/Ndh. **e**. Mass spectrometry analysis of SEC fraction 2 of YjlC/*Sa*Ndh. Source data are provided as a Source Data file.

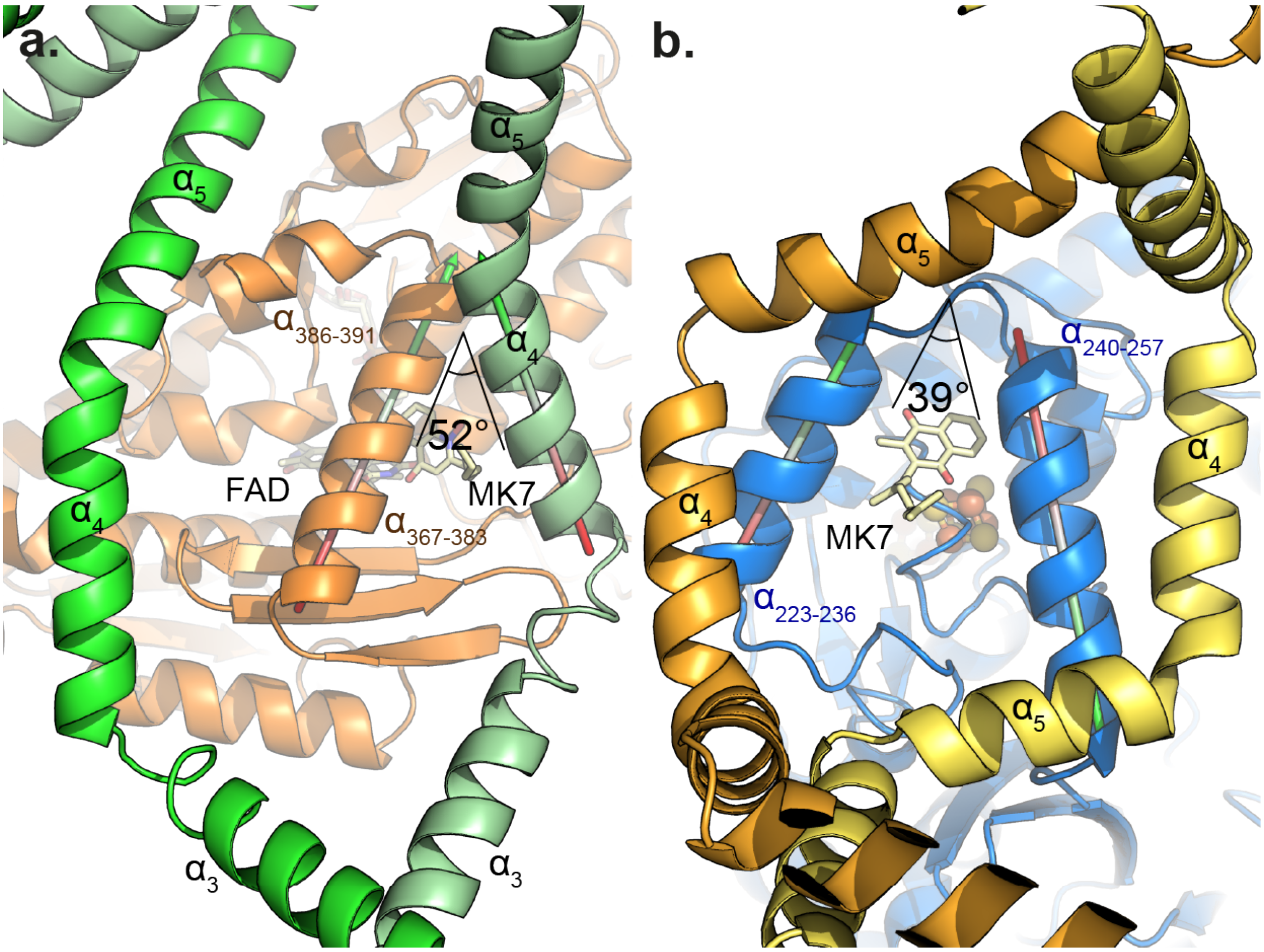

a.
α5
α5
α386-391
α4
52°
FAD
MK7
α4
α367-383
α3
α3
b.
α5
α240-257
39°
α4
α4
MK7
α223-236
α5

Figure S7. Comparison between the V-shape motif in YjlC-Ndh and ForC. a. Ndh is represented in cartoon colored in orange. α-helices of Ndh are colored in green and numbered. MK7 is positioned according to Alphafold3 prediction. FAD and MK7 are represented by sticks, with the carbon, oxygen, nitrogen, and phosphorus atoms colored light yellow, red, blue, and orange, respectively. **b.** ForC is represented in cartoon colored in dark blue. α-helices of ForE are colored in yellow and brown and numbered. Clusters are represented in balls and sticks and MK7 by sticks. Carbon, oxygen, nitrogen, sulfur and iron atoms are colored in light yellow, red, blue, yellow and brown, respectively.

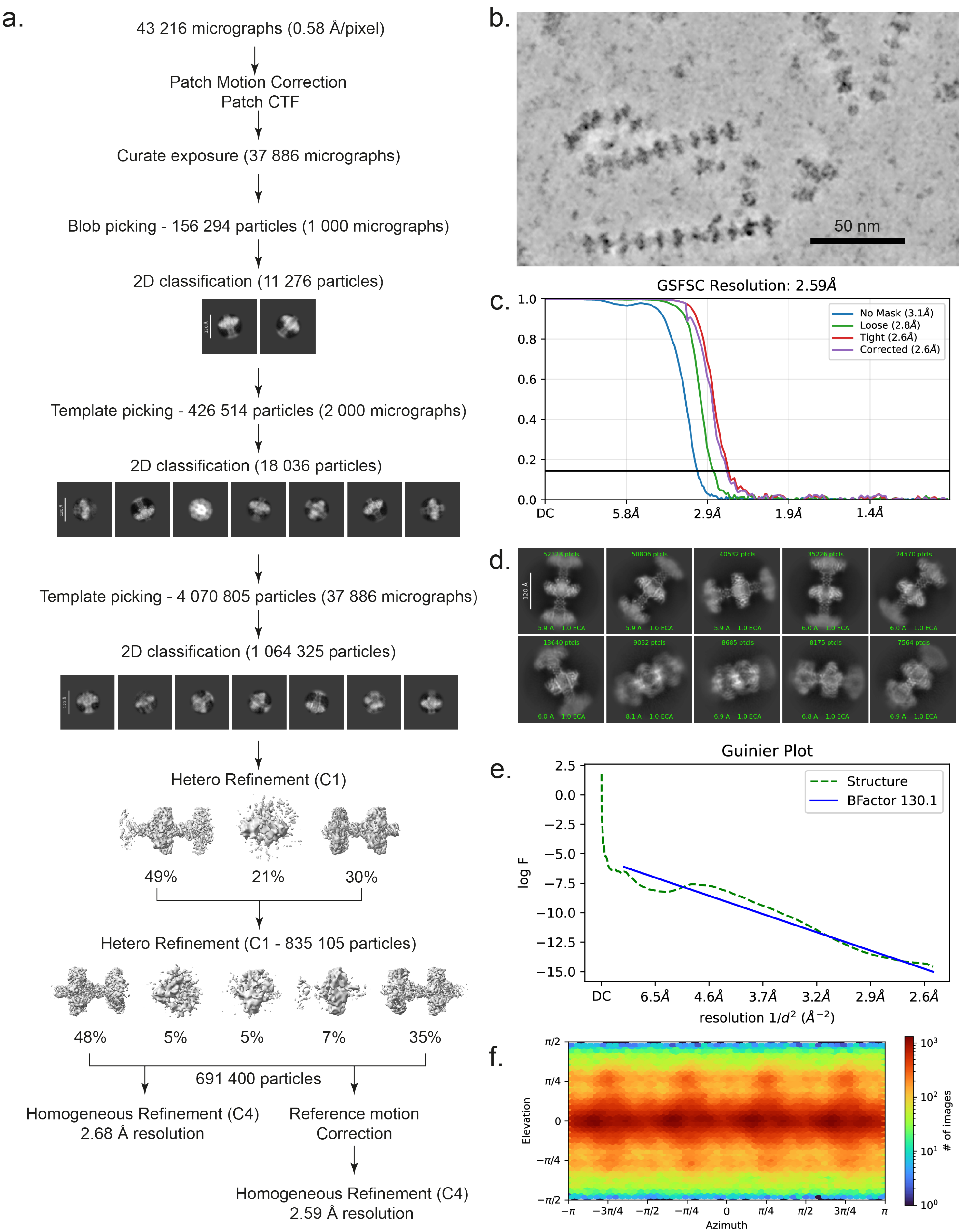
a.
43 216 micrographs (0.58 Å/pixel)
Patch Motion Correction
Patch CTF
Curate exposure (37 886 micrographs)
Blob picking - 156 294 particles (1 000 micrographs)
2D classification (11 276 particles)
Template picking - 426 514 particles (2 000 micrographs)
2D classification (18 036 particles)
Template picking - 4 070 805 particles (37 886 micrographs)
2D classification (1 064 325 particles)
Hetero Refinement (C1)
49%
21%
30%
Hetero Refinement (C1 - 835 105 particles)
48%
5%
5%
7%
35%
691 400 particles
Homogeneous Refinement (C4)
2.68 Å resolution
Reference motion
Correction
Homogeneous Refinement (C4)
2.59 Å resolution
b.
50 nm
c.
GSFSC Resolution: 2.59Å
No Mask (3.1Å)
Loose (2.8Å)
Tight (2.6Å)
Corrected (2.6Å)
DC
5.8Å
2.9Å
1.9Å
1.4Å
d.
52328 ptcls
50806 ptcls
40532 ptcls
35226 ptcls
24570 ptcls
13640 ptcls
9032 ptcls
8685 ptcls
8175 ptcls
7564 ptcls
e.
Guinier Plot
Structure
BFactor 130.1
log F
resolution $1/d^2$ ($Å^{-2}$)
DC
6.5Å
4.6Å
3.7Å
3.2Å
2.9Å
2.6Å
f.
Elevation
Azimuth
# of images

Figure S8. Cryo-EM reconstruction procedure of the YjlC-Ndh complex. a. YjlC-Ndh cryo-EM data processing workflow. **b.** Motion-corrected and denoised micrograph of YjlC-Ndh complex. Scale bar indicates 50 nm. **c.** Fourier-shell-correlation plots for independently refined half-maps. Horizontal black line indicates Fourier-shell-correlation values of 0.143. **d.** Examples of 2D classes of the final selection of particles. Number of the particles and estimated resolution in each 2D class are indicated. Scale bar indicates 120 Å. **e.** Guinier plot of the final homogeneous refinement indicating a sharpening B-factor of 130.1 $Å^2$. **f.** Angular distribution of the particles used for the final reconstruction.

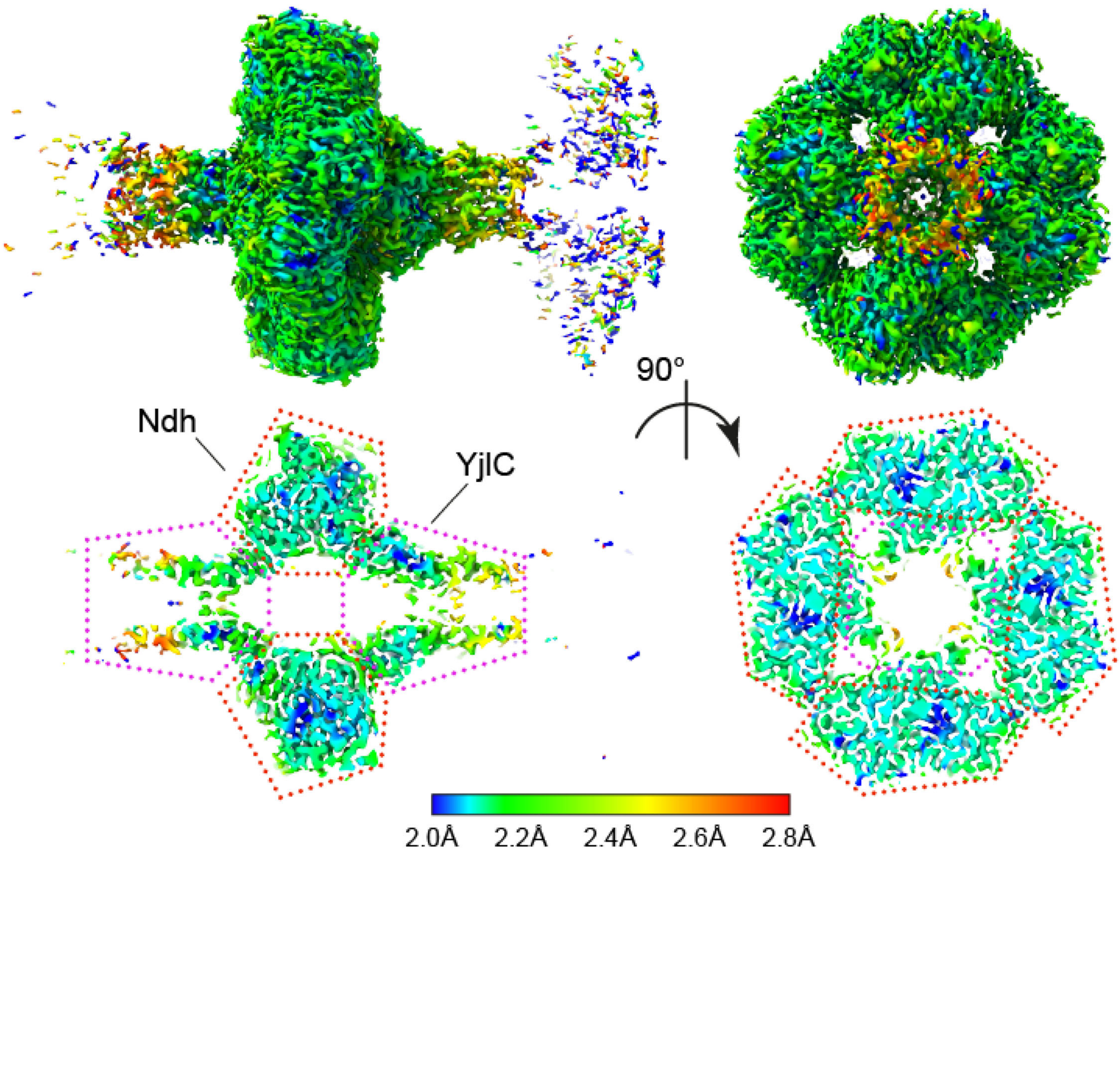
90°
Ndh
YjlC
2.0Å
2.2Å
2.4Å
2.6Å
2.8Å

Figure S9. YjlC-Ndh cryoEM reconstruction local resolution. The full reconstruction (Top) and a cross section (bottom) are colored according to local resolution, from dark blue (2.0 Å) to red (2.8 Å), and contoured at 3 σ. In the cross sections, the YjlC and Ndh tetramers are outlined with purple and red dashed lines, respectively.

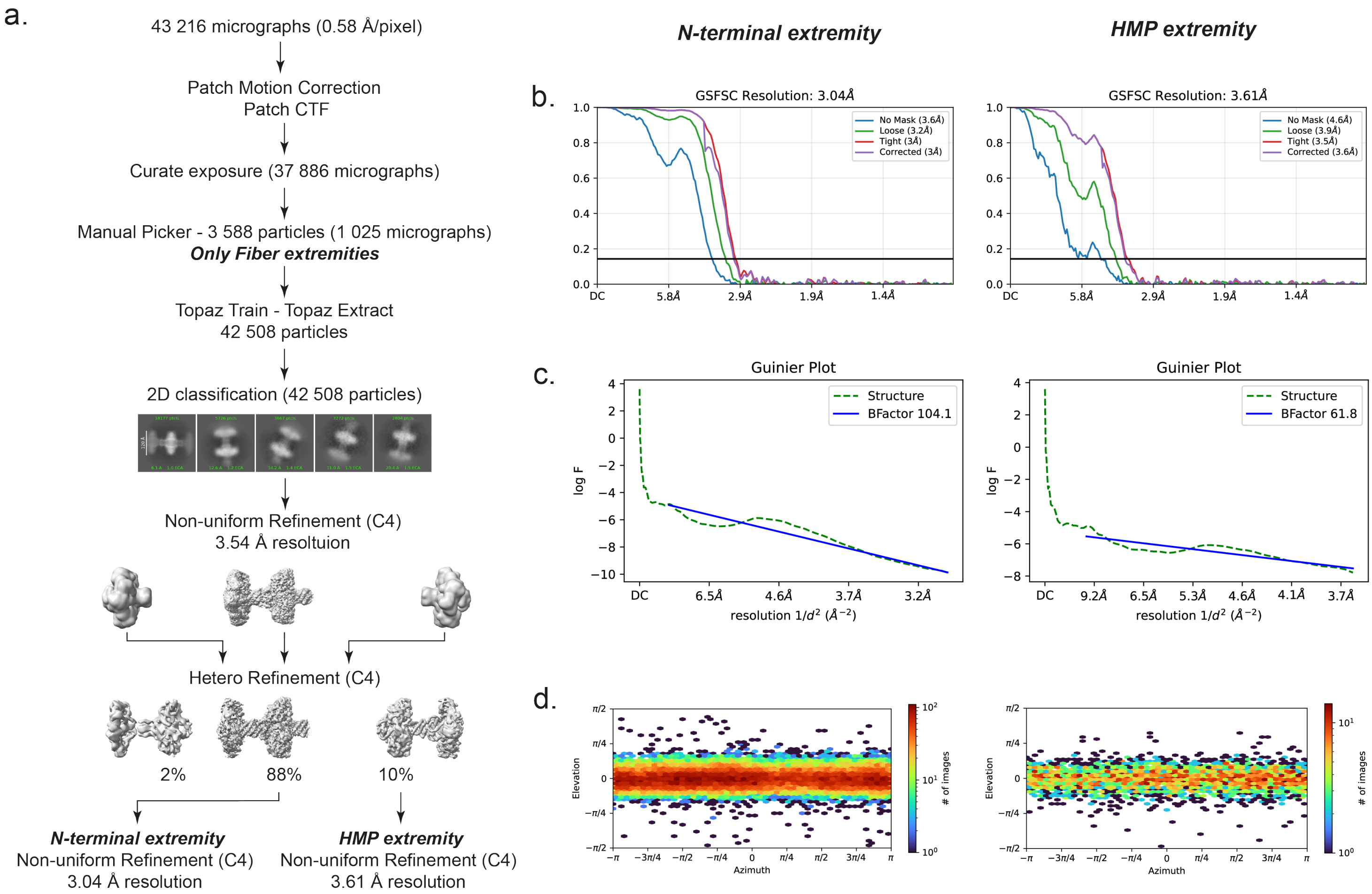

a.
43 216 micrographs (0.58 Å/pixel)
Patch Motion Correction
Patch CTF
Curate exposure (37 886 micrographs)
Manual Picker - 3 588 particles (1 025 micrographs)
Only Fiber extremities
Topaz Train - Topaz Extract
42 508 particles
2D classification (42 508 particles)
Non-uniform Refinement (C4)
3.54 Å resoltuion
Hetero Refinement (C4)
2%
88%
10%
N-terminal extremity
Non-uniform Refinement (C4)
3.04 Å resolution
HMP extremity
Non-uniform Refinement (C4)
3.61 Å resolution
N-terminal extremity
HMP extremity
b.
GSFSC Resolution: 3.04Å
No Mask (3.6Å)
Loose (3.2Å)
Tight (3Å)
Corrected (3Å)
GSFSC Resolution: 3.61Å
No Mask (4.6Å)
Loose (3.9Å)
Tight (3.5Å)
Corrected (3.6Å)
c.
Guinier Plot
Structure
BFactor 104.1
log F
resolution 1/d² (Å⁻²)
Guinier Plot
Structure
BFactor 61.8
log F
resolution 1/d² (Å⁻²)
d.
Elevation
Azimuth
# of images
Elevation
Azimuth
# of images

Figure S10. Cryo-EM reconstruction procedure of the two fiber extremities of YlcC-Ndh complex. a. YjlC-Ndh cryo-EM data processing workflow used to calculate the two extremities of the fibers. **b.** Fourier-shell-correlation plots for independently refined half-maps for N-terminal (left) and HMP extremities (right). Horizontal black line indicates Fourier-shell-correlation values of 0.143. **c.** Guinier plot of the final homogeneous refinement indicating a sharpening B-factor of 130.1 Å^2. **d**. Angular distribution of the particles used for the final reconstruction.

Table S1. Lists of genomes used for phylogenetic study.

Table S2. Taxonomic distribution of NDH-II, SQR and HMP in prokaryotes. The genomic clustering is indicated by a grouping in the same cell. Accession numbers are between parentheses.

Table S3. HPLC-ECD quantification of UQ and MK in lipid extracts of purified *Bs*YjlC/Ndh in *Bacillus subtilis* (n=3) and in *Escherichia coli* (n=2)

Enzyme purified in *Bs*	MK-7 (pmol/pmol YjlC/Ndh)	MK-8 (pmol/pmol YjlC/Ndh)
*Bs*YjlC/Ndh	0.54 ± 0.11	0.016 ± 0.004
Enzyme purified in *Ec*	UQ-8 (pmol/pmol YjlC/Ndh)	MK-8 (pmol/pmol YjlC/Ndh)
*Bs*YjlC/Ndh	0.35 ± 0.02	0.105 ± 0.007

Table S4. Strains, plasmids and primers used in this work.

**Strains**		
**Name**	**Description**	**Reference**
**WT**	*B. subtilis* 168 (*trpC2*) (4161)	BGSC
**Δ*yjlC* Δ*ndh***	*B. subtilis* 168 Δ*yjlC* Δ*ndh::kan* (4556).The Δ*yjlC* Δ*ndh* mutant was constructed by PCR amplification of a kanamycin resistance cassette flanked by 1 kb sequences upstream of *yjlC* and downstream of *ndh* using primers 2209, 2210, 2211, 2213, 2205 and 2214 followed by transformation into WT.	This work
**Δ*yjlC* Δ*ndh* p*ndh***	*B. subtilis* 168 Δ*yjlC* Δ*ndh::kan amyE::*p*ndh* (4549). *B. subtilis* 168 Δ*yjlC* Δ*ndh* was transformed with p*ndh.*	This work
**Δ*yjlC* p*yjlC***	*B. subtilis* 168 Δ*yjlC::kan amyE::*p*yjlC* (4553). *B. subtilis* 168 Δ*yjlC* was transformed with p*yjlC.*	This work
**Δ*yjlC***	*B. subtilis* 168 Δ*yjlC::kan* (BKK12280) (4337)	BGSC
**4355**	*B. subtilis* 168 *spec*-Pxyl*::*6xCAT-*yjlC-ndh*. For 6his-YjlC/Ndh overproduction, WT was transformed with a spectinomycin resistance cassette carrying an inducible promoter and a 6×CAT tag, flanked by 1 kb sequences upstream and downstream of *yjlC* using primers 2049, 2050, 2051, 1194, 1195 and 2052. The resulting strain expresses a 6×CAT–*yjlC* fusion and *ndh* at the native *yjlC* locus under the control of the xylose-inducible promoter.	This work
***BW25113* Δ*ndh::*kan**	*E. coli K12 Δ(araD-araB)567 ΔlacZ4787(::rrnB-3) λ– rph-1 Δ(rhaD-rhaB)568 hsdR514 Δndh::kan* (4570)	Keio collection[1]
E. coli Shuffle® T7	*E. coli K12 F´ lacIq, lacZ::T7 gene1, ΔtrxB, Δgor, λatt::pNEB3-r1-cDsbC (SpecR, lacIq), ΔmalF*	New England Biolabs
E. coli Shuffle® T7* Δ*ndh	*E. coli Shuffle® T7* Δ*ndh::kan* (4573). P1 transduction of Δ*ndh::kan* from BW25113 Δ*ndh::kan.* The *kan* cassette was removed with pCP20.	This work
**Plasmids**		
**Name**	**Description**	**Reference**
p*ndh*	6xCAT-*ndh* was amplified by PCR with 2172 and 2173 and the plasmid pSPH1 digested by *Avr*II and *Eco*RI (4516).	This work
p*yjlC*	6xCAT-*yjlC* was amplified by PCR with 2206 and 2207 and the plasmid pSPH1 digested by *Avr*II and *Eco*RI (4545).	This work
pET28a-*yjlC*-6×CAT–*ndh*	*yjlC* was amplified by PCR with 2245 and 2246 and the plasmid pET28a-6×CAT–*ndh* digested by *Xba*I (4558).	This work
pET28a-6×CAT–*ndh*	*ndh* was amplified by PCR with 2235 and 2234 and the plasmid pET28a digested by *Hind*III and *Bam*HI (4571).	This work
pLB6	*Sandh* was amplified with 2522 and 2515 from genomic DNA of *S. aureus* HG003, 6xCAT-*yjlC* with 2511 and 2521 from strain 4355 and the plasmid pET14a digested by *Xba*I. The three fragments were assembled by using the pro ligation free cloning master mix (abm) (4588).	This work
pCP20	To remove kan cassette in *BW25113* Δ*ndh::*kan	[2]
**Primers**		
**Name**	**Sequence**	**To amplify**

**2209-UP1 5'**	gcaggcgagaaaggagag	*kan* cassette
**2210-UP4 3'**	cgaggctcctgtcactgc	*kan* cassette
**2211-5pL-yjlC 5'**	tcatgcagatcaatggaatca	1 kb up *yjlC*
**2213-5pR-yjlC 3'**	ctctcctttctcgcctgccatatccaattctcctttac	1 kb up *yjlC*
**2205-UxaC 3'**	aaaccgcaaagaaactgaag	1 kb down *ndh*
**2214-3pL-ndh 5'**	gcagtgacaggagcctcgtaatccttttaatgaatctg	1 kb down *ndh*
**2206-avrII-yjlC 5'**	ctagaaaggagattcctaggatgcaccaccaccaccaccacccagaaacaatcgatcaa	6xCAT-*yjlC*
**2207-EcoRI-yjlC 3'**	ggccgctctagaactagaattcttatttttggttttcgcgttcg	6xCAT-*yjlC*
**2049-yjlB 5'**	gcagccgcaaatgtaatcggtgcg	1 kb up *yjlC*
**2050-pspec 3'**	taataattattctttattatacagatcgatcctctagagatccaattctcctttactat	1 kb down *ndh*
**2051-pspec 5'**	aaaaaaatcagcaaactgcttatagtaaaggagaattggatctctagaggatcgatctg	*spec*-xylose promoter-6xCAT-*yjlC*
**1194-yjlC 3'**	gacaatatgttttgacatcgtatatcctccgtcc	*spec*-xylose promoter-6xCAT-*yjlC*
**1195-ndh 5'**	cggaggatatacgatgtcaaaacatattgtcattc	*ndh*
**2052-ndh 3'**	ttagtaagccaggctgaaaagtcc	*ndh*
**2172-pXyl-NdhII 5'**	ctagaaaggagattcctaggatgcaccaccaccaccaccactcaaaacatattgtcatt	6xCAT-*ndh*
**2173-pSPH-NdhII 3'**	cggccgctctagaactagaattcttagtaagccaggctgaaaagtcc	6xCAT-*ndh*
**2245-XbaI yjlC 5'**	ctagtctagaaaggagatataccatgccagaaacaatcgatcaaac	*yjlC*
**2246-XbaI yjlC 3'**	ctagtctagattatttttggttttcgcgttcgttcatgacttcaagg	*yjlC*
**2235-BamHI Ndh 5'**	gcaggaggatccatgtcaaaacatattgtc	*ndh*
**2234-HindIII NDH 3'**	atatgaaagcttagtaagccaggctgaa	*ndh*
**2522-LB12- 5'**	aggatatacgatggctcaagatcgtaaaaaag	*Sandh*
**2515-LB5-3'**	ttaaagttaaacaaaattatttctagaatttacctttttgaatgc	*Sandh*
**2511-LB1-5'**	gagaccacaacggtttccctagaaaggagattcctaggatg	6xCAT-*yjlC*
**2521-LB11-3'**	cttgagccatcgtatatcctccgtcctttatatc	6xCAT-*yjlC*

1. Baba, T., Ara, T., Hasegawa, M., Takai, Y., Okumura, Y., Baba, M., Datsenko, K.A., Tomita, M., Wanner, B.L., and Mori, H. (2006). Construction of Escherichia coli K-12 in-frame, single-gene knockout mutants: the Keio collection. Mol. Syst. Biol. *2*, 2006.0008. https://doi.org/10.1038/msb4100050.

2. Cherepanov, P.P., and Wackernagel, W. (1995). Gene disruption in Escherichia coli: TcR and KmR cassettes with the option of Flp-catalyzed excision of the antibiotic-resistance determinant. Gene *158*, 9–14. https://doi.org/10.1016/0378-1119(95)00193-a.

Table S5. Cryo-EM data collection, refinement and validation statistics

	YjlC-Ndh<br>(EMDB-55048)<br>(PDB 9SNK)	YjlC-Ndh fiber's HMP extremity<br>(EMDB-55049)	YjlC-Ndh fiber's N-ter extremity<br>(EMDB-55050)
**Data collection and processing**			
Magnification	215 000		
Voltage (kV)	300		
Electron exposure (e^-/Å^2)	40		
Defocus range (μm)	-0.5 to -2.9		
Pixel size (Å)	0.58		
Symmetry imposed	C4	C4	C4
Initial particle images (no.)	4 070 805	42 508	42 508
Final particle images (no.)	691 400	4 133	37 373
Map resolution (Å)<br>FSC threshold	2.59<br>0.143	3.61<br>0.143	3.04<br>0.143
Map resolution range (Å)	1.58 – 9.30	1.21 – 57.81	1.21 – 48.52
**Refinement**			
Initial model used (PDB code)	Alphafold model	–	–
Model resolution (Å)<br>FSC threshold	2.9<br>0.143	–	–
Map sharpening *B* factor (Å^2)	-130.1	-61.8	-104.1
Model composition<br>Non-hydrogen atoms<br>Protein residues<br>Ligands<br>Water	 17 888<br>2 052<br>104<br>988	–	–
B factors (Å^2)<br>Protein<br>Ligand<br>Water	 42.76<br>54.33<br>41.24	–	–
R.m.s. deviations<br>Bond lengths (Å)<br>Bond angles (°)	 0.004<br>0.920	–	–
Validation<br>MolProbity score<br>Clashscore<br>Rotamers Outliers (%)	 1.34<br>6.24<br>0.70	–	–
Ramachandran plot<br>Favored (%)<br>Allowed (%)<br>Disallowed (%)<br>Model vs. Data<br>CC (mask)<br>CC (main chain)<br>CC (side chain)<br>Mean CC for ligands	 98.43<br>1.57<br>0.00<br><br>0.88<br>0.88<br>0.81<br>0.66	–	–

Table S6. List of internal standards

Cpd Group	Cpd Name	Prec Ion	Prod Ion	Frag (V)	CE (V)	Cell Acc (V)	Ret Time (min)	Ret Window	Polarity
CL	CL-C58:0_C29:0	1286.91	509.5	166	42	4	10.55	2.11	Positive
CL	CL-C58:0_C31:0	1286.91	537.5	166	42	4	8.03	1.61	Positive
CL	CL-C59:0_C29:0	1300.9	509.5	166	42	4	10.65	2.13	Positive
CL	CL-C59:0_C31:0	1300.9	537.5	166	42	4	10.64	2.13	Positive
CL	CL-C60:0_C29:0	1314.94	509.5	166	42	4	10.7	2.14	Positive
CL	CL-C60:0_C30:0	1314.94	523.5	166	42	4	10.7	2.14	Positive
CL	CL-C61:0_C29:0	1328.96	509.5	166	42	4	10.76	2.15	Positive
CL	CL-C61:0_C30:0	1328.96	523.5	166	42	4	10.77	2.34	Positive
CL	CL-C62:0_C30:0	1342.98	523.5	166	42	4	10.82	2.66	Positive
CL	CL-C62:0_C31:0	1342.98	537.5	166	42	4	10.84	3.17	Positive
CL	CL-C63:0_C31:0	1356.99	537.5	166	42	4	10.9	4.27	Positive
CL	CL-C63:0_C33:0	1356.99	565.5	166	42	4	10.92	2.18	Positive
CL	CL-C64:0_C30:0	1371.01	523.5	166	42	4	11.01	2.2	Positive
CL	CL-C64:0_C31:0	1371.01	537.5	166	42	4	11.1	2.22	Positive
CL	CL-C64:0_C32:0	1371.01	845.5	166	42	4	11.06	4.19	Positive
CL	CL-C64:0_C32:0	1371.01	551.5	166	42	4	11.06	4.19	Positive
CL	CL-C65:0_C31:0	1385.02	537.5	166	42	4	11.23	2.25	Positive
CL	CL-C65:0_C32:0	1385.02	551.5	166	42	4	11.23	3.88	Positive
CL	CL-C66:0_C31:0	1399.04	537.5	166	42	4	11.42	2.29	Positive
CL	CL-C66:0_C33:0	1399.04	565.6	166	42	4	11.38	2.28	Positive
CL	CL-C66:0_C34:0	1399.04	579.6	166	42	4	11.38	2.28	Positive
CL	CL-C67:0_C31:0	1413.05	537.5	166	42	4	11.41	2.28	Positive
CL	CL-C67:0_C32:0	1413.05	551.5	166	42	4	11.43	2.29	Positive
CL	CL-C67:0_C33:0	1413.05	565.6	166	42	4	11.39	2.28	Positive
CL	CL-C68:0_C34:0	1427.07	579.6	166	42	4	11.62	2.33	Positive
DAG	DAG_C24:0	474.42	439.42	166	25	4	6	2	Positive
DAG	DAG_C25:0	488.43	453.43	166	25	4	6.16	1.23	Positive
DAG	DAG_C26:0	502.45	467.45	166	25	4	6.5	2	Positive
DAG	DAG_C27:0	516.46	481.46	166	25	4	7	2	Positive
DAG	DAG_C28:0	530.48	495.48	166	25	4	7.33	2	Positive
DAG	DAG_C29:0	544.49	509.49	166	25	4	8.09	1.8	Positive
DAG	DAG_C30:0	558.51	523.51	166	25	4	8.69	1.9	Positive
DAG	DAG_C31:0	572.53	537.53	166	25	4	9.35	2.17	Positive
DAG	DAG_C32:0	586.54	551.54	166	25	4	9.94	1.99	Positive
DAG	DAG_C33:0	600.56	565.56	166	25	4	10.07	2.01	Positive
DAG	DAG_C34:0	614.57	579.57	166	25	4	10.17	2.03	Positive
DAG	DAG_C35:0	628.59	593.59	166	25	4	10.27	2.05	Positive
DAG	DAG_C36:0	642.6	607.6	166	25	4	10.38	2.08	Positive
DAG	DAG_C37:0	656.62	621.62	166	25	4	10.41	2.08	Positive
DAG	DAG_C38:0	670.64	635.64	166	25	4	10.51	2.1	Positive
FFA	FA(08:0)	143.2	143.2	166	0	4	1	4	Negative
FFA	FA(09:0)	157.2	157.2	166	0	4	2.1	4	Negative
FFA	FA(10:0)	171.2	171.2	166	0	4	2.3	4	Negative
FFA	FA(11:0)	185.2	185.2	166	0	4	2.5	4	Negative
FFA	FA(12:0)	199.2	199.2	166	0	4	2.8	4	Negative
FFA	FA(14:0)	227.2	227.2	166	0	4	3.1	4	Negative
FFA	FA(14:0)	227.2	227.2	166	0	4	3.1	4	Negative
FFA	FA(15:0)	241.2	241.2	166	0	4	3.5	4	Negative
FFA	FA(16:0)	255.2	255.2	166	0	4	3.915	4	Negative
FFA	FA(17:0)	269.3	269.2	166	0	4	3.925	4	Negative
FFA	FA(18:0)	283.3	283.3	166	0	4	4.652	4	Negative
FFA	FA(19:0)	297.3	297.3	166	0	4	5	4	Negative
LPE	LPE_C08:0	342.23	201.2	166	17	5	1.28	1.5	Positive
LPE	LPE_C09:0	356.23	215.2	166	17	5	2	1.5	Positive
LPE	LPE_C10:0	370.23	229.2	166	17	5	2	1.5	Positive
LPE	LPE_C11:0	384.23	243.2	166	17	5	2	1.5	Positive
LPE	LPE_C12:0	398.23	257.2	166	17	5	2.04	1.5	Positive
LPE	LPE_C13:0	412.25	271.2	166	17	5	2	1.5	Positive
LPE	LPE_C14:0	426.26	285.3	166	17	5	1.94	1.5	Positive
LPE	LPE_C15:0	440.28	299.3	166	17	5	2.25	1.5	Positive
LPE	LPE_C16:0	454.29	313.3	166	17	5	2.76	1.5	Positive
LPE	LPE_C17:0	468.31	327.3	166	17	5	3.05	1.5	Positive
LPE	LPE_C18:0	482.32	341.3	166	17	5	2.98	1.5	Positive
LPE	LPE_C19:0	496.33	355.3	166	17	5	3.73	1.5	Positive
LPE	LPE_C20:0	510.36	369.4	166	17	5	4	1.5	Positive
LyPG	LyPG_C24:0	739.488	439.3	166	41	4	5	2.14	Positive
LyPG	LyPG_C25:0	753.503	453.3	166	41	4	5	1.72	Positive
LyPG	LyPG_C26:0	767.519	467.3	166	41	4	4.36	2.05	Positive
LyPG	LyPG_C27:0	781.535	481.3	166	41	4	5	2.03	Positive
LyPG	LyPG_C28:0	795.55	495.4	166	41	4	5	2.21	Positive
LyPG	LyPG_C29:0	809.566	509.4	166	41	4	5.43	1.09	Positive
LyPG	LyPG_C30:0	823.582	523.4	166	41	4	5.88	1.33	Positive
LyPG	LyPG_C31:0	837.597	537.4	166	41	4	6.36	1.27	Positive

LyPG	LyPG_C32:0	851.613	551.4	166	41	4	6.81	2.12	Positive
LyPG	LyPG_C33:0	865.629	565.4	166	41	4	7	2.3	Positive
LyPG	LyPG_C34:0	879.644	579.4	166	41	4	7.2	2.32	Positive
LyPG	LyPG_C35:0	893.66	593.5	166	41	4	7.2	2.34	Positive
LyPG	LyPG_C36:0	907.676	607.5	166	41	4	7.3	2.37	Positive
LyPG	LyPG_C37:0	921.691	621.5	166	41	4	8	2.16	Positive
LyPG	LyPG_C38:0	935.707	635.5	166	41	4	9	2.41	Positive
PA	PA_C24:0	554.38	439.38	166	13	4	4.47	3	Positive
PA	PA_C25:0	568.4	453.4	166	13	4	3.02	3	Positive
PA	PA_C26:0	582.41	467.41	166	13	4	4.71	3	Positive
PA	PA_C27:0	596.43	481.43	166	13	4	5	3	Positive
PA	PA_C28:0	610.45	495.45	166	13	4	5.84	3	Positive
PA	PA_C29:0	624.46	509.46	166	13	4	6.81	3	Positive
PA	PA_C30:0	638.48	523.48	166	13	4	7.07	3	Positive
PA	PA_C31:0	652.49	537.49	166	13	4	7.82	3	Positive
PA	PA_C32:0	666.51	551.51	166	13	4	8.39	3	Positive
PA	PA_C33:0	680.52	565.52	166	13	4	9.88	3	Positive
PA	PA_C34:0	694.54	579.54	166	13	4	10	3	Positive
PA	PA_C35:0	708.55	593.55	166	13	4	11	3	Positive
PA	PA_C36:0	722.57	607.57	166	13	4	11	3	Positive
PA	PA_C37:0	736.59	621.59	166	13	4	11.5	3	Positive
PA	PA_C38:0	750.6	635.6	166	13	4	11.5	3	Positive
PE	PE_C24:0	580.4	439.4	166	19	4	4.1	1.09	Positive
PE	PE_C25:0	594.41	453.41	166	19	4	4.4	0.88	Positive
PE	PE_C26:0	608.43	467.43	166	19	4	4.6	1.6	Positive
PE	PE_C27:0	622.45	481.45	166	19	4	4.97	1.29	Positive
PE	PE_C28:0	636.46	495.46	166	19	4	5.33	2.2	Positive
PE	PE_C29:0	650.48	509.48	166	19	4	5.78	2.45	Positive
PE	PE_C30:0	664.49	523.49	166	19	4	6.2	2.29	Positive
PE	PE_C31:0	678.51	537.51	166	19	4	6.71	3.25	Positive
PE	PE_C32:0	692.52	551.52	166	19	4	7.22	2.49	Positive
PE	PE_C33:0	706.54	565.54	166	19	4	7.98	2.09	Positive
PE	PE_C34:0	720.55	579.55	166	19	4	8.41	2.34	Positive
PE	PE_C35:0	734.57	593.57	166	19	4	7.09	2.24	Positive
PE	PE_C36:0	748.59	607.59	166	19	4	9.65	1.93	Positive
PE	PE_C37:0	762.6	621.6	166	19	4	10.06	2.01	Positive
PE	PE_C38:0	776.62	635.62	166	19	4	10.18	2.04	Positive
PG	PG_C24:0	628.42	439.42	166	17	4	10.16	2.03	Positive
PG	PG_C25:0	642.43	453.43	166	17	4	3.7	1.15	Positive
PG	PG_C26:0	656.45	467.45	166	17	4	3.86	1.2	Positive
PG	PG_C27:0	670.47	481.47	166	17	4	4.09	1.16	Positive
PG	PG_C28:0	684.48	495.48	166	17	4	4.32	1.58	Positive
PG	PG_C29:0	698.5	509.5	166	17	4	4.61	1.87	Positive
PG	PG_C30:0	712.51	523.51	166	17	4	4.89	1.83	Positive
PG	PG_C31:0	726.53	537.53	166	17	4	5.24	2.23	Positive
PG	PG_C32:0	740.54	551.54	166	17	4	5.59	2.03	Positive
PG	PG_C33:0	754.56	565.56	166	17	4	6.15	1.66	Positive
PG	PG_C34:0	768.58	579.58	166	17	4	6.48	2.3	Positive
PG	PG_C35:0	782.59	593.59	166	17	4	5.51	1.5	Positive
PG	PG_C36:0	796.61	607.61	166	17	4	7.49	1.65	Positive
PG	PG_C37:0	810.62	621.62	166	17	4	8.16	1.63	Positive
PG	PG_C38:0	824.64	635.64	166	17	4	8.69	1.74	Positive
Std	LyPG_C36:2	903.644	603.4	166	41	4	7.5	2.29	Positive
Std	15:0-18:1(d7) DAG	605.55	299.5	166	25	4	7.47	1.5	Positive
Std	15:0-18:1(d7) PA (Na Salt)	685.6	570.6	166	13	5	10.5	3	Positive
Std	15:0-18:1(d7) PE	711.6	570.5	166	19	5	7.15	1.8	Positive
Std	15:0-18:1(d7) PG (Na Salt)	759.6	570.6	166	17	4	4.63	1.65	Positive
Std	18:1(d7) Lyso PE	487.3	346.2	166	17	5	2.98	2	Positive
Std	CL 72:4 d5 (IS)	1472.2	599.5	166	45	5	13.8	1.2	Positive
Std	FA(13:0)	213.2	213.2	166	0	4	3.1	4	Negative
