# Supplementary material for: A Bacillales-specific tubular scaffold essential for NADH dehydrogenase activity
Source: Nat Commun. 2026 Jun 18;17:7717. doi: 10.1038/s41467-026-74385-2 (PMC13433803; doi:10.1038/s41467-026-74385-2)
Supplement: Supplementary file 2 [file 41467_2026_74385_MOESM2_ESM.pdf]

nature portfolio

Peer Review File

A Bacillales-specific tubular scaffold essential for NADH dehydrogenase activity

Corresponding Author: Dr Axel Magalon

This file contains all reviewer reports in order by version, followed by all author rebuttals in order by version.

Version 0:

Reviewer comments:

Reviewer #1

(Remarks to the Author)
The manuscript presents an important addition (NDH-II enzyme) to the recently discovered family of redox proteins interacting with quinones via associated hydrophobic tunnels. It will be of interest to the wide research community, data is well presented, it is clearly written and needs only minor revisions:

Fig. 1 is very difficult to read – this data should be reformatted in some way or put into supplement.

L. 171 – what is the evidence that C-terminal helices of YjlC actually interact with membrane? Protein was purified without any detergent treatment, so any association with membrane must be rather weak. This should be discussed in detail.

Reviewer #2

(Remarks to the Author)
NDH-II NADH-quinone oxidoreductases are ubiquitous and are usually monotopic membrane proteins. The authors have identified an NDH-II in Bacillus subtilis, Ndh, that associates with a helical membrane plugin, yjlC. By genomic analysis they showed that this association is specific for Bacillales. Both proteins are essential for NADH oxidation. Cryo-EM maps showed that the proteins form large tubular structures containing NDH-2 tetramers flanked by tubes of four YjlC units, acting as conduits for quinones and lipids over long distances.

The authors have used state-of-the-art techniques and the paper is well-written. It will be of interest to a large audience. I have just a few small suggestions for improvements.

Minor issues:

Line 371, quinone extraction: I don't understand the word "essentially" here. Either remove or move (essentially as described?).

Line 395: 200 kV, not 200KV. 50,000, not 50.000.

Line 491: it looks like the equation on line 496 has to follow here, but it has been moved down to the end of the paragraph.

Line 502: bracket to remove.

Line 505-6: Titan Krios with a cold FEG.

Line 525-531: This section has several inaccuracies. Homogeneous, not homogenous, refinement. C4 symmetry was applied during refinement, not at a later stage. It's a Coulomb potential map without "density". The resolution estimate is not influenced by the B-factor sharpening as suggested here, but by applying a mask in the same step. I suggest to rewrite something like:

"...to calculate an initial 3D reconstruction using a homogeneous refinement job, applying C4 symmetry. This led to a map with a resolution of 2.68 Å, which was sharpened with a B factor of −127.1 Å². Next, a reference-based motion correction job was performed, and the final map, applying C4 symmetry, had a resolution of 2.59 Å using the Fourier shell correlation (FSC) criterion of 0.143 and was sharpened with a B factor of −130.1 Å²." I could not find the local resolution map. It should be added to figure S7.

Line 640: I would simply say "cryo-EM densities".

Figure 3e: how was the membrane position identified?

wwPDB Validation reports, last page:
EMD-55049: author reported resolution: 3.61 Å; from FSC curve: 3.04 Å.
EMD-55050: author reported resolution: 3.04 Å; from FSC curve: 3.61 Å.
This suggests that the authors may accidentally have exchanged something. They should check if it's just the reported resolution or something more serious, and request a correction from the wwPDB.

Reviewer #3

(Remarks to the Author)
The manuscript 'A Bacillales-specific tubular scaffold essential for NADH dehydrogenase activity' by Seduk et al. describes an interaction of an NDH-II enzyme with membrane-bound redox elements facilitated by a tubular intermediate made up of YjlC/HMP (helical membrane plug) proteins. The authors present a phylogenetic approach to isolate possible partner proteins that may be involved in coordinating NDH-II binding to membranes. From here, the authors demonstrate that one of the associated proteins, HMP, is tightly associated with NDH-II in B. subtilis and is essential for NADH-based growth. This is followed by protein purification and assembly analysis through biochemical and structural work (cryo-EM), where the overall architecture is described to a high resolution; however, no native substrates could be confidently identified.

Overall, the work is robust and adds to the portfolio of redox enzymes mediated to membranes via hydrophobic tubular protein assemblies. I only have minor comments requesting further information on certain aspects that are mentioned during the manuscript, and there are some points that I believe should be addressed in the Discussion.

Specific comments:

1. Line 79: "To explore the sequence diversity of NDH-II and identify potential partners" is vague with respect to 'partners' in this part of the manuscript. Add something specific about the search for membrane attachment mediators, that was briefly mentioned in the introduction. Adding a sentence on the logic behind this approach, i.e., partner proteins are likely located in the same region/are conserved in a genome in a similar manner will also be of benefit.

2. Figure 1 is very hard to read both the text and the symbols due to the small font size and restriction to the page length. It may be worth considering reducing the information into a more schematic and appropriate format for a main text figure and this original figure can then be included as an SI figure and/or SI data only.

3. Figure 3 shows a "low resolution surface representation". Please state the resolution value and if it was a result of a modification to your final map (filtering).

4. Figure 8 suggests that some of the spots are pink, but given the small size of the symbols in the image, this is difficult to see. Consider making panel (a) the full-page width and/or increasing the symbol sizes. Axes labels can also be increased in size. Also, panel (c) has repeated spelling error "residus".

5. On line 118, other NDH-II enzymes (YumB, YutJ) are mentioned but not explored due to not being co-localised with YjlC. Why do the authors think these are not sufficient to sustain growth in the Δndh variant, despite having conserved catalytic motifs?

6. On line 136, the authors state "UV–visible spectra showed characteristic flavoprotein absorption peaks at 375 and 450 nm, corresponding to ~0.5 FAD per complex (Fig. S2b)." - please add the extinction coefficient(s) for the flavin and an appropriate reference.

7. On line 144, the authors compare their NADH:DCPIP reaction to previous papers, suggesting this one has a higher catalytic efficiency from previously characterised - but those papers do not use the same electron acceptor, they use quinones/quinone analogues. The comparison is therefore not valid as rate determining steps may vary between the substrates and it is not appropriate to make the conclusion about higher efficiency. To do the comparison the authors could use the same substrates as used in the other papers for a like-for-like comparison.

8. On line 165, the authors state "These units stack head-to-tail with a translation of ~97.5 Å and a 45° rotation, creating a polarized fiber that can extend to as many as ten $\alpha_4\beta_4$ units (Fig. 3a)", but do not state how these were counted. Is this based on micrograph images, or is it some other method?

9. From line 198, the authors describe the local appearance of the N-terminus of the fiber and conclude that there appears to be nothing occupying the opening. The authors do not posit a suggestion for why this might be. Is it solvent exposed and

solvent filled? An issue with the targeted classification of the N-terminus?

10. From line 216, the authors include calculations about free energy of dissociation, but do not include information in the methods about where the contact areas and calculations were performed (software).

11. Line 505 typo. “Titan Kryos” and not ‘Krios’.

12. Alphafold3 (initial model and those with ligands) is not cited in the references. Please add.

13. Figure S2 does not state what the error bars are for c) and d) in the legend. Also, there are green symbols on e) that are without explanation.

General comments:

The authors included a purification tag and then performed an overexpression of the complex. It is notable that the micrograph images showed variable length complexes for both organisms used (line 158), but how physiologically relevant is this? Is it possible that this is a result of overexpression? Are there any issues regarding quinone sequestration the authors can discuss, considering distances of 1000Å from the membrane?

The authors mention that hydrophobic tunnels act as conduits to facilitate the exchange of quinones, but they do not discuss why some organisms have these and not others. Comments and hypotheses may add interest to the Discussion, and can highlight previous suggestions. (Is it due to membrane protein crowding? Isolation of quinones from the membrane to stop the redox enzymes have unwanted chemical reactions with other, less hydrophobic species in the membrane? Adding NADH antennae, similar to that proposed for complex I extended hydrophilic domain?)

Reviewer #4

(Remarks to the Author)
I co-reviewed this manuscript with one of the reviewers who provided the listed reports. This is part of the Nature Communications initiative to facilitate training in peer review and to provide appropriate recognition for Early Career Researchers who co-review manuscripts.

Version 1:

Reviewer comments:

Reviewer #1

(Remarks to the Author)
The manuscript has been revised well and can be accepted.

Reviewer #2

(Remarks to the Author)
The authors have addressed all issues and I support publication of the paper.

Reviewer #3

(Remarks to the Author)
The changes the authors have made to the manuscript have addressed our earlier concerns.

Reviewer #4

(Remarks to the Author)
I co-reviewed this manuscript with one of the reviewers who provided the listed reports. This is part of the Nature Communications initiative to facilitate training in peer review and to provide appropriate recognition for Early Career Researchers who co-review manuscripts.

Reviewer #5

(Remarks to the Author)
I agree with all other reviewers and my comments are mainly focused on lipidomic analyses:
1. For lipidomic analysis method (Line 397-407): the authors referred to two references instead of providing detailed methods. However, the two references used different methods. It is not clear what exact methods were used for lipidomic analyses.
2. This is a targeted lipidomics analysis, what lipid targets were analyzed? What mass spec, GC or LC method/parameters used? Were all reported lipid species quantified by their own internal standards? The authors should provide more

information about the method. If refer to other paper, should be more clear about which paper and which part of that paper.
3. On Fig S4: b is CL and c is PG. This is not consistent with Fig S4's figure legend (line 742).
4. On Fig S4b and c: prefer to delete "Fatty acids" because the name of CL or PG including their fatty acids have been clearly shown.

Reviewer #6

(Remarks to the Author)
In their manuscript "A Bacillales-specific tubular scaffold essential for NADH dehydrogenase activity" Seduk et al. convincingly show the co-dependence between Ndh and YjlC at a genomic, structural, and functional level using detailed phylogenetic, mutagenic, structural, functional and mass spectrometric analyses. CryoEM data accompanied by MS lipidomic analyses and docking simulations support the authors' argument for the role of the Ndh-YjlC scaffold to act as hydrophobic channels. The methods in the paper complemented each other very well to build a compelling story one the specialized architecture of NDH-II catalysis in Bacillales. This manuscript will be of broad interest to the fields of bacterial metabolism, bioenergetics, structural biology, oxidoreductase mechanism and evolution of metabolic complexes.

The authors satisfactorily answered the questions raised by the previous reviewers. The paper was well-written and easy to read.

Strengths:
The combination of bioinformatics, mutagenesis, enzymology, lipidomics and cryoEM, combine to provide significant insight into the mechanism of NADH:quinone oxidoreduction in Bacillales. This manuscript significantly adds to our understanding of evolutionary strategies to maximize metabolic flux in bacteria, providing an additional example of tubular arrays of oxidoreductases anchored to the membrane.

Weaknesses:
No significant weaknesses identified.

One minor points to be addressed
What were the models used in the initial C1 heterogeneous refinement?

Very minor typographic comments for later consideration:

1. Inconsistent use of periods vs commas to signify a decimal point throughout the manuscript. Although this is a stylistic choice, please stick to a format to avoid confusion (lines 150 kcat/KM=19,3) (Fig 8a y-axis), and particularly the extinction coefficient in methods: DCPIP (21.000 M-1cm-1) and NADH (6.220 M-1cm-1) vs FAD (11,300 M-1cm-1)

2. Line 424: n-terminal should be N-terminal

3. Line 326: (first sentence in section Methods/strain construction): missing a parenthesis. (trpC2 …)

4. Figure 8b, top panel. For consistency, "Hmp" should be "HMP" on the right-hand y-axis

5. Lines 228-231, Greek letters are corrupted in our version of the manuscript and appear as squares.

6. Lines 394, under Methods/SEC-MALS analysis: consider putting the concentrations first before the chemical name for consistency (Tris 50 mM to 50 mM Tris).

Reviewer #7

(Remarks to the Author)
I co-reviewed this manuscript with one of the reviewers who provided the listed reports. This is part of the Nature Communications initiative to facilitate training in peer review and to provide appropriate recognition for Early Career Researchers who co-review manuscripts.

Version 2:

Reviewer comments:

Reviewer #5

(Remarks to the Author)
The authors have addressed all issues and I support publication of the paper.

Reviewer #6

(Remarks to the Author)

The authors have satisfactorily address all our concerns.

Reviewer #7

(Remarks to the Author)
I co-reviewed this manuscript with one of the reviewers who provided the listed reports. This is part of the Nature Communications initiative to facilitate training in peer review and to provide appropriate recognition for Early Career Researchers who co-review manuscripts.

Open Access This Peer Review File is licensed under a Creative Commons Attribution 4.0 International License, which permits use, sharing, adaptation, distribution and reproduction in any medium or format, as long as you give appropriate credit to the original author(s) and the source, provide a link to the Creative Commons license, and indicate if changes were made.
In cases where reviewers are anonymous, credit should be given to 'Anonymous Referee' and the source.
The images or other third party material in this Peer Review File are included in the article's Creative Commons license, unless indicated otherwise in a credit line to the material. If material is not included in the article's Creative Commons license and your intended use is not permitted by statutory regulation or exceeds the permitted use, you will need to obtain permission directly from the copyright holder.
To view a copy of this license, visit https://creativecommons.org/licenses/by/4.0/

REVIEWER COMMENTS

Reviewer #1 (Remarks to the Author):

The manuscript presents an important addition (NDH-II enzyme) to the recently discovered family of redox proteins interacting with quinones via associated hydrophobic tunnels. It will be of interest to the wide research community, data is well presented, it is clearly written and needs only minor revisions:

[Our response] We thank the reviewer for their very positive assessment of our work

Fig. 1 is very difficult to read – this data should be reformatted in some way or put into supplement.

[Our response] : We agreed and simplified Figure 1. We collapsed most of the clades and kept only the NDH-HMP neighboring clades. The text is now readable. A supplementary figure with the full phylogenies has been added (Figure S2).

L. 171 – what is the evidence that C-terminal helices of YjlC actually interact with membrane? Protein was purified without any detergent treatment, so any association with membrane must be rather weak. This should be discussed in detail.

[Our response] We thank the reviewer for their comment. In our previous structural analysis of ForCE, we showed that the HMP domain is amphipathic and engages in transient peripheral interactions with the membrane. In the cryo-EM map, the C-terminal end of the HMP tube is capped by elongated densities modelled as additional fatty acids, and the positively charged surface of the domain is ideally positioned to interact with the negatively charged lipid headgroups. As discussed in that study, this excludes any deep membrane insertion and is consistent with the purification of ForCE (and Huc) in the soluble fraction.

In the NDH system described here, the HMP tube displays the same amphipathic organisation and C-terminal arrangement, connecting the dehydrogenase to the membrane through an interface highly similar to that observed in ForCE. The fact that NDH, like ForCE and Huc, can be isolated without detergent further supports a model based on weak, reversible peripheral interactions rather than stable membrane embedding.

Taken together, the conserved amphipathic properties of the HMP domain and the recurrence of the same structural features across systems strongly support our interpretation that YjlC interacts with the membrane through transient HMP-mediated peripheral contacts, consistent with our previously published conclusions. This comment is now included in the results section : "*Consistent with this, the architecture*

and physicochemical properties of the HMP domain support transient, peripheral membrane contacts, similar to those previously observed in ForCE."

Reviewer #2 (Remarks to the Author):

NDH-II NADH-quinone oxidoreductases are ubiquitous and are usually monotopic membrane proteins. The authors have identified an NDH-II in Bacillus subtilis, Ndh, that associates with a helical membrane plugin, yjlC. By genomic analysis they showed that this association is specific for Bacillales. Both proteins are essential for NADH oxidation. Cryo-EM maps showed that the proteins form large tubular structures containing NDH-2 tetramers flanked by tubes of four YjlC units, acting as conduits for quinones and lipids over long distances.

The authors have used state-of-the-art techniques and the paper is well-written. It will be of interest to a large audience. I have just a few small suggestions for improvements.

[Our response] We thank the reviewer for their positive and constructive evaluation of our work

Minor issues:

Line 371, quinone extraction: I don't understand the word "essentially" here. Either remove or move (essentially as described?).

[Our response] Corrected. The word has been removed.

Line 395: 200 kV, not 200KV. 50,000, not 50.000.

[Our response] Done

Line 491: it looks like the equation on line 496 has to follow here, but it has been moved down to the end of the paragraph.

[Our response] Done

Line 502: bracket to remove.

[Our response] Done

Line 505-6: Titan Krios with a cold FEG.

[Our response] Done

Line 525-531: This section has several inaccuracies. Homogeneous, not homogenous, refinement. C4 symmetry was applied during refinement, not at a later stage. It's a Coulomb potential map without "density". The resolution estimate is not influenced by the B-factor sharpening as suggested here, but by applying a mask in the same step. I suggest to rewrite something like:
"...to calculate an initial 3D reconstruction using a homogeneous refinement job, applying C4 symmetry. This led to a map with a resolution of 2.68 Å, which was sharpened with a B factor of −127.1 Å². Next, a reference-based motion correction job was performed, and the final map, applying C4 symmetry, had a resolution of 2.59 Å using the Fourier shell correlation (FSC) criterion of 0.143 and was sharpened with a B factor of −130.1 Å²." I could not find the local resolution map. It should be added to figure S7.

[Our response] We thank the reviewer for this clarification of the text which has been modified accordingly and a supplementary figure showing the local resolution map has been added (Figure S9).

Line 640: I would simply say "cryo-EM densities".

[Our response] Done

Figure 3e: how was the membrane position identified?

[Our response] The answer to this question has already been provided to reviewer #1, see above.

wwPDB Validation reports, last page:
EMD-55049: author reported resolution: 3.61 Å; from FSC curve: 3.04 Å.
EMD-55050: author reported resolution: 3.04 Å; from FSC curve: 3.61 Å.
This suggests that the authors may accidentally have exchanged something. They should check if it's just the reported resolution or something more serious, and request a correction from the wwPDB.

[Our response] We thank the reviewer for pointing out this inversion. When we deposited our structural data to the EMDB, we inadvertently swapped the maps for two datasets: that is, the deposition corresponding to the cryo-EM data of the HMP end includes the map from the N-terminal region dataset, and vice versa. This has now been corrected.

Reviewer #3 (Remarks to the Author):

The manuscript 'A Bacillales-specific tubular scaffold essential for NADH dehydrogenase activity' by Seduk et al. describes an interaction of an NDH-II enzyme

with membrane-bound redox elements facilitated by a tubular intermediate made up of YjlC/HMP (helical membrane plug) proteins. The authors present a phylogenetic approach to isolate possible partner proteins that may be involved in coordinating NDH-II binding to membranes. From here, the authors demonstrate that one of the associated proteins, HMP, is tightly associated with NDH-II in B. subtilis and is essential for NADH-based growth. This is followed by protein purification and assembly analysis through biochemical and structural work (cryo-EM), where the overall architecture is described to a high resolution; however, no native substrates could be confidently identified.

Overall, the work is robust and adds to the portfolio of redox enzymes mediated to membranes via hydrophobic tubular protein assemblies. I only have minor comments requesting further information on certain aspects that are mentioned during the manuscript, and there are some points that I believe should be addressed in the Discussion.

[Our response] We thank the reviewer for their positive and encouraging assessment of our study.

Specific comments:

1. Line 79: "To explore the sequence diversity of NDH-II and identify potential partners" is vague with respect to 'partners' in this part of the manuscript. Add something specific about the search for membrane attachment mediators, that was briefly mentioned in the introduction. Adding a sentence on the logic behind this approach, i.e., partner proteins are likely located in the same region/are conserved in a genome in a similar manner will also be of benefit.

[Our response] We thank the reviewer for raising this point of clarification of our approach. This has now been included in the text as the following "*To explore the sequence diversity of NDH-II and to identify additional protein partners likely to mediate membrane association and thus provide NDH-II access to the quinone pool, we used a phylogenetic approach combined with comparative genomics. Because such accessory components are expected to functionally cooperate with NDH-II, we reasoned that they would most likely be encoded by genes located in close proximity to ndh.*"

2. Figure 1 is very hard to read both the text and the symbols due to the small font size and restriction to the page length. It may be worth considering reducing the information into a more schematic and appropriate format for a main text figure and this original figure can then be included as an SI figure and/or SI data only.

[Our response] According to the reviewer's suggestion, we simplified Figure 1, with most of the clades being collapsed. We only kept the NDH-HMP neighboring clades

as uncollapsed, allowing the text to be readable. A supplementary figure with the full phylogenies has been added (Figure S2).

3. Figure 3 shows a "low resolution surface representation". Please state the resolution value and if it was a result of a modification to your final map (filtering).

[Our response] We thank the reviewer for their comment. Indeed, Figure 3A shows a schematic representation of the surface of the YjlC-Ndh fiber. This surface was generated from the protein model after applying a 10 Å Gaussian filter using the "new_map" procedure in PyMOL. We have clarified the figure legend accordingly: "*Schematic representation of YjlC-Ndh fibre showing three YjlC4–Ndh4 units. The low resolution surface corresponds to a surface representation of the fiber protein model generated with a 10 Å Gaussian filter*."

4. Figure 8 suggests that some of the spots are pink, but given the small size of the symbols in the image, this is difficult to see. Consider making panel (a) the full-page width and/or increasing the symbol sizes. Axes labels can also be increased in size. Also, panel (c) has repeated spelling error "residus".

[Our response] The figure was modified according to the reviewer's comments. In panel (a), the size of the symbols was increased, and their color was modified to improve the figure's readability. Axes labels were increased. Spelling errors in panel (c) were corrected.

5. On line 118, other NDH-II enzymes (YumB, YutJ) are mentioned but not explored due to not being co-localised with YjlC. Why do the authors think these are not sufficient to sustain growth in the ∆ndh variant, despite having conserved catalytic motifs?

[Our response] We thank the reviewer for their comment. Previous work has shown that *yutJ* and *yumB* are not required for growth on LB plates, as the corresponding single mutants grow comparably to the wild-type strain (10.1128/JB.00601-06). These observations indicate that, under aerobic conditions, whether in rich medium or in the controlled malate medium used here, *yutJ* and *yumB* are likely not expressed at levels sufficient to support NADH oxidation. This interpretation is further supported by transcriptional data showing that *yumB* expression follows a regulatory pattern opposite to that of *ndh* (10.1126/science.1206848), reinforcing the view that neither *yutJ* nor *yumB* contributes significantly to growth in the ∆*ndh* background under the conditions tested.

6. On line 136, the authors state "UV–visible spectra showed characteristic flavoprotein absorption peaks at 375 and 450 nm, corresponding to ~0.5 FAD per complex (Fig. S2b)." - please add the extinction coefficient(s) for the flavin and an appropriate reference.

[Our response] The text has been modified accordingly in the methods : "*FAD quantification : The FAD content of the purified complex was determined by UV-visible spectroscopy. Absorbance at 450 nm was converted to FAD concentration using the molar extinction coefficient of oxidized FAD (ε_{450} = 11,300 M^{-1} cm^{-1}; 10.1042/bj0540437), and the FAD-to-protein ratio was calculated by comparison with independently measured protein concentrations."*

7. On line 144, the authors compare their NADH:DCPIP reaction to previous papers, suggesting this one has a higher catalytic efficiency from previously characterised - but those papers do not use the same electron acceptor, they use quinones/quinone analogues. The comparison is therefore not valid as rate determining steps may vary between the substrates and it is not appropriate to make the conclusion about higher efficiency. To do the comparison the authors could use the same substrates as used in the other papers for a like-for-like comparison.

[Our response] We thank the reviewer for raising this important point. We fully agree with them that comparing catalytic efficiencies across studies using different electron acceptors (e.g., DCPIP vs. quinones or quinone analogues), as well as distinct pH and temperature conditions, is not appropriate because the rate-determining steps may differ. For this reason, we have removed these comparisons from the revised manuscript.

We nevertheless note that, even considering the variability in assay conditions across studies, no NDH-II characterized so far has reported a Km for NADH as low as the one we observe. However, we now present this as an observation rather than as evidence for higher catalytic efficiency, in line with the reviewer's recommendation.

"*Steady-state kinetics of NADH oxidation using DCPIP as an artificial electron acceptor showed Michaelis–Menten behavior with a low turnover number (k_{cat}=27 s^{-1}) but high substrate affinity (K_M^{NADH}=1.4 µM) (Fig. S3c). The very low K_M^{NADH} suggests that the YjlC/Ndh complex is adapted to operate efficiently at low NADH concentrations*."

8. On line 165, the authors state "These units stack head-to-tail with a translation of ~97.5 Å and a 45° rotation, creating a polarized fiber that can extend to as many as ten $\alpha_4\beta_4$ units (Fig. 3a)", but do not state how these were counted. Is this based on micrograph images, or is it some other method?

[Our response] We agree with the reviewer, and the text has been clarified accordingly. The maximum number of fiber units was determined by direct visual inspection of the micrograph images. "*These units stack head-to-tail with a translation of ~97.5 Å and a 45° rotation, creating a polarized fiber that can extend to as many as ten $\alpha_4\beta_4$ units, as observed in micrograph images* (Fig. 3a)."

9. From line 198, the authors describe the local appearance of the N-terminus of the fiber and conclude that there appears to be nothing occupying the opening. The authors do not posit a suggestion for why this might be. Is it solvent exposed and solvent filled? An issue with the targeted classification of the N-terminus?

[Our response] We thank the reviewer for their comment. When comparing the N-terminal end of an octameric unit with the terminal end of the fiber, we consistently observe that, unlike in ForCE, no ordered densities that would correspond to lipids are present inside the YjlC tube. This absence of density does not imply that the region is empty. It is most likely occupied by solvent molecules and/or disordered lipids that remain invisible due to insufficient ordering, a situation also reflected by the overall lower number of ordered internal densities in Ndh2 compared to ForCE.

Importantly, the hydrophobic character of the inner surface strongly supports the presence of lipids within the tube, consistent with our lipidomic data and with the lipid occupancy observed in the equivalent region of ForCE. Therefore, the lack of visible density is best explained by disorder, not by an issue with the targeted classification of particles used to reconstruct the N-terminal end.

10. From line 216, the authors include calculations about free energy of dissociation, but do not include information in the methods about where the contact areas and calculations were performed (software).

[Our response] We used the software PISA to perform this analysis. Although PISA and its reference were already mentioned in the legend of figure 7, we have now added this information explicitly in the main text. "*We next analyzed the interface between Ndh and the YjlC scaffold using PISA (10.1016/j.jmb.2007.05.022).*"

11. Line 505 typo. "Titan Kryos" and not 'Krios'.

[Our response] Done

12. Alphafold3 (initial model and those with ligands) is not cited in the references. Please add.

[Our response] Done

13. Figure S2 does not state what the error bars are for c) and d) in the legend. Also, there are green symbols on e) that are without explanation.

[Our response] Done

General comments:

The authors included a purification tag and then performed an overexpression of the complex. It is notable that the micrograph images showed variable length complexes for both organisms used (line 158), but how physiologically relevant is this? Is it possible that this is a result of overexpression? Are there any issues regarding quinone sequestration the authors can discuss, considering distances of 1000Å from the membrane?

[Our response] Thank you for this comment. We agree that the physiological relevance of the YjlC–Ndh fibers remains uncertain and is currently under investigation. Notably, in our previous work on ForCE, purified under similar overexpression conditions, we did not observe filamentous assemblies, suggesting that fiber formation is not merely an overexpression artefact. Regarding potential quinone sequestration, both the internal dimensions and the physicochemical properties of the YjlC tube, as discussed in the manuscript, are fully compatible with quinone diffusion along the fiber, consistent with mechanisms proposed for ForCE and HucSLM. Thus, even when Ndh molecules are positioned far from the membrane, the architecture of the tube inherently supports their functional connection to the quinone pool.

The authors mention that hydrophobic tunnels act as conduits to facilitate the exchange of quinones, but they do not discuss why some organisms have these and not others. Comments and hypotheses may add interest to the Discussion, and can highlight previous suggestions. (Is it due to membrane protein crowding? Isolation of quinones from the membrane to stop the redox enzymes have unwanted chemical reactions with other, less hydrophobic species in the membrane? Adding NADH antennae, similar to that proposed for complex I extended hydrophilic domain?)

[Our response] We thank the reviewer for this stimulating comment. Interestingly, our findings do not suggest that hydrophobic tunnels are used exclusively by certain organisms; rather, they represent an additional membrane-interaction strategy found across diverse bacteria and archaea, alongside classical modes of quinone access. Some oxidoreductases, such as Ndh-II described here, or previously in ForCE and HucSLM, seem particularly adapted to this architecture, although we do not exclude that others may also exploit it.

A key advantage of this organization is the drastic reduction in membrane surface occupancy: an octameric YjlC–Ndh unit binding four Ndh molecules occupies ~16 nm^2, compared with ~84 nm^2 for four individual Ndh enzymes, and well below if supramolecular fibers exist in vivo or the ~72 nm^2 occupied by Complex I.

Moreover, the tube, whose dimensions and physicochemical properties enable diffusion of quinones and lipids, mimics a functional extension of the membrane. Such

confinement may help channel quinone turnover, maintain local redox efficiency, and limit unwanted reactions with O_2, as proposed for ForCE and HucSLM.

We now highlight these hypotheses in the Discussion.

"*Beyond channeling quinones, the tubular architecture may also offer functional advantages. By clustering several catalytic units around a single HMP scaffold, the assembly drastically reduces membrane surface occupancy, from ~84 nm² for four individual Ndh enzymes to ~16 nm² for the corresponding hetero-octamer unit. For comparison, Complex I, which catalyzes the same NADH oxidation reaction, occupies ~72 nm² of membrane surface (10.1038/nature11871, 10.1038/nature09066). In addition, the dimensions and physicochemical properties of the lumen, which behaves as an extension of the membrane, confine quinone turnover, thereby enabling efficient electron transfer while limiting unwanted reactions with molecular oxygen. These functional implications remain hypothetical and warrant future investigation.*"

Reviewer #4 (Remarks to the Author):

I co-reviewed this manuscript with one of the reviewers who provided the listed reports. This is part of the Nature Communications initiative to facilitate training in peer review and to provide appropriate recognition for Early Career Researchers who co-review manuscripts.

REVIEWER COMMENTS

Reviewer #1 (Remarks to the Author):

The manuscript has been revised well and can be accepted.

[Our response] We thank the reviewer for the very positive assessment of our work

Reviewer #2 (Remarks to the Author):

The authors have addressed all issues and I support publication of the paper.

[Our response] We thank the reviewer for the very positive assessment of our work

Reviewer #3 (Remarks to the Author):

The changes the authors have made to the manuscript have addressed our earlier concerns.

[Our response] We thank the reviewer for the very positive assessment of our work

Reviewer #4 (Remarks to the Author):

I co-reviewed this manuscript with one of the reviewers who provided the listed reports. This is part of the Nature Communications initiative to facilitate training in peer review and to provide appropriate recognition for Early Career Researchers who co-review manuscripts.

[Our response] We thank the reviewer for the very positive assessment of our work

Reviewer #5 (Remarks to the Author):

I agree with all other reviewers and my comments are mainly focused on lipidomic analyses:

1. For lipidomic analysis method (Line 397-407): the authors referred to two references instead of providing detailed methods. However, the two references used different methods. It is not clear what exact methods were used for lipidomic analyses.
2. This is a targeted lipidomics analysis, what lipid targets were analyzed? What mass spec, GC or LC method/parameters used? Were all reported lipid species quantified by their own internal standards? The authors should provide more information about the method. If refer to other paper, should be more clear about which paper and which part of that paper.

[Our response] We thank the reviewer for raising this point of clarification of our approach. This has now been included in the text as the following: "***Mass spectrometry based-Lipidomic analyses on purified protein***

Total lipid was extracted from the 10 µL of a 6.25 mg/mL purified protein sample or from two negative control proteins that do not bind lipids (namely, catalase 1 and catalase 2). The extraction process utilized 350 µL chloroform/methanol (4:3 v/v) containing 1 nmol each of internal standards (as listed in Table S6), followed by the addition of 140 µL of water to induce biphasic separation. The lower organic phase was collected and subsequently dried in a vial insert. The recovered lipids were dissolved in methanol (50 µL) and aliquot (10 µL) was injected to a Agilent 1290 infinity/Infinity II LCMS system equipped with ZORBAX Eclipse Plus C18, 100 x 2.1 mm, 1.8 um reversed-phase column (Agilent) with Infinity II inline filter, 0.3 um (Agilent) maintained at 45C. Samples were separated by the change in gradient of solvent A (water:acetonitrile:isopropanol, 5:3:2 (v/v/v), 10 mM ammonium formate) and solvent B (isopropanol:acetonitrile:water, 90:9:1 (v/v/v), 10 mM ammonium formate). The gradient was as follows, starting with a flow rate of 0.4 mL/min at 15% B and increasing to 50% over 2.5 min then to 57% at 2.6 min, then to 70% over 9 min and to 93% at 9.1 min, then to 96% over 11 min and to 100% at 11.1 min and hold to 12 min then back to 15% at 12.2 min to 16 min (total of 16 min). MS (Agilent 6495c triple quadrupole) was operated in for targeted analysis with DMRM (dynamic multiple reaction monitoring) with 650 ms of cycle time with following setting: Agilent Jet stream ion source with positive and negative switching with gas temperature at 150°C, drying gas (nitrogen) 17 L/min, Nebulizer gas 20 psi, Sheath gas temperature at 200°C with flow at 10 L/min, capillary voltage 3500 V for positive and -3000 V for negative mode, nozzle voltage at 1000 V for positive and -1500 V for negative mode. As described in Hyunh et al. 2019 (10.1016/j.chembiol.2018.10.008), for targeted lipidomic analysis in B. subtilis, first, collision energy (CE) and characteristic fragmentation for Lysil-phosphatidylglycerol (LyPG), was obtained by Mass Hunter Optimiser software (Agilent) using authentic standard LyPG C18:1 (Sigma). Then the fatty acid combination for each lipid species was confirmed by B. subtilis total lipid using neutral loss ion scan (NL m/z 35 for DAG with CE 35 V; m/z 141 for LPE and PE with CE 19 and 17 V, respectively; m/z 300 for LyPG with CE 41 V; m/z 189 for PG with CE 19 V, m/z 115 for PA with CE 13 V in positive mode). For CL, PG species detected as above was further used to determine CL species in B. subtilis. The resulted LCMS data was combined to pre-existing dynamic multiple reaction monitoring (DMRM) method for optimized targeted lipidomic analysis for B. subtilis (as shown in Table S6).

Table S6: List of internal standards

Cpd Group	Cpd Name	Prec Ion	Prod Ion	Frag (V)	CE (V)	Cell Acc (V)	Ret Time (min)	Ret Window	Polarity
CL	CL-C58:0_C29:0	1286.91	509.5	166	42	4	10.55	2.11	Positive
CL	CL-C58:0_C31:0	1286.91	537.5	166	42	4	8.03	1.61	Positive
CL	CL-C59:0_C29:0	1300.9	509.5	166	42	4	10.65	2.13	Positive
CL	CL-C59:0_C31:0	1300.9	537.5	166	42	4	10.64	2.13	Positive
CL	CL-C60:0_C29:0	1314.94	509.5	166	42	4	10.7	2.14	Positive
CL	CL-C60:0_C30:0	1314.94	523.5	166	42	4	10.7	2.14	Positive
CL	CL-C61:0_C29:0	1328.96	509.5	166	42	4	10.76	2.15	Positive
CL	CL-C61:0_C30:0	1328.96	523.5	166	42	4	10.77	2.34	Positive
CL	CL-C62:0_C30:0	1342.98	523.5	166	42	4	10.82	2.66	Positive

CL	CL-C62:0_C31:0	1342.98	537.5	166	42	4	10.84	3.17	Positive
CL	CL-C63:0_C31:0	1356.99	537.5	166	42	4	10.9	4.27	Positive
CL	CL-C63:0_C33:0	1356.99	565.5	166	42	4	10.92	2.18	Positive
CL	CL-C64:0_C30:0	1371.01	523.5	166	42	4	11.01	2.2	Positive
CL	CL-C64:0_C31:0	1371.01	537.5	166	42	4	11.1	2.22	Positive
CL	CL-C64:0_C32:0	1371.01	845.5	166	42	4	11.06	4.19	Positive
CL	CL-C64:0_C32:0	1371.01	551.5	166	42	4	11.06	4.19	Positive
CL	CL-C65:0_C31:0	1385.02	537.5	166	42	4	11.23	2.25	Positive
CL	CL-C65:0_C32:0	1385.02	551.5	166	42	4	11.23	3.88	Positive
CL	CL-C66:0_C31:0	1399.04	537.5	166	42	4	11.42	2.29	Positive
CL	CL-C66:0_C33:0	1399.04	565.6	166	42	4	11.38	2.28	Positive
CL	CL-C66:0_C34:0	1399.04	579.6	166	42	4	11.38	2.28	Positive
CL	CL-C67:0_C31:0	1413.05	537.5	166	42	4	11.41	2.28	Positive
CL	CL-C67:0_C32:0	1413.05	551.5	166	42	4	11.43	2.29	Positive
CL	CL-C67:0_C33:0	1413.05	565.6	166	42	4	11.39	2.28	Positive
CL	CL-C68:0_C34:0	1427.07	579.6	166	42	4	11.62	2.33	Positive
DAG	DAG_C24:0	474.42	439.42	166	25	4	6	2	Positive
DAG	DAG_C25:0	488.43	453.43	166	25	4	6.16	1.23	Positive
DAG	DAG_C26:0	502.45	467.45	166	25	4	6.5	2	Positive
DAG	DAG_C27:0	516.46	481.46	166	25	4	7	2	Positive
DAG	DAG_C28:0	530.48	495.48	166	25	4	7.33	2	Positive
DAG	DAG_C29:0	544.49	509.49	166	25	4	8.09	1.8	Positive
DAG	DAG_C30:0	558.51	523.51	166	25	4	8.69	1.9	Positive
DAG	DAG_C31:0	572.53	537.53	166	25	4	9.35	2.17	Positive
DAG	DAG_C32:0	586.54	551.54	166	25	4	9.94	1.99	Positive
DAG	DAG_C33:0	600.56	565.56	166	25	4	10.07	2.01	Positive
DAG	DAG_C34:0	614.57	579.57	166	25	4	10.17	2.03	Positive
DAG	DAG_C35:0	628.59	593.59	166	25	4	10.27	2.05	Positive
DAG	DAG_C36:0	642.6	607.6	166	25	4	10.38	2.08	Positive
DAG	DAG_C37:0	656.62	621.62	166	25	4	10.41	2.08	Positive
DAG	DAG_C38:0	670.64	635.64	166	25	4	10.51	2.1	Positive
FFA	FA(08:0)	143.2	143.2	166	0	4	1	4	Negative
FFA	FA(09:0)	157.2	157.2	166	0	4	2.1	4	Negative
FFA	FA(10:0)	171.2	171.2	166	0	4	2.3	4	Negative
FFA	FA(11:0)	185.2	185.2	166	0	4	2.5	4	Negative
FFA	FA(12:0)	199.2	199.2	166	0	4	2.8	4	Negative
FFA	FA(14:0)	227.2	227.2	166	0	4	3.1	4	Negative
FFA	FA(14:0)	227.2	227.2	166	0	4	3.1	4	Negative
FFA	FA(15:0)	241.2	241.2	166	0	4	3.5	4	Negative
FFA	FA(16:0)	255.2	255.2	166	0	4	3.915	4	Negative
FFA	FA(17:0)	269.3	269.2	166	0	4	3.925	4	Negative
FFA	FA(18:0)	283.3	283.3	166	0	4	4.652	4	Negative
FFA	FA(19:0)	297.3	297.3	166	0	4	5	4	Negative
LPE	LPE_C08:0	342.23	201.2	166	17	5	1.28	1.5	Positive
LPE	LPE_C09:0	356.23	215.2	166	17	5	2	1.5	Positive
LPE	LPE_C10:0	370.23	229.2	166	17	5	2	1.5	Positive
LPE	LPE_C11:0	384.23	243.2	166	17	5	2	1.5	Positive
LPE	LPE_C12:0	398.23	257.2	166	17	5	2.04	1.5	Positive
LPE	LPE_C13:0	412.25	271.2	166	17	5	2	1.5	Positive
LPE	LPE_C14:0	426.26	285.3	166	17	5	1.94	1.5	Positive
LPE	LPE_C15:0	440.28	299.3	166	17	5	2.25	1.5	Positive
LPE	LPE_C16:0	454.29	313.3	166	17	5	2.76	1.5	Positive
LPE	LPE_C17:0	468.31	327.3	166	17	5	3.05	1.5	Positive
LPE	LPE_C18:0	482.32	341.3	166	17	5	2.98	1.5	Positive
LPE	LPE_C19:0	496.33	355.3	166	17	5	3.73	1.5	Positive
LPE	LPE_C20:0	510.36	369.4	166	17	5	4	1.5	Positive
LyPG	LyPG_C24:0	739.488	439.3	166	41	4	5	2.14	Positive
LyPG	LyPG_C25:0	753.503	453.3	166	41	4	5	1.72	Positive
LyPG	LyPG_C26:0	767.519	467.3	166	41	4	4.36	2.05	Positive
LyPG	LyPG_C27:0	781.535	481.3	166	41	4	5	2.03	Positive
LyPG	LyPG_C28:0	795.55	495.4	166	41	4	5	2.21	Positive
LyPG	LyPG_C29:0	809.566	509.4	166	41	4	5.43	1.09	Positive
LyPG	LyPG_C30:0	823.582	523.4	166	41	4	5.88	1.33	Positive
LyPG	LyPG_C31:0	837.597	537.4	166	41	4	6.36	1.27	Positive
LyPG	LyPG_C32:0	851.613	551.4	166	41	4	6.81	2.12	Positive
LyPG	LyPG_C33:0	865.629	565.4	166	41	4	7	2.3	Positive
LyPG	LyPG_C34:0	879.644	579.4	166	41	4	7.2	2.32	Positive
LyPG	LyPG_C35:0	893.66	593.5	166	41	4	7.2	2.34	Positive
LyPG	LyPG_C36:0	907.676	607.5	166	41	4	7.3	2.37	Positive
LyPG	LyPG_C37:0	921.691	621.5	166	41	4	8	2.16	Positive
LyPG	LyPG_C38:0	935.707	635.5	166	41	4	9	2.41	Positive
PA	PA_C24:0	554.38	439.38	166	13	4	4.47	3	Positive
PA	PA_C25:0	568.4	453.4	166	13	4	3.02	3	Positive
PA	PA_C26:0	582.41	467.41	166	13	4	4.71	3	Positive
PA	PA_C27:0	596.43	481.43	166	13	4	5	3	Positive
PA	PA_C28:0	610.45	495.45	166	13	4	5.84	3	Positive
PA	PA_C29:0	624.46	509.46	166	13	4	6.81	3	Positive
PA	PA_C30:0	638.48	523.48	166	13	4	7.07	3	Positive
PA	PA_C31:0	652.49	537.49	166	13	4	7.82	3	Positive
PA	PA_C32:0	666.51	551.51	166	13	4	8.39	3	Positive
PA	PA_C33:0	680.52	565.52	166	13	4	9.88	3	Positive

PA	PA_C34:0	694.54	579.54	166	13	4	10	3	Positive
PA	PA_C35:0	708.55	593.55	166	13	4	11	3	Positive
PA	PA_C36:0	722.57	607.57	166	13	4	11	3	Positive
PA	PA_C37:0	736.59	621.59	166	13	4	11.5	3	Positive
PA	PA_C38:0	750.6	635.6	166	13	4	11.5	3	Positive
PE	PE_C24:0	580.4	439.4	166	19	4	4.1	1.09	Positive
PE	PE_C25:0	594.41	453.41	166	19	4	4.4	0.88	Positive
PE	PE_C26:0	608.43	467.43	166	19	4	4.6	1.6	Positive
PE	PE_C27:0	622.45	481.45	166	19	4	4.97	1.29	Positive
PE	PE_C28:0	636.46	495.46	166	19	4	5.33	2.2	Positive
PE	PE_C29:0	650.48	509.48	166	19	4	5.78	2.45	Positive
PE	PE_C30:0	664.49	523.49	166	19	4	6.2	2.29	Positive
PE	PE_C31:0	678.51	537.51	166	19	4	6.71	3.25	Positive
PE	PE_C32:0	692.52	551.52	166	19	4	7.22	2.49	Positive
PE	PE_C33:0	706.54	565.54	166	19	4	7.98	2.09	Positive
PE	PE_C34:0	720.55	579.55	166	19	4	8.41	2.34	Positive
PE	PE_C35:0	734.57	593.57	166	19	4	7.09	2.24	Positive
PE	PE_C36:0	748.59	607.59	166	19	4	9.65	1.93	Positive
PE	PE_C37:0	762.6	621.6	166	19	4	10.06	2.01	Positive
PE	PE_C38:0	776.62	635.62	166	19	4	10.18	2.04	Positive
PG	PG_C24:0	628.42	439.42	166	17	4	10.16	2.03	Positive
PG	PG_C25:0	642.43	453.43	166	17	4	3.7	1.15	Positive
PG	PG_C26:0	656.45	467.45	166	17	4	3.86	1.2	Positive
PG	PG_C27:0	670.47	481.47	166	17	4	4.09	1.16	Positive
PG	PG_C28:0	684.48	495.48	166	17	4	4.32	1.58	Positive
PG	PG_C29:0	698.5	509.5	166	17	4	4.61	1.87	Positive
PG	PG_C30:0	712.51	523.51	166	17	4	4.89	1.83	Positive
PG	PG_C31:0	726.53	537.53	166	17	4	5.24	2.23	Positive
PG	PG_C32:0	740.54	551.54	166	17	4	5.59	2.03	Positive
PG	PG_C33:0	754.56	565.56	166	17	4	6.15	1.66	Positive
PG	PG_C34:0	768.58	579.58	166	17	4	6.48	2.3	Positive
PG	PG_C35:0	782.59	593.59	166	17	4	5.51	1.5	Positive
PG	PG_C36:0	796.61	607.61	166	17	4	7.49	1.65	Positive
PG	PG_C37:0	810.62	621.62	166	17	4	8.16	1.63	Positive
PG	PG_C38:0	824.64	635.64	166	17	4	8.69	1.74	Positive
Std	LyPG_C36:2	903.644	603.4	166	41	4	7.5	2.29	Positive
Std	15:0-18:1(d7) DAG	605.55	299.5	166	25	4	7.47	1.5	Positive
Std	15:0-18:1(d7) PA (Na Salt)	685.6	570.6	166	13	5	10.5	3	Positive
Std	15:0-18:1(d7) PE	711.6	570.5	166	19	5	7.15	1.8	Positive
Std	15:0-18:1(d7) PG (Na Salt)	759.6	570.6	166	17	4	4.63	1.65	Positive
Std	18:1(d7) Lyso PE	487.3	346.2	166	17	5	2.98	2	Positive
Std	CL 72:4 d5 (IS)	1472.2	599.5	166	45	5	13.8	1.2	Positive
Std	FA(13:0)	213.2	213.2	166	0	4	3.1	4	Negative

The only reference to be considered herein is: Huynh, K., Barlow, C.K., Jayawardana, K.S., Weir, J.M., Mellett, N.A., Cinel, M., Magliano, D.J., Shaw, J.E., Drew, B.G., and Meikle, P.J. (2019). High-Throughput Plasma Lipidomics: Detailed Mapping of the Associations with Cardiometabolic Risk Factors. Cell Chem. Biol. 26, 71-84.e4. (https://doi.org/10.1016/j.chembiol.2018.10.008).

3. On Fig S4: b is CL and c is PG. This is not consistent with Fig S4's figure legend (line 742).

[Our response] We thank the reviewer for raising this error. The legend has been modified accordingly as follows: ***Figure S4. Lipidomic analysis reveals enrichment of cardiolipin and phosphatidylglycerol in the purified YjlC-Ndh complex****. a. Mass spectrometry-based lipidomic analyses of the purified YjlC-Ndh complex (blue bar) and two negative controls unable to specifically bind lipids (catalase 1, red bars, and catalase 2, green bars) (see Methods). MSMS analyses reveal the molecular profiles of the CL (b), and PG (c) species associated with the complex.*

4. On Fig S4b and c: prefer to delete "Fatty acids" because the name of CL or PG including their fatty acids have been clearly shown.

[Our response] The figure has now been modified as recommended by the reviewer.

Reviewer #6 (Remarks to the Author):

In their manuscript “A Bacillales-specific tubular scaffold essential for NADH dehydrogenase activity” Seduk et al. convincingly show the co-dependence between Ndh and YjlC at a genomic, structural, and functional level using detailed phylogenetic, mutagenic, structural, functional and mass spectrometric analyses. CryoEM data accompanied by MS lipidomic analyses and docking simulations support the authors’ argument for the role of the Ndh-YjlC scaffold to act as hydrophobic channels. The methods in the paper complemented each other very well to build a compelling story one the specialized architecture of NDH-II catalysis in Bacillales. This manuscript will be of broad interest to the fields of bacterial metabolism, bioenergetics, structural biology, oxidoreductase mechanism and evolution of metabolic complexes.

The authors satisfactorily answered the questions raised by the previous reviewers. The paper was well-written and easy to read.

Strengths:
The combination of bioinformatics, mutagenesis, enzymology, lipidomics and cryoEM, combine to provide significant insight into the mechanism of NADH:quinone oxidoreduction in Bacillales. This manuscript significantly adds to our understanding of evolutionary strategies to maximize metabolic flux in bacteria, providing an additional example of tubular arrays of oxidoreductases anchored to the membrane.

Weaknesses:
No significant weaknesses identified.

[Our response] We thank the reviewer for the very positive assessment of our work

One minor points to be addressed
What were the models used in the initial C1 heterogeneous refinement?

[Our response] The Cryo-EM reconstruction procedure for the YjlC-Ndh complex involved two steps of particles curation through hetero-refinement job with C1 symmetry. As mentioned in the Material and Method session, both steps used *ab initio* models (three and five models for the first and second hetero refinement jobs, respectively), which were calculated from the best particles selected for the previous step (2D classification and first heterogenous refinement for the first and second hetero refinement jobs, respectively).

Very minor typographic comments for later consideration:

1. Inconsistent use of periods vs commas to signify a decimal point throughout the manuscript. Although this is a stylistic choice, please stick to a format to avoid confusion (lines 150 kcat/KM=19,3) (Fig 8a y-axis), and particularly the extinction coefficient in methods: DCPIP (21.000 M-1cm-1) and NADH (6.220 M-1cm-1) vs FAD (11,300 M-1cm-1)

[Our response] Done

2. Line 424: n-terminal should be N-terminal

[Our response] Done

3. Line 326: (first sentence in section Methods/strain construction): missing a parenthesis. (trpC2 …)

[Our response] Done

4. Figure 8b, top panel. For consistency, “Hmp” should be “HMP” on the right-hand y-axis

[Our response] Done

5. Lines 228-231, Greek letters are corrupted in our version of the manuscript and appear as squares.

[Our response] Done

6. Lines 394, under Methods/SEC-MALS analysis: consider putting the concentrations first before the chemical name for consistency (Tris 50 mM to 50 mM Tris).

[Our response] Done

Reviewer #7 (Remarks to the Author):

I co-reviewed this manuscript with one of the reviewers who provided the listed reports. This is part of the Nature Communications initiative to facilitate training in peer review and to provide appropriate recognition for Early Career Researchers who co-review manuscripts.

[Our response] We thank the reviewer for the very positive assessment of our work
