# Supplementary material for: A Bacillales-specific tubular scaffold essential for NADH dehydrogenase activity
Source: Nat Commun. 2026 Jun 18;17:7717. doi: 10.1038/s41467-026-74385-2 (PMC13433803; doi:10.1038/s41467-026-74385-2)

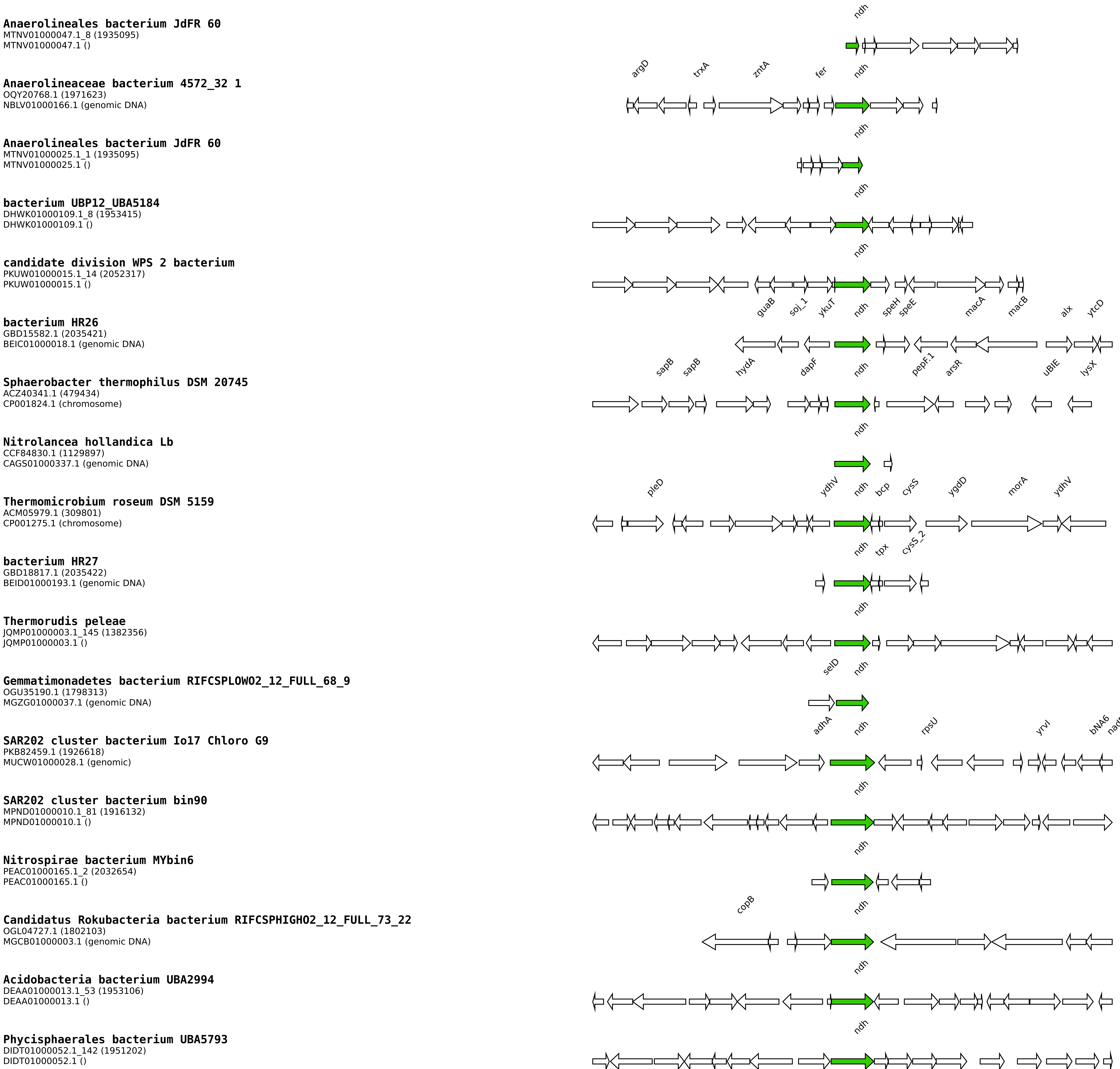
Anaerolineales bacterium JdFR 60
MTNV01000047.1_8 (1935095)
MTNV01000047.1 ()
ndh
Anaerolineaceae bacterium 4572_32 1
OQY20768.1 (1971623)
NBLV01000166.1 (genomic DNA)
argD
trxA
zntA
fer
ndh
Anaerolineales bacterium JdFR 60
MTNV01000025.1_1 (1935095)
MTNV01000025.1 ()
ndh
bacterium UBP12_UBA5184
DHWK01000109.1_8 (1953415)
DHWK01000109.1 ()
ndh
candidate division WPS 2 bacterium
PKUW01000015.1_14 (2052317)
PKUW01000015.1 ()
ndh
bacterium HR26
GBD15582.1 (2035421)
BEIC01000018.1 (genomic DNA)
guaB
soj_1
ykuT
ndh
speH
speE
macA
macB
alx
ytcD
Sphaerobacter thermophilus DSM 20745
ACZ40341.1 (479434)
CP001824.1 (chromosome)
sapB
sapB
hydA
dapF
ndh
pepF.1
arsR
uBIE
lysX
Nitrolancea hollandica Lb
CCF84830.1 (1129897)
CAGS01000337.1 (genomic DNA)
ndh
Thermomicrobium roseum DSM 5159
ACM05979.1 (309801)
CP001275.1 (chromosome)
pleD
ydhV
ndh
bcp
cysS
ygdD
morA
ydhV
bacterium HR27
GBD18817.1 (2035422)
BEID01000193.1 (genomic DNA)
ndh
tpx
cysS_2
Thermorudis peleae
JQMP01000003.1_145 (1382356)
JQMP01000003.1 ()
ndh
Gemmatimonadetes bacterium RIFCSPLOWO2_12_FULL_68_9
OGU35190.1 (1798313)
MGZG01000037.1 (genomic DNA)
selD
ndh
SAR202 cluster bacterium Io17 Chloro G9
PKB82459.1 (1926618)
MUCW01000028.1 (genomic)
adhA
ndh
rpsU
yrvI
bNA6
SAR202 cluster bacterium bin90
MPND01000010.1_81 (1916132)
MPND01000010.1 ()
ndh
Nitrospirae bacterium MYbin6
PEAC01000165.1_2 (2032654)
PEAC01000165.1 ()
ndh
Candidatus Rokubacteria bacterium RIFCSPHIGHO2_12_FULL_73_22
OGL04727.1 (1802103)
MGCB01000003.1 (genomic DNA)
copB
ndh
Acidobacteria bacterium UBA2994
DEAA01000013.1_53 (1953106)
DEAA01000013.1 ()
ndh
Phycisphaerales bacterium UBA5793
DIDT01000052.1_142 (1951202)
DIDT01000052.1 ()
ndh

Candidatus Lindowbacteria bacterium RIFCSPLOWO2_12_FULL_62_27
OGH61445.1 (1817870)
MFPR01000006.1 (genomic DNA)

Ignavibacteria bacterium UBA6688
DKJQ01000168.1_1 (1948685)
DKJQ01000168.1 ()

Cyanobacteria bacterium UBA7694
DLGM01000289.1_1 (1947897)
DLGM01000289.1 ()

Chlamydiae bacterium CG10_big_fil_rev_8_21_14_0_10_35_9
PIS01452.1 (1973935)
PFAC01000012.1 (genomic DNA)

Simkania negevensis Z
CCB88552.1 (331113)
FR872582.1 (genomic DNA)

Candidatus Aerophobetes bacterium
PCI78064.1 (2030807)
NVUK01000009.1 (genomic DNA)

Verrucomicrobium sp GAS474
SDT95772.1 (1882831)
LT629781.1 (genomic DNA)

Candidatus Dadabacteria bacterium RIFCSPHIGHO2_12_FULL_53_21
OGE21430.1 (1797756)
MFCJ01000108.1 (genomic DNA)

Candidatus Dadabacteria bacterium UBA2774
DEIM01000073.1_3 (1953209)
DEIM01000073.1 ()

Candidatus Dadabacteria bacterium
QIHJ01000431.1_3 (2080303)
QIHJ01000431.1 ()

bacterium HR10
GBC81620.1 (2035405)
BEHM01000007.1 (genomic DNA)

Verrucomicrobia bacterium 13_1_20CM_3_54_17
OLE11074.1 (1805405)
MNJH01000100.1 (genomic DNA)

Acidobacteria bacterium 13_1_20CM_2_55_15
OLE88372.1 (1803424)
MNKP01000104.1 (genomic DNA)

Chloracidobacterium sp UBA7659
DLHV01000060.1_21 (1946322)
DLHV01000060.1 ()

Chloracidobacterium sp UBA7659
DLHV01000060.1_22 (1946322)
DLHV01000060.1 ()

Chloracidobacterium sp UBA2345
DDUF01000075.1_240 (1946316)
DDUF01000075.1 ()

Nitrospira sp UBA6909
DKBD01000054.1_3 (1946995)
DKBD01000054.1 ()

Thaumarchaeota archaeon 13_1_40CM_2_39_4
OLD40890.1 (1805387)
MNHP01000141.1 (genomic DNA)

Nitrososphaeraceae archaeon UBA273
DAOR01000048.1_3 (1915684)
DAOR01000048.1 ()

Candidatus Nitrosocosmicus oleophilus
ALI35518.1 (1353260)
CP012850.1 (chromosome)

Nitrosotalea sp Nd2
SHO47753.1 (1499975)
FRFC01000005.1 (genomic DNA)

Nitrosopumilaceae archaeon
QIDA01000099.1_6 (2202732)
QIDA01000099.1 ()

bacterium SPGG8
LXTE01000263.1_6 (1842518)
LXTE01000263.1 ()

Nitrosopumilus sp D6
RNJ76534.1 (2478554)
RHFD01000032.1 (genomic DNA)

Nitrosopumilus sp H13
RNJ77183.1 (2478556)
RHFB01000033.1 (genomic DNA)

Candidatus Nitrosopumilus piranensis
AJM91772.1 (1582439)
CP010868.1 (chromosome)

Thaumarchaeota archaeon
PBO84281.1 (2026795)
NVWC01000025.1 (genomic DNA)

Nitrosopumilales archaeon CG_4_9_14_0_2_um_filter_34_16
PJC50735.1 (2022696)
PFRJ01000049.1 (genomic DNA)

Thaumarchaeota archaeon casp thauma3
LFEY01000008.1_1 (1661713)
LFEY01000008.1 ()

Thaumarchaeota archaeon S15
RNJ73843.1 (2478559)
RHEY01000134.1 (genomic DNA)

Nitrosotalea sp SbT1

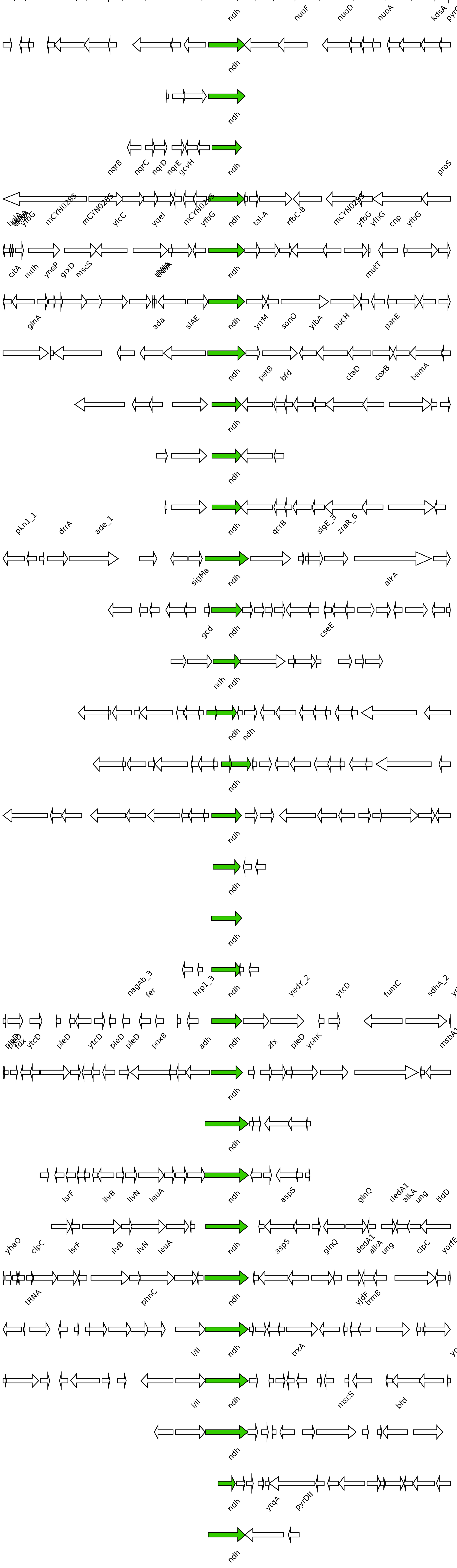

Nitrosotalea sp SbT1
FUYK01000013.1_64 (1903277)
FUYK01000013.1 ()

Candidatus Nitrosotalea devanaterra
CUR52159.1 (1078905)
LN890280.1 (genomic DNA)

Nitrosotalea sp CS
SMH70430.1 (1903276)
LT841358.1 (genomic DNA)

Nitrosotalea sp Nd2
SHO45711.1 (1499975)
FRFC01000003.1 (genomic DNA)

Candidatus Nitrosotenuis chungbukensis
AVSQ01000017.1_104 (1353246)
AVSQ01000017.1 ()

Thaumarchaeota archaeon BIN5
MRWN01000122.1_1 (1927150)
MRWN01000122.1 ()

Candidatus Nitrosotenuis aquarius
CP024808.1_490 (1846278)
CP024808.1 ()

Candidatus Nitrosotenuis cloacae
AJZ75320.1 (1603555)
CP011097.2 (chromosome)

Thaumarchaeota archaeon N4
CDI05638.1 (1407055)
CBTY010000008.1 (genomic DNA)

Thaumarchaeota archaeon CSP1 1
KRT60638.1 (1640512)
LDXL01000008.1 (genomic DNA)

Nitrosopumilaceae archaeon
QIDA01000008.1_17 (2202732)
QIDA01000008.1 ()

Candidatus Nitrosopumilus adriaticus
AJW70825.1 (1580092)
CP011070.1 (chromosome)

Nitrosopumilus sp PRT SC01
KPU81025.1 (1527301)
JPUE01000101.1 (genomic DNA)

Crenarchaeota archaeon
RDJ36706.1 (2056631)
QQVA01000004.1 (genomic DNA)

Candidatus Nitrosopumilus salaria BD31
EIJ66150.1 (859350)
AEXL02000083.1 (genomic DNA)

Candidatus Nitrosoarchaeum limnia SFB1
EGG41768.1 (886738)
CM001158.1 (chromosome)

Candidatus Nitrosoarchaeum limnia SFB1
EGG42651.1 (886738)
CM001158.1 (chromosome)

Nitrosopumilus sp LS_AOA
LFLC01000049.1_2 (1671876)
LFLC01000049.1 ()

Thaumarchaeota archaeon
PBO85509.1 (2026795)
NVWC01000005.1 (genomic DNA)

Candidatus Nitrosopumilus sp DDS1
KV575202.1_229 (1679446)
KV575202.1 ()

Candidatus Nitrosopumilus adriaticus
AJW70485.1 (1580092)
CP011070.1 (chromosome)

Candidatus Nitrosopumilus sp DDS1
KV575202.1_228 (1679446)
KV575202.1 ()

Candidatus Nitrosopumilus sp NM25
GBH33937.1 (718286)
BGKI01000004.1 (genomic DNA)

Euryarchaeota archaeum SCGC AAA287 N16
AXWK01000002.1_24 (1298894)
AXWK01000002.1 ()

Thaumarchaeota archaeon SCGC AC 337_F14
RAS38578.1 (1465461)
QLTN01000002.1 (genomic DNA)

Candidatus Nitrosopelagicus sp
MBO48715.1 (2026808)
PBPS01000026.1 (genomic DNA)

Candidatus Nitrosopelagicus brevis
AJA92222.1 (1410606)
CP007026.1 (chromosome)

Candidatus Nitrosotenuis chungbukensis
AVSQ01000009.1_97 (1353246)
AVSQ01000009.1 ()

Thaumarchaeota archaeon N4
CDI06700.1 (1407055)
CBTY010000011.1 (genomic DNA)

Candidatus Nitrosotenuis cloacae
AJZ75967.1 (1603555)
CP011097.2 (chromosome)

Candidatus Nitrosotenuis aquarius
CP024808.1_1531 (1846278)
CP024808.1 ()

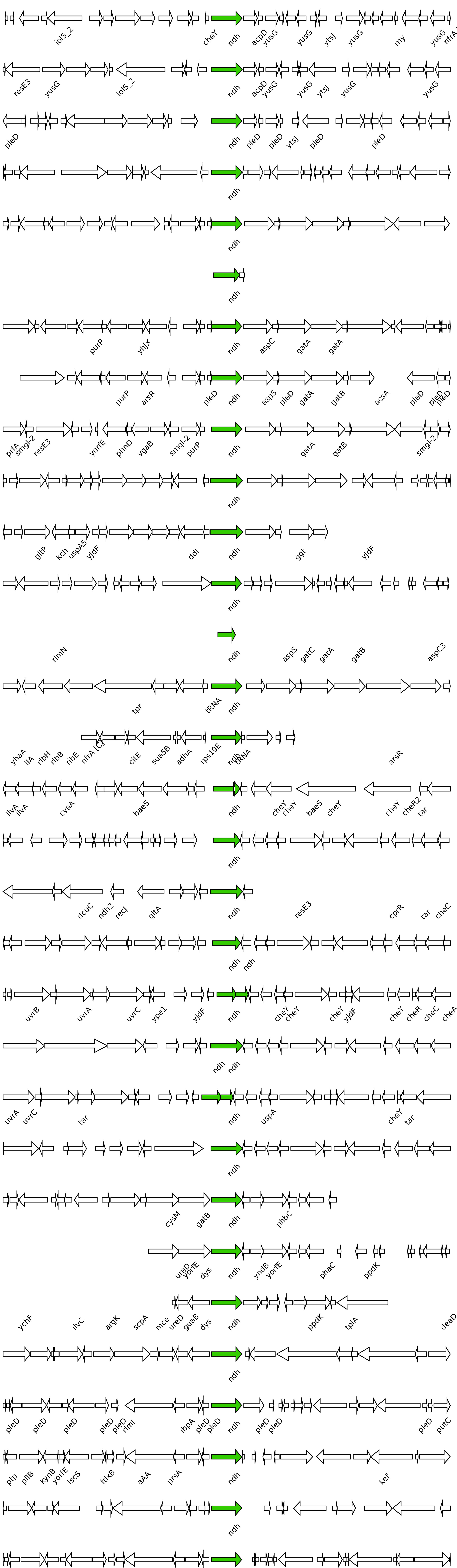

Nitrosopumilales archaeon UBA218
DAMC01000033.1_5 (1915705)
DAMC01000033.1 ()

Nitrosopumilales archaeon CG15_BIG_FIL_POST_REV_8_21_14_020_37_12
PIW31698.1 (2022694)
PFFZ01000102.1 (genomic DNA)

Candidatus Nitrosopumilus piranensis
AJM91485.1 (1582439)
CP010868.1 (chromosome)

Nitrosopumilus maritimus SCM1
ABX11908.1 (436308)
CP000866.1 (chromosome)

Marine Group I thaumarchaeote SCGC AAA799 E16
KER05954.1 (1502292)
JNVL01000021.1 (genomic DNA)

Candidatus Nitrosopumilus sp NM25
GBH34993.1 (718286)
BGKI01000010.1 (genomic DNA)

Candidatus Nitrosopumilus adriaticus
AJW69760.1 (1580092)
CP011070.1 (chromosome)

Candidatus Nitrosopumilus sp DDS1
KV575206.1_218 (1679446)
KV575206.1 ()

Crenarchaeota archaeon
RDJ38602.1 (2056631)
QQVA01000002.1 (genomic DNA)

Candidatus Nitrosoarchaeum sp
PHY08779.1 (2026886)
NSIR01000005.1 (genomic DNA)

Candidatus Nitrosoarchaeum limnia SFB1
EGG41530.1 (886738)
CM001158.1 (chromosome)

Nitrosarchaeum koreense MY1
EGP94656.1 (1001994)
AFPU01000001.1 (genomic)

Nitrosopumilales archaeon CG15_BIG_FIL_POST_REV_8_21_14_020_33_23
PIW34359.1 (2022693)
PFGA01000553.1 (genomic DNA)

Nitrosopumilaceae archaeon
QIDA01000179.1_3 (2202732)
QIDA01000179.1 ()

Thaumarchaeota archaeon casp thauma3
LFEY01000007.1_8 (1661713)
LFEY01000007.1 ()

Nitrosopumilus sp PRT SC01
KPU81070.1 (1527301)
JPUE01000085.1 (genomic DNA)

Thaumarchaeota archaeon SCGC AAA282 K18
AXWL01000003.1_15 (1298885)
AXWL01000003.1 ()

Candidatus Nitrosopumilus salaria BD31
EIJ66802.1 (859350)
AEXL02000023.1 (genomic DNA)

Nitrosopumilales archaeon CG_4_9_14_0_2_um_filter_34_16
PJC51239.1 (2022696)
PFRJ01000009.1 (genomic DNA)

Candidatus Nitrosopumilus sediminis
AFS83723.1 (1229909)
CP003843.1 (chromosome)

Nitrosopumilus sp BACL13 MAG 121220 bin23
KRO26740.1 (1655561)
LIAL01000129.1 (genomic DNA)

uncultured marine crenarchaeote Gulf of Maine
JH165224.1_57 (1089683)
JH165224.1 ()

uncultured marine crenarchaeote Gulf of Maine
JH165510.1_152 (1089683)
JH165510.1 ()

uncultured marine crenarchaeote Gulf of Maine
JH165510.1_153 (1089683)
JH165510.1 ()

uncultured marine crenarchaeote Gulf of Maine
AGBE01001169.1_1 (1089683)
AGBE01001169.1 ()

uncultured marine crenarchaeote Gulf of Maine
JH165482.1_8 (1089683)
JH165482.1 ()

uncultured marine crenarchaeote Gulf of Maine
AGBE01001784.1_2 (1089683)
AGBE01001784.1 ()

uncultured marine crenarchaeote Gulf of Maine
AGBE01000338.1_1 (1089683)
AGBE01000338.1 ()

Candidatus Nitrosomarinus sp
RMW33225.1 (2478979)
REGF01000093.1 (genomic DNA)

Nitrosopumilus sp Nsub
LQMW01000008.1_44 (1776294)
LQMW01000008.1 ()

uncultured marine crenarchaeote Gulf of Maine

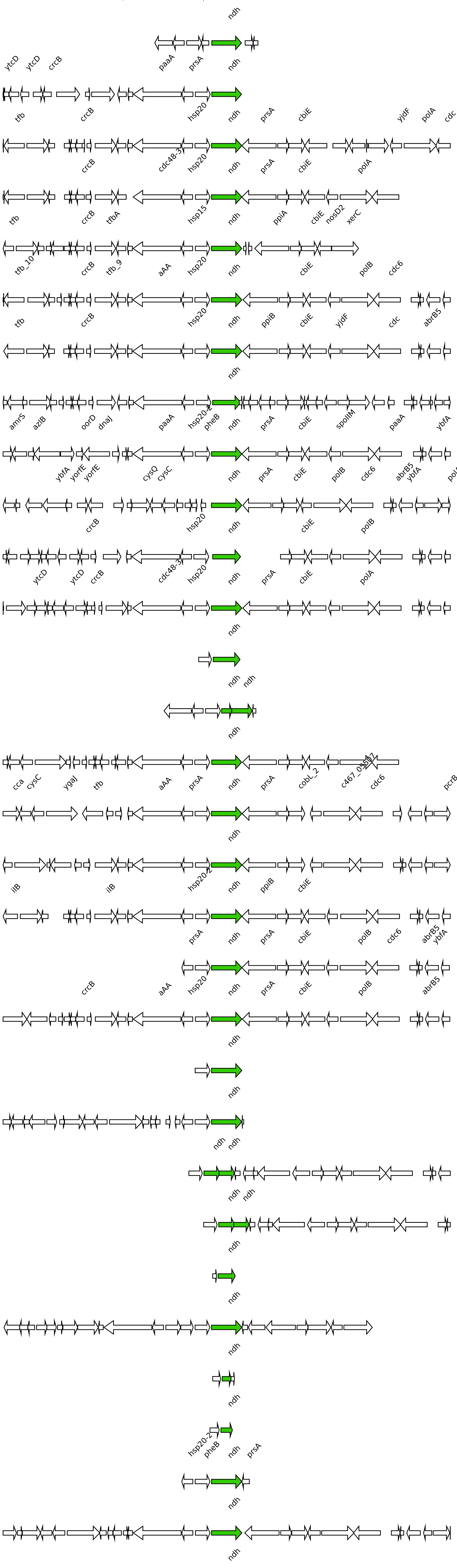

uncultured marine crenarchaeote Gulf of Maine
AGBE01001004.1_1 (1089683)
AGBE01001004.1 ()

Nitrosopumilus sp
MAI01498.1 (2024843)
NZEZ01000016.1 (genomic DNA)

Candidatus Nitrosomarinus catalina
ARS63642.1 (1898749)
CP021324.1 (chromosome)

Thaumarchaeota archaeon casp thauma1
LFEW01000026.1_62 (1661711)
LFEW01000026.1 ()

uncultured marine crenarchaeote Gulf of Maine
AGBE01000547.1_2 (1089683)
AGBE01000547.1 ()

Nitrosopumilus sp LS_AOA
LFLC01000036.1_10 (1671876)
LFLC01000036.1 ()

Thaumarchaeota archaeon
PBO85831.1 (2026795)
NVWC01000002.1 (genomic DNA)

Nitrosopumilus sp UBA526
DAXZ01000062.1_9 (1915597)
DAXZ01000062.1 ()

Nitrosopumilaceae archaeon
QIDA01000179.1_2 (2202732)
QIDA01000179.1 ()

Nitrosopumilus sp D6
RNJ76679.1 (2478554)
RHFD01000030.1 (genomic DNA)

Nitrosopumilus sp H13
RNJ75494.1 (2478556)
RHFB01000070.1 (genomic DNA)

Cenarchaeum symbiosum A
ABK78455.1 (414004)
DP000238.1 (chromosome)

Thaumarchaeota archaeon 13_1_20CM_2_39_11
OLE43890.1 (1805382)
MNJT01000094.1 (genomic DNA)

Thaumarchaeota archaeon 13_1_40CM_2_39_13_2
OLD33474.1 (1805386)
MNGY01000029.1 (genomic DNA)

Nitrosotalea sp Nd2
SHO43540.1 (1499975)
FRFC01000003.1 (genomic DNA)

Nitrosotalea sp SbT1
FUYK01000009.1_86 (1903277)
FUYK01000009.1 ()

Candidatus Nitrosotalea devanaterra
CUR52863.1 (1078905)
LN890280.1 (genomic DNA)

Nitrosotalea sp CS
SMH72061.1 (1903276)
LT841358.1 (genomic DNA)

candidate division NC10 bacterium SPGG6
LXTG01000015.1_11 (1842517)
LXTG01000015.1 ()

candidate division NC10 bacterium RIFCSPLOWO2_12_FULL_66_18
OGB98996.1 (1801659)
METI01000153.1 (genomic DNA)

candidate division NC10 bacterium RBG_16_65_8
OGB92998.1 (1801657)
METG01000156.1 (genomic DNA)

Verrucomicrobia bacterium
ATC66052.1 (2026799)
CP023344.1 (chromosome)

Opitutaceae bacterium UBA4046
DFZW01000248.1_12 (1952998)
DFZW01000248.1 ()

Opitutae bacterium Tous C4FEB
PAW89623.1 (1982321)
NEVA01000008.1 (genomic DNA)

Akkermansiaceae bacterium UBA4581
DGKB01000009.1_3 (1951362)
DGKB01000009.1 ()

Akkermansiaceae bacterium UBA2367
DDTJ01000022.1_20 (1951357)
DDTJ01000022.1 ()

Verrucomicrobiae bacterium DG1235
EDY83159.1 (382464)
DS990592.1 (genomic)

Verrucomicrobia bacterium UBA6056
DIYK01000077.1_2 (1948270)
DIYK01000077.1 ()

Verrucomicrobia bacterium UBA6053
DIYN01000031.1_129 (1948269)
DIYN01000031.1 ()

Verrucomicrobia bacterium UBA6056
DIYK01000077.1_3 (1948270)
DIYK01000077.1 ()

Nitrospirae bacterium SPGG5
LXTH01000017.1_4 (1842520)
LXTH01000017.1 ()

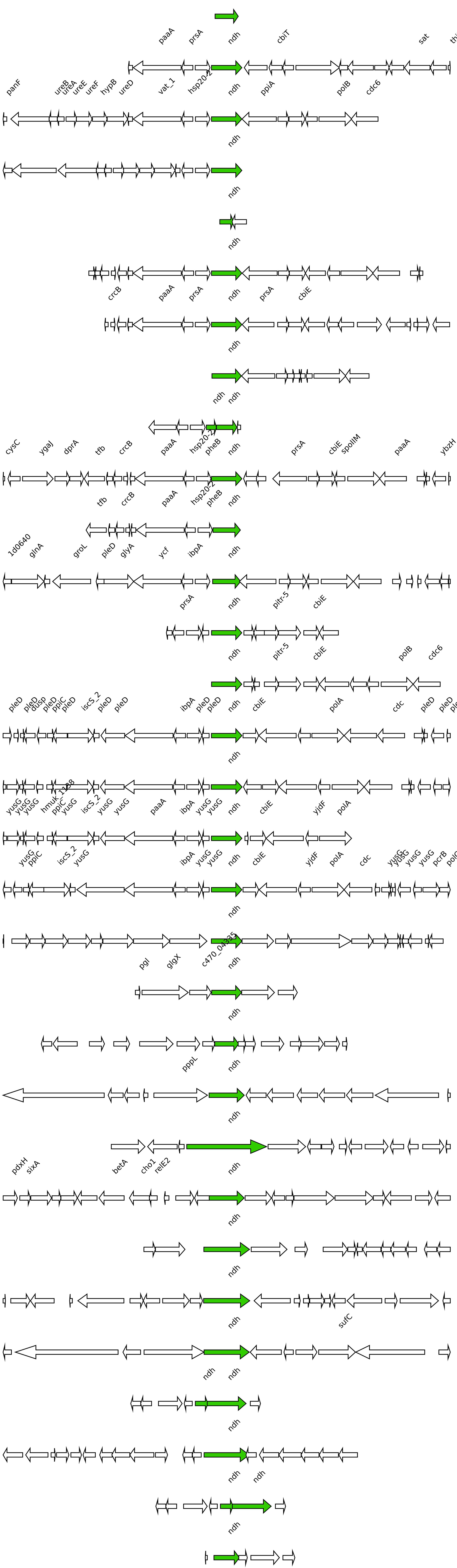

Candidatus Dadabacteria bacterium RIFCSPHIGHO2_12_FULL_53_21
OGE18956.1 (1797756)
MFCJ01000142.1 (genomic DNA)

Candidatus Dadabacteria bacterium UBA2774
DEIM01000177.1_1 (1953209)
DEIM01000177.1 ()

Gammaproteobacteria bacterium HGW Gammaproteobacteria 3
PKM11399.1 (2013799)
PGZD01000039.1 (genomic DNA)

Chromatocurvus halotolerans
QQSW01000001.1_321 (1132028)
QQSW01000001.1 ()

Alphaproteobacteria bacterium PA4
OYU14939.1 (2015572)
NKIQ01000015.1 (genomic DNA)

Betaproteobacteria bacterium GR16 43
APV48640.1 (1904640)
CP019169.1 (chromosome)

Methylibium petroleiphilum PM1
ABM93122.1 (420662)
CP000555.1 (chromosome)

Burkholderiales bacterium UBA4200
DFTY01000027.1_3 (1950777)
DFTY01000027.1 ()

Aquabacterium sp SJQ9
RRS04848.1 (2493092)
RSED01000005.1 (genomic DNA)

Thermobaculum terrenum ATCC BAA 798
ACZ43019.1 (525904)
CP001826.1 (chromosome)

Acidimicrobiia bacterium UBA6912
DKBA01000273.1_31 (1948321)
DKBA01000273.1 ()

Solirubrobacterales bacterium 67 14
OJU94063.1 (1895832)
MKST01000010.1 (genomic DNA)

Conexibacter sp Seoho 28
PTL56248.1 (2133960)
PYYB01000002.1 (genomic DNA)

Patulibacter americanus DSM 16676
ATUD01000004.1_34 (1122939)
ATUD01000004.1 ()

Patulibacter medicamentivorans
EHN08954.1 (1097667)
AGUD01000308.1 (genomic DNA)

Saccharomonospora azurea NA 128
EHY90536.1 (882081)
CM001466.1 (chromosome)

Saccharomonospora sp LRS4 154
OQO89891.1 (1962155)
MWIH01000008.1 (genomic DNA)

Saccharomonospora halophila 8
KB912479.1_8 (928729)
KB912479.1 ()

Saccharomonospora viridis DSM 43017
ACU95904.1 (471857)
CP001683.1 (chromosome)

Humibacter albus DSM 18994
ATXT01000011.1_385 (1121952)
ATXT01000011.1 ()

Rathayibacter rathayi
PPF14052.1 (33887)
PSUL01000015.1 (genomic DNA)

Microbacteriaceae bacterium 13S1 3
QANL01000001.1_544 (2144185)
QANL01000001.1 ()

Cryobacterium sp NEAU 85
RNE62465.1 (2486026)
RDSR01000010.1 (genomic DNA)

Frondihabitans sp 762G35
ARC56827.1 (1446794)
CP014619.1 (chromosome)

Frigoribacterium sp Leaf164
KQR47019.1 (1736282)
LMOX01000001.1 (genomic DNA)

Curtobacterium sp BH 2 1 1
AOX67329.1 (1905847)
CP017580.1 (chromosome)

Curtobacterium sp B18
BACZ01000243.1_4 (95614)
BACZ01000243.1 ()

Subtercola sp Z020
PPF81907.1 (2080582)
PSTT01000072.1 (genomic DNA)

Leifsonia sp ALI 44 B
ONI64058.1 (1933776)
MTQL01000006.1 (genomic)

Humibacter sp BT305
AXH34971.1 (2282656)
CP031192.1 (chromosome)

Amnibacterium sp DD4a

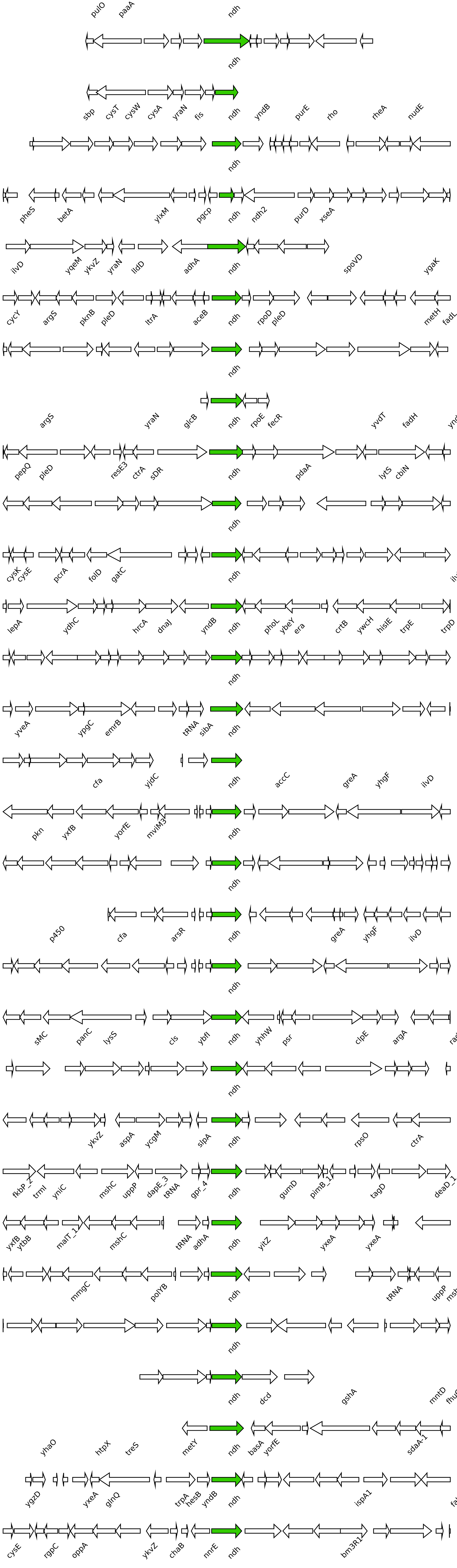

RIX27614.1 (2306585)
QXTG01000002.1 (genomic DNA)

Salinispora arenicola CNS 205
ABV96766.1 (391037)
CP000850.1 (chromosome)

Catenuloplanes indicus JCM 9534
BBHZ01000408.1_2 (1307450)
BBHZ01000408.1 ()

Geodermatophilus obscurus DSM 43160
ADB73688.1 (526225)
CP001867.1 (chromosome)

Acidothermus cellulolyticus 11B
ABK53674.1 (351607)
CP000481.1 (chromosome)

Frankia symbiont of Datisca glomerata
AEH08278.1 (656024)
CP002801.1 (chromosome)

Frankia casuarinae
ABD13268.1 (106370)
CP000249.1 (chromosome)

Thermomonospora curvata DSM 43183
ACY96627.1 (471852)
CP001738.1 (chromosome)

Motilibacter peucedani
RKS68630.1 (598650)
RBWV01000016.1 (genomic DNA)

Frankiales bacterium YIM 75000
RJK97928.1 (2339232)
QZEZ01000001.1 (genomic DNA)

Thermasporomyces composti
REF35680.1 (696763)
QTUC01000001.1 (genomic DNA)

Actinobacteria bacterium YIM 96077
AYY14390.1 (2487353)
CP033729.1 (chromosome)

Actinobacteria bacterium 69 20
OJV28832.1 (1895696)
MKSW01000011.1 (genomic DNA)

Frankia sp Iso899
ATXA01000005.1_30 (1283283)
ATXA01000005.1 ()

Pseudonocardiales bacterium
PZS31383.1 (2201155)
QHCB01000198.1 (genomic DNA)

Actinoalloteichus cyanogriseus DSM 43889
AUBJ01000017.1_170 (1120930)
AUBJ01000017.1 ()

Pseudonocardia tetrahydrofuranoxydans JCM 14745
BBFU01001640.1_2 (1303679)
BBFU01001640.1 ()

Actinoalloteichus cyanogriseus DSM 43889
AUBJ01000001.1_445 (1120930)
AUBJ01000001.1 ()

Saccharopolyspora rectivirgula
KEI45654.1 (28042)
JNVU01000012.1 (genomic DNA)

Halopolyspora algeriensis
RCW39997.1 (1500506)
QPJC01000013.1 (genomic DNA)

Actinopolyspora alba
SFD61355.1 (673379)
FOMZ01000001.1 (genomic DNA)

Thermocrispum agreste DSM 44070
KE386603.1_78 (1111738)
KE386603.1 ()

Haloechinothrix alba
SNR32474.1 (664784)
FZNW01000002.1 (genomic DNA)

Saccharomonospora viridis DSM 43017
ACU98190.1 (471857)
CP001683.1 (chromosome)

Saccharomonospora azurea NA 128
EHY88896.1 (882081)
CM001466.1 (chromosome)

Saccharomonospora sp LRS4 154
OQO92507.1 (1962155)
MWIH01000005.1 (genomic DNA)

Prauserella sp Am3
KID32002.1 (1515610)
JTJI01000002.1 (genomic DNA)

Saccharomonospora halophila 8
KB912490.1_15 (928729)
KB912490.1 ()

Thermobispora bispora DSM 43833
ADG88232.1 (469371)
CP001874.1 (chromosome)

Saccharopolyspora rectivirgula
KEI43354.1 (28042)
JNVU01000039.1 (genomic DNA)

Halopolyspora algeriensis
RCW42870.1 (1500506)
QPJC01000008.1 (genomic DNA)

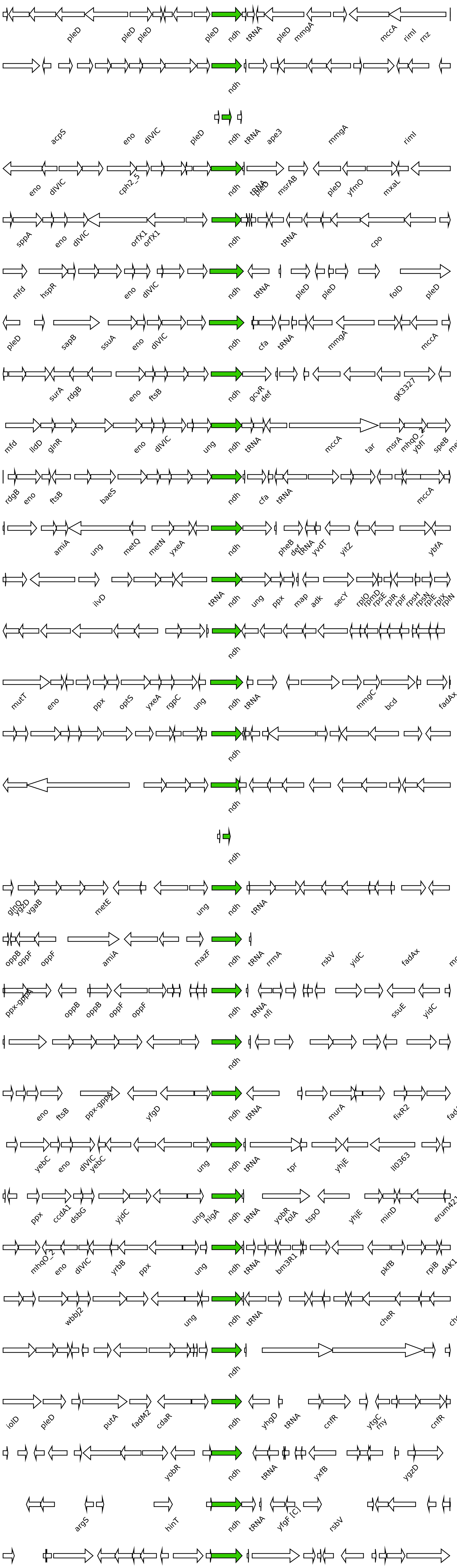

Pseudonocardia tetrahydrofuranoxydans JCM 14745
BBFU01001866.1_1 (1303679)
BBFU01001866.1 ()

Thermobispora bispora DSM 43833
ADG89821.1 (469371)
CP001874.1 (chromosome)

Thermomonospora curvata DSM 43183
ACY97257.1 (471852)
CP001738.1 (chromosome)

Streptomyces oceani
OEU96440.1 (1075402)
LJGU01000141.1 (genomic DNA)

Streptomyces somaliensis DSM 40738
AJJM01000042.1_24 (1134445)
AJJM01000042.1 ()

Streptomyces sp TP A0874
BBZK01000005.1_559 (549819)
BBZK01000005.1 ()

Streptomyces sp JCM 17819
BBFI01001698.1_1 (1302248)
BBFI01001698.1 ()

Streptomyces sp TAA040
AUKR01000024.1_3 (1288083)
AUKR01000024.1 ()

Streptomyces gilvigriseus
OIV38847.1 (1428644)
MLCF01000011.1 (genomic DNA)

Streptomyces sp TP A0874
BBZK01000010.1_170 (549819)
BBZK01000010.1 ()

Streptomyces somaliensis DSM 40738
AJJM01000080.1_6 (1134445)
AJJM01000080.1 ()

Streptomyces lavendulae subsp lavendulae JCM 4055
BBGA01000428.1_1 (1303691)
BBGA01000428.1 ()

Streptomyces sp TAA040
AUKR01000009.1_127 (1288083)
AUKR01000009.1 ()

Streptomyces sp JCM 17819
BBFI01000663.1_2 (1302248)
BBFI01000663.1 ()

Streptomyces oceani
OEV05367.1 (1075402)
LJGU01000100.1 (genomic DNA)

Haloechinothrix alba
SNR83474.1 (664784)
FZNW01000022.1 (genomic DNA)

Streptomyces gilvigriseus
OIV35366.1 (1428644)
MLCF01000161.1 (genomic DNA)

Thermobifida fusca YX
AAZ54470.1 (269800)
CP000088.1 (chromosome)

Marinactinospora thermotolerans DSM 45154
SKA33268.1 (1122192)
FUWS01000012.1 (genomic DNA)

Streptomonospora alba
KIH97873.1 (183763)
JROO01000031.1 (genomic DNA)

Nocardiopsaceae bacterium YIM 96095
RNL82827.1 (2487137)
RJMB01000020.1 (genomic DNA)

Nocardiopsis sp CNT312
AZXF01000031.1_36 (1137268)
AZXF01000031.1 ()

Nocardiopsis alkaliphila YIM 80379
ANBD01000120.1_20 (1246461)
ANBD01000120.1 ()

Nocardiopsis kunsanensis DSM 44524
ANAY01000018.1_85 (1246445)
ANAY01000018.1 ()

Streptomyces thermoautotrophicus
KWX05070.1 (1469144)
JYIJ01000013.1 (genomic DNA)

Demequina flava
BBRA01000001.1_549 (1095025)
BBRA01000001.1 ()

Demequina aestuarii
BBRD01000001.1_2073 (327095)
BBRD01000001.1 ()

Actinobacteria bacterium HGW Actinobacteria 8
PKQ18749.1 (2013653)
PHES01000021.1 (genomic DNA)

Demequina sp NBRC 110053
BBRJ01000002.1_1527 (1570342)
BBRJ01000002.1 ()

Demequina sp NBRC 110055
BBRL01000004.1_960 (1570344)
BBRL01000004.1 ()

Demequina aurantiaca

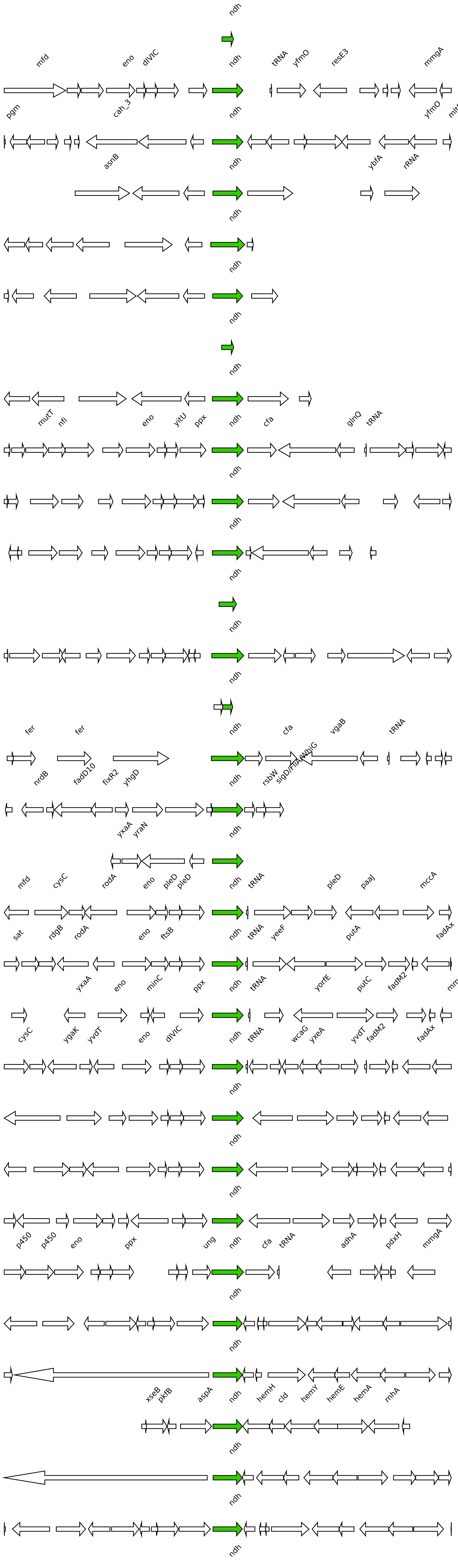

BBRF01000001.1_1168 (676200)
BBRF01000001.1 ()

Lysinimicrobium pelophilum
BBLY01000004.1_317 (1638984)
BBLY01000004.1 ()

Demequina sp NBRC 110052
BBRI01000003.1_260 (1570341)
BBRI01000003.1 ()

Lysinimicrobium aestuarii
BBLX01000002.1_215 (1638982)
BBLX01000002.1 ()

Demequina salsinemoris
BBQZ01000004.1_99 (577470)
BBQZ01000004.1 ()

Actinobacteria bacterium HGW Actinobacteria 4
PKQ25975.1 (2013649)
PHEW01000005.1 (genomic DNA)

Georgenia satyanarayanai
PYG00888.1 (860221)
QKLZ01000002.1 (genomic DNA)

Georgenia sp ZLJ0423
CP033325.1_514 (2483799)
CP033325.1 ()

Ruania albidiflava DSM 18029
ATWL01000020.1_55 (1123065)
ATWL01000020.1 ()

Haloactinobacterium album
SEE96208.1 (648782)
FNTX01000002.1 (genomic DNA)

Beutenbergia cavernae DSM 12333
ACQ79290.1 (471853)
CP001618.1 (chromosome)

Serinibacter salmoneus
PFG20449.1 (556530)
PDJD01000001.1 (genomic DNA)

Miniimonas sp PCH200
PWD51990.1 (2135789)
PYHR01000002.1 (genomic DNA)

Miniimonas sp S16
CP029049.1_946 (2171623)
CP029049.1 ()

Micrococcales bacterium 73 15
OJX98482.1 (1895792)
MKVO01000002.1 (genomic DNA)

Actinotalea fermentans ATCC 43279 JCM 9966 DSM 3133
KGM14899.1 (862422)
AXCX01000489.1 (genomic DNA)

Cellulomonas sp KRMCY2
JAGF01000001.1_341 (1304865)
JAGF01000001.1 ()

Cellulomonas bogoriensis 69B4 DSM 16987
KGM09899.1 (1386082)
AXCZ01000165.1 (genomic DNA)

Actinotalea ferrariae CF5 4
EYR62246.1 (948458)
AXCW01000286.1 (genomic DNA)

Sanguibacter marinus
SFF27535.1 (285351)
FONZ01000004.1 (genomic DNA)

Sanguibacter soli
PFG37522.1 (442709)
PDJH01000001.1 (genomic DNA)

Sanguibacter massiliensis
LT964979.1_2490 (1973217)
LT964979.1 ()

Cellulomonas sp UBA6250
DJVS01000060.1_5 (1946310)
DJVS01000060.1 ()

Cellulomonas timonensis
FCOT01000012.1_168 (1689271)
FCOT01000012.1 ()

Cellulomonas sp PSBB021
ASR56564.1 (2003551)
CP021430.1 (chromosome)

Cellulomonas terrae JCM 14899
BBGZ01000502.1_1 (1305858)
BBGZ01000502.1 ()

Cellulomonas xylanilytica JCM 14281
BBGX01000938.1_1 (1305856)
BBGX01000938.1 ()

Cellulomonas sp PhB150
ROS22997.1 (2485188)
RKHX01000005.1 (genomic DNA)

Cellulomonas fimi
VEH35363.1 (1708)
LR134387.1 (genomic DNA)

Cellulomonas marina
SFA95284.1 (988821)
FOKA01000004.1 (genomic DNA)

Cellulomonas flavigena DSM 20109
ADG73825.1 (446466)
CP001964.1 (chromosome)

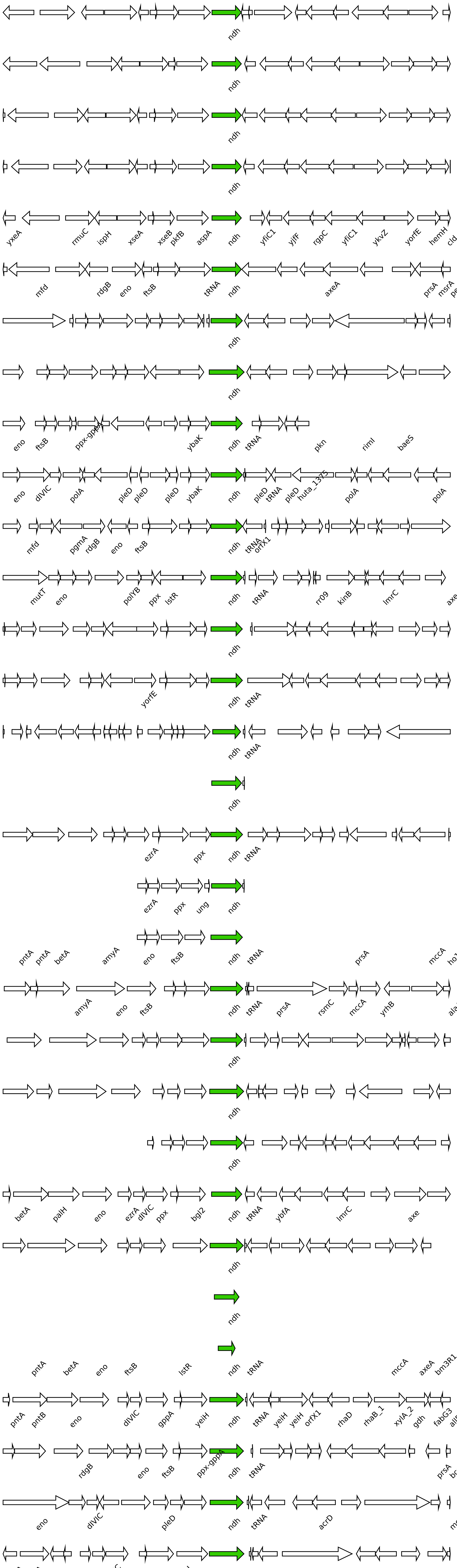

Cellulomonas sp 14 74 6
OZB50460.1 (1970333)
NCKG01000029.1 (genomic DNA)

Cellulomonas sp NEAU YY56
RMI06732.1 (2483352)
RFFI01000096.1 (genomic DNA)

Cellulomonas sp WB94
PVU83519.1 (2173174)
QEES01000002.1 (genomic)

Timonella senegalensis JC301
HE978644.1_284 (1095772)
HE978644.1 ()

Jonesia quinghaiensis DSM 15701
AUHN01000005.1_217 (1122130)
AUHN01000005.1 ()

Jonesia denitrificans DSM 20603
ACV09562.1 (471856)
CP001706.1 (chromosome)

Sanguibacter keddieii DSM 10542
ACZ20744.1 (446469)
CP001819.1 (chromosome)

Paraoerskovia marina
SDS23412.1 (545619)
LT629776.1 (genomic DNA)

Sediminihabitans luteus
PJJ69894.1 (1138585)
PGFE01000004.1 (genomic DNA)

Cellulomonas sp PhB143
ROS78656.1 (2485186)
RKIJ01000009.1 (genomic DNA)

Cellulosimicrobium cellulans
LWGL01000096.1_6 (1710)
LWGL01000096.1 ()

Xylanimonas cellulosilytica DSM 15894
ACZ31690.1 (446471)
CP001821.1 (chromosome)

Promicromonosporaceae bacterium CFH 30434
QXTH01000002.1_400 (2304675)
QXTH01000002.1 ()

Krasilnikoviella flava
SKC37086.1 (526729)
FUZQ01000001.1 (genomic DNA)

Isoptericola variabilis 225
AEG45203.1 (743718)
CP002810.1 (chromosome)

Isoptericola dokdonensis DS 3
ANC32744.1 (1300344)
CP014209.1 (chromosome)

Myceligenerans xiligouense
RPF19986.1 (253184)
RKQZ01000001.1 (genomic DNA)

Xylanimicrobium pachnodae JCM 13526 NBRC 107786
BBGW01000547.1_2 (1305855)
BBGW01000547.1 ()

Luteimicrobium subarcticum
PJI94139.1 (620910)
PGTZ01000007.1 (genomic DNA)

Promicromonosporaceae bacterium W15
DF158873.1_347 (1214064)
DF158873.1 ()

Bogoriella caseilytica
ROR72222.1 (56055)
RKHK01000001.1 (genomic DNA)

Georgenia sp SYP B2076
RXOZ01000031.1_240 (2495881)
RXOZ01000031.1 ()

Georgenia soli
PFG38805.1 (638953)
PDJI01000004.1 (genomic DNA)

Georgenia muralis
RPF26058.1 (154117)
RKRA01000001.1 (genomic DNA)

Micrococcales bacterium KH10
SNT12649.1 (1945885)
FZPE01000004.1 (genomic DNA)

Actinomyces radicidentis
AMD88280.1 (111015)
CP014228.1 (chromosome)

Actinomyces sp 553
PPPL01000094.1_11 (2072014)
PPPL01000094.1 ()

Actinomyces sp oral taxon 414
ALC98916.1 (712122)
CP012590.1 (chromosome)

Actinomyces urogenitalis DORA_12
ETJ06246.1 (1403939)
AZLV01000343.1 (genomic DNA)

Actinomyces howellii
VEG25491.1 (52771)
LR134350.1 (genomic DNA)

Actinomyces sp 2129
[illegible]

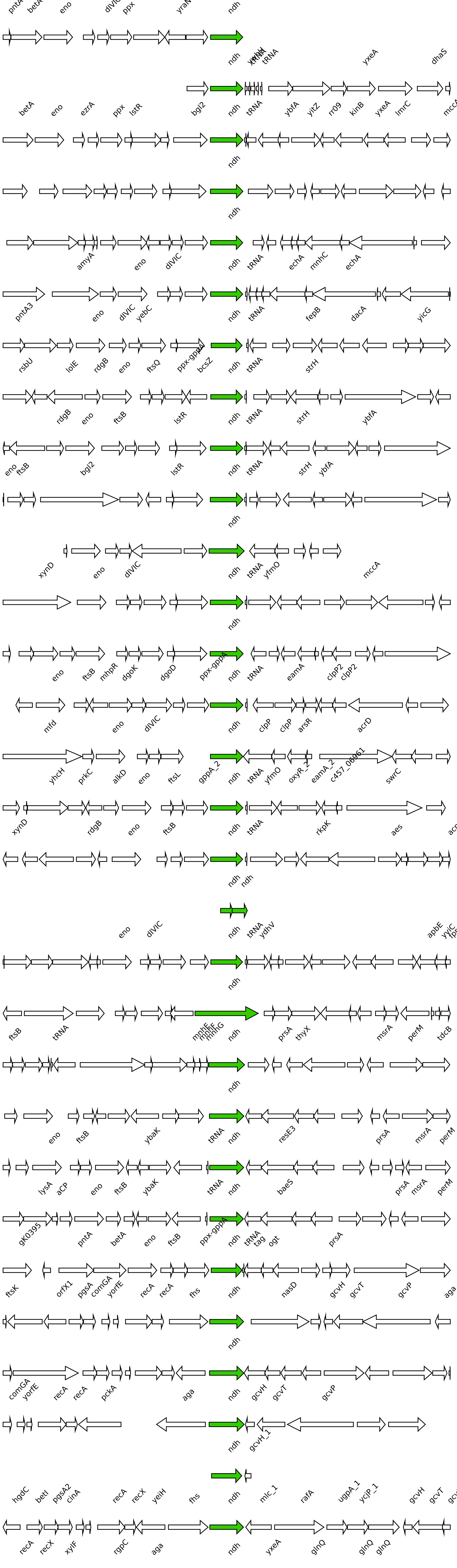

AYD90061.1 (2321394)
CP032514.1 (chromosome)

Actinomyces naeslundii
OLO87287.1 (1655)
MSKZ01000010.1 (genomic)

Actinomyces viscosus
VEI14500.1 (1656)
LR134477.1 (genomic DNA)

Actinomyces marimammalium
OKL50250.1 (156892)
MPDM01000002.1 (genomic DNA)

Actinomyces sp VUL4_3
AOZ73504.1 (1912795)
CP017812.1 (chromosome)

Actinomyces liubingyangii
OKL46526.1 (1921764)
MQSU01000004.1 (genomic DNA)

Kineococcus xinjiangensis
PPK97223.1 (512762)
PTJD01000004.1 (genomic DNA)

Quadrisphaera granulorum
PWJ56098.1 (317664)
QGDQ01000001.1 (genomic DNA)

Quadrisphaera sp DSM 44207
SDQ68973.1 (1881057)
FNKA01000003.1 (genomic DNA)

Haloglycomyces albus DSM 45210
AZUQ01000001.1_1776 (1089549)
AZUQ01000001.1 ()

Glycomyces arizonensis DSM 44726
AXWO01000026.1_24 (1121926)
AXWO01000026.1 ()

Glycomyces xiaoerkulensis
PGGV01000032.1_74 (2038139)
PGGV01000032.1 ()

Glycomyces sp YIM 121974
RRR97626.1 (2493553)
RSEB01000005.1 (genomic DNA)

Acidimicrobiales bacterium
PZS06639.1 (2201156)
QHCE01000090.1 (genomic DNA)

Actinobacteria bacterium YIM 96077
AYY15200.1 (2487353)
CP033729.1 (chromosome)

Xylanimicrobium pachnodae JCM 13526 NBRC 107786
BBGW01000547.1_1 (1305855)
BBGW01000547.1 ()

Devriesea agamarum
LN849456.1_158 (472569)
LN849456.1 ()

Dermabacter vaginalis
ANP27464.1 (1630135)
CP012117.1 (chromosome)

Brachybacterium nesterenkovii
SLM95391.1 (47847)
FWFG01000109.1 (genomic DNA)

Brachybacterium sp Marseille P4339
LT984895.1_1280 (2050896)
LT984895.1 ()

Brachybacterium endophyticum
PWH06978.1 (2182385)
QFKX01000002.1 (genomic DNA)

Brachybacterium muris UCD AY4
EYT48263.1 (1249481)
AORC01000016.1 (genomic DNA)

Brachybacterium squillarum M 6 3
AGBX01000006.1_268 (1074488)
AGBX01000006.1 ()

Brachybacterium sp P6 10 X1
APX31565.1 (1903186)
CP017297.1 (chromosome)

Brachybacterium faecium DSM 4810
ACU85189.1 (446465)
CP001643.1 (chromosome)

Brachybacterium zhongshanense JCM 15471
BBHA01000030.1_7 (1305859)
BBHA01000030.1 ()

Dermacoccus nishinomiyaensis
AIF39888.1 (1274)
CP008889.1 (chromosome)

Brevibacterium luteolum
PMB96992.1 (199591)
PNFZ01000011.1 (genomic DNA)

Brevibacterium ihuae
FXWZ01000002.1_997 (1631743)
FXWZ01000002.1 ()

Brevibacterium epidermidis
PDHL01000031.1_46 (1698)
PDHL01000031.1 ()

Brevibacterium paucivorans
PMD05876.1 (170994)
PNHK01000001.1 (genomic DNA)

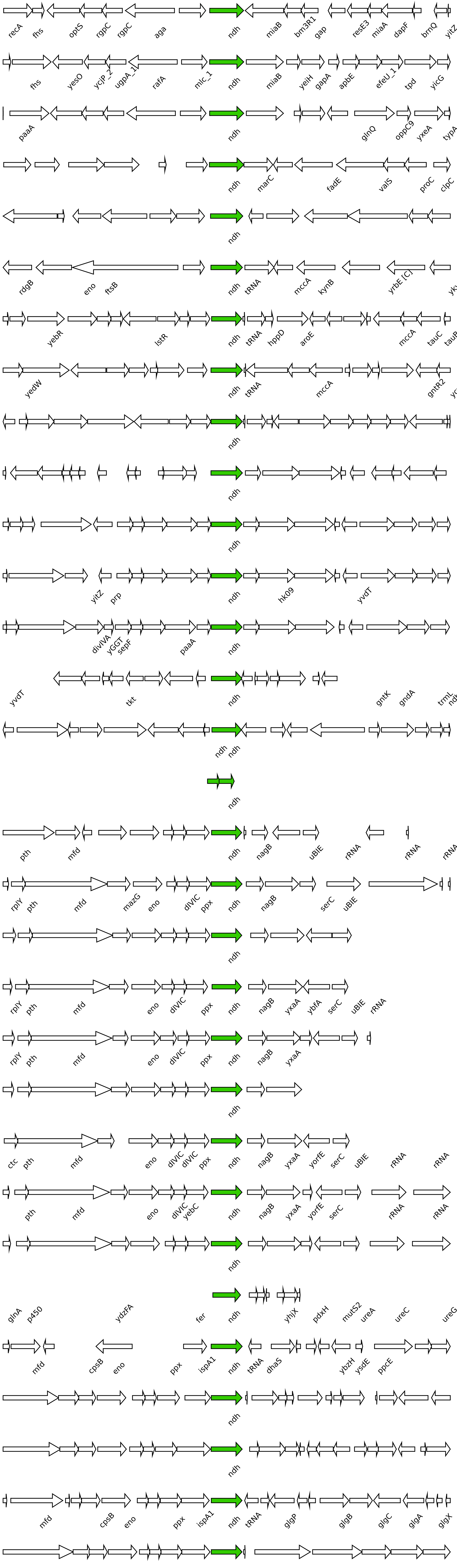

Brevibacterium album DSM 18261
AUFJ01000014.1_41 (1121122)
AUFJ01000014.1 ()

Brevibacterium senegalense
HE978604.1_39 (1033736)
HE978604.1 ()

Brevibacterium jeotgali
SMY12452.1 (1262550)
FXZM01000009.1 (genomic DNA)

Brevibacterium sp HMSC07C04
OFS26046.1 (1581130)
KV823266.1 (genomic)

Kocuria sp M5W7 7
ROZ61963.1 (2485125)
RKMF01000016.1 (genomic DNA)

Kocuria palustris
ALB02678.1 (71999)
CP012507.1 (chromosome)

Kocuria rosea
PWF80852.1 (1275)
QFBL01000008.1 (genomic DNA)

Kocuria rhizophila DC2201
BAG30124.1 (378753)
AP009152.1 (genomic DNA)

Kocuria atrinae C3 8
AJXN01000019.1_46 (1179225)
AJXN01000019.1 ()

Curtobacterium sp S6
KL537414.1_96 (1479623)
KL537414.1 ()

Kocuria kristinae
OAX52879.1 (37923)
LJBJ02000001.1 (genomic DNA)

Rothia sp ND6WE1A
LXTU01000071.1_18 (1848190)
LXTU01000071.1 ()

Rothia sp
HCN40694.1 (1885016)
DPZD01000016.1 (genomic DNA)

Micrococcaceae bacterium UBA7136
DKXC01000019.1_18 (1952261)
DKXC01000019.1 ()

Rothia dentocariosa ATCC 17931
ADP40830.1 (762948)
CP002280.1 (chromosome)

Rothia mucilaginosa DY 18
BAI65463.1 (680646)
AP011540.1 (genomic DNA)

Tersicoccus phoenicis
OMH23780.1 (554083)
MRDE01000068.1 (genomic DNA)

Arthrobacter saudimassiliensis
CEA07436.1 (1461584)
LN483070.1 (genomic DNA)

Arthrobacter pityocampae
PPB49654.1 (547334)
PRKW01000003.1 (genomic DNA)

Neomicrococcus aestuarii
APF40538.1 (556325)
CP018135.1 (chromosome)

Glutamicibacter sp UBA6655
DKKX01000129.1_5 (1946574)
DKKX01000129.1 ()

Glutamicibacter nicotianae
CP033081.1_772 (37929)
CP033081.1 ()

Arthrobacter sp MYb213
PRB68705.1 (1848595)
PCQA01000005.1 (genomic DNA)

Arthrobacter sp 7749
ASN40255.1 (2020130)
CP022462.1 (chromosome)

Glutamicibacter sp BW78
PCC24857.1 (2024403)
NRGU01000038.1 (genomic DNA)

Zhihengliuella halotolerans
PGGT01000079.1_24 (370736)
PGGT01000079.1 ()

Arthrobacter woluwensis
PSS45973.1 (156980)
PVZO01000001.1 (genomic DNA)

Arthrobacter sp FB24
ABK02546.1 (290399)
CP000454.1 (chromosome)

Sinomonas atrocyanea
AMM32080.1 (37927)
CP014518.1 (chromosome)

Arthrobacter sp 35W
KI519454.1_147 (1132441)
KI519454.1 ()

Arthrobacter alpinus
ALV44477.1 (656366)

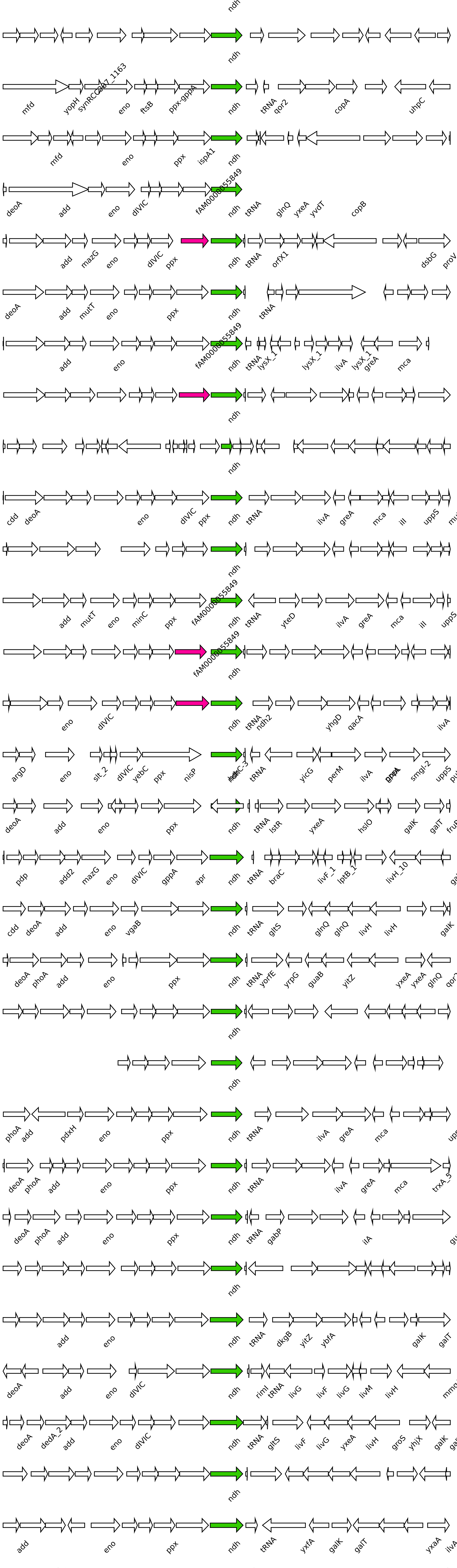

[illegible]
CP013745.1 (chromosome)

Arthrobacter sp ERGS1 01
ALE05088.1 (1704044)
CP012479.1 (chromosome)

Psychromicrobium lacuslunae
AJT40397.1 (1618207)
CP011005.1 (chromosome)

Renibacterium salmoninarum ATCC 33209
ABY22093.1 (288705)
CP000910.1 (chromosome)

Acaricomes phytoseiuli DSM 14247
AQXM01000056.1_3 (1120917)
AQXM01000056.1 ()

Arthrobacter crystallopoietes
AUI51713.1 (37928)
CP018863.1 (chromosome)

Haematomicrobium sanguinis DSM 21259
JIAG01000003.1_35 (1121020)
JIAG01000003.1 ()

Nesterenkonia sp F
AFRW01000057.1_29 (795955)
AFRW01000057.1 ()

Nesterenkonia sp JCM 19054
BAXI01000565.1_4 (1460646)
BAXI01000565.1 ()

Nesterenkonia sp PF2B19
OSM43018.1 (1881858)
MDSS02000034.1 (genomic DNA)

Nesterenkonia massiliensis
HG005170.1_77 (1232429)
HG005170.1 ()

Nesterenkonia sp M8
RJN32035.1 (2174932)
QYZP01000002.1 (genomic DNA)

Nesterenkonia sp RB2
QWLD01000003.1_359 (2282648)
QWLD01000003.1 ()

Nesterenkonia alba DSM 19423
ATXP01000010.1_39 (1122602)
ATXP01000010.1 ()

Nesterenkonia sandarakina
PRZ18128.1 (272918)
PVTY01000003.1 (genomic DNA)

Citricoccus muralis
REE05258.1 (169134)
QREH01000001.1 (genomic DNA)

Micrococcus terreus
SFV23063.1 (574650)
FPCG01000006.1 (genomic DNA)

Micrococcus luteus NCTC 2665
ACS30042.1 (465515)
CP001628.1 (chromosome)

Micrococcus lylae
PMC30729.1 (1273)
PNGL01000005.1 (genomic DNA)

Auritidibacter sp NML130574
AXR74003.1 (2170745)
CP031746.1 (chromosome)

Enteractinococcus helveticum
OAV60205.1 (1837282)
LXEY01000020.1 (genomic)

Yaniella halotolerans DSM 15476
ATVQ01000001.1_212 (1123507)
ATVQ01000001.1 ()

Micrococcaceae bacterium UBA3381
DEPX01000066.1_4 (1952256)
DEPX01000066.1 ()

Arthrobacter castelli DSM 16402
AUMN01000006.1_90 (1121019)
AUMN01000006.1 ()

Arthrobacter sp HMSC08H08
OFT24472.1 (1581143)
KV789371.1 (genomic)

Arthrobacter sp JZ R 183
RKW70601.1 (2283632)
QQXL01000003.1 (genomic DNA)

Krasilnikoviella flava
SKC38620.1 (526729)
FUZQ01000001.1 (genomic DNA)

Friedmanniella luteola
SDT40335.1 (546871)
LT629749.1 (genomic DNA)

Auraticoccus monumenti
SDE08951.1 (675864)
LT629688.1 (genomic DNA)

Cellulomonas terrae JCM 14899
BBGZ01001349.1_1 (1305858)
BBGZ01001349.1 ()

Curtobacterium sp BH 2 1 1
AOX66213.1 (1905847)
CP017580.1 (chromosome)

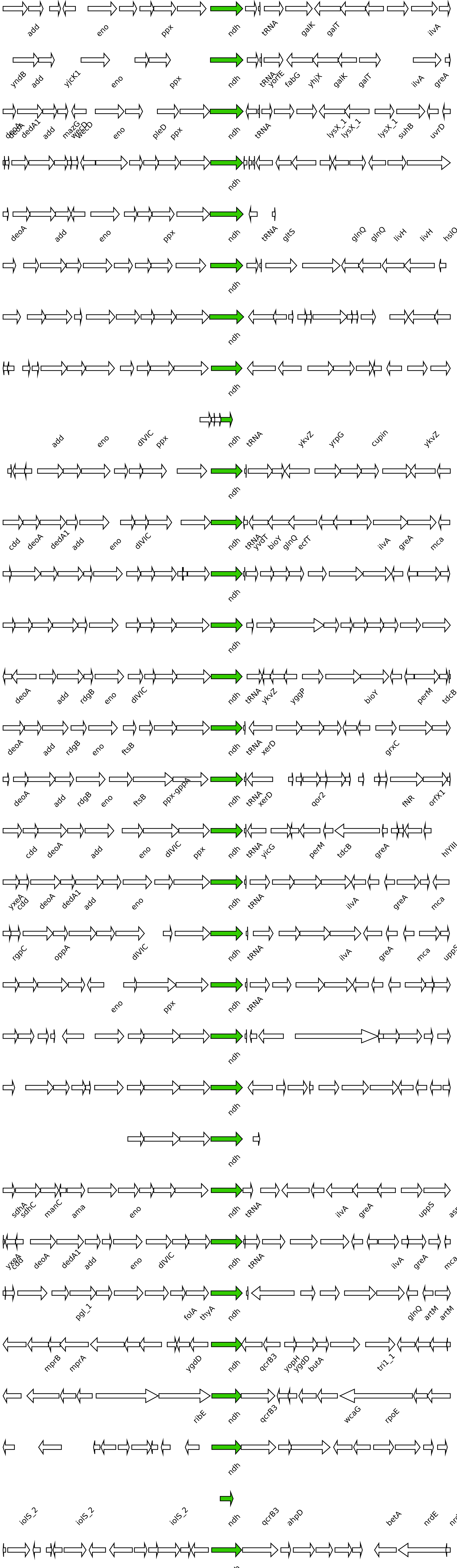

Curtobacterium sp B18
BACZ01000302.1_5 (95614)
BACZ01000302.1 ()

Diaminobutyricimonas aerilata
PJJ73373.1 (1162967)
PGFF01000001.1 (genomic DNA)

Amnibacterium sp DD4a
RIX28411.1 (2306585)
QXTG01000002.1 (genomic DNA)

Herbiconiux ginsengi
SDY81658.1 (381665)
FNPZ01000001.1 (genomic DNA)

Corynebacterium like bacterium B27
BADC01000145.1_5 (95617)
BADC01000145.1 ()

Cellulomonas sp NEAU YY56
RMI12506.1 (2483352)
RFFI01000036.1 (genomic DNA)

Promicromonosporaceae bacterium CFH 30434
QXTH01000005.1_294 (2304675)
QXTH01000005.1 ()

Beutenbergia cavernae DSM 12333
ACQ80390.1 (471853)
CP001618.1 (chromosome)

Propionibacteriaceae bacterium 16Sb5 5
RCK68029.1 (2268447)
QOUI01000015.1 (genomic DNA)

Nocardioides halotolerans DSM 19273
KE383922.1_114 (1122609)
KE383922.1 ()

Nocardioides terrae
SFC76421.1 (574651)
FOLB01000011.1 (genomic DNA)

Auraticoccus monumenti
SDE38678.1 (675864)
LT629688.1 (genomic DNA)

Demequina sp NBRC 110053
BBRJ01000003.1_304 (1570342)
BBRJ01000003.1 ()

Acidobacteriales bacterium 59 55
OJV42058.1 (1895690)
MKSV01000007.1 (genomic DNA)

Micrococcales bacterium UBA3913
DGEZ01000146.1_3 (1951170)
DGEZ01000146.1 ()

Pseudoclavibacter soli DSM 23366
AUIC01000003.1_555 (1123013)
AUIC01000003.1 ()

Pseudoclavibacter sp AY1F1
PPF46941.1 (2080583)
PSTQ01000001.1 (genomic DNA)

Zimmermannella sp YIM 102482 1
RRJ86412.1 (2488791)
RQVS01000009.1 (genomic DNA)

Zimmermannella faecalis ATCC 13722
KB907082.1_136 (1278308)
KB907082.1 ()

Gulosibacter sp 10
SJM66467.1 (1255570)
FUIC01000030.1 (genomic DNA)

Gulosibacter molinativorax DSM 13485
AUDX01000006.1_101 (1121934)
AUDX01000006.1 ()

Zimmermannella bifida NBRC 103089
BCSP01000005.1_12 (1349787)
BCSP01000005.1 ()

Zimmermannella alba NBRC 15616
BCSO01000001.1_35 (1349786)
BCSO01000001.1 ()

actinobacterium acMicro 1
KGA06512.1 (1504316)
JNSC01000032.1 (genomic)

Aurantimicrobium minutum
BAU99520.1 (708131)
AP017457.1 (genomic DNA)

Microbacteriaceae bacterium MWH Ta3
PTW90631.1 (207608)
QBKD01000001.1 (genomic DNA)

Micrococcales bacterium 32 70 13
OYX59434.1 (1970540)
NCEJ01000001.1 (genomic DNA)

Microcella alkaliphila
BAU31712.1 (279828)
AP017315.1 (genomic DNA)

Yonghaparkia sp Root332
KQV26388.1 (1736516)
LMCX01000001.1 (genomic DNA)

Lysinimonas sp 2DFWR 13
AYF98428.1 (2419774)
CP032630.1 (chromosome)

Micrococcales bacterium 72 143
OJX66360.1 (1895790)

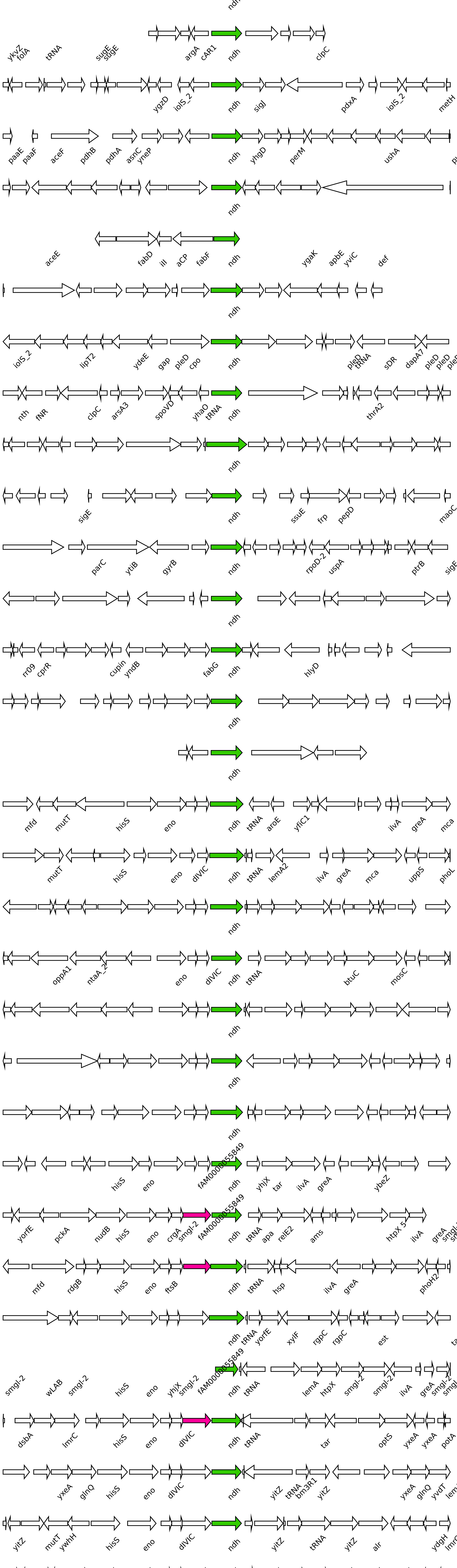

MKVM01000005.1 (genomic DNA)

marine actinobacterium PHSC20C1
EAR24498.1 (312284)
CH672415.1 (genomic)

Micrococcales bacterium 70 64
OJX84705.1 (1895789)
MKVP01000001.1 (genomic DNA)

Cryobacterium mesophilum
PJJX01000002.1_2073 (433647)
PJJX01000002.1 ()

Diaminobutyricimonas aerilata
PJJ71478.1 (1162967)
PGFF01000001.1 (genomic DNA)

Microbacteriaceae bacterium 13S1 3
QANL01000002.1_484 (2144185)
QANL01000002.1 ()

Agromyces sp 30A
AWB94879.1 (2080742)
CP028913.1 (chromosome)

Agromyces aureus
ANJ26495.1 (453304)
CP013979.1 (chromosome)

Plantibacter sp PA 3 X8
AZH83323.1 (2480625)
CP033107.1 (chromosome)

Leifsonia xyli subsp xyli str CTCB07
AAT89475.1 (281090)
AE016822.1 (chromosome)

Leifsonia sp Root227
KRC49610.1 (1736496)
LMIO01000003.1 (genomic DNA)

Compostimonas suwonensis
PJJ62170.1 (1048394)
PGFB01000003.1 (genomic DNA)

Leifsonia sp Root112D2
KQV07482.1 (1736426)
LMCU01000001.1 (genomic DNA)

Glaciibacter superstes DSM 21135
ATWH01000013.1_123 (1121924)
ATWH01000013.1 ()

Cryobacterium arcticum
ANP73307.1 (670052)
CP016282.1 (chromosome)

Leifsonia sp Leaf325
KQQ94440.1 (1736337)
LMOK01000002.1 (genomic DNA)

Mycetocola sp 449
AWB86969.1 (2079792)
CP026949.1 (chromosome)

Labedella endophytica
RUQ99182.1 (1523160)
RZGZ01000003.1 (genomic DNA)

Leifsonia sp ALI 44 B
ONI64030.1 (1933776)
MTQL01000006.1 (genomic)

Microbacterium humi
SEB35410.1 (640635)
FNRY01000001.1 (genomic DNA)

Microbacterium agarici
PFG31777.1 (630514)
PDJE01000001.1 (genomic DNA)

Cryobacterium flavum
SDN61799.1 (1424659)
FNIB01000006.1 (genomic DNA)

Agreia pratensis
SMG41358.1 (150121)
FXAY01000004.1 (genomic DNA)

Amnibacterium sp M8JJ 5
PVZ93698.1 (2173173)
QEOP01000002.1 (genomic DNA)

Clavibacter michiganensis subsp michiganensis NCPPB 382
CAN02331.1 (443906)
AM711867.1 (genomic DNA)

Subtercola sp Z020
PPF88275.1 (2080582)
PSTT01000022.1 (genomic DNA)

Humibacter sp BT305
AXH35596.1 (2282656)
CP031192.1 (chromosome)

Corynebacterium like bacterium B27
BADC01001101.1_10 (95617)
BADC01001101.1 ()

Herbiconiux ginsengi
SDZ35151.1 (381665)
FNPZ01000003.1 (genomic DNA)

Rathayibacter rathayi
PPF16008.1 (33887)
PSUL01000002.1 (genomic DNA)

Candidatus Lumbricidophila eiseniae
PDQ34905.1 (1969409)
NAEP01000044.1 (genomic DNA)

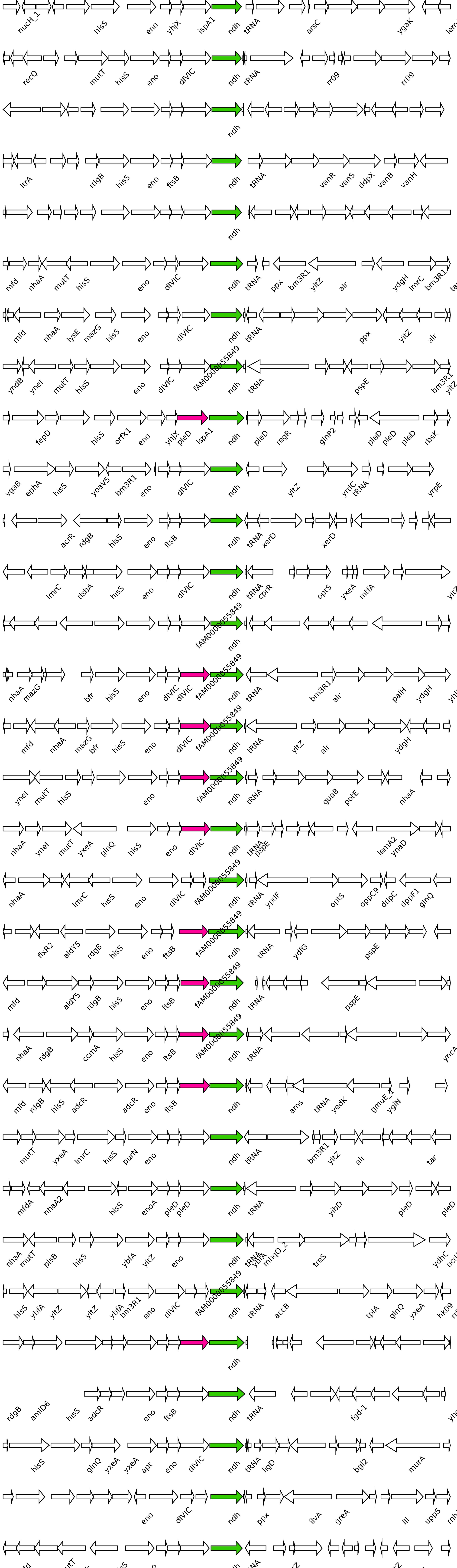

Salinibacterium sp CGMCC 1 16371
AWB89788.1 (2079791)
CP026951.1 (chromosome)

Mycetocola reblochoni REB411
SJN18916.1 (1255698)
FUKR01000008.1 (genomic DNA)

Actinomycetales bacterium SN12
PKI92168.1 (2058180)
PJCV01000002.1 (genomic DNA)

Microbacterium sp cl152
SEG27911.1 (1761808)
FNUQ01000004.1 (genomic DNA)

Microbacterium profundi
JPSY01000001.1_442 (450380)
JPSY01000001.1 ()

Microbacterium sp CGR1
AKV87810.1 (1696072)
CP012299.1 (chromosome)

Microbacterium kitamiense
PGGU01000006.1_145 (76884)
PGGU01000006.1 ()

Microbacterium ketosireducens
KJL39956.1 (92835)
JYIZ01000048.1 (genomic DNA)

Microbacterium pygmaeum
SDH03666.1 (370764)
LT629692.1 (genomic DNA)

Microbacterium sp UBA1612
DCMW01000004.1_119 (1946942)
DCMW01000004.1 ()

Microbacterium sp CF335
JUGQ01000003.1_102 (1500279)
JUGQ01000003.1 ()

Microbacterium sp No 7
ALJ19843.1 (1714373)
CP012697.1 (chromosome)

Microbacterium aurum
APZ34029.1 (36805)
CP018762.1 (chromosome)

Microbacterium sp UNCCL10
JQKT01000007.1_564 (1449058)
JQKT01000007.1 ()

Microbacterium sp LKL04
SCY29853.1 (912630)
LT627736.1 (genomic DNA)

Microbacterium lemovicicum
AZS37322.1 (1072463)
CP031423.1 (chromosome)

Microbacterium sp AG1240
RKT36465.1 (2183992)
RBXJ01000001.1 (genomic DNA)

Microbacterium sp 10M 3C3
CP034245.1_1290 (2483401)
CP034245.1 ()

Microbacterium luticocti DSM 19459
AULS01000001.1_444 (1122239)
AULS01000001.1 ()

Microbacterium testaceum StLB037
BAJ75964.1 (979556)
AP012052.1 (genomic DNA)

Microbacterium sp C448
CDK01466.1 (1177594)
HG779647.1 (genomic DNA)

Microbacterium sp ABRD_28
AZC14344.1 (2268461)
CP031015.1 (chromosome)

Microbacterium paludicola
APF33111.1 (300019)
CP018134.1 (chromosome)

Microbacterium mangrovi
KHK98478.1 (1348253)
JTDK01000006.1 (genomic DNA)

Microbacterium sp Leaf351
KQR90598.1 (1736348)
LMPK01000038.1 (genomic DNA)

Microbacterium ginsengiterrae
RKE16061.1 (546115)
RAPT01000001.1 (genomic DNA)

Microbacterium gorillae
LN648874.1_49 (1231063)
LN648874.1 ()

Microbacterium sp Root53
KQZ05031.1 (1736553)
LMFR01000012.1 (genomic DNA)

Microbacterium sp YZYP518
KZ672985.1_15 (2067812)
KZ672985.1 ()

Microbacterium halotolerans
KZ984051.1_1648 (246613)
KZ984051.1 ()

Microbacterium sediminis
OCG76584.1 (904291)

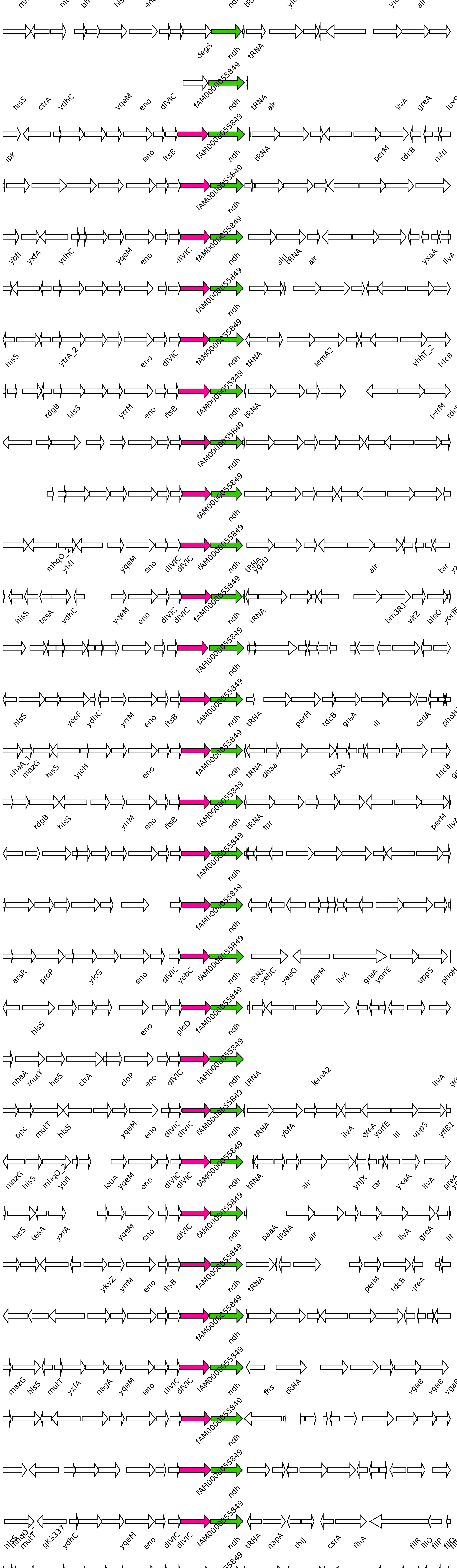

KV744790.1 (genomic)

Microbacterium sp YZGP509
PNEY01000001.1_519 (2067814)
PNEY01000001.1 ()

Microbacterium gubbeenense DSM 15944
AUGQ01000006.1_49 (1122237)
AUGQ01000006.1 ()

Microbacterium sorbitolivorans
RCK62003.1 (1867410)
KZ848473.1 (genomic)

Microbacterium indicum DSM 19969
AULR01000001.1_22 (1122238)
AULR01000001.1 ()

Curtobacterium sp B18
BACZ01000226.1_1 (95614)
BACZ01000226.1 ()

Curtobacterium sp BH 2 1 1
AOX65008.1 (1905847)
CP017580.1 (chromosome)

Humibacter albus DSM 18994
ATXT01000014.1_222 (1121952)
ATXT01000014.1 ()

Mycetocola lacteus
RLP80890.1 (76637)
RCUY01000011.1 (genomic DNA)

Mycetocola lacteus
RLP84675.1 (76637)
RCUY01000001.1 (genomic DNA)

actinobacterium acMicro 4
KGA05106.1 (1504317)
JNSD01000012.1 (genomic)

Pontimonas salivibrio
AVG23510.1 (1159327)
CP026923.1 (chromosome)

Cellulomonadaceae bacterium TMED98
OUV17542.1 (1986624)
NHEZ01000024.1 (genomic DNA)

Amnibacterium sp DD4a
RIX30855.1 (2306585)
QXTG01000001.1 (genomic DNA)

Candidatus Rhodoluna planktonica
AOY56692.1 (535712)
CP015208.1 (chromosome)

Rhodoluna lacicola
AIC47803.1 (529884)
CP007490.1 (chromosome)

Micrococcales bacterium UBA7469
DLPB01000012.1_12 (1951173)
DLPB01000012.1 ()

Micrococcales bacterium UBA2037
DDBH01000013.1_71 (1951164)
DDBH01000013.1 ()

Candidatus Aquiluna sp
HAE73821.1 (2053504)
DMBI01000030.1 (genomic DNA)

Candidatus Aquiluna sp UB MaderosW2red
SCX08803.1 (1855377)
LT627734.1 (genomic DNA)

Gryllotalpicola sp 2DFW10M 5
AYG02624.1 (2419771)
CP032624.1 (chromosome)

Leucobacter sp OH2974_COT 288
RRD35284.1 (2491050)
RQYU01000004.1 (genomic DNA)

Leucobacter sp OH1287
RRD60526.1 (2491049)
RQYM01000008.1 (genomic DNA)

Leucobacter chromiiresistens
KTR81763.1 (1079994)
LDRK01000117.1 (genomic DNA)

Leucobacter sp Ag1
KKI20900.1 (1642040)
LAYO01000088.1 (genomic DNA)

Leucobacter sp UCD THU
EYT51983.1 (1292023)
KB714605.1 (genomic)

Leucobacter salsicius M1 8
AOCN01000022.1_148 (1238186)
AOCN01000022.1 ()

Mycolicibacterium mucogenicum 261Sha1 1M5
RKQ94987.1 (1415782)
RBIK01000003.1 (genomic DNA)

Agrococcus casei LMG 22410
SJM66029.1 (1255656)
FUHU01000043.1 (genomic DNA)

Agrococcus carbonis
SDR80003.1 (684552)
LT629734.1 (genomic DNA)

Agrococcus lahaulensis DSM 17612
AULD01000006.1_490 (1120958)
AULD01000006.1 ()

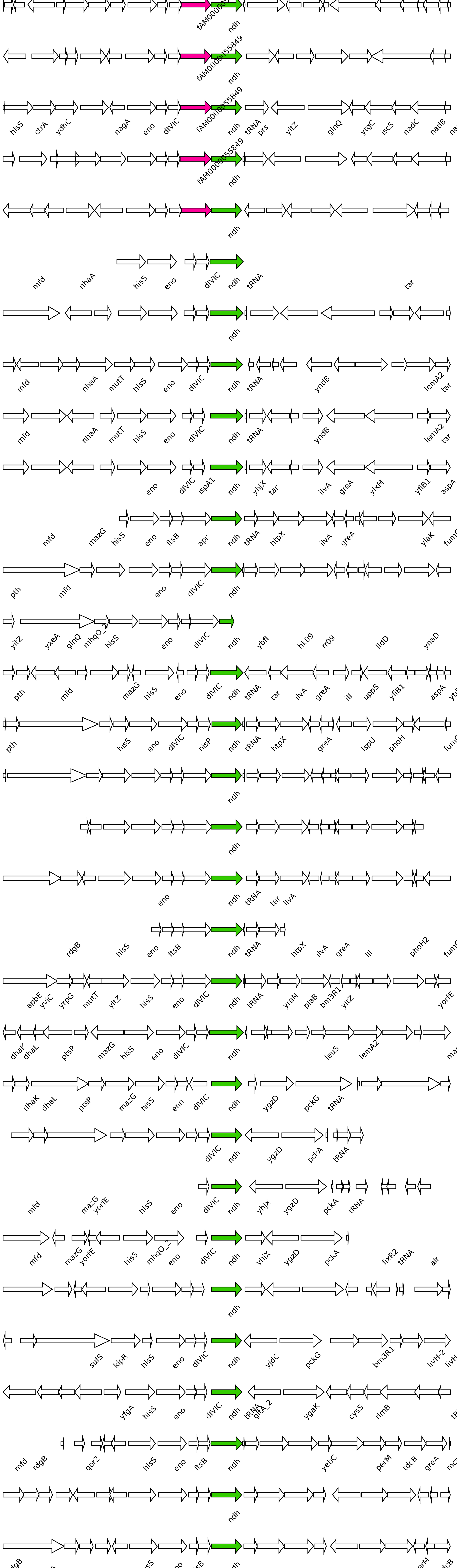

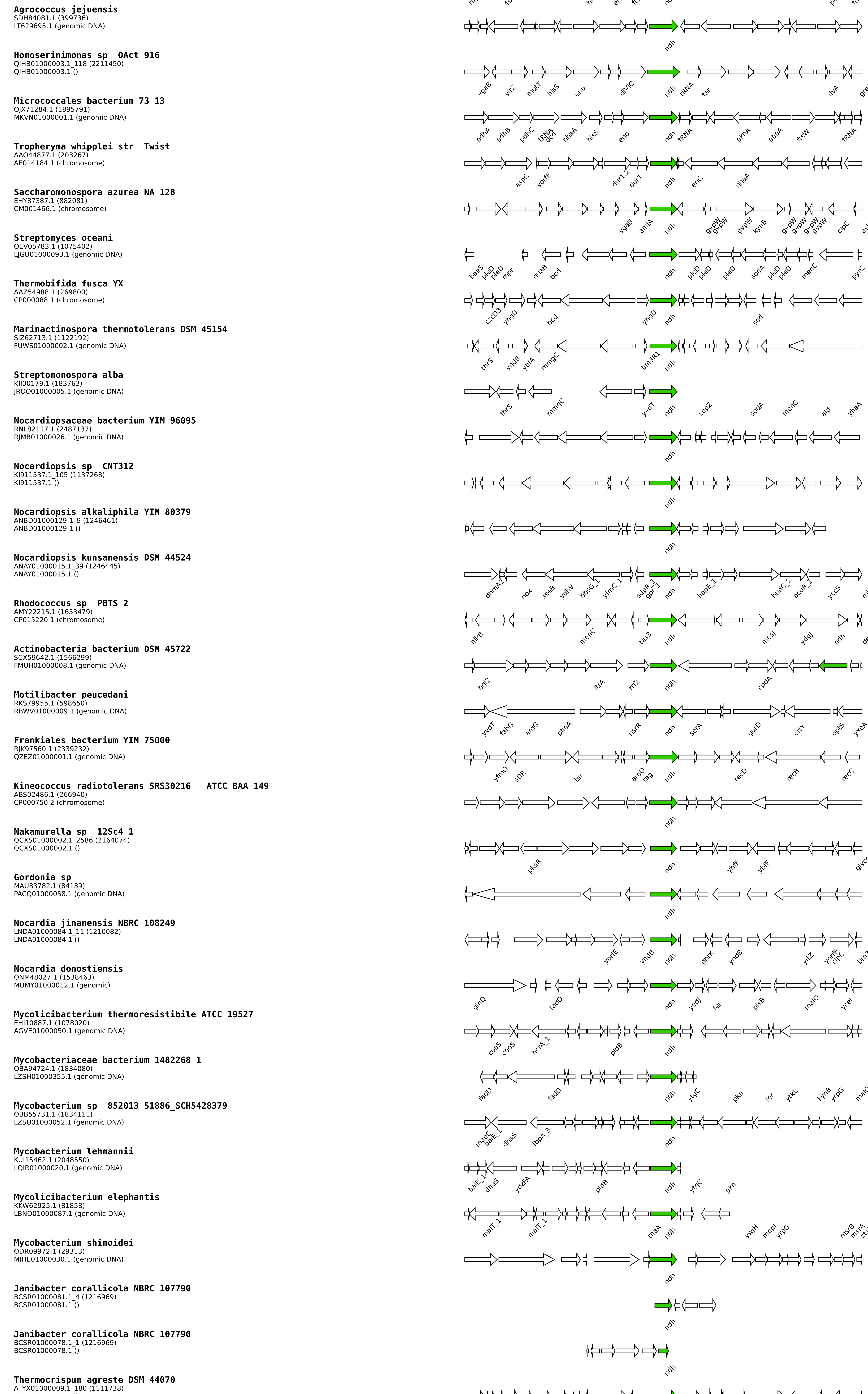
Agrococcus jejuensis
SDH84081.1 (399736)
LT629695.1 (genomic DNA)
Homoserinimonas sp OAct 916
QJHB01000003.1_118 (2211450)
QJHB01000003.1 ()
Micrococcales bacterium 73 13
OJX71284.1 (1895791)
MKVN01000001.1 (genomic DNA)
Tropheryma whipplei str Twist
AAO44877.1 (203267)
AE014184.1 (chromosome)
Saccharomonospora azurea NA 128
EHY87387.1 (882081)
CM001466.1 (chromosome)
Streptomyces oceani
OEV05783.1 (1075402)
LJGU01000093.1 (genomic DNA)
Thermobifida fusca YX
AAZ54988.1 (269800)
CP000088.1 (chromosome)
Marinactinospora thermotolerans DSM 45154
SJZ62713.1 (1122192)
FUWS01000002.1 (genomic DNA)
Streptomonospora alba
KII00179.1 (183763)
JROO01000005.1 (genomic DNA)
Nocardiopsaceae bacterium YIM 96095
RNL82117.1 (2487137)
RJMB01000026.1 (genomic DNA)
Nocardiopsis sp CNT312
KI911537.1_105 (1137268)
KI911537.1 ()
Nocardiopsis alkaliphila YIM 80379
ANBD01000129.1_9 (1246461)
ANBD01000129.1 ()
Nocardiopsis kunsanensis DSM 44524
ANAY01000015.1_39 (1246445)
ANAY01000015.1 ()
Rhodococcus sp PBTS 2
AMY22215.1 (1653479)
CP015220.1 (chromosome)
Actinobacteria bacterium DSM 45722
SCX59642.1 (1566299)
FMUH01000008.1 (genomic DNA)
Motilibacter peucedani
RKS79955.1 (598650)
RBWV01000009.1 (genomic DNA)
Frankiales bacterium YIM 75000
RJK97560.1 (2339232)
QZEZ01000001.1 (genomic DNA)
Kineococcus radiotolerans SRS30216 ATCC BAA 149
ABS02486.1 (266940)
CP000750.2 (chromosome)
Nakamurella sp 12Sc4 1
QCXS01000002.1_2586 (2164074)
QCXS01000002.1 ()
Gordonia sp
MAU83782.1 (84139)
PACQ01000058.1 (genomic DNA)
Nocardia jinanensis NBRC 108249
LNDA01000084.1_11 (1210082)
LNDA01000084.1 ()
Nocardia donostiensis
ONM48027.1 (1538463)
MUMY01000012.1 (genomic)
Mycolicibacterium thermoresistibile ATCC 19527
EHI10887.1 (1078020)
AGVE01000050.1 (genomic DNA)
Mycobacteriaceae bacterium 1482268 1
OBA94724.1 (1834080)
LZSH01000355.1 (genomic DNA)
Mycobacterium sp 852013 51886_SCH5428379
OBB55731.1 (1834111)
LZSU01000052.1 (genomic DNA)
Mycobacterium lehmannii
KUI15462.1 (2048550)
LQIR01000020.1 (genomic DNA)
Mycolicibacterium elephantis
KKW62925.1 (81858)
LBNO01000087.1 (genomic DNA)
Mycobacterium shimoidei
ODR09972.1 (29313)
MIHE01000030.1 (genomic DNA)
Janibacter corallicola NBRC 107790
BCSR01000081.1_4 (1216969)
BCSR01000081.1 ()
Janibacter corallicola NBRC 107790
BCSR01000078.1_1 (1216969)
BCSR01000078.1 ()
Thermocrispum agreste DSM 44070
ATYX01000009.1_180 (1111738)

ATYX01000009.1 ()

Prauserella sp Am3
KID29820.1 (1515610)
JTJI01000007.1 (genomic DNA)

Actinopolyspora alba
SFE34245.1 (673379)
FOMZ01000011.1 (genomic DNA)

Frankia sp Iso899
ATXA01000001.1_381 (1283283)
ATXA01000001.1 ()

Frankia sp Iso899
ATXA01000019.1_28 (1283283)
ATXA01000019.1 ()

Streptomyces sp TP A0874
BBZK01000004.1_145 (549819)
BBZK01000004.1 ()

Salinispora arenicola CNS 205
ABV98130.1 (391037)
CP000850.1 (chromosome)

Streptomyces somaliensis DSM 40738
AJJM01000013.1_25 (1134445)
AJJM01000013.1 ()

Streptomyces sp TAA040
AUKR01000025.1_71 (1288083)
AUKR01000025.1 ()

Rhodospirillaceae bacterium BRH_c57
KJS36353.1 (1629718)
LAEA01000201.1 (genomic DNA)

Hyphomicrobiaceae bacterium UBA4118
DFXC01000192.1_3 (1951936)
DFXC01000192.1 ()

Methyloceanibacter marginalis
ODS04043.1 (1774971)
LPWD01000023.1 (genomic DNA)

Methyloceanibacter caenitepidi
BAQ16057.1 (1384459)
AP014648.1 (genomic DNA)

Methyloligella halotolerans
ODA67759.1 (1177755)
MASI01000002.1 (genomic DNA)

Methyloceanibacter marginalis
ODS04042.1 (1774971)
LPWD01000023.1 (genomic DNA)

Flavobacteriaceae bacterium Hp12
PVZ64349.1 (2171752)
QDDL01000013.1 (genomic DNA)

Flavobacteriaceae bacterium Hp12
PVZ71893.1 (2171752)
QDDL01000001.1 (genomic DNA)

Rubrimonas cliftonensis
SEA72123.1 (89524)
FNQM01000010.1 (genomic DNA)

Rhodobacteraceae bacterium DSL 40
QKPN01000002.1_279 (2219044)
QKPN01000002.1 ()

Amaricoccus sp HAR UPW R2A 40
PJN95414.1 (1985299)
PHNO01000461.1 (genomic DNA)

Tropicibacter naphthalenivorans
CUH82516.1 (441103)
CYSE01000014.1 (genomic DNA)

Thioclava sp SK 1
OCX62821.1 (1889770)
MDHA01000054.1 (genomic DNA)

Actibacterium atlanticum
KCV83287.1 (1461693)
AQQY01000001.1 (genomic DNA)

Thalassococcus sp WRAS1
RDD66366.1 (2282382)
QPMK01000006.1 (genomic DNA)

Rhodobacteraceae bacterium TG 679
PWR02114.1 (2072018)
QGKU01000041.1 (genomic DNA)

Rhodobacter vinaykumarii
SIS77283.1 (407234)
FTOM01000004.1 (genomic DNA)

Rhodobacter veldkampii DSM 11550
PTE19063.1 (1185920)
PZKF01000002.1 (genomic DNA)

Rhodobacter sp CZR27
CP023549.1_81 (2033869)
CP023549.1 ()

Rhodobacter sphaeroides ATCC 17029
ABN78535.1 (349101)
CP000578.1 (chromosome)

Rhodobacter capsulatus SB 1003
ADE86315.1 (272942)
CP001312.1 (chromosome)

Rhodovulum sp MB263
ARC88194.1 (308754)
CP020384.1 (chromosome)

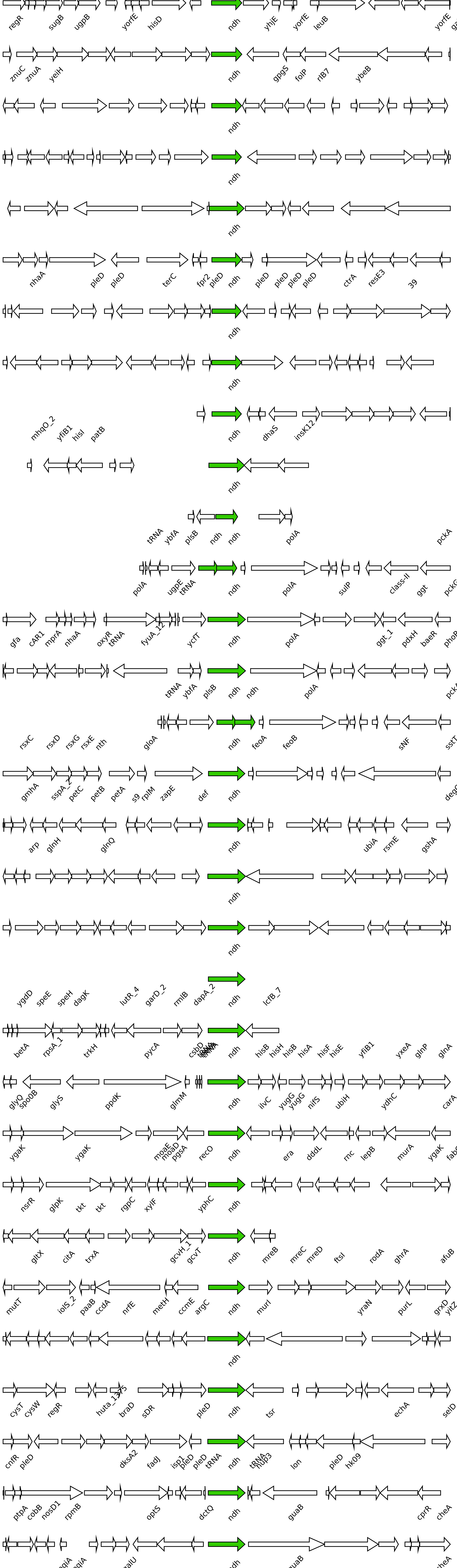

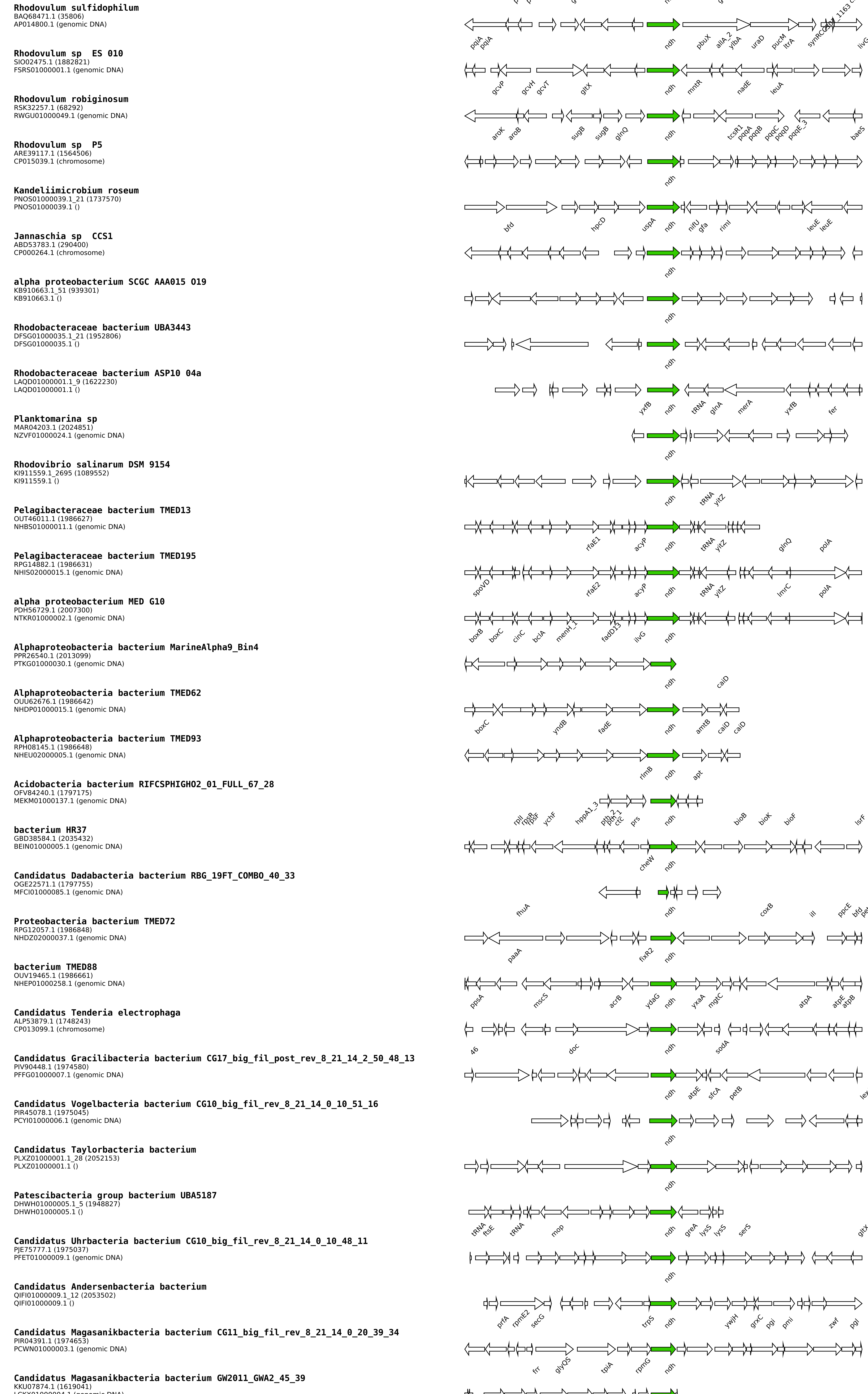
Rhodovulum sulfidophilum
BAQ68471.1 (35806)
AP014800.1 (genomic DNA)
Rhodovulum sp ES 010
SIO02475.1 (1882821)
FSRS01000001.1 (genomic DNA)
Rhodovulum robiginosum
RSK32257.1 (68292)
RWGU01000049.1 (genomic DNA)
Rhodovulum sp P5
ARE39117.1 (1564506)
CP015039.1 (chromosome)
Kandeliimicrobium roseum
PNOS01000039.1_21 (1737570)
PNOS01000039.1 ()
Jannaschia sp CCS1
ABD53783.1 (290400)
CP000264.1 (chromosome)
alpha proteobacterium SCGC AAA015 O19
KB910663.1_51 (939301)
KB910663.1 ()
Rhodobacteraceae bacterium UBA3443
DFSG01000035.1_21 (1952806)
DFSG01000035.1 ()
Rhodobacteraceae bacterium ASP10 04a
LAQD01000001.1_9 (1622230)
LAQD01000001.1 ()
Planktomarina sp
MAR04203.1 (2024851)
NZVF01000024.1 (genomic DNA)
Rhodovibrio salinarum DSM 9154
KI911559.1_2695 (1089552)
KI911559.1 ()
Pelagibacteraceae bacterium TMED13
OUT46011.1 (1986627)
NHBS01000011.1 (genomic DNA)
Pelagibacteraceae bacterium TMED195
RPG14882.1 (1986631)
NHIS02000015.1 (genomic DNA)
alpha proteobacterium MED G10
PDH56729.1 (2007300)
NTKR01000002.1 (genomic DNA)
Alphaproteobacteria bacterium MarineAlpha9_Bin4
PPR26540.1 (2013099)
PTKG01000030.1 (genomic DNA)
Alphaproteobacteria bacterium TMED62
OUU62676.1 (1986642)
NHDP01000015.1 (genomic DNA)
Alphaproteobacteria bacterium TMED93
RPH08145.1 (1986648)
NHEU02000005.1 (genomic DNA)
Acidobacteria bacterium RIFCSPHIGHO2_01_FULL_67_28
OFV84240.1 (1797175)
MEKM01000137.1 (genomic DNA)
bacterium HR37
GBD38584.1 (2035432)
BEIN01000005.1 (genomic DNA)
Candidatus Dadabacteria bacterium RBG_19FT_COMBO_40_33
OGE22571.1 (1797755)
MFCI01000085.1 (genomic DNA)
Proteobacteria bacterium TMED72
RPG12057.1 (1986848)
NHDZ02000037.1 (genomic DNA)
bacterium TMED88
OUV19465.1 (1986661)
NHEP01000258.1 (genomic DNA)
Candidatus Tenderia electrophaga
ALP53879.1 (1748243)
CP013099.1 (chromosome)
Candidatus Gracilibacteria bacterium CG17_big_fil_post_rev_8_21_14_2_50_48_13
PIV90448.1 (1974580)
PFFG01000007.1 (genomic DNA)
Candidatus Vogelbacteria bacterium CG10_big_fil_rev_8_21_14_0_10_51_16
PIR45078.1 (1975045)
PCYI01000006.1 (genomic DNA)
Candidatus Taylorbacteria bacterium
PLXZ01000001.1_28 (2052153)
PLXZ01000001.1 ()
Patescibacteria group bacterium UBA5187
DHWH01000005.1_5 (1948827)
DHWH01000005.1 ()
Candidatus Uhrbacteria bacterium CG10_big_fil_rev_8_21_14_0_10_48_11
PJE75777.1 (1975037)
PFET01000009.1 (genomic DNA)
Candidatus Andersenbacteria bacterium
QIFI01000009.1_12 (2053502)
QIFI01000009.1 ()
Candidatus Magasanikbacteria bacterium CG11_big_fil_rev_8_21_14_0_20_39_34
PIR04391.1 (1974653)
PCWN01000003.1 (genomic DNA)
Candidatus Magasanikbacteria bacterium GW2011_GWA2_45_39
KKU07874.1 (1619041)

LCKX01000004.1 (genomic DNA)

Candidatus Magasanikbacteria bacterium CG10_big_fil_rev_8_21_14_0_10_47_10
PIR74778.1 (1974652)
PFCB01000006.1 (genomic DNA)

Candidatus Andersenbacteria bacterium RIFCSPHIGHO2_12_FULL_45_11b
OGY37197.1 (1797282)
MHHS01000016.1 (genomic DNA)

Candidatus Andersenbacteria bacterium RIFCSPHIGHO2_12_FULL_45_11
OGY33970.1 (1797281)
MHHR01000024.1 (genomic DNA)

Candidatus Harrisonbacteria bacterium CG10_big_fil_rev_8_21_14_0_10_42_17
PIT92062.1 (1974584)
PFBA01000035.1 (genomic DNA)

Candidatus Ryanbacteria bacterium CG10_big_fil_rev_8_21_14_0_10_43_42
PJE64085.1 (1974864)
PFEF01000010.1 (genomic DNA)

Candidatus Ryanbacteria bacterium RIFCSPHIGHO2_02_FULL_45_13b
OGZ45018.1 (1802117)
MHNN01000025.1 (genomic DNA)

Candidatus Ryanbacteria bacterium RIFCSPHIGHO2_01_FULL_45_22
OGZ43890.1 (1802114)
MHNK01000010.1 (genomic DNA)

Candidatus Magasanikbacteria bacterium CG10_big_fil_rev_8_21_14_0_10_43_6
PIT86386.1 (1974650)
PFBZ01000151.1 (genomic DNA)

Candidatus Vogelbacteria bacterium CG10_big_fil_rev_8_21_14_0_10_45_14
PIR47186.1 (1975042)
PCYL01000004.1 (genomic DNA)

Armatimonadetes bacterium CSP1 3
KRT76229.1 (1640515)
LDXQ01000058.1 (genomic DNA)

Natronorubrum thiooxidans
SIS13744.1 (308853)
FTNR01000013.1 (genomic DNA)

Chloracidobacterium sp CP2_5A
OYT69990.1 (2012633)
NKPT01000047.1 (genomic DNA)

Chloracidobacterium thermophilum B
AEP13790.1 (981222)
CP002515.1 (chromosome)

Candidatus Methylomirabilis lanthanidiphila
SYW89072.1 (2211376)
ULHC01000022.1 (genomic DNA)

Candidatus Methylomirabilis oxyfera
CBE68759.1 (671143)
FP565575.1 (genomic DNA)

Candidatus Methylomirabilis sp
NSJM01000111.1_1 (2032687)
NSJM01000111.1 ()

candidate division NC10 bacterium
PWB46002.1 (2072417)
PQAM01000010.1 (genomic DNA)

Candidatus Methylomirabilis sp
NSJM01000038.1_1 (2032687)
NSJM01000038.1 ()

Nocardioides sp CCTCC AB 2018079
PWN03963.1 (2201891)
QGDD01000002.1 (genomic DNA)

Euzebya rosea
PGDQ01000006.1_18 (2052804)
PGDQ01000006.1 ()

Myxococcales bacterium UBA4151
DFVV01000155.1_12 (1961466)
DFVV01000155.1 ()

Verrucomicrobia bacterium
ATC64884.1 (2026799)
CP023344.1 (chromosome)

Candidatus Marsarchaeota G2 archaeon ECH_B_3
PSN99434.1 (1978161)
NEXI01000016.1 (genomic DNA)

Thermobaculum terrenum ATCC BAA 798
ACZ41511.1 (525904)
CP001825.1 (chromosome)

Chloroflexi bacterium RBG_13_51_36
OGN97207.1 (1797625)
MGMS01000016.1 (genomic DNA)

Halodesulfovibrio marinisediminis DSM 17456
SIN93049.1 (1121457)
FSRG01000004.1 (genomic DNA)

Spirochaetia bacterium UBA5368
DHPI01000022.1_121 (1948758)
DHPI01000022.1 ()

Spirochaetes bacterium RBG_13_51_14
OHD63351.1 (1802193)
MIBH01000072.1 (genomic DNA)

Spirochaetia bacterium UBA4802
DHGI01000003.1_30 (1948757)
DHGI01000003.1 ()

Chroococcidiopsis sp TS 821
PPS44097.1 (1378066)
MVDI01000002.1 (genomic)

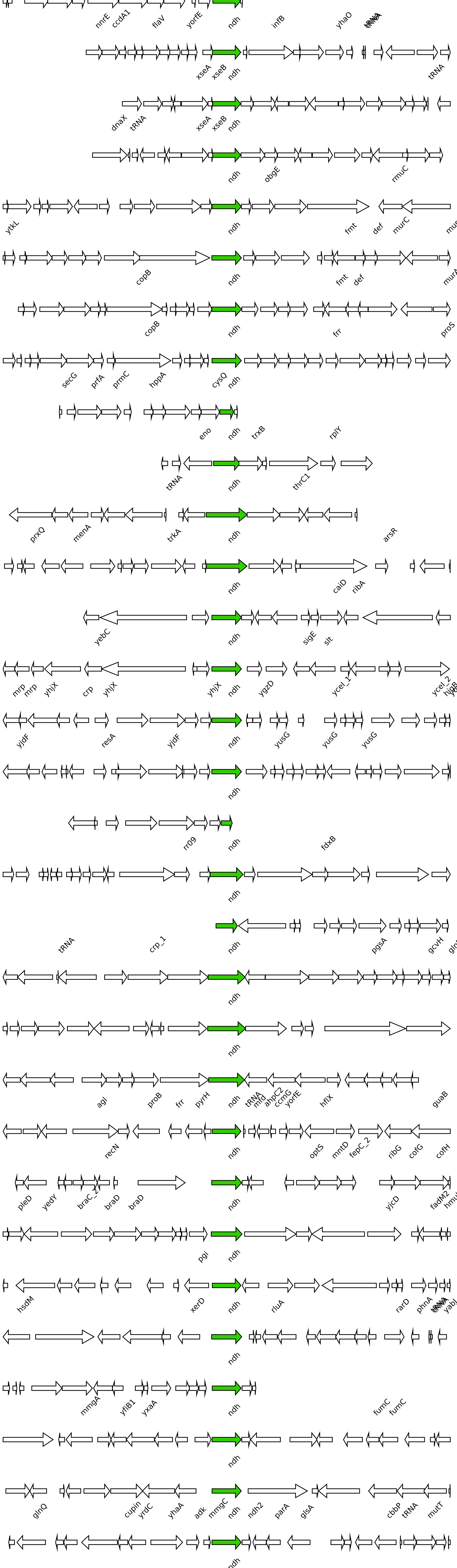

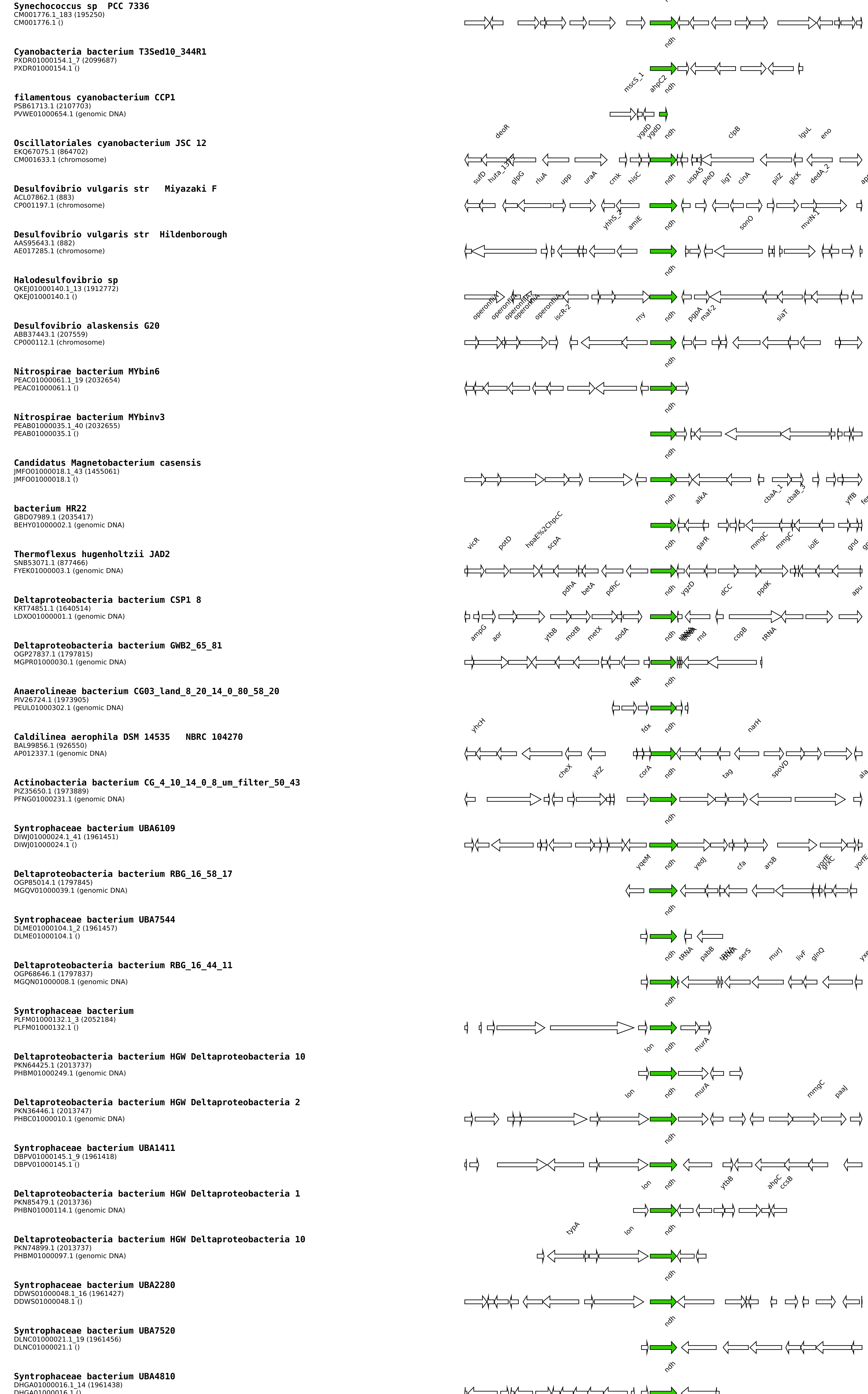
Synechococcus sp PCC 7336
CM001776.1_183 (195250)
CM001776.1 ()
Cyanobacteria bacterium T3Sed10_344R1
PXDR01000154.1_7 (2099687)
PXDR01000154.1 ()
filamentous cyanobacterium CCP1
PSB61713.1 (2107703)
PVWE01000654.1 (genomic DNA)
Oscillatoriales cyanobacterium JSC 12
EKQ67075.1 (864702)
CM001633.1 (chromosome)
Desulfovibrio vulgaris str Miyazaki F
ACL07862.1 (883)
CP001197.1 (chromosome)
Desulfovibrio vulgaris str Hildenborough
AAS95643.1 (882)
AE017285.1 (chromosome)
Halodesulfovibrio sp
QKEJ01000140.1_13 (1912772)
QKEJ01000140.1 ()
Desulfovibrio alaskensis G20
ABB37443.1 (207559)
CP000112.1 (chromosome)
Nitrospirae bacterium MYbin6
PEAC01000061.1_19 (2032654)
PEAC01000061.1 ()
Nitrospirae bacterium MYbinv3
PEAB01000035.1_40 (2032655)
PEAB01000035.1 ()
Candidatus Magnetobacterium casensis
JMFO01000018.1_43 (1455061)
JMFO01000018.1 ()
bacterium HR22
GBD07989.1 (2035417)
BEHY01000002.1 (genomic DNA)
Thermoflexus hugenholtzii JAD2
SNB53071.1 (877466)
FYEK01000003.1 (genomic DNA)
Deltaproteobacteria bacterium CSP1 8
KRT74851.1 (1640514)
LDXO01000001.1 (genomic DNA)
Deltaproteobacteria bacterium GWB2_65_81
OGP27837.1 (1797815)
MGPR01000030.1 (genomic DNA)
Anaerolineae bacterium CG03_land_8_20_14_0_80_58_20
PIV26724.1 (1973905)
PEUL01000302.1 (genomic DNA)
Caldilinea aerophila DSM 14535 NBRC 104270
BAL99856.1 (926550)
AP012337.1 (genomic DNA)
Actinobacteria bacterium CG_4_10_14_0_8_um_filter_50_43
PIZ35650.1 (1973889)
PFNG01000231.1 (genomic DNA)
Syntrophaceae bacterium UBA6109
DIWJ01000024.1_41 (1961451)
DIWJ01000024.1 ()
Deltaproteobacteria bacterium RBG_16_58_17
OGP85014.1 (1797845)
MGQV01000039.1 (genomic DNA)
Syntrophaceae bacterium UBA7544
DLME01000104.1_2 (1961457)
DLME01000104.1 ()
Deltaproteobacteria bacterium RBG_16_44_11
OGP68646.1 (1797837)
MGQN01000008.1 (genomic DNA)
Syntrophaceae bacterium
PLFM01000132.1_3 (2052184)
PLFM01000132.1 ()
Deltaproteobacteria bacterium HGW Deltaproteobacteria 10
PKN64425.1 (2013737)
PHBM01000249.1 (genomic DNA)
Deltaproteobacteria bacterium HGW Deltaproteobacteria 2
PKN36446.1 (2013747)
PHBC01000010.1 (genomic DNA)
Syntrophaceae bacterium UBA1411
DBPV01000145.1_9 (1961418)
DBPV01000145.1 ()
Deltaproteobacteria bacterium HGW Deltaproteobacteria 1
PKN85479.1 (2013736)
PHBN01000114.1 (genomic DNA)
Deltaproteobacteria bacterium HGW Deltaproteobacteria 10
PKN74899.1 (2013737)
PHBM01000097.1 (genomic DNA)
Syntrophaceae bacterium UBA2280
DDWS01000048.1_16 (1961427)
DDWS01000048.1 ()
Syntrophaceae bacterium UBA7520
DLNC01000021.1_19 (1961456)
DLNC01000021.1 ()
Syntrophaceae bacterium UBA4810
DHGA01000016.1_14 (1961438)
DHGA01000016.1 ()

DHGA01000010.1 ()

Clostridia bacterium
PKEP01000101.1_9 (2044939)
PKEP01000101.1 ()

Limnochorda pilosa
BAS26466.1 (1555112)
AP014924.1 (genomic DNA)

Syntrophaceae bacterium UBA1163
DBZJ01000173.1_12 (1961416)
DBZJ01000173.1 ()

Candidatus Magnetoovum chiemensis
KJR43271.1 (1609970)
JZJI01000223.1 (genomic DNA)

candidate division Zixibacteria bacterium RBG_16_53_22
OGC92027.1 (1802787)
MEWP01000104.1 (genomic DNA)

Deltaproteobacteria bacterium YD0425bin51
PDZS01000031.1_33 (2032657)
PDZS01000031.1 ()

Desulfovibrio sp L21 Syr AB
JOMJ01000004.1_468 (1307759)
JOMJ01000004.1 ()

Desulfovibrio sp X2
EPR37596.1 (941449)
ATHV01000064.1 (genomic DNA)

Desulfovibrio alkalitolerans DSM 16529
EPR34929.1 (1121439)
ATHI01000005.1 (genomic DNA)

Clostridiales bacterium PH28_bin88
KKM08551.1 (1605376)
LAKY01000080.1 (genomic DNA)

uncultured delta proteobacterium
SBW06253.1 (34034)
LT599018.1 (genomic DNA)

Herpetosiphon sp
HBW48474.1 (71864)
DOPT01000005.1 (genomic DNA)

Chloroflexus aurantiacus J 10 fl
ABY36543.1 (324602)
CP000909.1 (chromosome)

Candidatus Thermofonsia Clade 3 bacterium
PJF45495.1 (2364212)
PGTN01001036.1 (genomic DNA)

Oscillochloris trichoides DG 6
EFO79540.1 (765420)
GL501404.1 (genomic)

Chloroflexaceae bacterium UBA1466
DCSM01000118.1_4 (1951568)
DCSM01000118.1 ()

Candidatus Jettenia caeni
GAB61072.1 (247490)
BAFH01000002.1 (genomic DNA)

Geobacter sp DSM 2909
PTV80629.1 (2135459)
QAXR01000044.1 (genomic DNA)

Geobacteraceae bacterium GWC2_53_11
OGU14351.1 (1798316)
MGZJ01000070.1 (genomic DNA)

Geobacter sp UBA4050
DFZS01000004.1_14 (1961391)
DFZS01000004.1 ()

Geobacter sp UBA4057
DFZL01000028.1_138 (1961392)
DFZL01000028.1 ()

Deltaproteobacteria bacterium HGW Deltaproteobacteria 4
PKN13426.1 (2013754)
PHAV01000002.1 (genomic DNA)

Geobacter lovleyi SZ
ACD94756.1 (398767)
CP001089.1 (chromosome)

Geobacter sp UBA6802
DKFG01000053.1_2 (1961401)
DKFG01000053.1 ()

Geoalkalibacter ferrihydriticus DSM 17813
KIH77215.1 (1121915)
JWJD01000001.1 (genomic DNA)

Desulfuromonadaceae bacterium UBA2294
DDWE01000085.1_2 (1961570)
DDWE01000085.1 ()

Geobacter lovleyi SZ
ACD93946.1 (398767)
CP001089.1 (chromosome)

Geobacteraceae bacterium GWC2_55_20
OGU03173.1 (1798317)
MGZK01000129.1 (genomic DNA)

Geobacter sp OR 1
GAM09907.1 (1266765)
BAZF01000008.1 (genomic DNA)

Geobacter metallireducens GS 15
ABB33256.1 (269799)
CP000148.1 (chromosome)

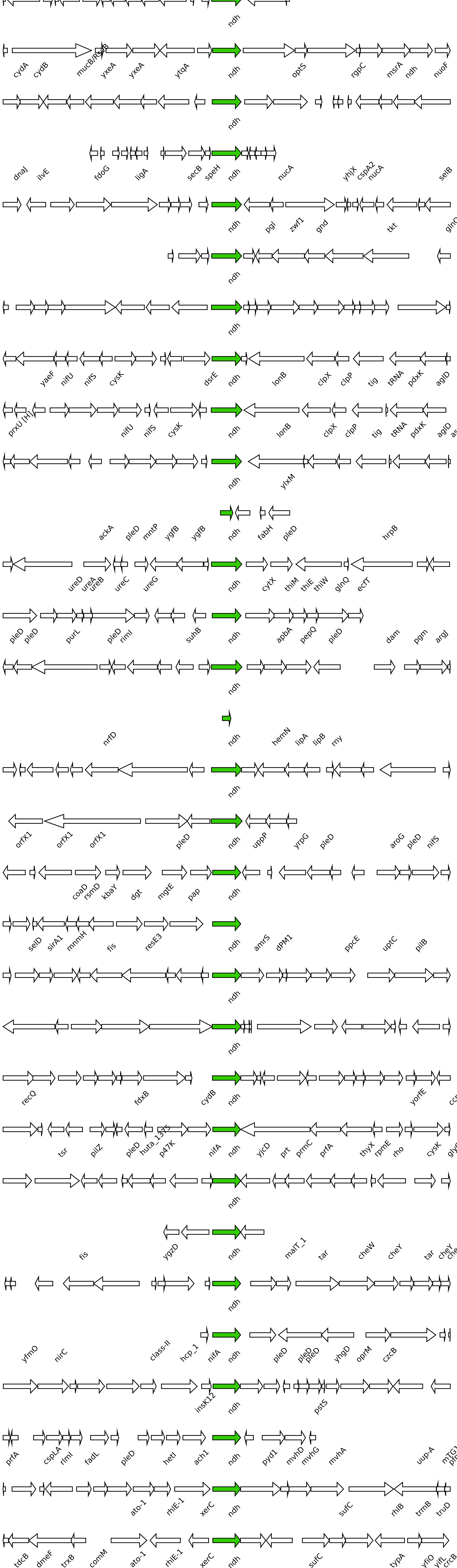

Geobacter sulfurreducens PCA
AAR33825.1 (243231)
AE017180.2 (chromosome)

Geobacter pickeringii
AJE04946.1 (345632)
CP009788.1 (chromosome)

Geobacter sp DSM 9736
SNB45210.1 (1277350)
LT896716.1 (genomic DNA)

Anaerolineales bacterium UBA3940
DGDY01000127.1_4 (1950207)
DGDY01000127.1 ()

Longilinea arvoryzae
GAP13280.1 (360412)
DF967972.1 (genomic DNA)

Chloroflexi bacterium GWB2_54_36
OGN82354.1 (1797613)
MGMG01000005.1 (genomic DNA)

Anaerolineales bacterium Chloro_03
LUZW01000164.1_3 (1674892)
LUZW01000164.1 ()

Bellilinea caldifistulae
GAP09270.1 (360411)
DF967971.1 (genomic DNA)

Anaerolinea thermophila UNI 1
BAJ64893.1 (926569)
AP012029.1 (genomic DNA)

Anaerolineales bacterium UBA2285
DDWN01000092.1_55 (1950193)
DDWN01000092.1 ()

Chloroflexi bacterium HGW Chloroflexi 6
PKN94625.1 (2013729)
PHBU01000003.1 (genomic DNA)

Lentisphaerae bacterium GWF2_57_35
OGV41528.1 (1798576)
MHBK01000064.1 (genomic DNA)

Chloroflexi bacterium RBG_13_68_17
OGO15734.1 (1797638)
MGNF01000032.1 (genomic DNA)

Gemmatimonadetes bacterium 13_1_20CM_4_69_16
OLD95940.1 (1805174)
MNIR01000030.1 (genomic DNA)

Sporomusaceae bacterium UBA7701
DLGF01000014.1_84 (1953031)
DLGF01000014.1 ()

Anaerolineales bacterium UBA5796
DIDQ01000164.1_18 (1950216)
DIDQ01000164.1 ()

Gallionellales bacterium RBG_16_56_9
OGS96457.1 (1798249)
MGWU01000188.1 (genomic DNA)

Chloroflexi bacterium RBG_16_51_16
OGO24708.1 (1797644)
MGNL01000056.1 (genomic DNA)

Anaerolineales bacterium UBA5797
DIDP01000024.1_4 (1950217)
DIDP01000024.1 ()

Anaerolineae bacterium UTCFX3
OQY84132.1 (1961102)
MWSZ01000119.1 (genomic DNA)

Anaerolineales bacterium UBA2317
DDVH01000004.1_33 (1950194)
DDVH01000004.1 ()

Anaerolineales bacterium UBA2356
DDTU01000029.1_5 (1950196)
DDTU01000029.1 ()

Anaerolineaceae bacterium UBA3924
DGEO01000045.1_14 (1951408)
DGEO01000045.1 ()

Methylomusa anaerophila
BBB91875.1 (1930071)
AP018449.1 (genomic DNA)

Acetonema longum DSM 6540
EGO62177.1 (1009370)
AFGF01000240.1 (genomic)

Propionispora sp 2 2 37
CUH94584.1 (1677858)
CYSP01000002.1 (genomic DNA)

Propionispora vibrioides
SEP16918.1 (112903)
FODY01000012.1 (genomic DNA)

Anaerolinea thermolimosa
GAP07417.1 (229919)
DF967965.1 (genomic DNA)

Chloroflexi bacterium HGW Chloroflexi 2
PKO08005.1 (2013725)
PHBY01000027.1 (genomic DNA)

Chloroflexi bacterium HGW Chloroflexi 8
PKN85495.1 (2013731)
PHBS01000075.1 (genomic DNA)

Chloroflexi bacterium 44 23
OJX40668.1 (1895927)
MKUW01000019.1 (genomic DNA)

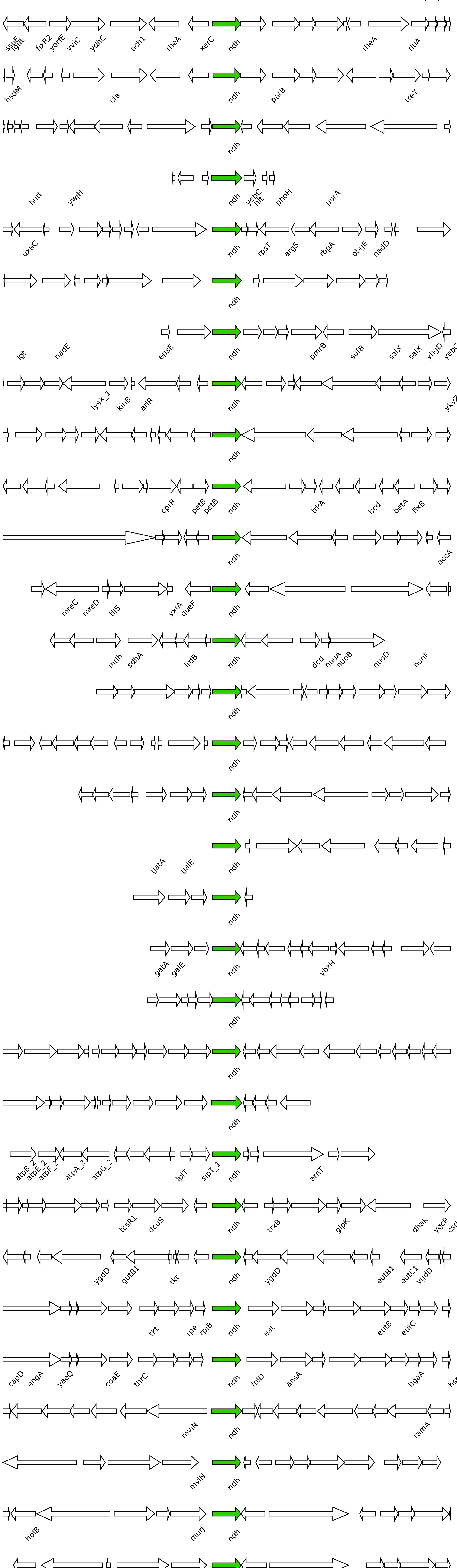

Anaeromusa acidaminophila DSM 3853
KB894582.1_99 (1120992)
KB894582.1 ()

Anaerosporomusa subterranea
KYZ77758.1 (1794912)
LSGP01000008.1 (genomic DNA)

Pelosinus fermentans JBW45
AJQ25810.1 (1192197)
CP010978.1 (chromosome)

Pelosinus fermentans JBW45
AJQ28248.1 (1192197)
CP010978.1 (chromosome)

Dendrosporobacter quercicolus
SDN35579.1 (146817)
FNHB01000021.1 (genomic DNA)

Sporomusa malonica
SMD09250.1 (112901)
FWXI01000024.1 (genomic DNA)

Firmicutes bacterium UBA5324
DHRA01000053.1_2 (1948050)
DHRA01000053.1 ()

Anaerosporomusa subterranea
KYZ77671.1 (1794912)
LSGP01000012.1 (genomic DNA)

bacterium JKG1
JAGA01000002.1_1725 (1128421)
JAGA01000002.1 ()

Anaeromusa acidaminophila DSM 3853
KB894587.1_107 (1120992)
KB894587.1 ()

Propionispira raffinosivorans DSM 20765
KB905839.1_308 (1123511)
KB905839.1 ()

Massilibacillus massiliensis
LT575483.1_1089 (1806837)
LT575483.1 ()

Propionispira raffinosivorans DSM 20765
KB905839.1_287 (1123511)
KB905839.1 ()

Megamonas sp UBA1792
DCFY01000001.1_1 (1946840)
DCFY01000001.1 ()

Megamonas sp UBA1796
DCFU01000011.1_18 (1946841)
DCFU01000011.1 ()

Selenomonas sputigena ATCC 35185
EEX75988.1 (546271)
GG698599.1 (genomic)

Selenomonadaceae bacterium UBA7286
DKRI01000024.1_4 (1953026)
DKRI01000024.1 ()

Selenomonadaceae bacterium UBA2829
DEGJ01000015.1_2 (1953021)
DEGJ01000015.1 ()

Selenomonas ruminantium subsp lactilytica TAM6421
BAL84014.1 (927704)
AP012292.1 (genomic DNA)

Selenomonas sp ND2010
JNKI01000062.1_1 (1410618)
JNKI01000062.1 ()

Mitsuokella jalaludinii
CUO02266.1 (187979)
CYYU01000020.1 (genomic DNA)

Mitsuokella sp oral taxon 131 str W9106
ERL05621.1 (1321781)
KI273050.1 (genomic)

uncultured Veillonellaceae bacterium
OMTH01000147.1_1 (194923)
OMTH01000147.1 ()

Selenomonas ruminantium AB3002
JNIO01000002.1_380 (1392502)
JNIO01000002.1 ()

Selenomonadaceae bacterium UBA2913
DEDD01000086.1_2 (1953023)
DEDD01000086.1 ()

Selenomonadales bacterium UBA3815
DFDZ01000058.1_46 (1951242)
DFDZ01000058.1 ()

Selenomonadaceae bacterium UBA3796
DFES01000039.1_3 (1953024)
DFES01000039.1 ()

Anaerovibrio lipolyticus DSM 3074
SHI79330.1 (1120997)
FQYW01000013.1 (genomic DNA)

uncultured Negativicutes bacterium
OMTO01000078.1_21 (1294148)
OMTO01000078.1 ()

Megamonas sp UBA1799
DCFR01000039.1_17 (1946842)
DCFR01000039.1 ()

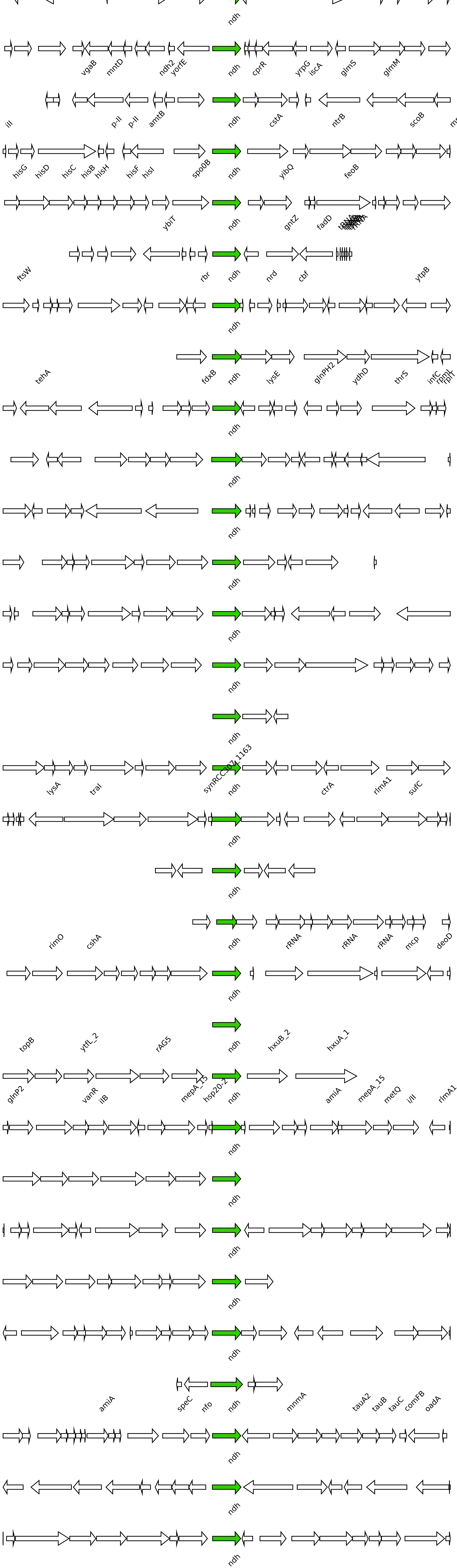

Megamonas hypermegale DSM 1672
AUHW01000005.1_129 (1122216)
AUHW01000005.1 ()

Megamonas hypermegale ART12 1
CBL06668.1 (657316)
FP929048.1 (genomic DNA)

Selenomonas sp F0473
EKU70710.1 (999423)
JH992902.1 (genomic)

Selenomonas sp oral taxon 478
AKT53280.1 (712538)
CP012071.1 (chromosome)

Selenomonas infelix ATCC 43532
EHG20459.1 (679201)
JH376799.1 (genomic)

Selenomonas sp oral taxon 920
AOH48457.1 (1884263)
CP017042.1 (chromosome)

Selenomonas artemidis F0399
EFW30236.1 (749551)
GL638130.1 (genomic)

Selenomonadaceae bacterium UBA2829
DEGJ01000152.1_10 (1953021)
DEGJ01000152.1 ()

Selenomonadaceae bacterium UBA7286
DKRI01000083.1_36 (1953026)
DKRI01000083.1 ()

Candidatus Microthrix parvicella RN1
CCM62571.1 (1229780)
HG422565.1 (genomic DNA)

Euzebya tangerina
PPDK01000002.1_23 (591198)
PPDK01000002.1 ()

Euzebya rosea
PGDQ01000021.1_45 (2052804)
PGDQ01000021.1 ()

Acidimicrobiaceae bacterium UBA668
DBNQ01000341.1_2 (1951340)
DBNQ01000341.1 ()

Rhodothermaceae bacterium RA
ARA94047.1 (1779382)
CP020382.1 (chromosome)

Rhodothermus marinus DSM 4252
ACY49534.1 (518766)
CP001807.1 (chromosome)

bacterium HR18
GBD00365.1 (2035413)
BEHU01000001.1 (genomic DNA)

Rubricoccus marinus
OZC03363.1 (716817)
MQWB01000001.1 (genomic DNA)

Rubrivirga marina
PAP77657.1 (1196024)
MQWD01000001.1 (genomic DNA)

Rubrivirga sp SAORIC476
PAP82424.1 (1961794)
MVOI01000002.1 (genomic DNA)

Bacteroidetes bacterium QS_8_68_28
PSR02467.1 (1919110)
PXTS01000034.1 (genomic DNA)

Bacteroidetes bacterium QS_8_68_15
PSQ80831.1 (1919109)
PXTR01000071.1 (genomic DNA)

Salisaeta longa DSM 21114
ATTH01000001.1_752 (1089550)
ATTH01000001.1 ()

Longibacter salinarum
PEN14414.1 (1850348)
PDEQ01000002.1 (genomic DNA)

Bacteroidetes bacterium SW_9_63_38
PSQ97379.1 (1919118)
PXUA01000005.1 (genomic DNA)

Salinibacter sp 10B
PQJ34115.1 (1923971)
MQWC01000004.1 (genomic DNA)

Salinibacter ruber DSM 13855
ABC43955.1 (309807)
CP000159.1 (chromosome)

Bacteroidetes bacterium QS_1_63_11
PSQ81292.1 (1919103)
PXTM01000070.1 (genomic DNA)

Salinivenus lutea
NCRC01000017.1_16 (890426)
NCRC01000017.1 ()

Rhodothermaceae bacterium Tc Br11_B2g6_7
LKNB01000016.1_8 (1729468)
LKNB01000016.1 ()

Longimonas halophila
PEN08544.1 (1469170)
PDEP01000003.1 (genomic DNA)

Actinobacteria bacterium QS_8_72_14
PSO46852.1 (1919086)
PXPH01000054.1 (genomic DNA)

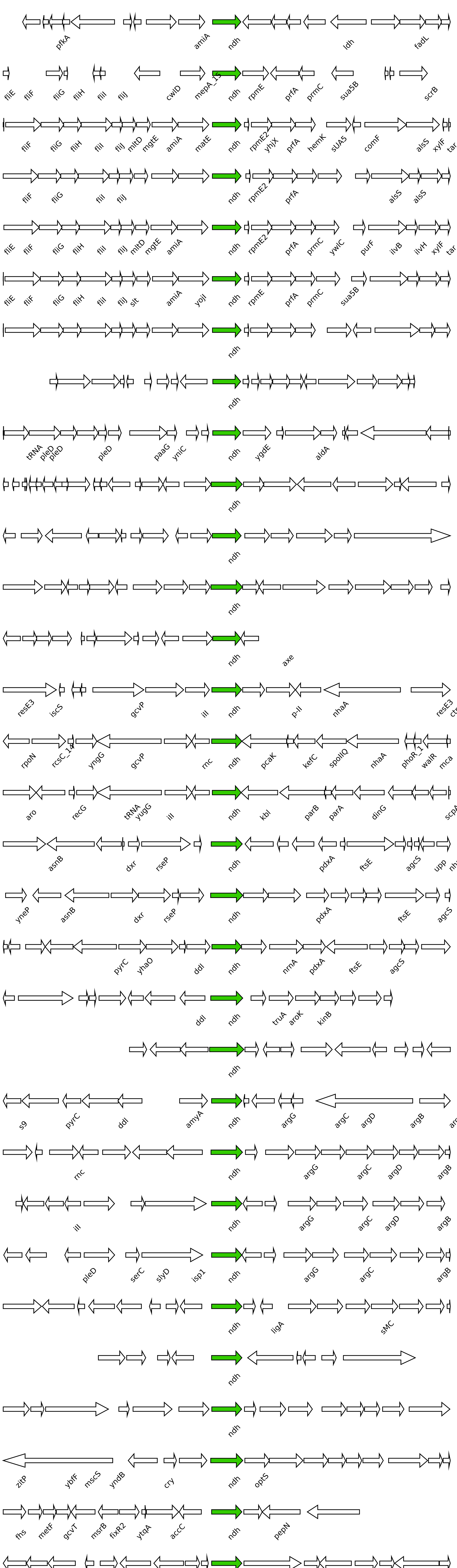

Candidatus Microthrix sp UBA4742
DHIQ01000160.1_2 (1946189)
DHIQ01000160.1 ()

Acidithrix ferrooxidans
KJF16986.1 (1280514)
JXYS01000069.1 (genomic)

Ferrithrix thermotolerans DSM 19514
SHE48762.1 (1121881)
FQUL01000007.1 (genomic DNA)

Ferrimicrobium acidiphilum DSM 19497
JQKF01000003.1_16 (1121877)
JQKF01000003.1 ()

Acidimicrobium ferrooxidans DSM 10331
ACU54464.1 (525909)
CP001631.1 (chromosome)

Actinobacteria bacterium UBA5176
DHWS01000061.1_18 (1953126)
DHWS01000061.1 ()

bacterium HR33
GBD31757.1 (2035428)
BEIJ01000001.1 (genomic DNA)

Gemmatimonadetes bacterium RIFCSPLOWO2_12_FULL_68_9
OGU18502.1 (1798313)
MGZG01000712.1 (genomic DNA)

Angustibacter sp Root456
KQX61939.1 (1736539)
LMER01000020.1 (genomic DNA)

Pseudokineococcus lusitanus
ROP26950.1 (763993)
RJKN01000008.1 (genomic DNA)

Raineyella antarctica
SDB79843.1 (1577474)
FMYF01000001.1 (genomic DNA)

Friedmanniella luteola
SDT36307.1 (546871)
LT629749.1 (genomic DNA)

Friedmanniella sagamiharensis
SDU98832.1 (546874)
LT629799.1 (genomic DNA)

Auraticoccus monumenti
SDD62335.1 (675864)
LT629688.1 (genomic DNA)

Propionibacteriaceae bacterium 16Sb5 5
RCK69598.1 (2268447)
QOUI01000005.1 (genomic DNA)

Propionibacteriaceae bacterium NML 020265
OYO09392.1 (2016499)
NMVP01000036.1 (genomic DNA)

Aestuariimicrobium kwangyangense DSM 21549
ATXE01000004.1_95 (1120954)
ATXE01000004.1 ()

Propionimicrobium lymphophilum ACS 093 V SCH5
EPD32684.1 (883161)
KE150269.1 (genomic)

Propionibacterium sp oral taxon 192 str F0372
EPH05583.1 (1203605)
KE340297.1 (genomic)

Actinomycetales bacterium UBA1383
DBQX01000082.1_12 (1950141)
DBQX01000082.1 ()

Actinomycetales bacterium UBA1416
DBPQ01000098.1_34 (1950186)
DBPQ01000098.1 ()

Mobilicoccus massiliensis
LN651331.1_73 (1522310)
LN651331.1 ()

Demetria terragena DSM 11295
AQXW01000004.1_1946 (1121385)
AQXW01000004.1 ()

Actinomyces hordeovulneris
OKL50944.1 (52770)
MQVS01000012.1 (genomic DNA)

Cutibacterium acnes KPA171202
AAT81910.1 (267747)
AE017283.1 (chromosome)

Acidipropionibacterium jensenii
VEI03099.1 (1749)
LR134473.1 (genomic DNA)

Acidipropionibacterium acidipropionici ATCC 4875
AFV88337.1 (1171373)
CP003493.1 (chromosome)

Propionibacterium acidifaciens
AYW78198.1 (556499)
CP033719.1 (chromosome)

Propionibacterium cyclohexanicum
SER92961.1 (64702)
FOGZ01000019.1 (genomic DNA)

Propionibacteriaceae bacterium NML 150081
OYN88651.1 (2016505)
NMVJ01000011.1 (genomic DNA)

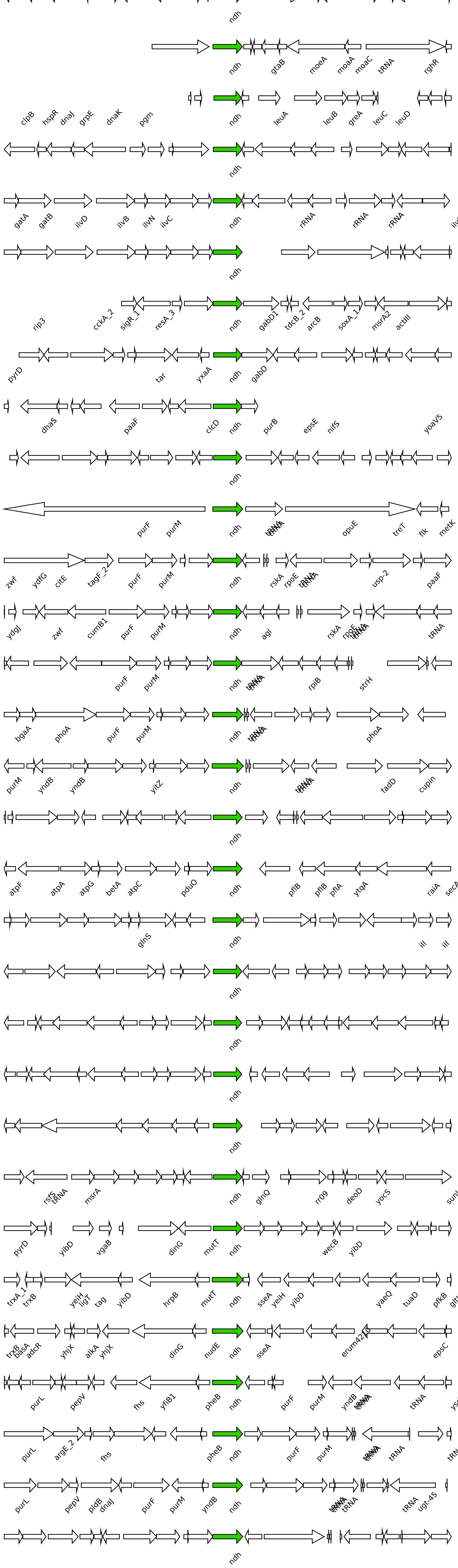

Granulicoccus phenolivorans DSM 17626
AUHH01000031.1_42 (1121933)
AUHH01000031.1 ()

Micrococcales bacterium KH10
SNS66859.1 (1945885)
FZPE01000001.1 (genomic DNA)

Frigoribacterium sp Leaf164
KQR44841.1 (1736282)
LMOX01000003.1 (genomic DNA)

Curtobacterium sp B18
BACZ01000054.1_5 (95614)
BACZ01000054.1 ()

Curtobacterium sp BH 2 1 1
AOX66365.1 (1905847)
CP017580.1 (chromosome)

Promicromonosporaceae bacterium W15
DF158877.1_1 (1214064)
DF158877.1 ()

Luteimicrobium subarcticum
PJI90911.1 (620910)
PGTZ01000009.1 (genomic DNA)

Microbacterium aurum
APZ35526.1 (36805)
CP018762.1 (chromosome)

Paraoerskovia marina
SDS04007.1 (545619)
LT629776.1 (genomic DNA)

Cellulomonas sp PhB143
ROS79163.1 (2485186)
RKIJ01000001.1 (genomic DNA)

Sediminihabitans luteus
PJJ68578.1 (1138585)
PGFE01000007.1 (genomic DNA)

Oerskovia sp Root22
KRC33020.1 (1736494)
LMIL01000011.1 (genomic DNA)

Sanguibacter keddieii DSM 10542
ACZ20306.1 (446469)
CP001819.1 (chromosome)

Cellulosimicrobium cellulans
LWGL01000026.1_23 (1710)
LWGL01000026.1 ()

Promicromonosporaceae bacterium CFH 30434
QXTH01000004.1_117 (2304675)
QXTH01000004.1 ()

Isoptericola variabilis 225
AEG43024.1 (743718)
CP002810.1 (chromosome)

Krasilnikoviella flava
SKC80949.1 (526729)
FUZQ01000008.1 (genomic DNA)

Sanguibacter marinus
SFF17553.1 (285351)
FONZ01000003.1 (genomic DNA)

Nakamurella panacisegetis
SDP39966.1 (1090615)
LT629710.1 (genomic DNA)

Nakamurella silvestris
RIJ70998.1 (1645681)
QXCQ01000185.1 (genomic DNA)

Nakamurella sp 12Sc4 1
QCXS01000002.1_822 (2164074)
QCXS01000002.1 ()

Nakamurella sp s14 144
AZI58025.1 (1902245)
CP034170.1 (chromosome)

Actinomycetales bacterium JB111
SJM55691.1 (1434822)
FUHX01000041.1 (genomic DNA)

Intrasporangium chromatireducens Q5 1
EWT03886.1 (584657)
AWQS01000408.1 (genomic DNA)

Calidifontibacter indicus
REF32081.1 (419650)
QTUA01000001.1 (genomic DNA)

Anticoccus suffuscus
PRZ44388.1 (1629062)
PVUE01000001.1 (genomic DNA)

Ruania albidiflava DSM 18029
ATWL01000012.1_79 (1123065)
ATWL01000012.1 ()

Actinotalea ferrariae CF5 4
EYR65255.1 (948458)
AXCW01000001.1 (genomic DNA)

Aeromicrobium sp 592
AWB93673.1 (2079793)
CP026952.1 (chromosome)

Aeromicrobium sp A1 2
AXT83781.1 (2107713)
CP027482.1 (chromosome)

Aeromicrobium marinum DSM 15272
EFQ82200.1 (585531)
CM001024.1 (chromosome)

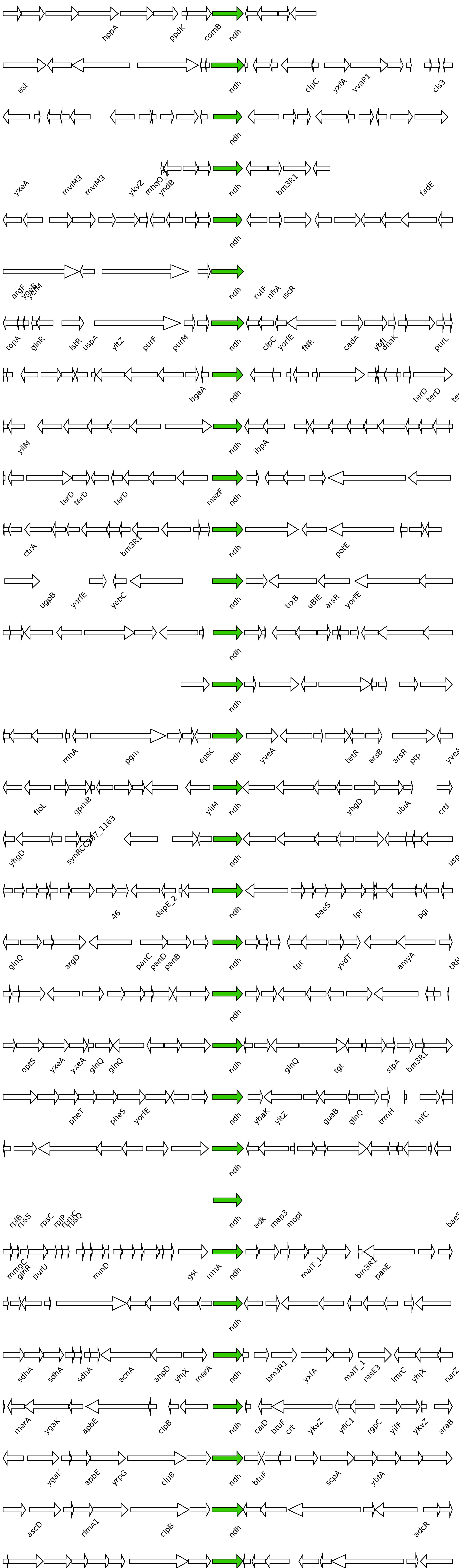

Aeromicrobium sp CPCC 204604
QLVF01000493.1_3 (2233852)
QLVF01000493.1 ()

Aeromicrobium sp Root495
KQY60826.1 (1736550)
LMFJ01000001.1 (genomic DNA)

actinobacterium LLX17
KI301992.1_2960 (1229203)
KI301992.1 ()

Aeromicrobium erythreum
ALX03420.1 (2041)
CP011502.1 (chromosome)

Aeromicrobium choanae
SKB07848.1 (1736691)
LT796768.1 (genomic DNA)

Aeromicrobium camelliae
RQN09651.1 (1538144)
RQJX01000002.1 (genomic DNA)

Aeromicrobium sp PE09 221
OUZ12895.1 (1898043)
MIJB01000001.1 (genomic DNA)

Ruaniaceae bacterium KH17
SNU01661.1 (1945888)
FZQV01000014.1 (genomic DNA)

Actinomycetaceae bacterium sk1b4
PWF27357.1 (2175228)
QETB01000001.1 (genomic DNA)

Nakamurella sp s14 144
AZI58804.1 (1902245)
CP034170.1 (chromosome)

Flaviflexus sp H23T48
AZQ76749.1 (2496867)
CP034593.1 (chromosome)

Flaviflexus salsibiostraticola
AZN29526.1 (1282737)
CP034438.1 (chromosome)

Actinomycetaceae bacterium Marseille P6182
UWPE01000001.1_443 (2364794)
UWPE01000001.1 ()

Actinobaculum suis
KMY23589.1 (1657)
LFUS01000018.1 (genomic DNA)

Actinobaculum massiliense ACS 171 V Col2
EKU94535.1 (883066)
JH992956.1 (genomic)

Actinotignum urinale
PMC89956.1 (190146)
PNHX01000006.1 (genomic DNA)

Actinotignum schaalii
AIE82363.1 (59505)
CP008802.1 (chromosome)

Anaerolineales bacterium
PWB54655.1 (2073117)
PQAL01000013.1 (genomic DNA)

Candidatus Methanoperedens nitroreducens
KCZ70380.1 (1392998)
JMIY01000008.1 (genomic DNA)

filamentous cyanobacterium ESFC 1
KB904821.1_2785 (1128427)
KB904821.1 ()

bacterium HR16
GBC96274.1 (2035411)
BEHS01000115.1 (genomic DNA)

Marinithermus hydrothermalis DSM 14884
AEB11325.1 (869210)
CP002630.1 (chromosome)

Meiothermus luteus
RIH85292.1 (2026184)
QWKZ01000047.1 (genomic DNA)

Meiothermus ruber DSM 1279
ADD27557.1 (504728)
CP001743.1 (chromosome)

Meiothermus chliarophilus DSM 9957
KE387027.1_844 (926560)
KE387027.1 ()

Meiothermus granaticius NBRC 107808
RIH93742.1 (1227551)
QWLB01000003.1 (genomic DNA)

Truepera radiovictrix DSM 17093
ADI15861.1 (649638)
CP002049.1 (chromosome)

Motilibacter peucedani
RKS74072.1 (598650)
RBWV01000012.1 (genomic DNA)

Bradymonadaceae bacterium TMQ3
RDV38596.1 (2283320)
QRGZ01000002.1 (genomic DNA)

Armatimonadetes bacterium OLB18
KXK10629.1 (1617424)
JZQX01000207.1 (genomic DNA)

Ignavibacteria bacterium UBA2403

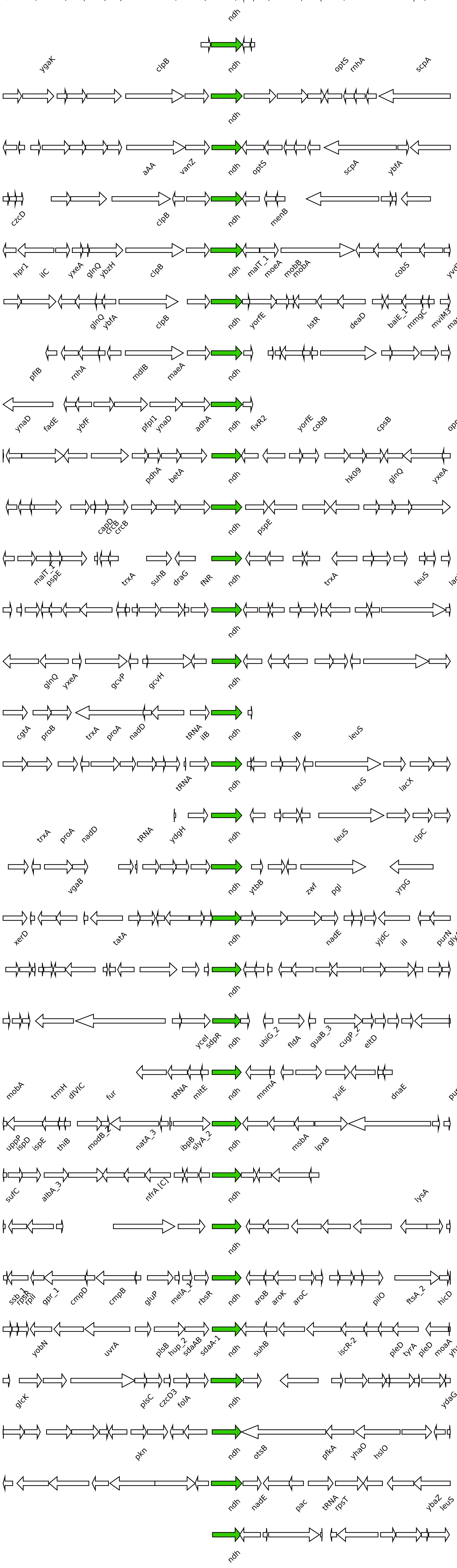

Ignavibacteria bacterium UBA2403
DDRZ01000111.1_10 (1948672)
DDRZ01000111.1 ()

Ignavibacteria bacterium RIFOXYA2_FULL_35_10
OGU91314.1 (1798441)
MHAN01000113.1 (genomic DNA)

Ignavibacteriae bacterium HGW Ignavibacteriae 1
PKL84927.1 (2013808)
PGYU01000019.1 (genomic DNA)

Ignavibacteriales bacterium UTCHB3
OQY76145.1 (1961106)
MWSU01000120.1 (genomic DNA)

Balneolaceae bacterium UBA6773
DKGJ01000033.1_42 (1951518)
DKGJ01000033.1 ()

Balneola vulgaris DSM 17893
AQXH01000004.1_140 (1121104)
AQXH01000004.1 ()

Gracilimonas sp 8A47
PWN05805.1 (2079485)
QGGB01000008.1 (genomic DNA)

Balneolaceae bacterium SMO_bin1
NNSL01000404.1_8 (2021605)
NNSL01000404.1 ()

Aliifodinibius roseus
SHG49643.1 (1194090)
FQUS01000030.1 (genomic DNA)

Aliifodinibius sp WN023
PAU95226.1 (2032627)
NSKE01000002.1 (genomic DNA)

Aliifodinibius roseus
SHF76647.1 (1194090)
FQUS01000013.1 (genomic DNA)

Candidatus Cyclonatronum proteinivorum
AXI99389.1 (1457365)
CP027806.1 (chromosome)

Ignavibacterium album JCM 16511
AFH49472.1 (945713)
CP003418.1 (chromosome)

Ignavibacteria bacterium GWC2_35_8
OGU50634.1 (1798426)
MGZY01000206.1 (genomic DNA)

Ignavibacteriae bacterium HGW Ignavibacteriae 2
PKL90091.1 (2013809)
PGYT01000003.1 (genomic DNA)

Ignavibacteriae bacterium
MAT56809.1 (2026749)
PAAX01000007.1 (genomic DNA)

bacterium BRH_c32
KUO59033.1 (1734400)
LOEU01000037.1 (genomic DNA)

Ignavibacteriae bacterium HGW Ignavibacteriae 3
PKL82931.1 (2013810)
PGYS01000048.1 (genomic DNA)

Ignavibacteria bacterium UBA7675
DLHF01000026.1_21 (1948687)
DLHF01000026.1 ()

Chlamydiales bacterium SCGC AG 110 P3
SCA63329.1 (1871323)
FLYP01000016.1 (genomic DNA)

Waddlia chondrophila WSU 86 1044
ADI37802.1 (716544)
CP001928.1 (chromosome)

Criblamydia sequanensis CRIB 18
CDR34592.1 (1437425)
CCEJ010000008.1 (genomic DNA)

Chlamydia sp 32 24
OJU80227.1 (1895742)
MKSG01000084.1 (genomic DNA)

Chlamydiae bacterium
PWU13546.1 (2081524)
PSRO01000072.1 (genomic DNA)

Chlamydiae bacterium Ga0074140
LNES01000006.1_164 (1752208)
LNES01000006.1 ()

Simkania negevensis Z
CCB88439.1 (331113)
FR872582.1 (genomic DNA)

Chlamydiae bacterium CG10_big_fil_rev_8_21_14_0_10_35_9
PIS00599.1 (1973935)
PFAC01000045.1 (genomic DNA)

Verrucomicrobia bacterium RIFCSPHIGHO2_12_FULL_41_10
OHE76259.1 (1802433)
MIDR01000140.1 (genomic DNA)

Chlamydiae bacterium RIFCSPLOWO2_12_FULL_45_20
OGN69488.1 (1797610)
MGMD01000021.1 (genomic DNA)

Chlamydiae bacterium CG10_big_fil_rev_8_21_14_0_10_42_34
PIS02477.1 (1973936)
PFAD01000015.1 (genomic DNA)

Parachlamydiales bacterium
PLXF01000033.1_1 (2052178)
PLXF01000033.1 ()

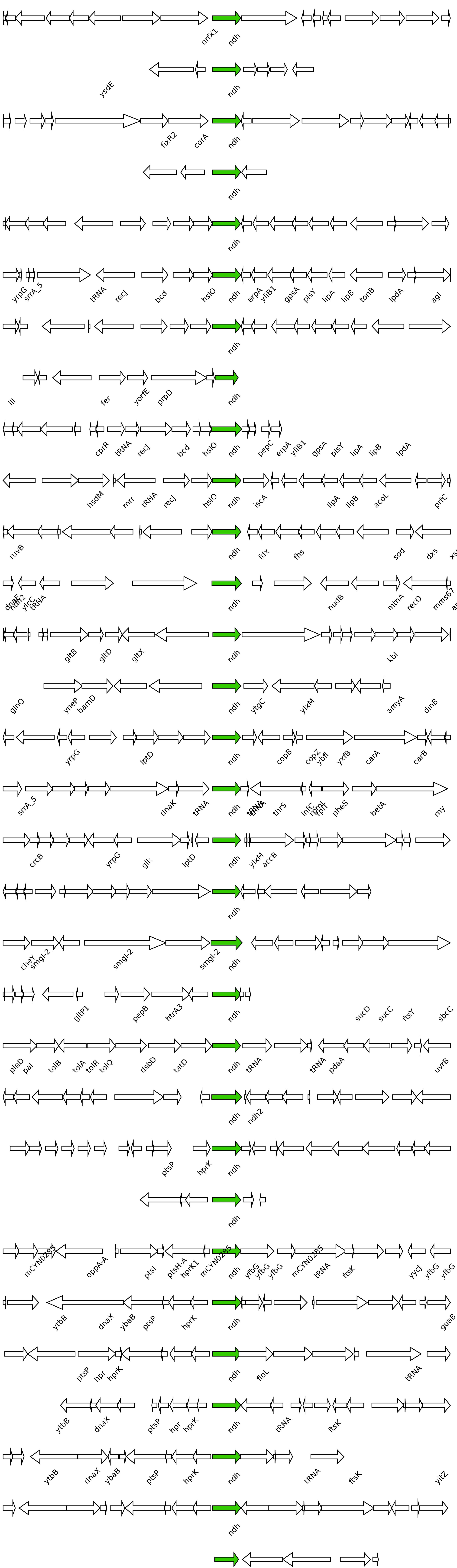

Candidatus Aerophobetes bacterium
PCI78028.1 (2030807)
NVUK01000009.1 (genomic DNA)

Chlamydiales bacterium 38 26
OJV09280.1 (1895743)
MKSK01000003.1 (genomic DNA)

Neochlamydia sp TUME1
KIC75858.1 (1478174)
JRXI01000111.1 (genomic DNA)

Candidatus Protochlamydia amoebophila UWE25
CAF24533.1 (264201)
BX908798.1 (genomic DNA)

Parachlamydiaceae bacterium HS T3
BBPT01000016.1_37 (1314958)
BBPT01000016.1 ()

Rhodohalobacter sp SW132
REL24645.1 (2293433)
QUOK01000011.1 (genomic DNA)

Balneolia bacterium
PUKH01000070.1_80 (2094027)
PUKH01000070.1 ()

Bacteriovorax sp Seq25_V
EQC47124.1 (1201288)
AUNI01000009.1 (genomic DNA)

Halobacteriovorax marinus SJ
CBW26818.1 (862908)
FQ312005.1 (genomic DNA)

Bacteriovorax sp BSW11_IV
EQC48625.1 (1353529)
AUNE01000025.1 (genomic DNA)

Gammaproteobacteria bacterium 39 13
OJV91154.1 (1895767)
MKTN01000034.1 (genomic DNA)

Candidatus Berkiella aquae
KRG20881.1 (295108)
LKAJ01000008.1 (genomic DNA)

Legionella massiliensis
CDZ77568.1 (1034943)
CCSB01000002.1 (genomic DNA)

Ignavibacteria bacterium UBA2333
DDUR01000095.1_35 (1948667)
DDUR01000095.1 ()

Ignavibacteria bacterium UBA5521
DIOF01000120.1_10 (1948677)
DIOF01000120.1 ()

Candidatus Kapabacteria thiocyanatum
OJX56273.1 (1895771)
MKVH01000025.1 (genomic DNA)

Bacteroidetes Chlorobi group bacterium Naka2016
ROL56761.1 (2382229)
RCNP01000005.1 (genomic DNA)

Bacteroidetes Chlorobi group bacterium MS B_bin 24
ROL59673.1 (2382228)
RCNO01000013.1 (genomic DNA)

Nitrospira moscoviensis
ALA58129.1 (42253)
CP011801.1 (chromosome)

Nitrospira japonica
SLM46505.1 (1325564)
LT828648.1 (pure strain)

Chlorobi bacterium
RMF32961.1 (2268192)
RFIK01000209.1 (genomic DNA)

bacterium HR36
GBD37085.1 (2035431)
BEIM01000270.1 (genomic DNA)

Turneriella parva DSM 21527
AFM10793.1 (869212)
CP002959.1 (chromosome)

Halobacteriovorax marinus
OUR99613.1 (97084)
MAAO01000002.1 (genomic DNA)

Bacteriovorax sp MedPE SWde
OIQ19043.1 (1860085)
MPCU01000004.1 (genomic DNA)

Bacteriovorax sp DB6_IX
EQC47662.1 (1353530)
AUNJ01000465.1 (genomic DNA)

Halobacteriovorax sp
MAE57796.1 (2020862)
NZAF01000038.1 (genomic DNA)

Candidatus Berkiella cookevillensis
KRG18253.1 (437022)
LKHV01000008.1 (genomic DNA)

OCS155 cluster bacterium AG 422 J23
QCMT01000002.1_35 (2162608)
QCMT01000002.1 ()

OCS155 cluster bacterium AG 349 E07
QCCZ01000001.1_248 (2162613)
QCCZ01000001.1 ()

Candidatus Lambdaproteobacteria bacterium

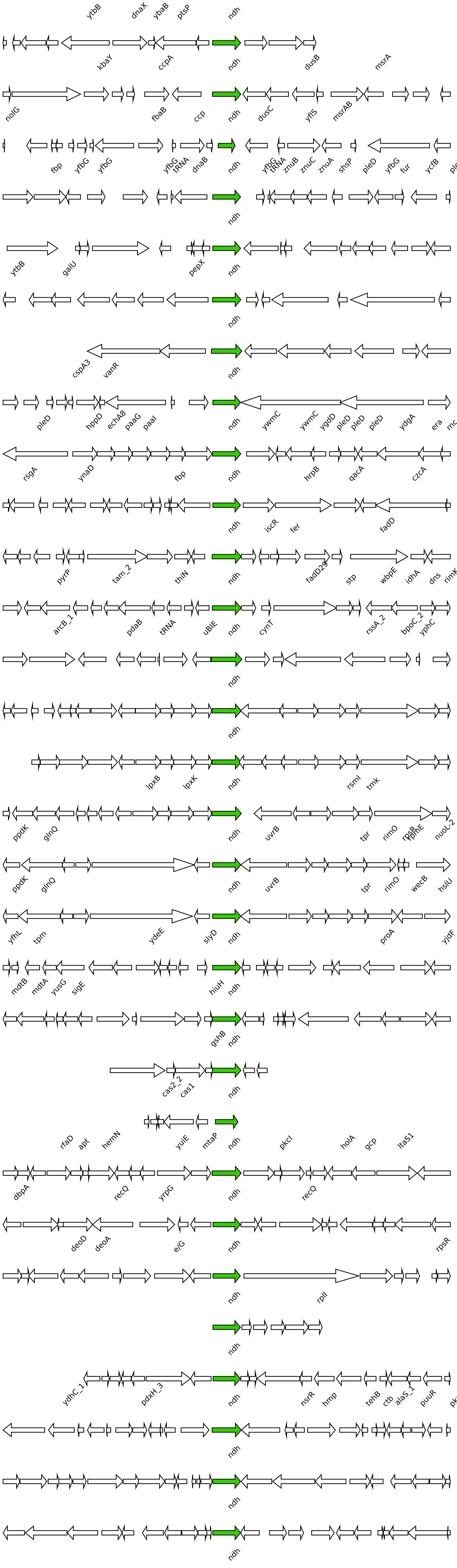

[illegible]
PDZZ01000210.1_1 (2035771)
PDZZ01000210.1 ()

Desulfobacteraceae bacterium
PLX52070.1 (2049433)
PKTV01000322.1 (genomic DNA)

SAR324 cluster bacterium
PCI26790.1 (2024889)
NVSR01000084.1 (genomic DNA)

Candidatus Marinimicrobia bacterium CG1_02_48_14
OIO56721.1 (1805240)
MNWS01000263.1 (genomic DNA)

Desulfobulbaceae bacterium UBA2273
DDWZ01000014.1_38 (1961547)
DDWZ01000014.1 ()

Desulfobulbaceae bacterium
PLX49451.1 (2053307)
PKTX01000034.1 (genomic DNA)

Geovibrio sp L21 Ace BES
ATWF01000001.1_860 (1304888)
ATWF01000001.1 ()

Chlorobium limicola DSM 245
ACD89815.1 (290315)
CP001097.1 (chromosome)

Chlorobium phaeobacteroides DSM 266
ABL65114.1 (290317)
CP000492.1 (chromosome)

Prosthecochloris sp CIB 2401
ANT65496.1 (1868325)
CP016432.1 (chromosome)

Prosthecochloris aestuarii DSM 271
ACF45651.1 (290512)
CP001108.1 (chromosome)

Prosthecochloris sp ZM_2
RNA64470.1 (2045206)
PDNY02000071.1 (genomic DNA)

Prosthecochloris sp HL 130 GSB
ARM30450.1 (1974213)
CP020873.1 (chromosome)

Prosthecochloris sp V1
PWW82198.1 (2017681)
PDNZ01000004.1 (genomic DNA)

Chlorobium phaeobacteroides BS1
ACE03598.1 (331678)
CP001101.1 (chromosome)

Prosthecochloris sp GSB1
ASQ90983.1 (281093)
CP022571.1 (chromosome)

Chlorobium limicola DSM 245
ACD90987.1 (290315)
CP001097.1 (chromosome)

Chlorobium phaeobacteroides DSM 266
ABL64667.1 (290317)
CP000492.1 (chromosome)

Pelodictyon phaeoclathratiforme BU 1
ACF43032.1 (324925)
CP001110.1 (chromosome)

Chlorobium ferrooxidans DSM 13031
EAT59675.1 (377431)
AASE01000002.1 (genomic DNA)

Chlorobium chlorochromatii CaD3
ABB27564.1 (340177)
CP000108.1 (chromosome)

Pelodictyon luteolum DSM 273
ABB24436.1 (319225)
CP000096.1 (chromosome)

Chlorobium phaeovibrioides DSM 265
ABP37384.1 (290318)
CP000607.1 (chromosome)

Chlorobaculum tepidum TLS
AAM71615.1 (194439)
AE006470.1 (chromosome)

Desulforegula conservatrix Mb1Pa
AUEY01000014.1_5 (1304885)
AUEY01000014.1 ()

Opitutae bacterium UBA1333
DBSV01000008.1_52 (1948706)
DBSV01000008.1 ()

Opitutae bacterium UBA3151
DEYT01000025.1_2 (1948718)
DEYT01000025.1 ()

Candidatus Lambdaproteobacteria bacterium RIFOXYD2_FULL_56_26
OGH03409.1 (1817773)
MFNF01000017.1 (genomic DNA)

Bdellovibrio exovorus JSS
AGH95992.1 (1184267)
CP003537.1 (chromosome)

Bdellovibrio sp CG10_big_fil_rev_8_21_14_0_10_47_8
PIS11977.1 (1975526)
PEZR01000015.1 (genomic DNA)

Bdellovibrio sp UBA4095
DFXZ01000004.1_894 (1961378)
DFXZ01000004.1 ()

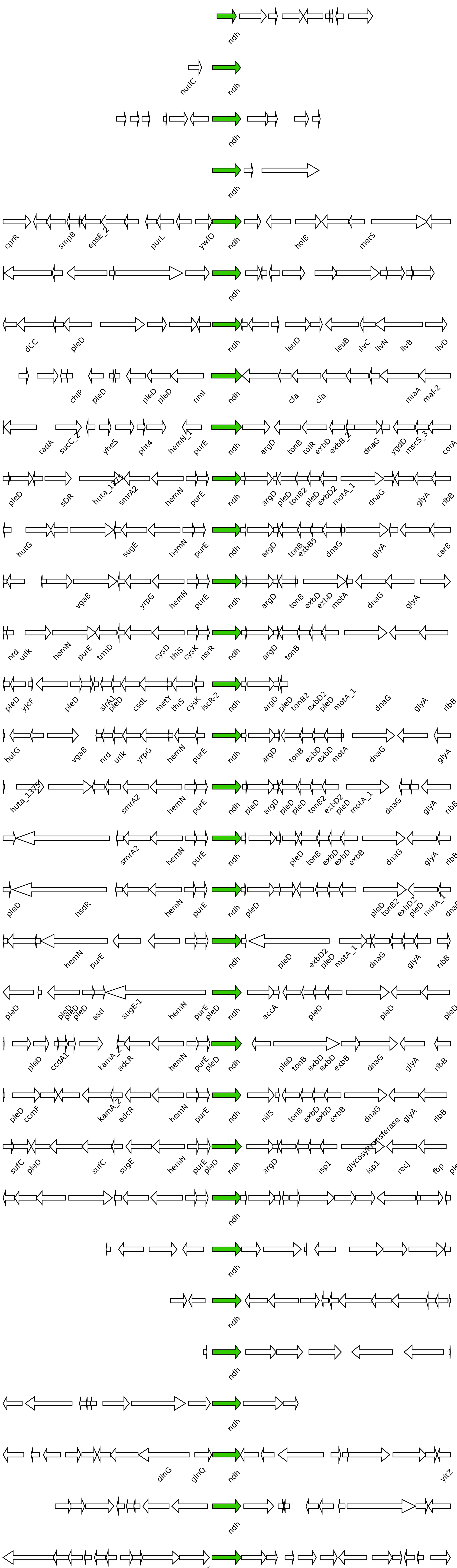

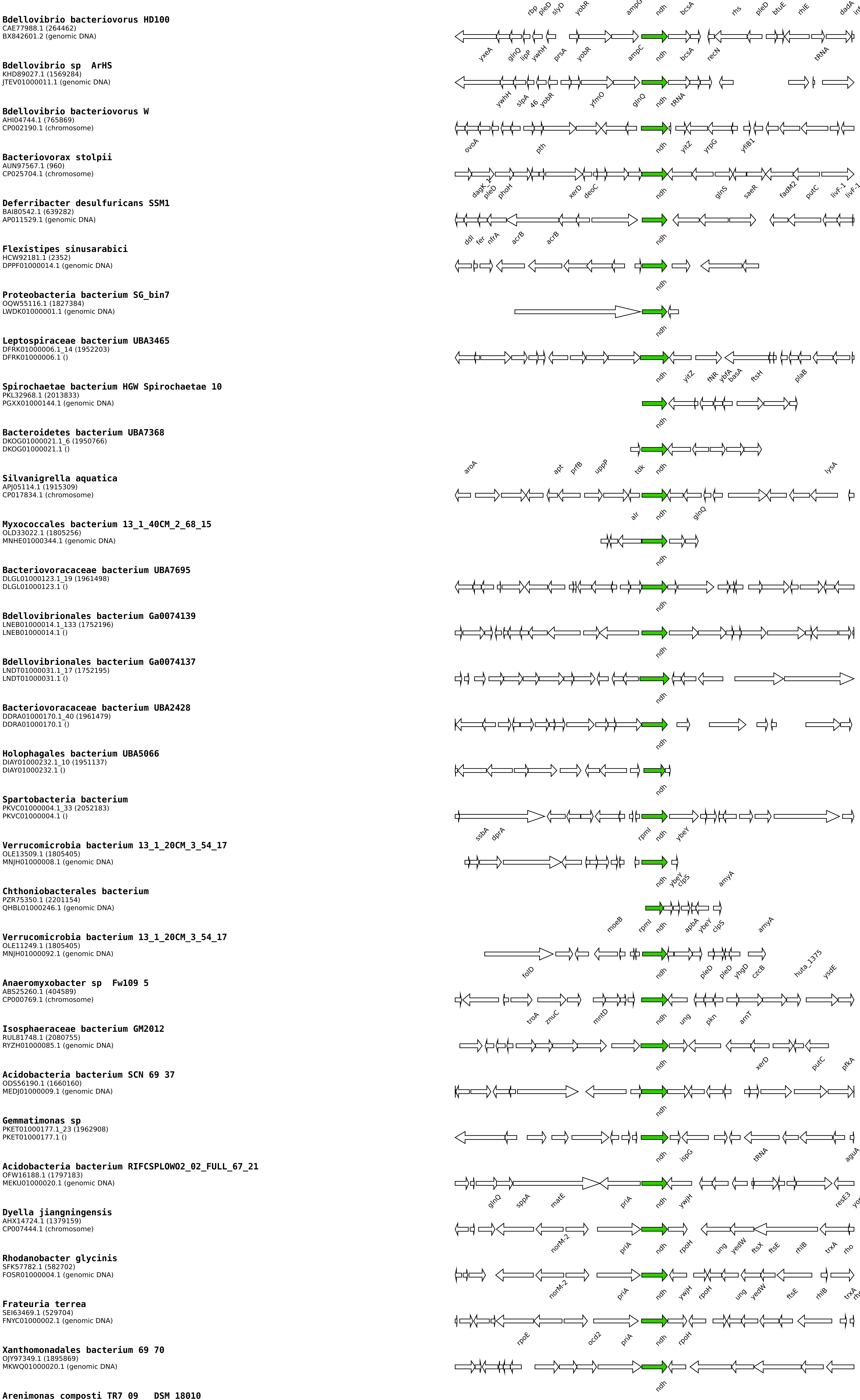
Bdellovibrio bacteriovorus HD100
CAE77988.1 (264462)
BX842601.2 (genomic DNA)
Bdellovibrio sp ArHS
KHD89027.1 (1569284)
JTEV01000011.1 (genomic DNA)
Bdellovibrio bacteriovorus W
AHI04744.1 (765869)
CP002190.1 (chromosome)
Bacteriovorax stolpii
AUN97567.1 (960)
CP025704.1 (chromosome)
Deferribacter desulfuricans SSM1
BAI80542.1 (639282)
AP011529.1 (genomic DNA)
Flexistipes sinusarabici
HCW92181.1 (2352)
DPPF01000014.1 (genomic DNA)
Proteobacteria bacterium SG_bin7
OQW55116.1 (1827384)
LWDK01000001.1 (genomic DNA)
Leptospiraceae bacterium UBA3465
DFRK01000006.1_14 (1952203)
DFRK01000006.1 ()
Spirochaetae bacterium HGW Spirochaetae 10
PKL32968.1 (2013833)
PGXX01000144.1 (genomic DNA)
Bacteroidetes bacterium UBA7368
DKOG01000021.1_6 (1950766)
DKOG01000021.1 ()
Silvanigrella aquatica
APJ05114.1 (1915309)
CP017834.1 (chromosome)
Myxococcales bacterium 13_1_40CM_2_68_15
OLD33022.1 (1805256)
MNHE01000344.1 (genomic DNA)
Bacteriovoracaceae bacterium UBA7695
DLGL01000123.1_19 (1961498)
DLGL01000123.1 ()
Bdellovibrionales bacterium Ga0074139
LNEB01000014.1_133 (1752196)
LNEB01000014.1 ()
Bdellovibrionales bacterium Ga0074137
LNDT01000031.1_17 (1752195)
LNDT01000031.1 ()
Bacteriovoracaceae bacterium UBA2428
DDRA01000170.1_40 (1961479)
DDRA01000170.1 ()
Holophagales bacterium UBA5066
DIAY01000232.1_10 (1951137)
DIAY01000232.1 ()
Spartobacteria bacterium
PKVC01000004.1_33 (2052183)
PKVC01000004.1 ()
Verrucomicrobia bacterium 13_1_20CM_3_54_17
OLE13509.1 (1805405)
MNJH01000008.1 (genomic DNA)
Chthoniobacterales bacterium
PZR75350.1 (2201154)
QHBL01000246.1 (genomic DNA)
Verrucomicrobia bacterium 13_1_20CM_3_54_17
OLE11249.1 (1805405)
MNJH01000092.1 (genomic DNA)
Anaeromyxobacter sp Fw109 5
ABS25260.1 (404589)
CP000769.1 (chromosome)
Isosphaeraceae bacterium GM2012
RUL81748.1 (2080755)
RYZH01000085.1 (genomic DNA)
Acidobacteria bacterium SCN 69 37
ODS56190.1 (1660160)
MEDJ01000009.1 (genomic DNA)
Gemmatimonas sp
PKET01000177.1_23 (1962908)
PKET01000177.1 ()
Acidobacteria bacterium RIFCSPLOWO2_02_FULL_67_21
OFW16188.1 (1797183)
MEKU01000020.1 (genomic DNA)
Dyella jiangningensis
AHX14724.1 (1379159)
CP007444.1 (chromosome)
Rhodanobacter glycinis
SFK57782.1 (582702)
FOSR01000004.1 (genomic DNA)
Frateuria terrea
SEI63469.1 (529704)
FNYC01000002.1 (genomic DNA)
Xanthomonadales bacterium 69 70
OJY97349.1 (1895869)
MKWQ01000020.1 (genomic DNA)
Arenimonas composti TR7 09 DSM 18010

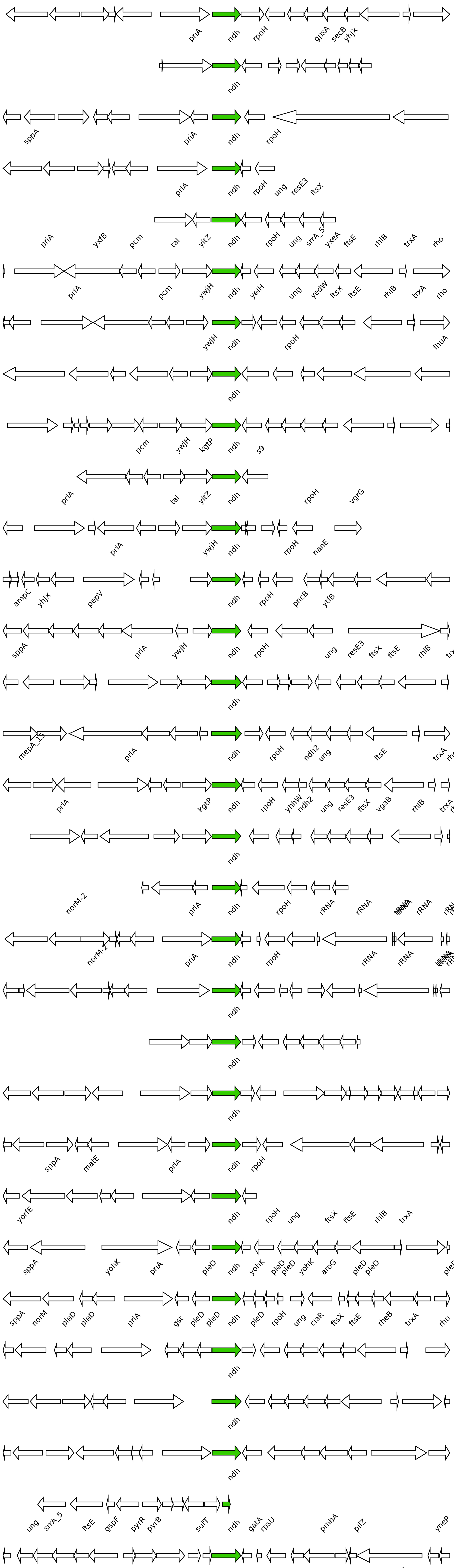

AUFF01000007.1_39 (1121013)
AUFF01000007.1 ()

Lysobacter dokdonensis DS 58
KGQ17833.1 (1300345)
JRKJ01000024.1 (genomic DNA)

Luteimonas mephitis DSM 12574
KE383816.1_606 (1122183)
KE383816.1 ()

Lysobacter sp WF 2
RFP59081.1 (2303539)
QVPD01000015.1 (genomic DNA)

Lysobacter arseniciresistens ZS79
KGM53182.1 (913325)
AVPT01000052.1 (genomic DNA)

Lysobacter soli
RDY66768.1 (453783)
QTJR01000007.1 (genomic DNA)

Vulcaniibacterium tengchongense
RPE79851.1 (1273429)
RKQN01000002.1 (genomic DNA)

Lysobacter sp Root559
KQZ56976.1 (1736559)
LMGE01000015.1 (genomic DNA)

Lysobacter sp C8 1
KZ987400.1_373 (2315424)
KZ987400.1 ()

Xanthomonadales bacterium RIFOXYD1_FULL_69_11
OHE86142.1 (1802657)
MIDW01000140.1 (genomic DNA)

Lysobacter sp ZS60
RNF84519.1 (1327343)
RIBS01000003.1 (genomic DNA)

Lysobacter sp Root690
KRB11516.1 (1736588)
LMHM01000001.1 (genomic DNA)

Lysobacter maris
AWV06084.1 (1605891)
CP029843.1 (chromosome)

Lysobacter sp Root494
KQY54842.1 (1736549)
LMFH01000001.1 (genomic DNA)

Luteimonas abyssi
KQ759763.1_72 (1247514)
KQ759763.1 ()

Pseudoxanthomonas suwonensis 11 1
ADV28594.1 (743721)
CP002446.1 (chromosome)

Pseudoxanthomonas suwonensis
AKC86950.1 (314722)
CP011144.1 (chromosome)

Pseudoxanthomonas mexicana
LSIF01000014.1_4 (128785)
LSIF01000014.1 ()

Pseudoxanthomonas wuyuanensis
SOD54829.1 (1073196)
OCND01000005.1 (genomic DNA)

Pseudoxanthomonas sp CF125
SDR07394.1 (1855303)
FNLD01000003.1 (genomic DNA)

Xanthomonas translucens
LNSU01000173.1_8 (343)
LNSU01000173.1 ()

Thermomonas fusca DSM 15424
AUIV01000030.1_17 (1123377)
AUIV01000030.1 ()

Luteimonas sp J29
AWZR01000002.1_364 (935863)
AWZR01000002.1 ()

Stenotrophomonas ginsengisoli
KRG76985.1 (336566)
LDJM01000020.1 (genomic DNA)

Stenotrophomonas acidaminiphila
ALJ26584.1 (128780)
CP012900.1 (chromosome)

Stenotrophomonas maltophilia K279a
CAQ47588.1 (522373)
AM743169.1 (genomic DNA)

Xanthomonas campestris pv campestris str ATCC 33913
AAM43026.1 (190485)
AE008922.1 (chromosome)

Xanthomonas massiliensis
FCOY01000004.1_68 (1720302)
FCOY01000004.1 ()

Rudaea cellulosilytica DSM 22992
KB899241.1_1186 (1123073)
KB899241.1 ()

Rhodanobacteraceae bacterium UBA703
DBMH01000120.1_1 (1953068)
DBMH01000120.1 ()

Ahniella affigens
AVP99677.1 (2021234)
CP027860.1 (chromosome)

Xanthomonadales bacterium 66 474
OJY82841.1 (1895868)
MKWP01000015.1 (genomic DNA)

Planctomycetes bacterium UBA2386
DDSQ01000195.1_3 (1948127)
DDSQ01000195.1 ()

Candidatus Rokubacteria bacterium 13_2_20CM_69_15_1
OLB07146.1 (1805372)
MNCR01000048.1 (genomic DNA)

Bacteroidetes bacterium CG12_big_fil_rev_8_21_14_0_65_60_17
PIQ62688.1 (1973924)
PCUY01000040.1 (genomic DNA)

Anaeromyxobacter dehalogenans 2CP C
ABC81923.1 (290397)
CP000251.1 (chromosome)

Anaeromyxobacter sp Fw109 5
ABS25829.1 (404589)
CP000769.1 (chromosome)

Acidobacteria bacterium Mor1
ANM30157.1 (1660251)
CP011806.1 (chromosome)

Gemmatimonadetes bacterium SCN 70 22
ODT04113.1 (1660163)
MEDZ01000009.1 (genomic DNA)

Gemmatimonadales bacterium UBA4718
DHJO01000089.1_2 (1951117)
DHJO01000089.1 ()

Gemmatimonadales bacterium UBA4720
DHJM01000002.1_9 (1951118)
DHJM01000002.1 ()

Gemmatimonadetes bacterium 2013_60CM_65_52
NGFI01000013.1_191 (1978118)
NGFI01000013.1 ()

Gemmatimonadetes bacterium 21 71 4
OYV74426.1 (1970514)
NCBA01000007.1 (genomic DNA)

Gemmatimonas aurantiaca T 27
BAH37437.1 (379066)
AP009153.1 (genomic DNA)

Gemmatimonas sp UBA7669
DLHL01000053.1_40 (1946568)
DLHL01000053.1 ()

Gemmatimonas phototrophica
AMW06587.1 (1379270)
CP011454.1 (chromosome)

Acidimicrobiia bacterium UBA4744
DHIO01000022.1_78 (1948319)
DHIO01000022.1 ()

Mitsuaria sp 7
ANH69520.1 (1658665)
CP011514.1 (chromosome)

Herbaspirillum seropedicae
AKN65312.1 (964)
CP011930.1 (chromosome)

Schlegelella thermodepolymerans
PPE69262.1 (215580)
PSNY01000014.1 (genomic DNA)

Methylibium petroleiphilum PM1
ABM95172.1 (420662)
CP000555.1 (chromosome)

Burkholderiales bacterium UBA4200
DFTY01000145.1_6 (1950777)
DFTY01000145.1 ()

Aquabacterium sp SJQ9
RRS03052.1 (2493092)
RSED01000015.1 (genomic DNA)

Burkholderiales bacterium RIFCSPLOWO2_12_FULL_64_99
OGB60898.1 (1797568)
MESK01000110.1 (genomic DNA)

Burkholderiales bacterium RIFCSPHIGHO2_01_FULL_63_240
OGA84746.1 (1797552)
MERU01000002.1 (genomic DNA)

Aquabacterium sp UBA666
DBNS01000012.1_23 (1946043)
DBNS01000012.1 ()

Aquabacterium olei
AWI55237.1 (1296669)
CP029210.1 (chromosome)

Rhizobacter sp Root404
KQW36013.1 (1736528)
LMDS01000005.1 (genomic DNA)

Betaproteobacteria bacterium RIFCSPLOWO2_12_FULL_67_28
OGA69252.1 (1797503)
MERO01000074.1 (genomic DNA)

Rhodoferax saidenbachensis
APW43430.1 (1484693)
CP019239.1 (chromosome)

beta proteobacterium AAP99
KPF69997.1 (1523428)
LJIA01000002.1 (genomic DNA)

Ramlibacter tataouinensis TTB310

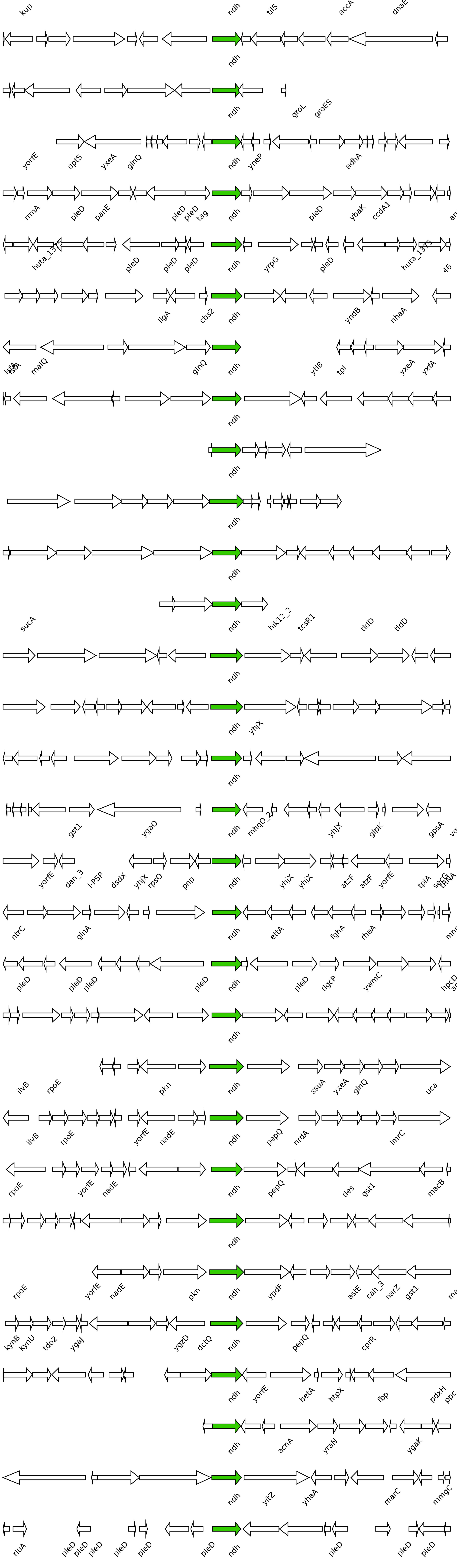

AEG92278.1 (365046)
CP000245.1 (chromosome)

Ramlibacter tataouinensis
AMO24324.1 (94132)
CP010951.1 (chromosome)

Xylophilus sp Leaf220
KQM75779.1 (1735686)
LMKO01000012.1 (genomic DNA)

Pseudacidovorax intermedius
KTT22141.1 (433924)
LDSL01000061.1 (genomic DNA)

Variovorax sp PAMC 28711
AMM23430.1 (1795631)
CP014517.1 (chromosome)

Comamonas sp BIGb0124
ROR23212.1 (2485130)
RJVF01000009.1 (genomic DNA)

Corticibacter populi
RMX02660.1 (1550736)
RDQO01000008.1 (genomic DNA)

Lampropedia hyalina DSM 16112
SHE28573.1 (1122156)
FQUZ01000001.1 (genomic DNA)

Acidobacteria bacterium Ga0077522
LNDN01000242.1_4 (1752183)
LNDN01000242.1 ()

Nitrospira defluvii
CBK39814.1 (330214)
FP929003.1 (genomic DNA)

Chloracidobacterium sp UBA2345
DDUF01000075.1_9 (1946316)
DDUF01000075.1 ()

Acidobacteria bacterium OLB17
KXK02165.1 (1617423)
LLEU01000030.1 (genomic DNA)

Chloracidobacterium sp UBA7659
DLHV01000164.1_20 (1946322)
DLHV01000164.1 ()

Chloracidobacterium sp UBA3360
DEQS01000040.1_8 (1946317)
DEQS01000040.1 ()

Acidobacteria bacterium Ga0077534
LNEI01000018.1_303 (1752184)
LNEI01000018.1 ()

Acidobacteria bacterium Ga0074141
LNDP01000023.1_522 (1752182)
LNDP01000023.1 ()

Bacteriovoracaceae bacterium UBA1018
DBAE01000195.1_3 (1961475)
DBAE01000195.1 ()

Thermogemmatispora tikiterensis
RAQ98246.1 (1825093)
MCIF01000002.1 (genomic DNA)

Ktedonobacter sp
HAE83385.1 (1932716)
DMCL01000338.1 (genomic DNA)

Candidatus Koribacter versatilis Ellin345
ABF41527.1 (204669)
CP000360.1 (chromosome)

Geothrix fermentans DSM 14018
KE386810.1_262 (1121920)
KE386810.1 ()

Holophagaceae bacterium
PMJK01000129.1_1 (2052165)
PMJK01000129.1 ()

bacterium HR15
GBC93594.1 (2035410)
BEHR01000028.1 (genomic DNA)

Armatimonadetes bacterium GBS
LDXZ01000060.1_1 (1661580)
LDXZ01000060.1 ()

candidate division NC10 bacterium CSP1 5
KRT68285.1 (1640516)
LDXR01000024.1 (genomic DNA)

Chloroflexi bacterium SPGG9
LXTD01000048.1_5 (1842519)
LXTD01000048.1 ()

bacterium Candidatus Blackallbacteria CG13_big_fil_rev_8_21_14_2_50_49_14
PIW49423.1 (2014260)
PFGG01000027.1 (genomic DNA)

Cyanobacteria bacterium UBA7694
DLGM01000366.1_40 (1947897)
DLGM01000366.1 ()

Candidatus Thermofonsia Clade 1 bacterium
PJF33798.1 (2364210)
PGTM01000753.1 (genomic DNA)

Candidatus Thermofonsia Clade 1 bacterium
PJF35674.1 (2364210)
PGTM01000123.1 (genomic DNA)

Spirulina major PCC 6313
KV878783.1_3196 (129961)
KV878783.1 ()

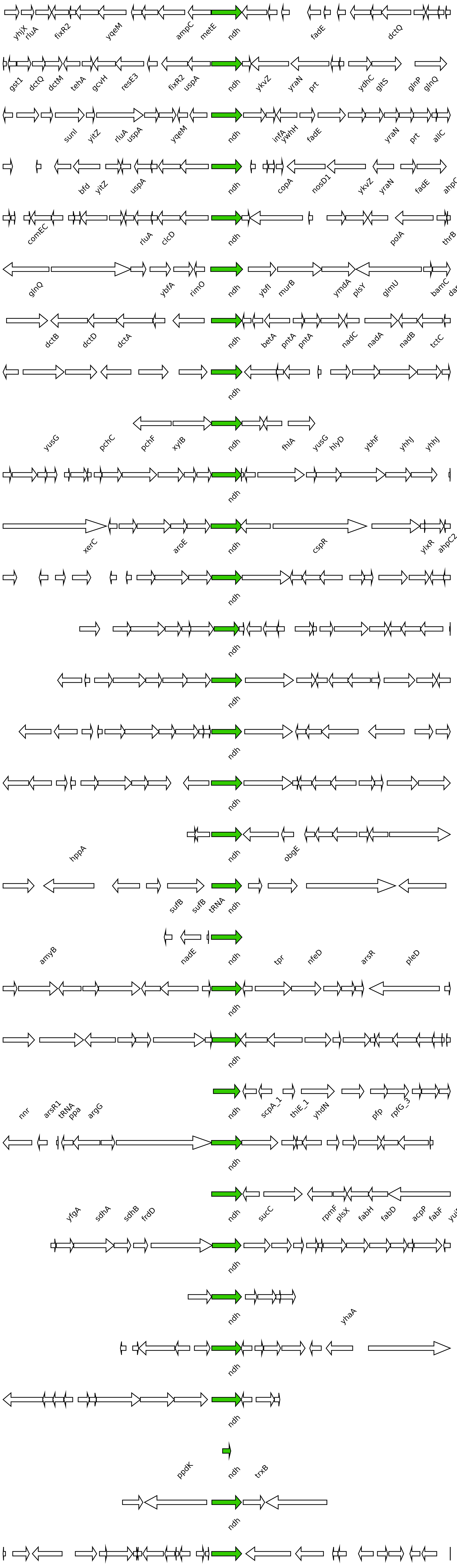

filamentous cyanobacterium ESFC 1
KB904821.1_471 (1128427)
KB904821.1 ()

Synechococcus sp PCC 6312
AFY59507.1 (195253)
CP003558.1 (chromosome)

filamentous cyanobacterium CCP1
PSB65705.1 (2107703)
PVWE01000398.1 (genomic DNA)

Oscillatoriales cyanobacterium JSC 12
EKQ68332.1 (864702)
CM001633.1 (chromosome)

Crinalium epipsammum PCC 9333
AFZ12274.1 (1173022)
CP003620.1 (chromosome)

Leptolyngbya sp
PZV13755.1 (47254)
QBMH01000062.1 (genomic DNA)

Chlorogloea sp CCALA 695
PSB31761.1 (2107693)
PVWN01000019.1 (genomic DNA)

Aliterella atlantica CENA595
KJH73048.1 (1618023)
JYON01000002.1 (genomic DNA)

Chroococcidiopsis sp TS 821
PPS43197.1 (1378066)
MVDI01000003.1 (genomic)

Fischerella sp NIES 3754
BAU06590.1 (1752063)
AP017305.1 (genomic DNA)

Anabaena sp CA ATCC 33047
LUHI01000056.1_144 (52271)
LUHI01000056.1 ()

Anabaena sp CA ATCC 33047
LUHI01000056.1_145 (52271)
LUHI01000056.1 ()

Anabaena sp CA ATCC 33047
LUHI01000040.1_296 (52271)
LUHI01000040.1 ()

Anabaena sp 90
AFW94718.1 (46234)
CP003284.1 (chromosome)

Nostoc azollae 0708
ADI66154.1 (551115)
CP002059.1 (chromosome)

Raphidiopsis curvata NIES 932
BAZ90277.1 (1973482)
AP018317.1 (genomic DNA)

Anabaena sp 90
AFW93832.1 (46234)
CP003284.1 (chromosome)

Richelia sp
HAE05904.1 (2053605)
DLZW01000227.1 (genomic DNA)

Calothrix sp NIES 3974
BAZ06672.1 (2005462)
AP018254.1 (genomic DNA)

Calothrix sp NIES 3974
BAZ04363.1 (2005462)
AP018254.1 (genomic DNA)

Leptolyngbya sp O 77
BAU42440.1 (1080068)
AP017367.1 (genomic DNA)

Cyanobacteria bacterium J055
RMG11989.1 (2420338)
RFHR01000143.1 (genomic DNA)

Synechococcus sp PCC 7002
ACB00289.1 (32049)
CP000951.1 (chromosome)

Stanieria cyanosphaera PCC 7437
AFZ34491.1 (111780)
CP003653.1 (chromosome)

Geitlerinema sp PCC 7407
AFY64538.1 (1173025)
CP003591.1 (chromosome)

Cyanobacteria bacterium UBA6050
DIYQ01000007.1_106 (1947889)
DIYQ01000007.1 ()

Cyanothece sp PCC 8801
ACK67450.1 (41431)
CP001287.1 (chromosome)

Crocosphaera watsonii WH 8502
CCQ50034.1 (423474)
CAQK01000229.1 (genomic DNA)

Snowella sp
PZV22826.1 (2164131)
QBMS01000149.1 (genomic DNA)

Microcystis aeruginosa NIES 2549
AKE63413.1 (1641812)
CP011304.1 (chromosome)

Candidatus Atelocyanobacterium thalassa isolate ALOHA

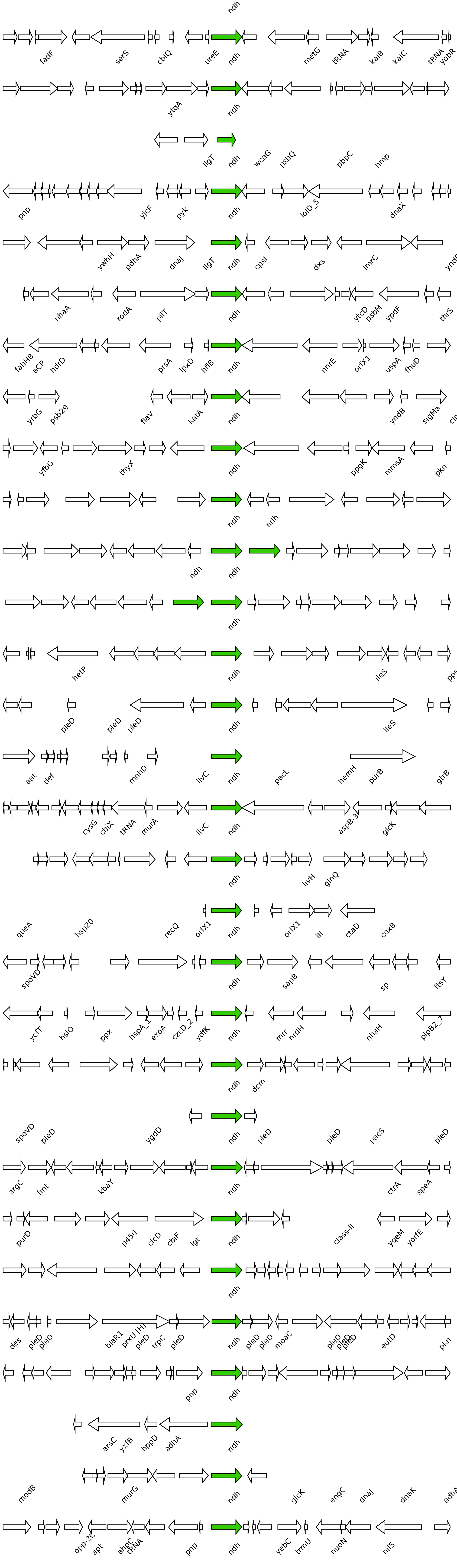

ADB95216.1 (1453429)
CP001842.1 (chromosome)

Synechocystis sp PCC 6714
AIE74868.1 (1147)
CP007542.1 (chromosome)

Aphanothece hegewaldii CCALA 016
PSF37178.1 (2107694)
PXOH01000011.1 (genomic DNA)

Chondrocystis sp NIES 4102
BAZ44351.1 (2005460)
AP018281.1 (genomic DNA)

Desertifilum sp IPPAS B 1220
OEJ74656.1 (1781255)
MJGC01000063.1 (genomic DNA)

Prochlorothrix hollandica PCC 9006 CALU 1027
KB235939.1_140 (317619)
KB235939.1 ()

Cyanobacterium sp HL 69
AUC61690.1 (2054282)
CP024912.1 (chromosome)

Geminocystis herdmanii PCC 6308
CM001775.1_1920 (113355)
CM001775.1 ()

Cyanobacterium aponinum PCC 10605
AFZ52735.1 (755178)
CP003947.1 (chromosome)

Phormidesmis priestleyi Ana
KPQ35465.1 (1666911)
LJZR01000012.1 (genomic DNA)

Cyanobacteria bacterium T3Sed10_344R1
PXDR01000593.1_5 (2099687)
PXDR01000593.1 ()

Pseudanabaena sp ABRG5 3
BBC24983.1 (685565)
AP017560.1 (genomic DNA)

Synechococcus sp PCC 7502
AFY74048.1 (1173263)
CP003594.1 (chromosome)

Leptolyngbya antarctica
PZO40420.1 (268115)
QBMN01000073.1 (genomic DNA)

Cyanobacteria bacterium CSSed10_82m
PWRG01000410.1_6 (2099684)
PWRG01000410.1 ()

Trichodesmium erythraeum IMS101
ABG51303.1 (203124)
CP000393.1 (chromosome)

Raphidiopsis curvata NIES 932
BAZ90052.1 (1973482)
AP018317.1 (genomic DNA)

Candidatus Dadabacteria bacterium RIFCSPHIGHO2_12_FULL_53_21
OGE22140.1 (1797756)
MFCJ01000099.1 (genomic DNA)

Candidatus Dadabacteria bacterium
QIHJ01000391.1_9 (2080303)
QIHJ01000391.1 ()

Candidatus Dadabacteria bacterium UBA2774
DEIM01000127.1_1 (1953209)
DEIM01000127.1 ()

Candidatus Dadabacteria bacterium UBA2774
DEIM01000121.1_1 (1953209)
DEIM01000121.1 ()

Latescibacteria bacterium UBA2998
DDZW01000090.1_12 (1948114)
DDZW01000090.1 ()

Synechococcus elongatus PCC 7942
ABB56133.1 (1140)
CP000100.1 (chromosome)

Deltaproteobacteria bacterium GWA2_45_12
OGP08092.1 (1797805)
MGPH01000052.1 (genomic DNA)

Planctomycetaceae bacterium Ga0077529
LNDY01000425.1_10 (1752218)
LNDY01000425.1 ()

Planctopirus limnophila DSM 3776
ADG66768.1 (521674)
CP001744.1 (chromosome)

Rubinisphaera sp
MBV08314.1 (2024857)
PCCB01000046.1 (genomic DNA)

Rubinisphaera brasiliensis DSM 5305
ADY57684.1 (756272)
CP002546.1 (chromosome)

Stanieria cyanosphaera PCC 7437
AFZ34854.1 (111780)
CP003653.1 (chromosome)

Crocosphaera watsonii WH 8502
CCQ51773.1 (423474)
CAQK01000525.1 (genomic DNA)

cyanobacterium endosymbiont of Epithemia turgida isolate EtSB Lake Yunoko
BAP17956.1 (1228987)
AP012549.1 (genomic DNA)

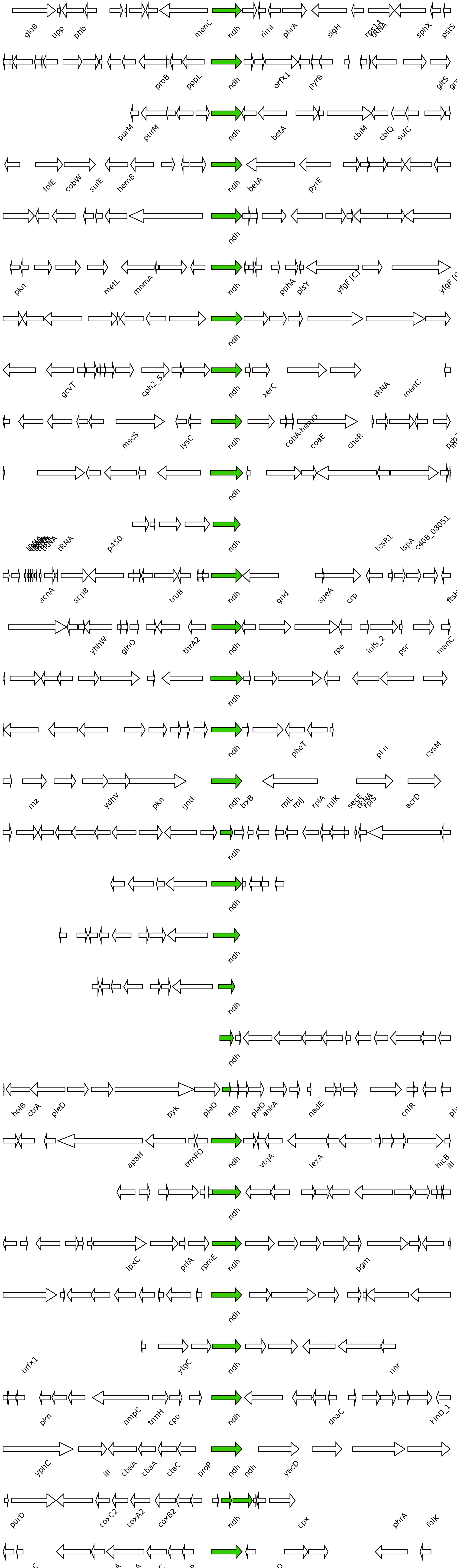

Crocosphaera watsonii WH 8502
CCQ51774.1 (423474)
CAQK01000525.1 (genomic DNA)

Syntrophaceae bacterium UBA4797
DHGN01000150.1_4 (1961436)
DHGN01000150.1 ()

Acidobacteria bacterium 13_2_20CM_2_57_6
OLB85932.1 (1803461)
MNEA01000096.1 (genomic DNA)

Acidobacteria bacterium 13_2_20CM_2_57_6
OLB84256.1 (1803461)
MNEA01000129.1 (genomic DNA)

candidate division GN15 bacterium
PWB73504.1 (2072418)
PQAP01000053.1 (genomic DNA)

Actinobacteria bacterium Ga0077541
LNEQ01000230.1_56 (1752189)
LNEQ01000230.1 ()

Anaerolineales bacterium UBA2796
DEHQ01000045.1_4 (1950201)
DEHQ01000045.1 ()

Rhodopirellula sp TMED283
OUX51003.1 (1986660)
NHMC01000024.1 (genomic DNA)

Vampirovibrio chlorellavorus
LAPX01000013.1_124 (758823)
LAPX01000013.1 ()

Candidatus Promineofilum breve
CUS03820.2 (1806508)
LN890655.2 (genomic DNA)

Deltaproteobacteria bacterium TMED126
RPG78236.1 (1986843)
NHGB02000004.1 (genomic DNA)

Deltaproteobacteria bacterium TMED58
OUU32681.1 (1986674)
NHDL01000021.1 (genomic DNA)

Candidatus Dadabacteria bacterium UBA1144
DCAB01000019.1_94 (1953208)
DCAB01000019.1 ()

Acidisphaera rubrifaciens HS AP3
GAN76380.1 (1231350)
BANB01000088.1 (genomic DNA)

Competibacteraceae bacterium UBA4410
DGQQ01000053.1_7 (1951622)
DGQQ01000053.1 ()

Competibacteraceae bacterium UBA2792
DEHU01000091.1_6 (1951620)
DEHU01000091.1 ()

Candidatus Competibacter denitrificans Run_A_D11
CDI03450.1 (1400863)
CBTJ020000057.1 (genomic DNA)

Competibacteraceae bacterium UBA4410
DGQQ01000051.1_15 (1951622)
DGQQ01000051.1 ()

Competibacteraceae bacterium UBA2788
DEHY01000172.1_2 (1951619)
DEHY01000172.1 ()

Proteobacteria bacterium ST_bin12
OQW70572.1 (1978760)
MSXP01000001.1 (genomic)

Betaproteobacteria bacterium UBA6910
DKBC01000194.1_1 (1948462)
DKBC01000194.1 ()

Acidiferrobacter sp SPIII_3
AWP24023.1 (1281578)
CP027663.1 (chromosome)

Acidithiobacillus ferrooxidans ATCC 23270
ACK80940.1 (243159)
CP001219.1 (chromosome)

Sphingomonas sp YR710
SDB98026.1 (1882773)
FMZJ01000001.1 (genomic DNA)

Methyloferula stellata AR4
ARWA01000001.1_308 (876269)
ARWA01000001.1 ()

Rhodospirillaceae bacterium SYSU D60014
QOZR01000001.1_646 (2268457)
QOZR01000001.1 ()

Sphingomonas sp URHD0007
PVZ17640.1 (1298856)
QEQM01000001.1 (genomic DNA)

Novosphingobium lentum NBRC 107847
BCTW01000007.1_144 (1219033)
BCTW01000007.1 ()

Erythrobacter sp RIFCSPHIGHO2_12_FULL_63_10
OGS51710.1 (1797975)
MGVU01000361.1 (genomic DNA)

Sphingomonadales bacterium BRH_c42
KUO56775.1 (1734407)
LOEX01000024.1 (genomic DNA)

alpha proteobacterium AAP81b
KPF71546.1 (1523432)

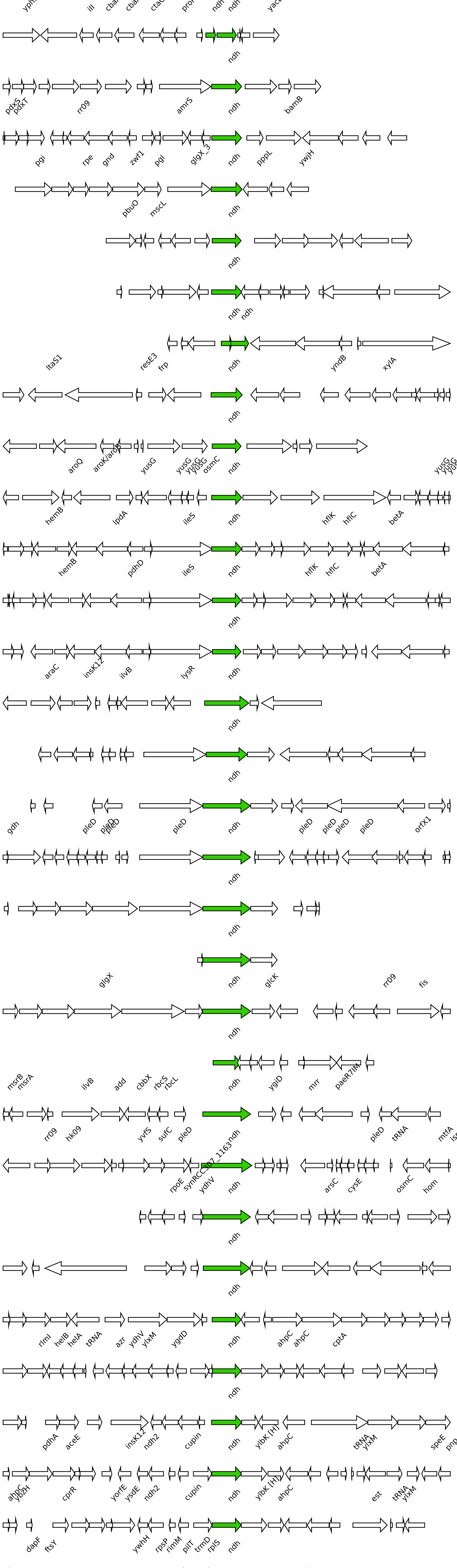

[illegible]
LJHX01000103.1 (genomic DNA)

Proteobacteria bacterium ST_bin13
OQW70958.1 (1978761)
MSXQ01000028.1 (genomic)

Sphingomonas sp Root710
KRB86904.1 (1736594)
LMIB01000001.1 (genomic DNA)

Sphingomonas sp UBA1000
DBAW01000079.1_15 (1947531)
DBAW01000079.1 ()

Sphingomonas sp IBVSS1
OSZ63707.1 (1985171)
NFUS01000007.1 (genomic DNA)

Proteobacteria bacterium ST_bin13
OQW71283.1 (1978761)
MSXQ01000025.1 (genomic)

Proteobacteria bacterium SG_bin5
OQW42034.1 (1827382)
LWDI01000062.1 (genomic DNA)

Eilatimonas milleporae
RMB02913.1 (911205)
REFR01000014.1 (genomic DNA)

Chromatiales bacterium USCg_Taylor
OOO03152.1 (1934255)
MUGK01000020.1 (genomic)

Gammaproteobacteria bacterium HGW Gammaproteobacteria 2
PKM16220.1 (2013798)
PGZE01000011.1 (genomic DNA)

Xanthomonadales bacterium CG17_big_fil_post_rev_8_21_14_2_50_64_11
PIQ36349.1 (1974109)
PCUP01000232.1 (genomic DNA)

Hydrocarboniphaga daqingensis
SHG95448.1 (490188)
FQWZ01000004.1 (genomic DNA)

Nevskia ramosa DSM 11499
ATVI01000008.1_83 (1122603)
ATVI01000008.1 ()

Uliginosibacterium sp TH139
PLK49076.1 (2067453)
PKRR01000003.1 (genomic DNA)

Proteobacteria bacterium UBA981
DBBP01000027.1_56 (1948226)
DBBP01000027.1 ()

Proteobacteria bacterium UBA1844
DDIS01000023.1_22 (1948138)
DDIS01000023.1 ()

Pseudolabrys sp Root1462
KQY97987.1 (1736466)
LMFS01000003.1 (genomic DNA)

Starkeya novella DSM 506
ADH90860.1 (639283)
CP002026.1 (chromosome)

Rhizobiales bacterium 65 9
OJY38223.1 (1895816)
MKVY01000027.1 (genomic DNA)

Microvirga sp 17 mud 1 3
AWM88364.1 (2082949)
CP029481.1 (chromosome)

Salinarimonas rosea DSM 21201
AUBC01000038.1_29 (1123229)
AUBC01000038.1 ()

Azorhizobium caulinodans ORS 571
BAF86802.1 (438753)
AP009384.1 (genomic DNA)

Methylopila sp M107
ARWB01000001.1_2242 (1101190)
ARWB01000001.1 ()

Methylopila sp Yamaguchi
GBD50583.1 (1437817)
BAUX01000021.1 (genomic DNA)

Methyloferula stellata AR4
ARWA01000001.1_1840 (876269)
ARWA01000001.1 ()

Niveispirillum irakense DSM 11586
AUCG01000006.1_28 (1121034)
AUCG01000006.1 ()

Parvibaculum lavamentivorans DS 1
ABS62042.1 (402881)
CP000774.1 (chromosome)

Phenylobacterium hankyongense
RAK60562.1 (1813876)
QFYP01000001.1 (genomic DNA)

Phenylobacterium sp Root1290
KQW95973.1 (1736445)
LMEE01000001.1 (genomic DNA)

Sphingomonas indica
SMF61140.1 (941907)
LT840185.1 (genomic DNA)

Sphingomonas spermidinifaciens
PCD01799.1 (1141889)
NWMW01000003.1 (genomic DNA)

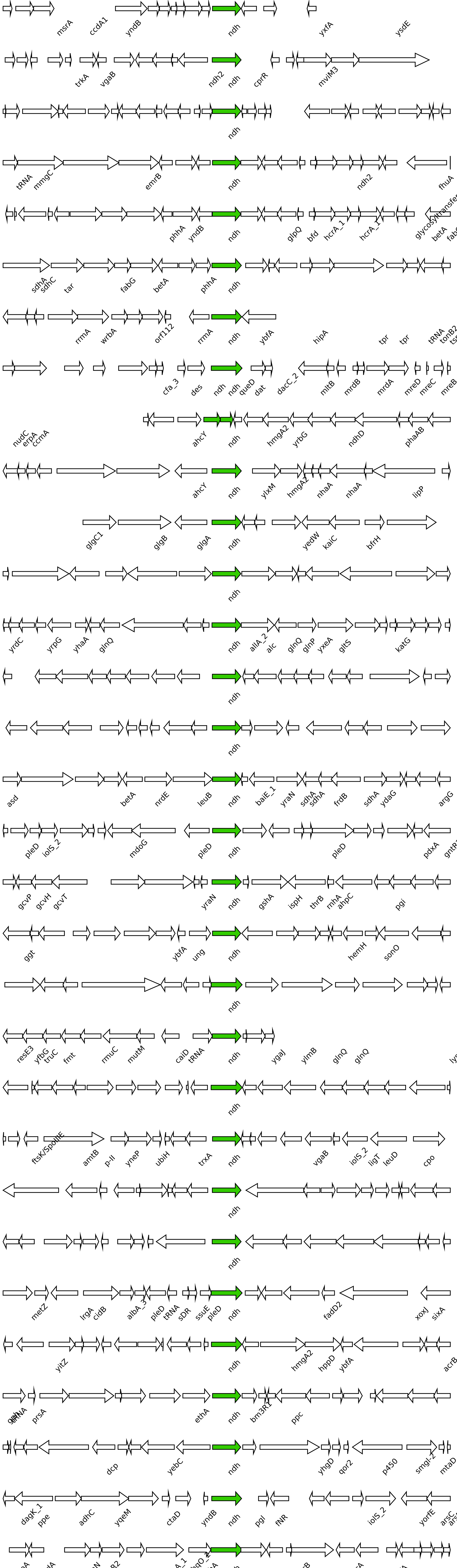

Sphingobium baderi
ALR23113.1 (1332080)
CP013266.1 (plasmid)

Novosphingobium lindaniclasticum LE124
EQB17730.1 (1096930)
ATHL01000052.1 (genomic)

Pusillimonas sp T2
OXR48657.1 (1548123)
NIQA01000007.1 (genomic DNA)

Aliiglaciecola lipolytica E3
GAC13283.1 (1127673)
BAEN01000016.1 (genomic DNA)

Aliiglaciecola lipolytica E3
GAC13296.1 (1127673)
BAEN01000016.1 (genomic DNA)

Kaistia soli DSM 19436
SHG63865.1 (1122133)
FQUP01000006.1 (genomic DNA)

Sphingomonas crusticola
QTJP01000001.1_576 (1697973)
QTJP01000001.1 ()

Sphingomonas asaccharolytica NBRC 15499
BCYU01000036.1_18 (1219040)
BCYU01000036.1 ()

Sphingobium sp 66 54
OJY60544.1 (1895845)
MKWI01000036.1 (genomic DNA)

Haematobacter sp UBA3484
DFQR01000014.1_11 (1946582)
DFQR01000014.1 ()

Mesorhizobium sp DCY119
CP031834.1_2607 (2108445)
CP031834.1 ()

Afipia sp P52 10
ETR77138.1 (1429916)
AZSJ01000003.1 (genomic DNA)

Mesorhizobium sp LA 28
RFC67599.1 (2294114)
QURN01000007.1 (genomic DNA)

Pseudaminobacter salicylatoxidans
PWJ86185.1 (93369)
QGGG01000001.1 (genomic DNA)

Methylocella silvestris BL2
ACK51538.1 (395965)
CP001280.1 (chromosome)

Rhizobiales bacterium 65 79
OJU02191.1 (1895815)
MKRJ01000032.1 (genomic DNA)

Aminobacter sp Root100
KQU63982.1 (1736420)
LMCL01000034.1 (genomic DNA)

Phyllobacterium leguminum
PYE86654.1 (314237)
QJTF01000021.1 (genomic DNA)

Bosea sp CS1GBMeth4
FYAS01000030.1_207 (1892849)
FYAS01000030.1 ()

Phyllobacteriaceae bacterium UBA1940
DDFA01000066.1_32 (1953060)
DDFA01000066.1 ()

Agrobacterium vitis
RCU54973.1 (373)
LMVL02000002.1 (genomic DNA)

Rhizobium sp JAB6
PST19153.1 (2127050)
PYJQ01000009.1 (genomic DNA)

Rhizobium sp Leaf321
KQQ72772.1 (1736335)
LMOF01000007.1 (genomic DNA)

Agrobacterium bohemicum
KXG86064.1 (2052828)
KQ961024.1 (genomic)

Rhizobium sp Y9
AUC10099.1 (1914541)
CP017999.1 (chromosome)

Agrobacterium genomosp 13 str CFBP 6927
CUX24906.1 (1183428)
LT009756.1 (genomic DNA)

Pleomorphomonas koreensis DSM 23070
AULH01000013.1_138 (1122962)
AULH01000013.1 ()

Asticcacaulis sp CF398
PTW93596.1 (2135738)
QBKE01000008.1 (genomic DNA)

Rhizobium sp Root482
KQY26982.1 (1736543)
LMFA01000001.1 (genomic DNA)

Mycoplana dimorpha
PTM91981.1 (28320)
PZZZ01000008.1 (genomic DNA)

Ketogulonicigenium vulgare Y25
ADO41627.1 (880591)

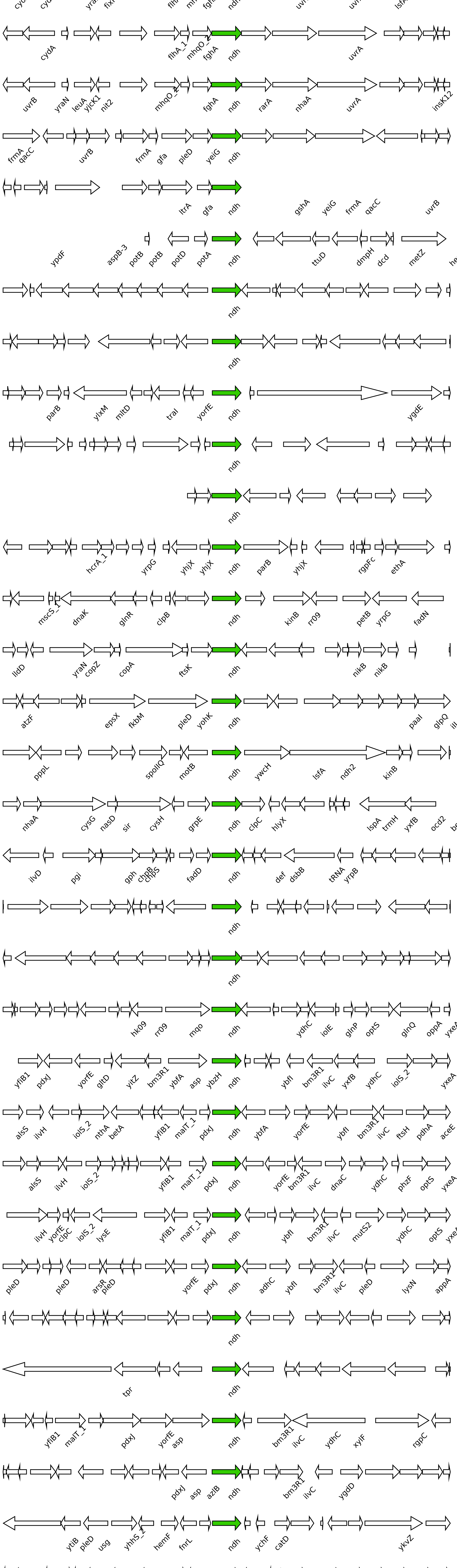

CP002224.1 (chromosome)

Paracoccus pantotrophus
RDD96361.1 (82367)
QOZU01000050.1 (genomic DNA)

Paracoccus alcaliphilus
SEO19375.1 (34002)
FODE01000043.1 (genomic DNA)

Aquamicrobium aerolatum DSM 21857
SFJ52393.1 (1121003)
FORF01000025.1 (genomic DNA)

Paracoccus aminophilus JCM 7686
AGT10974.1 (1367847)
CP006652.1 (plasmid)

Paracoccus sp BM15
AUH35130.1 (1529068)
CP025408.1 (chromosome)

Paracoccus zhejiangensis
AUH63882.1 (1077935)
CP025430.1 (chromosome)

Pseudochrobactrum asaccharolyticum
RBO97668.1 (354351)
QNRH01000002.1 (genomic DNA)

Falsochrobactrum ovis
RAK30992.1 (1293442)
QLMK01000003.1 (genomic DNA)

Brucella suis 1330
AAN33671.1 (204722)
AE014292.2 (chromosome)

Phyllobacterium sp YR531
EJN04870.1 (1144343)
AKIZ01000015.1 (genomic DNA)

Devosia sp A16
CP012945.1_3669 (1736675)
CP012945.1 ()

Devosia sp 66 22
OJX51501.1 (1895753)
MKUZ01000017.1 (genomic DNA)

Acetobacteraceae bacterium SCN 69 10
ODU54930.1 (1660159)
MEFR01000207.1 (genomic DNA)

Devosia sp H5989
AKR54776.1 (1643450)
CP011300.1 (chromosome)

Devosia geojensis
KKB12181.1 (443610)
JZEX01000088.1 (genomic DNA)

Devosia sp I507
AVF05713.1 (2083786)
CP026747.1 (chromosome)

Devosia sp Leaf420
KQT49892.1 (1736374)
LMQU01000009.1 (genomic DNA)

Gemmobacter sp YIM 102744 1
RRH72817.1 (2488730)
RRAZ01000021.1 (genomic DNA)

Alphaproteobacteria bacterium HGW Alphaproteobacteria 12
PKQ02206.1 (2013658)
PHEN01000531.1 (genomic DNA)

Caulobacteraceae bacterium OTSz_A_272
ANP46130.1 (1759059)
CP013244.1 (chromosome)

Micavibrio sp UBA2020
DDBY01000224.1_2 (1946912)
DDBY01000224.1 ()

Legionella sp km772
RUR04252.1 (2498111)
RZGQ01000340.1 (genomic DNA)

Micavibrio aeruginosavorus
PZO89028.1 (349221)
QFNK01000002.1 (genomic DNA)

Micavibrio sp UBA3393
DEPL01000064.1_10 (1946921)
DEPL01000064.1 ()

Alsobacter sp SH9
PSC06067.1 (2109933)
PVZS01000004.1 (genomic DNA)

Defluviimonas aquaemixtae
SPH17516.1 (1542388)
OMOQ01000001.1 (genomic DNA)

Hartmannibacter diazotrophicus
SON57750.1 (1482074)
LT960614.1 (genomic DNA)

Sphingomonas sp CCH20 B6
LSJC01000176.1_2 (1768769)
LSJC01000176.1 ()

Woeseia oceani
ANO50449.1 (1548547)
CP016268.1 (chromosome)

Sphingomonas sp URHD0007
PVZ18593.1 (1298856)
QEQM01000001.1 (genomic DNA)

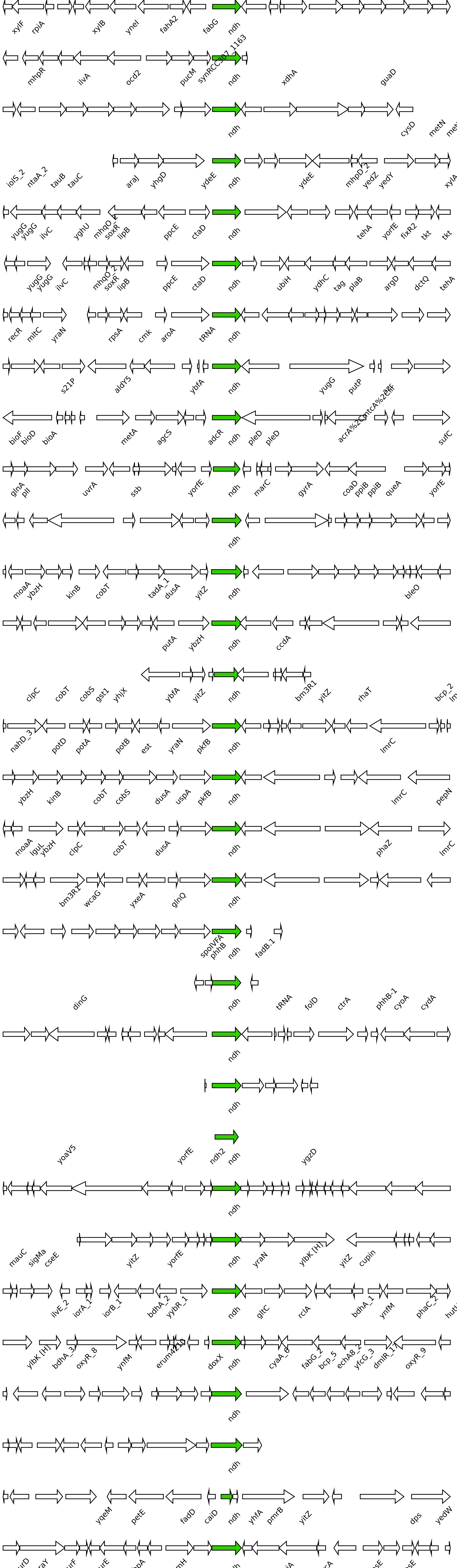

Methylocystis rosea
AZG76938.1 (173366)
CP034086.1 (chromosome)

Cucumibacter marinus DSM 18995
AUCR01000002.1_72 (422296)
AUCR01000002.1 ()

Candidatus Competibacter denitrificans Run_A_D11
CDI04447.1 (1400863)
CBTJ020000111.1 (genomic DNA)

Candidatus Competibacteraceae bacterium
HAO31468.1 (2053538)
DMQI01000016.1 (genomic DNA)

Thalassospira sp TSL5 1
OKH89466.1 (1544451)
KV880637.1 (genomic)

Thalassospira sp UBA2642
DENO01000345.1_16 (1947674)
DENO01000345.1 ()

Thalassospira indica
AXO14491.1 (1891279)
CP031555.1 (chromosome)

Cohaesibacter haloalkalitolerans
QNUM01000009.1_138 (1162980)
QNUM01000009.1 ()

Tateyamaria omphalii
APX13577.1 (299262)
CP019312.1 (chromosome)

Hydrocarboniphaga effusa AP103
EIT71414.1 (1172194)
AKGD01000001.1 (genomic DNA)

Acidovorax delafieldii
LSHP01000057.1_13 (47920)
LSHP01000057.1 ()

Sphingorhabdus sp Alg231 15
LT703001.1_234 (1922222)
LT703001.1 ()

Erythrobacter sp KY5
AWW75045.1 (2011159)
CP021912.1 (chromosome)

Erythrobacter longus
KEO91707.1 (1044)
JMIW01000001.1 (genomic DNA)

Erythrobacter sp Alg231 14
LT702999.1_647 (1922225)
LT702999.1 ()

Erythrobacter sp YT30
KWV93214.1 (1735012)
LMAF01000001.1 (genomic DNA)

Alphaproteobacteria bacterium HGW Alphaproteobacteria 16
PKP89613.1 (2013662)
PHEJ01000138.1 (genomic DNA)

Devosia enhydra
SFZ80623.1 (665118)
FPKU01000001.1 (genomic DNA)

Pelagibacterium montanilacus
RZMX01000046.1_14 (2185280)
RZMX01000046.1 ()

Pelagibacterium lentulum
RZMW01000029.1_210 (2029865)
RZMW01000029.1 ()

Pelagibacterium luteolum
SDG38735.1 (440168)
FNCS01000002.1 (genomic DNA)

Pelagibacterium halotolerans B2
AEQ50644.1 (1082931)
CP003075.1 (chromosome)

Arsenicitalea aurantiaca
RUT34751.1 (1783274)
RZNJ01000001.1 (genomic DNA)

Rhizobiaceae bacterium CPCC 101076
QDFX01000002.1_190 (2172049)
QDFX01000002.1 ()

Lutibaculum baratangense AMV1
ESR27005.1 (631454)
AWXZ01000012.1 (genomic DNA)

Alphaproteobacteria bacterium UBA5181
DHWN01000125.1_3 (1948408)
DHWN01000125.1 ()

Mangrovicella endophytica
PKMZ01000002.1_507 (2066697)
PKMZ01000002.1 ()

Citreicella sp
HBM62019.1 (1960284)
DOAJ01000233.1 (genomic DNA)

Roseivivax isoporae LMG 25204
ETX28979.1 (1449351)
JAME01000013.1 (genomic)

Jannaschia sp 12N15
QPLJ01000036.1_21 (2259592)
QPLJ01000036.1 ()

Silicimonas algicola
PWK59060.1 (1826607)
[illegible]

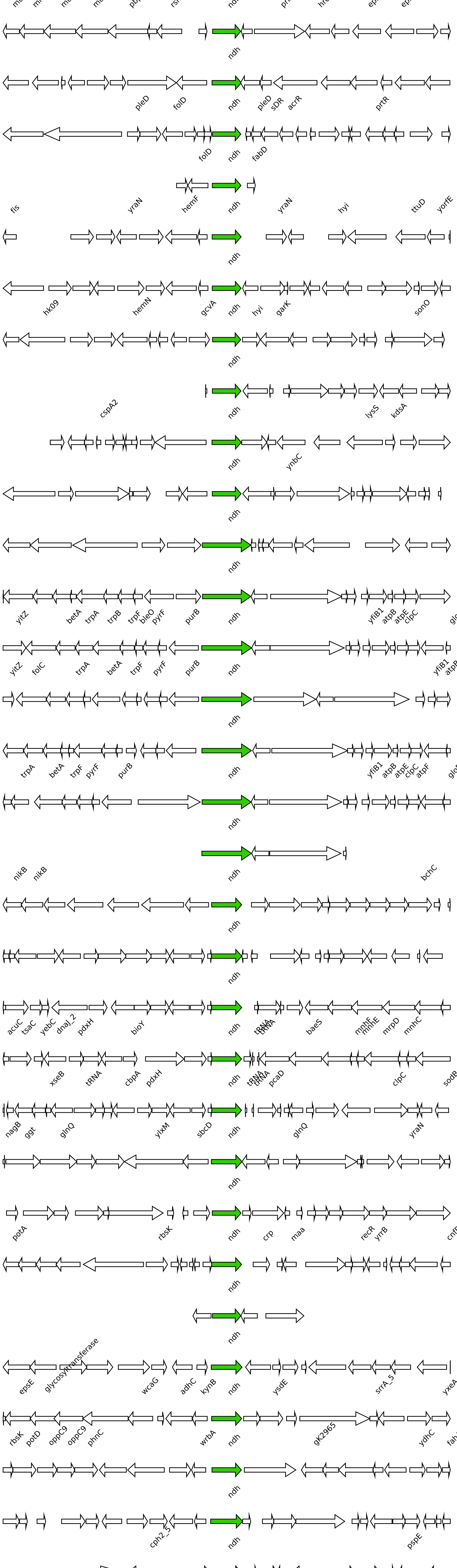

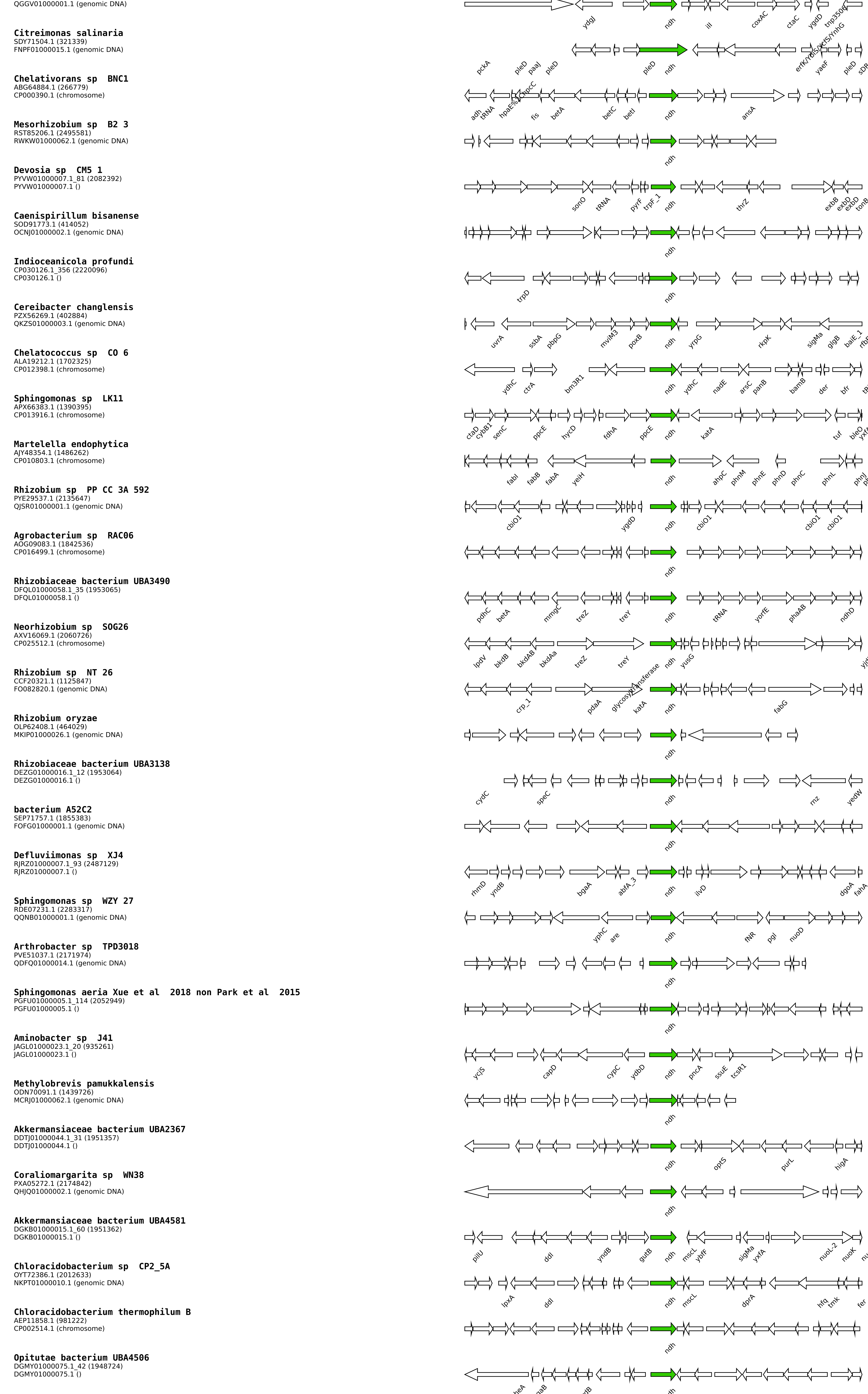

QGGV01000001.1 (genomic DNA)
ydgJ ndh ill coxAC ctaC ygdD tnp3506
Citreimonas salinaria
SDY71504.1 (321339)
FNPF01000015.1 (genomic DNA)
pckA pleD paaJ pleD pleD ndh erfK/YbiS/YcfS/YnhG yaeF pleD SDR
Chelativorans sp BNC1
ABG64884.1 (266779)
CP000390.1 (chromosome)
adh tRNA hpaE%2ChpcC fis betA betC betI ndh ansA
Mesorhizobium sp B2 3
RST85206.1 (2495581)
RWKW01000062.1 (genomic DNA)
ndh
Devosia sp CM5 1
PYVW01000007.1_81 (2082392)
PYVW01000007.1 ()
sonO tRNA pyrF trpF_1 ndh thrZ exbB exbD exbD tonB
Caenispirillum bisanense
SOD91773.1 (414052)
OCNJ01000002.1 (genomic DNA)
ndh
Indioceanicola profundi
CP030126.1_356 (2220096)
CP030126.1 ()
trpD ndh
Cereibacter changlensis
PZX56269.1 (402884)
QKZS01000003.1 (genomic DNA)
uvrA ssbA pbpG mviM3 poxB ndh yrpG rkpK sigMa glgB baiE_1
Chelatococcus sp CO 6
ALA19212.1 (1702325)
CP012398.1 (chromosome)
ydhC ctrA bm3R1 ndh ydhC nadE arsC panB bamB der bfr
Sphingomonas sp LK11
APX66383.1 (1390395)
CP013916.1 (chromosome)
ctaD cybB1 senC ppcE hycD fdhA ppcE ndh katA tuf bleO
Martelella endophytica
AJY48354.1 (1486262)
CP010803.1 (chromosome)
fabI fabB fabA yeiH ndh ahpC phnM phnE phnD phnC phnL phnJ
Rhizobium sp PP CC 3A 592
PYE29537.1 (2135647)
QJSR01000001.1 (genomic DNA)
cbiO1 ygdD ndh cbiO1 cbiO1 cbiO1
Agrobacterium sp RAC06
AOG09083.1 (1842536)
CP016499.1 (chromosome)
ndh
Rhizobiaceae bacterium UBA3490
DFQL01000058.1_35 (1953065)
DFQL01000058.1 ()
pdhC betA mmgC treZ treY ndh tRNA yorfE phaAB ndhD
Neorhizobium sp SOG26
AXV16069.1 (2060726)
CP025512.1 (chromosome)
lpdV bkdB bkdAB bkdAa treZ treY ndh yusG
Rhizobium sp NT 26
CCF20321.1 (1125847)
FO082820.1 (genomic DNA)
crp_1 pdaA glycosyltransferase katA ndh fabG
Rhizobium oryzae
OLP62408.1 (464029)
MKIP01000026.1 (genomic DNA)
ndh
Rhizobiaceae bacterium UBA3138
DEZG01000016.1_12 (1953064)
DEZG01000016.1 ()
cydC speC ndh rnz yedW
bacterium A52C2
SEP71757.1 (1855383)
FOFG01000001.1 (genomic DNA)
ndh
Defluviimonas sp XJ4
RJRZ01000007.1_93 (2487129)
RJRZ01000007.1 ()
rhmD yndB bgaA abfA_3 ndh ilvD dgoA fahA
Sphingomonas sp WZY 27
RDE07231.1 (2283317)
QQNB01000001.1 (genomic DNA)
yphC are ndh fNR pgl nuoD
Arthrobacter sp TPD3018
PVE51037.1 (2171974)
QDFQ01000014.1 (genomic DNA)
ndh
Sphingomonas aeria Xue et al 2018 non Park et al 2015
PGFU01000005.1_114 (2052949)
PGFU01000005.1 ()
ndh
Aminobacter sp J41
JAGL01000023.1_20 (935261)
JAGL01000023.1 ()
yciS capD cypC ydbD ndh pncA ssuE tcsR1
Methylobrevis pamukkalensis
ODN70091.1 (1439726)
MCRJ01000062.1 (genomic DNA)
ndh
Akkermansiaceae bacterium UBA2367
DDTJ01000044.1_31 (1951357)
DDTJ01000044.1 ()
ndh optS purL higA
Coraliomargarita sp WN38
PXA05272.1 (2174842)
QHJQ01000002.1 (genomic DNA)
ndh
Akkermansiaceae bacterium UBA4581
DGKB01000015.1_60 (1951362)
DGKB01000015.1 ()
pilU ddl yndB gutB ndh mscL ybfF sigMa yxfA nuoL-2 nuoK
Chloracidobacterium sp CP2_5A
OYT72386.1 (2012633)
NKPT01000010.1 (genomic DNA)
lpxA ddl ndh mscL dprA hfq tmk fer
Chloracidobacterium thermophilum B
AEP11858.1 (981222)
CP002514.1 (chromosome)
ndh
Opitutae bacterium UBA4506
DGMY01000075.1_42 (1948724)
DGMY01000075.1 ()

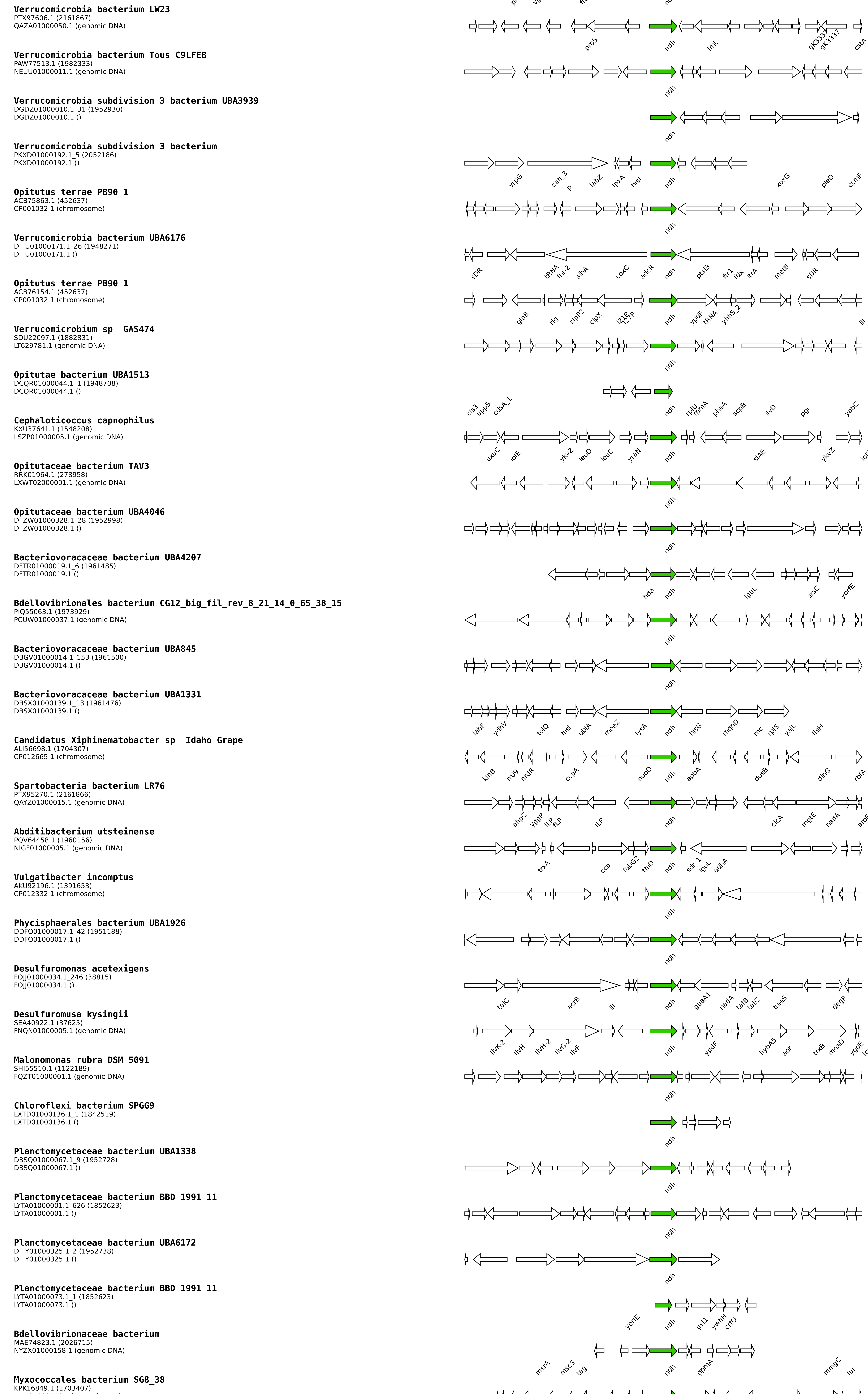

Verrucomicrobia bacterium LW23
PTX97606.1 (2161867)
QAZA01000050.1 (genomic DNA)
Verrucomicrobia bacterium Tous C9LFEB
PAW77513.1 (1982333)
NEUU01000011.1 (genomic DNA)
Verrucomicrobia subdivision 3 bacterium UBA3939
DGDZ01000010.1_31 (1952930)
DGDZ01000010.1 ()
Verrucomicrobia subdivision 3 bacterium
PKXD01000192.1_5 (2052186)
PKXD01000192.1 ()
Opitutus terrae PB90 1
ACB75863.1 (452637)
CP001032.1 (chromosome)
Verrucomicrobia bacterium UBA6176
DITU01000171.1_26 (1948271)
DITU01000171.1 ()
Opitutus terrae PB90 1
ACB76154.1 (452637)
CP001032.1 (chromosome)
Verrucomicrobium sp GAS474
SDU22097.1 (1882831)
LT629781.1 (genomic DNA)
Opitutae bacterium UBA1513
DCQR01000044.1_1 (1948708)
DCQR01000044.1 ()
Cephaloticoccus capnophilus
KXU37641.1 (1548208)
LSZP01000005.1 (genomic DNA)
Opitutaceae bacterium TAV3
RRK01964.1 (278958)
LXWT02000001.1 (genomic DNA)
Opitutaceae bacterium UBA4046
DFZW01000328.1_28 (1952998)
DFZW01000328.1 ()
Bacteriovoracaceae bacterium UBA4207
DFTR01000019.1_6 (1961485)
DFTR01000019.1 ()
Bdellovibrionales bacterium CG12_big_fil_rev_8_21_14_0_65_38_15
PIQ55063.1 (1973929)
PCUW01000037.1 (genomic DNA)
Bacteriovoracaceae bacterium UBA845
DBGV01000014.1_153 (1961500)
DBGV01000014.1 ()
Bacteriovoracaceae bacterium UBA1331
DBSX01000139.1_13 (1961476)
DBSX01000139.1 ()
Candidatus Xiphinematobacter sp Idaho Grape
ALJ56698.1 (1704307)
CP012665.1 (chromosome)
Spartobacteria bacterium LR76
PTX95270.1 (2161866)
QAYZ01000015.1 (genomic DNA)
Abditibacterium utsteinense
PQV64458.1 (1960156)
NIGF01000005.1 (genomic DNA)
Vulgatibacter incomptus
AKU92196.1 (1391653)
CP012332.1 (chromosome)
Phycisphaerales bacterium UBA1926
DDFO01000017.1_42 (1951188)
DDFO01000017.1 ()
Desulfuromonas acetexigens
FOJJ01000034.1_246 (38815)
FOJJ01000034.1 ()
Desulfuromusa kysingii
SEA40922.1 (37625)
FNQN01000005.1 (genomic DNA)
Malonomonas rubra DSM 5091
SHI55510.1 (1122189)
FQZT01000001.1 (genomic DNA)
Chloroflexi bacterium SPGG9
LXTD01000136.1_1 (1842519)
LXTD01000136.1 ()
Planctomycetaceae bacterium UBA1338
DBSQ01000067.1_9 (1952728)
DBSQ01000067.1 ()
Planctomycetaceae bacterium BBD 1991 11
LYTA01000001.1_626 (1852623)
LYTA01000001.1 ()
Planctomycetaceae bacterium UBA6172
DITY01000325.1_2 (1952738)
DITY01000325.1 ()
Planctomycetaceae bacterium BBD 1991 11
LYTA01000073.1_1 (1852623)
LYTA01000073.1 ()
Bdellovibrionaceae bacterium
MAE74823.1 (2026715)
NYZX01000158.1 (genomic DNA)
Myxococcales bacterium SG8_38
KPK16849.1 (1703407)

Myxococcales bacterium SG8_38_1
KPK54842.1 (1703408)
LJTO01000006.1 (genomic DNA)

Phycisphaerales bacterium UBA1924
DDFQ01000006.1_339 (1951187)
DDFQ01000006.1 ()

Candidatus Sulfotelmatomonas gaucii
SPE30665.1 (2043161)
OKRB01000141.1 (genomic DNA)

Terracidiphilus gabretensis
LAIJ01000012.1_435 (1577687)
LAIJ01000012.1 ()

Acidobacterium ailaaui
JIAL01000001.1_1082 (1382359)
JIAL01000001.1 ()

Acidipila sp 4G K13
RFU16937.1 (2303751)
QVQT01000003.1 (genomic DNA)

Acidobacterium capsulatum ATCC 51196
ACO33593.1 (240015)
CP001472.1 (chromosome)

Terriglobus roseus DSM 18391
AFL87150.1 (926566)
CP003379.1 (chromosome)

Terriglobus saanensis SP1PR4
ADV83923.1 (401053)
CP002467.1 (chromosome)

Granulicella rosea
SNT27266.1 (474952)
FZOU01000006.1 (genomic DNA)

Granulicella tundricola MP5ACTX9
ADW70265.1 (1198114)
CP002480.1 (chromosome)

Acidobacteriales bacterium 59 55
OJV39808.1 (1895690)
MKSV01000010.1 (genomic DNA)

Edaphobacter dinghuensis
RKQ65774.1 (1560005)
RBIF01000001.1 (genomic DNA)

Granulicella pectinivorans
SFS09517.1 (474950)
FOZL01000001.1 (genomic DNA)

Granulicella mallensis MP5ACTX8
AEU34947.1 (682795)
CP003130.1 (chromosome)

Bryocella elongata
SEG49957.1 (863522)
FNVA01000005.1 (genomic DNA)

Litoreibacter ponti
PTX56534.1 (1510457)
QBKS01000001.1 (genomic DNA)

Micavibrio sp UBA1672
DCKO01000007.1_9 (1946908)
DCKO01000007.1 ()

Methylomarinum vadi
JPON01000001.1_2240 (438855)
JPON01000001.1 ()

Methylomonas methanica MC09
AEG00817.1 (857087)
CP002738.1 (chromosome)

Ectothiorhodospira sp BSL 9
ANB01785.1 (1442136)
CP011994.1 (chromosome)

Chromatiales bacterium USCg_Taylor
OOO03151.1 (1934255)
MUGK01000020.1 (genomic)

Myxococcales bacterium SG8_38
KPK17386.1 (1703407)
LJTN01000004.1 (genomic DNA)

Myxococcales bacterium SG8_38_1
KPK54880.1 (1703408)
LJTO01000005.1 (genomic DNA)

Proteobacteria bacterium UBA2008
DDCK01000066.1_24 (1948142)
DDCK01000066.1 ()

Gammaproteobacteria bacterium REDSEA S15_B12
LUSG01000028.1_2 (1811666)
LUSG01000028.1 ()

Planctomycetaceae bacterium UBA969
DBCB01000114.1_11 (1952747)
DBCB01000114.1 ()

Brevundimonas sp LM2
AQR60987.1 (1938605)
CP019508.1 (chromosome)

Bacillus sp 2B10
RPSH01000015.1_71 (2483355)
RPSH01000015.1 ()

Pacificimonas flava
EMD83589.1 (1234595)
AMRV01000002.1 (genomic DNA)

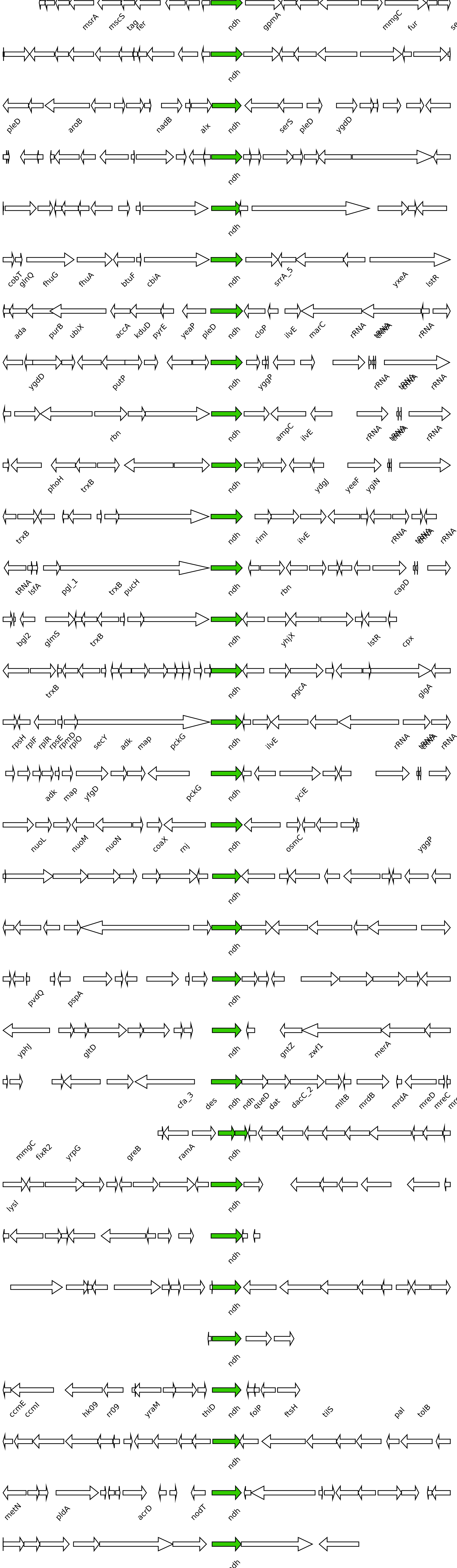

Erythrobacter sp REDSEA S45_B7
LUNY01000099.1_1 (1811540)
LUNY01000099.1 ()

Erythrobacter xanthus
RIV82768.1 (1784712)
QXFM01000116.1 (genomic DNA)

Erythrobacter atlanticus
ANC50562.1 (1648404)
CP015441.1 (plasmid)

Erythrobacter flavus
ASP29366.1 (192812)
CP022528.1 (chromosome)

Croceicoccus marinus
ARU18483.1 (450378)
CP019604.1 (plasmid)

Altererythrobacter atlanticus
AKH42740.1 (1267766)
CP011452.2 (chromosome)

Erythrobacteraceae bacterium UBA2014
DDCE01000022.1_4 (1951752)
DDCE01000022.1 ()

Roseovarius lutimaris
SFO25564.1 (1005928)
FOVP01000021.1 (genomic DNA)

Sphingomonas sp Leaf339
KQU45166.1 (1736343)
LMRS01000061.1 (genomic DNA)

Chloroflexi bacterium OLB15
KXK14552.1 (1617416)
LMZS01000161.1 (genomic DNA)

Ilumatobacter coccineus YM16 304
BAN03570.1 (1313172)
AP012057.1 (genomic DNA)

Ignavibacteriales bacterium CG_4_9_14_3_um_filter_34_10
PJA97667.1 (1974044)
PFVB01000004.1 (genomic DNA)

Ignavibacteriales bacterium CG18_big_fil_WC_8_21_14_2_50_31_20
PIQ09079.1 (1974047)
PCUD01000092.1 (genomic DNA)

Ignavibacteriales bacterium CG18_big_fil_WC_8_21_14_2_50_31_20
PIQ09939.1 (1974047)
PCUD01000047.1 (genomic DNA)

Ignavibacteriales bacterium UTCHB1
OQY79055.1 (1961104)
MWSW01000004.1 (genomic DNA)

Ignavibacteria bacterium UBA2330
DDUU01000026.1_26 (1948666)
DDUU01000026.1 ()

Ignavibacteria bacterium UBA6667
DKKL01000007.1_225 (1948682)
DKKL01000007.1 ()

Leptospira interrogans serovar Lai str 56601
AAN51595.1 (189518)
AE010301.2 (chromosome)

Leptospira hartskeerlii
PJZ33188.1 (2023177)
NPDL01000006.1 (genomic DNA)

Leptospira wolffii serovar Khorat str Khorat H2
EPG65975.1 (1193069)
AKWX02000013.1 (genomic)

Leptospira sp
PJE00684.1 (178)
PEQN01000037.1 (genomic DNA)

Leptospira biflexa serovar Patoc strain Patoc 1 Ames
ABZ95845.1 (355278)
CP000778.1 (chromosome)

Leptospira sp E30
GBF48377.1 (1917830)
BFBA01000014.1 (genomic DNA)

Leptospira sp YH101
GBF51911.1 (1917863)
BFBB01000009.1 (genomic DNA)

Proteobacteria bacterium UBA5335
DHQP01000012.1_17 (1948181)
DHQP01000012.1 ()

Salinisphaeraceae bacterium
MAA75641.1 (2026789)
NYTH01000047.1 (genomic DNA)

Salinisphaera sp UBA6602
DJIE01000148.1_29 (1947455)
DJIE01000148.1 ()

Salinisphaera halophila YIM 95161
ROO25720.1 (1051139)
AYKF01000110.1 (genomic)

Algiphilus aromaticivorans DG1253
JPOG01000001.1_2344 (1415780)
JPOG01000001.1 ()

Sinobacteraceae bacterium UBA5061
DIBD01000042.1_96 (1953077)
DIBD01000042.1 ()

Solimonas sp K1W22B 7
AXQ31337.1 (2303331)
CP031704.1 (chromosome)

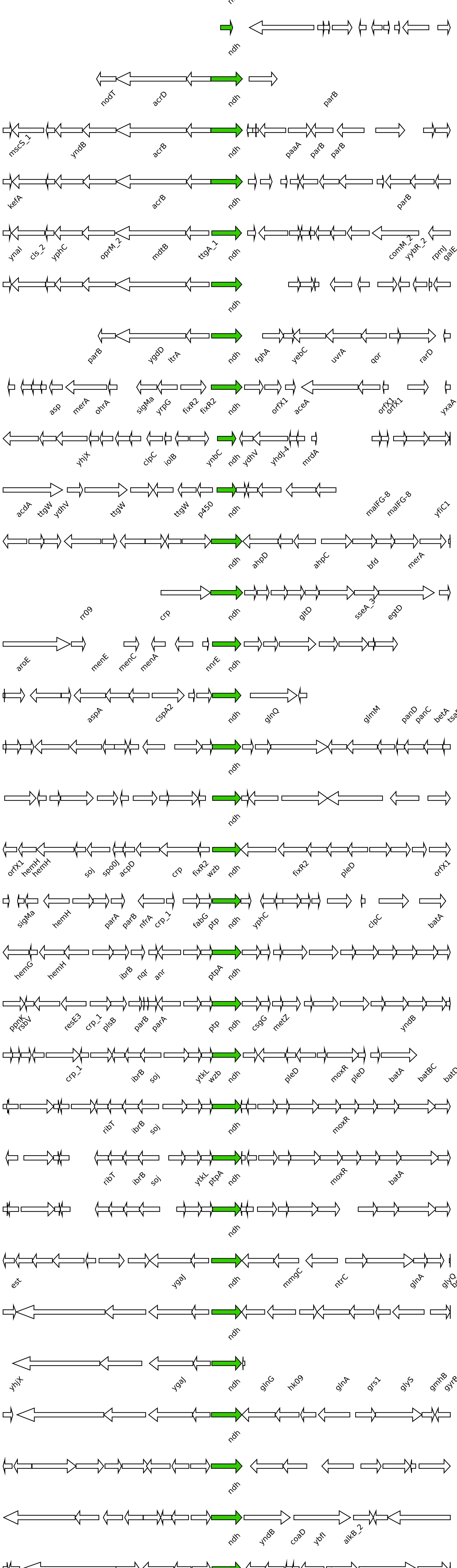

CP031704.1 (chromosome)

Fontimonas thermophila
SFF22511.1 (1076937)
FOOC01000001.1 (genomic DNA)

Sinimarinibacterium flocculans
PXV66572.1 (985250)
QICN01000007.1 (genomic DNA)

Sinobacteraceae bacterium GT1R17
PTU31202.1 (2029166)
QANS01000003.1 (genomic DNA)

Nevskiales bacterium
MBV61039.1 (2026766)
PCCX01000029.1 (genomic DNA)

Oceanococcus atlanticus
ORE89515.1 (1317117)
AQQV01000001.1 (genomic DNA)

Chthonomonas calidirosea T49
CCW35094.1 (1303518)
HF951689.1 (genomic DNA)

Alphaproteobacteria bacterium MarineAlpha5_Bin2
PPR54379.1 (2013077)
PTLC01000033.1 (genomic DNA)

Pelagibacteraceae bacterium AG 464 P07
QCSA01000008.1_24 (2162008)
QCSA01000008.1 ()

alpha proteobacterium SCGC AB 629 G21
AQVO01000008.1_21 (1131276)
AQVO01000008.1 ()

alpha proteobacterium SCGC AB 629 F11
AQVN01000025.1_1 (1131277)
AQVN01000025.1 ()

Dyella sp 4G K06
RDS81304.1 (1927958)
QRBE01000006.1 (genomic DNA)

Sphingorhabdus sp
MAC11224.1 (1902408)
NYUA01000040.1 (genomic DNA)

Akkermansiaceae bacterium UBA1506
DCQY01000001.1_4 (1951351)
DCQY01000001.1 ()

Rubritalea squalenifaciens DSM 18772
SHJ65259.1 (1123071)
FQYR01000004.1 (genomic DNA)

Rubritalea profundi
PQJ28737.1 (1658618)
MQWA01000001.1 (genomic DNA)

Rubritalea marina DSM 17716
KB899255.1_90 (1123070)
KB899255.1 ()

Akkermansiaceae bacterium UBA1315
DBTN01000042.1_43 (1951350)
DBTN01000042.1 ()

Akkermansiaceae bacterium UBA7811
DLBZ01000156.1_9 (1951373)
DLBZ01000156.1 ()

Akkermansiaceae bacterium UBA1011
DBAL01000329.1_2 (1951348)
DBAL01000329.1 ()

Phycisphaera mikurensis NBRC 102666
BAM04584.1 (1142394)
AP012338.1 (genomic DNA)

Bradyrhizobium sp JGI 0001002 A22
AUOJ02000012.1_28 (1094929)
AUOJ02000012.1 ()

Edaphobacter dinghuensis
RKQ65164.1 (1560005)
RBIF01000001.1 (genomic DNA)

Acidobacterium capsulatum ATCC 51196
ACO34099.1 (240015)
CP001472.1 (chromosome)

Solirubrobacterales bacterium
PKXY01000033.1_126 (1909295)
PKXY01000033.1 ()

Pseudonocardia sp AL041005 10
ALE80779.1 (445576)
CP011862.1 (chromosome)

Geodermatophilus obscurus DSM 43160
ADB74634.1 (526225)
CP001867.1 (chromosome)

Catenuloplanes indicus JCM 9534
BBHZ01001715.1_1 (1307450)
BBHZ01001715.1 ()

Pseudopropionibacterium propionicum F0230a
AFN46434.1 (767029)
CP002734.1 (chromosome)

Trueperella bialowiezensis
VEI13637.1 (312285)
LR134476.1 (genomic DNA)

Trueperella pyogenes TP8
AJC68814.1 (1435056)
CP007003.1 (chromosome)

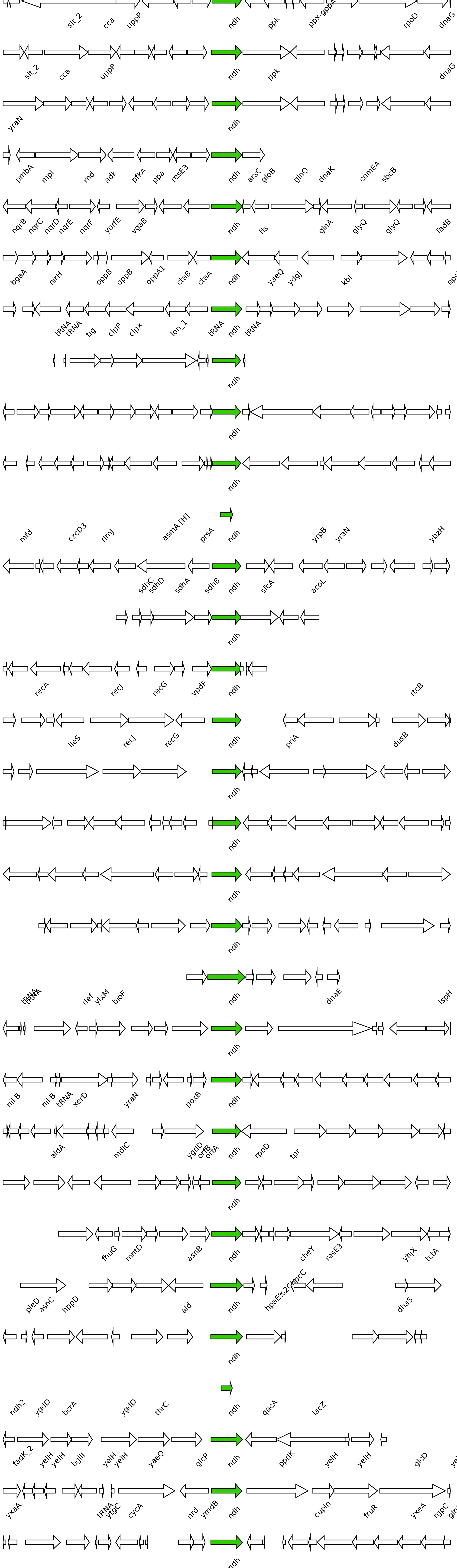

Actinomyces mediterranea
FTPB01000004.1_271 (1871028)
FTPB01000004.1 ()

Actinomyces cardiffensis F0333
ENO17624.1 (888050)
KB822675.1 (genomic)

Actinomyces vaccimaxillae DSM 15804
ATUX01000007.1_344 (1120947)
ATUX01000007.1 ()

Actinomyces suimastitidis DSM 15538
AUBF01000007.1_154 (1120946)
AUBF01000007.1 ()

Streptomyces cattleya
JWDF01000009.1_172 (29303)
JWDF01000009.1 ()

Actinomyces meyeri
AKU64969.1 (52773)
CP012072.1 (chromosome)

Actinomyces canis
RRC95113.1 (100469)
RQZF01000006.1 (genomic DNA)

Actinomyces provencensis
LT700212.1_29 (1720198)
LT700212.1 ()

Actinomyces sp oral taxon 897
AVM61770.1 (2081702)
CP027236.1 (chromosome)

Actinomyces coleocanis DSM 15436
EEH63507.1 (525245)
DS999541.1 (genomic)

Kineosphaera limosa NBRC 100340
GAB97157.1 (1184609)
BAHD01000058.1 (genomic DNA)

Tessaracoccus oleiagri
SDL21757.1 (686624)
FNGP01000001.1 (genomic DNA)

Tessaracoccus sp JDX10
RMB61991.1 (2479848)
REFW01000001.1 (genomic DNA)

Tessaracoccus flavescens
AQP50392.1 (399497)
CP019607.1 (chromosome)

Tessaracoccus sp ZS01
OMG53361.1 (1906324)
MTHC01000015.1 (genomic DNA)

Tessaracoccus flavus
AQP45459.1 (1610493)
CP019605.1 (chromosome)

Tessaracoccus sp T2 5 30
AQX15551.1 (1909732)
CP019229.1 (chromosome)

Actinomyces neuii
KWZ72884.1 (33007)
KQ955235.1 (genomic)

Arcanobacterium urinimassiliense
LT632435.1_715 (1871014)
LT632435.1 ()

Arcanobacterium haemolyticum DSM 20595
ADH92883.1 (644284)
CP002045.1 (chromosome)

Tessaracoccus sp Marseille P5995
LT996886.1_1519 (2161816)
LT996886.1 ()

Actinomyces minihominis
FYEG01000006.1_18 (2002838)
FYEG01000006.1 ()

Austwickia chelonae
CP031447.1_1740 (100225)
CP031447.1 ()

Blastococcus sp Marseille P5729
OMPO01000001.1_754 (2086582)
OMPO01000001.1 ()

Antricoccus suffuscus
PRZ40419.1 (1629062)
PVUE01000017.1 (genomic DNA)

Ornithinimicrobium sp KCTC 49018
QDDJ01000009.1_92 (2144182)
QDDJ01000009.1 ()

Tetrasphaera jenkinsii Ben 74
CCI53238.1 (1193518)
HF571038.1 (genomic DNA)

Tetrasphaera australiensis Ben110
CCH72978.1 (1193182)
HG764815.1 (genomic DNA)

Actinobacteria bacterium DSM 45722
SCX59613.1 (1566299)
FMUH01000008.1 (genomic DNA)

Tetrasphaera japonica T1 X7
CCH77295.1 (1194083)
HF570958.1 (genomic DNA)

Phycicoccus cremeus
SER74684.1 (587636)
FOHB01000001.1 (genomic DNA)

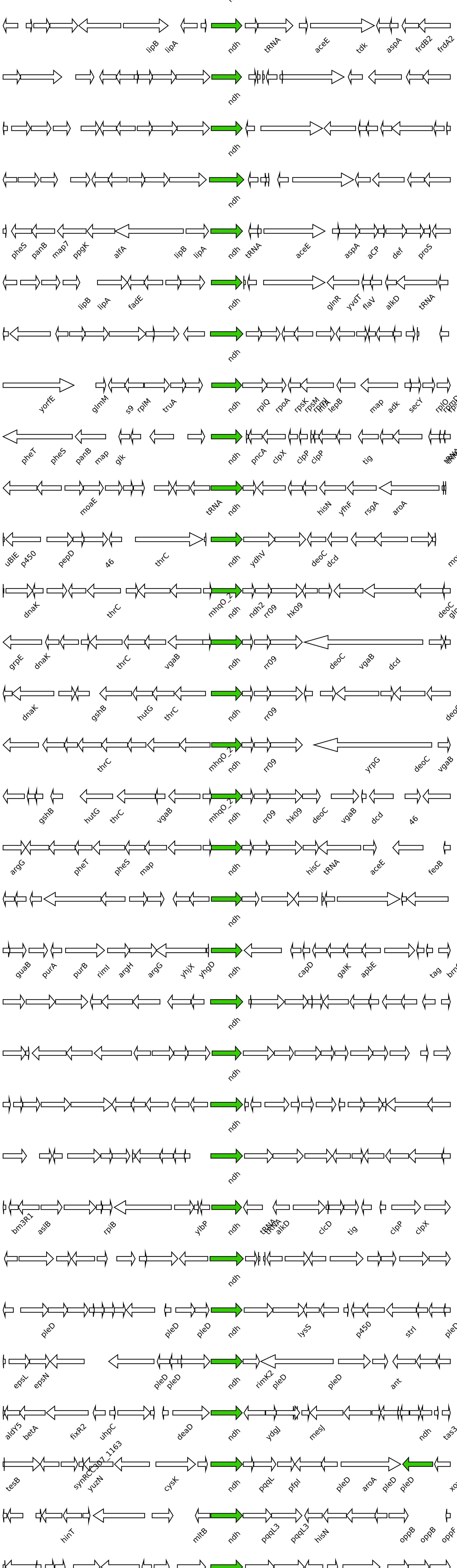

[illegible] (genomic DNA)

Phycicoccus dokdonensis
SDO63465.1 (443156)
LT629711.1 (genomic DNA)

Frankia casuarinae
ABD10359.1 (106370)
CP000249.1 (chromosome)

Intrasporangium calvum DSM 43043
ADU48939.1 (710696)
CP002343.1 (chromosome)

Tetrasphaera remsis
PRY62961.1 (407159)
PVTI01000003.1 (genomic DNA)

Janibacter sp HTCC2649
EAP99957.1 (313589)
CH672413.1 (genomic)

Tetrasphaera elongata Lp2
CCH71049.1 (1193181)
HF570956.1 (genomic DNA)

Kribbia dieselivorans NBRC 106261
BCRE01000116.1_16 (1223526)
BCRE01000116.1 ()

Janibacter sp Marseille P4121
LT985196.1_78 (2058291)
LT985196.1 ()

Tetrasphaera sp Soil756
KRE61232.1 (1736399)
LMSE01000004.1 (genomic DNA)

Nocardioides sp CF167
PTX39931.1 (2135685)
QBKO01000002.1 (genomic DNA)

Nocardioides sp CCTCC AB 2018079
PWN01152.1 (2201891)
QGDD01000011.1 (genomic DNA)

Nocardioides sp CF204
PTW71630.1 (2135677)
QAYJ01000001.1 (genomic DNA)

Marmoricola aequoreus
JOJN01000003.1_429 (397278)
JOJN01000003.1 ()

Marmoricola sp URHB0036
AUEP01000010.1_162 (1298863)
AUEP01000010.1 ()

Marmoricola scoriae
SDT00242.1 (642780)
LT629757.1 (genomic DNA)

Marmoricola sp Leaf446
KQT92219.1 (1736379)
LMRF01000002.1 (genomic DNA)

Nocardioides terrae
SFC34161.1 (574651)
FOLB01000005.1 (genomic DNA)

Nocardioides sp Root140
KQY56967.1 (1736460)
LMFI01000010.1 (genomic DNA)

Nocardioides daejeonensis
QHKZ01000011.1_84 (1046556)
QHKZ01000011.1 ()

Nocardioides psychrotolerans
SFI43249.1 (1005945)
FOQG01000008.1 (genomic DNA)

Nocardioides alkalitolerans DSM 16699
KE384533.1_216 (1122608)
KE384533.1 ()

Nocardioides dokdonensis FR1436
ANH37302.1 (1300347)
CP015079.1 (chromosome)

Nocardioides sp Root122
KQV77667.1 (1736431)
LMDE01000001.1 (genomic DNA)

Nocardioides sp Soil777
KRF02607.1 (1736409)
LMSN01000012.1 (genomic DNA)

Nocardioides sp CF8
EON22795.1 (110319)
CM001852.1 (chromosome)

Nocardioides sp DN36
BBEJ01000681.1_2 (652434)
BBEJ01000681.1 ()

Nocardioides sp WSJ 1
PVG84440.1 (2138300)
QDGZ01000001.1 (genomic DNA)

Nocardioides sp SZ4R5S7
QZVR01000011.1_58 (2340821)
QZVR01000011.1 ()

Nocardioides sp JS614
ABL83786.1 (196162)
CP000509.1 (chromosome)

Nocardioides sp PD653 B2
GAW51775.1 (1892811)
BDJE01000047.1 (genomic DNA)

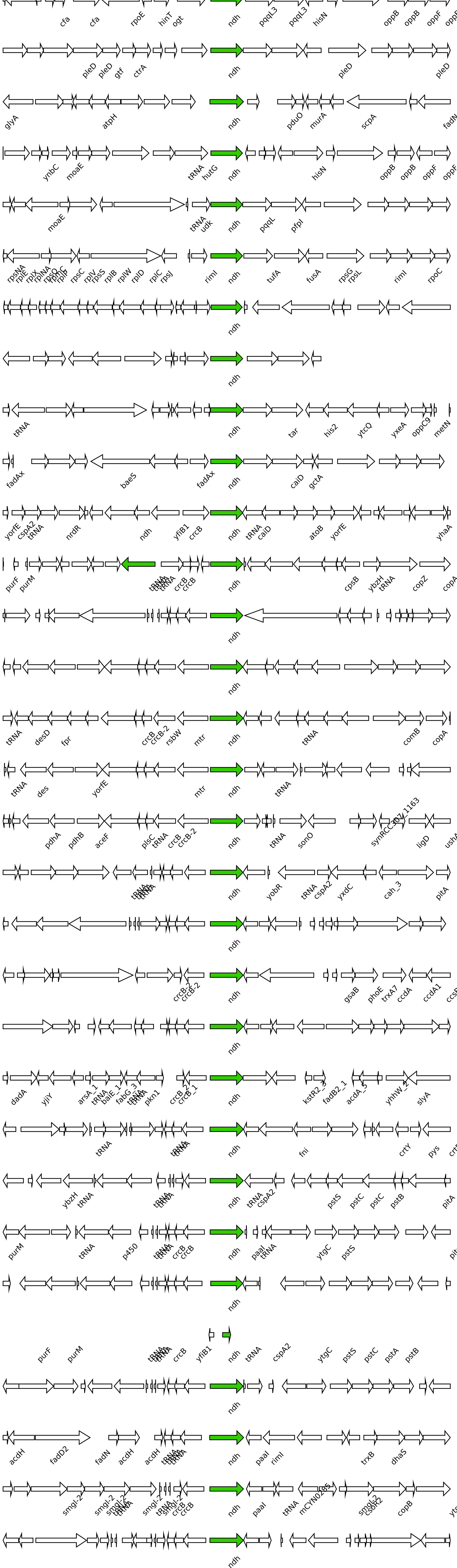

Nocardioides sp URHA0020
KK211169.1_17 (1380392)
KK211169.1 ()

Nocardioides sp Leaf307
KQQ42882.1 (1736331)
LMNY01000001.1 (genomic DNA)

Nocardioides pyridinolyticus JCM 10369
BBGV01000578.1_2 (1305854)
BBGV01000578.1 ()

Nocardioides sp 603
CP033324.1_3483 (2483798)
CP033324.1 ()

Nocardioides sp CCTCC AB 2018079
PWN01157.1 (2201891)
QGDD01000011.1 (genomic DNA)

Nocardioides nitrophenolicus JCM 10703
BBGE01000683.1_1 (1303688)
BBGE01000683.1 ()

Nocardioides sp Root190
KRB77676.1 (1736488)
LMIA01000008.1 (genomic DNA)

Nocardioides sp CF167
PTX35802.1 (2135685)
QBKO01000020.1 (genomic DNA)

Nocardioides gilvus
QHKY01000001.1_993 (1735589)
QHKY01000001.1 ()

Nocardioides sp 78
CP025581.1_538 (2045452)
CP025581.1 ()

Nocardioides solisilvae
QHKX01000001.1_1111 (1542435)
QHKX01000001.1 ()

Marmoricola pocheonensis
RNM14770.1 (661485)
RJSF01000037.1 (genomic DNA)

Marmoricola ginsengisoli
RNL60380.1 (661483)
RJSE01000009.1 (genomic DNA)

Marmoricola solisilvae
RNL77539.1 (1542737)
RJSG01000003.1 (genomic DNA)

Nocardioides halotolerans DSM 19273
KE383924.1_17 (1122609)
KE383924.1 ()

Nocardioides massiliensis
CCXJ01000767.1_1 (1325935)
CCXJ01000767.1 ()

Nocardioides insulae DSM 17944
KE383928.1_477 (1122610)
KE383928.1 ()

Nocardioides sp Iso805N
KB822577.1_422 (1283287)
KB822577.1 ()

Nocardioides baekrokdamisoli
BBH17178.1 (1804624)
AP019307.1 (genomic DNA)

Geodermatophilus obscurus DSM 43160
ADB76427.1 (526225)
CP001867.1 (chromosome)

Propionicicella superfundia DSM 22317
KE384020.1_107 (1122999)
KE384020.1 ()

Branchiibius hedensis
PWJ24400.1 (672460)
QGDN01000001.1 (genomic DNA)

Luteipulveratus mongoliensis
AKU17585.1 (571913)
CP011112.1 (chromosome)

Demetria terragena DSM 11295
AQXW01000004.1_1018 (1121385)
AQXW01000004.1 ()

Janibacter corallicola NBRC 107790
BCSR01000023.1_19 (1216969)
BCSR01000023.1 ()

Janibacter indicus
APH00772.1 (857417)
CP013290.1 (chromosome)

Janibacter melonis
OAB86547.1 (262209)
LQZG01000004.1 (genomic DNA)

Flexivirga sp BO 16
RNI21142.1 (2294115)
RJJQ01000012.1 (genomic DNA)

Luteococcus japonicus
ROR54476.1 (33984)
RKHG01000001.1 (genomic DNA)

Propionibacteriaceae bacterium UBA4569
DGKN01000134.1_25 (1952960)
DGKN01000134.1 ()

Micropruina glycogenica
SPD85393.1 (75385)
LT985188.1 (genomic DNA)

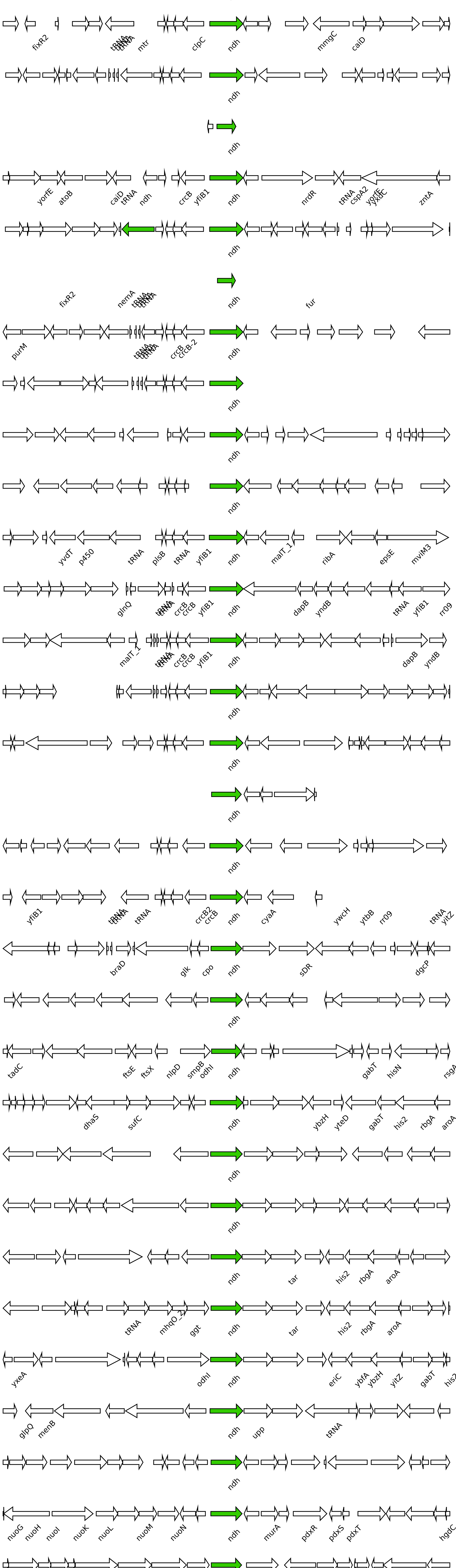

Actinobacteria bacterium HGW Actinobacteria 5
PKQ24367.1 (2013650)
PHEV01000014.1 (genomic DNA)

Frankineae bacterium MT45
SDI93481.1 (1882833)
LT629697.1 (genomic DNA)

Jatrophihabitans endophyticus
SHH27938.1 (1206085)
FQVU01000005.1 (genomic DNA)

Dermatophilus congolensis
SNV24017.1 (1863)
LT906453.1 (genomic DNA)

Rhodococcus sp PBTS 1
AMY21303.1 (1653478)
CP015219.1 (chromosome)

Hoyosella subflava DQS3 9A1
AEF41563.1 (443218)
CP002786.1 (chromosome)

Hoyosella subflava DQS3 9A1
AEF40912.1 (443218)
CP002786.1 (chromosome)

Tomitella biformata AHU 1821
BAVQ01000079.1_44 (1448389)
BAVQ01000079.1 ()

Rhodococcus coprophilus
SQI28632.1 (38310)
LS483468.1 (genomic DNA)

Rhodococcus sp WMMA185
AOW92631.1 (679318)
CP017014.1 (chromosome)

Rhodococcus tukisamuensis
SDD44856.1 (168276)
FNAB01000004.1 (genomic DNA)

Rhodococcus hoagii 103S
CBH48447.1 (685727)
FN563149.1 (genomic DNA)

Rhodococcus defluvii
JPOC01000008.1_155 (1323361)
JPOC01000008.1 ()

Rhodococcus rhodnii LMG 5362
EOM75053.1 (1273125)
APMY01000109.1 (genomic)

Millisia brevis NBRC 105863
BCRN01000006.1_225 (1219361)
BCRN01000006.1 ()

Rhodococcus sp PBTS 1
AMY19178.1 (1653478)
CP015219.1 (chromosome)

Rhodococcus sp PBTS 2
AMY25342.1 (1653479)
CP015220.1 (chromosome)

Nocardia sp CNY236
AXVD01000004.1_227 (1169152)
AXVD01000004.1 ()

Tsukamurella paurometabola DSM 20162
ADG78917.1 (521096)
CP001966.1 (chromosome)

Skermania piniformis NBRC 15059
BCRV01000029.1_4 (1223536)
BCRV01000029.1 ()

Williamsia sterculiae
SIR69700.1 (1344003)
FTNT01000001.1 (genomic DNA)

Mycobacteriaceae bacterium 1482268 1
OBB13122.1 (1834080)
LZSH01000062.1 (genomic DNA)

Mycobacterium shimoidei
ODR13411.1 (29313)
MIHE01000009.1 (genomic DNA)

Mycobacterium xenopi
ORX21902.1 (1789)
LQQB01000032.1 (genomic DNA)

Mycobacterium tuberculosis H37Rv
CCP43122.1 (83332)
AL123456.3 (genomic DNA)

Mycobacteroides abscessus ATCC 19977
CAM62510.1 (561007)
CU458896.1 (genomic DNA)

Mycobacteriaceae bacterium 1482268 1
OBA89781.1 (1834080)
LZSH01000453.1 (genomic DNA)

Mycolicibacterium hassiacum DSM 44199
EKF23638.1 (1122247)
AMRA01000059.1 (genomic DNA)

Mycobacterium lehmannii
KUI20923.1 (2048550)
LQIR01000001.1 (genomic DNA)

Mycolicibacterium elephantis
KKW62465.1 (81858)
LBNO01000120.1 (genomic DNA)

[illegible]

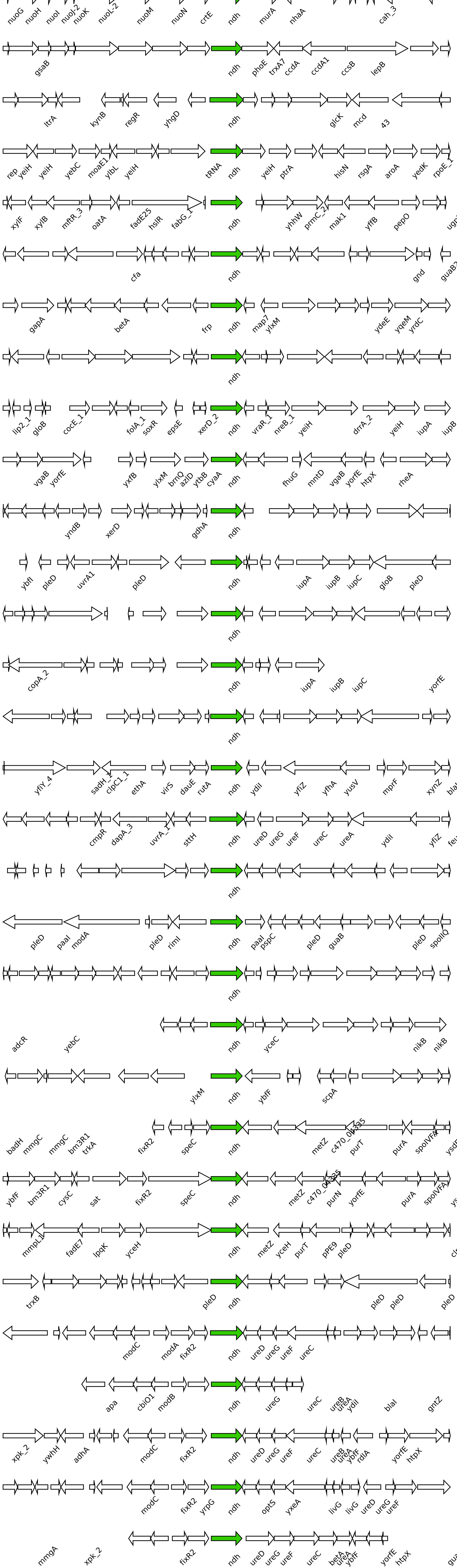

Mycobacterium sp 852013 51886_SCH5428379
OBB55678.1 (1834111)
LZSU01000052.1 (genomic DNA)

Mycobacterium sp 141
KB908273.1_63 (1120797)
KB908273.1 ()

Mycolicibacterium neoaurum VKM Ac 1815D
AHC25944.1 (700508)
CP006936.2 (chromosome)

Mycolicibacterium thermoresistibile ATCC 19527
EHI10937.1 (1078020)
AGVE01000050.1 (genomic DNA)

Mycobacterium shimoidei
ODR13264.1 (29313)
MIHE01000010.1 (genomic DNA)

Mycobacterium xenopi
ORX19500.1 (1789)
LQQB01000106.1 (genomic DNA)

Mycobacterium ulcerans Agy99
ABL05277.1 (362242)
CP000325.1 (chromosome)

Mycobacterium tuberculosis H37Rv
CCP44620.1 (83332)
AL123456.3 (genomic DNA)

Mycobacterium leprae TN
CAC31016.1 (272631)
AL450380.1 (genomic DNA)

Mycobacterium lepromatosis
KJX74787.1 (480418)
JRPY01000089.1 (genomic)

Mycolicibacter sinensis
AEF35943.1 (875328)
CP002329.1 (chromosome)

Mycobacterium sp 1274756 6
OBJ69922.1 (1834076)
LZLJ01000072.1 (genomic DNA)

Mycolicibacillus koreensis
ODR11462.1 (1069220)
MIHD01000002.1 (genomic DNA)

Mycolicibacterium fallax
ORV03474.1 (1793)
LQOJ01000037.1 (genomic DNA)

Gordonia amarae NBRC 15530
GAB06308.1 (1075090)
BAED01000050.1 (genomic DNA)

Gordonia kroppenstedtii DSM 45133
AQYG01000035.1_20 (468556)
AQYG01000035.1 ()

Williamsia sp D3
ETD32687.1 (1313067)
AYTE01000018.1 (genomic DNA)

Williamsia sp Leaf354
KQR98479.1 (1736349)
LMPL01000002.1 (genomic DNA)

Gordonia sp UBA6683
DKJV01000073.1_108 (1946577)
DKJV01000073.1 ()

Gordonia amarae NBRC 15530
GAB03668.1 (1075090)
BAED01000003.1 (genomic DNA)

Gordonia sp
MAU84903.1 (84139)
PACQ01000073.1 (genomic DNA)

Gordonia bronchialis DSM 43247
ACY21968.1 (526226)
CP001802.1 (chromosome)

Gordonia soli NBRC 108243
GAC69671.1 (1223545)
BANX01000026.1 (genomic DNA)

Gordonia effusa NBRC 100432
GAB20027.1 (1077974)
BAEH01000105.1 (genomic DNA)

Kroppenstedtia sanguinis
RQM42955.1 (1380684)
QWDJ01000002.1 (genomic DNA)

Gordonia araii NBRC 100433
GAB09201.1 (1073574)
BAEE01000033.1 (genomic DNA)

Gordonia sp UBA7599
DLKD01000088.1_32 (1946578)
DLKD01000088.1 ()

Gordonia sp UBA5067
DIAX01000072.1_10 (1946575)
DIAX01000072.1 ()

Gordonia phthalatica
ALG84958.1 (1136941)
CP011853.1 (chromosome)

Gordonia iterans
AVM00819.1 (1004901)
CP027433.1 (chromosome)

Williamsia sterculiae
SIS19054.1 (1344003)
FTNT01000011.1 (genomic DNA)

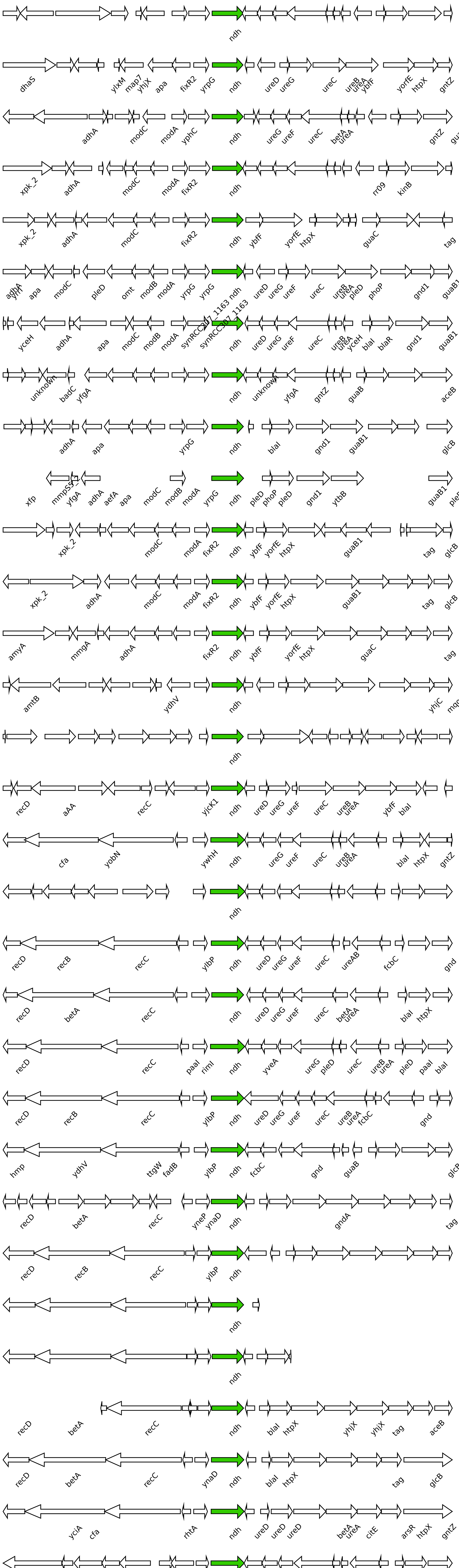

Gordonia soli NBRC 108243
GAC68633.1 (1223545)
BANX01000017.1 (genomic DNA)

Gordonia bronchialis DSM 43247
ACY21632.1 (526226)
CP001802.1 (chromosome)

Dietzia timorensis
LMTB01000003.1_32 (499555)
LMTB01000003.1 ()

Dietzia timorensis
LMTB01000006.1_227 (499555)
LMTB01000006.1 ()

Dietzia lutea
AWH94065.1 (546160)
CP015449.1 (chromosome)

Dietzia sp UBA5065
DIAZ01000165.1_19 (1946422)
DIAZ01000165.1 ()

Dietzia lutea
AWH92034.1 (546160)
CP015449.1 (chromosome)

Segniliparus rotundus DSM 44985
ADG98232.1 (640132)
CP001958.1 (chromosome)

Cellulomonas sp UBA6250
DJVS01000095.1_76 (1946310)
DJVS01000095.1 ()

Ornithinimicrobium sp AMA3305
AXH96221.1 (2283195)
CP031229.1 (chromosome)

Serinicoccus sp JLT9
ANS79959.1 (1758689)
CP014989.1 (chromosome)

Ornithinimicrobium pekingense
SOC57192.1 (384677)
OBQK01000011.1 (genomic DNA)

Actinotalea ferrariae CF5 4
EYR64004.1 (948458)
AXCW01000055.1 (genomic DNA)

Cellulomonas sp PhB150
ROS27880.1 (2485188)
RKHX01000002.1 (genomic DNA)

Cellulomonas sp NEAU YY56
RMI09502.1 (2483352)
RFFI01000047.1 (genomic DNA)

Cellulomonas denverensis JCM 14733
BBHK01001098.1_1 (1305869)
BBHK01001098.1 ()

Cellulomonas timonensis
FCOT01000013.1_566 (1689271)
FCOT01000013.1 ()

Nigerium massiliense
CCYM01000002.1_419 (1522317)
CCYM01000002.1 ()

Corynebacterium kroppenstedtii DSM 44385
ACR17633.1 (645127)
CP001620.1 (chromosome)

Corynebacterium provencense
AWT26263.1 (1737425)
CP024988.1 (chromosome)

Corynebacterium glyciniphilum AJ 3170
AHW64247.1 (1404245)
CP006842.1 (chromosome)

Corynebacterium bovis
RRO92631.1 (36808)
PQNE01000005.1 (genomic DNA)

Corynebacterium resistens DSM 45100
AEI09355.1 (662755)
CP002857.1 (chromosome)

Corynebacterium jeikeium K411
CAI37078.1 (306537)
CR931997.1 (genomic DNA)

bacterium UBA5646
DIJK01000125.1_21 (1947810)
DIJK01000125.1 ()

Corynebacterium urealyticum DSM 7109
CAQ05023.1 (504474)
AM942444.1 (genomic DNA)

Corynebacterium massiliense DSM 45435
ATVG01000001.1_17 (1121364)
ATVG01000001.1 ()

Corynebacterium segmentosum
VEH72898.1 (43990)
LR134408.1 (genomic DNA)

Corynebacterium aurimucosum ATCC 700975
ACP32884.1 (548476)
CP001601.1 (chromosome)

Corynebacterium camporealensis
AVH88355.1 (161896)
CP027001.1 (chromosome)

Corynebacterium sp DNF00584

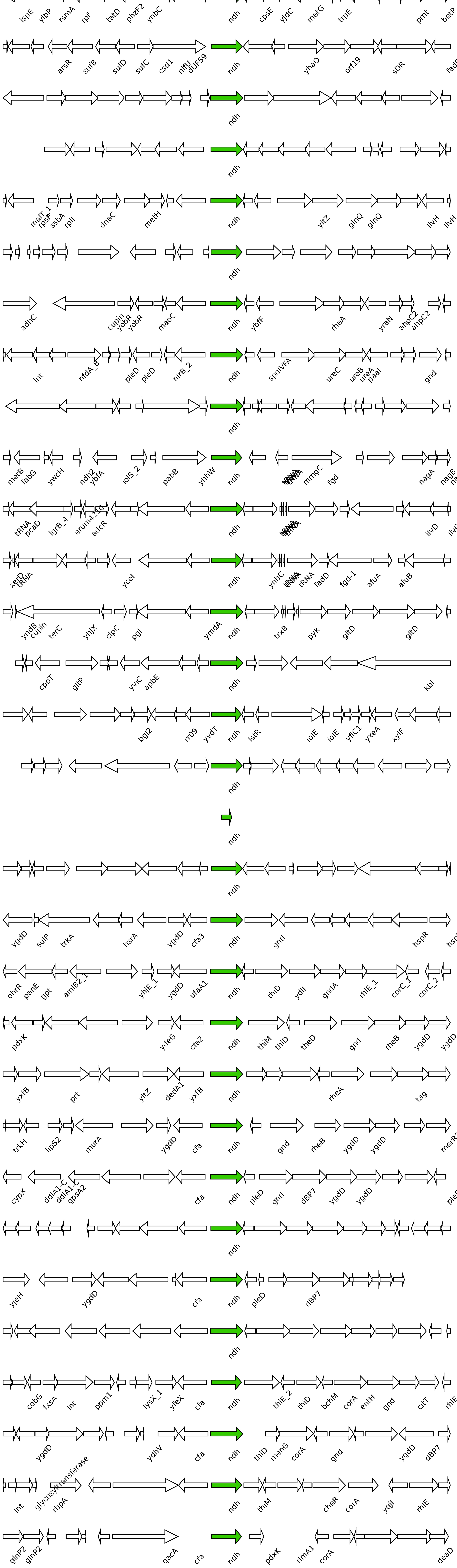

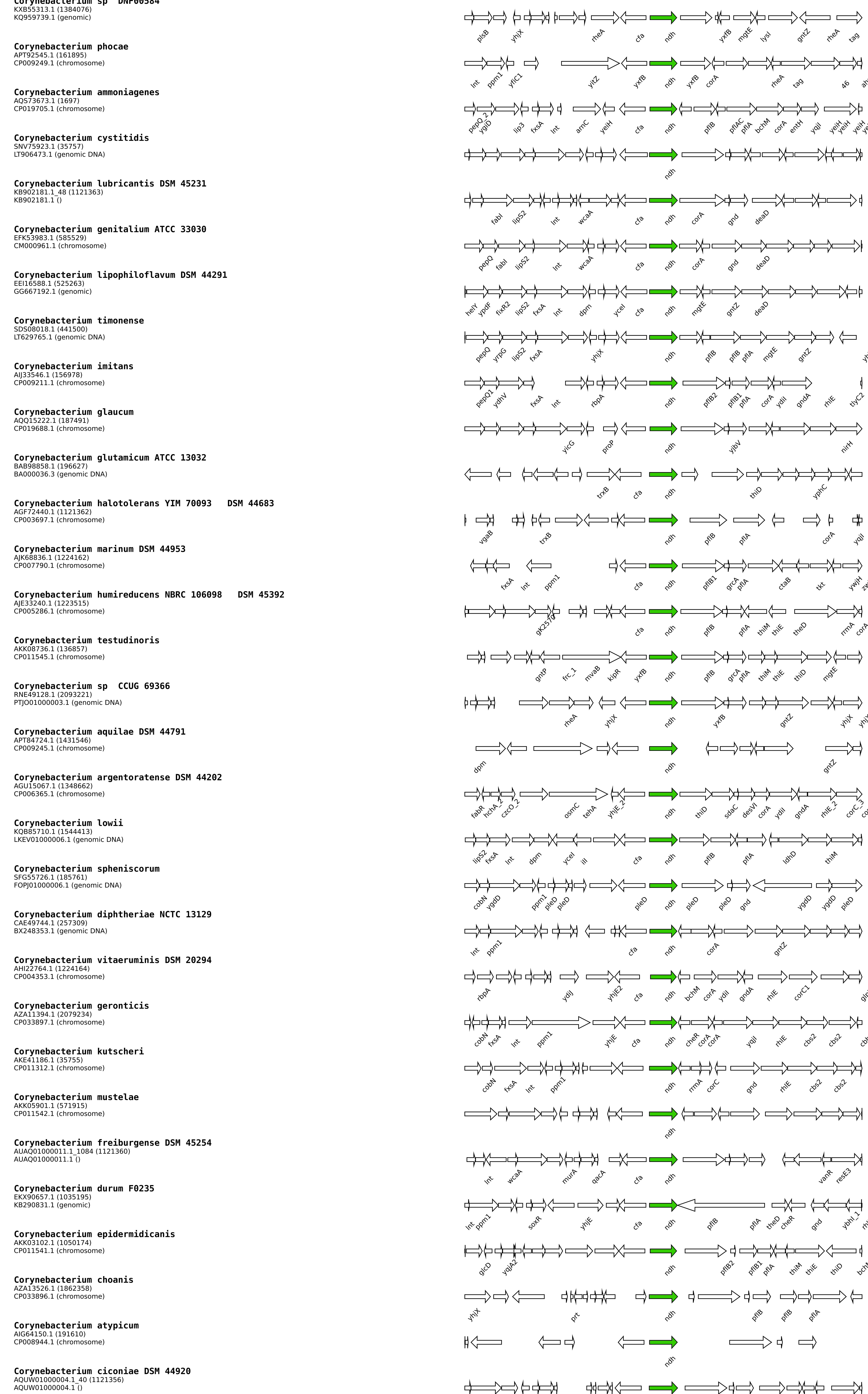

Corynebacterium sp DNF00584
KXB55313.1 (1384076)
KQ959739.1 (genomic)
Corynebacterium phocae
APT92545.1 (161895)
CP009249.1 (chromosome)
Corynebacterium ammoniagenes
AQS73673.1 (1697)
CP019705.1 (chromosome)
Corynebacterium cystitidis
SNV75923.1 (35757)
LT906473.1 (genomic DNA)
Corynebacterium lubricantis DSM 45231
KB902181.1_48 (1121363)
KB902181.1 ()
Corynebacterium genitalium ATCC 33030
EFK53983.1 (585529)
CM000961.1 (chromosome)
Corynebacterium lipophiloflavum DSM 44291
EEI16588.1 (525263)
GG667192.1 (genomic)
Corynebacterium timonense
SDS08018.1 (441500)
LT629765.1 (genomic DNA)
Corynebacterium imitans
AIJ33546.1 (156978)
CP009211.1 (chromosome)
Corynebacterium glaucum
AQQ15222.1 (187491)
CP019688.1 (chromosome)
Corynebacterium glutamicum ATCC 13032
BAB98858.1 (196627)
BA000036.3 (genomic DNA)
Corynebacterium halotolerans YIM 70093 DSM 44683
AGF72440.1 (1121362)
CP003697.1 (chromosome)
Corynebacterium marinum DSM 44953
AJK68836.1 (1224162)
CP007790.1 (chromosome)
Corynebacterium humireducens NBRC 106098 DSM 45392
AJE33240.1 (1223515)
CP005286.1 (chromosome)
Corynebacterium testudinoris
AKK08736.1 (136857)
CP011545.1 (chromosome)
Corynebacterium sp CCUG 69366
RNE49128.1 (2093221)
PTJO01000003.1 (genomic DNA)
Corynebacterium aquilae DSM 44791
APT84724.1 (1431546)
CP009245.1 (chromosome)
Corynebacterium argentoratense DSM 44202
AGU15067.1 (1348662)
CP006365.1 (chromosome)
Corynebacterium lowii
KQB85710.1 (1544413)
LKEV01000006.1 (genomic DNA)
Corynebacterium spheniscorum
SFG55726.1 (185761)
FOPJ01000006.1 (genomic DNA)
Corynebacterium diphtheriae NCTC 13129
CAE49744.1 (257309)
BX248353.1 (genomic DNA)
Corynebacterium vitaeruminis DSM 20294
AHI22764.1 (1224164)
CP004353.1 (chromosome)
Corynebacterium geronticis
AZA11394.1 (2079234)
CP033897.1 (chromosome)
Corynebacterium kutscheri
AKE41186.1 (35755)
CP011312.1 (chromosome)
Corynebacterium mustelae
AKK05901.1 (571915)
CP011542.1 (chromosome)
Corynebacterium freiburgense DSM 45254
AUAQ01000011.1_1084 (1121360)
AUAQ01000011.1 ()
Corynebacterium durum F0235
EKX90657.1 (1035195)
KB290831.1 (genomic)
Corynebacterium epidermidicanis
AKK03102.1 (1050174)
CP011541.1 (chromosome)
Corynebacterium choanis
AZA13526.1 (1862358)
CP033896.1 (chromosome)
Corynebacterium atypicum
AIG64150.1 (191610)
CP008944.1 (chromosome)
Corynebacterium ciconiae DSM 44920
AQUW01000004.1_40 (1121356)
AQUW01000004.1 ()

Corynebacterium sp 2183
AWB81922.1 (2080740)
CP026947.1 (chromosome)

Corynebacterium uterequi
AKK11064.1 (1072256)
CP011546.1 (chromosome)

Corynebacterium ulceribovis DSM 45146
AQUY01000001.1_1046 (1121370)
AQUY01000001.1 ()

Corynebacterium sp HMSC11E11
OFU53624.1 (1581089)
KV787152.1 (genomic)

Corynebacterium sphenisci DSM 44792
APT90818.1 (1437874)
CP009248.1 (chromosome)

Corynebacterium amycolatum
ODQ40354.1 (43765)
MIFV01000034.1 (genomic DNA)

Corynebacterium sputi DSM 45148
ATYV01000022.1_4 (1111729)
ATYV01000022.1 ()

Corynebacterium caspium DSM 44850
KB903375.1_35 (1121355)
KB903375.1 ()

Corynebacterium doosanense CAU 212 DSM 45436
AQUX01000003.1_29 (558173)
AQUX01000003.1 ()

Corynebacterium renale
LDYE01000011.1_331 (1724)
LDYE01000011.1 ()

Corynebacterium sp KPL1989
ERS71874.1 (1203622)
KI515717.1 (genomic)

Corynebacterium otitidis ATCC 51513
EJZ82396.1 (883169)
JH815193.1 (genomic)

Corynebacterium maris DSM 45190
AGS34809.1 (1224163)
CP003924.1 (chromosome)

Corynebacterium renale
LDYE01000011.1_332 (1724)
LDYE01000011.1 ()

Dietzia timorensis
LMTB01000164.1_19 (499555)
LMTB01000164.1 ()

Kytococcus aerolatus
SNC61901.1 (592308)
FYEZ01000001.1 (genomic DNA)

Kytococcus sedentarius DSM 20547
ACV06905.1 (478801)
CP001686.1 (chromosome)

Raineyella antarctica
SDB89730.1 (1577474)
FMYF01000007.1 (genomic DNA)

Nocardioides nitrophenolicus JCM 10703
BBGE01000464.1_2 (1303688)
BBGE01000464.1 ()

Sorangiineae bacterium NIC37A_2
OQX68702.1 (1970199)
NASX01000016.1 (genomic DNA)

Verrucomicrobium sp 3C
KB901875.1_748 (1134055)
KB901875.1 ()

Methylacidiphilum infernorum V4
ACD83662.1 (481448)
CP000975.1 (chromosome)

Verrucomicrobia bacterium LP2A
JAFS01000001.1_663 (478741)
JAFS01000001.1 ()

Bdellovibrionales bacterium CG10_big_fil_rev_8_21_14_0_10_45_34
PIT99606.1 (1973927)
PEZS01000011.1 (genomic DNA)

Bacteriovoracaceae bacterium UBA923
DBDV01000025.1_59 (1961501)
DBDV01000025.1 ()

Bdellovibrionaceae bacterium UBA3535
DFOS01000015.1_58 (1961513)
DFOS01000015.1 ()

Legionella israelensis
KTD32010.1 (454)
LNYH01000017.1 (genomic DNA)

Legionella tunisiensis
CALJ01000240.1_15 (1034944)
CALJ01000240.1 ()

Legionella spiritensis
KTD61728.1 (452)
LNYX01000031.1 (genomic DNA)

Legionella pneumophila subsp pneumophila str Philadelphia 1
AAU26760.1 (272624)
AE017354.1 (chromosome)

Legionella fallonii LLAP 10

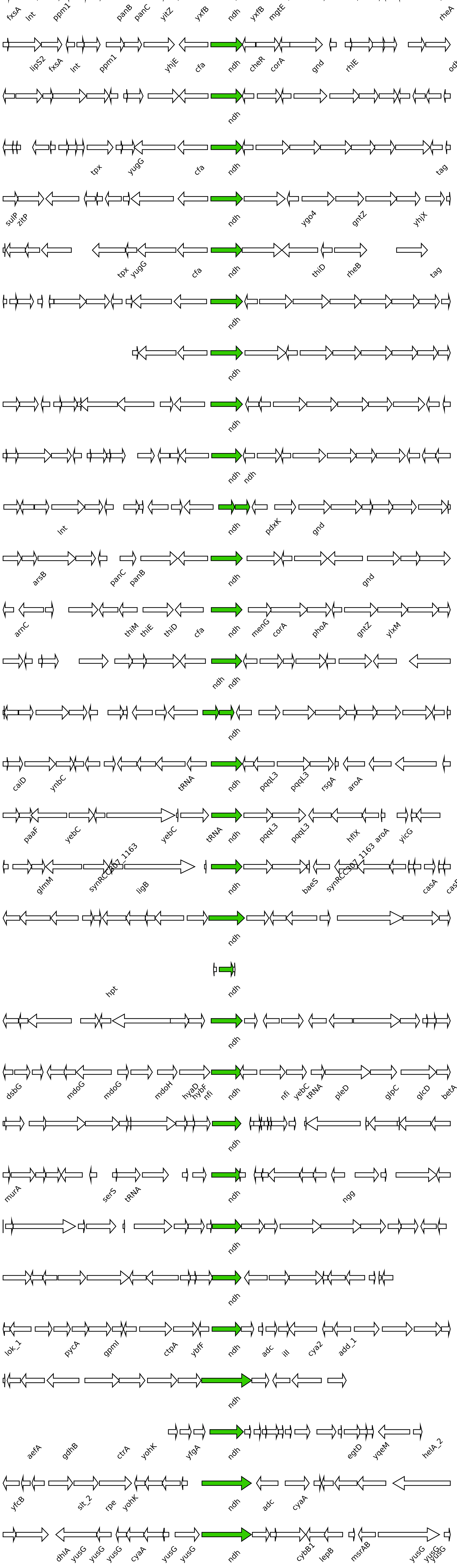

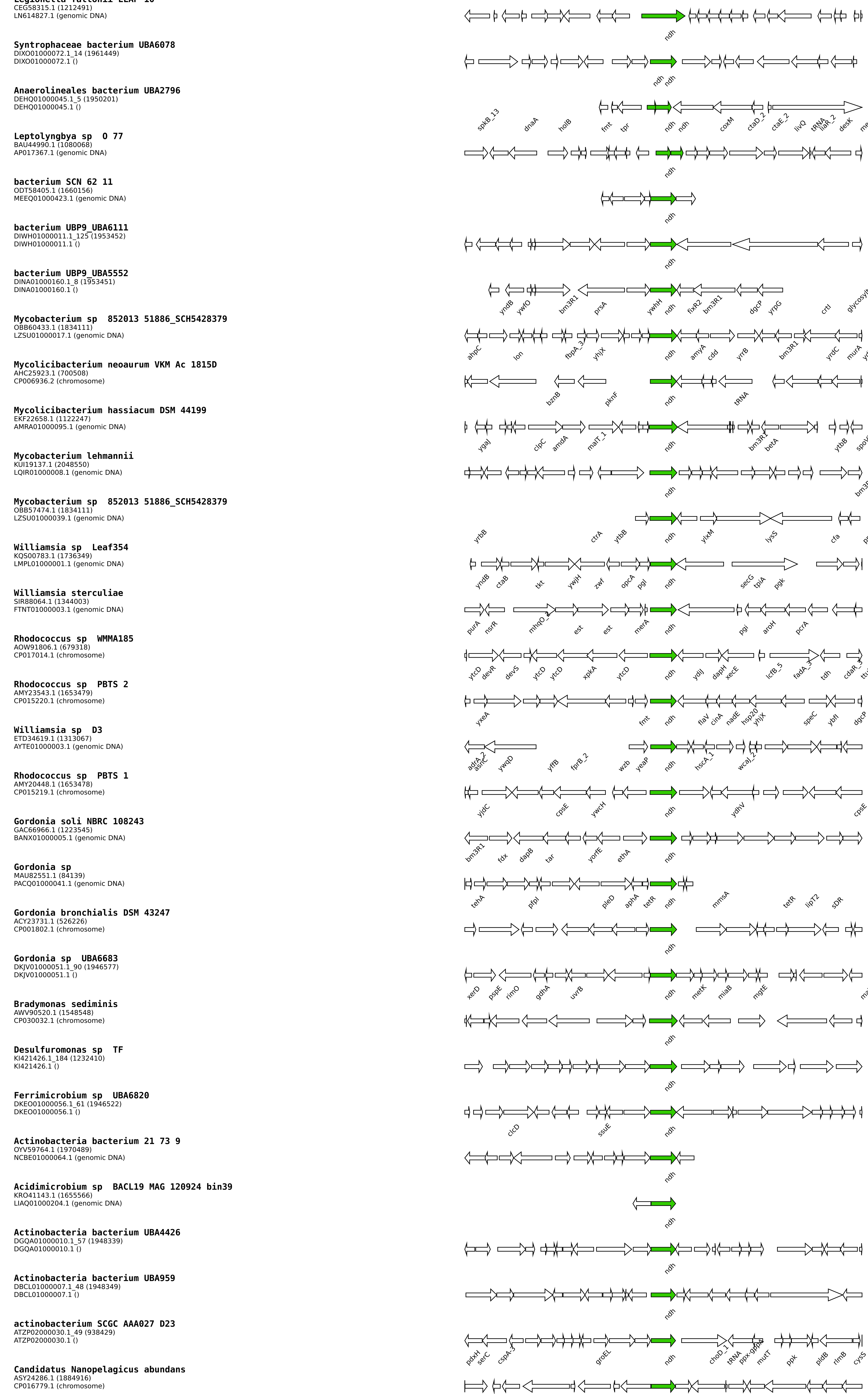
Legionella fallonii LLAP 10
CEG58315.1 (1212491)
LN614827.1 (genomic DNA)
Syntrophaceae bacterium UBA6078
DIXO01000072.1_14 (1961449)
DIXO01000072.1 ()
Anaerolineales bacterium UBA2796
DEHQ01000045.1_5 (1950201)
DEHQ01000045.1 ()
Leptolyngbya sp O 77
BAU44990.1 (1080068)
AP017367.1 (genomic DNA)
bacterium SCN 62 11
ODT58405.1 (1660156)
MEEQ01000423.1 (genomic DNA)
bacterium UBP9_UBA6111
DIWH01000011.1_125 (1953452)
DIWH01000011.1 ()
bacterium UBP9_UBA5552
DINA01000160.1_8 (1953451)
DINA01000160.1 ()
Mycobacterium sp 852013 51886_SCH5428379
OBB60433.1 (1834111)
LZSU01000017.1 (genomic DNA)
Mycolicibacterium neoaurum VKM Ac 1815D
AHC25923.1 (700508)
CP006936.2 (chromosome)
Mycolicibacterium hassiacum DSM 44199
EKF22658.1 (1122247)
AMRA01000095.1 (genomic DNA)
Mycobacterium lehmannii
KUI19137.1 (2048550)
LQIR01000008.1 (genomic DNA)
Mycobacterium sp 852013 51886_SCH5428379
OBB57474.1 (1834111)
LZSU01000039.1 (genomic DNA)
Williamsia sp Leaf354
KQS00783.1 (1736349)
LMPL01000001.1 (genomic DNA)
Williamsia sterculiae
SIR88064.1 (1344003)
FTNT01000003.1 (genomic DNA)
Rhodococcus sp WMMA185
AOW91806.1 (679318)
CP017014.1 (chromosome)
Rhodococcus sp PBTS 2
AMY23543.1 (1653479)
CP015220.1 (chromosome)
Williamsia sp D3
ETD34619.1 (1313067)
AYTE01000003.1 (genomic DNA)
Rhodococcus sp PBTS 1
AMY20448.1 (1653478)
CP015219.1 (chromosome)
Gordonia soli NBRC 108243
GAC66966.1 (1223545)
BANX01000005.1 (genomic DNA)
Gordonia sp
MAU82551.1 (84139)
PACQ01000041.1 (genomic DNA)
Gordonia bronchialis DSM 43247
ACY23731.1 (526226)
CP001802.1 (chromosome)
Gordonia sp UBA6683
DKJV01000051.1_90 (1946577)
DKJV01000051.1 ()
Bradymonas sediminis
AWV90520.1 (1548548)
CP030032.1 (chromosome)
Desulfuromonas sp TF
KI421426.1_184 (1232410)
KI421426.1 ()
Ferrimicrobium sp UBA6820
DKEO01000056.1_61 (1946522)
DKEO01000056.1 ()
Actinobacteria bacterium 21 73 9
OYV59764.1 (1970489)
NCBE01000064.1 (genomic DNA)
Acidimicrobium sp BACL19 MAG 120924 bin39
KRO41143.1 (1655566)
LIAQ01000204.1 (genomic DNA)
Actinobacteria bacterium UBA4426
DGQA01000010.1_57 (1948339)
DGQA01000010.1 ()
Actinobacteria bacterium UBA959
DBCL01000007.1_48 (1948349)
DBCL01000007.1 ()
actinobacterium SCGC AAA027 D23
ATZP02000030.1_49 (938429)
ATZP02000030.1 ()
Candidatus Nanopelagicus abundans
ASY24286.1 (1884916)
CP016779.1 (chromosome)

Actinobacteria bacterium UBA7398
DKNC01000067.1_13 (1948346)
DKNC01000067.1 ()

Clavibacter sp
GBL22420.1 (1871044)
BGOH01000001.1 (genomic DNA)

Actinobacteria bacterium UBA3066
DFCA01000012.1_14 (1948336)
DFCA01000012.1 ()

Candidatus Planktophila limnetica
ASY27223.1 (573600)
CP016782.1 (chromosome)

Candidatus Planktophila vernalis
ASY20448.1 (1884907)
CP016776.1 (chromosome)

Candidatus Planktophila dulcis
ASY11491.1 (1884914)
CP016770.1 (chromosome)

Actinobacteria bacterium IMCC26077
CP015606.1_62 (1848755)
CP015606.1 ()

Ignavibacteriales bacterium CG12_big_fil_rev_8_21_14_0_65_30_8
PIW70566.1 (1974046)
PFGS01000040.1 (genomic DNA)

Chlamydiae bacterium RIFCSPHIGHO2_12_FULL_49_11
OGN63031.1 (1797603)
MGLW01000005.1 (genomic DNA)

Deltaproteobacteria bacterium HGW Deltaproteobacteria 8
PKN08790.1 (2013758)
PHAR01000003.1 (genomic DNA)

Anaerolineae bacterium UTCFX5
OQY84205.1 (1961103)
MWSX01000106.1 (genomic DNA)

Lentisphaerae bacterium GWF2_38_69
OGV19509.1 (1798570)
MHBE01000032.1 (genomic DNA)

Spartobacteria bacterium UBA695
DBMP01000006.1_202 (1948752)
DBMP01000006.1 ()

Actinobacteria bacterium IMCC26207
KLR60699.1 (1641811)
LCZK01000009.1 (genomic DNA)

Bacteroidetes bacterium UBA2412
DDRQ01000024.1_5 (1950764)
DDRQ01000024.1 ()

Acidiferrobacter sp SPIII_3
AWP23590.1 (1281578)
CP027663.1 (chromosome)

Acidithiobacillus sp CJ 2
RNF72825.1 (1867749)
RIZI01000081.1 (genomic DNA)

Acidithiobacillus ferrooxidans ATCC 23270
ACK79177.1 (243159)
CP001219.1 (chromosome)

Acidithiobacillus sp UBA2486
DDOU01000038.1_7 (1945924)
DDOU01000038.1 ()

Mycobacterium sp 141
KB908256.1_321 (1120797)
KB908256.1 ()

Mycolicibacillus koreensis
ODR10225.1 (1069220)
MIHD01000005.1 (genomic DNA)

Mycobacterium sp 1274756 6
OBJ72024.1 (1834076)
LZLJ01000044.1 (genomic DNA)

Tomitella biformata AHU 1821
BAVQ01000002.1_53 (1448389)
BAVQ01000002.1 ()

Tomitella biformata AHU 1821
BAVQ01000002.1_17 (1448389)
BAVQ01000002.1 ()

Microbacterium humi
SEC59672.1 (640635)
FNRY01000002.1 (genomic DNA)

Salinibacterium sp CGMCC 1 16371
AWB88725.1 (2079791)
CP026951.1 (chromosome)

Promicromonosporaceae bacterium W15
DF158873.1_997 (1214064)
DF158873.1 ()

Sinomonas atrocyanea
AMM34059.1 (37927)
CP014518.1 (chromosome)

Ornithinimicrobium sp AMA3305
AXH97054.1 (2283195)
CP031229.1 (chromosome)

Synechococcus sp MW101C3
NQKX01000007.1_195 (210768)
NQKX01000007.1 ()

Synechococcales bacterium UBA8138

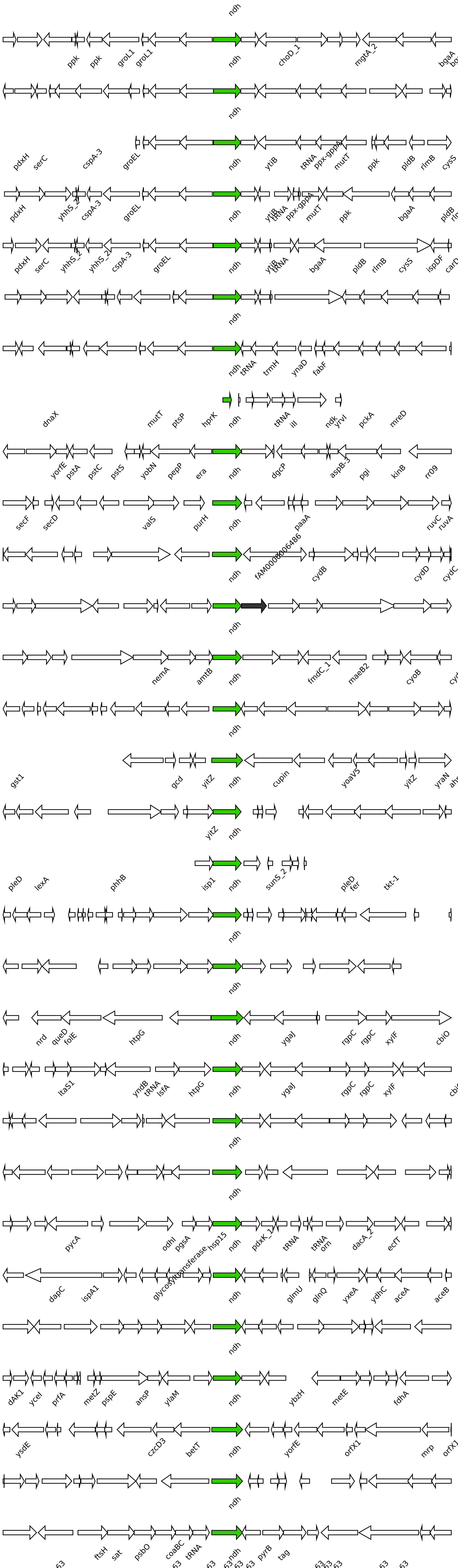

[illegible]
HBU27419.1 (2055796)
DOMQ01000199.1 (genomic DNA)

Synechococcus sp RCC307
CAK29106.1 (316278)
CT978603.1 (genomic DNA)

Cyanobacteria bacterium UBA7373
DKOB01000065.1_3 (1947895)
DKOB01000065.1 ()

Synechococcus sp CPC35
MAB55428.1 (2024880)
NYUU01000097.1 (genomic DNA)

Synechococcus sp WH 8102
CAE06812.1 (84588)
BX548020.1 (genomic DNA)

Synechococcus sp CC9311
ABI47422.1 (64471)
CP000435.1 (chromosome)

Prochlorococcus marinus str MIT 9313
CAE21981.1 (74547)
BX548175.1 (genomic DNA)

Cyanobium gracile PCC 6307
AFY29884.1 (292564)
CP003495.1 (chromosome)

Synechococcus sp MW101C3
NQKX01000012.1_33 (210768)
NQKX01000012.1 ()

Cyanobium gracile PCC 6307
AFY28276.1 (292564)
CP003495.1 (chromosome)

Rhodococcus sp PBTS 2
AMY21700.1 (1653479)
CP015220.1 (chromosome)

Rhodococcus rhodnii LMG 5362
EOM74457.1 (1273125)
APMY01000131.1 (genomic)

Rhodococcus coprophilus
SQI34700.1 (38310)
LS483468.1 (genomic DNA)

Rhodococcus sp WMMA185
AOW93560.1 (679318)
CP017014.1 (chromosome)

Rhodococcus tukisamuensis
SDC59060.1 (168276)
FNAB01000001.1 (genomic DNA)

Rhodococcus defluvii
JPOC01000037.1_52 (1323361)
JPOC01000037.1 ()

Rhodococcus hoagii 103S
CBH46528.1 (685727)
FN563149.1 (genomic DNA)

Tomitella biformata AHU 1821
BAVQ01000043.1_83 (1448389)
BAVQ01000043.1 ()

Skermania piniformis NBRC 15059
BCRV01000007.1_91 (1223536)
BCRV01000007.1 ()

Nocardia sp CNY236
AXVD01000003.1_45 (1169152)
AXVD01000003.1 ()

Nocardia donostiensis
ONM49263.1 (1538463)
MUMY01000005.1 (genomic)

Nocardia jinanensis NBRC 108249
LNDA01000069.1_26 (1210082)
LNDA01000069.1 ()

Williamsia sp D3
ETD34766.1 (1313067)
AYTE01000001.1 (genomic DNA)

Gordonia kroppenstedtii DSM 45133
AQYG01000050.1_15 (468556)
AQYG01000050.1 ()

Williamsia sp Leaf354
KQS00218.1 (1736349)
LMPL01000001.1 (genomic DNA)

Tsukamurella paurometabola DSM 20162
ADG77954.1 (521096)
CP001966.1 (chromosome)

Gordonia iterans
AVM01477.1 (1004901)
CP027433.1 (chromosome)

Gordonia effusa NBRC 100432
GAB17687.1 (1077974)
BAEH01000036.1 (genomic DNA)

Williamsia sterculiae
SIR94612.1 (1344003)
FTNT01000004.1 (genomic DNA)

Actinobacteria bacterium Ga0077560
LNFL01000163.1_77 (1752190)
LNFL01000163.1 ()

Phycisphaeraceae bacterium TMED231
OUX03404.1 (1986831)
NHKC01000003.1 (genomic DNA)

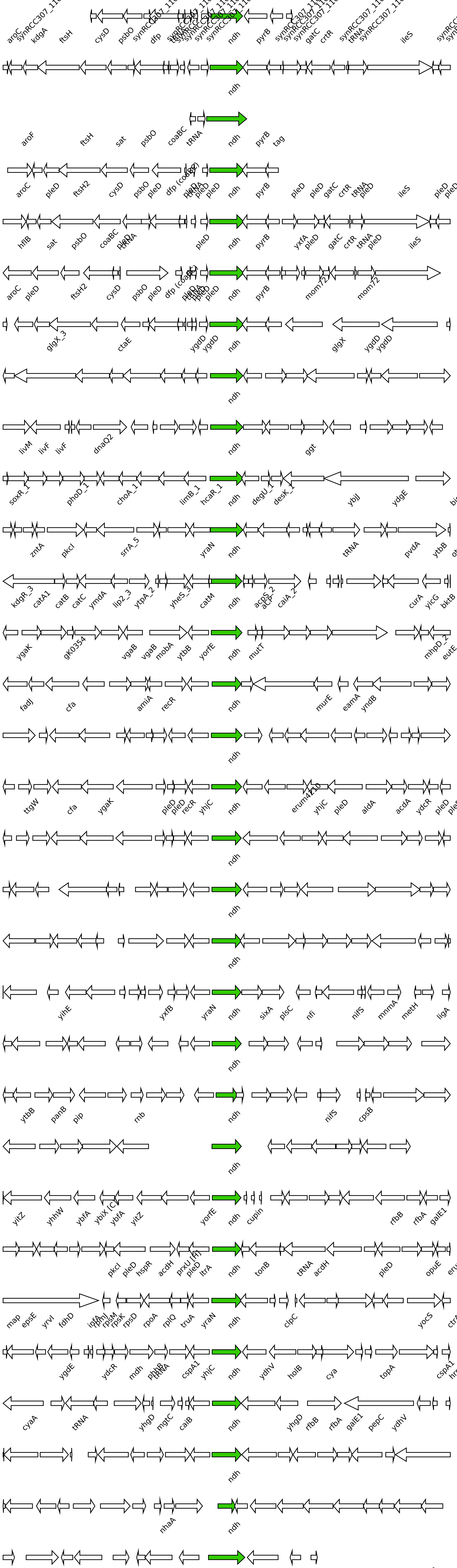

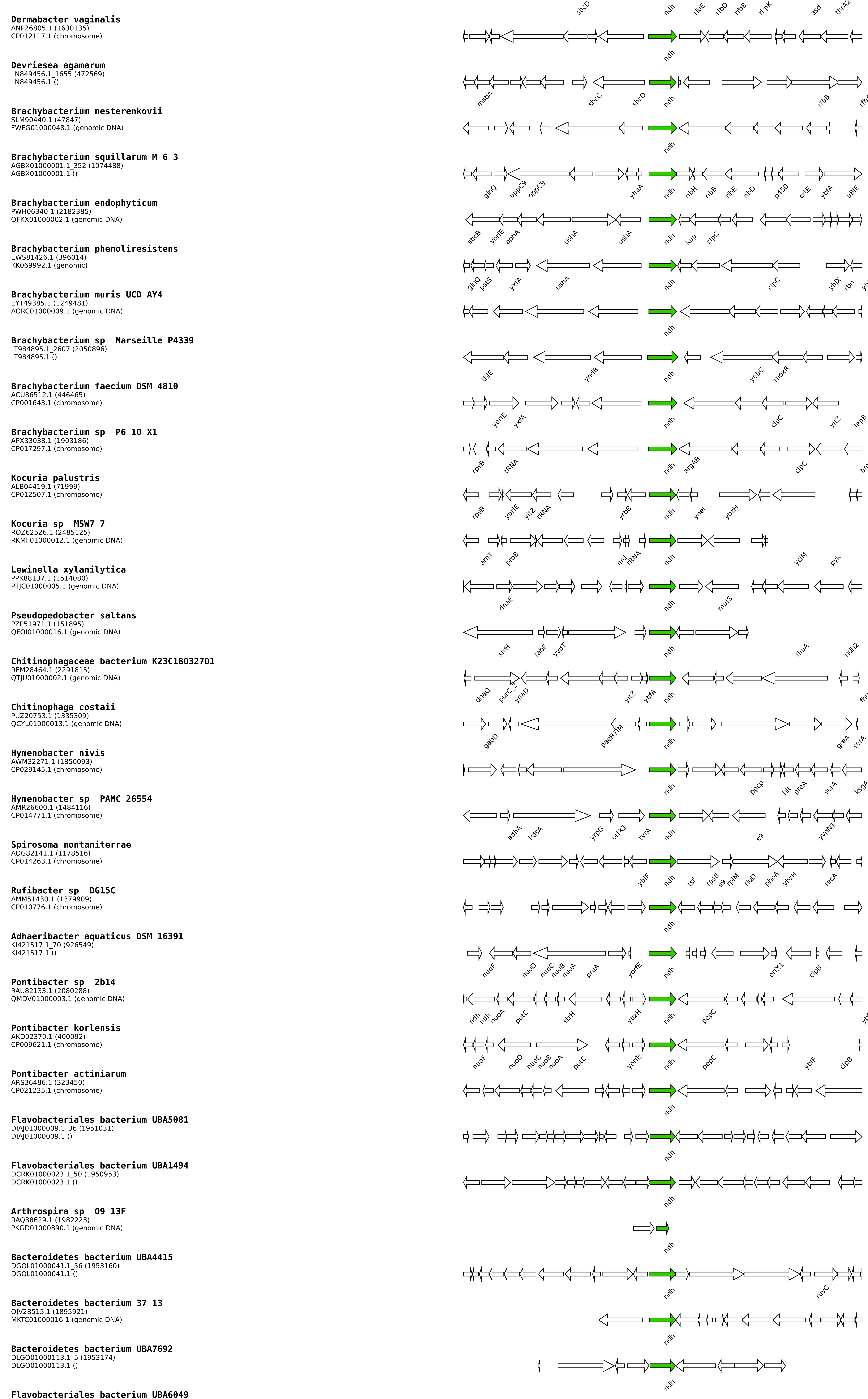

sbcD ndh ribE rfbD rfbB rkpK asd thrA2
Dermabacter vaginalis
ANP26805.1 (1630135)
CP012117.1 (chromosome)
ndh
Devriesea agamarum
LN849456.1_1655 (472569)
LN849456.1 ()
msbA sbcC sbcD ndh rfbB
Brachybacterium nesterenkovii
SLM90440.1 (47847)
FWFG01000048.1 (genomic DNA)
ndh
Brachybacterium squillarum M 6 3
AGBX01000001.1_352 (1074488)
AGBX01000001.1 ()
glnQ oppC9 oppC9 yhaA ndh ribH ribB ribE ribD p450 crtE ybfA uBIE
Brachybacterium endophyticum
PWH06340.1 (2182385)
QFKX01000002.1 (genomic DNA)
sbcB yorfE aphA ushA ushA ndh kup clpC
Brachybacterium phenoliresistens
EWS81426.1 (396014)
KK069992.1 (genomic)
glnQ pstS yxfA ushA ndh clpC yhjX rbn
Brachybacterium muris UCD AY4
EYT49385.1 (1249481)
AORC01000009.1 (genomic DNA)
ndh
Brachybacterium sp Marseille P4339
LT984895.1_2607 (2050896)
LT984895.1 ()
thiE yndB ndh yebC moxR
Brachybacterium faecium DSM 4810
ACU86512.1 (446465)
CP001643.1 (chromosome)
yorfE yxfA ndh clpC yitZ lepB
Brachybacterium sp P6 10 X1
APX33038.1 (1903186)
CP017297.1 (chromosome)
rpsB tRNA ndh argAB clpC
Kocuria palustris
ALB04419.1 (71999)
CP012507.1 (chromosome)
rpsB yorfE yitZ tRNA yrbB ndh yneI ybzH
Kocuria sp M5W7 7
ROZ62526.1 (2485125)
RKMF01000012.1 (genomic DNA)
arnT proB nrd tRNA ndh yciM pyk
Lewinella xylanilytica
PPK88137.1 (1514080)
PTJC01000005.1 (genomic DNA)
dnaE ndh mutS
Pseudopedobacter saltans
PZP51971.1 (151895)
QFOI01000016.1 (genomic DNA)
strH fabF yvdT ndh fhuA ndh2
Chitinophagaceae bacterium K23C18032701
RFM28464.1 (2291815)
QTJU01000002.1 (genomic DNA)
dnaQ purC_2 ynaD yitZ ybfA ndh
Chitinophaga costaii
PUZ20753.1 (1335309)
QCYL01000013.1 (genomic DNA)
gabD paeR7IM ndh greA serA
Hymenobacter nivis
AWM32271.1 (1850093)
CP029145.1 (chromosome)
ndh pgcp hit greA serA ksgA
Hymenobacter sp PAMC 26554
AMR26600.1 (1484116)
CP014771.1 (chromosome)
adhA kdsA yrpG orfX1 tyrA ndh s9 yvgN1
Spirosoma montaniterrae
AQG82141.1 (1178516)
CP014263.1 (chromosome)
ybfF ndh tsf rpsB s9 rplM rluD phoA ybzH recA
Rufibacter sp DG15C
AMM51430.1 (1379909)
CP010776.1 (chromosome)
ndh
Adhaeribacter aquaticus DSM 16391
KI421517.1_70 (926549)
KI421517.1 ()
nuoF nuoD nuoC nuoB nuoA pruA yorfE ndh orfX1 clpB
Pontibacter sp 2b14
RAU82133.1 (2080288)
QMDV01000003.1 (genomic DNA)
ndh ndh nuoA putC strH ybzH ndh pepC
Pontibacter korlensis
AKD02370.1 (400092)
CP009621.1 (chromosome)
nuoF nuoD nuoC nuoB nuoA putC yorfE ndh pepC ybfF clpB
Pontibacter actiniarum
ARS36486.1 (323450)
CP021235.1 (chromosome)
ndh
Flavobacteriales bacterium UBA5081
DIAJ01000009.1_36 (1951031)
DIAJ01000009.1 ()
ndh
Flavobacteriales bacterium UBA1494
DCRK01000023.1_50 (1950953)
DCRK01000023.1 ()
ndh
Arthrospira sp O9 13F
RAQ38629.1 (1982223)
PKGD01000890.1 (genomic DNA)
ndh
Bacteroidetes bacterium UBA4415
DGQL01000041.1_56 (1953160)
DGQL01000041.1 ()
ndh ruvC
Bacteroidetes bacterium 37 13
OJV28515.1 (1895921)
MKTC01000016.1 (genomic DNA)
ndh
Bacteroidetes bacterium UBA7692
DLGO01000113.1_5 (1953174)
DLGO01000113.1 ()
ndh
Flavobacteriales bacterium UBA6049

DIYR01000024.1_14 (1951045)
DIYR01000024.1 ()

Owenweeksia hongkongensis DSM 17368
AEV31771.1 (926562)
CP003156.1 (chromosome)

Flavobacteriales bacterium UBA2113
DCYJ01000013.1_116 (1950973)
DCYJ01000013.1 ()

Ornithobacterium rhinotracheale ORT UMN 88
AIP99881.1 (1401325)
CP006828.1 (chromosome)

Flavobacteriales bacterium UBA3376
DEQC01000083.1_4 (1950991)
DEQC01000083.1 ()

Moheibacter sediminis
SMC43851.1 (1434700)
FWXS01000002.1 (genomic DNA)

Capnocytophaga gingivalis ATCC 33624
EEK13807.1 (553178)
ACLQ01000024.1 (genomic)

Winogradskyella sp J14 2
APY09282.1 (1936080)
CP019388.1 (chromosome)

Hanstruepera neustonica
PNQ72192.1 (1445657)
POWF01000010.1 (genomic)

Bizionia echini
SFN74725.1 (649333)
FOVN01000003.1 (genomic DNA)

Flavobacteriaceae bacterium UBA5535
DINR01000135.1_22 (1951858)
DINR01000135.1 ()

Flavobacteriaceae bacterium T5 Br10_B2g0
LKMZ01000034.1_4 (1729469)
LKMZ01000034.1 ()

Croceibacter atlanticus HTCC2559
EAP86018.1 (216432)
CP002046.1 (chromosome)

Salinimicrobium catena
SDL68883.1 (390640)
FNGG01000008.1 (genomic DNA)

Gramella forsetii KT0803
CAL65987.1 (411154)
CU207366.1 (genomic DNA)

Gillisia sp Hel1_33_143
SDS46862.1 (1336796)
LT629737.1 (genomic DNA)

Flavobacteriaceae bacterium UBA3168
DEYC01000149.1_21 (1951823)
DEYC01000149.1 ()

Mesonia algae
PZW37797.1 (213248)
QKYV01000010.1 (genomic DNA)

Aquimarina sp AD1
AXT54239.1 (1714848)
CP031966.1 (chromosome)

Aquimarina agarilytica ZC1
JH621265.1_15 (620914)
JH621265.1 ()

Maribacter vaceletii
RKR12885.1 (1206816)
RBIQ01000008.1 (genomic DNA)

Psychroflexus torquis ATCC 700755
AFU67184.1 (313595)
CP003879.1 (chromosome)

Flavobacteriaceae bacterium 14752
RRO17111.1 (2491711)
RRZV01000066.1 (genomic DNA)

Pustulibacterium marinum
SFU55469.1 (1224947)
FPBK01000007.1 (genomic DNA)

Cellulophaga algicola DSM 14237
ADV49249.1 (688270)
CP002453.1 (chromosome)

Zobellia galactanivorans
CAZ96419.1 (63186)
FP476056.1 (genomic DNA)

Maribacter sp HTCC2170
EAR02350.1 (313603)
CP002157.1 (chromosome)

Bacteroidetes bacterium HGW Bacteroidetes 2
PKP26341.1 (2013688)
PHDJ01000005.1 (genomic DNA)

Flavobacteriaceae bacterium UBA3594
DFML01000017.1_322 (1951833)
DFML01000017.1 ()

Aequorivita sublithincola DSM 14238
AFL81638.1 (746697)
CP003280.1 (chromosome)

Flavobacteriaceae bacterium UBA3200
DEWW01000118.1_41 (1951826)
DEWW01000118.1 ()

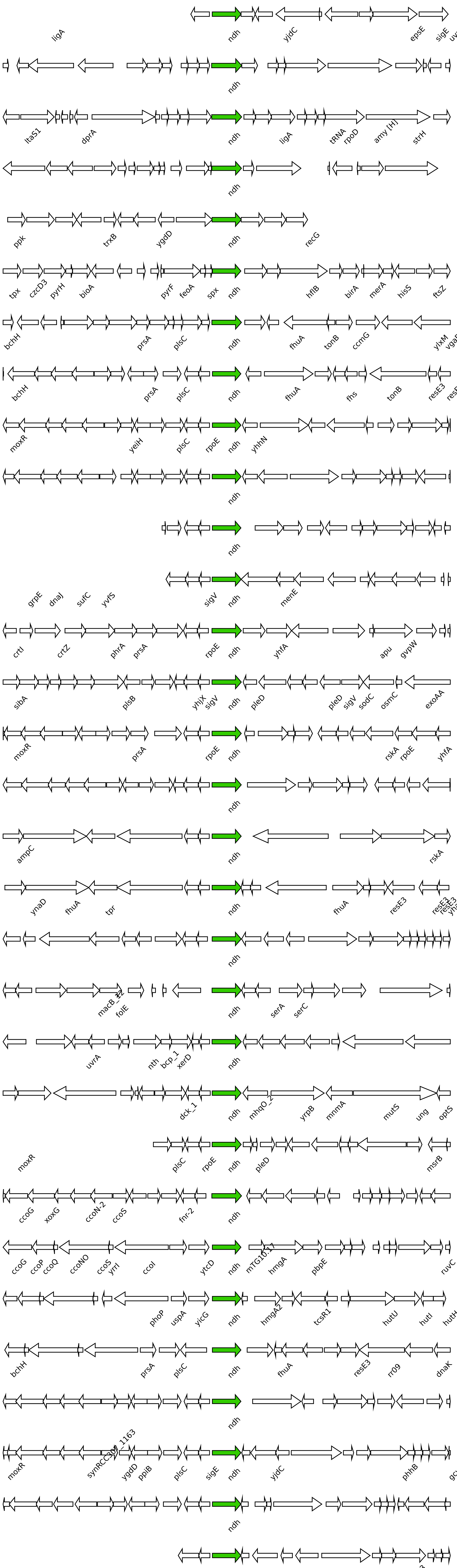

Marinirhabdus gelatinilytica
RDK87130.1 (1703343)
QRAO01000002.1 (genomic DNA)

Flavobacteriaceae bacterium UBA4504
DGNA01000008.1_22 (1951851)
DGNA01000008.1 ()

Aureitalea sp RR4 38
AVI49666.1 (2094025)
CP027062.1 (chromosome)

Ulvibacter antarcticus
RMA64728.1 (442714)
REFC01000012.1 (genomic DNA)

Leeuwenhoekiella blandensis MED217
EAQ48939.1 (398720)
CH672395.1 (genomic)

Robiginitalea biformata HTCC2501
EAR16055.1 (313596)
CP001712.1 (chromosome)

Lutibacter maritimus
SFS74043.1 (593133)
FOZP01000008.1 (genomic DNA)

Tenacibaculum jejuense
SNR15190.1 (584609)
LT899436.1 (genomic DNA)

Tenacibaculum maritimum NCIMB 2154
BAUG01000026.1_28 (1349785)
BAUG01000026.1 ()

Polaribacter sp ALD11
AUC85096.1 (2058137)
CP025119.1 (chromosome)

Tenacibaculum todarodis
APG63971.1 (1850252)
CP018155.1 (chromosome)

Tenacibaculum sp DSM 106434
AZJ36268.1 (2358479)
CP032548.1 (chromosome)

Lutibacter oricola
SDX16829.1 (762486)
FNNJ01000003.1 (genomic DNA)

Myroides odoratus
LROZ01000007.1_24 (256)
LROZ01000007.1 ()

Myroides guanonis
SFI83660.1 (1150112)
FORU01000001.1 (genomic DNA)

Flavobacteriaceae bacterium UR159
PJR03972.1 (2008672)
NIPO01000001.1 (genomic DNA)

Flavobacterium sp YIM 102701 2
RRJ89193.1 (2488728)
RQVQ01000032.1 (genomic DNA)

Flavobacterium marinum
SEH66941.1 (1159016)
FNXE01000007.1 (genomic DNA)

Chryseobacterium sp SCN 40 13
ODS82478.1 (1660093)
MEDR01000110.1 (genomic DNA)

Flavobacterium psychrophilum JIP02 86
CAL43997.1 (402612)
AM398681.2 (genomic DNA)

Flavobacterium columnare
AMA49309.1 (996)
CP013992.1 (chromosome)

Flavobacteria bacterium GWF1_32_7
OGS63087.1 (1798014)
MGWI01000006.1 (genomic DNA)

Flavobacterium gelidilacus DSM 15343
AUGN01000004.1_11 (1121891)
AUGN01000004.1 ()

Flavobacterium indicum GPTSA100 9 DSM 17447
CCG52752.1 (1094466)
HE774682.1 (genomic DNA)

Flavobacterium terrigena
SEJ33758.1 (402734)
FNYA01000010.1 (genomic DNA)

Sediminicola luteus
PCE65091.1 (319238)
KZ300476.1 (genomic)

Muricauda ruestringensis DSM 13258
AEM71421.1 (886377)
CP002999.1 (chromosome)

Flagellimonas sp HME9304
AWX45635.1 (1383885)
CP030104.1 (chromosome)

Muricauda lutaonensis
AKA34031.1 (516051)
CP011071.1 (chromosome)

Croceivirga radicis
AFOE01000050.1_142 (1929488)
AFOE01000050.1 ()

Lacinutrix sp 5H 3 7 4

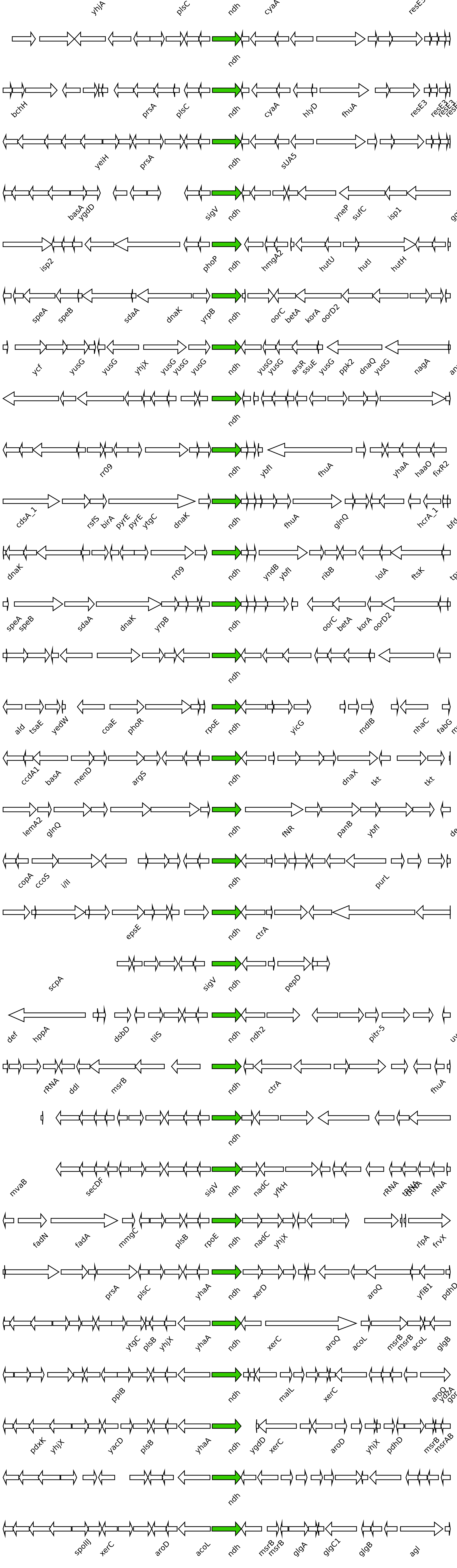

AEH02527.1 (983544)
CP002825.1 (chromosome)

Jejuia pallidilutea
GAL67291.1 (504487)
BBNR01000009.1 (genomic DNA)

Algibacter alginicilyticus
ALJ04226.1 (1736674)
CP012898.1 (chromosome)

Flavobacteriaceae bacterium UBA5535
DINR01000040.1_6 (1951858)
DINR01000040.1 ()

Formosa agariphila KMM 3901
CDF79436.1 (1347342)
HG315671.1 (genomic DNA)

Arenibacter sp
HCO84435.1 (1872633)
DQDD01000335.1 (genomic DNA)

Arenibacter sp AK53
MTAZ01000019.1_135 (1406873)
MTAZ01000019.1 ()

Cellulophaga algicola DSM 14237
ADV50140.1 (688270)
CP002453.1 (chromosome)

Maribacter vaceletii
RKR06466.1 (1206816)
RBIQ01000014.1 (genomic DNA)

Zobellia galactanivorans
CAZ94602.1 (63186)
FP476056.1 (genomic DNA)

Kordia sp SMS9
AXG70494.1 (2282170)
CP031153.1 (chromosome)

Imtechella halotolerans K1
EID74681.1 (946077)
AJJU01000009.1 (genomic)

Sinomicrobium oceani
SFW72181.1 (1150368)
FPJE01000027.1 (genomic DNA)

Zhouia amylolytica
SFT09557.1 (376730)
FPAG01000008.1 (genomic DNA)

Joostella marina DSM 19592
EIJ39469.1 (926559)
JH651379.1 (genomic)

Aureicoccus marinus
PQJ15459.1 (754435)
MQVX01000001.1 (genomic DNA)

Flavobacteriales bacterium CG_4_9_14_3_um_filter_40_17
PJB12199.1 (1974005)
PFUR01000060.1 (genomic DNA)

Wenyingzhuangia fucanilytica
ANW97251.1 (1790137)
CP014224.1 (chromosome)

Nonlabens sp MIC269
ALM20167.1 (1476901)
CP011373.1 (chromosome)

Nonlabens spongiae
ARN77295.1 (331648)
CP019344.1 (chromosome)

Nonlabens sp MB 3u 79
AUC79520.1 (2058134)
CP025116.1 (chromosome)

Nonlabens dokdonensis
OUS21409.1 (328515)
MAAX01000018.1 (genomic DNA)

Nonlabens dokdonensis DSW 6
AGC78697.1 (592029)
CP001397.1 (chromosome)

Balneolaceae bacterium UBA3974
DGCQ01000013.1_243 (1951504)
DGCQ01000013.1 ()

Bacteroidetes endosymbiont of Geopemphigus sp
PEIH01000005.1_43 (2047937)
PEIH01000005.1 ()

Persicobacter sp CCB QB2
LBGV01000001.1_598 (1561025)
LBGV01000001.1 ()

Bacteroidetes bacterium UBA6063
DIYD01000048.1_14 (1953164)
DIYD01000048.1 ()

Crocinitomix algicola
LYPF02000017.1_17 (1740263)
LYPF02000017.1 ()

Crocinitomix catalasitica ATCC 23190
JHXV01000006.1_70 (1408433)
JHXV01000006.1 ()

Reichenbachiella versicolor
QFZQ01000002.1_224 (1821036)
QFZQ01000002.1 ()

Crocinitomix sp SM1701
PZE17062.1 (2219161)
QKSB01000005.1 (genomic DNA)

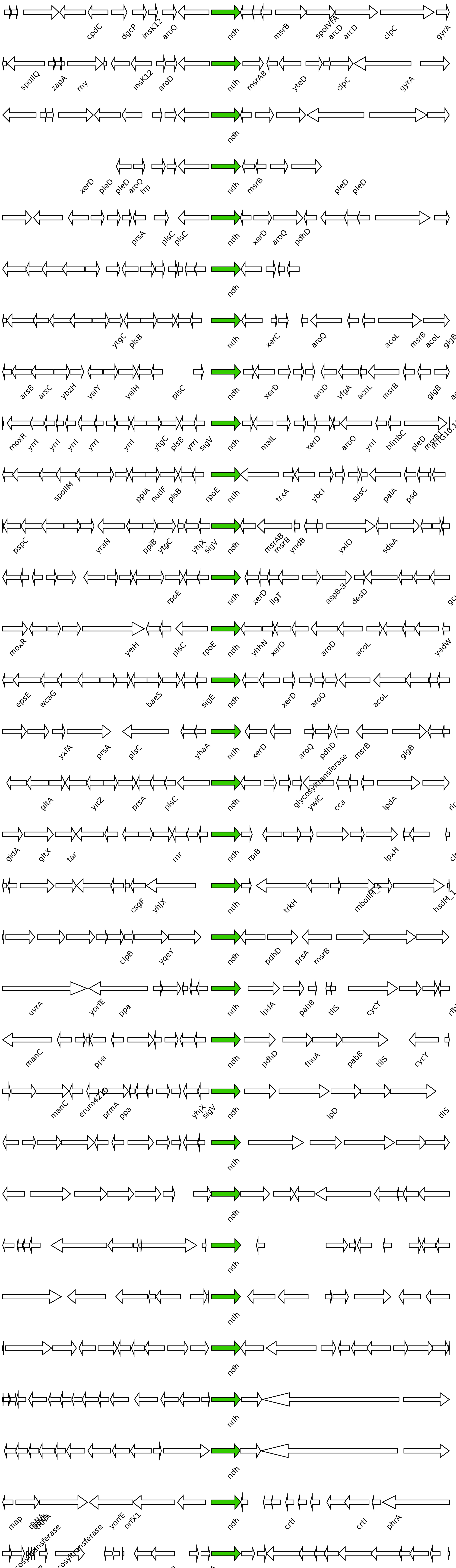

Fluviicola sp XM 24bin1
PWL24525.1 (2133950)
QCWI01000012.1 (genomic DNA)

Saprospiraceae bacterium UBA7328
DKPS01000022.1_1 (1952882)
DKPS01000022.1 ()

Solitalea canadensis DSM 3403
AFD07340.1 (929556)
CP003349.1 (chromosome)

Sphingobacteriaceae bacterium GW460 11 11 14 LB5
ARS40548.1 (1986952)
CP021237.1 (chromosome)

Pedobacter heparinus DSM 2366
ACU03633.1 (485917)
CP001681.1 (chromosome)

Sphingobacteriaceae bacterium UBA2059
DDAL01000024.1_150 (1953078)
DDAL01000024.1 ()

Arcticibacter pallidicorallinus
PRY55448.1 (1259464)
PVTH01000001.1 (genomic DNA)

Pedobacter sp FJ4 8
PWG79765.1 (2173175)
QEAS01000012.1 (genomic DNA)

Pedobacter luteus
SKB39169.1 (572036)
FUYR01000001.1 (genomic DNA)

Mucilaginibacter yixingensis
PTR01336.1 (1295612)
QAOQ01000001.1 (genomic DNA)

Mucilaginibacter gotjawali
BAU55272.1 (1550579)
AP017313.1 (genomic DNA)

Mucilaginibacter sp PPCGB 2223
OCX51542.1 (1886027)
MDJS01000003.1 (genomic DNA)

bacterium A37T11
SEM16210.1 (1855384)
FOBR01000002.1 (genomic DNA)

Parapedobacter luteus
SKB41612.1 (623280)
FUYS01000002.1 (genomic DNA)

Sphingobacterium sp ML3W
AIM37810.1 (1538644)
CP009278.1 (chromosome)

Sphingobacterium psychroaquaticum
SMG06840.1 (561061)
FXAU01000001.1 (genomic DNA)

Sphingobacterium nematocida
SKB54924.1 (1513896)
FUZF01000003.1 (genomic DNA)

Sphingobacterium sp T2
JXAC01000172.1_3 (1590596)
JXAC01000172.1 ()

Sphingobacterium sp 2c 3
PVH24653.1 (2171749)
QDKG01000005.1 (genomic DNA)

Sphingobacterium mizutaii
SDL28633.1 (1010)
FNGK01000002.1 (genomic DNA)

Pseudopedobacter saltans DSM 12145
ADY52599.1 (762903)
CP002545.1 (chromosome)

Pedobacter arcticus A12
JH947127.1_591 (1206103)
JH947127.1 ()

Mucilaginibacter sp PPCGB 2223
OCX52909.1 (1886027)
MDJS01000001.1 (genomic DNA)

Mucilaginibacter gotjawali
BAU54731.1 (1550579)
AP017313.1 (genomic DNA)

Mucilaginibacter yixingensis
PTQ96866.1 (1295612)
QAOQ01000004.1 (genomic DNA)

Chitinophaga costaii
PUZ20709.1 (1335309)
QCYL01000013.1 (genomic DNA)

Bacteroidetes bacterium UBA7662
DLHS01000007.1_130 (1953171)
DLHS01000007.1 ()

Thermoflexibacter ruber
SFF57054.1 (1003)
FONY01000061.1 (genomic DNA)

Flammeovirgaceae bacterium UBA1842
DDIU01000022.1_27 (1951769)
DDIU01000022.1 ()

Cesiribacter andamanensis AMV16
EMR04695.1 (1279009)
AODQ01000002.1 (genomic DNA)

Nafulsella turpanensis ZLM 10

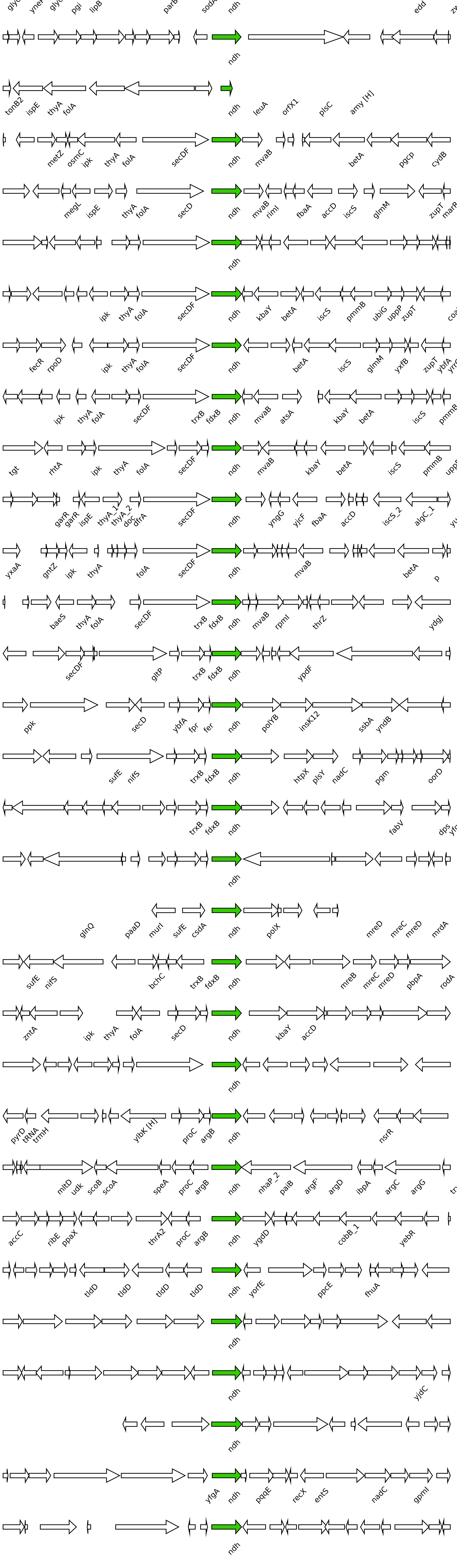

ANNU01000006.1_168 (1124780)
ANNU01000006.1 ()

Marivirga sp ANT B6
LXQK01000170.1_17 (1844760)
LXQK01000170.1 ()

Marivirga tractuosa DSM 4126
ADR22526.1 (643867)
CP002349.1 (chromosome)

Cytophagales bacterium CG12_big_fil_rev_8_21_14_0_65_40_12
PIQ50348.1 (1973954)
PCUU01000002.1 (genomic DNA)

Fabibacter pacificus
RKQ50786.1 (1267423)
RBHZ01000001.1 (genomic DNA)

Roseivirga sp UBA6052
DIYO01000009.1_667 (1947389)
DIYO01000009.1 ()

Fabibacter misakiensis
OEK07037.1 (1563681)
MDGQ01000003.1 (genomic DNA)

Roseivirga sp UBA4591
DGJR01000257.1_61 (1947388)
DGJR01000257.1 ()

Roseivirga sp XM 24bin3
PWL30786.1 (2133949)
QCWJ01000002.1 (genomic DNA)

Reichenbachiella faecimaris
SMD35407.1 (692418)
FWYF01000002.1 (genomic DNA)

Reichenbachiella agariperforans
SHK55559.1 (156994)
FRAA01000006.1 (genomic DNA)

Ekhidna lutea
SNS53386.1 (447679)
FZPD01000001.1 (genomic DNA)

Bernardetia litoralis DSM 6794
AFM03225.1 (880071)
CP003345.1 (chromosome)

Adhaeribacter aquaticus DSM 16391
KI421517.1_3721 (926549)
KI421517.1 ()

Hymenobacter sp PAMC 26554
AMR27825.1 (1484116)
CP014771.1 (chromosome)

Hymenobacter nivis
AWM34514.1 (1850093)
CP029145.1 (chromosome)

Raineya orbicola
PKQ70917.1 (2016530)
NKXO01000001.1 (genomic DNA)

bacterium 336 3
KOY87507.1 (1664068)
LJIE01000001.1 (genomic DNA)

Eisenibacter elegans DSM 3317
AUMD01000019.1_35 (1121902)
AUMD01000019.1 ()

Microscillaceae bacterium
HAI75188.1 (2053581)
DMHB01000099.1 (genomic DNA)

Hugenholtzia roseola DSM 9546
AUGI01000038.1_48 (1121903)
AUGI01000038.1 ()

Flammeovirgaceae bacterium UBA2352
DDTY01000015.1_2 (1951776)
DDTY01000015.1 ()

Flammeovirgaceae bacterium UBA2338
DDUM01000052.1_2 (1951775)
DDUM01000052.1 ()

Flammeovirgaceae bacterium UBA2384
DDSS01000134.1_3 (1951779)
DDSS01000134.1 ()

Cytophagaceae bacterium Ys
GCC53310.1 (2482724)
BHXQ01000007.1 (genomic DNA)

Bacteroidetes bacterium 2013_40CM_41_45
NGFH01000001.1_70 (1978119)
NGFH01000001.1 ()

Bacteroidetes bacterium OLB9
KXK37309.1 (1617420)
LNBW01000066.1 (genomic DNA)

Lishizhenia tianjinensis
SFT44112.1 (477690)
FPAS01000001.1 (genomic DNA)

Robiginitalea biformata HTCC2501
EAR14319.1 (313596)
CP001712.1 (chromosome)

Robiginitalea myxolifaciens
SFR32233.1 (400055)
FOYQ01000001.1 (genomic DNA)

Aquimarina agarilytica ZC1
JH621254.1_63 (620914)
JH621254.1 ()

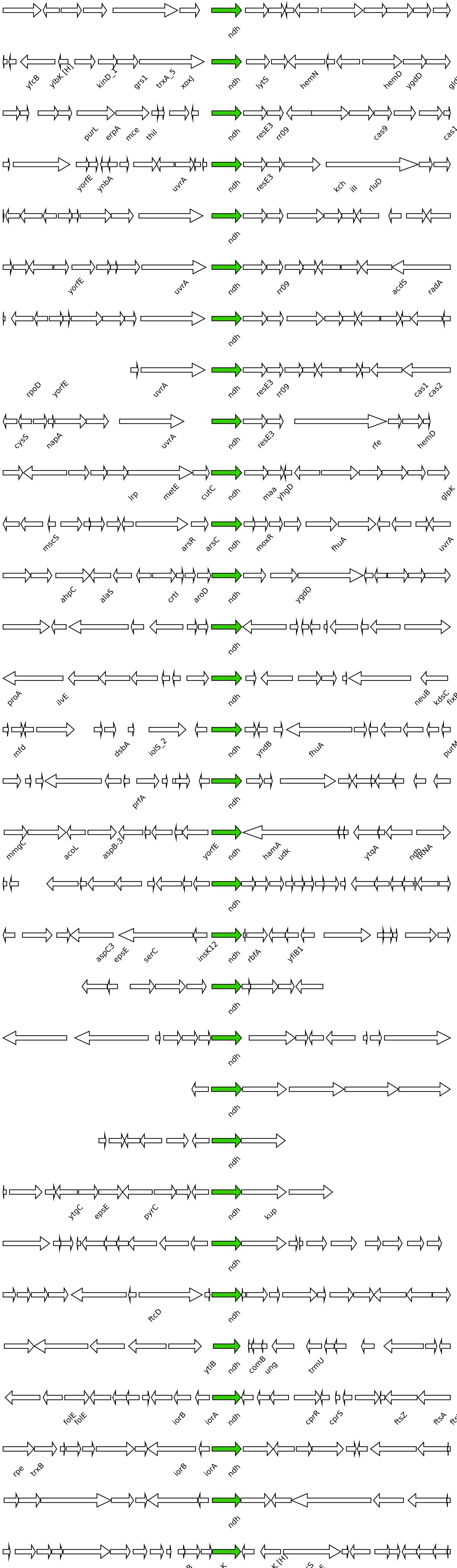

Arenibacter sp
HCO82837.1 (1872633)
DQDD01000134.1 (genomic DNA)

Bacteroidetes bacterium HGW Bacteroidetes 3
PKP13459.1 (2013693)
PHDE01000032.1 (genomic DNA)

Cellulophaga lytica
AIM61639.1 (979)
CP009239.1 (chromosome)

Lutibacter sp LPB0138
AOW19798.1 (1850246)
CP017478.1 (chromosome)

Flavobacteriaceae bacterium UBA1473
DCSF01000023.1_69 (1951799)
DCSF01000023.1 ()

Alistipes sp ZOR0009
JTLD01000012.1_24 (1339253)
JTLD01000012.1 ()

Flavobacteriales bacterium UBA2104
DCYS01000032.1_22 (1950971)
DCYS01000032.1 ()

Flavobacteriales bacterium UBA2423
DDRF01000056.1_1 (1950977)
DDRF01000056.1 ()

Hymenobacter actinosclerus
SET81851.1 (82805)
FOHS01000003.1 (genomic DNA)

Hymenobacter sp APR13
AII51371.1 (1356852)
CP006587.1 (chromosome)

Hymenobacter sp sh 6
AYA37379.1 (2319843)
CP032317.1 (chromosome)

Hymenobacter daecheongensis DSM 21074
SHJ15917.1 (1121955)
FQYN01000004.1 (genomic DNA)

Hymenobacter glacialis
OGX87790.1 (1908236)
MDZC01000028.1 (genomic DNA)

Hymenobacter nivis
AWM34667.1 (1850093)
CP029145.1 (chromosome)

Hymenobacter sp PAMC 26554
AMR27018.1 (1484116)
CP014771.1 (chromosome)

Bacteroidales bacterium UBA6229
DJWN01000107.1_26 (1950671)
DJWN01000107.1 ()

Bacteroidales bacterium UBA5536
DINQ01000015.1_132 (1950645)
DINQ01000015.1 ()

Bacteroidetes bacterium UBA2331
DDUT01000123.1_2 (1953146)
DDUT01000123.1 ()

Schleiferia sp LA
GCD78740.1 (2173829)
BHZE01000042.1 (genomic DNA)

Bacteroidetes bacterium UKL13 3
AMS27503.1 (1690483)
CP012155.1 (chromosome)

Bacteroidetes bacterium UBA6183
DITN01000103.1_7 (1953166)
DITN01000103.1 ()

Chitinophaga caeni
ATL47492.1 (2029983)
CP023777.1 (chromosome)

Chitinophaga costaii
PUZ30459.1 (1335309)
QCYL01000001.1 (genomic DNA)

Bacteroidetes bacterium UBA7236
DKTG01000453.1_3 (1953170)
DKTG01000453.1 ()

Thermonema rossianum DSM 10300
AUGC01000014.1_1 (1313301)
AUGC01000014.1 ()

Brumimicrobium aurantiacum
RFC54829.1 (1737063)
QURB01000003.1 (genomic)

Flavobacteriales bacterium UBA852
DBGO01000005.1_4 (1951088)
DBGO01000005.1 ()

Flavobacteriales bacterium UBA5051
DIBN01000006.1_209 (1951030)
DIBN01000006.1 ()

Cyclobacterium marinum DSM 745
AEL28254.1 (880070)
CP002955.1 (chromosome)

Flammeovirgaceae bacterium UBA1842
DDIU01000015.1_159 (1951769)
DDIU01000015.1 ()

Lutibacter profundi
AMC10953.1 (1622118)

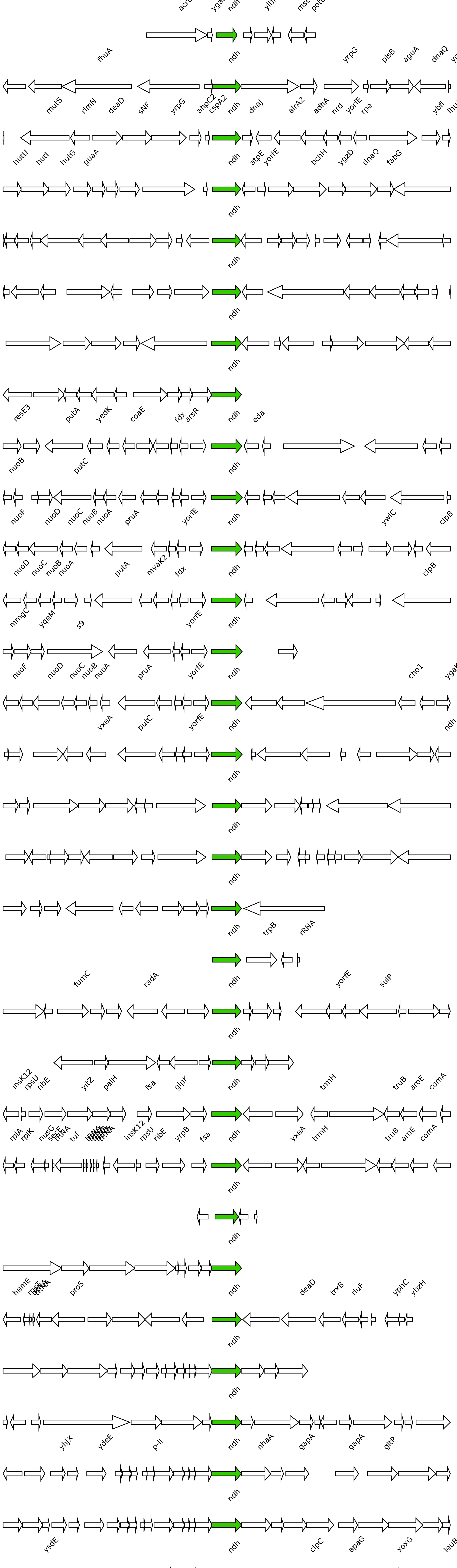

[illegible]
CP013355.1 (chromosome)

Arenibacter sp
HCO82575.1 (1872633)
DQDD01000097.1 (genomic DNA)

Flavobacteriaceae bacterium UBA7302
DKQS01000003.1_161 (1951874)
DKQS01000003.1 ()

Lutibacter sp LPB0138
AOW21258.1 (1850246)
CP017478.1 (chromosome)

Bizionia echini
SFN40109.1 (649333)
FOVN01000001.1 (genomic DNA)

Leeuwenhoekiella blandensis MED217
EAQ49985.1 (398720)
CH672395.1 (genomic)

Joostella marina DSM 19592
EIJ37501.1 (926559)
JH651380.1 (genomic)

Sinomicrobium oceani
SFW33069.1 (1150368)
FPJE01000005.1 (genomic DNA)

Nonlabens spongiae
ARN78926.1 (331648)
CP019344.1 (chromosome)

Flavobacteriaceae bacterium UBA1473
DCSF01000011.1_55 (1951799)
DCSF01000011.1 ()

Flavobacteriales bacterium UBA2113
DCYJ01000021.1_85 (1950973)
DCYJ01000021.1 ()

Flavobacteriaceae bacterium UJ101
APD08186.1 (1150389)
CP016269.1 (chromosome)

Saprospirales bacterium
MAT54107.1 (2026790)
PAAY01000035.1 (genomic DNA)

Saprospiraceae bacterium
QIDR01000257.1_1 (2202734)
QIDR01000257.1 ()

Bacteroidetes bacterium HGW Bacteroidetes 4
PKP09523.1 (2013694)
PHDD01000014.1 (genomic DNA)

Flavobacteriales bacterium CG_4_9_14_3_um_filter_32_8
PJB14585.1 (1974004)
PFUQ01000161.1 (genomic DNA)

Flavobacteriales bacterium CG_4_10_14_0_2_um_filter_35_18
PJA04897.1 (1973999)
PFPU01000164.1 (genomic DNA)

Bacteroidetes bacterium HGW Bacteroidetes 3
PKP14308.1 (2013693)
PHDE01000004.1 (genomic DNA)

Flavobacteriales bacterium UBA852
DBGO01000012.1_61 (1951088)
DBGO01000012.1 ()

Bacteroidetes bacterium 24 39 8
OYZ51269.1 (1970493)
NCHJ01000040.1 (genomic DNA)

Flavobacterium sp BFFFF2
OYU84851.1 (2015558)
NKJD01000009.1 (genomic DNA)

Bacteroidetes bacterium B1 2017
OYU95510.1 (2015582)
NKJG01000003.1 (genomic DNA)

Hydrotalea sandarakina
PZX65791.1 (1004304)
QKZV01000001.1 (genomic DNA)

Chitinophagaceae bacterium UBA4407
DGQT01000067.1_13 (1951557)
DGQT01000067.1 ()

Chitinophagaceae bacterium UBA806
DBII01000056.1_34 (1951567)
DBII01000056.1 ()

Chitinophagaceae bacterium K23C18032701
RFM28097.1 (2291815)
QTJU01000003.1 (genomic DNA)

Parafilimonas terrae
SFP89972.1 (1465490)
FOXQ01000003.1 (genomic DNA)

Chitinophagaceae bacterium UBA1931
DDFJ01000001.1_58 (1951545)
DDFJ01000001.1 ()

Chitinophagaceae bacterium UBA4412
DGQO01000058.1_21 (1951559)
DGQO01000058.1 ()

Chitinophagaceae bacterium UBA1596
DCNM01000136.1_5 (1951543)
DCNM01000136.1 ()

Sphingobacteriales bacterium 46 32
OJW30070.1 (1895839)
MKTY01000027.1 (genomic DNA)

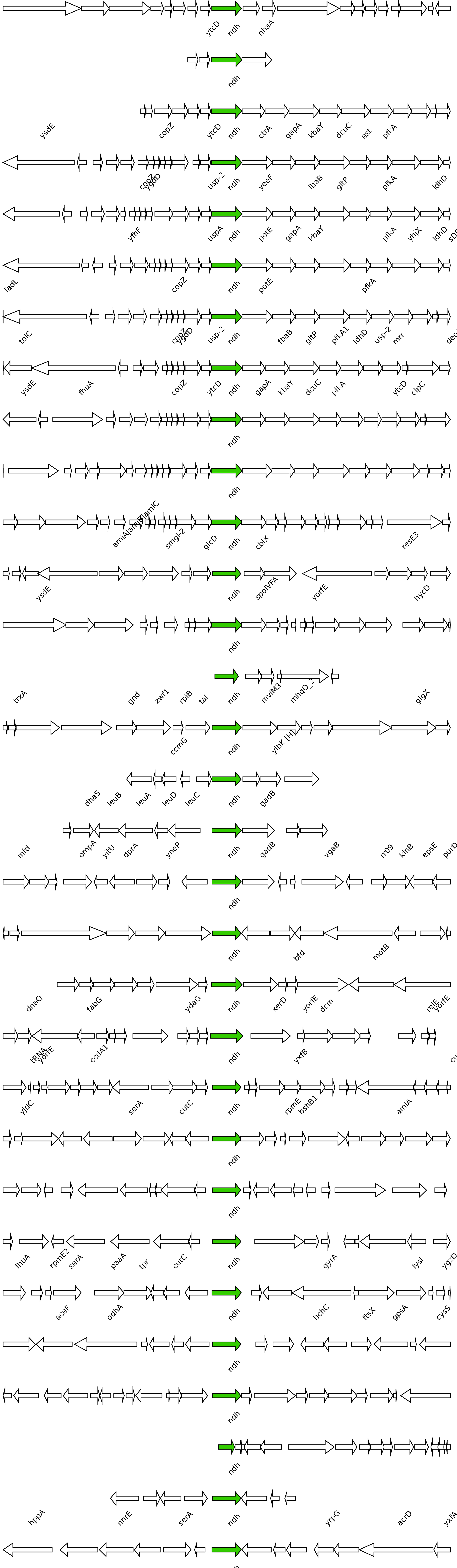

Chitinophagaceae bacterium UBA4857
DHEF01000011.1_22 (1951561)
DHEF01000011.1 ()

Chitinophagales bacterium 62 2
MWYU01000611.1_12 (1962172)
MWYU01000611.1 ()

Chitinophagaceae bacterium HBUM179779
RCWL01000002.1_198 (2479004)
RCWL01000002.1 ()

Niastella sp SCN 39 18
ODT52098.1 (1660119)
MEEN01000028.1 (genomic DNA)

Chitinophagaceae bacterium UBA3961
DGDD01000183.1_4 (1951555)
DGDD01000183.1 ()

Sphingobacteriales bacterium 12 47 4
OYW17698.1 (1970580)
NCCA01000444.1 (genomic DNA)

Algibacter alginicilyticus
ALJ06352.1 (1736674)
CP012898.1 (chromosome)

Taibaiella chishuiensis
PSK92190.1 (1434707)
PYGD01000004.1 (genomic DNA)

Bacteroidetes bacterium 47 18
OJU75120.1 (1895926)
MKSF01000021.1 (genomic DNA)

Bacteroidetes bacterium UBA6221
DISB01000013.1_49 (1953167)
DISB01000013.1 ()

Bacteroidetes bacterium UBA7674
DLHG01000013.1_212 (1953172)
DLHG01000013.1 ()

Bacteroidetes bacterium 46 16
OJW83188.1 (1895925)
MKUN01000019.1 (genomic DNA)

Bacteroidetes bacterium UBA1947
DDET01000016.1_52 (1953142)
DDET01000016.1 ()

Bacteroidetes bacterium 43 93
OJW95553.1 (1895924)
MKUO01000027.1 (genomic DNA)

Taibaiella soli
PZF71404.1 (1649169)
QKTW01000025.1 (genomic DNA)

Flavipsychrobacter stenotrophus
PQJ10826.1 (2077091)
PPSL01000003.1 (genomic DNA)

Weeksella virosa DSM 16922
ADX68758.1 (865938)
CP002455.1 (chromosome)

Vaginella massiliensis
LT593747.1_364 (1816680)
LT593747.1 ()

Flavobacteriaceae bacterium YIM 102668
RLZ09871.1 (2479857)
RDOJ01000008.1 (genomic DNA)

Algoriella xinjiangensis
SFM65302.1 (684065)
FOUZ01000001.1 (genomic DNA)

Arachidicoccus sp KIS59 12
AYD47005.1 (2341117)
CP032489.1 (chromosome)

Arachidicoccus sp BS20
ANI88314.1 (1850526)
CP015971.1 (chromosome)

Arachidicoccus rhizosphaerae
SDZ83325.1 (551991)
FNQY01000002.1 (genomic DNA)

Pedobacter sp FJ4 8
PWG79075.1 (2173175)
QEAS01000017.1 (genomic DNA)

Flavobacteriales bacterium UBA5871
DJFN01000188.1_4 (1951041)
DJFN01000188.1 ()

Glaciecola sp THG 3 7
CP034120.1_1173 (2489595)
CP034120.1 ()

Fluviicola taffensis DSM 16823
AEA46025.1 (755732)
CP002542.1 (chromosome)

Bacteroidetes bacterium UBA4416
DGQK01000002.1_16 (1953161)
DGQK01000002.1 ()

Sphingobacteriaceae bacterium
PBQ32862.1 (2021370)
NVQB01000001.1 (genomic DNA)

Porphyromonadaceae bacterium CG2_30_38_12
OIP83145.1 (1805325)
MNZK01000062.1 (genomic DNA)

Bacteroidales bacterium Bact_04
LUYV01000053.1_7 (1674818)

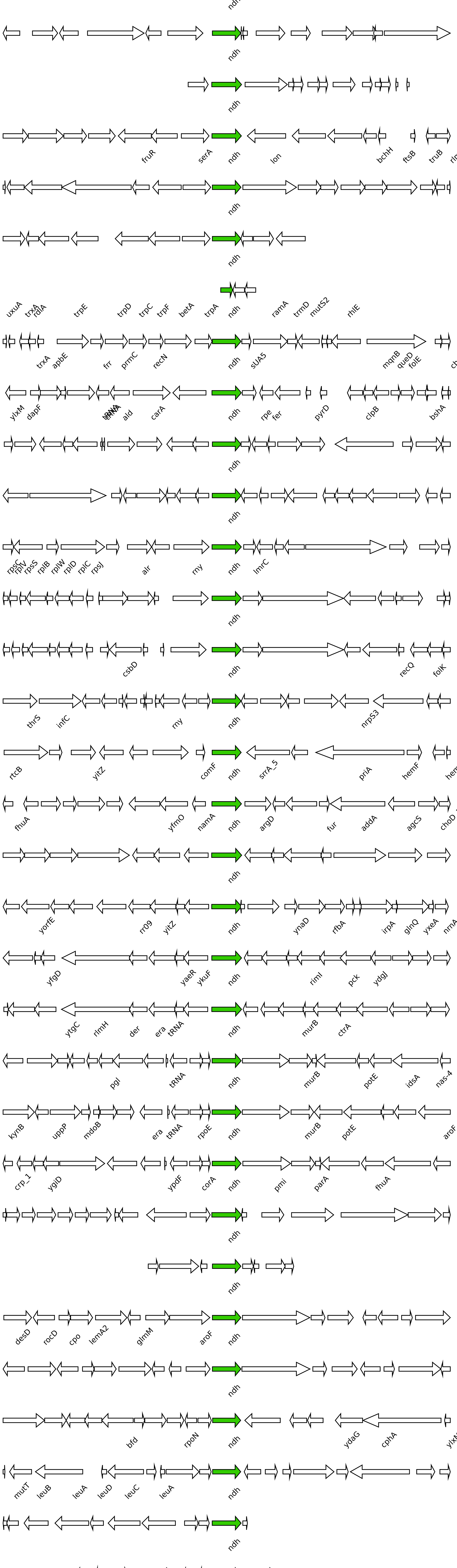

LUYV01000053.1 ()

Bacteroidales bacterium 36 12
OJV45164.1 (1895718)
MKSZ01000005.1 (genomic DNA)

Paludibacter propionicigenes WB4
ADQ78836.1 (694427)
CP002345.1 (chromosome)

Porphyromonadaceae bacterium H1
BDEJ01000042.1_18 (1658779)
BDEJ01000042.1 ()

Elizabethkingia meningoseptica
KUY23178.1 (238)
LNOH01000002.1 (genomic DNA)

Elizabethkingia meningoseptica
KUY15151.1 (238)
LNOH01000021.1 (genomic DNA)

Chryseobacterium sp H6466
AZI56281.1 (2487072)
CP034160.1 (chromosome)

Soonwooa buanensis
SKB71239.1 (619805)
FUYZ01000002.1 (genomic DNA)

Flavobacteriales bacterium UBA7623
DLJF01000005.1_2 (1951082)
DLJF01000005.1 ()

Flavobacteriales bacterium UBA1937
DDFD01000044.1_59 (1950958)
DDFD01000044.1 ()

Riemerella anatipestifer
AKP68839.1 (34085)
CP007503.1 (chromosome)

Chryseobacterium taklimakanense
AZI20939.1 (536441)
CP034171.1 (chromosome)

Chryseobacterium sp SCN 40 13
ODS89082.1 (1660093)
MEDR01000029.1 (genomic DNA)

Flavobacteriales bacterium UBA1354
DBSA01000024.1_5 (1950952)
DBSA01000024.1 ()

Chryseobacterium carnis
AZI34217.1 (1241979)
CP034159.1 (chromosome)

Bergeyella zoohelcum CCUG 30536
EKB61684.1 (883098)
JH932282.1 (genomic)

Flavobacteriales bacterium UBA1694
DCJS01000025.1_89 (1950956)
DCJS01000025.1 ()

Flavobacteriaceae bacterium 3519 10
ACU08284.1 (531844)
CP001673.1 (chromosome)

Flavobacteriaceae bacterium KYPW7
REFA01000035.1_7 (2479858)
REFA01000035.1 ()

Riemerella columbipharyngis
SDE03866.1 (1071918)
FNAS01000002.1 (genomic DNA)

Cruoricaptor ignavus
SHJ04556.1 (1118202)
FQYI01000008.1 (genomic DNA)

Chryseobacterium frigidisoli
SFI54401.1 (1125876)
FOQT01000005.1 (genomic DNA)

Bergeyella cardium
QYCZ01000001.1_1255 (1585976)
QYCZ01000001.1 ()

Flavobacteriales bacterium UBA1354
DBSA01000023.1_1 (1950952)
DBSA01000023.1 ()

Bacteroidales bacterium UBA5507
DIOT01000005.1_133 (1950642)
DIOT01000005.1 ()

Bacteroidetes bacterium GWC2_40_13
OFX57093.1 (1797325)
MENO01000164.1 (genomic DNA)

Alkaliflexus imshenetskii DSM 15055
KI912105.1_1138 (927658)
KI912105.1 ()

Marinilabiliaceae bacterium UBA5213
DHVH01000206.1_8 (1952210)
DHVH01000206.1 ()

Geofilum rhodophaeum
MWUJ01000001.1_352 (1965019)
MWUJ01000001.1 ()

Marinilabiliaceae bacterium
HBG86006.1 (2053574)
DNQT01000008.1 (genomic DNA)

Marinilabiliaceae bacterium UBA5279
DHST01000037.1_1 (1952211)
DHST01000037.1 ()

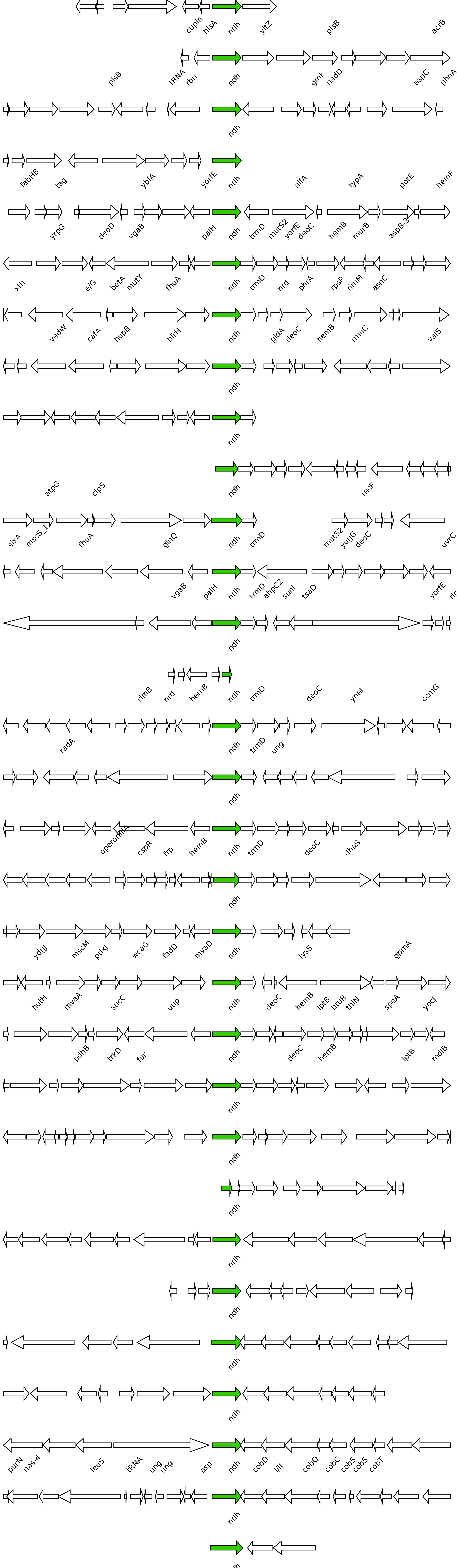

Marinilabilia sp UBA5340
DHQK01000202.1_6 (1946809)
DHQK01000202.1 ()

Marinilabilia salmonicolor
PRZ02241.1 (989)
PVTS01000001.1 (genomic DNA)

Bacteroidales bacterium Bact_23
LUZO01000434.1_5 (1674837)
LUZO01000434.1 ()

Bacteroidetes bacterium HGW Bacteroidetes 15
PKP38367.1 (2013683)
PHDO01000184.1 (genomic DNA)

Bacteroidetes bacterium HGW Bacteroidetes 11
PKP48646.1 (2013679)
PHDS01000028.1 (genomic DNA)

Bacteroidetes bacterium HGW Bacteroidetes 9
PKO95182.1 (2013699)
PHCY01000438.1 (genomic DNA)

Bacteroidetes bacterium CG2_30_33_31
OIP02497.1 (1805033)
MNXP01000011.1 (genomic DNA)

Bacteroidetes bacterium RIFCSPLOWO2_12_FULL_35_15
OFY85828.1 (1797364)
MEPB01000045.1 (genomic DNA)

Bacteroidetes bacterium HGW Bacteroidetes 3
PKP13335.1 (2013693)
PHDE01000037.1 (genomic DNA)

Bacteroidetes bacterium HGW Bacteroidetes 15
PKP37087.1 (2013683)
PHDO01000257.1 (genomic DNA)

Bacteroidetes bacterium HGW Bacteroidetes 1
PKP51431.1 (2013677)
PHDU01000059.1 (genomic DNA)

Bacteroidales bacterium UBA6192
DITE01000025.1_15 (1950669)
DITE01000025.1 ()

Marinilabiliaceae bacterium
HBG87163.1 (2053574)
DNQT01000067.1 (genomic DNA)

Bacteroidales bacterium UBA5349
DHQB01000017.1_65 (1950624)
DHQB01000017.1 ()

Bacteroidales bacterium UBA6013
DJAB01000256.1_17 (1950665)
DJAB01000256.1 ()

Bacteroidetes bacterium OLB10
KXK45579.1 (1617421)
LNBX01000039.1 (genomic DNA)

Bacteroidales bacterium UBA1064
DCDD01000203.1_12 (1950370)
DCDD01000203.1 ()

Cytophaga hutchinsonii ATCC 33406
ABG57542.1 (269798)
CP000383.1 (chromosome)

Bacteroidetes bacterium OLB11
KXK41898.1 (1617422)
LNFQ01000053.1 (genomic DNA)

Sediminibacterium salmoneum NBRC 103935
KI866530.1_70 (1400522)
KI866530.1 ()

Parabacteroides chartae
SKB66484.1 (1037355)
FUYQ01000016.1 (genomic DNA)

Saprospiraceae bacterium UBA7687
DLGT01000107.1_4 (1952883)
DLGT01000107.1 ()

Bacteroidales bacterium UBA2632
DENY01000226.1_3 (1950447)
DENY01000226.1 ()

Bacteroidales bacterium UBA3143
DEZB01000056.1_11 (1950476)
DEZB01000056.1 ()

Bacteroidales bacterium UBA2632
DENY01000226.1_4 (1950447)
DENY01000226.1 ()

Bacteroidales bacterium UBA5299
DHRZ01000005.1_12 (1950619)
DHRZ01000005.1 ()

Petrimonas mucosa
SCM59147.1 (1642646)
LT608328.1 (genomic DNA)

Proteiniphilum saccharofermentans
SCD19675.1 (1642647)
LT605205.1 (genomic DNA)

Proteiniphilum sp 51_7
KUL19018.1 (1641377)
LGHH01000095.1 (genomic DNA)

Proteiniphilum sp UBA5463
DIQL01000013.1_35 (1947281)
DIQL01000013.1 ()

Bacteroidetes bacterium GWC2_46_850
OFX55741.1 (1797327)

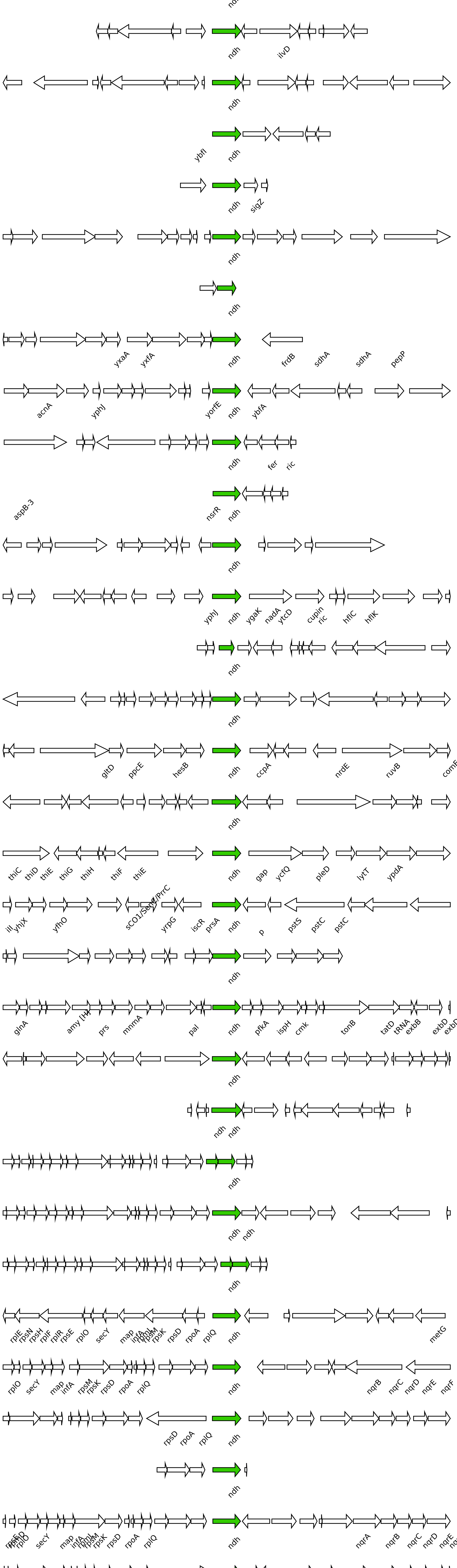

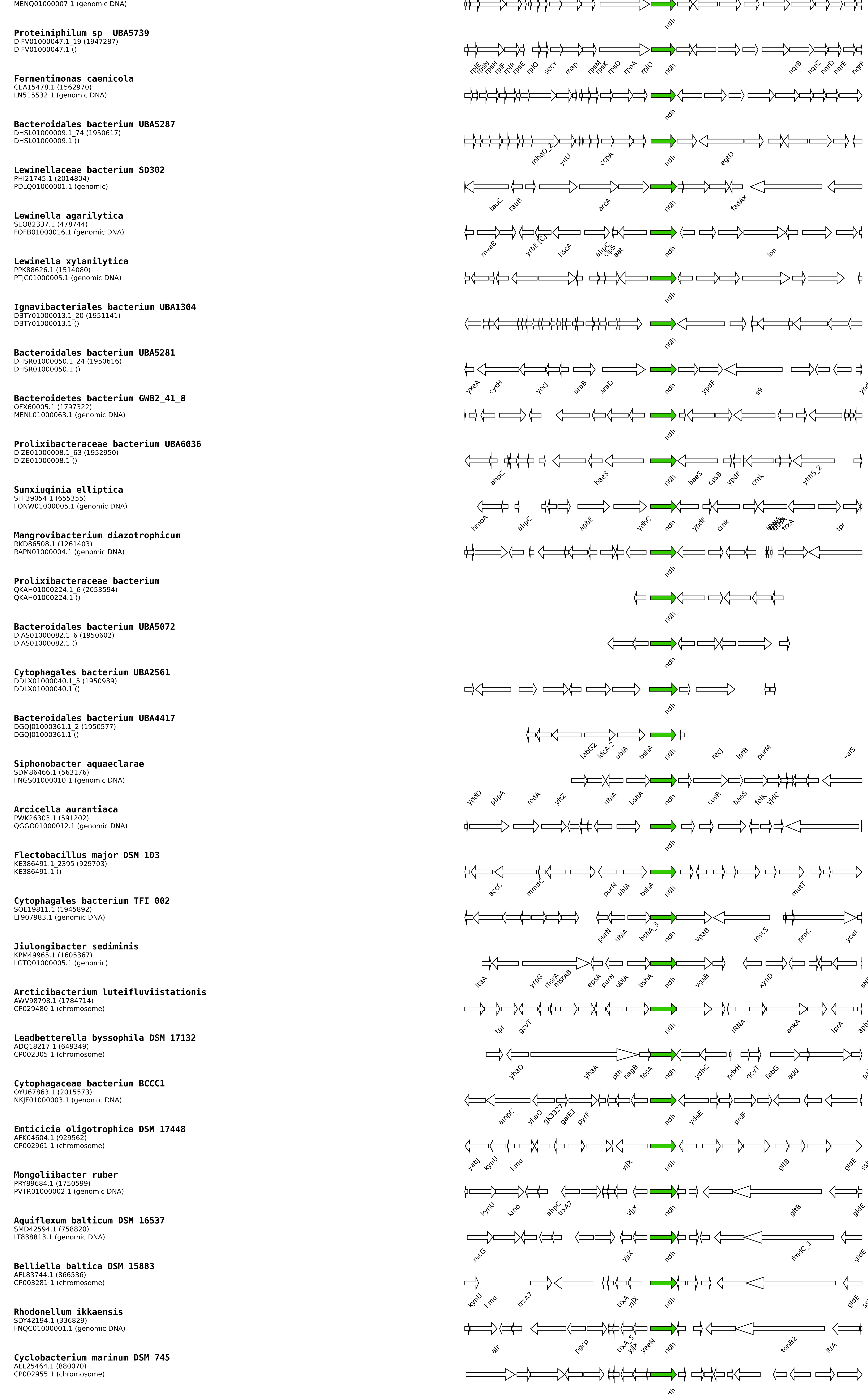
MENQ01000007.1 (genomic DNA)
Proteiniphilum sp UBA5739
DIFV01000047.1_19 (1947287)
DIFV01000047.1 ()
Fermentimonas caenicola
CEA15478.1 (1562970)
LN515532.1 (genomic DNA)
Bacteroidales bacterium UBA5287
DHSL01000009.1_74 (1950617)
DHSL01000009.1 ()
Lewinellaceae bacterium SD302
PHI21745.1 (2014804)
PDLQ01000001.1 (genomic)
Lewinella agarilytica
SEQ82337.1 (478744)
FOFB01000016.1 (genomic DNA)
Lewinella xylanilytica
PPK88626.1 (1514080)
PTJC01000005.1 (genomic DNA)
Ignavibacteriales bacterium UBA1304
DBTY01000013.1_20 (1951141)
DBTY01000013.1 ()
Bacteroidales bacterium UBA5281
DHSR01000050.1_24 (1950616)
DHSR01000050.1 ()
Bacteroidetes bacterium GWB2_41_8
OFX60005.1 (1797322)
MENL01000063.1 (genomic DNA)
Prolixibacteraceae bacterium UBA6036
DIZE01000008.1_63 (1952950)
DIZE01000008.1 ()
Sunxiuqinia elliptica
SFF39054.1 (655355)
FONW01000005.1 (genomic DNA)
Mangrovibacterium diazotrophicum
RKD86508.1 (1261403)
RAPN01000004.1 (genomic DNA)
Prolixibacteraceae bacterium
QKAH01000224.1_6 (2053594)
QKAH01000224.1 ()
Bacteroidales bacterium UBA5072
DIAS01000082.1_6 (1950602)
DIAS01000082.1 ()
Cytophagales bacterium UBA2561
DDLX01000040.1_5 (1950939)
DDLX01000040.1 ()
Bacteroidales bacterium UBA4417
DGQJ01000361.1_2 (1950577)
DGQJ01000361.1 ()
Siphonobacter aquaeclarae
SDM86466.1 (563176)
FNGS01000010.1 (genomic DNA)
Arcicella aurantiaca
PWK26303.1 (591202)
QGGO01000012.1 (genomic DNA)
Flectobacillus major DSM 103
KE386491.1_2395 (929703)
KE386491.1 ()
Cytophagales bacterium TFI 002
SOE19811.1 (1945892)
LT907983.1 (genomic DNA)
Jiulongibacter sediminis
KPM49965.1 (1605367)
LGTQ01000005.1 (genomic)
Arcticibacterium luteifluviistationis
AWV98798.1 (1784714)
CP029480.1 (chromosome)
Leadbetterella byssophila DSM 17132
ADQ18217.1 (649349)
CP002305.1 (chromosome)
Cytophagaceae bacterium BCCC1
OYU67863.1 (2015573)
NKJF01000003.1 (genomic DNA)
Emticicia oligotrophica DSM 17448
AFK04604.1 (929562)
CP002961.1 (chromosome)
Mongoliibacter ruber
PRY89684.1 (1750599)
PVTR01000002.1 (genomic DNA)
Aquiflexum balticum DSM 16537
SMD42594.1 (758820)
LT838813.1 (genomic DNA)
Belliella baltica DSM 15883
AFL83744.1 (866536)
CP003281.1 (chromosome)
Rhodonellum ikkaensis
SDY42194.1 (336829)
FNQC01000001.1 (genomic DNA)
Cyclobacterium marinum DSM 745
AEL25464.1 (880070)
CP002955.1 (chromosome)
ndh

Cyclobacteriaceae bacterium
PXCO01000184.1_54 (2099675)
PXCO01000184.1 ()

Echinicola strongylocentroti
AWW33165.1 (1795355)
CP030041.1 (chromosome)

Algoriphagus sp M8 2
AMQ55140.1 (1727163)
CP012836.1 (chromosome)

Algoriphagus machipongonensis
EAZ81933.1 (388413)
CM001023.1 (chromosome)

Algoriphagus marinus
MSPQ01000001.1_105 (1925762)
MSPQ01000001.1 ()

Algoriphagus aquimarinus
SFA80427.1 (237018)
FOKK01000001.1 (genomic DNA)

Anditalea andensis
KEO73507.1 (1048983)
JMIH01000021.1 (genomic DNA)

Bacteroidetes bacterium HGW Bacteroidetes 1
PKP50120.1 (2013677)
PHDU01000086.1 (genomic DNA)

Bacteroidetes bacterium HGW Bacteroidetes 9
PKP02207.1 (2013699)
PHCY01000053.1 (genomic DNA)

Flavobacteriales bacterium UBA6770
DKGM01000015.1_19 (1951056)
DKGM01000015.1 ()

Flavobacteriales bacterium UBA2108
DCYO01000059.1_9 (1950972)
DCYO01000059.1 ()

Flavobacteriales bacterium UBA7360
DKOO01000014.1_19 (1951066)
DKOO01000014.1 ()

Salinivirga cyanobacteriivorans
ALO17105.1 (1307839)
CP013118.1 (chromosome)

Bacteroidetes bacterium HGW Bacteroidetes 4
PKP09270.1 (2013694)
PHDD01000016.1 (genomic DNA)

Labilibacter aurantiacus
LYBV02000031.1_25 (1849719)
LYBV02000031.1 ()

Saccharicrinis fermentans DSM 9555 JCM 21142
KI912107.1_3776 (869213)
KI912107.1 ()

Marinifilum flexuosum
RKE00079.1 (1117708)
RAPQ01000010.1 (genomic DNA)

Marinifilaceae bacterium T3 2 S1 C
RUT73387.1 (2487017)
RJJX01000022.1 (genomic DNA)

Marinifilaceae bacterium SPP2
BAX80424.1 (1717717)
AP018042.1 (genomic DNA)

Ancylomarina sp M1P
RRG19860.1 (2283627)
QQWG01000016.1 (genomic DNA)

Bacteroidetes bacterium CG_4_10_14_3_um_filter_42_6
PIY34279.1 (1973920)
PFKN01000235.1 (genomic DNA)

Bacteroidales bacterium UBA6244
DJVY01000244.1_2 (1950672)
DJVY01000244.1 ()

Bacteroidetes bacterium GWF2_49_14
OFY48051.1 (1797354)
MEOR01000131.1 (genomic DNA)

Williamwhitmania taraxaci
SDB90832.1 (1640674)
FMYP01000008.1 (genomic DNA)

Flavobacteriales bacterium UBA1993
DDCZ01000041.1_5 (1950965)
DDCZ01000041.1 ()

Flavobacteriales bacterium UBA1991
DDDB01000032.1_104 (1950964)
DDDB01000032.1 ()

Flavobacteriales bacterium UBA6057
DIYJ01000018.1_39 (1951047)
DIYJ01000018.1 ()

Bacteroidales bacterium 6E
GAP70609.1 (1688776)
DF970690.1 (genomic DNA)

Mariniphaga sediminis
RIH63340.1 (1628158)
QWET01000022.1 (genomic DNA)

Tangfeifania diversioriginum
SHJ89338.1 (1168035)
FQZE01000038.1 (genomic DNA)

Arcticibacter pallidicorallinus
PRY49862.1 (1259464)

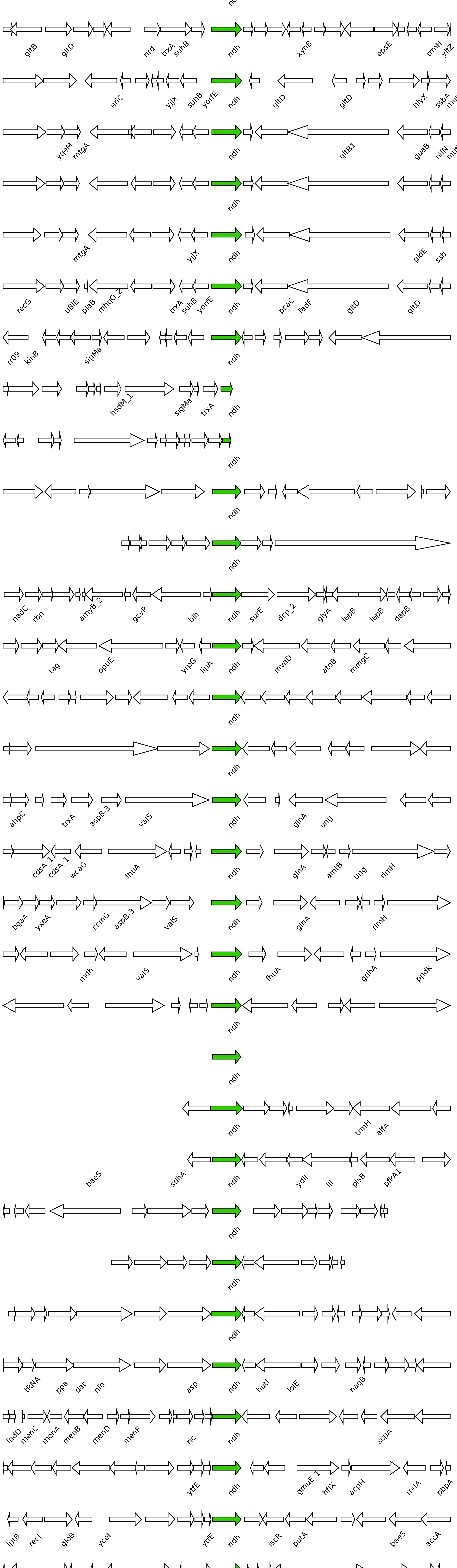

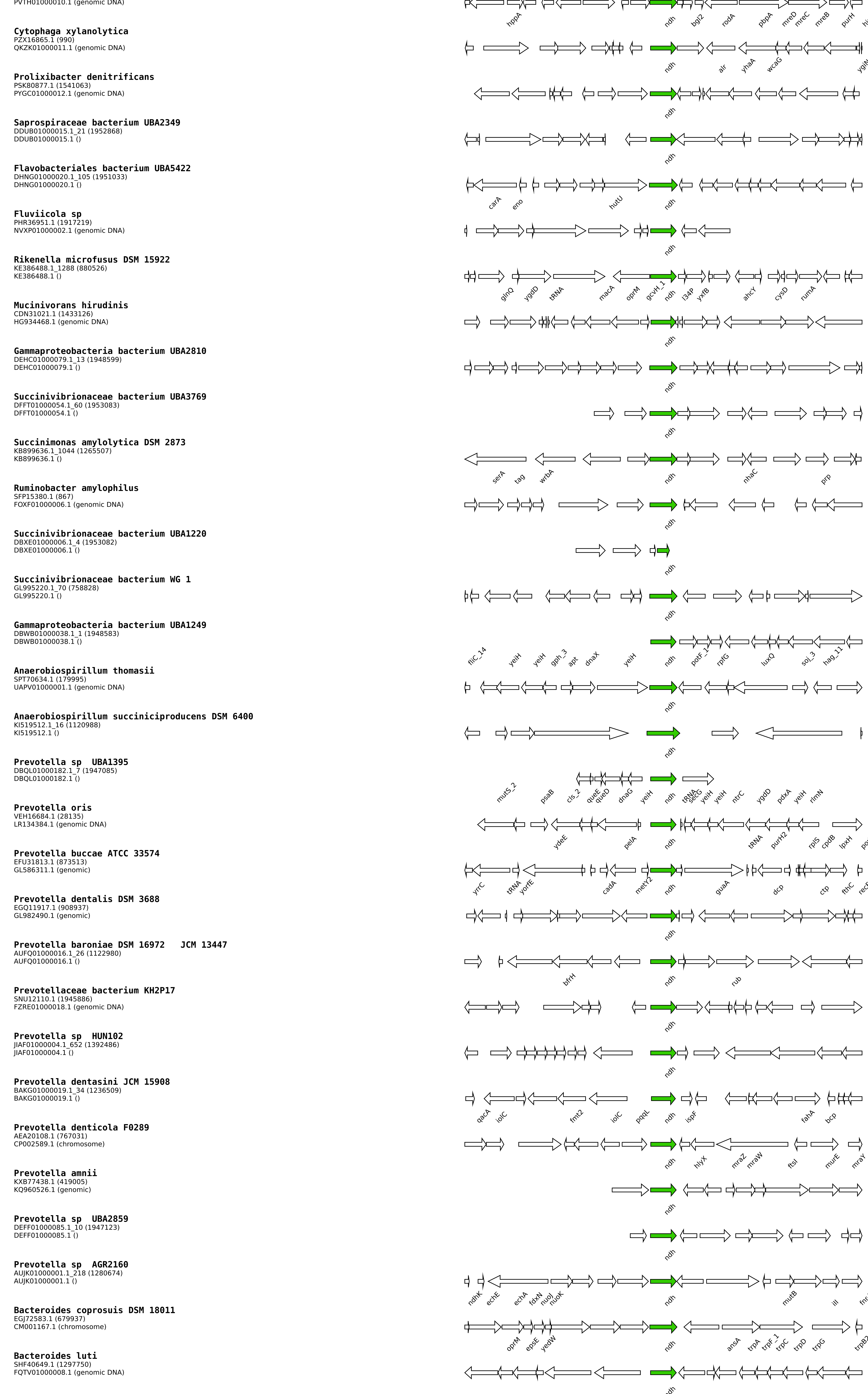
PVTH01000010.1 (genomic DNA)
hppA bgl2 rodA pbpA mreD mreC mreB purH
Cytophaga xylanolytica
PZX16865.1 (990)
QKZK01000011.1 (genomic DNA)
ndh alr yhaA wcaG
Prolixibacter denitrificans
PSK80877.1 (1541063)
PYGC01000012.1 (genomic DNA)
ndh
Saprospiraceae bacterium UBA2349
DDUB01000015.1_21 (1952868)
DDUB01000015.1 ()
ndh
Flavobacteriales bacterium UBA5422
DHNG01000020.1_105 (1951033)
DHNG01000020.1 ()
carA eno hutU ndh
Fluviicola sp
PHR36951.1 (1917219)
NVXP01000002.1 (genomic DNA)
ndh
Rikenella microfusus DSM 15922
KE386488.1_1288 (880526)
KE386488.1 ()
glnQ ygdD tRNA macA oprM gcvH_1 ndh l34P yxfB ahcY cysD rumA
Mucinivorans hirudinis
CDN31021.1 (1433126)
HG934468.1 (genomic DNA)
ndh
Gammaproteobacteria bacterium UBA2810
DEHC01000079.1_13 (1948599)
DEHC01000079.1 ()
ndh
Succinivibrionaceae bacterium UBA3769
DFFT01000054.1_60 (1953083)
DFFT01000054.1 ()
ndh
Succinimonas amylolytica DSM 2873
KB899636.1_1044 (1265507)
KB899636.1 ()
serA tag wrbA ndh nhaC prp
Ruminobacter amylophilus
SFP15380.1 (867)
FOXF01000006.1 (genomic DNA)
ndh
Succinivibrionaceae bacterium UBA1220
DBXE01000006.1_4 (1953082)
DBXE01000006.1 ()
ndh
Succinivibrionaceae bacterium WG 1
GL995220.1_70 (758828)
GL995220.1 ()
ndh
Gammaproteobacteria bacterium UBA1249
DBWB01000038.1_1 (1948583)
DBWB01000038.1 ()
fliC_14 yeiH yeiH gph_3 apt dnaX yeiH ndh potF_1 rpfG luxQ soj_3 hag_11
Anaerobiospirillum thomasii
SPT70634.1 (179995)
UAPV01000001.1 (genomic DNA)
ndh
Anaerobiospirillum succiniciproducens DSM 6400
KI519512.1_16 (1120988)
KI519512.1 ()
ndh
Prevotella sp UBA1395
DBQL01000182.1_7 (1947085)
DBQL01000182.1 ()
mutS_2 psaB cls_2 queE queD dnaG yeiH ndh tRNA secG yeiH yeiH ntrC ygdD pdxA yeiH rlmN
Prevotella oris
VEH16684.1 (28135)
LR134384.1 (genomic DNA)
ydeE pelA ndh tRNA purH2 rplS cpdB lpxH
Prevotella buccae ATCC 33574
EFU31813.1 (873513)
GL586311.1 (genomic)
yrrC tRNA yorfE cadA metY2 ndh guaA dcp ctp fthC
Prevotella dentalis DSM 3688
EGQ11917.1 (908937)
GL982490.1 (genomic)
ndh
Prevotella baroniae DSM 16972 JCM 13447
AUFQ01000016.1_26 (1122980)
AUFQ01000016.1 ()
bfrH ndh rub
Prevotellaceae bacterium KH2P17
SNU12110.1 (1945886)
FZRE01000018.1 (genomic DNA)
ndh
Prevotella sp HUN102
JIAF01000004.1_652 (1392486)
JIAF01000004.1 ()
ndh
Prevotella dentasini JCM 15908
BAKG01000019.1_34 (1236509)
BAKG01000019.1 ()
qacA iolC fmt2 iolC pqqL ndh ispF fahA bcp
Prevotella denticola F0289
AEA20108.1 (767031)
CP002589.1 (chromosome)
ndh hlyX mraZ mraW ftsI murE mraY
Prevotella amnii
KXB77438.1 (419005)
KQ960526.1 (genomic)
ndh
Prevotella sp UBA2859
DEFF01000085.1_10 (1947123)
DEFF01000085.1 ()
ndh
Prevotella sp AGR2160
AUJK01000001.1_218 (1280674)
AUJK01000001.1 ()
ndhK echE echA fdxN nuoJ nuoK ndh mutB ilI
Bacteroides coprosuis DSM 18011
EGJ72583.1 (679937)
CM001167.1 (chromosome)
oprM epsE yedW ndh ansA trpA trpF_1 trpC trpD trpG
Bacteroides luti
SHF40649.1 (1297750)
FQTV01000008.1 (genomic DNA)

Bacteroides sp UBA1317
DBTL01000111.1_28 (1946083)
DBTL01000111.1 ()

Bacteroides sp CAG 462
CDD83996.1 (1262740)
FR896530.1 (genomic DNA)

Prevotella sp CAG 1320
CDA93883.1 (1262922)
FR882118.1 (genomic DNA)

Bacteroides sp AF16 49
RHR81585.1 (2292192)
QUHA01000003.1 (genomic DNA)

Coprobacter fastidiosus
PWM12185.1 (1099853)
QALP01000006.1 (genomic DNA)

Bacteroides thetaiotaomicron VPI 5482
AAO76644.1 (226186)
AE015928.1 (chromosome)

Bacteroides reticulotermitis JCM 10512
GAE83681.1 (1445607)
BAIV01000010.1 (genomic DNA)

Bacteroides paurosaccharolyticus
DHEE01000033.1_7 (732242)
DHEE01000033.1 ()

Prevotella sp CAG 755
CCY15691.1 (1262935)
HF989227.1 (genomic DNA)

Prevotella sp CAG 617
CDF21336.1 (1262933)
FR903617.1 (genomic DNA)

Prevotella sp MGM2
GAY30428.1 (2033406)
BEWW01000259.1 (genomic DNA)

Prevotella sp CAG 5226
CDA42001.1 (1262930)
FR879216.1 (genomic DNA)

Prevotella sp CAG 891
CDE87616.1 (1262937)
FR901692.1 (genomic DNA)

Alloprevotella sp
RKV74033.1 (1872471)
RBJJ01000022.1 (genomic DNA)

Prevotella sp oral taxon 473 str F0040
EKX94059.1 (1035197)
KB290705.1 (genomic)

Alloprevotella sp OH1205_COT 284
RRD80227.1 (2491043)
RQYI01000007.1 (genomic DNA)

Alloprevotella rava F0323
EHG20855.1 (679199)
JH376839.1 (genomic)

Prevotella saccharolytica F0055
EKY02372.1 (1127699)
KB290980.1 (genomic)

Prevotella sp DNF00663
KXB82731.1 (1384078)
KQ960625.1 (genomic)

Prevotella sp CAG 1058
CCX64940.1 (1262918)
HF986718.1 (genomic DNA)

Prevotella sp CAG 924
CCY02749.1 (1262938)
HF988619.1 (genomic DNA)

Parabacteroides sp Marseille P3160
LT799418.1_3709 (1917887)
LT799418.1 ()

Prevotella sp AG 487_50_53
OKZ27185.1 (1897014)
MNQZ01000017.1 (genomic DNA)

Prevotella sp CAG 520
CDB04661.1 (1262929)
FR882709.1 (genomic DNA)

Prevotella copri DSM 18205
EFB35836.1 (537011)
GG703853.1 (genomic)

Prevotella sp UBA2726
DEKI01000003.1_33 (1947097)
DEKI01000003.1 ()

Prevotella multisaccharivorax DSM 17128
EGN56106.1 (688246)
GL945017.1 (genomic)

Prevotella sp UBA7770
DLDO01000087.1_2 (1947235)
DLDO01000087.1 ()

Prevotella sp UBA7765
DLDT01000063.1_31 (1947234)
DLDT01000063.1 ()

Bacteroidales bacterium UBA1309
DBTT01000050.1_35 (1950405)
DBTT01000050.1 ()

Bacteroidales bacterium UBA1309
DBTT01000054.1_145 (1950405)

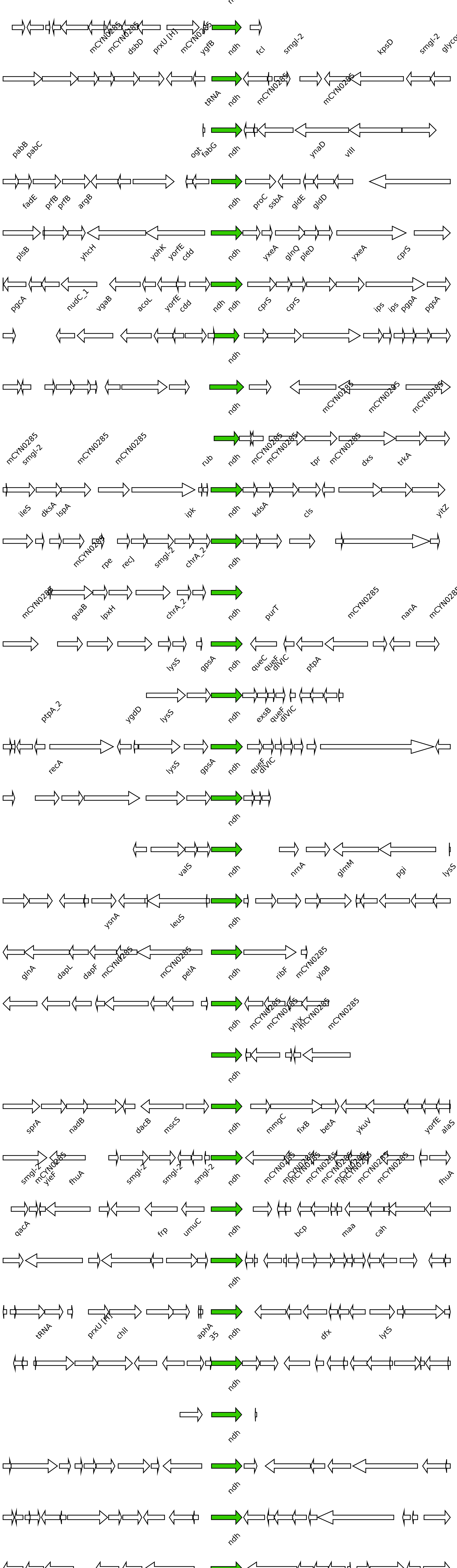

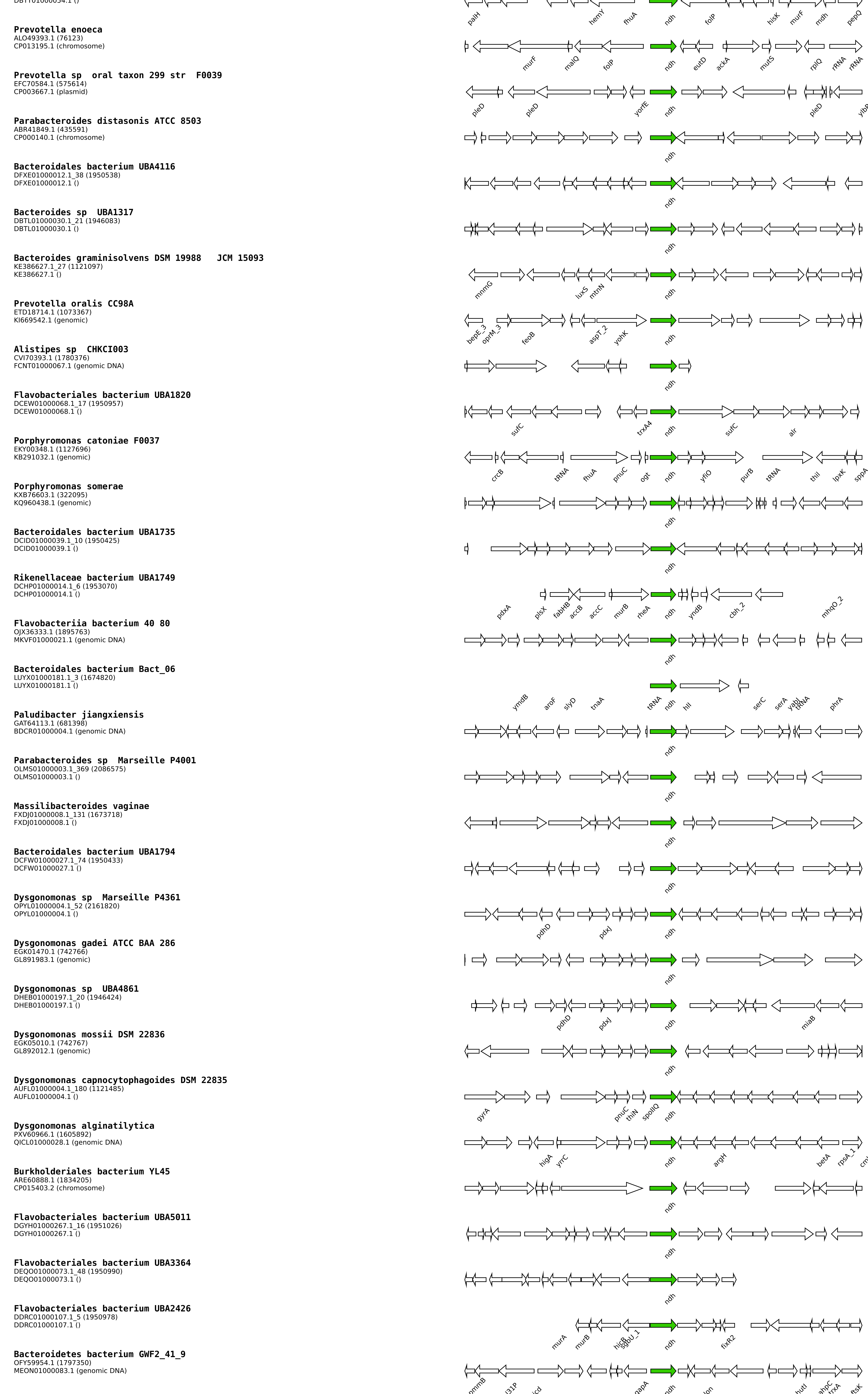
DBTT01000034.1 ()
palH
hemY
fhuA
ndh
folP
hisK
murF
mdh
pepQ
Prevotella enoeca
ALO49393.1 (76123)
CP013195.1 (chromosome)
murF
malQ
folP
ndh
eutD
ackA
mutS
rplQ
rRNA
rRNA
Prevotella sp oral taxon 299 str F0039
EFC70584.1 (575614)
CP003667.1 (plasmid)
pleD
pleD
yorfE
ndh
pleD
Parabacteroides distasonis ATCC 8503
ABR41849.1 (435591)
CP000140.1 (chromosome)
ndh
Bacteroidales bacterium UBA4116
DFXE01000012.1_38 (1950538)
DFXE01000012.1 ()
ndh
Bacteroides sp UBA1317
DBTL01000030.1_21 (1946083)
DBTL01000030.1 ()
ndh
Bacteroides graminisolvens DSM 19988 JCM 15093
KE386627.1_27 (1121097)
KE386627.1 ()
mnmG
luxS
mtnN
ndh
Prevotella oralis CC98A
ETD18714.1 (1073367)
KI669542.1 (genomic)
bepE_3
oprM_3
feoB
aspT_2
yohK
ndh
Alistipes sp CHKCI003
CVI70393.1 (1780376)
FCNT01000067.1 (genomic DNA)
ndh
Flavobacteriales bacterium UBA1820
DCEW01000068.1_17 (1950957)
DCEW01000068.1 ()
sufC
trxA4
ndh
sufC
alr
Porphyromonas catoniae F0037
EKY00348.1 (1127696)
KB291032.1 (genomic)
crcB
tRNA
fhuA
pnuC
ogt
ndh
yfiO
purB
tRNA
thiI
lpxK
sppA
Porphyromonas somerae
KXB76603.1 (322095)
KQ960438.1 (genomic)
ndh
Bacteroidales bacterium UBA1735
DCID01000039.1_10 (1950425)
DCID01000039.1 ()
ndh
Rikenellaceae bacterium UBA1749
DCHP01000014.1_6 (1953070)
DCHP01000014.1 ()
pdxA
plsX
fabHB
accB
accC
murB
rheA
ndh
yndB
cbh_2
mhqO_2
Flavobacteriia bacterium 40 80
OJX36333.1 (1895763)
MKVF01000021.1 (genomic DNA)
ndh
Bacteroidales bacterium Bact_06
LUYX01000181.1_3 (1674820)
LUYX01000181.1 ()
ymdB
aroF
slyD
tnaA
tRNA
ndh
hll
serC
serA
yabJ
tRNA
phrA
Paludibacter jiangxiensis
GAT64113.1 (681398)
BDCR01000004.1 (genomic DNA)
ndh
Parabacteroides sp Marseille P4001
OLMS01000003.1_369 (2086575)
OLMS01000003.1 ()
ndh
Massilibacteroides vaginae
FXDJ01000008.1_131 (1673718)
FXDJ01000008.1 ()
ndh
Bacteroidales bacterium UBA1794
DCFW01000027.1_74 (1950433)
DCFW01000027.1 ()
ndh
Dysgonomonas sp Marseille P4361
OPYL01000004.1_52 (2161820)
OPYL01000004.1 ()
pdhD
pdxJ
ndh
Dysgonomonas gadei ATCC BAA 286
EGK01470.1 (742766)
GL891983.1 (genomic)
ndh
Dysgonomonas sp UBA4861
DHEB01000197.1_20 (1946424)
DHEB01000197.1 ()
pdhD
pdxJ
ndh
miaB
Dysgonomonas mossii DSM 22836
EGK05010.1 (742767)
GL892012.1 (genomic)
ndh
Dysgonomonas capnocytophagoides DSM 22835
AUFL01000004.1_180 (1121485)
AUFL01000004.1 ()
gyrA
pnuC
thiN
spoIIQ
ndh
Dysgonomonas alginatilytica
PXV60966.1 (1605892)
QICL01000028.1 (genomic DNA)
higA
yrrC
ndh
argH
betA
rpsA_1
Burkholderiales bacterium YL45
ARE60888.1 (1834205)
CP015403.2 (chromosome)
ndh
Flavobacteriales bacterium UBA5011
DGYH01000267.1_16 (1951026)
DGYH01000267.1 ()
ndh
Flavobacteriales bacterium UBA3364
DEQO01000073.1_48 (1950990)
DEQO01000073.1 ()
ndh
Flavobacteriales bacterium UBA2426
DDRC01000107.1_5 (1950978)
DDRC01000107.1 ()
murA
murB
higB
sgbU_1
ndh
fixR2
Bacteroidetes bacterium GWF2_41_9
OFY59954.1 (1797350)
MEON01000083.1 (genomic DNA)

Psychroflexus salarius
SHE78264.1 (1155689)
FQTW01000005.1 (genomic DNA)

Marinoscillum furvescens DSM 4134
REE05702.1 (1122208)
QREG01000001.1 (genomic DNA)

Saprospiraceae bacterium UBA3362
DEQQ01000063.1_8 (1952878)
DEQQ01000063.1 ()

Porphyromonadaceae bacterium UBA7332
DKPO01000016.1_156 (1952687)
DKPO01000016.1 ()

Coprobacter sp
PWM88385.1 (1941478)
QAMD01000027.1 (genomic DNA)

Barnesiella viscericola DSM 18177
AHF12018.1 (880074)
CP007034.1 (chromosome)

Barnesiella intestinihominis
HAC14217.1 (487174)
DLXC01000064.1 (genomic DNA)

Prevotella sp CAG 485
CDE09433.1 (1262927)
FR897821.1 (genomic DNA)

Porphyromonadaceae bacterium UBA7082
DKZE01000064.1_2 (1952623)
DKZE01000064.1 ()

Porphyromonadaceae bacterium UBA3314
DESM01000048.1_62 (1952566)
DESM01000048.1 ()

Porphyromonadaceae bacterium UBA7154
DKWK01000067.1_8 (1952655)
DKWK01000067.1 ()

Bacteroidales bacterium H10
DAAV01000148.1_5 (1807758)
DAAV01000148.1 ()

Prevotella sp CAG 279
CDE40215.1 (1262924)
FR899590.1 (genomic DNA)

Bacteroidales bacterium M7
LUJR01000035.1_122 (1807735)
LUJR01000035.1 ()

Synergistes sp Zagget9
MSHK01000031.1_10 (1930003)
MSHK01000031.1 ()

Bacteroidales bacterium M3
LUJN01000010.1_96 (1807731)
LUJN01000010.1 ()

Bacteroidales bacterium GP4
DAAL01000079.1_16 (1807748)
DAAL01000079.1 ()

Bacteroidales bacterium H7
DAAS01000009.1_102 (1807755)
DAAS01000009.1 ()

Muribaculum intestinale
ANU63960.1 (1796646)
CP015402.2 (chromosome)

Muribaculaceae bacterium Zag1
NWBJ01000034.1_104 (1958821)
NWBJ01000034.1 ()

Bacteroidales bacterium M9
LUJT01000077.1_15 (1807737)
LUJT01000077.1 ()

Prevotella sp CAG 873
CDE57662.1 (1262936)
FR900385.1 (genomic DNA)

Prevotella sp CAG 1031
CCX43193.1 (1262917)
HF985559.1 (genomic DNA)

Bacteroidales bacterium M1
LUJL01000024.1_126 (1807729)
LUJL01000024.1 ()

Bacteroidales bacterium M13
LUJX01000013.1_12 (1807741)
LUJX01000013.1 ()

Bacteroidales bacterium M2
LUJM01000051.1_32 (1807730)
LUJM01000051.1 ()

Bacteroides sp CAG 927
CCX46622.1 (1262753)
HF985620.1 (genomic DNA)

Bacteroidales bacterium M6
LUJQ01000057.1_2 (1807734)
LUJQ01000057.1 ()

Porphyromonadaceae bacterium UBA7077
DKZJ01000005.1_53 (1952620)
DKZJ01000005.1 ()

Porphyromonadaceae bacterium
HAB40380.1 (2049046)
DLWC01000009.1 (genomic DNA)

Dyadobacter jejuensis
PWJ56846.1 (1082580)
QGDT01000010.1 (genomic DNA)

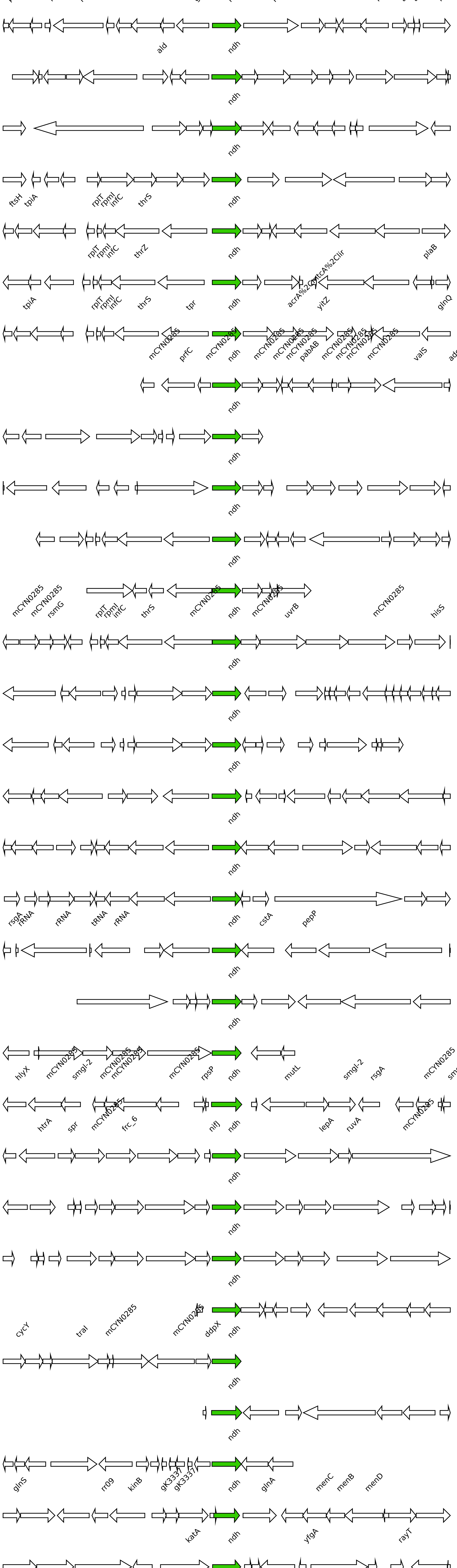

Arachidicoccus rhizosphaerae
SEA16643.1 (551991)
FNQY01000009.1 (genomic DNA)

Bacteroidetes bacterium 43 16
OJV55280.1 (1895923)
MKTB01000005.1 (genomic DNA)

Taibaiella sp KBW10
RQO31375.1 (2153357)
QAJI01000003.1 (genomic DNA)

Sphingobacteriales bacterium 40 81
OJY99059.1 (1895835)
MKWF01000008.1 (genomic DNA)

Cnuella takakiae
OLY90782.1 (1302690)
MTFE01000010.1 (genomic)

Flexibacter flexilis DSM 6793
SFC67808.1 (927664)
FOLE01000008.1 (genomic DNA)

Pedobacter sp FJ4 8
PWG82186.1 (2173175)
QEAS01000002.1 (genomic DNA)

Chitinophagaceae bacterium UBA2791
DEHV01000059.1_14 (1951552)
DEHV01000059.1 ()

Bacteroidetes bacterium UBA1952
DDEO01000238.1_2 (1953143)
DDEO01000238.1 ()

Pedobacter sp SM1810
QRGB01000001.1_2927 (2292082)
QRGB01000001.1 ()

Ferruginibacter sp BO 59
RNI40323.1 (2294116)
RJJR01000001.1 (genomic DNA)

Parapedobacter luteus
SKB33617.1 (623280)
FUYS01000002.1 (genomic DNA)

bacterium A37T11
SEM93995.1 (1855384)
FOBR01000009.1 (genomic DNA)

Chitinophagaceae bacterium UBA2791
DEHV01000059.1_15 (1951552)
DEHV01000059.1 ()

Chryseobacterium sp P1 3
KFF74638.1 (1517683)
JPEQ01000007.1 (genomic)

Sphingobacterium psychroaquaticum
SMG16938.1 (561061)
FXAU01000001.1 (genomic DNA)

Olivibacter sitiensis DSM 17696
ATZA01000001.1_183 (1122621)
ATZA01000001.1 ()

Parafilimonas terrae
SFP84121.1 (1465490)
FOXQ01000002.1 (genomic DNA)

Dyadobacter jejuensis
PWJ58849.1 (1082580)
QGDT01000003.1 (genomic DNA)

Pseudopedobacter saltans DSM 12145
ADY54224.1 (762903)
CP002545.1 (chromosome)

Cytophagaceae bacterium SCN 52 12
ODS73148.1 (1660162)
MEDT01000255.1 (genomic DNA)

Sphingobacteriales bacterium 44 61
OJW03852.1 (1895838)
MKTW01000002.1 (genomic DNA)

Flavobacterium sp
MBE99822.1 (239)
PAXH01000006.1 (genomic DNA)

Flavobacterium album
AWH87020.1 (2175091)
CP029186.1 (chromosome)

Flavobacterium tegetincola DSM 22377
AUDN01000007.1_194 (1121901)
AUDN01000007.1 ()

Flavobacterium sp JRM
KIA95564.1 (1566022)
JSYO01000093.1 (genomic DNA)

Niabella ginsenosidivorans
ANH82959.1 (1176587)
CP015772.1 (chromosome)

Sphingobacteriales bacterium 41 5
OJU24886.1 (1895836)
MKRO01000159.1 (genomic DNA)

Niabella yanshanensis
QLKR01000002.1_516 (577386)
QLKR01000002.1 ()

Capnocytophaga haemolytica
AMD84992.1 (45243)
CP014227.1 (chromosome)

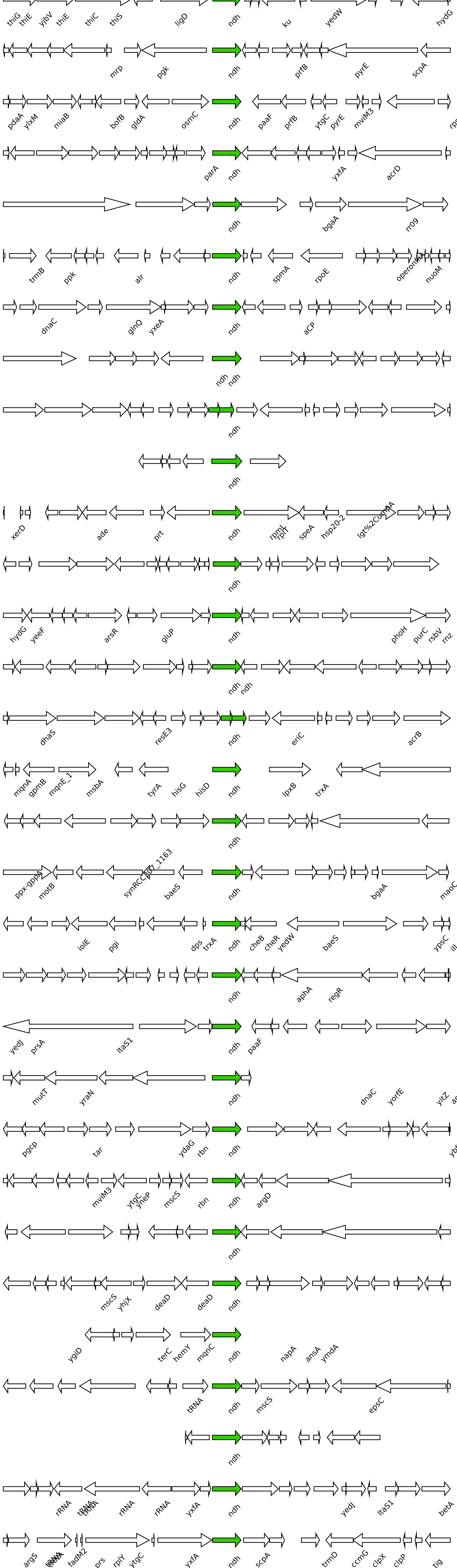

Capnocytophaga sputigena
ATA83674.1 (1019)
CP022385.1 (chromosome)

Tannerella sp oral taxon HOT 286
AOH40844.1 (712710)
CP017038.2 (chromosome)

Joostella marina DSM 19592
EIJ38992.1 (926559)
JH651379.1 (genomic)

Formosa agariphila KMM 3901
CDF78476.1 (1347342)
HG315671.1 (genomic DNA)

Arenibacter sp AK53
MTAZ01000002.1_34 (1406873)
MTAZ01000002.1 ()

Tenacibaculum sp DSM 106434
AZJ36850.1 (2358479)
CP032548.1 (chromosome)

Flavobacterium gelidilacus DSM 15343
AUGN01000001.1_451 (1121891)
AUGN01000001.1 ()

Lewinella xylanilytica
PPK84757.1 (1514080)
PTJC01000007.1 (genomic DNA)

Leeuwenhoekiella blandensis MED217
EAQ50307.1 (398720)
CH672395.1 (genomic)

Flavobacteriaceae bacterium MAR_2010_188
SDB63392.1 (1250295)
LT629302.1 (genomic DNA)

Pustulibacterium marinum
SFU38821.1 (1224947)
FPBK01000002.1 (genomic DNA)

Candidatus Korarchaeota archaeon
PMB78870.1 (2056630)
PNIS01000389.1 (genomic DNA)

Nitrosospira multiformis ATCC 25196
ABB75296.1 (323848)
CP000103.1 (chromosome)

Flavobacteriaceae bacterium UBA3168
DEYC01000113.1_5 (1951823)
DEYC01000113.1 ()

Olivibacter sitiensis DSM 17696
ATZA01000051.1_8 (1122621)
ATZA01000051.1 ()

Psychroflexus halocasei
SDZ86627.1 (908615)
FNQF01000002.1 (genomic DNA)

Robiginitalea myxolifaciens
SFR31342.1 (400055)
FOYQ01000001.1 (genomic DNA)

Apibacter mensalis
LIVM01000004.1_163 (1586267)
LIVM01000004.1 ()

Sinomicrobium oceani
SFW19771.1 (1150368)
FPJE01000002.1 (genomic DNA)

Cardiobacterium hominis ATCC 15826
EEV87628.1 (638300)
GG694027.1 (genomic)

Bacteroidetes bacterium
PCH66464.1 (1898104)
NVRJ01000069.1 (genomic DNA)

Saprospiraceae bacterium UBA7328
DKPS01000101.1_36 (1952882)
DKPS01000101.1 ()

Alistipes indistinctus
RGU38207.1 (626932)
QRXW01000001.1 (genomic DNA)

Pseudopedobacter saltans
PZP42622.1 (151895)
QFOI01000422.1 (genomic DNA)

Abditibacterium utsteinense
PQV64076.1 (1960156)
NIGF01000007.1 (genomic DNA)

Chloroflexi bacterium UBA5183
DHWL01000139.1_143 (1947834)
DHWL01000139.1 ()

Arthrospira sp O9 13F
RAQ45862.1 (1982223)
PKGD01000100.1 (genomic DNA)

Spirochaetia bacterium UBA5550
DINC01000165.1_9 (1948759)
DINC01000165.1 ()

Deltaproteobacteria bacterium CG11_big_fil_rev_8_21_14_0_20_49_13
PIR18099.1 (1973976)
PCWZ01000018.1 (genomic DNA)

Schwartzia succinivorans DSM 10502
SHE94650.1 (1123243)
FQUG01000005.1 (genomic DNA)

Selenomonas sputigena ATCC 35185
EEX78541.1 (546271)
GG698596.1 (genomic)

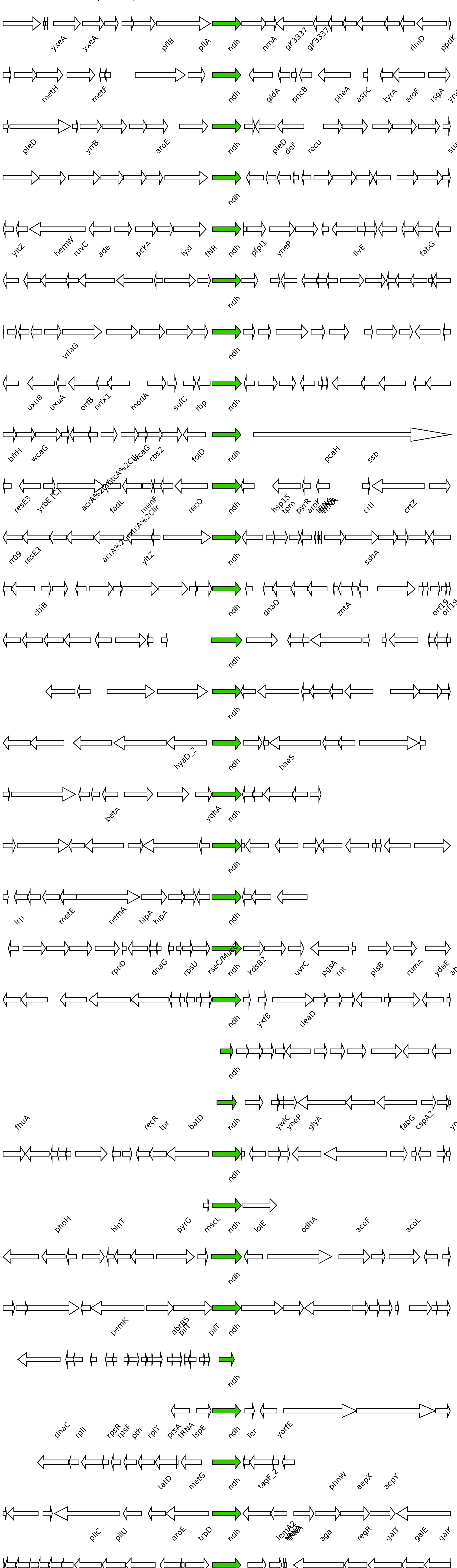

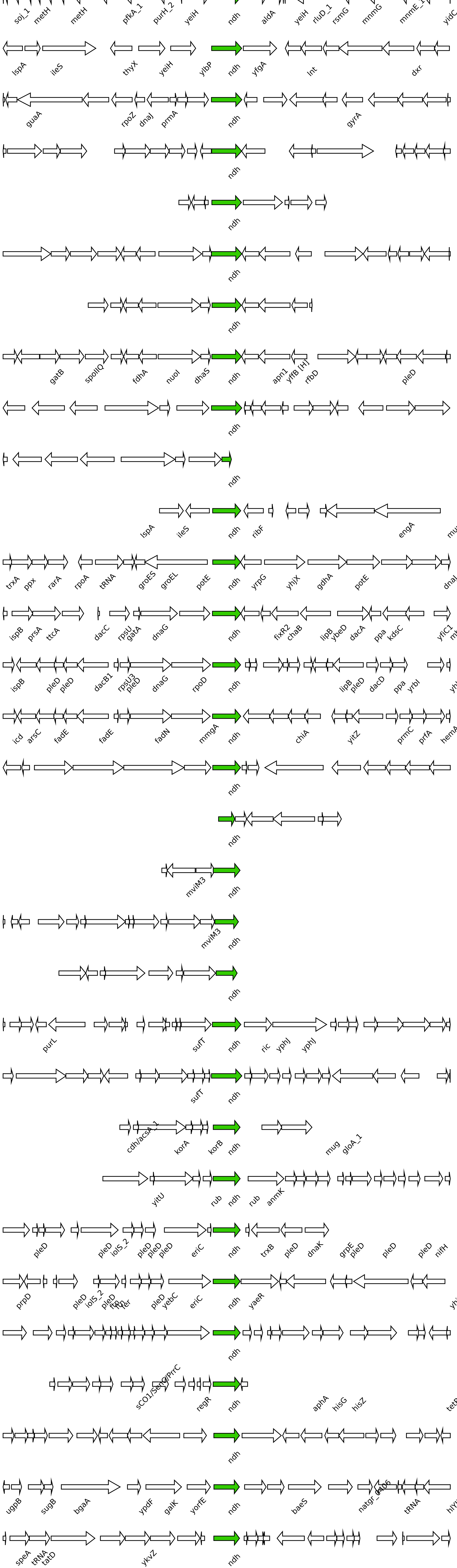

Mitsuokella jalaludinii
CUN48930.1 (187979)
CYYU01000002.1 (genomic DNA)

Fimbriimonas ginsengisoli Gsoil 348
AIE83465.1 (661478)
CP007139.1 (chromosome)

Armatimonadetes bacterium 55 13
OJU62227.1 (1895717)
MKRX01000018.1 (genomic DNA)

Planctomycetaceae bacterium UBA1338
DBSQ01000292.1_5 (1952728)
DBSQ01000292.1 ()

Phycisphaerales bacterium UBA3160
DEYK01000054.1_117 (1951197)
DEYK01000054.1 ()

Phycisphaerales bacterium UBA3472
DFRD01000105.1_5 (1951199)
DFRD01000105.1 ()

Phycisphaerales bacterium UBA3158
DEYM01000057.1_20 (1951196)
DEYM01000057.1 ()

Sphaerobacter thermophilus DSM 20745
ACZ39088.1 (479434)
CP001823.1 (chromosome)

Sphaerobacteraceae bacterium
PWOJ01000431.1_8 (2099683)
PWOJ01000431.1 ()

Candidatus Marinamargulisbacteria bacterium SCGC AG 414 C22
RAP29930.1 (2184344)
QFBR01000033.1 (genomic DNA)

Francisella sp CA97 1460
APC97481.1 (1542390)
CP009654.1 (chromosome)

Allofrancisella guangzhouensis
AJC49458.1 (594679)
CP010427.1 (chromosome)

Francisella sp FSC1006
AIT08651.1 (1547445)
CP009574.1 (chromosome)

Francisella tularensis subsp tularensis SCHU S4
CAG45667.1 (177416)
AJ749949.2 (genomic DNA)

Francisella sp FDC440
AXA33373.1 (2007306)
CP021781.1 (chromosome)

Cyanobacteria bacterium UBA999
DBAX01000076.1_1 (1947904)
DBAX01000076.1 ()

Cyanobacteria bacterium UBA8156
HAN47099.1 (2055767)
DMPD01000346.1 (genomic DNA)

candidate division Zixibacteria bacterium RBG_19FT_COMBO_42_43
OGC85922.1 (1802788)
MEWQ01000021.1 (genomic DNA)

candidate division Zixibacteria bacterium RBG_16_48_11
OGC91223.1 (1802785)
MEWN01000095.1 (genomic DNA)

Geopsychrobacter electrodiphilus DSM 16401
ARWE01000001.1_3577 (1121918)
ARWE01000001.1 ()

Ignavibacteriales bacterium UTCHB2
OQY71923.1 (1961105)
MWSV01000141.1 (genomic DNA)

Ignavibacteria bacterium CG1_02_37_35
OIO21743.1 (1805220)
MNVA01000048.1 (genomic DNA)

Candidatus Scalindua brodae
KHE93033.1 (237368)
JRYO01000081.1 (genomic DNA)

Candidatus Brocadia sapporoensis
OQD46847.1 (392547)
MJUW02000019.1 (genomic)

Candidatus Jettenia caeni
GAB62087.1 (247490)
BAFH01000003.1 (genomic DNA)

Candidatus Brocadia sinica JPN1
GAN35226.1 (1197129)
BAFN01000001.1 (genomic DNA)

Candidatus Kuenenia stuttgartiensis
DIDR01000133.1_2 (174633)
DIDR01000133.1 ()

Deinococcus maricopensis DSM 21211
ADV67796.1 (709986)
CP002454.1 (chromosome)

Deinococcus misasensis DSM 22328
JQKG01000029.1_18 (1121382)
JQKG01000029.1 ()

Deinococcus peraridilitoris DSM 19664
AFZ65947.1 (937777)
CP003382.1 (chromosome)

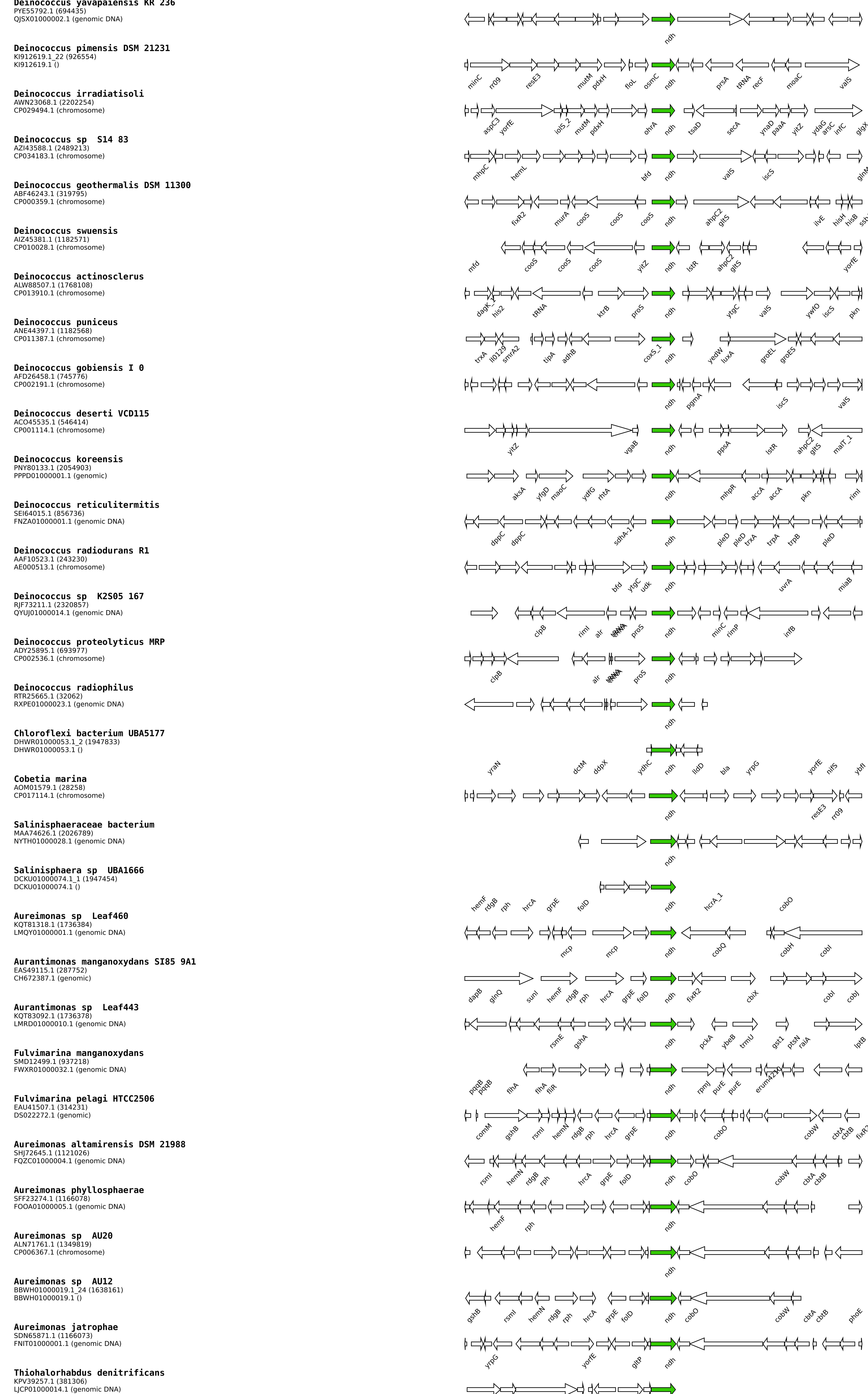
Deinococcus yavapaiensis KR 236
PYE55792.1 (694435)
QJSX01000002.1 (genomic DNA)
ndh
Deinococcus pimensis DSM 21231
KI912619.1_22 (926554)
KI912619.1 ()
minC rr09 resE3 mutM pdxH floL osmC ndh prsA tRNA recF moaC valS
Deinococcus irradiatisoli
AWN23068.1 (2202254)
CP029494.1 (chromosome)
aspC3 yorfE iolS_2 mutM pdxH ohrA ndh tsaD secA ynaD paaA yitZ ydaG arsC infC glgX
Deinococcus sp S14 83
AZI43588.1 (2489213)
CP034183.1 (chromosome)
mhpC hemL bfd ndh valS iscS
Deinococcus geothermalis DSM 11300
ABF46243.1 (319795)
CP000359.1 (chromosome)
fixR2 murA cooS cooS cooS ndh ahpC2 gltS ilvE hisH hisB
Deinococcus swuensis
AIZ45381.1 (1182571)
CP010028.1 (chromosome)
mfd cooS cooS cooS yitZ ndh lstR ahpC2 gltS yorfE
Deinococcus actinosclerus
ALW88507.1 (1768108)
CP013910.1 (chromosome)
dagK_1 his2 tRNA ktrB proS ndh ytgC valS ywfO iscS pkn
Deinococcus puniceus
ANE44397.1 (1182568)
CP011387.1 (chromosome)
trxA ll0129 smrA2 tipA adhB coxS_1 ndh yedW luxA groEL groES
Deinococcus gobiensis I 0
AFD26458.1 (745776)
CP002191.1 (chromosome)
ndh pgmA iscS valS
Deinococcus deserti VCD115
ACO45535.1 (546414)
CP001114.1 (chromosome)
yitZ vgaB ndh ppsA lstR ahpC2 gltS malT_1
Deinococcus koreensis
PNY80133.1 (2054903)
PPPD01000001.1 (genomic)
aksA yfgD maoC ydfG rhtA ndh mhpR accA accA pkn rimI
Deinococcus reticulitermitis
SEI64015.1 (856736)
FNZA01000001.1 (genomic DNA)
dppC dppC sdhA-1 ndh pleD pleD trxA trpA trpB pleD
Deinococcus radiodurans R1
AAF10523.1 (243230)
AE000513.1 (chromosome)
bfd ytgC udk ndh uvrA miaB
Deinococcus sp K2S05 167
RJF73211.1 (2320857)
QYUJ01000014.1 (genomic DNA)
clpB rimI alr tRNA tRNA proS ndh minC rimP infB
Deinococcus proteolyticus MRP
ADY25895.1 (693977)
CP002536.1 (chromosome)
clpB alr tRNA tRNA proS ndh
Deinococcus radiophilus
RTR25665.1 (32062)
RXPE01000023.1 (genomic DNA)
ndh
Chloroflexi bacterium UBA5177
DHWR01000053.1_2 (1947833)
DHWR01000053.1 ()
yraN dctM ddpX ydhC ndh lldD bla yrpG yorfE nifS ybfI
Cobetia marina
AOM01579.1 (28258)
CP017114.1 (chromosome)
ndh resE3 rr09
Salinisphaeraceae bacterium
MAA74626.1 (2026789)
NYTH01000028.1 (genomic DNA)
ndh
Salinisphaera sp UBA1666
DCKU01000074.1_1 (1947454)
DCKU01000074.1 ()
hemF rdgB rph hrcA grpE folD ndh hcrA_1 cobO
Aureimonas sp Leaf460
KQT81318.1 (1736384)
LMQY01000001.1 (genomic DNA)
mcp mcp ndh cobQ cobH cobI
Aurantimonas manganoxydans SI85 9A1
EAS49115.1 (287752)
CH672387.1 (genomic)
dapB glnQ sunI hemF rdgB rph hrcA grpE folD ndh fixR2 cbiX cobI cobJ
Aurantimonas sp Leaf443
KQT83092.1 (1736378)
LMRD01000010.1 (genomic DNA)
rsmE gshA ndh pckA ybeB trmU gst1 ptsN raiA lptB
Fulvimarina manganoxydans
SMD12499.1 (937218)
FWXR01000032.1 (genomic DNA)
pqqB pqqB flhA flhA fliR ndh rpmJ purE purE
Fulvimarina pelagi HTCC2506
EAU41507.1 (314231)
DS022272.1 (genomic)
comM gshB rsmI hemN rdgB rph hrcA grpE ndh cobO cobW cbtA cbtB fixR2
Aureimonas altamirensis DSM 21988
SHJ72645.1 (1121026)
FQZC01000004.1 (genomic DNA)
rsmI hemN rdgB rph hrcA grpE folD ndh cobO cobW cbtA cbtB
Aureimonas phyllosphaerae
SFF23274.1 (1166078)
FOOA01000005.1 (genomic DNA)
hemF rph ndh
Aureimonas sp AU20
ALN71761.1 (1349819)
CP006367.1 (chromosome)
ndh
Aureimonas sp AU12
BBWH01000019.1_24 (1638161)
BBWH01000019.1 ()
gshB rsmI hemN rdgB rph hrcA grpE folD ndh cobO cobW cbtA cbtB phoE
Aureimonas jatrophae
SDN65871.1 (1166073)
FNIT01000001.1 (genomic DNA)
yrpG yorfE gltP ndh
Thiohalorhabdus denitrificans
KPV39257.1 (381306)
LJCP01000014.1 (genomic DNA)

Symbiobacterium thermophilum IAM 14863
BAD39948.1 (292459)
AP006840.1 (genomic DNA)

Kyrpidia tusciae DSM 2912
ADG05943.1 (562970)
CP002017.1 (chromosome)

Thermaerobacter marianensis DSM 12885
ADU52115.1 (644966)
CP002344.1 (chromosome)

Effusibacillus pohliae DSM 22757
AQXL01000126.1_71 (1120973)
AQXL01000126.1 ()

Effusibacillus lacus
GAX90675.1 (1348429)
BDUF01000061.1 (genomic DNA)

Tumebacillus permanentifrigoris
PWK08374.1 (378543)
QGGL01000015.1 (genomic DNA)

Tumebacillus algifaecis
ASS76120.1 (1214604)
CP022657.1 (chromosome)

Acidibacillus sulfuroxidans
PWI57183.1 (1765684)
MPDK01000017.1 (genomic DNA)

Alicyclobacillus montanus
SHJ51913.1 (1830138)
FRAF01000001.1 (genomic DNA)

Alicyclobacillus macrosporangiidus
SFU93935.1 (392015)
FPBV01000014.1 (genomic DNA)

Alicyclobacillus contaminans DSM 17975
AUCA01000020.1_43 (1120971)
AUCA01000020.1 ()

Alicyclobacillus ferrooxydans
KPV39001.1 (471514)
LJCO01000108.1 (genomic DNA)

Alicyclobacillus herbarius DSM 13609
AUMH01000001.1_243 (1120972)
AUMH01000001.1 ()

Alicyclobacillus acidoterrestris ATCC 49025
EPZ44320.1 (1356854)
AURB01000149.1 (genomic)

Alicyclobacillus acidocaldarius subsp acidocaldarius DSM 446
ACV59020.1 (521098)
CP001727.1 (chromosome)

Firmicutes bacterium HGW Firmicutes 8
PKM42455.1 (2013789)
PGZN01000045.1 (genomic DNA)

Firmicutes bacterium HGW Firmicutes 14
PKM82938.1 (2013775)
PHAB01000002.1 (genomic DNA)

Clostridiales bacterium PH28_bin88
KKM09863.1 (1605376)
LAKY01000058.1 (genomic DNA)

Kyrpidia tusciae DSM 2912
ADG05814.1 (562970)
CP002017.1 (chromosome)

Acidibacillus ferrooxidans
KUO95742.1 (1765683)
LPVJ01000037.1 (genomic DNA)

Acidibacillus sulfuroxidans
PWI58947.1 (1765684)
MPDK01000002.1 (genomic DNA)

Tumebacillus algifaecis
ASS76303.1 (1214604)
CP022657.1 (chromosome)

Tumebacillus permanentifrigoris
PWK10235.1 (378543)
QGGL01000012.1 (genomic DNA)

Alicyclobacillus macrosporangiidus
SFU38229.1 (392015)
FPBV01000001.1 (genomic DNA)

Firmicutes bacterium I2511
RIV21693.1 (2048547)
QXHL01000030.1 (genomic DNA)

Bacillus sp Marseille P3661
LT965956.1_65 (1936234)
LT965956.1 ()

Bacillus flexus
AQX54937.1 (86664)
CP016790.1 (chromosome)

Paenibacillus lactis
HAG01064.1 (228574)
DMBX01000049.1 (genomic DNA)

Paenibacillus beijingensis
AJY74650.1 (1126833)
CP011058.1 (chromosome)

Paenibacillaceae bacterium GAS479
SDS91577.1 (1882832)
LT629764.1 (genomic DNA)

Paenibacillus sp FJAT 26967

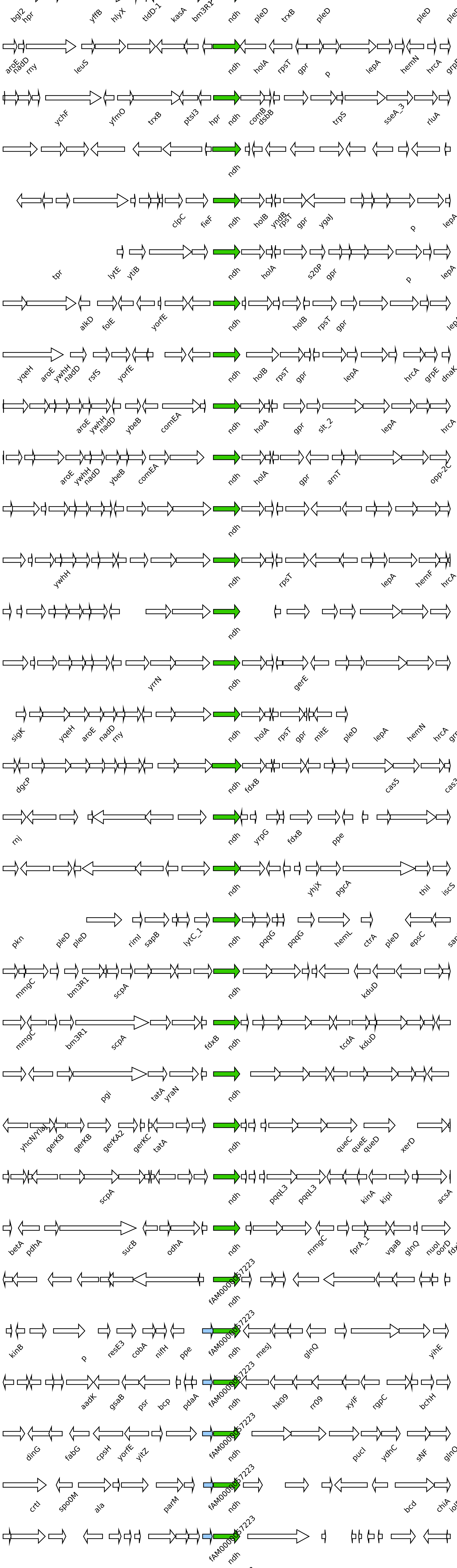

Paenibacillus sp FJAT 26967
LMXH01000004.1_210 (1729690)
LMXH01000004.1 ()

Paenibacillus pini JCM 16418
GAF07694.1 (1236976)
BAVZ01000004.1 (genomic DNA)

Cohnella laeviribosi DSM 21336
KB899872.1_2 (1121346)
KB899872.1 ()

Cohnella thermotolerans DSM 17683
AUCP01000006.1_97 (1121347)
AUCP01000006.1 ()

Paenibacillus curdlanolyticus YK9
EFM08619.1 (717606)
AEDD01000015.1 (genomic DNA)

Paenibacillus sp 453mf
SFS77859.1 (1761874)
FPAC01000003.1 (genomic DNA)

Paenibacillus polymyxa E681
ADM72419.1 (349520)
CP000154.2 (chromosome)

Tuberibacillus sp Marseille P3662
FXBS01000003.1_399 (1965358)
FXBS01000003.1 ()

Paenibacillus sp 32O W
ALS28245.1 (1695218)
CP013653.1 (chromosome)

Paenibacillus yonginensis
ANS76668.1 (1462996)
CP014167.1 (chromosome)

Bacillus coagulans
KGB28378.1 (1398)
JPSK01000137.1 (genomic DNA)

Paenibacillus xerothermodurans
PZE21055.1 (1977292)
NHRJ02000004.1 (genomic DNA)

Bacillus thermoamylovorans
CEE01011.1 (35841)
CCRF01000039.1 (genomic DNA)

Bacillus andreraoultii
CCFJ01000057.1_183 (1499685)
CCFJ01000057.1 ()

Bacillus circulans
KLV25747.1 (1397)
LDPH01000013.1 (genomic DNA)

Bacillaceae bacterium UBA4111
DFXJ01000095.1_8 (1951478)
DFXJ01000095.1 ()

Bacillus thuringiensis str Al Hakam
ABK87793.1 (412694)
CP000485.1 (chromosome)

Bacillus endophyticus
KYG28326.1 (135735)
LTAP01000034.1 (genomic DNA)

Bacillus subtilis subsp subtilis str 168
CAB13086.1 (224308)
AL009126.3 (genomic DNA)

Bacillus gobiensis
ALC80385.1 (1441095)
CP012600.1 (chromosome)

Bacillus sp OV322
SFD02631.1 (1882764)
FOMA01000015.1 (genomic DNA)

Bacillus acidicola
KV440953.1_1390 (209389)
KV440953.1 ()

Bacillus kribbensis DSM 17871
KE387242.1_267 (1121092)
KE387242.1 ()

Bacillus obstructivus
OJH19630.1 (1914540)
MPHG01000142.1 (genomic DNA)

Bacillus psychrosaccharolyticus ATCC 23296
AJTN02000176.1_7 (1174504)
AJTN02000176.1 ()

Bacillus loiseleuriae
KMY52421.1 (1679170)
LFZW01000001.1 (genomic DNA)

Paenibacillus sp IHBB 10380
AJS60586.1 (1566358)
CP010976.1 (chromosome)

Bacillus fastidiosus NBRC 101226
BCVG01000112.1_9 (1348628)
BCVG01000112.1 ()

Bacillus massilioanorexius AP8
CAPG01000090.1_2 (1211814)
CAPG01000090.1 ()

Bacillus butanolivorans
KON68581.1 (421767)
LGYA01000001.1 (genomic DNA)

Bacillus tuaregi
LT629731.1_2976 (1816695)
LT629731.1 ()

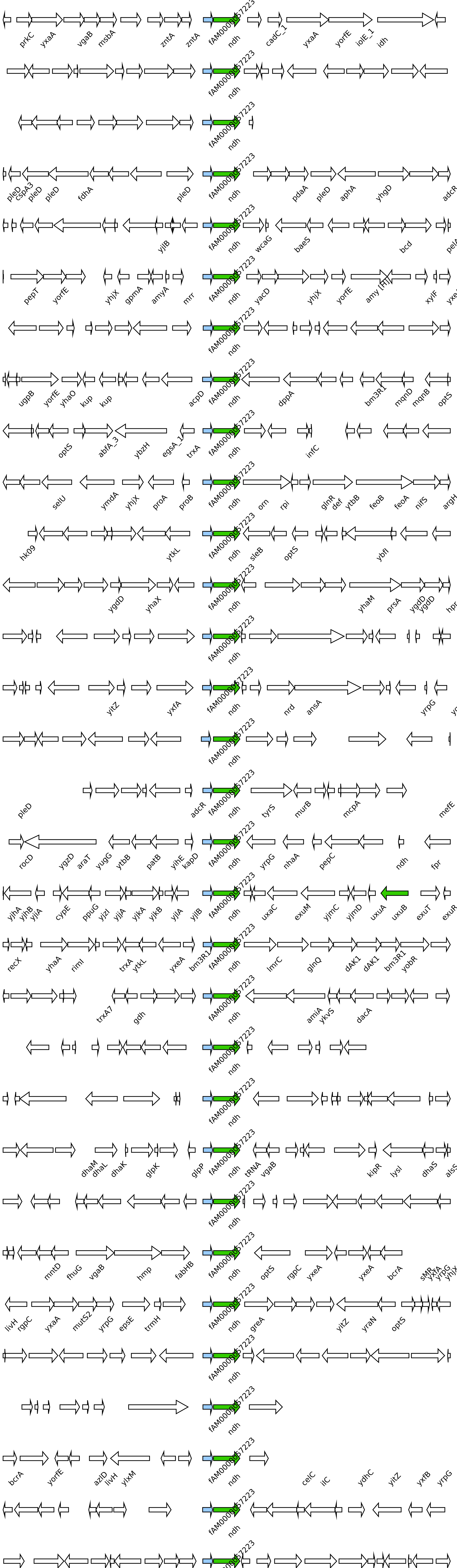

Bacillus testis
LN851186.1_3379 (1622072)
LN851186.1 ()

Terribacillus goriensis
AIF65294.1 (386490)
CP008876.1 (chromosome)

Ureibacillus thermosphaericus
AP018335.1_838 (51173)
AP018335.1 ()

Lysinibacillus sp Marseille P5727
LT985980.1_1930 (2086577)
LT985980.1 ()

Rummeliibacillus stabekisii
AMW98934.1 (241244)
CP014806.1 (chromosome)

Lysinibacillus sphaericus C3 41
ACA38389.1 (444177)
CP000817.1 (chromosome)

Caryophanon latum
OCS94245.1 (33977)
MATO01000003.1 (genomic DNA)

Solibacillus silvestris StLB046
BAK16415.1 (1002809)
AP012157.1 (genomic DNA)

Sporosarcina koreensis
LN651337.1_586 (334735)
LN651337.1 ()

Tuberibacillus calidus DSM 17572
AUMM01000029.1_37 (1123405)
AUMM01000029.1 ()

Anaerobacillus macyae
KMM39168.1 (157733)
LELK01000001.1 (genomic DNA)

Kurthia massiliensis
HE610997.1_491 (1033739)
HE610997.1 ()

Kurthia sp 11kri321
AMA64515.1 (1750719)
CP013217.1 (chromosome)

Kurthia sibirica
PWI26101.1 (202750)
QFVR01000004.1 (genomic DNA)

Carboxydocella thermautotrophica
AVX20097.1 (178899)
CP028491.1 (chromosome)

Desulfuribacillus alkaliarsenatis
OEF96681.1 (766136)
MIJE01000030.1 (genomic DNA)

Desulfuribacillus stibiiarsenatis
OEH85335.1 (1390249)
MJAT01000022.1 (genomic DNA)

Firmicutes bacterium UBA5301
DHRX01000039.1_25 (1948048)
DHRX01000039.1 ()

Bacillus massilioanorexius AP8
CAPG01000027.1_456 (1211814)
CAPG01000027.1 ()

Bacillus campisalis
KKK34421.1 (1408103)
LAYY01000097.1 (genomic DNA)

Bacillus bacterium
HAQ08877.1 (2053512)
DMSR01000553.1 (genomic DNA)

Bacillus jeotgali
CP025025.1_1212 (129985)
CP025025.1 ()

Bacillus sp FJAT 18017
ALC90824.1 (1705566)
CP012602.1 (chromosome)

Bacillus rubiinfantis
CCFE01000023.1_200 (1499680)
CCFE01000023.1 ()

Bacillus sp AFS006103
PEQ94073.1 (2033483)
NTXX01000011.1 (genomic DNA)

Anaerobacillus arseniciselenatis
OIJ12991.1 (85682)
MLQQ01000018.1 (genomic DNA)

Bacillus sp 1NLA3E
AGK53289.1 (666686)
CP005586.1 (chromosome)

Bacillus sp Marseille P3661
LT965955.1_70 (1936234)
LT965955.1 ()

Gemella asaccharolytica
KXB58538.1 (502393)
KQ959861.1 (genomic)

Gemella cuniculi DSM 15828
KE384432.1_32 (1121914)
KE384432.1 ()

Gemella sanguinis ATCC 700632

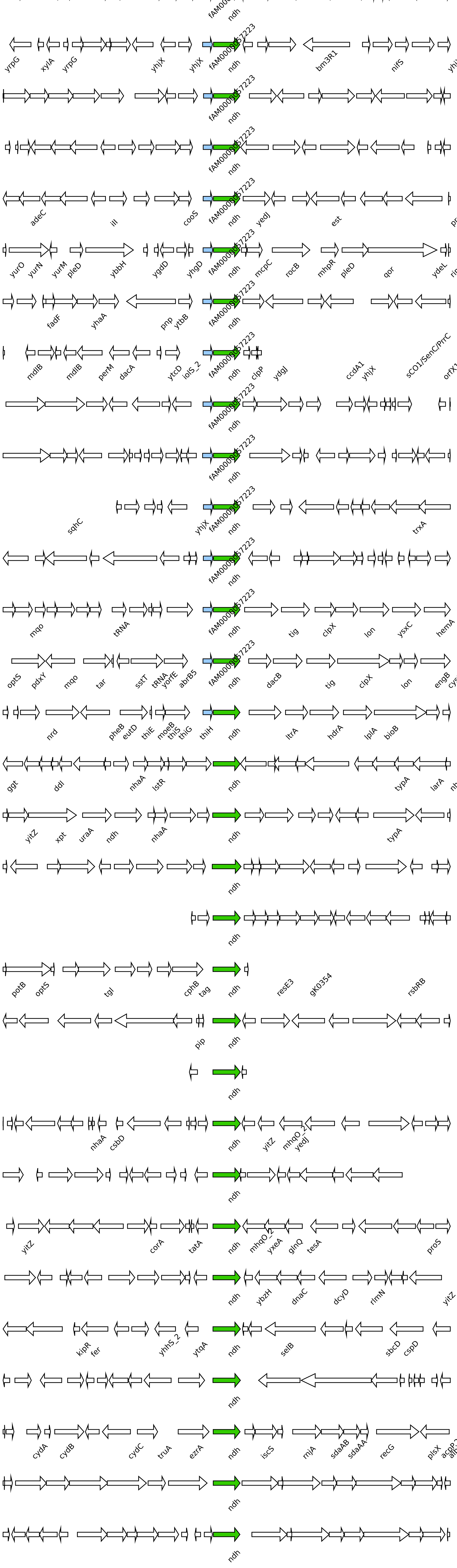

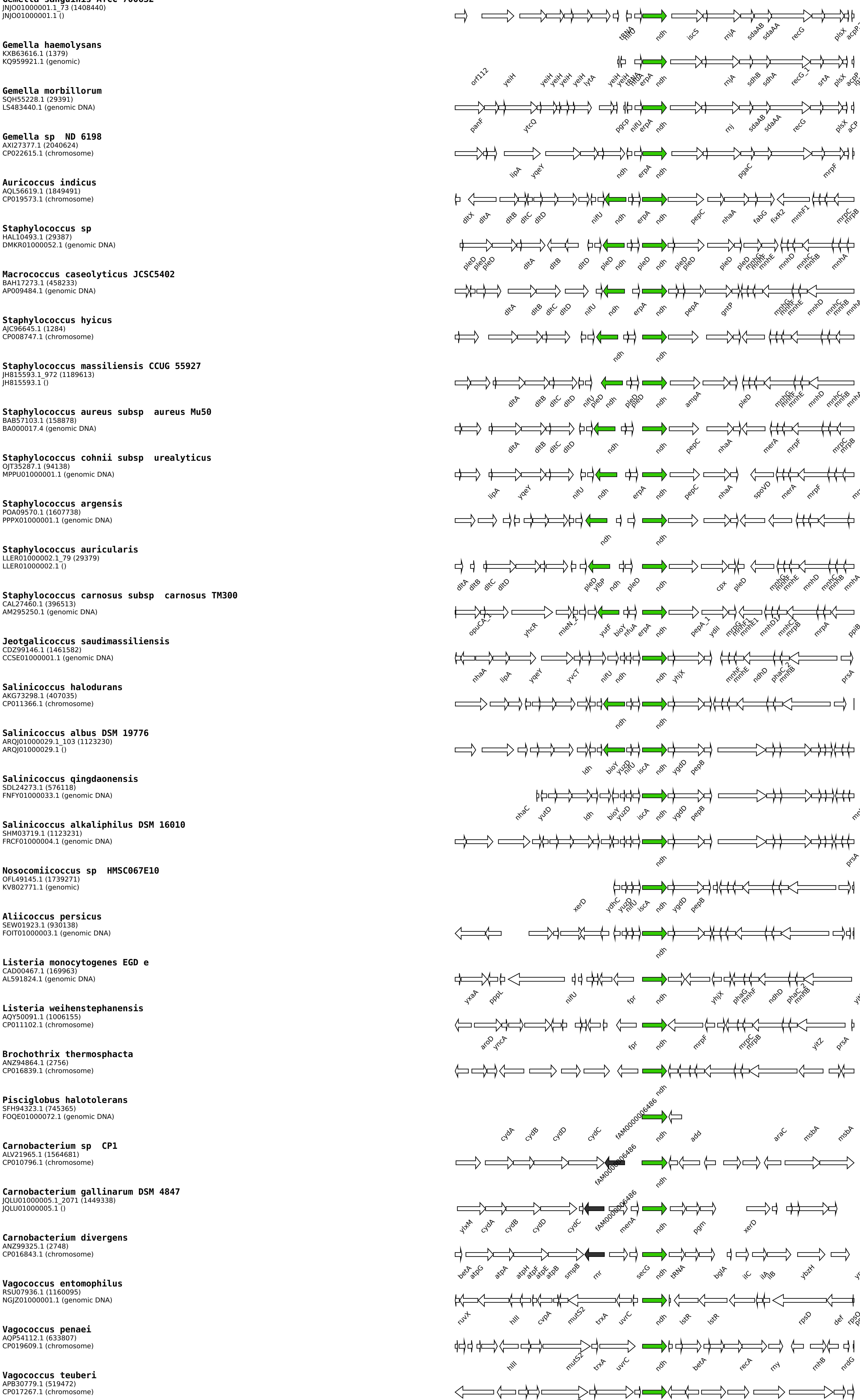
JNJO01000001.1_73 (1408440)
JNJO01000001.1 ()
Gemella haemolysans
KXB63616.1 (1379)
KQ959921.1 (genomic)
Gemella morbillorum
SQH55228.1 (29391)
LS483440.1 (genomic DNA)
Gemella sp ND 6198
AXI27377.1 (2040624)
CP022615.1 (chromosome)
Auricoccus indicus
AQL56619.1 (1849491)
CP019573.1 (chromosome)
Staphylococcus sp
HAL10493.1 (29387)
DMKR01000052.1 (genomic DNA)
Macrococcus caseolyticus JCSC5402
BAH17273.1 (458233)
AP009484.1 (genomic DNA)
Staphylococcus hyicus
AJC96645.1 (1284)
CP008747.1 (chromosome)
Staphylococcus massiliensis CCUG 55927
JH815593.1_972 (1189613)
JH815593.1 ()
Staphylococcus aureus subsp aureus Mu50
BAB57103.1 (158878)
BA000017.4 (genomic DNA)
Staphylococcus cohnii subsp urealyticus
OJT35287.1 (94138)
MPPU01000001.1 (genomic DNA)
Staphylococcus argensis
POA09570.1 (1607738)
PPPX01000001.1 (genomic DNA)
Staphylococcus auricularis
LLER01000002.1_79 (29379)
LLER01000002.1 ()
Staphylococcus carnosus subsp carnosus TM300
CAL27460.1 (396513)
AM295250.1 (genomic DNA)
Jeotgalicoccus saudimassiliensis
CDZ99146.1 (1461582)
CCSE01000001.1 (genomic DNA)
Salinicoccus halodurans
AKG73298.1 (407035)
CP011366.1 (chromosome)
Salinicoccus albus DSM 19776
ARQJ01000029.1_103 (1123230)
ARQJ01000029.1 ()
Salinicoccus qingdaonensis
SDL24273.1 (576118)
FNFY01000033.1 (genomic DNA)
Salinicoccus alkaliphilus DSM 16010
SHM03719.1 (1123231)
FRCF01000004.1 (genomic DNA)
Nosocomiicoccus sp HMSC067E10
OFL49145.1 (1739271)
KV802771.1 (genomic)
Aliicoccus persicus
SEW01923.1 (930138)
FOIT01000003.1 (genomic DNA)
Listeria monocytogenes EGD e
CAD00467.1 (169963)
AL591824.1 (genomic DNA)
Listeria weihenstephanensis
AQY50091.1 (1006155)
CP011102.1 (chromosome)
Brochothrix thermosphacta
ANZ94864.1 (2756)
CP016839.1 (chromosome)
Pisciglobus halotolerans
SFH94323.1 (745365)
FOQE01000072.1 (genomic DNA)
Carnobacterium sp CP1
ALV21965.1 (1564681)
CP010796.1 (chromosome)
Carnobacterium gallinarum DSM 4847
JQLU01000005.1_2071 (1449338)
JQLU01000005.1 ()
Carnobacterium divergens
ANZ99325.1 (2748)
CP016843.1 (chromosome)
Vagococcus entomophilus
RSU07936.1 (1160095)
NGJZ01000001.1 (genomic DNA)
Vagococcus penaei
AQP54112.1 (633807)
CP019609.1 (chromosome)
Vagococcus teuberi
APB30779.1 (519472)
CP017267.1 (chromosome)

Vagococcus fluvialis
RCX15222.1 (2738)
QPJV01000002.1 (genomic DNA)

Vagococcus humatus
RST90275.1 (1889241)
PXZH01000001.1 (genomic DNA)

Vagococcus salmoninarum
RST94308.1 (2739)
NGJU01000015.1 (genomic DNA)

Vagococcus lutrae LBD1
EST89832.1 (1408226)
AYSH01000015.1 (genomic DNA)

Sporolactobacillus pectinivorans
NXGA01000001.1_642 (1591408)
NXGA01000001.1 ()

Sporolactobacillus nakayamae
SFG28425.1 (269670)
FOOY01000007.1 (genomic DNA)

Lactobacillus salivarius UCC118
ABD99834.1 (362948)
CP000233.1 (chromosome)

Lactobacillus agilis DSM 20509
KRM65638.1 (1423718)
AYYP01000012.1 (genomic)

Streptococcus agalactiae NEM316
CAD47447.1 (211110)
AL732656.1 (genomic DNA)

Streptococcus urinalis
VEF33021.1 (149016)
LR134323.1 (genomic DNA)

Eremococcus coleocola DSM 15696
AUAT01000010.1_7 (1121871)
AUAT01000010.1 ()

Enterococcus faecalis V583
AAO81788.1 (226185)
AE016830.1 (chromosome)

Enterococcus phoeniculicola
OJG73152.1 (154621)
JXKY01000003.1 (genomic)

Enterococcus gallinarum EG2
EEV34253.1 (565653)
GG670291.1 (genomic DNA)

Lactobacillus satsumensis DSM 16230 JCM 12392
BBCQ01000027.1_37 (1423801)
BBCQ01000027.1 ()

Lactobacillus hordei DSM 19519
KRL06785.1 (1423759)
AZDX01000014.1 (genomic)

Lactobacillus cacaonum DSM 21116
KRM92751.1 (1423729)
AYZE01000005.1 (genomic)

Lactobacillus ghanensis DSM 18630
KRM05806.1 (1423750)
AZGB01000018.1 (genomic)

Lactobacillus vini DSM 20605
AHYZ01000079.1_4 (1133569)
AHYZ01000079.1 ()

Lactobacillus aquaticus DSM 21051
KRM96136.1 (1423725)
AYZD01000017.1 (genomic)

Aerococcus sp 1KP 2016
OYQ66882.1 (1981982)
NEEY01000019.1 (genomic DNA)

Lactobacillus acidipiscis
KRN84401.1 (89059)
JQBK01000032.1 (genomic)

Pediococcus claussenii ATCC BAA 344
AEV94634.1 (701521)
CP003137.1 (chromosome)

Enterococcus canis
OJG18390.1 (214095)
JXKH01000004.1 (genomic)

Enterococcus avium
JUZB01000322.1_38 (33945)
JUZB01000322.1 ()

Enterococcus aquimarinus
OJG09781.1 (328396)
JXKD01000013.1 (genomic)

Enterococcus italicus DSM 15952
EFU74234.1 (888064)
GL622241.1 (genomic)

Enterococcus saccharolyticus subsp saccharolyticus ATCC 43076
EOT25953.1 (1139996)
KE136391.1 (genomic)

Tetragenococcus osmophilus
AYW47763.1 (526944)
CP027783.1 (chromosome)

Tetragenococcus halophilus
LSFG01000026.1_21 (51669)
LSFG01000026.1 ()

Enterococcus avium

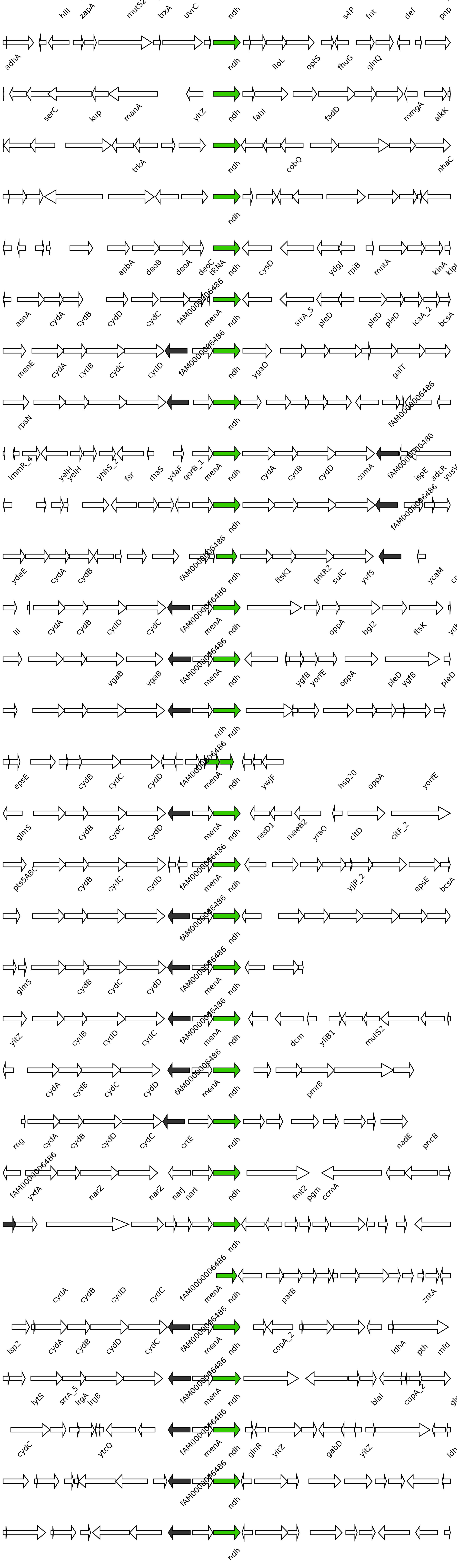

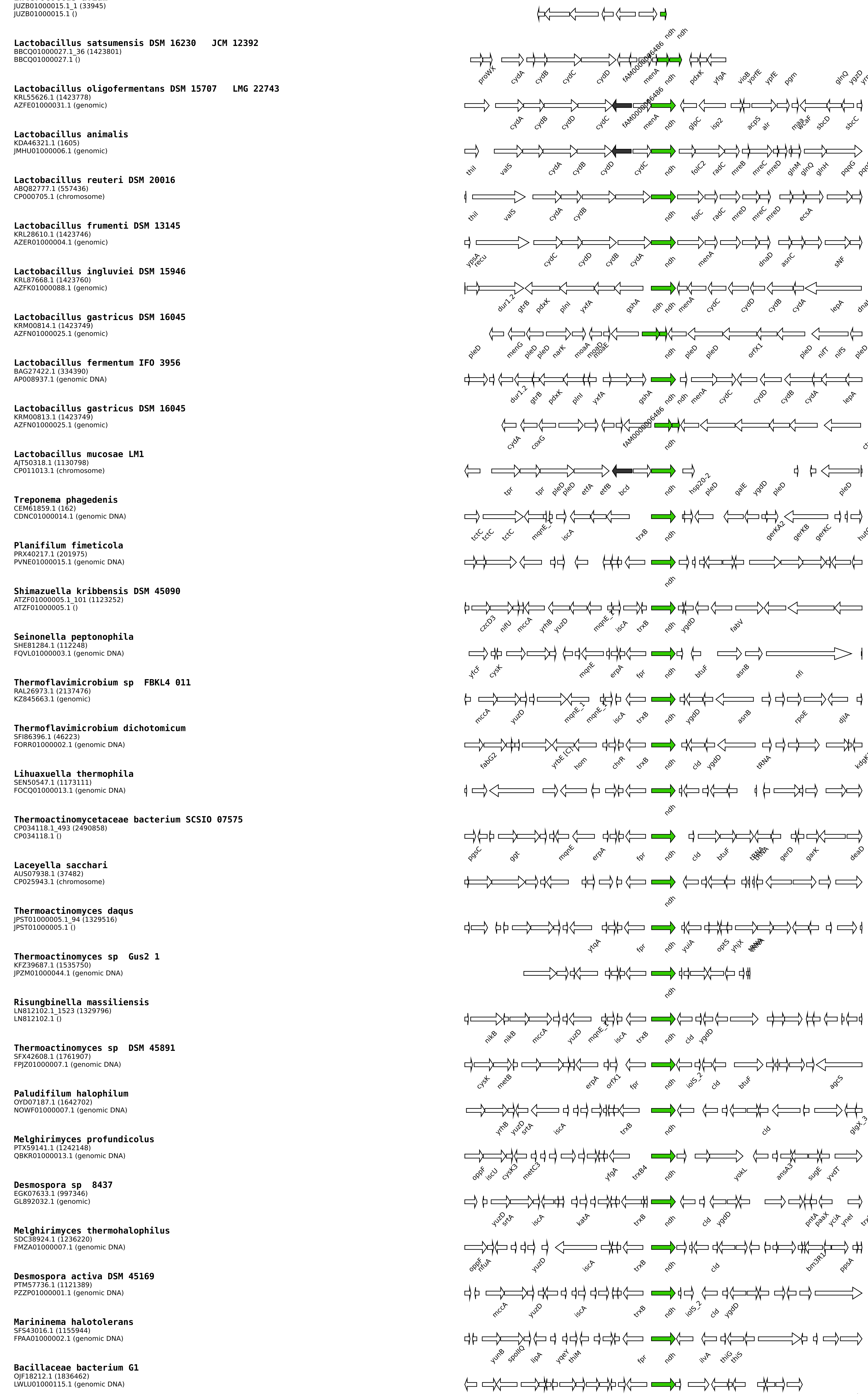

JUZB01000015.1_1 (33945)
JUZB01000015.1 ()
Lactobacillus satsumensis DSM 16230 JCM 12392
BBCQ01000027.1_36 (1423801)
BBCQ01000027.1 ()
Lactobacillus oligofermentans DSM 15707 LMG 22743
KRL55626.1 (1423778)
AZFE01000031.1 (genomic)
Lactobacillus animalis
KDA46321.1 (1605)
JMHU01000006.1 (genomic)
Lactobacillus reuteri DSM 20016
ABQ82777.1 (557436)
CP000705.1 (chromosome)
Lactobacillus frumenti DSM 13145
KRL28610.1 (1423746)
AZER01000004.1 (genomic)
Lactobacillus ingluviei DSM 15946
KRL87668.1 (1423760)
AZFK01000088.1 (genomic)
Lactobacillus gastricus DSM 16045
KRM00814.1 (1423749)
AZFN01000025.1 (genomic)
Lactobacillus fermentum IFO 3956
BAG27422.1 (334390)
AP008937.1 (genomic DNA)
Lactobacillus gastricus DSM 16045
KRM00813.1 (1423749)
AZFN01000025.1 (genomic)
Lactobacillus mucosae LM1
AJT50318.1 (1130798)
CP011013.1 (chromosome)
Treponema phagedenis
CEM61859.1 (162)
CDNC01000014.1 (genomic DNA)
Planifilum fimeticola
PRX40217.1 (201975)
PVNE01000015.1 (genomic DNA)
Shimazuella kribbensis DSM 45090
ATZF01000005.1_101 (1123252)
ATZF01000005.1 ()
Seinonella peptonophila
SHE81284.1 (112248)
FQVL01000003.1 (genomic DNA)
Thermoflavimicrobium sp FBKL4 011
RAL26973.1 (2137476)
KZ845663.1 (genomic)
Thermoflavimicrobium dichotomicum
SFI86396.1 (46223)
FORR01000002.1 (genomic DNA)
Lihuaxuella thermophila
SEN50547.1 (1173111)
FOCQ01000013.1 (genomic DNA)
Thermoactinomycetaceae bacterium SCSIO 07575
CP034118.1_493 (2490858)
CP034118.1 ()
Laceyella sacchari
AUS07938.1 (37482)
CP025943.1 (chromosome)
Thermoactinomyces daqus
JPST01000005.1_94 (1329516)
JPST01000005.1 ()
Thermoactinomyces sp Gus2 1
KFZ39687.1 (1535750)
JPZM01000044.1 (genomic DNA)
Risungbinella massiliensis
LN812102.1_1523 (1329796)
LN812102.1 ()
Thermoactinomyces sp DSM 45891
SFX42608.1 (1761907)
FPJZ01000007.1 (genomic DNA)
Paludifilum halophilum
OYD07187.1 (1642702)
NOWF01000007.1 (genomic DNA)
Melghirimyces profundicolus
PTX59141.1 (1242148)
QBKR01000013.1 (genomic DNA)
Desmospora sp 8437
EGK07633.1 (997346)
GL892032.1 (genomic)
Melghirimyces thermohalophilus
SDC38924.1 (1236220)
FMZA01000007.1 (genomic DNA)
Desmospora activa DSM 45169
PTM57736.1 (1121389)
PZZP01000001.1 (genomic DNA)
Marininema halotolerans
SFS43016.1 (1155944)
FPAA01000002.1 (genomic DNA)
Bacillaceae bacterium G1
OJF18212.1 (1836462)
LWLU01000115.1 (genomic DNA)

Bacillus thermozeamaize
OUM86488.1 (230954)
LZRT01000090.1 (genomic DNA)

Caldalkalibacillus thermarum TA2 A1
EGL84165.1 (986075)
AFCE01000037.1 (genomic DNA)

Bacillus mannanilyticus JCM 10596
BAMO01000030.1_4 (1236954)
BAMO01000030.1 ()

Calditerricola satsumensis JCM 14719
BBCF01000123.1_7 (1294024)
BBCF01000123.1 ()

Brevibacillus laterosporus
JNFS01000001.1_690 (1465)
JNFS01000001.1 ()

Brevibacillus thermoruber 423
KE695649.1_320 (1346613)
KE695649.1 ()

Brevibacillus sp SCSIO 07484
CP034541.1_3047 (2496837)
CP034541.1 ()

Aneurinibacillus terranovensis DSM 18919
AUMJ01000009.1_77 (1121002)
AUMJ01000009.1 ()

Aneurinibacillus thermoaerophilus
SDH37704.1 (143495)
FNDE01000022.1 (genomic DNA)

Aneurinibacillus soli
BAU26889.1 (1500254)
AP017312.1 (genomic DNA)

Ammoniphilus oxalaticus
RKD21715.1 (66863)
MCHY01000011.1 (genomic DNA)

Paenibacillaceae bacterium ZCTH02 B3
OUN00782.1 (1861638)
LZRV01000037.1 (genomic DNA)

Cohnella sp 18JY8 7
AYQ71375.1 (2480923)
CP033433.1 (chromosome)

Cohnella panacarvi Gsoil 349
KI911563.1_3757 (979226)
KI911563.1 ()

Cohnella thermotolerans DSM 17683
AUCP01000046.1_6 (1121347)
AUCP01000046.1 ()

Cohnella laeviribosi DSM 21336
KB899819.1_43 (1121346)
KB899819.1 ()

Paenibacillus sp 32O W
ALS26773.1 (1695218)
CP013653.1 (chromosome)

Paenibacillus darwinianus
EXX89222.1 (1380763)
KK082133.1 (genomic)

Paenibacillus beijingensis
AJY77785.1 (1126833)
CP011058.1 (chromosome)

Paenibacillaceae bacterium GAS479
SDT20601.1 (1882832)
LT629764.1 (genomic DNA)

Thermobacillus composti KWC4
AGA58833.1 (717605)
CP003255.1 (chromosome)

Paenibacillus curdlanolyticus YK9
EFM08744.1 (717606)
AEDD01000014.1 (genomic DNA)

Paenibacillus swuensis
ANE45034.1 (1178515)
CP011388.1 (chromosome)

Paenibacillus sp FJAT 26967
LMXH01000001.1_697 (1729690)
LMXH01000001.1 ()

Paenibacillus xerothermodurans
PZE21312.1 (1977292)
NHRJ02000003.1 (genomic DNA)

Paenibacillus lentus
AZK46272.1 (1338368)
CP034248.1 (chromosome)

Paenibacillus lactis
HAF97137.1 (228574)
DMBX01000004.1 (genomic DNA)

Paenibacillus sp IHBB 10380
AJS57419.1 (1566358)
CP010976.1 (chromosome)

Paenibacillus pini JCM 16418
GAF08621.1 (1236976)
BAVZ01000007.1 (genomic DNA)

Paenibacillus durus
AIQ14278.1 (44251)
CP009288.1 (chromosome)

Paenibacillus yonginensis

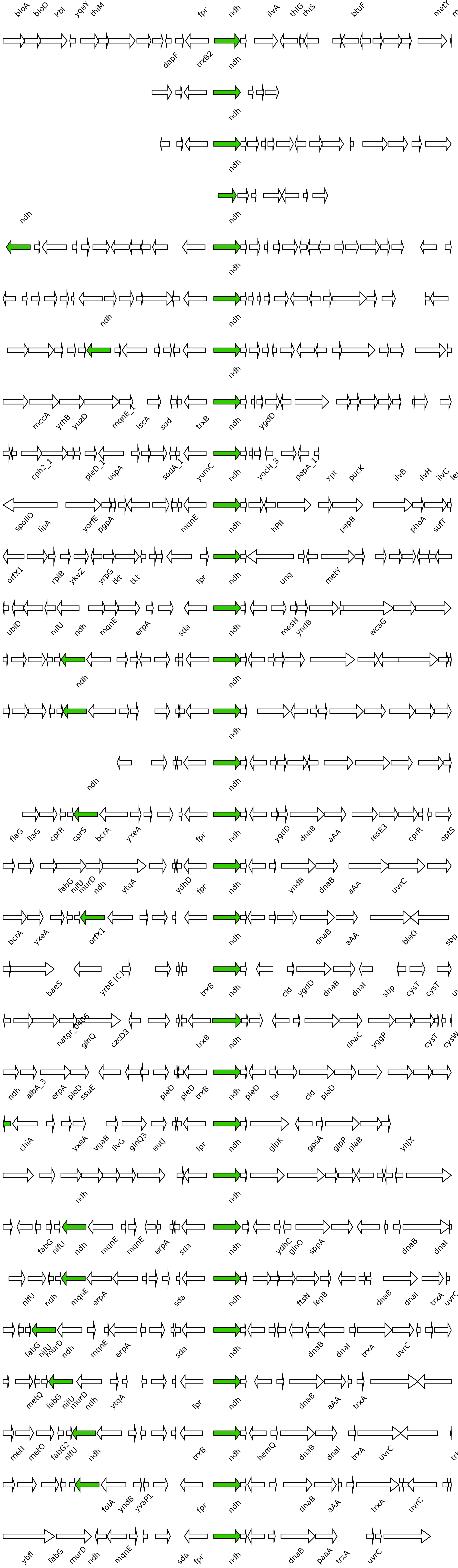

ANS75627.1 (1462996)
CP014167.1 (chromosome)

Gorillibacterium timonense
LN881723.1_851 (1689269)
LN881723.1 ()

Paenibacillus assamensis DSM 18201
AULU01000010.1_98 (1122916)
AULU01000010.1 ()

Paenibacillus popilliae ATCC 14706
GAC41731.1 (1212764)
BALG01000047.1 (genomic DNA)

Paenibacillus sp 453mf
SFS91764.1 (1761874)
FPAC01000007.1 (genomic DNA)

Paenibacillus polymyxa E681
ADM71680.1 (349520)
CP000154.2 (chromosome)

Paenibacillus bovis
ANF98174.1 (1616788)
CP013023.1 (chromosome)

Paenibacillaceae bacterium JTherm
PDO09823.1 (1906605)
MOXJ01000026.1 (genomic DNA)

Paenibacillus larvae subsp larvae B 3650
PCK69565.1 (741161)
ADZY03000325.1 (genomic DNA)

Bacillales bacterium UBA4045
DFZX01000002.1_42 (1950274)
DFZX01000002.1 ()

Saccharibacillus kuerlensis DSM 22868
KB899305.1_5 (1123226)
KB899305.1 ()

Tepidibacillus sp HK 1
GAU64159.1 (1883407)
BDGM01000073.1 (genomic DNA)

Novibacillus thermophilus
AQS55918.1 (1471761)
CP019699.1 (chromosome)

Numidum massiliense
CTDZ01000009.1_3293 (1522315)
CTDZ01000009.1 ()

Thermicanus aegyptius DSM 12793
KI783301.1_2654 (1089548)
KI783301.1 ()

Candidatus Carbobacillus altaicus
PTQ57627.1 (2163959)
PEBX01000004.1 (genomic DNA)

Hydrogenibacillus schlegelii
KWX07873.1 (1484)
JYFD01000117.1 (genomic DNA)

Rubeoparvulum massiliense
CVPE01000005.1_41 (1631346)
CVPE01000005.1 ()

Lihuaxuella thermophila
SEN34043.1 (1173111)
FOCQ01000009.1 (genomic DNA)

Bacillus tuaregi
LT629731.1_727 (1816695)
LT629731.1 ()

Bacillus oleivorans
SNX75254.1 (1448271)
OAOP01000011.1 (genomic DNA)

Bacillus salsus
SDP92925.1 (930152)
FNJU01000013.1 (genomic DNA)

Geobacillus sp 8
KZM55277.1 (1817674)
LVHY01000098.1 (genomic DNA)

Bacillus sp FJAT 42376
AZB44960.1 (2014076)
CP033906.1 (chromosome)

Bacillus indicus
KEZ47852.1 (246786)
JNVC02000015.1 (genomic DNA)

Bacillus weihaiensis
APH03826.1 (1547283)
CP016020.1 (chromosome)

Bacillus fastidiosus NBRC 101226
BCVG01000066.1_21 (1348628)
BCVG01000066.1 ()

Anoxybacillus flavithermus WK1
ACJ34759.1 (491915)
CP000922.1 (chromosome)

Anoxybacillus amylolyticus
ANB59822.1 (294699)
CP015438.1 (chromosome)

Geobacillus kaustophilus HTA426
BAD77238.1 (235909)
BA000043.1 (genomic DNA)

Geobacillus sp WCH70
ACS25564.1 (471223)
CP001638.1 (chromosome)

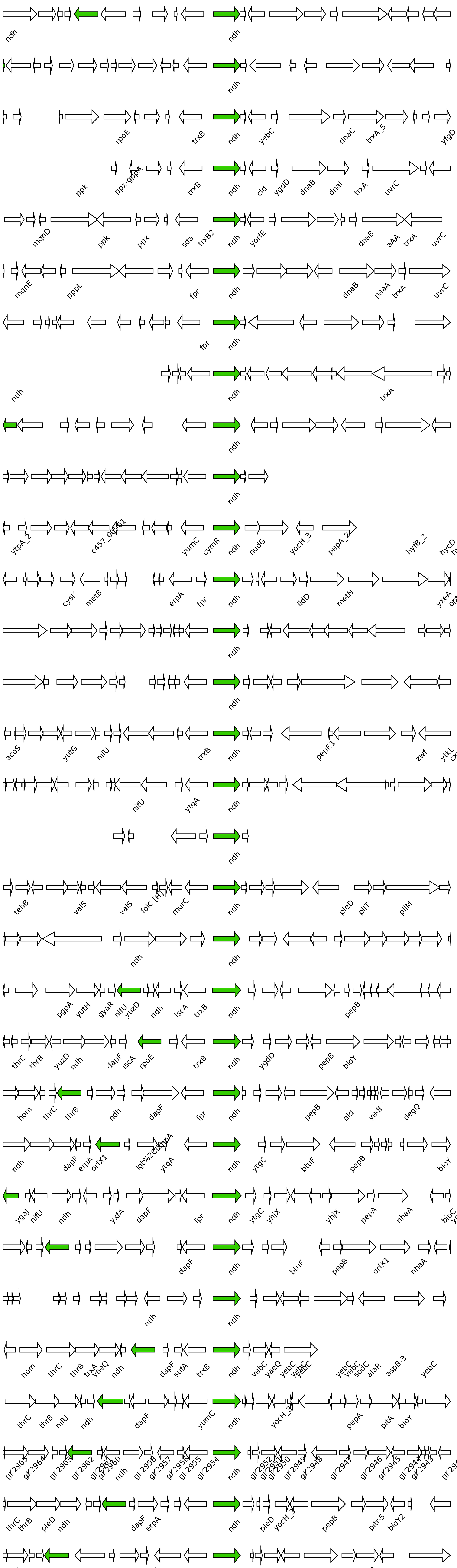

Bacillus cohnii
AST93126.1 (33932)
CP018866.1 (chromosome)

Bacillus sp FJAT 45066
NISQ01000001.1_3808 (2011010)
NISQ01000001.1 ()

Bacillus halmapalus
KV917375.1_858 (79882)
KV917375.1 ()

Bacillus kribbensis DSM 17871
KE387239.1_358 (1121092)
KE387239.1 ()

Bacillus sp V47 23a
RFU70138.1 (2303992)
QVTE01000017.1 (genomic DNA)

Bacillus acanthi
QBBX01000008.1_109 (2171554)
QBBX01000008.1 ()

Bacillus sp V3 13
PLR79354.1 (2053728)
PGUZ01000002.1 (genomic DNA)

Bacillus firmus
KML38794.1 (1399)
LDWQ01000052.1 (genomic DNA)

Bacillus bacterium
HAQ06333.1 (2053512)
DMSR01000064.1 (genomic DNA)

Bacillus jeotgali
CP025025.1_3851 (129985)
CP025025.1 ()

Bacillus campisalis
KKK39330.1 (1408103)
LAYY01000003.1 (genomic DNA)

Bacillus sp UNCCL13
SFB12827.1 (1502772)
FOJZ01000006.1 (genomic DNA)

Bacillus cavernae
RUQ25927.1 (1674310)
RYZZ01000037.1 (genomic DNA)

Bacillus sp AFS006103
PEQ84101.1 (2033483)
NTXX01000081.1 (genomic DNA)

Bacillus sp X1 2014
AIM16061.1 (1565991)
CP008855.1 (chromosome)

Bacillus rubiinfantis
CCFE01000011.1_112 (1499680)
CCFE01000011.1 ()

Bacillus methanolicus MGA3
EIJ77929.1 (796606)
ADWW01000005.1 (genomic DNA)

Bacillus sp Y1
AYA77699.1 (352858)
CP030028.1 (chromosome)

Bacillus massilionigeriensis
LT630337.1_3326 (1805475)
LT630337.1 ()

Bacillaceae bacterium UBA7792
DLCS01000169.1_1 (1951479)
DLCS01000169.1 ()

Bacillus sp 1NLA3E
AGK55465.1 (666686)
CP005586.1 (chromosome)

Falsibacillus pallidus
RDI41474.1 (493781)
QQAY01000008.1 (genomic DNA)

Domibacillus aminovorans
OAH61567.1 (29332)
LQWY01000017.1 (genomic DNA)

Bacillus badius
KIL79498.1 (1455)
JXLP01000003.1 (genomic)

Bacillus sp FJAT 27231
KMY55172.1 (1679168)
LFZU01000001.1 (genomic DNA)

Bacillus sp FJAT 18017
ALC89492.1 (1705566)
CP012602.1 (chromosome)

Bacillus butanolivorans
KON67108.1 (421767)
LGYA01000005.1 (genomic DNA)

Bacillus sp OV322
SFC29361.1 (1882764)
FOMA01000002.1 (genomic DNA)

Bacillus enclensis
KSU59416.1 (1402860)
KQ758487.1 (genomic)

Bacillus smithii
AKP48279.1 (1479)
CP012024.1 (chromosome)

Bacillus coahuilensis m2 6

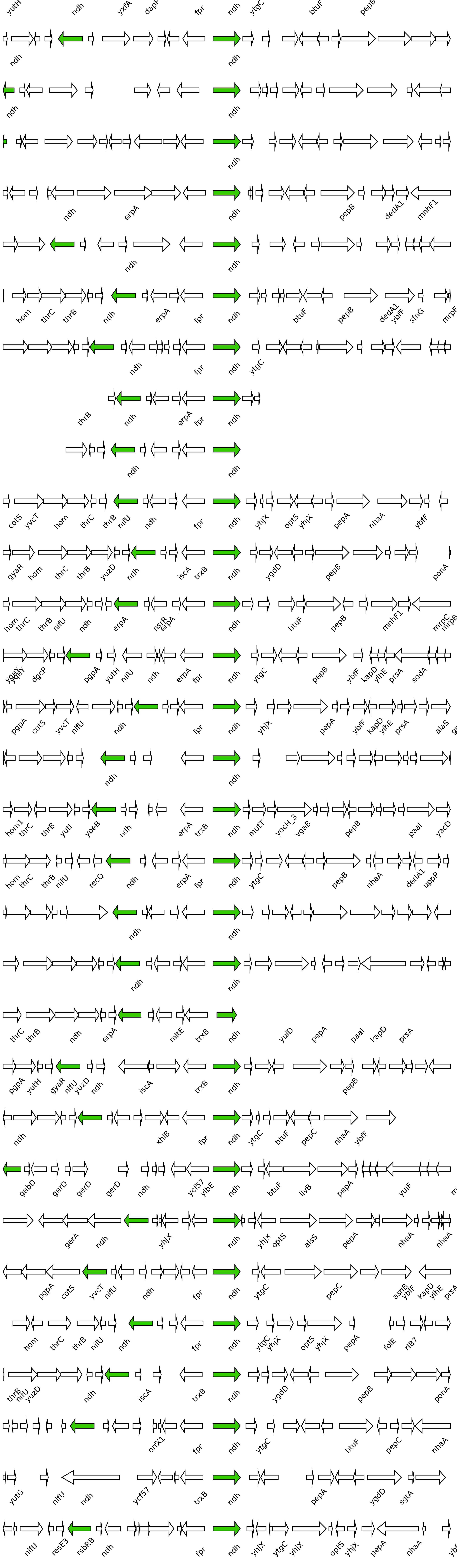

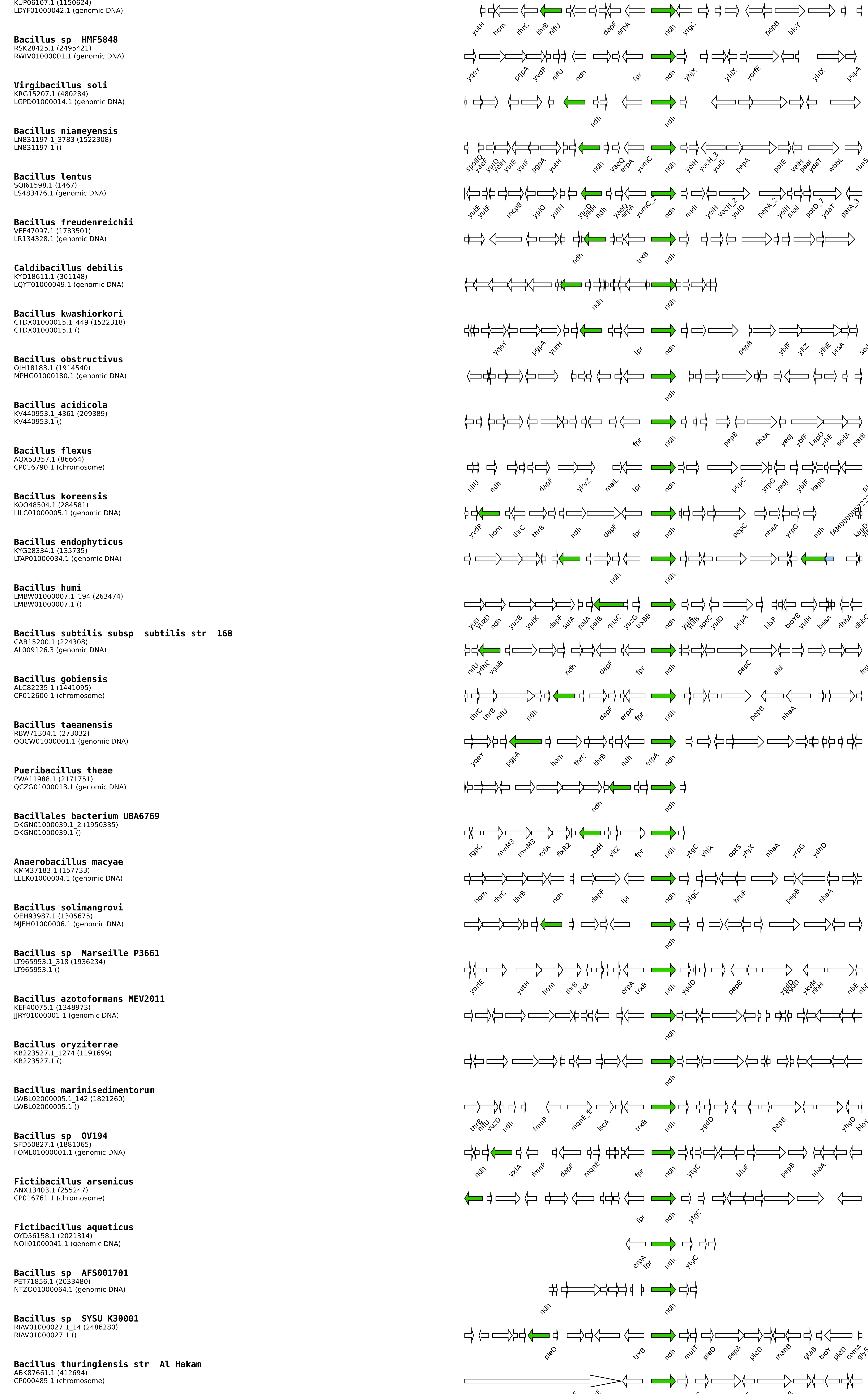
KUP06107.1 (1150624)
LDYF01000042.1 (genomic DNA)
yutH hom thrC thrB nifU dapF erpA ndh ytgC pepB bioY
Bacillus sp HMF5848
RSK28425.1 (2495421)
RWIV01000001.1 (genomic DNA)
yqeY pgpA yvdP nifU ndh fpr ndh yhjX yhjX yorfE yhjX pepA
Virgibacillus soli
KRG15207.1 (480284)
LGPD01000014.1 (genomic DNA)
ndh ndh
Bacillus niameyensis
LN831197.1_3783 (1522308)
LN831197.1 ()
spoIIQ yaeF yutD yeiH yutE yutF pgpA yutH ndh yaeQ erpA yumC ndh yeiH yocH_3 yuiD pepA potE yeiH paaI ydaT wbbL sunS
Bacillus lentus
SQI61598.1 (1467)
LS483476.1 (genomic DNA)
yutE yutF mcpB ypjQ yutH yuzD yeiH ndh yaeQ erpA yumC_2 ndh nudI yeiH yocH_2 yuiD pepA_2 yeiH paaI potD_7 ydaT gatA_3
Bacillus freudenreichii
VEF47097.1 (1783501)
LR134328.1 (genomic DNA)
ndh trxB ndh
Caldibacillus debilis
KYD18611.1 (301148)
LQYT01000049.1 (genomic DNA)
ndh ndh
Bacillus kwashiorkori
CTDX01000015.1_449 (1522318)
CTDX01000015.1 ()
yqeY pgpA yutH fpr ndh pepB ybfF yitZ yihE prsA
Bacillus obstructivus
OJH18183.1 (1914540)
MPHG01000180.1 (genomic DNA)
ndh
Bacillus acidicola
KV440953.1_4361 (209389)
KV440953.1 ()
fpr ndh pepB nhaA yedJ ybfF kapD yihE sodA patB
Bacillus flexus
AQX53357.1 (86664)
CP016790.1 (chromosome)
nifU ndh dapF ykvZ malL fpr ndh pepC yrpG yedJ ybfF kapD
Bacillus koreensis
KOO48504.1 (284581)
LILC01000005.1 (genomic DNA)
yvdP hom thrC thrB ndh dapF fpr ndh pepC nhaA yrpG ndh
Bacillus endophyticus
KYG28334.1 (135735)
LTAP01000034.1 (genomic DNA)
ndh ndh
Bacillus humi
LMBW01000007.1_194 (263474)
LMBW01000007.1 ()
yutI yuzD ndh yuzB yutK dapF sufA paiA paiB guaC yuzG trxBB ndh yutJA yutE spsC yuiD pepA hisP bioYB yuiH besA dhbA dhbC
Bacillus subtilis subsp subtilis str 168
CAB15200.1 (224308)
AL009126.3 (genomic DNA)
nifU ydhC vgaB ndh dapF fpr ndh pepC ald
Bacillus gobiensis
ALC82235.1 (1441095)
CP012600.1 (chromosome)
thrC thrB nifU ndh dapF erpA fpr ndh pepB nhaA
Bacillus taeanensis
RBW71304.1 (273032)
QOCW01000001.1 (genomic DNA)
yqeY pgpA hom thrC thrB ndh erpA ndh
Pueribacillus theae
PWA11988.1 (2171751)
QCZG01000013.1 (genomic DNA)
ndh ndh
Bacillales bacterium UBA6769
DKGN01000039.1_2 (1950335)
DKGN01000039.1 ()
rgpC mviM3 mviM3 xylA fixR2 ybzH yitZ fpr ndh ytgC yhjX optS yhjX nhaA yrpG ydhD
Anaerobacillus macyae
KMM37183.1 (157733)
LELK01000004.1 (genomic DNA)
hom thrC thrB ndh dapF fpr ndh ytgC btuF pepB nhaA
Bacillus solimangrovi
OEH93987.1 (1305675)
MJEH01000006.1 (genomic DNA)
ndh
Bacillus sp Marseille P3661
LT965953.1_318 (1936234)
LT965953.1 ()
yorfE yutH hom thrB trxA erpA trxB ndh ygdD pepB ygdD ykvM ribH ribE
Bacillus azotoformans MEV2011
KEF40075.1 (1348973)
JJRY01000001.1 (genomic DNA)
ndh
Bacillus oryziterrae
KB223527.1_1274 (1191699)
KB223527.1 ()
ndh
Bacillus marinisedimentorum
LWBL02000005.1_142 (1821260)
LWBL02000005.1 ()
thrB nifU yuzD ndh fmnP mqnE_1 iscA trxB ndh ygdD pepB yhgD
Bacillus sp OV194
SFD50827.1 (1881065)
FOML01000001.1 (genomic DNA)
ndh yxfA fmnP dapF mqnE fpr ndh ytgC btuF pepB nhaA
Fictibacillus arsenicus
ANX13403.1 (255247)
CP016761.1 (chromosome)
fpr ndh ytgC
Fictibacillus aquaticus
OYD56158.1 (2021314)
NOII01000041.1 (genomic DNA)
erpA fpr ndh ytgC
Bacillus sp AFS001701
PET71856.1 (2033480)
NTZO01000064.1 (genomic DNA)
ndh ndh
Bacillus sp SYSU K30001
RIAV01000027.1_14 (2486280)
RIAV01000027.1 ()
pleD trxB ndh mutT pleD pepA pleD manB gtaB bioY pleD comA
Bacillus thuringiensis str Al Hakam
ABK87661.1 (412694)
CP000485.1 (chromosome)

Anaerobacillus isosaccharinicus
OIJ13716.1 (1532552)
LQXD01000122.1 (genomic DNA)

Bacillus cellulosilyticus DSM 2522
ADU31644.1 (649639)
CP002394.1 (chromosome)

Bacillus sp FJAT 45348
CP025024.1_3660 (2011012)
CP025024.1 ()

Salisediminibacterium halotolerans
RPE89234.1 (517425)
RKQQ01000001.1 (genomic DNA)

Salipaludibacillus agaradhaerens
KV917378.1_3414 (76935)
KV917378.1 ()

Bacillus chagannorensis DSM 18086
AUCK01000013.1_7 (1121087)
AUCK01000013.1 ()

Bacillus urumqiensis
PRO65520.1 (1548213)
PVNS01000007.1 (genomic)

Alkalicoccus saliphilus
PTL38928.1 (200989)
PZJJ01000012.1 (genomic)

Salsuginibacillus kocurii DSM 18087
KB898623.1_529 (1123239)
KB898623.1 ()

Salsuginibacillus halophilus
PSL44465.1 (517424)
PYAV01000008.1 (genomic DNA)

Bacillus sp FJAT 44742
KZ454936.1_2849 (2014005)
KZ454936.1 ()

Salibacterium halotolerans
SFP51541.1 (1884432)
FOXD01000006.1 (genomic DNA)

Alteribacillus bidgolensis
KZ614149.1_2229 (930129)
KZ614149.1 ()

Sinobaca qinghaiensis
RKD68819.1 (342944)
RAPK01000012.1 (genomic DNA)

Alteribacillus iranensis
SFE94868.1 (930128)
FONT01000006.1 (genomic DNA)

Bacillus pseudofirmus
OLO28600.1 (79885)
MSLR01000019.1 (genomic)

Bacillus sp FJAT 45350
NISU01000002.1_195 (2011014)
NISU01000002.1 ()

Bacillus sp JCM 19034
BAXR01000002.1_123 (1481928)
BAXR01000002.1 ()

Bacillus pseudofirmus OF4
ADC49026.1 (398511)
CP001878.2 (chromosome)

Bacillus sp FJAT 45037
KZ454938.1_2547 (2011007)
KZ454938.1 ()

Bacillus halodurans C 125
BAB07126.1 (272558)
BA000004.3 (genomic DNA)

Bacillus bogoriensis ATCC BAA 922
JHYI01000006.1_418 (1408424)
JHYI01000006.1 ()

Bacillus sp JCM 19034
BAXR01000002.1_122 (1481928)
BAXR01000002.1 ()

Bacillus pseudalcaliphilus
KMK77671.1 (79884)
LFJO01000002.1 (genomic DNA)

Bacillus clausii KSM K16
BAD65458.1 (66692)
AP006627.1 (genomic DNA)

Bacillus lonarensis
SDB88378.1 (1464122)
FMYM01000002.1 (genomic DNA)

Bacillus testis
LN851186.1_3245 (1622072)
LN851186.1 ()

Bacillus massilioanorexius AP8
CAPG01000076.1_40 (1211814)
CAPG01000076.1 ()

Bacillaceae bacterium EAG3
PQD95175.1 (2064768)
PKOZ01000005.1 (genomic DNA)

Pontibacillus marinus BH030004 DSM 16465
AULJ01000019.1_21 (1385511)
AULJ01000019.1 ()

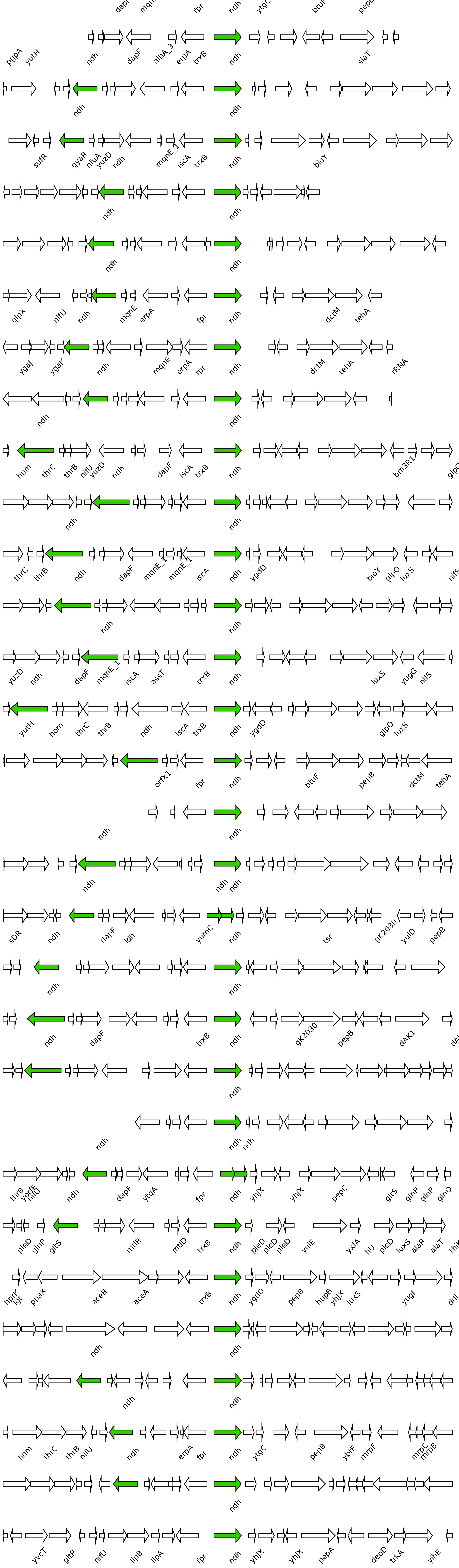

Pontibacillus chungwhensis BH030062
KGP91670.1 (1385513)

KGP91678.1 (1385513)
AVBG01000005.1 (genomic DNA)

Pontibacillus yanchengensis Y32
KGP70799.1 (1385514)
AVBF01000115.1 (genomic DNA)

Pontibacillus litoralis JSM 072002
KGX86717.1 (1385512)
AVPG01000011.1 (genomic DNA)

Pontibacillus halophilus JSM 076056 DSM 19796
AULI01000016.1_32 (1385510)
AULI01000016.1 ()

Thalassobacillus cyri
SEB08167.1 (571932)
FNQR01000015.1 (genomic DNA)

Salimicrobium flavidum
SIS43906.1 (570947)
FTOC01000003.1 (genomic DNA)

Halobacillus halophilus DSM 2266
CCG46243.1 (866895)
HE717023.1 (genomic DNA)

Sediminibacillus albus
SDK08014.1 (407036)
FNFL01000002.1 (genomic DNA)

Virgibacillus necropolis
ASN07330.1 (163877)
CP022437.1 (chromosome)

Virgibacillus salinus
SDQ28873.1 (553311)
FNKD01000001.1 (genomic DNA)

Virgibacillus ndiopensis
FZMZ01000006.1_1247 (2004408)
FZMZ01000006.1 ()

Citricoccus massiliensis
LT966071.1_1500 (2045010)
LT966071.1 ()

Lentibacillus sp Marseille P4043
LT984884.1_2101 (2040293)
LT984884.1 ()

Virgibacillus profundi
PAV28746.1 (2024555)
NPOA01000011.1 (genomic DNA)

Virgibacillus indicus
OZU88564.1 (2024554)
NPMS01000004.1 (genomic DNA)

Ornithinibacillus halophilus
SHG42550.1 (930117)
FQVW01000032.1 (genomic DNA)

Oceanobacillus limi
SET34063.1 (930131)
FOHE01000009.1 (genomic DNA)

Paucisalibacillus globulus
LT855389.1_44 (351095)
LT855389.1 ()

Virgibacillus sp LJ137
QTSZ01000014.1_17 (2293253)
QTSZ01000014.1 ()

Oceanobacillus iheyensis
AVQ99724.1 (182710)
CP020357.1 (chromosome)

Saliterribacillus persicus
RCW66939.1 (930114)
QPJJ01000008.1 (genomic DNA)

Gracilibacillus halophilus YIM C55 5
ENH97766.1 (1308866)
APML01000015.1 (genomic)

Gracilibacillus dipsosauri
PWU68175.1 (178340)
QGTD01000009.1 (genomic DNA)

Gracilibacillus kekensis
SHM92271.1 (1027249)
FRCZ01000002.1 (genomic DNA)

Gracilibacillus boraciitolerans JCM 21714
GAE94000.1 (1298598)
BAVS01000018.1 (genomic DNA)

Gracilibacillus boraciitolerans JCM 21714
GAE93999.1 (1298598)
BAVS01000018.1 (genomic DNA)

Salinibacillus kushneri
SES64828.1 (237682)
FOHJ01000001.1 (genomic DNA)

Salirhabdus sp Marseille P4669
OEQK01000006.1_41 (2042310)
OEQK01000006.1 ()

Halalkalibacillus sp B3227
PKR78112.1 (2018042)
PJNH01000002.1 (genomic)

Aquisalibacillus elongatus
RPF50314.1 (485577)
RKRF01000013.1 (genomic DNA)

Tenuibacillus multivorans
SDN97747.1 (237069)
FNIG01000013.1 (genomic DNA)

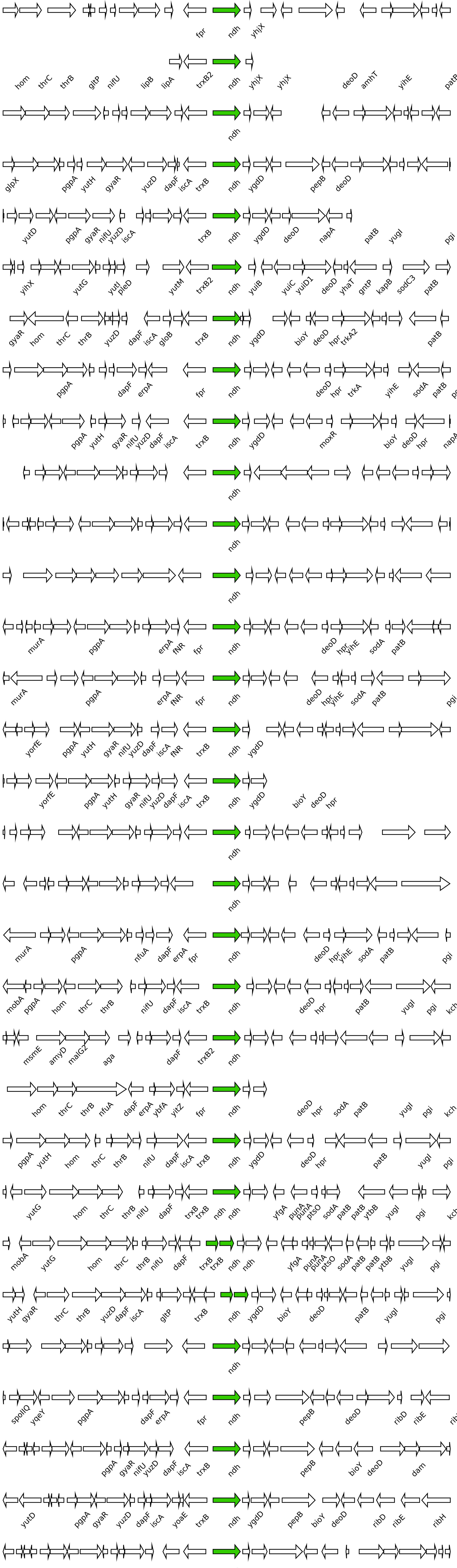

Alkalibacillus haloalkaliphilus C5
AKIF01000092.1_12 (1193119)
AKIF01000092.1 ()

Halalkalibacillus halophilus DSM 18494
AUHI01000011.1_65 (1121936)
AUHI01000011.1 ()

Lentibacillus halodurans
SFA91385.1 (237679)
FOJW01000003.1 (genomic DNA)

Lentibacillus amyloliquefaciens
ALX50495.1 (1472767)
CP013862.1 (chromosome)

Lentibacillus jeotgali
AGAV01000023.1_58 (558169)
AGAV01000023.1 ()

Oceanobacillus damuensis
KV392045.1_51 (937928)
KV392045.1 ()

Oceanobacillus rekensis
MTBQ01000002.1_603 (937927)
MTBQ01000002.1 ()

Oceanobacillus sp 160
AXI09854.1 (2052660)
CP024848.1 (chromosome)

Virgibacillus proomii
FUFN01000008.1_57 (84407)
FUFN01000008.1 ()

bacterium 1XD42 44
RKJ57204.1 (2320106)
RAZI01000008.1 (genomic DNA)

Virgibacillus pantothenticus
KNE22172.1 (1473)
LGTO01000004.1 (genomic DNA)

Lentibacillus sediminis
NIXK01000015.1_43 (1940529)
NIXK01000015.1 ()

Bacillus sp SA5d 4
OZM55749.1 (1904753)
NPIA01000014.1 (genomic DNA)

Terribacillus goriensis
AIF68231.1 (386490)
CP008876.1 (chromosome)

Massilibacterium senegalense
LN831786.1_1560 (1632858)
LN831786.1 ()

Jeotgalibacillus malaysiensis
AJD91917.1 (1508404)
CP009416.1 (chromosome)

Jeotgalibacillus campisalis
KIL43673.1 (220754)
JXRR01000021.1 (genomic DNA)

Lysinibacillus saudimassiliensis
CEA06016.1 (1461583)
LN483081.1 (genomic DNA)

Bacillus sp KCTC 13219
KYG90443.1 (1811976)
LUFJ01000012.1 (genomic DNA)

Solibacillus sp R5 41
ATP41559.1 (2048654)
CP024123.1 (chromosome)

Solibacillus silvestris StLB046
BAK15493.1 (1002809)
AP012157.1 (genomic DNA)

Bacillus sp FJAT 22090
ALC84604.1 (1581038)
CP012601.1 (chromosome)

Chryseomicrobium excrementi
PJK17246.1 (2041346)
PCGR01000002.1 (genomic DNA)

Planococcus maritimus
KYG57642.1 (192421)
LTZG01000041.1 (genomic DNA)

Paenisporosarcina sp K2R23 3
AYC29960.1 (2320858)
CP032418.1 (chromosome)

Paenisporosarcina sp TG20
ALJG01000183.1_1 (1211706)
ALJG01000183.1 ()

Sporosarcina sp HYO08
KXH79327.1 (1759557)
LPUT01000029.1 (genomic DNA)

Filibacter sp TB 66
VDC31937.1 (2483811)
UXAV01000043.1 (genomic DNA)

Sporosarcina psychrophila
AMQ05351.1 (1476)
CP014616.1 (chromosome)

Sporosarcina ureae
ARF13495.1 (1571)
CP015108.1 (chromosome)

Bacillus sp OxB 1
BAQ08521.1 (98228)

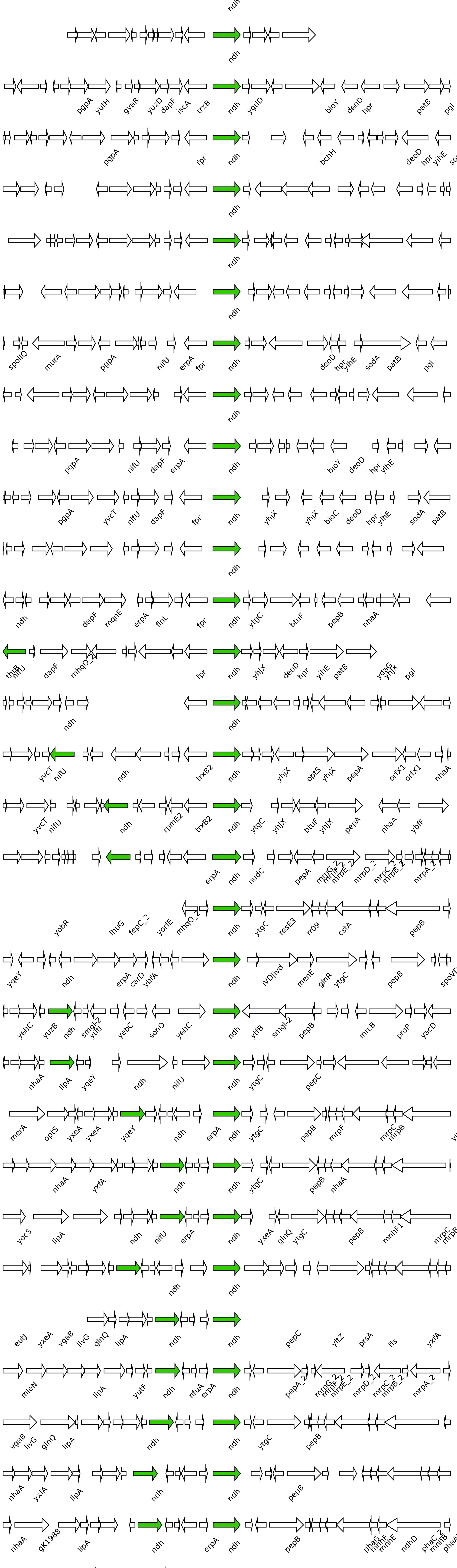

[illegible]
AP013294.1 (genomic DNA)

Sporosarcina pasteurii
SUJ15096.1 (1474)
UGYZ01000002.1 (genomic DNA)

Sporosarcina koreensis
LN651336.1_28 (334735)
LN651336.1 ()

Bhargavaea ginsengi
SEJ83582.1 (426757)
FNZF01000008.1 (genomic DNA)

Edaphobacillus lindanitolerans
SIT91980.1 (550447)
FTPL01000004.1 (genomic DNA)

Planomicrobium flavidum
NHTN01000016.1_125 (569883)
NHTN01000016.1 ()

Viridibacillus arvi
KOO47741.1 (263475)
LILB01000008.1 (genomic DNA)

Caryophanon latum
OCS92573.1 (33977)
MATO01000015.1 (genomic DNA)

Bacillus coagulans
KGB29067.1 (1398)
JPSK01000117.1 (genomic DNA)

Caenibacillus caldisaponilyticus
BDDQ01000021.1_8 (1674942)
BDDQ01000021.1 ()

Sporolactobacillus pectinivorans
NXGA01000001.1_1513 (1591408)
NXGA01000001.1 ()

Sporolactobacillus nakayamae
SFG93271.1 (269670)
FOOY01000031.1 (genomic DNA)

Tuberibacillus sp Marseille P3662
FXBS01000006.1_86 (1965358)
FXBS01000006.1 ()

Paraliobacillus ryukyuensis
RBO94571.1 (200904)
QNRI01000010.1 (genomic DNA)

Paraliobacillus sp X 1125
CP029797.1_1459 (2213194)
CP029797.1 ()

Paraliobacillus quinghaiensis
QJSH01000002.1_202 (470815)
QJSH01000002.1 ()

Gracilibacillus boraciitolerans JCM 21714
GAE92642.1 (1298598)
BAVS01000006.1 (genomic DNA)

Gracilibacillus kekensis
SHN08217.1 (1027249)
FRCZ01000003.1 (genomic DNA)

Gracilibacillus dipsosauri
PWU68279.1 (178340)
QGTD01000008.1 (genomic DNA)

Gracilibacillus halophilus YIM C55 5
ENH97941.1 (1308866)
APML01000010.1 (genomic)

Saliterribacillus persicus
RCW66383.1 (930114)
QPJJ01000009.1 (genomic DNA)

Sediminibacillus albus
SDJ77519.1 (407036)
FNFL01000001.1 (genomic DNA)

Salibacterium halotolerans
SFP49521.1 (1884432)
FOXD01000006.1 (genomic DNA)

Thalassobacillus cyri
SEA87709.1 (571932)
FNQR01000009.1 (genomic DNA)

Virgibacillus necropolis
ASN05967.1 (163877)
CP022437.1 (chromosome)

Virgibacillus salinus
SDQ66584.1 (553311)
FNKD01000002.1 (genomic DNA)

Virgibacillus siamensis
FUIH01000007.1_868 (480071)
FUIH01000007.1 ()

Lentibacillus halodurans
SFA85606.1 (237679)
FOJW01000002.1 (genomic DNA)

Lentibacillus amyloliquefaciens
ALX47284.1 (1472767)
CP013862.1 (chromosome)

Lentibacillus jeotgali
AGAV01000007.1_158 (558169)
AGAV01000007.1 ()

Virgibacillus ndiopensis
FZMZ01000005.1_48 (2004408)
FZMZ01000005.1 ()

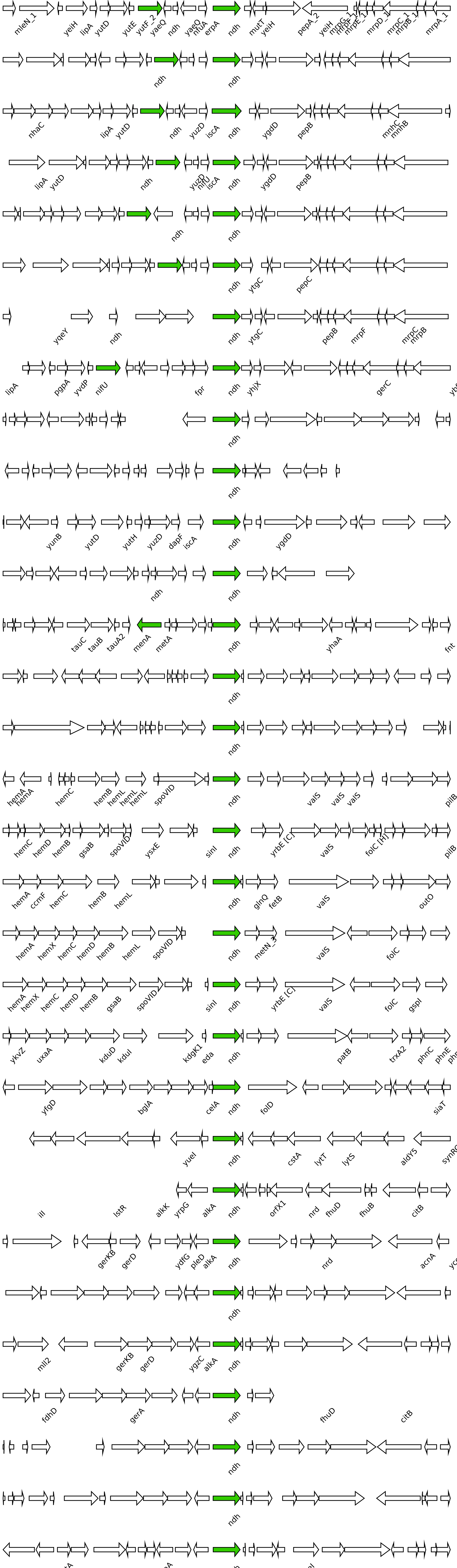

Ornithinibacillus halophilus
SHG78058.1 (930117)
FQVW01000061.1 (genomic DNA)

Oceanobacillus limi
SET27568.1 (930131)
FOHE01000008.1 (genomic DNA)

Lentibacillus sp Marseille P4043
LT984884.1_3207 (2040293)
LT984884.1 ()

Citricoccus massiliensis
LT966071.1_2579 (2045010)
LT966071.1 ()

Virgibacillus alimentarius
JFBD01000045.1_43 (698769)
JFBD01000045.1 ()

Virgibacillus sp LJ137
QTSZ01000017.1_308 (2293253)
QTSZ01000017.1 ()

Bacillales bacterium UBA6768
DKGO01000066.1_5 (1950334)
DKGO01000066.1 ()

Pseudogracilibacillus auburnensis
PXW87062.1 (1494959)
QJJQ01000006.1 (genomic DNA)

Bacillus sp JCM 19056
BAXM01000004.1_4 (1460650)
BAXM01000004.1 ()

Paucisalibacillus globulus
LT855386.1_358 (351095)
LT855386.1 ()

Oceanobacillus damuensis
LQNF01000014.1_90 (937928)
LQNF01000014.1 ()

Oceanobacillus rekensis
MTBQ01000003.1_280 (937927)
MTBQ01000003.1 ()

Oceanobacillus iheyensis HTE831
BAC13321.1 (221109)
BA000028.3 (genomic DNA)

Oceanobacillus sojae
LT727812.1_407 (582851)
LT727812.1 ()

Oceanobacillus sp 160
AXI08917.1 (2052660)
CP024848.1 (chromosome)

Oceanobacillus bengalensis
RKQ17513.1 (1435466)
RBZO01000004.1 (genomic DNA)

Oceanobacillus sp YLB 02
RLL45151.1 (2448030)
RCHR01000003.1 (genomic DNA)

Oceanobacillus senegalensis
LT839648.1_1872 (1936063)
LT839648.1 ()

Bacilli bacterium VT 13 104
KKE78993.1 (1637974)
LAZH01000039.1 (genomic DNA)

Lentibacillus sediminis
NIXK01000002.1_576 (1940529)
NIXK01000002.1 ()

Oceanobacillus iheyensis
AVQ98852.1 (182710)
CP020357.1 (chromosome)

Virgibacillus profundi
PAV28824.1 (2024555)
NPOA01000010.1 (genomic DNA)

Virgibacillus indicus
OZU90528.1 (2024554)
NPMS01000001.1 (genomic DNA)

Virgibacillus proomii
FUFN01000010.1_1382 (84407)
FUFN01000010.1 ()

Virgibacillus pantothenticus
KNE19994.1 (1473)
LGTO01000007.1 (genomic DNA)

bacterium 1XD42 44
RKJ54020.1 (2320106)
RAZI01000033.1 (genomic DNA)

Paenisporosarcina sp TG20
ALJG01000364.1_32 (1211706)
ALJG01000364.1 ()

Sinobaca qinghaiensis
RKD69731.1 (342944)
RAPK01000011.1 (genomic DNA)

Natribacillus halophilus
SDI79628.1 (549003)
FNEN01000006.1 (genomic DNA)

Salinibacillus kushneri
SET35377.1 (237682)
FOHJ01000004.1 (genomic DNA)

Geomicrobium sp JCM 19037
GAK05295.1 (1460634)

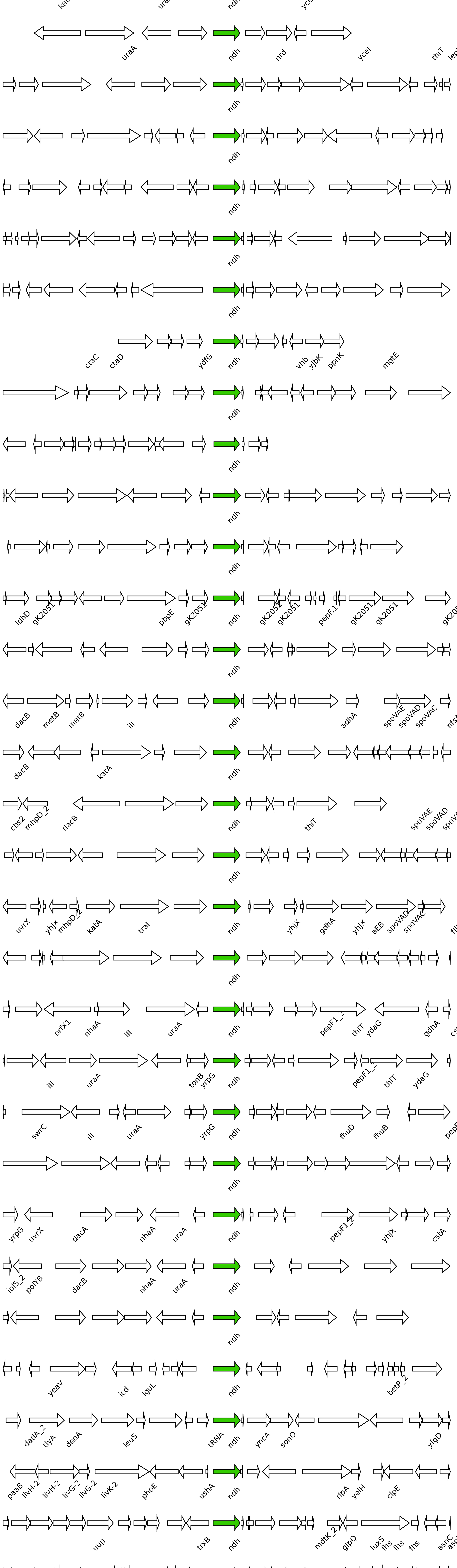

BAWZ01000033.1 (genomic DNA)

Ruegeria sp NKC1 1
AXF56124.1 (2099786)
CP031092.1 (chromosome)

Natribacillus halophilus
SDI81633.1 (549003)
FNEN01000006.1 (genomic DNA)

Marinococcus halophilus
OZT79437.1 (1371)
NPFA01000013.1 (genomic DNA)

Bacillus psychrosaccharolyticus ATCC 23296
AJTN02000200.1_47 (1174504)
AJTN02000200.1 ()

Virgibacillus alimentarius
JFBD01000074.1_41 (698769)
JFBD01000074.1 ()

Exiguobacterium sibiricum 255 15
ACB61763.1 (262543)
CP001022.1 (chromosome)

Exiguobacterium sp AT1b
ACQ69701.1 (360911)
CP001615.1 (chromosome)

Oceanobacillus iheyensis HTE831
BAC14306.1 (221109)
BA000028.3 (genomic DNA)

Bacilli bacterium VT 13 104
KKE79535.1 (1637974)
LAZH01000029.1 (genomic DNA)

Oceanobacillus sp YLB 02
RLL46491.1 (2448030)
RCHR01000002.1 (genomic DNA)

Lactobacillus acidophilus NCFM
AAV43434.1 (272621)
CP000033.3 (chromosome)

Lactobacillus pasteurii DSM 23907 CRBIP 24 76
CCI84307.1 (1423790)
CAKD01000001.1 (genomic DNA)

Lactobacillus gigeriorum DSM 23908 CRBIP 24 85
CCI87631.1 (1423751)
CAKC01000086.1 (genomic DNA)

Lactobacillus sp ASF360
EMZ25684.1 (97137)
KB822413.1 (genomic)

Lactobacillus acidophilus NCFM
AAV43433.1 (272621)
CP000033.3 (chromosome)

Weissella confusa
KRN22803.1 (1583)
JQAY01000007.1 (genomic)

Brochothrix thermosphacta
ANZ95625.1 (2756)
CP016839.1 (chromosome)

Oenococcus alcoholitolerans
KGO31661.1 (931074)
AXCV01000260.1 (genomic DNA)

Oenococcus oeni PSU 1
ABJ57673.1 (203123)
CP000411.1 (chromosome)

Oenococcus kitaharae DSM 17330
EHN59477.1 (1045004)
CM001398.1 (chromosome)

Lactobacillus shenzhenensis LY 73
ERL66608.1 (1231336)
KI271582.1 (genomic)

Lactobacillus perolens DSM 12744
KRL14101.1 (1423792)
AZEC01000002.1 (genomic)

Bifidobacterium asteroides PRL2011
AFU70885.1 (1147128)
CP003325.1 (chromosome)

Bifidobacterium aemilianum
RBP97430.1 (2493120)
PDCG01000006.1 (genomic DNA)

Bifidobacterium bombi DSM 19703
KFF31634.1 (1341695)
ATLK01000001.1 (genomic DNA)

Bifidobacterium xylocopae
RBP99125.1 (2493119)
PDCH01000010.1 (genomic DNA)

Bifidobacterium actinocoloniiforme DSM 22766
KFI40505.1 (1437605)
JGYK01000001.1 (genomic DNA)

Bifidobacterium commune
SCC79448.1 (1505727)
FMBL01000001.1 (genomic DNA)

Bifidobacterium bohemicum DSM 22767
KFI46991.1 (1437606)
JGYP01000001.1 (genomic DNA)

Bifidobacterium subtile
KFJ05321.1 (77635)
JGZR01000001.1 (genomic DNA)

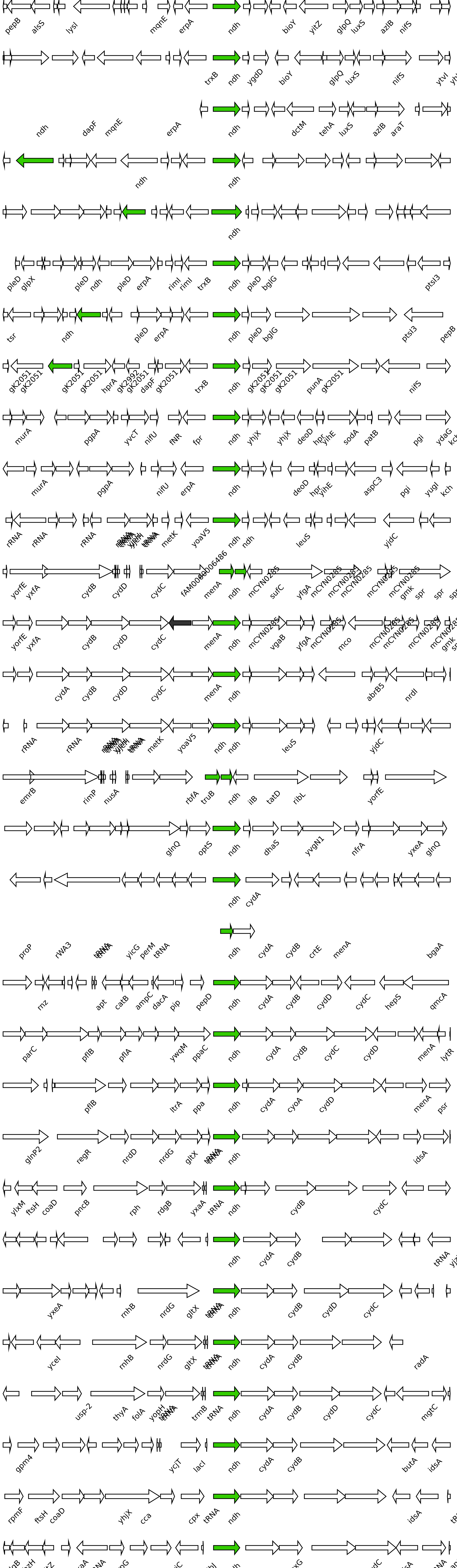

Bifidobacterium mongoliense
ROT87635.1 (518643)
QRAJ01000001.1 (genomic DNA)

Bifidobacterium aquikefiri
OZG65170.1 (1653207)
MWXA01000009.1 (genomic DNA)

Lactobacillus brevis ATCC 367
ABJ63486.1 (387344)
CP000416.1 (chromosome)

Lactobacillus plantarum WCFS1
CCC77837.1 (220668)
AL935263.2 (genomic DNA)

Lactobacillus brantae DSM 23927
KRM71596.1 (1423727)
AYZQ01000003.1 (genomic)

Lactobacillus saniviri JCM 17471   DSM 24301
BBBX01000026.1_19 (1293598)
BBBX01000026.1 ()

Lactobacillus composti DSM 18527   JCM 14202
GAF41795.1 (1423734)
BAMK01000047.1 (genomic DNA)

Lactobacillus sp 54 2
RHOY01000016.1_8 (2485997)
RHOY01000016.1 ()

Lactobacillus mellifer
KJY60524.1 (1218492)
KQ034029.1 (genomic)

Lactobacillus bombi
RHW48508.1 (1303590)
QOCS01000004.1 (genomic DNA)

Lactobacillus terrae
CP024610.1_245 (2269374)
CP024610.1 ()

Lactobacillus tucceti DSM 20183
KRK64108.1 (1423811)
AZDG01000016.1 (genomic)

Lactobacillus mindensis DSM 14500
KRL43419.1 (1423770)
AZEZ01000086.1 (genomic)

Lactobacillus heilongjiangensis
ALB30008.1 (1074467)
CP012559.1 (chromosome)

Lactobacillus allii
APX71559.1 (1847728)
CP019323.1 (chromosome)

Lactobacillus ginsenosidimutans
AKP67705.1 (1007676)
CP012034.1 (chromosome)

Lactobacillus hokkaidonensis JCM 18461
BAP86749.1 (1291742)
AP014680.1 (genomic DNA)

Lactobacillus coryniformis subsp coryniformis KCTC 3167   DSM 20001
GL544636.1_11 (913848)
GL544636.1 ()

Lactobacillus bifermentans DSM 20003
KRK40946.1 (1423726)
AZDA01000001.1 (genomic)

Paraliobacillus sp X 1125
CP029797.1_2639 (2213194)
CP029797.1 ()

Paraliobacillus quinghaiensis
QJSH01000023.1_25 (470815)
QJSH01000023.1 ()

Paraliobacillus ryukyuensis
RBO98102.1 (200904)
QNRI01000006.1 (genomic DNA)

Amphibacillus marinus
SEO71932.1 (872970)
FODJ01000011.1 (genomic DNA)

Amphibacillus xylanus NBRC 15112
BAM46978.1 (698758)
AP012050.1 (genomic DNA)

Amphibacillus jilinensis Y1
JH976436.1_26 (562743)
JH976436.1 ()

Amphibacillus sediminis NBRC 103570
BCQW01000014.1_61 (1220586)
BCQW01000014.1 ()

Pelagirhabdus alkalitolerans
SDC46580.1 (1612202)
FMYI01000009.1 (genomic DNA)

Streptohalobacillus salinus
PXW91392.1 (621096)
QJJR01000005.1 (genomic DNA)

Halolactibacillus alkaliphilus
SFP02940.1 (442899)
FOWN01000034.1 (genomic DNA)

Heliobacterium modesticaldum Ice1
ABZ83564.1 (498761)
CP000930.2 (chromosome)

Heliobacterium modesticaldum Ice1
ABZ84099.1 (498761)

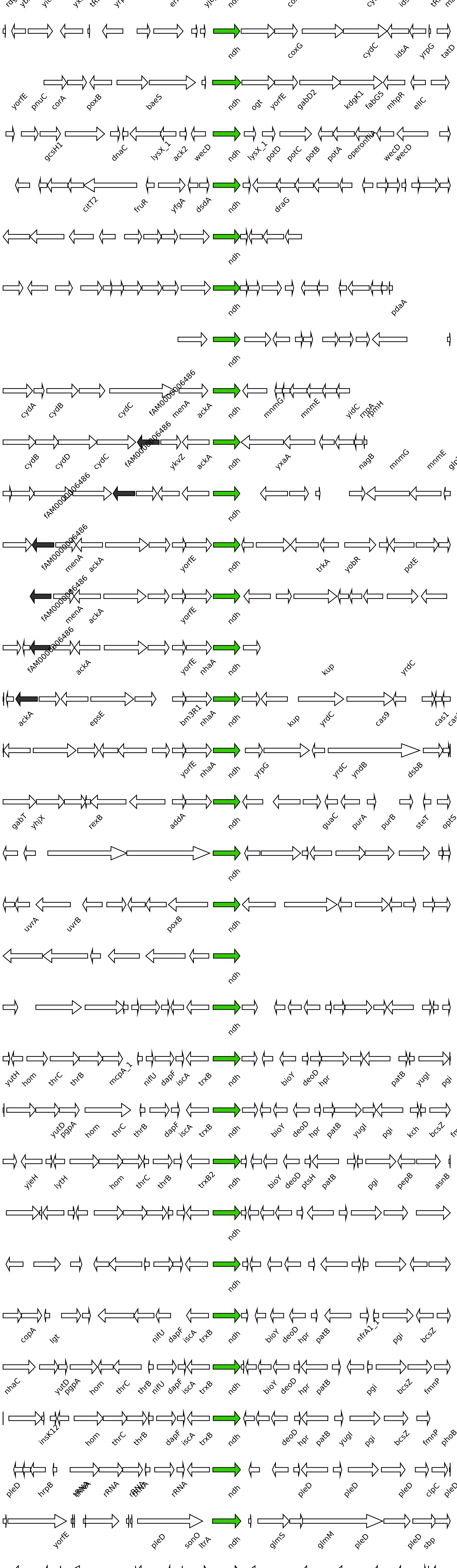

CP000930.2 (chromosome)

Kurthia massiliensis
HE611001.1_16 (1033739)
HE611001.1 ()

Lactobacillus lindneri
ANZ58396.1 (53444)
CP014872.1 (chromosome)

Lactobacillus sanfranciscensis DSM 20451
KRM80947.1 (1423800)
AYYM01000002.1 (genomic)

Lactobacillus homohiochii
KRN41385.1 (33961)
JQBN01000002.1 (genomic)

Lactobacillus kosoi
GAY79874.1 (2051900)
BEXE01000013.1 (genomic DNA)

Pasteuria penetrans
VDN74056.1 (86005)
UZAC01000005.1 (genomic DNA)

Alicyclobacillus acidocaldarius subsp acidocaldarius DSM 446
ACV57887.1 (521098)
CP001727.1 (chromosome)

Cyanobacteria bacterium UBA999
DBAX01000027.1_35 (1947904)
DBAX01000027.1 ()

Nitriliruptoraceae bacterium
PULK01000262.1_9 (2093808)
PULK01000262.1 ()

Egibacter sp
PWFH01000126.1_7 (2003555)
PWFH01000126.1 ()

Euzebya tangerina
PPDK01000003.1_223 (591198)
PPDK01000003.1 ()

Euzebya rosea
PGDQ01000001.1_176 (2052804)
PGDQ01000001.1 ()

Actinobacteria bacterium IMCC26207
KLR62507.1 (1641811)
LCZK01000003.1 (genomic DNA)

Cellulomonas sp PhB150
ROS28134.1 (2485188)
RKHX01000002.1 (genomic DNA)

Microbacterium mangrovi
KHK99107.1 (1348253)
JTDK01000005.1 (genomic DNA)

Micropruina glycogenica
SPD88338.1 (75385)
LT985188.1 (genomic DNA)

Cellulomonas fimi
VEH26179.1 (1708)
LR134387.1 (genomic DNA)

Georgenia muralis
RPF26949.1 (154117)
RKRA01000001.1 (genomic DNA)

Ornithinimicrobium pekingense
SOC56649.1 (384677)
OBQK01000008.1 (genomic DNA)

Ornithinimicrobium sp AMA3305
AXH96486.1 (2283195)
CP031229.1 (chromosome)

Deinococcus swuensis
AIZ44742.1 (1182571)
CP010028.1 (chromosome)

Deinococcus koreensis
PNY82310.1 (2054903)
PPPD01000001.1 (genomic)

Deinococcus pimensis DSM 21231
KI912676.1_87 (926554)
KI912676.1 ()

Deinococcus maricopensis DSM 21211
ADV67597.1 (709986)
CP002454.1 (chromosome)

Amnibacterium sp DD4a
RIX27587.1 (2306585)
QXTG01000002.1 (genomic DNA)

Hydrogenimonas sp
BBG66850.1 (2231112)
AP019005.1 (genomic DNA)

Hydrogenimonas thermophila
SFP64850.1 (223786)
FOXB01000030.1 (genomic DNA)

Nitratiruptor sp SB155 2
BAF70081.1 (387092)
AP009178.1 (genomic DNA)

Nitratiruptor tergarcus DSM 16512
SMC09788.1 (1069081)
FWWZ01000001.1 (genomic DNA)

Hydrogenimonas thermophila
SFP84520.1 (223786)
FOXB01000046.1 (genomic DNA)

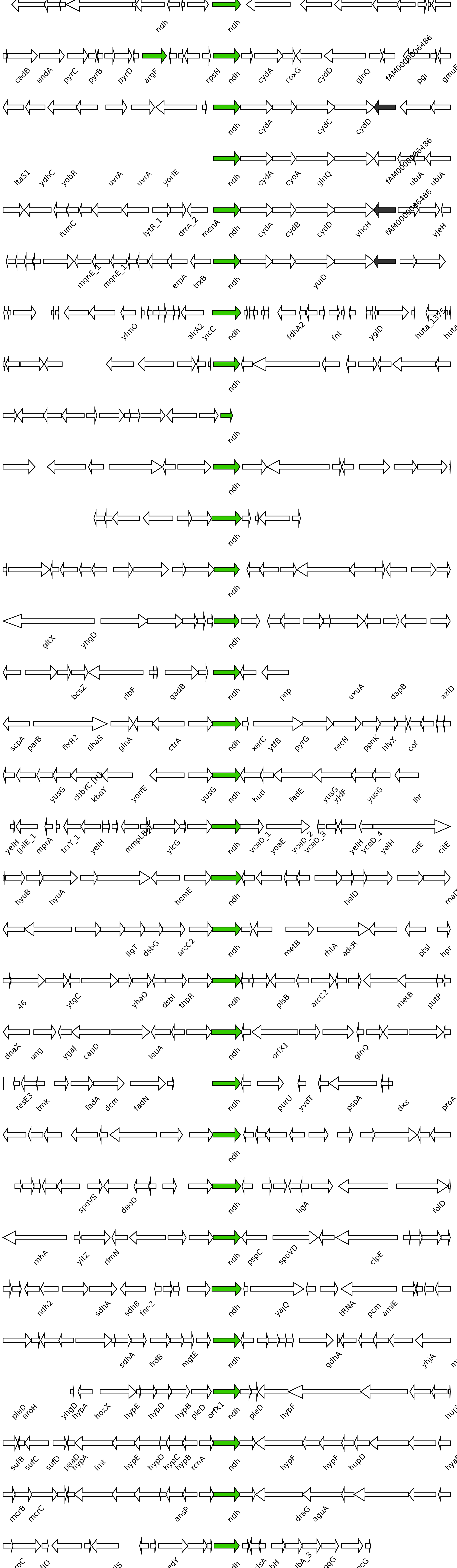

Sulfuricurvum kujiense DSM 16994
ADR32978.1 (709032)
CP002355.1 (chromosome)

Sulfuricurvum sp 17 40 25
OZA03351.1 (1970439)
NCIJ01000003.1 (genomic DNA)

Sulfuricurvum kujiense DSM 16994
ADR33482.1 (709032)
CP002355.1 (chromosome)

Sulfuricurvum sp UBA2173
DCWB01000004.1_103 (1947585)
DCWB01000004.1 ()

Sulfurimonas sp GWF2_37_8
OHD95016.1 (1802250)
MIBW01000033.1 (genomic DNA)

Sulfurimonas autotrophica DSM 16294
ADN08450.1 (563040)
CP002205.1 (chromosome)

Sulfurimonas hongkongensis
EQB39798.1 (1172190)
AUPZ01000005.1 (genomic DNA)

Sulfurimonas gotlandica GD1
EDZ63069.1 (929558)
DS995286.1 (genomic)

Sulfurimonas sp RIFCSPLOWO2_02_FULL_36_28
OHD98128.1 (1802252)
MIBY01000103.1 (genomic DNA)

Sulfurimonas sp RIFOXYB12_FULL_35_9
OHE04388.1 (1802256)
MICC01000087.1 (genomic DNA)

Sulfurimonas sp RIFOXYD12_FULL_33_39
OHE09689.1 (1802259)
MICF01000005.1 (genomic DNA)

Sulfurimonas sp UBA2011
DDCH01000011.1_89 (1947587)
DDCH01000011.1 ()

Epsilonproteobacteria bacterium tcs 49
LLYS01000001.1_93 (1742865)
LLYS01000001.1 ()

Epsilonproteobacteria bacterium UBA6211
DISL01000114.1_3 (1948573)
DISL01000114.1 ()

Sulfuricurvum sp PC08 66
KIM11054.1 (1539066)
JQIT01000006.1 (genomic DNA)

Sulfuricurvum sp
QKCD01000120.1_3 (2025608)
QKCD01000120.1 ()

Helicobacteraceae bacterium CG2_30_36_10
OIP53884.1 (1805215)
MNYP01000127.1 (genomic DNA)

Sulfurimonas sp UBA6014
DJAA01000072.1_60 (1947588)
DJAA01000072.1 ()

Sulfurimonas sp RIFOXYD12_FULL_33_39
OHE09040.1 (1802259)
MICF01000010.1 (genomic DNA)

Sulfurovum sp AR
EIF52021.1 (1165841)
AJLE01000001.1 (genomic DNA)

Sulfurovum sp 24 42 9
OYZ50230.1 (1970443)
NCHL01000006.1 (genomic DNA)

Sulfurimonas hongkongensis
EQB39716.1 (1172190)
AUPZ01000006.1 (genomic DNA)

Sulfurovum sp UBA6199
DISX01000002.1_230 (1947608)
DISX01000002.1 ()

Sulfurovum sp UBA12169
DAB41099.1 (2015906)
DLUH01000001.1 (genomic DNA)

Sulfurimonas sp RIFOXYB12_FULL_35_9
OHE05134.1 (1802256)
MICC01000062.1 (genomic DNA)

Sulfurimonas denitrificans DSM 1251
ABB44491.1 (326298)
CP000153.1 (chromosome)

Hydrogenimonas sp
BBG66267.1 (2231112)
AP019005.1 (genomic DNA)

Hydrogenivirga sp 128 5 R1 1
EDP73318.1 (392423)
ABHJ01000333.1 (genomic DNA)

Persephonella marina EX H1
ACO04606.1 (123214)
CP001230.1 (chromosome)

Persephonella hydrogeniphila
SNZ10079.1 (198703)
OBEI01000009.1 (genomic DNA)

Epsilonproteobacteria bacterium tcs 49
LLYS01000008.1_100 (1742865)

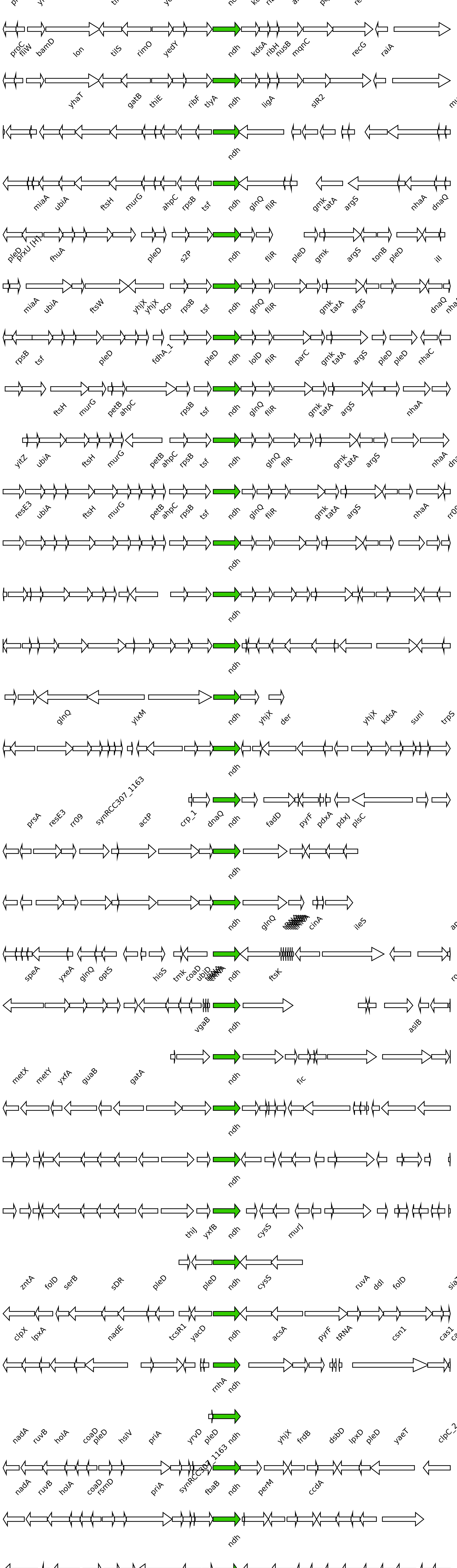

LLYS01000008.1 ()

Arcobacter halophilus
PKI79636.1 (197482)
NXIF01000093.1 (genomic DNA)

Arcobacter butzleri RM4018
ABV68433.1 (367737)
CP000361.1 (chromosome)

Arcobacter sp CECT 9188
RBQ26551.1 (2044505)
PDKI01000005.1 (genomic DNA)

Arcobacter sp AF1028
JART01000025.1_64 (1355368)
JART01000025.1 ()

Arcobacter anaerophilus
JXXG01000003.1_458 (877500)
JXXG01000003.1 ()

Arcobacter nitrofigilis DSM 7299
ADG93110.1 (572480)
CP001999.1 (chromosome)

Arcobacter sp UBA7394
DKNG01000174.1_7 (1946052)
DKNG01000174.1 ()

Arcobacter sp LPB0137
APW66791.1 (1850254)
CP019070.1 (chromosome)

Pseudoarcobacter caeni
PUE64422.1 (1912877)
MUXE01000007.1 (genomic)

Helicobacteraceae bacterium UBA6016
DIZY01000094.1_16 (1951927)
DIZY01000094.1 ()

Thermofilum uzonense
AKG38055.1 (1550241)
CP009961.1 (chromosome)

Thermofilum sp NZ13
PLJ77298.1 (1980981)
NDWX01000009.1 (genomic DNA)

Thermofilum carboxyditrophus 1505
AJB42524.1 (697581)
CP007493.1 (chromosome)

Thermofilum pendens Hrk 5
ABL77636.1 (368408)
CP000505.1 (chromosome)

Rubeoparvulum massiliense
CVPE01000006.1_1115 (1631346)
CVPE01000006.1 ()

Pontibacillus halophilus JSM 076056 DSM 19796
AULI01000013.1_82 (1385510)
AULI01000013.1 ()

Pontibacillus litoralis JSM 072002
KGX85579.1 (1385512)
AVPG01000021.1 (genomic DNA)

Pontibacillus marinus BH030004 DSM 16465
AULJ01000012.1_38 (1385511)
AULJ01000012.1 ()

Pontibacillus yanchengensis Y32
KGP71299.1 (1385514)
AVBF01000071.1 (genomic DNA)

Salirhabdus sp Marseille P4669
OEQK01000009.1_39 (2042310)
OEQK01000009.1 ()

Salinibacillus kushneri
SET95962.1 (237682)
FOHJ01000012.1 (genomic DNA)

Sediminibacillus albus
SDK04209.1 (407036)
FNFL01000002.1 (genomic DNA)

Thalassobacillus cyri
SEA83476.1 (571932)
FNQR01000009.1 (genomic DNA)

Pontibacillus chungwhensis BH030062
KGP92635.1 (1385513)
AVBG01000002.1 (genomic DNA)

Virgibacillus indicus
OZU89318.1 (2024554)
NPMS01000002.1 (genomic DNA)

Virgibacillus profundi
PAV31581.1 (2024555)
NPOA01000001.1 (genomic DNA)

Oceanobacillus iheyensis
AVR00735.1 (182710)
CP020357.1 (chromosome)

Oceanobacillus iheyensis
AVR00750.1 (182710)
CP020357.1 (chromosome)

Virgibacillus proomii
FUFN01000009.1_759 (84407)
FUFN01000009.1 ()

Virgibacillus pantothenticus
KNE21726.1 (1473)
LGTO01000005.1 (genomic DNA)

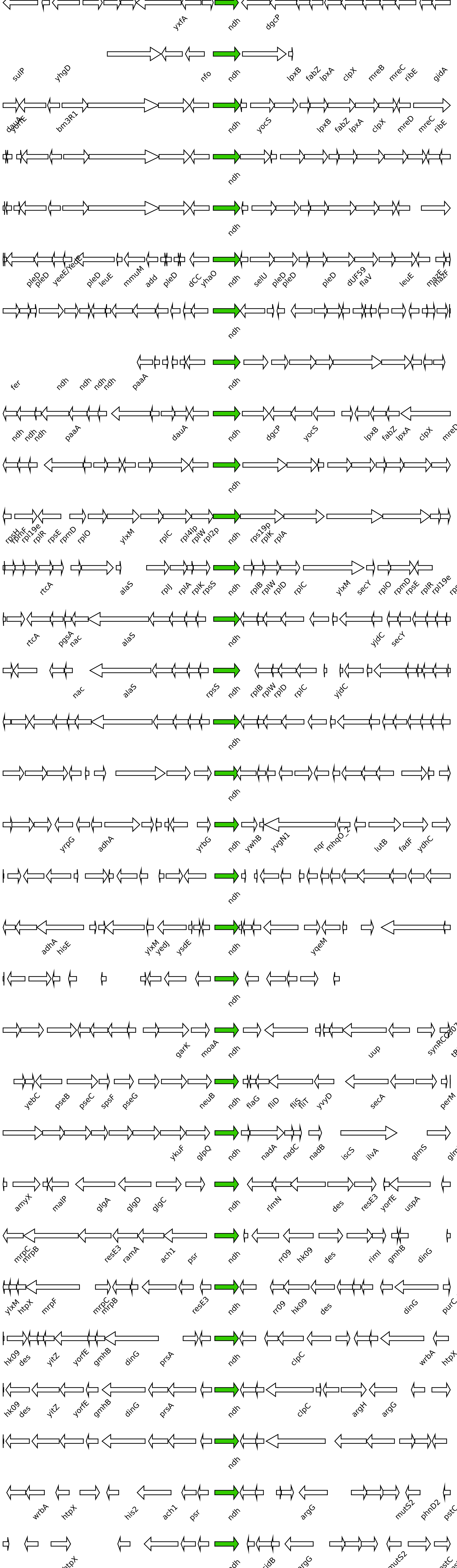

bacterium 1XD42 44
RKJ55664.1 (2320106)
RAZI01000017.1 (genomic DNA)

Virgibacillus ndiopensis
FZMZ01000006.1_346 (2004408)
FZMZ01000006.1 ()

Lentibacillus sp Marseille P4043
LT984884.1_1043 (2040293)
LT984884.1 ()

Citricoccus massiliensis
LT966071.1_4375 (2045010)
LT966071.1 ()

Oceanobacillus damuensis
LQNF01000030.1_2 (937928)
LQNF01000030.1 ()

Oceanobacillus rekensis
MTBQ01000004.1_212 (937927)
MTBQ01000004.1 ()

Oceanobacillus iheyensis HTE831
BAC15073.1 (221109)
BA000028.3 (genomic DNA)

Oceanobacillus sojae
LT727811.1_189 (582851)
LT727811.1 ()

Oceanobacillus sp 160
AXI10807.1 (2052660)
CP024848.1 (chromosome)

Oceanobacillus bengalensis
RKQ15806.1 (1435466)
RBZO01000011.1 (genomic DNA)

Oceanobacillus sp YLB 02
RLL42741.1 (2448030)
RCHR01000005.1 (genomic DNA)

Oceanobacillus senegalensis
LT839648.1_3473 (1936063)
LT839648.1 ()

Bacilli bacterium VT 13 104
KKE79271.1 (1637974)
LAZH01000033.1 (genomic DNA)

Virgibacillus necropolis
ASN03924.1 (163877)
CP022437.1 (chromosome)

Virgibacillus salinus
SDQ09009.1 (553311)
FNKD01000001.1 (genomic DNA)

Lentibacillus amyloliquefaciens
ALX49670.1 (1472767)
CP013862.1 (chromosome)

Lentibacillus jeotgali
AGAV01000015.1_253 (558169)
AGAV01000015.1 ()

Lentibacillus halodurans
SFA95885.1 (237679)
FOJW01000004.1 (genomic DNA)

Virgibacillus siamensis
FUIH01000007.1_3017 (480071)
FUIH01000007.1 ()

Ornithinibacillus halophilus
SHG01843.1 (930117)
FQVW01000012.1 (genomic DNA)

Oceanobacillus limi
SET53849.1 (930131)
FOHE01000014.1 (genomic DNA)

Paucisalibacillus globulus
LT855395.1_398 (351095)
LT855395.1 ()

Halobacillus halophilus DSM 2266
CCG44122.1 (866895)
HE717023.1 (genomic DNA)

Virgibacillus alimentarius
JFBD01000082.1_4 (698769)
JFBD01000082.1 ()

Virgibacillus sp LJ137
QTSZ01000041.1_67 (2293253)
QTSZ01000041.1 ()

Lentibacillus sediminis
NIXK01000013.1_32 (1940529)
NIXK01000013.1 ()

Pseudogracilibacillus auburnensis
PXW87548.1 (1494959)
QJJQ01000005.1 (genomic DNA)

Bacillales bacterium UBA6768
DKGO01000195.1_21 (1950334)
DKGO01000195.1 ()

Salimicrobium flavidum
SIS49106.1 (570947)
FTOC01000006.1 (genomic DNA)

Saliterribacillus persicus
RCW74710.1 (930114)
QPJJ01000003.1 (genomic DNA)

Gracilibacillus halophilus YIM C55 5
ENH98411.1 (1308866)
APML01000003.1 (genomic

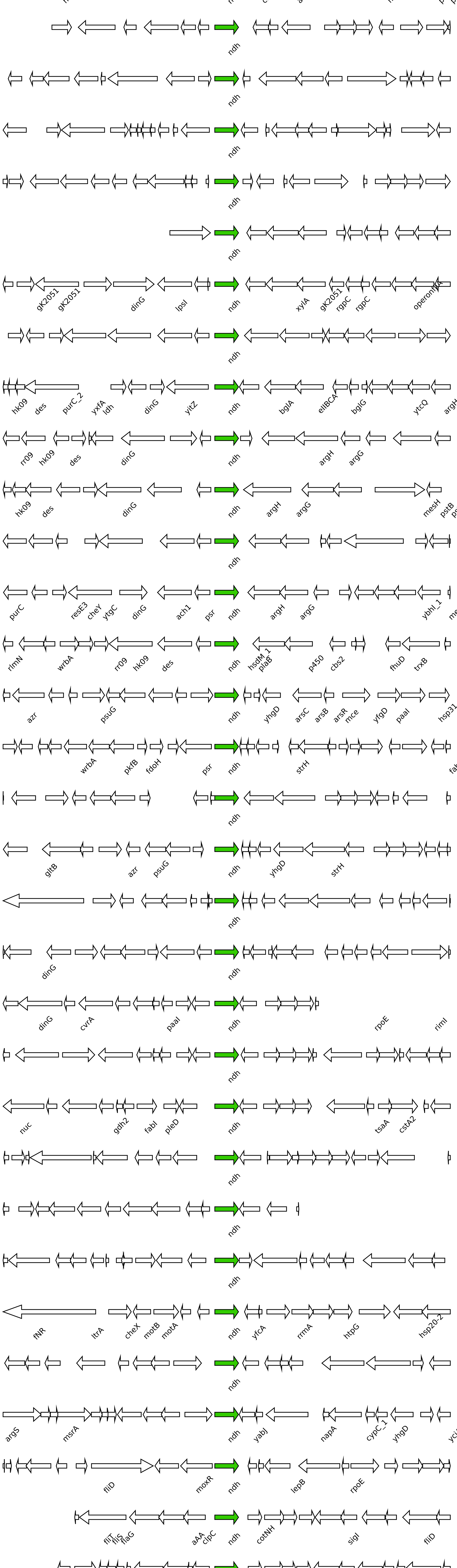

AFML01000003.1 (genomic

Gracilibacillus dipsosauri
PWU69292.1 (178340)
QGTD01000005.1 (genomic DNA)

Gracilibacillus kekensis
SHM96195.1 (1027249)
FRCZ01000002.1 (genomic DNA)

Gracilibacillus boraciitolerans JCM 21714
GAE95169.1 (1298598)
BAVS01000042.1 (genomic DNA)

Bacillus sp JCM 19056
BAXM01000026.1_53 (1460650)
BAXM01000026.1 ()

Brevibacillus sp SCSIO 07484
CP034541.1_3054 (2496837)
CP034541.1 ()

Brevibacillus thermoruber 423
KE695649.1_305 (1346613)
KE695649.1 ()

Brevibacillus laterosporus
JNFS01000001.1_701 (1465)
JNFS01000001.1 ()

Bacillus mannanilyticus JCM 10596
BAMO01000039.1_31 (1236954)
BAMO01000039.1 ()

Caldalkalibacillus thermarum TA2 A1
EGL83021.1 (986075)
AFCE01000129.1 (genomic DNA)

Anaerobacillus isosaccharinicus
OIJ22802.1 (1532552)
LQXD01000027.1 (genomic DNA)

Bacillus cellulosilyticus DSM 2522
ADU31651.1 (649639)
CP002394.1 (chromosome)

Bacillus sp FJAT 45348
CP025024.1_3652 (2011012)
CP025024.1 ()

Salipaludibacillus agaradhaerens
KV917378.1_3407 (76935)
KV917378.1 ()

Salisediminibacterium halotolerans
RPE89241.1 (517425)
RKQQ01000001.1 (genomic DNA)

Bacillus selenitireducens MLS10
ADH98689.1 (439292)
CP001791.1 (chromosome)

Alkalicoccus saliphilus
PTL38923.1 (200989)
PZJJ01000012.1 (genomic)

Bacillus chagannorensis DSM 18086
AUCK01000013.1_13 (1121087)
AUCK01000013.1 ()

Bacillus urumqiensis
PRO65527.1 (1548213)
PVNS01000007.1 (genomic)

Massilibacterium senegalense
LN831786.1_1568 (1632858)
LN831786.1 ()

Anaerobacillus macyae
KMM37164.1 (157733)
LELK01000004.1 (genomic DNA)

Bacillus taeanensis
RBW71296.1 (273032)
QOCW01000001.1 (genomic DNA)

Bacillus sp SA5d 4
OZM55740.1 (1904753)
NPIA01000014.1 (genomic DNA)

Bacillus bogoriensis ATCC BAA 922
JHYI01000031.1_4 (1408424)
JHYI01000031.1 ()

Bacillus halodurans C 125
BAB07134.1 (272558)
BA000004.3 (genomic DNA)

Bacillus pseudofirmus OF4
ADC49035.1 (398511)
CP001878.2 (chromosome)

Bacillus sp FJAT 45037
KZ454938.1_2556 (2011007)
KZ454938.1 ()

Bacillus pseudalcaliphilus
KMK77870.1 (79884)
LFJO01000002.1 (genomic DNA)

Bacillus sp JCM 19034
BAXR01000002.1_114 (1481928)
BAXR01000002.1 ()

Bacillus sp FJAT 45350
NISU01000002.1_187 (2011014)
NISU01000002.1 ()

Bacillus clausii KSM K16
BAD65471.1 (66692)
AP006627.1 (genomic DNA)

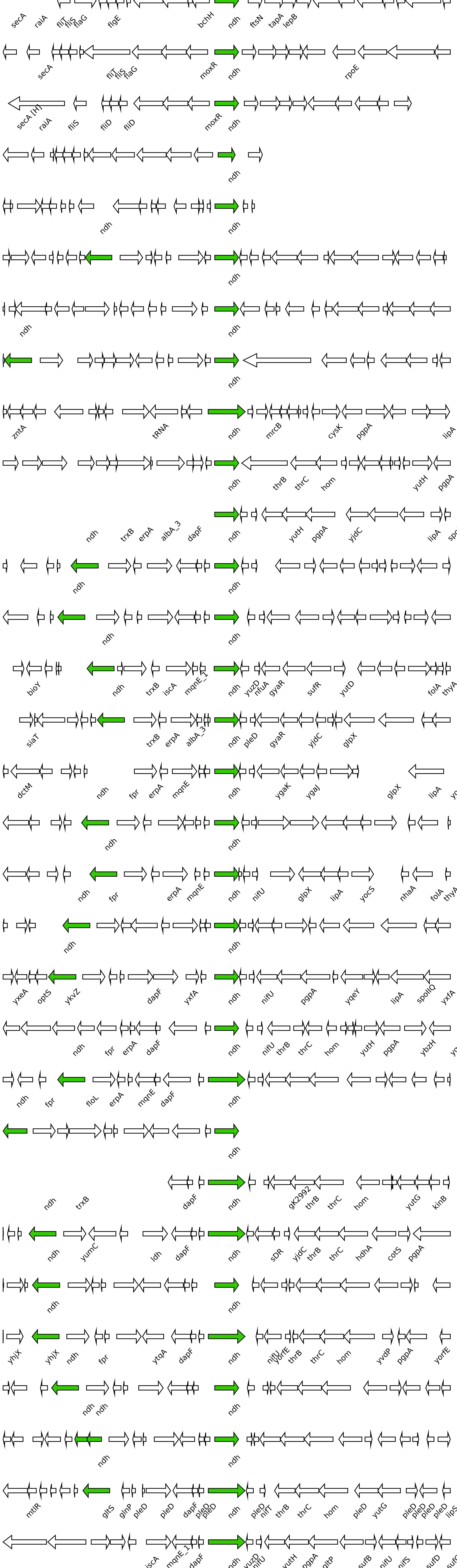

Bacillus lonarensis
SDB94236.1 (1464122)
FMYM01000004.1 (genomic DNA)

Bacillus pseudofirmus
OLO28580.1 (79885)
MSLR01000020.1 (genomic)

Bacillus sp FJAT 18017
ALC89488.1 (1705566)
CP012602.1 (chromosome)

Anoxybacillus flavithermus WK1
ACJ34766.1 (491915)
CP000922.1 (chromosome)

Anoxybacillus amylolyticus
ANB60994.1 (294699)
CP015438.1 (chromosome)

Geobacillus kaustophilus HTA426
BAD77244.1 (235909)
BA000043.1 (genomic DNA)

Geobacillus sp WCH70
ACS25571.1 (471223)
CP001638.1 (chromosome)

Bacillus acanthi
QBBX01000008.1_104 (2171554)
QBBX01000008.1 ()

Bacillus sp OV322
SFC29160.1 (1882764)
FOMA01000002.1 (genomic DNA)

Bacillus cavernae
RUQ25934.1 (1674310)
RYZZ01000037.1 (genomic DNA)

Bacillus butanolivorans
KON67104.1 (421767)
LGYA01000005.1 (genomic DNA)

Bacillus loiseleuriae
KMY52451.1 (1679170)
LFZW01000001.1 (genomic DNA)

Bacillus sp V47 23a
RFU70132.1 (2303992)
QVTE01000017.1 (genomic DNA)

Bacillus psychrosaccharolyticus ATCC 23296
AJTN02000200.1_52 (1174504)
AJTN02000200.1 ()

Bacillus massilioanorexius AP8
CAPG01000076.1_46 (1211814)
CAPG01000076.1 ()

Bacillus testis
LN851186.1_3252 (1622072)
LN851186.1 ()

Bacillus salsus
SDP92891.1 (930152)
FNJU01000013.1 (genomic DNA)

Bacillaceae bacterium UBA7792
DLCS01000169.1_6 (1951479)
DLCS01000169.1 ()

Bacillus tuaregi
LT629731.1_721 (1816695)
LT629731.1 ()

Bacillus sp Y1
AYA77704.1 (352858)
CP030028.1 (chromosome)

Bacillus firmus
KML38789.1 (1399)
LDWQ01000052.1 (genomic DNA)

Bacillus sp V3 13
PLR79347.1 (2053728)
PGUZ01000002.1 (genomic DNA)

Bacillus methanolicus MGA3
EIJ77924.1 (796606)
ADWW01000005.1 (genomic DNA)

Bacillus bacterium
HAQ06338.1 (2053512)
DMSR01000064.1 (genomic DNA)

Bacillus jeotgali
CP025025.1_3856 (129985)
CP025025.1 ()

Bacillus campisalis
KKK39334.1 (1408103)
LAYY01000003.1 (genomic DNA)

Bacillus sp 1NLA3E
AGK55472.1 (666686)
CP005586.1 (chromosome)

Bacillus massilionigeriensis
LT630337.1_3331 (1805475)
LT630337.1 ()

Bacillus sp UNCCL13
SFB12774.1 (1502772)
FOJZ01000006.1 (genomic DNA)

Bacillus sp X1 2014
AIM16065.1 (1565991)
CP008855.1 (chromosome)

Bacillus sp AFS006103
PEQ84105.1 (2033483)
NTXX01000081.1 (genomic DNA)

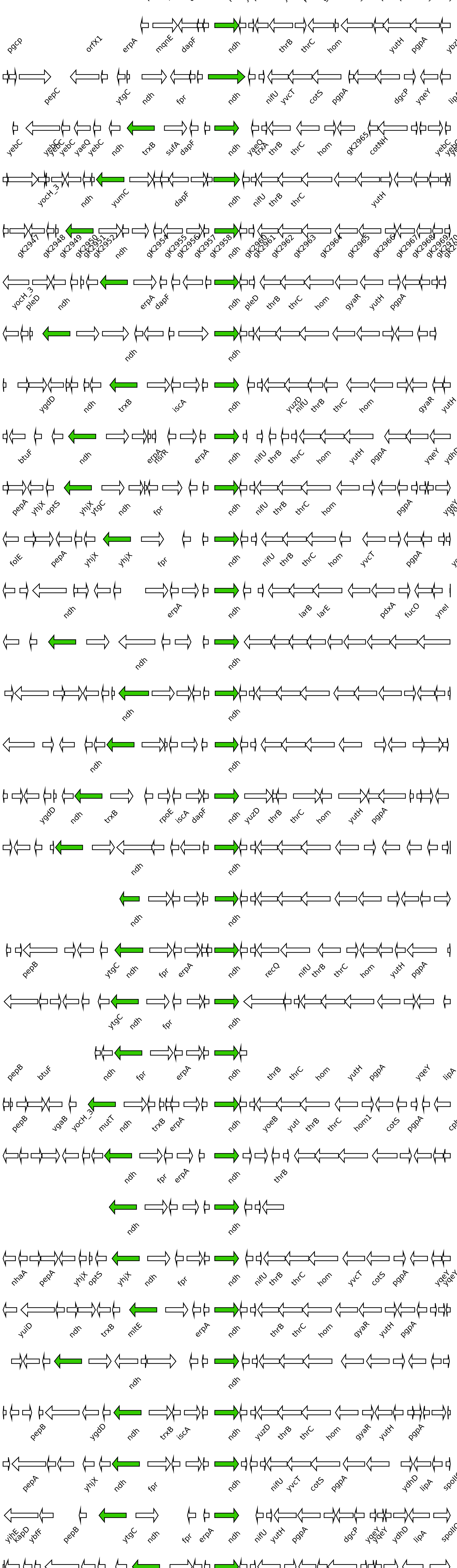

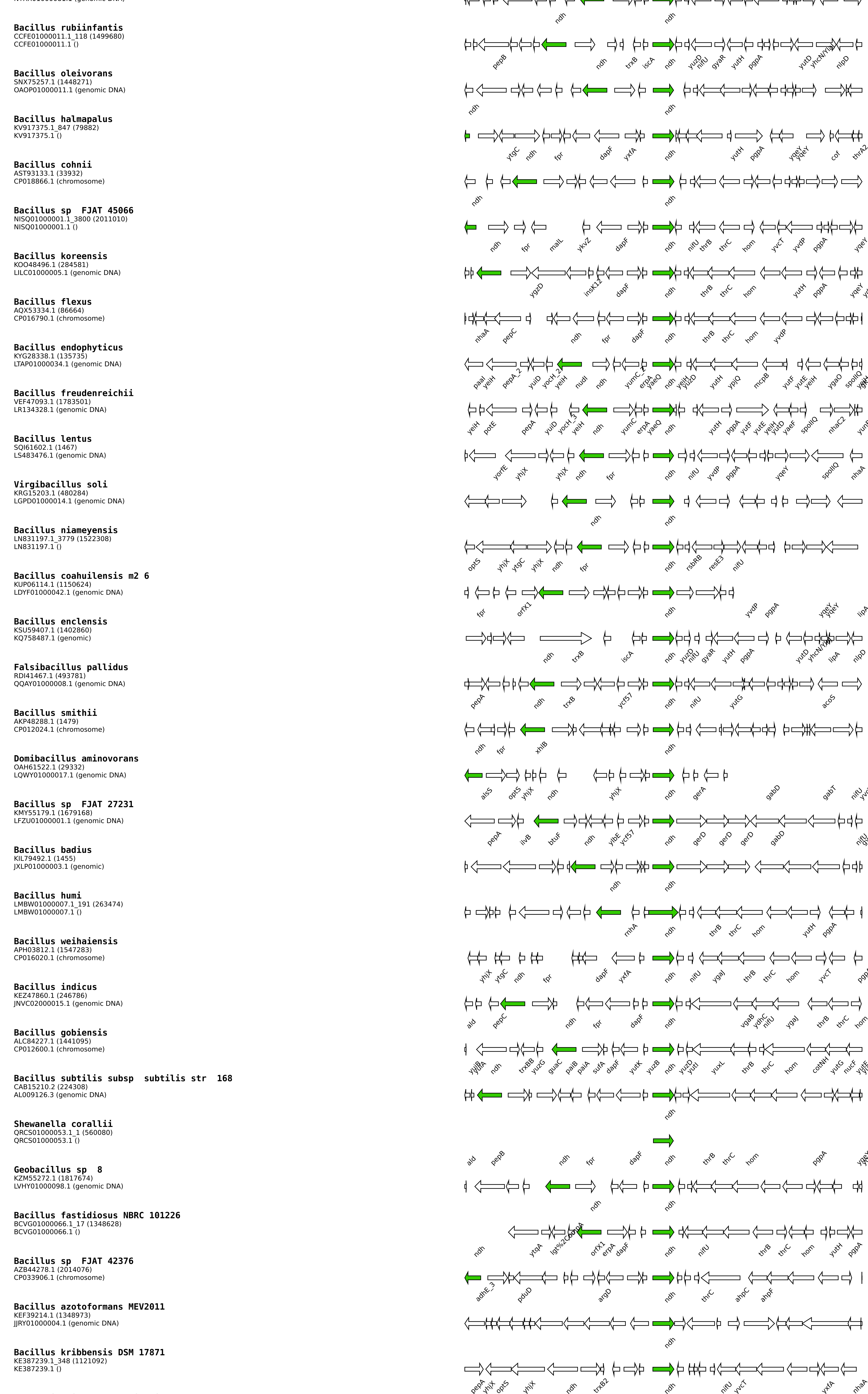
Bacillus rubiinfantis
CCFE01000011.1_118 (1499680)
CCFE01000011.1 ()
Bacillus oleivorans
SNX75257.1 (1448271)
OAOP01000011.1 (genomic DNA)
Bacillus halmapalus
KV917375.1_847 (79882)
KV917375.1 ()
Bacillus cohnii
AST93133.1 (33932)
CP018866.1 (chromosome)
Bacillus sp FJAT 45066
NISQ01000001.1_3800 (2011010)
NISQ01000001.1 ()
Bacillus koreensis
KOO48496.1 (284581)
LILC01000005.1 (genomic DNA)
Bacillus flexus
AQX53334.1 (86664)
CP016790.1 (chromosome)
Bacillus endophyticus
KYG28338.1 (135735)
LTAP01000034.1 (genomic DNA)
Bacillus freudenreichii
VEF47093.1 (1783501)
LR134328.1 (genomic DNA)
Bacillus lentus
SQI61602.1 (1467)
LS483476.1 (genomic DNA)
Virgibacillus soli
KRG15203.1 (480284)
LGPD01000014.1 (genomic DNA)
Bacillus niameyensis
LN831197.1_3779 (1522308)
LN831197.1 ()
Bacillus coahuilensis m2 6
KUP06114.1 (1150624)
LDYF01000042.1 (genomic DNA)
Bacillus enclensis
KSU59407.1 (1402860)
KQ758487.1 (genomic)
Falsibacillus pallidus
RDI41467.1 (493781)
QQAY01000008.1 (genomic DNA)
Bacillus smithii
AKP48288.1 (1479)
CP012024.1 (chromosome)
Domibacillus aminovorans
OAH61522.1 (29332)
LQWY01000017.1 (genomic DNA)
Bacillus sp FJAT 27231
KMY55179.1 (1679168)
LFZU01000001.1 (genomic DNA)
Bacillus badius
KIL79492.1 (1455)
JXLP01000003.1 (genomic)
Bacillus humi
LMBW01000007.1_191 (263474)
LMBW01000007.1 ()
Bacillus weihaiensis
APH03812.1 (1547283)
CP016020.1 (chromosome)
Bacillus indicus
KEZ47860.1 (246786)
JNVC02000015.1 (genomic DNA)
Bacillus gobiensis
ALC84227.1 (1441095)
CP012600.1 (chromosome)
Bacillus subtilis subsp subtilis str 168
CAB15210.2 (224308)
AL009126.3 (genomic DNA)
Shewanella corallii
QRCS01000053.1_1 (560080)
QRCS01000053.1 ()
Geobacillus sp 8
KZM55272.1 (1817674)
LVHY01000098.1 (genomic DNA)
Bacillus fastidiosus NBRC 101226
BCVG01000066.1_17 (1348628)
BCVG01000066.1 ()
Bacillus sp FJAT 42376
AZB44278.1 (2014076)
CP033906.1 (chromosome)
Bacillus azotoformans MEV2011
KEF39214.1 (1348973)
JJRY01000004.1 (genomic DNA)
Bacillus kribbensis DSM 17871
KE387239.1_348 (1121092)
KE387239.1 ()

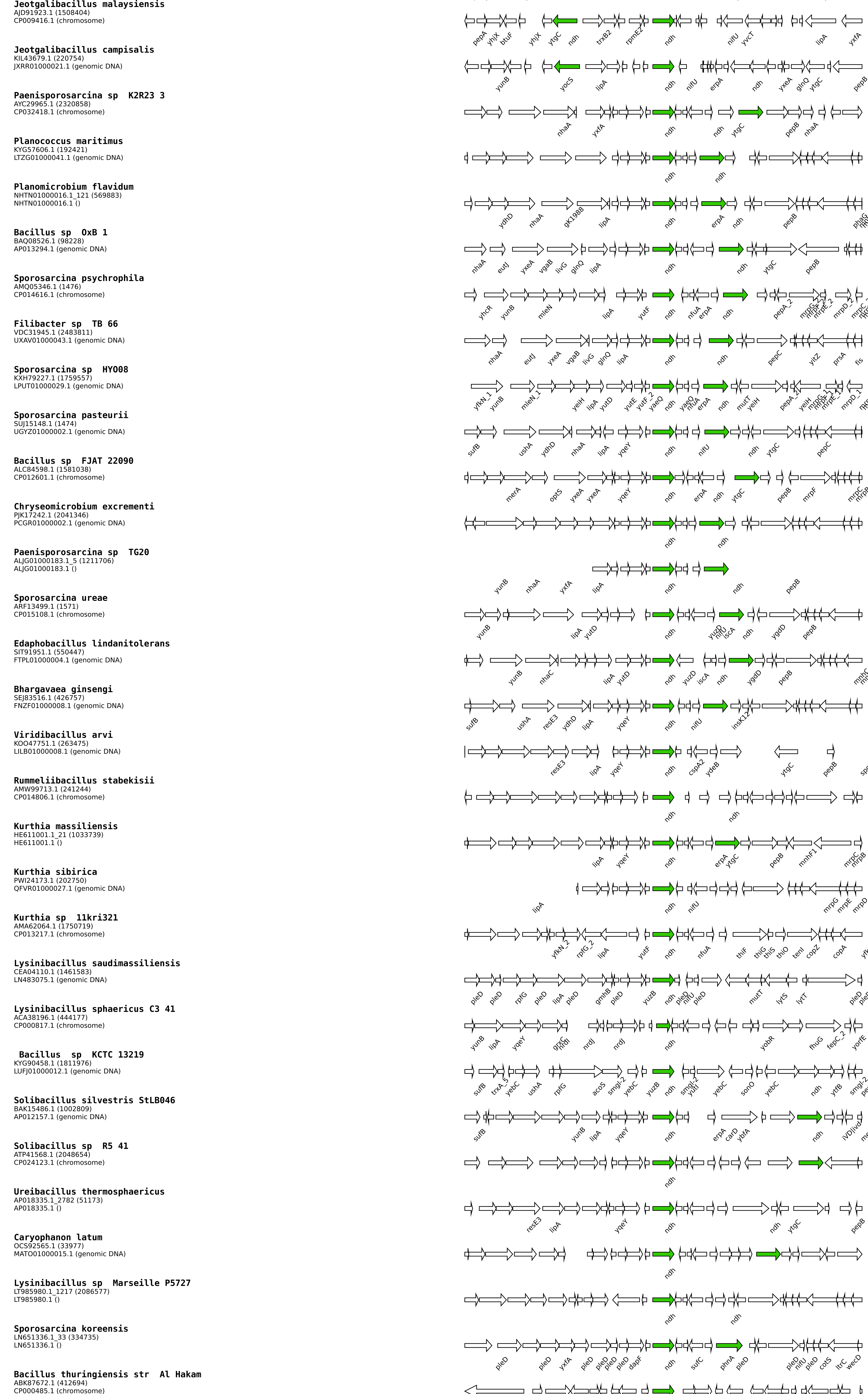
Jeotgalibacillus malaysiensis
AJD91923.1 (1508404)
CP009416.1 (chromosome)
Jeotgalibacillus campisalis
KIL43679.1 (220754)
JXRR01000021.1 (genomic DNA)
Paenisporosarcina sp K2R23 3
AYC29965.1 (2320858)
CP032418.1 (chromosome)
Planococcus maritimus
KYG57606.1 (192421)
LTZG01000041.1 (genomic DNA)
Planomicrobium flavidum
NHTN01000016.1_121 (569883)
NHTN01000016.1 ()
Bacillus sp OxB 1
BAQ08526.1 (98228)
AP013294.1 (genomic DNA)
Sporosarcina psychrophila
AMQ05346.1 (1476)
CP014616.1 (chromosome)
Filibacter sp TB 66
VDC31945.1 (2483811)
UXAV01000043.1 (genomic DNA)
Sporosarcina sp HYO08
KXH79227.1 (1759557)
LPUT01000029.1 (genomic DNA)
Sporosarcina pasteurii
SUJ15148.1 (1474)
UGYZ01000002.1 (genomic DNA)
Bacillus sp FJAT 22090
ALC84598.1 (1581038)
CP012601.1 (chromosome)
Chryseomicrobium excrementi
PJK17242.1 (2041346)
PCGR01000002.1 (genomic DNA)
Paenisporosarcina sp TG20
ALJG01000183.1_5 (1211706)
ALJG01000183.1 ()
Sporosarcina ureae
ARF13499.1 (1571)
CP015108.1 (chromosome)
Edaphobacillus lindanitolerans
SIT91951.1 (550447)
FTPL01000004.1 (genomic DNA)
Bhargavaea ginsengi
SEJ83516.1 (426757)
FNZF01000008.1 (genomic DNA)
Viridibacillus arvi
KOO47751.1 (263475)
LILB01000008.1 (genomic DNA)
Rummeliibacillus stabekisii
AMW99713.1 (241244)
CP014806.1 (chromosome)
Kurthia massiliensis
HE611001.1_21 (1033739)
HE611001.1 ()
Kurthia sibirica
PWI24173.1 (202750)
QFVR01000027.1 (genomic DNA)
Kurthia sp 11kri321
AMA62064.1 (1750719)
CP013217.1 (chromosome)
Lysinibacillus saudimassiliensis
CEA04110.1 (1461583)
LN483075.1 (genomic DNA)
Lysinibacillus sphaericus C3 41
ACA38196.1 (444177)
CP000817.1 (chromosome)
Bacillus sp KCTC 13219
KYG90458.1 (1811976)
LUFJ01000012.1 (genomic DNA)
Solibacillus silvestris StLB046
BAK15486.1 (1002809)
AP012157.1 (genomic DNA)
Solibacillus sp R5 41
ATP41568.1 (2048654)
CP024123.1 (chromosome)
Ureibacillus thermosphaericus
AP018335.1_2782 (51173)
AP018335.1 ()
Caryophanon latum
OCS92565.1 (33977)
MATO01000015.1 (genomic DNA)
Lysinibacillus sp Marseille P5727
LT985980.1_1217 (2086577)
LT985980.1 ()
Sporosarcina koreensis
LN651336.1_33 (334735)
LN651336.1 ()
Bacillus thuringiensis str Al Hakam
ABK87672.1 (412694)
CP000485.1 (chromosome)

Bacillus sp SYSU K30001
RIAV01000027.1_8 (2486280)
RIAV01000027.1 ()

Bacillus sp OV194
SFD50545.1 (1881065)
FOML01000001.1 (genomic DNA)

Fictibacillus arsenicus
ANX13414.1 (255247)
CP016761.1 (chromosome)

Fictibacillus aquaticus
OYD56025.1 (2021314)
NOII01000069.1 (genomic DNA)

Bacillus sp AFS001701
PET70066.1 (2033480)
NTZO01000092.1 (genomic DNA)

Bacillus thermoamylovorans
CEE02531.1 (35841)
CCRF01000079.1 (genomic DNA)

Bacillus andreraoultii
CCFJ01000045.1_12 (1499685)
CCFJ01000045.1 ()

Bacillus kwashiorkori
CTDX01000015.1_445 (1522318)
CTDX01000015.1 ()

Salsuginibacillus kocurii DSM 18087
KB898623.1_538 (1123239)
KB898623.1 ()

Salsuginibacillus halophilus
PSL44458.1 (517424)
PYAV01000008.1 (genomic DNA)

Geomicrobium sp JCM 19037
GAK05272.1 (1460634)
BAWZ01000033.1 (genomic DNA)

Ruegeria sp NKC1 1
AXF56137.1 (2099786)
CP031092.1 (chromosome)

Natribacillus halophilus
SDJ18574.1 (549003)
FNEN01000019.1 (genomic DNA)

Marinococcus halophilus
OZT79428.1 (1371)
NPFA01000013.1 (genomic DNA)

Alteribacillus iranensis
SFE94819.1 (930128)
FONT01000006.1 (genomic DNA)

Alteribacillus bidgolensis
KZ614149.1_2236 (930129)
KZ614149.1 ()

Bacillus sp FJAT 44742
KZ454936.1_2858 (2014005)
KZ454936.1 ()

Salibacterium halotolerans
SFP51253.1 (1884432)
FOXD01000006.1 (genomic DNA)

Sinobaca qinghaiensis
RKD68829.1 (342944)
RAPK01000012.1 (genomic DNA)

Bacillaceae bacterium EAG3
PQD95180.1 (2064768)
PKOZ01000005.1 (genomic DNA)

Bacillus solimangrovi
OEH93982.1 (1305675)
MJEH01000006.1 (genomic DNA)

Caldibacillus debilis
KYD18621.1 (301148)
LQYT01000049.1 (genomic DNA)

Pueribacillus theae
PWA11991.1 (2171751)
QCZG01000013.1 (genomic DNA)

Bacillales bacterium UBA6769
DKGN01000039.1_6 (1950335)
DKGN01000039.1 ()

Salinicoccus halodurans
AKG73295.1 (407035)
CP011366.1 (chromosome)

Salinicoccus albus DSM 19776
ARQJ01000029.1_106 (1123230)
ARQJ01000029.1 ()

Auricoccus indicus
AQL56621.1 (1849491)
CP019573.1 (chromosome)

Macrococcus caseolyticus JCSC5402
BAH17271.1 (458233)
AP009484.1 (genomic DNA)

Staphylococcus sp
HAL10496.1 (29387)
DMKR01000052.1 (genomic DNA)

Staphylococcus massiliensis CCUG 55927
JH815593.1_975 (1189613)
JH815593.1 ()

[illegible]

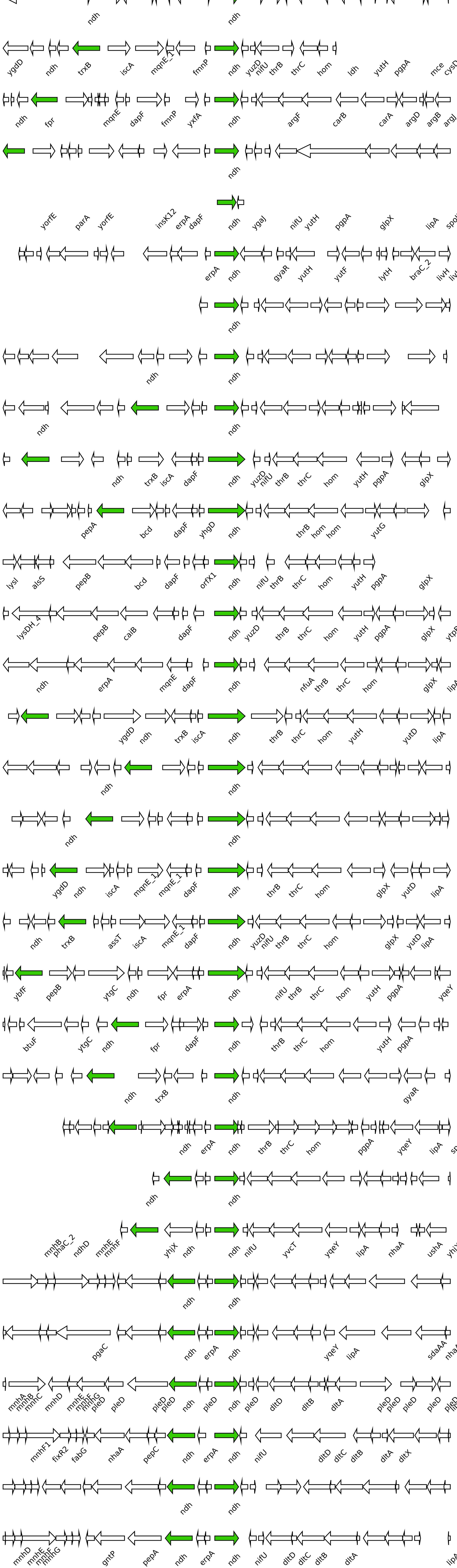

Staphylococcus hyicus
AJC96648.1 (1284)
CP008747.1 (chromosome)

Staphylococcus carnosus subsp carnosus TM300
CAL27457.1 (396513)
AM295250.1 (genomic DNA)

Staphylococcus aureus subsp aureus Mu50
BAB57100.1 (158878)
BA000017.4 (genomic DNA)

Staphylococcus cohnii subsp urealyticus
OJT35290.1 (94138)
MPPU01000001.1 (genomic DNA)

Staphylococcus argensis
POA09567.1 (1607738)
PPPX01000001.1 (genomic DNA)

Staphylococcus auricularis
LLER01000002.1_76 (29379)
LLER01000002.1 ()

Tuberibacillus sp Marseille P3662
FXBS01000006.1_80 (1965358)
FXBS01000006.1 ()

Paenibacillus pini JCM 16418
GAF08615.1 (1236976)
BAVZ01000007.1 (genomic DNA)

Paenibacillus sp IHBB 10380
AJS57412.1 (1566358)
CP010976.1 (chromosome)

Paenibacillus durus
AIQ14325.1 (44251)
CP009288.1 (chromosome)

Paenibacillus yonginensis
ANS75632.1 (1462996)
CP014167.1 (chromosome)

Paenibacillus lentus
AZK46262.1 (1338368)
CP034248.1 (chromosome)

Paenibacillus sp 453mf
SFS91868.1 (1761874)
FPAC01000007.1 (genomic DNA)

Paenibacillus polymyxa E681
ADM71703.1 (349520)
CP000154.2 (chromosome)

Paenibacillus lactis
HAF97145.1 (228574)
DMBX01000004.1 (genomic DNA)

Paenibacillus popilliae ATCC 14706
GAC41972.1 (1212764)
BALG01000056.1 (genomic DNA)

Paenibacillus assamensis DSM 18201
AULU01000010.1_88 (1122916)
AULU01000010.1 ()

Paenibacillus xerothermodurans
PZE21525.1 (1977292)
NHRJ02000003.1 (genomic DNA)

Paenibacillus beijingensis
AJY77779.1 (1126833)
CP011058.1 (chromosome)

Paenibacillaceae bacterium GAS479
SDT19420.1 (1882832)
LT629764.1 (genomic DNA)

Paenibacillus sp 32O W
ALS26760.1 (1695218)
CP013653.1 (chromosome)

Paenibacillus sp FJAT 26967
LMXH01000001.1_688 (1729690)
LMXH01000001.1 ()

Paenibacillus larvae subsp larvae B 3650
PCK69557.1 (741161)
ADZY03000325.1 (genomic DNA)

Paenibacillus swuensis
ANE45045.1 (1178515)
CP011388.1 (chromosome)

Paenibacillus darwinianus
EXX89216.1 (1380763)
KK082133.1 (genomic)

Paenibacillus curdlanolyticus YK9
EFM08733.1 (717606)
AEDD01000014.1 (genomic DNA)

Cohnella panacarvi Gsoil 349
KI911563.1_3749 (979226)
KI911563.1 ()

Cohnella sp 18JY8 7
AYQ71368.1 (2480923)
CP033433.1 (chromosome)

Cohnella laeviribosi DSM 21336
KB899819.1_50 (1121346)
KB899819.1 ()

Cohnella thermotolerans DSM 17683
AUCP01000073.1_6 (1121347)
AUCP01000073.1 ()

Gorillibacterium timonense
LN881723.1_864 (1689269)
LN881723.1 ()

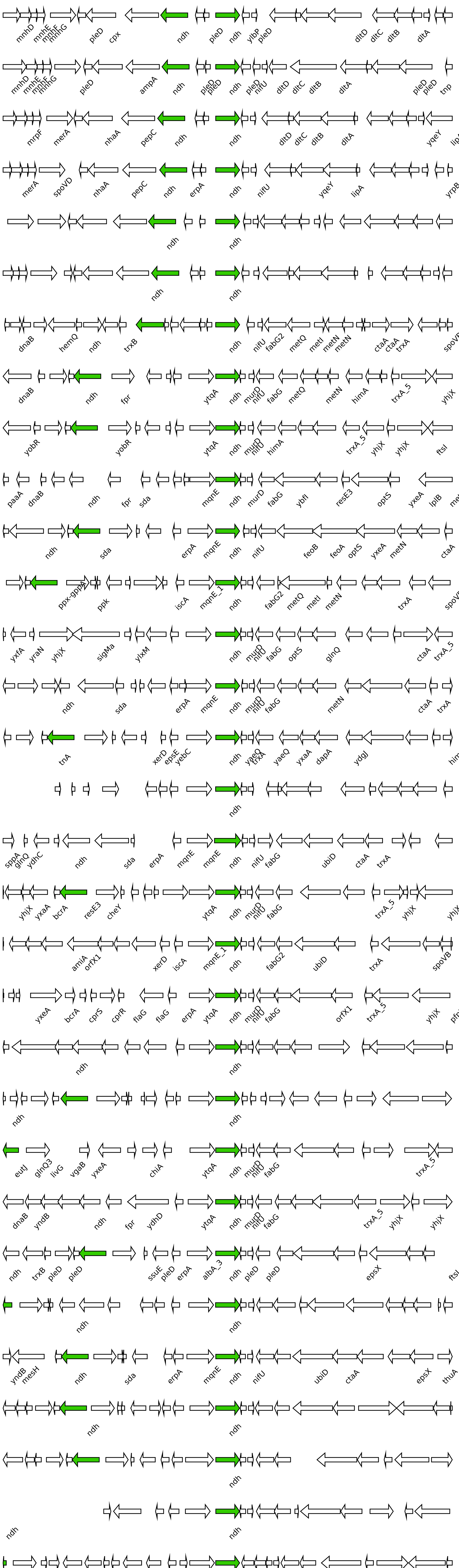

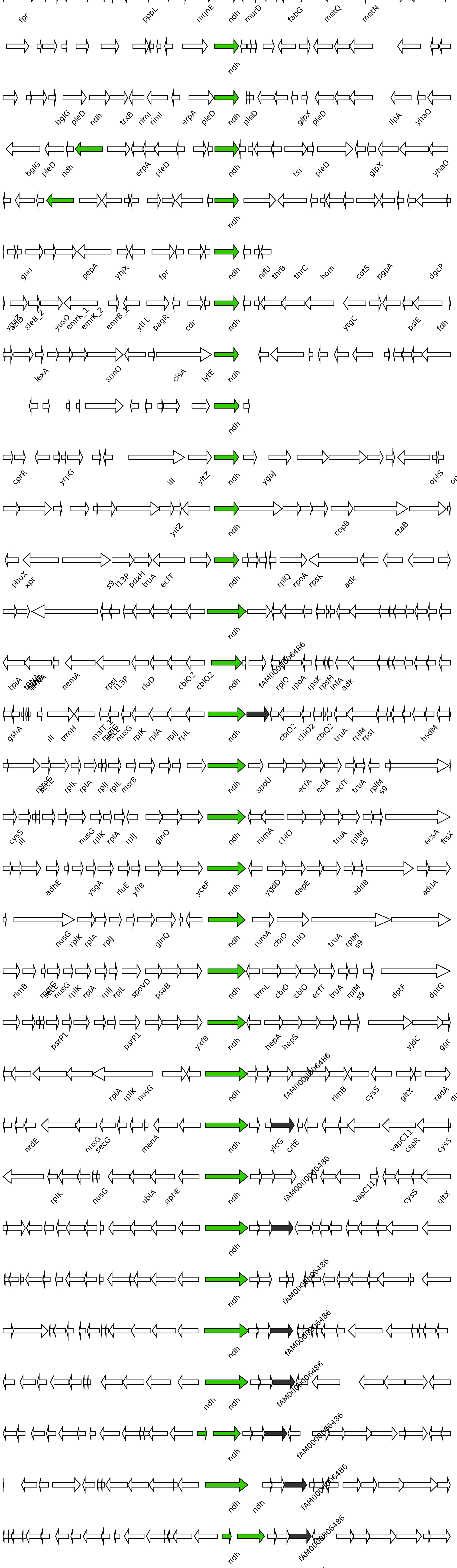

Paenibacillus bovis
ANF98186.1 (1616788)
CP013023.1 (chromosome)

Saccharibacillus kuerlensis DSM 22868
KB899305.1_17 (1123226)
KB899305.1 ()

Exiguobacterium sibiricum 255 15
ACB61771.1 (262543)
CP001022.1 (chromosome)

Exiguobacterium sp AT1b
ACQ69710.1 (360911)
CP001615.1 (chromosome)

Bacillaceae bacterium UBA4111
DFXJ01000275.1_33 (1951478)
DFXJ01000275.1 ()

Bacillus circulans
KLV26298.1 (1397)
LDPH01000009.1 (genomic DNA)

Aneurinibacillus soli
BAU28466.1 (1500254)
AP017312.1 (genomic DNA)

Aneurinibacillus thermoaerophilus
SDH78896.1 (143495)
FNDE01000060.1 (genomic DNA)

Aneurinibacillus terranovensis DSM 18919
KE387172.1_71 (1121002)
KE387172.1 ()

Acidibacillus ferrooxidans
KUO95959.1 (1765683)
LPVJ01000030.1 (genomic DNA)

Acidibacillus sulfuroxidans
PWI58554.1 (1765684)
MPDK01000003.1 (genomic DNA)

Convivina intestini
PVY83441.1 (1505726)
QEKT01000007.1 (genomic DNA)

Leuconostoc fallax KCTC 3537
AEIZ01000020.1_34 (907931)
AEIZ01000020.1 ()

Leuconostoc mesenteroides subsp mesenteroides ATCC 8293
ABJ61355.1 (203120)
CP000414.1 (chromosome)

Weissella oryzae SG25
GAK29937.1 (1329250)
DF820484.1 (genomic DNA)

Weissella ceti
AIG65986.1 (759620)
CP007588.1 (chromosome)

Weissella viridescens
KRN46482.1 (1629)
JQBM01000002.1 (genomic)

Weissella soli
AOT55830.1 (155866)
CP017326.1 (chromosome)

Weissella confusa
KRN22650.1 (1583)
JQAY01000008.1 (genomic)

Weissella paramesenteroides
ATF40617.1 (1249)
CP023501.1 (chromosome)

Oenococcus kitaharae DSM 17330
EHN58148.1 (1045004)
CM001398.1 (chromosome)

Lactobacillus amylotrophicus DSM 20534
KRK37458.1 (1423722)
AZCV01000005.1 (genomic)

Lactobacillus paracasei ATCC 334
ABJ71041.1 (321967)
CP000423.1 (chromosome)

Lactobacillus brantae DSM 23927
KRM71483.1 (1423727)
AYZQ01000004.1 (genomic)

Lactobacillus saniviri JCM 17471 DSM 24301
BBBX01000014.1_23 (1293598)
BBBX01000014.1 ()

Lactobacillus nasuensis JCM 17158
BBAM01000002.1_46 (1291734)
BBAM01000002.1 ()

Lactobacillus sp 33 1
RHOG01000021.1_33 (2486015)
RHOG01000021.1 ()

Lactobacillus camelliae DSM 22697 JCM 13995
BBAJ01000010.1_47 (1423730)
BBAJ01000010.1 ()

Lactobacillus sp 143 4 a
RHOH01000006.1_50 (2486014)
RHOH01000006.1 ()

Lactobacillus camelliae DSM 22697 JCM 13995
BBAJ01000010.1_46 (1423730)
BBAJ01000010.1 ()

Lactobacillus manihotivorans DSM 13343 JCM 12514

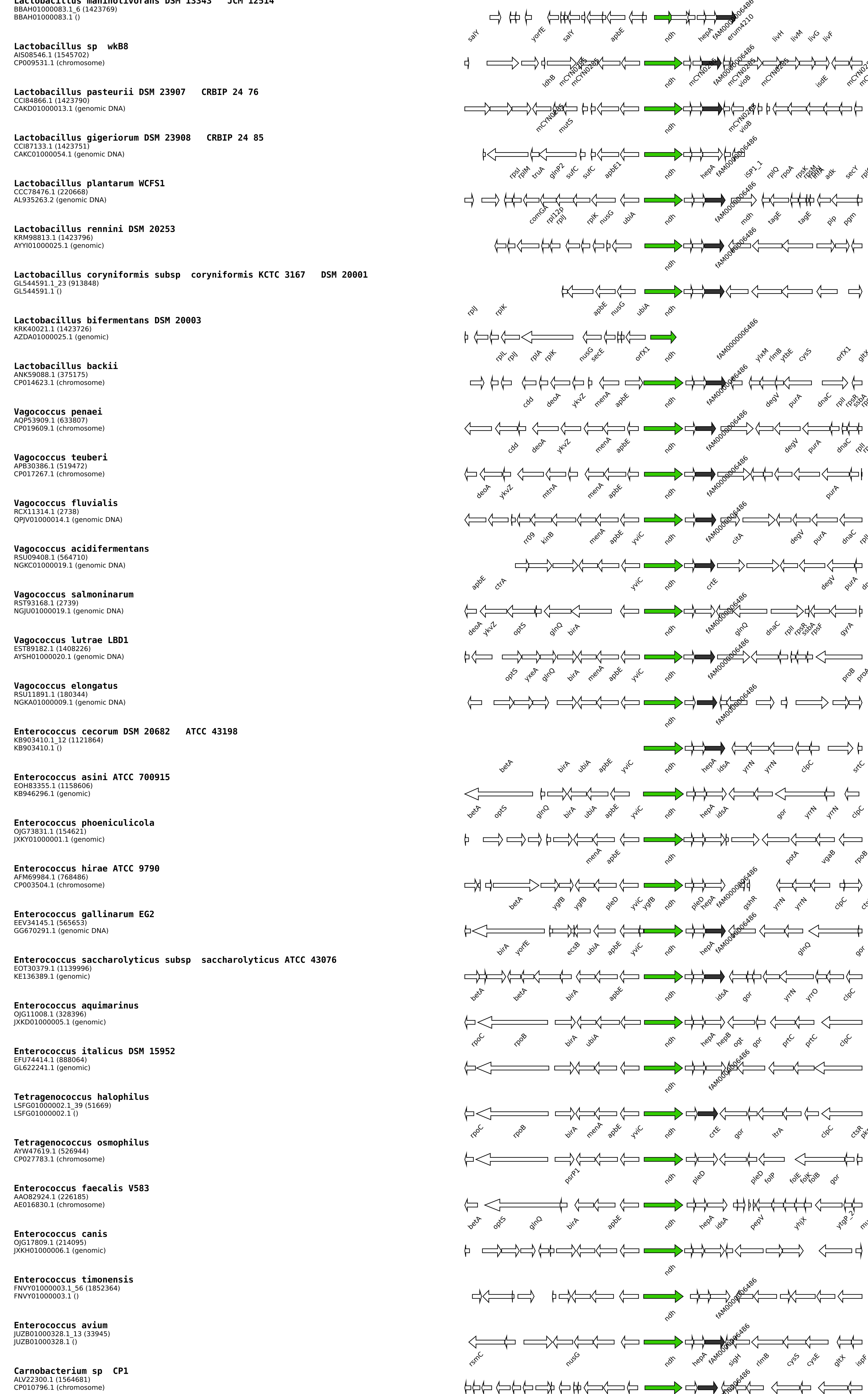
Lactobacillus manihotivorans DSM 13343 JCM 12514
BBAH01000083.1_6 (1423769)
BBAH01000083.1 ()
Lactobacillus sp wkB8
AIS08546.1 (1545702)
CP009531.1 (chromosome)
Lactobacillus pasteurii DSM 23907 CRBIP 24 76
CCI84866.1 (1423790)
CAKD01000013.1 (genomic DNA)
Lactobacillus gigeriorum DSM 23908 CRBIP 24 85
CCI87133.1 (1423751)
CAKC01000054.1 (genomic DNA)
Lactobacillus plantarum WCFS1
CCC78476.1 (220668)
AL935263.2 (genomic DNA)
Lactobacillus rennini DSM 20253
KRM98813.1 (1423796)
AYYI01000025.1 (genomic)
Lactobacillus coryniformis subsp coryniformis KCTC 3167 DSM 20001
GL544591.1_23 (913848)
GL544591.1 ()
Lactobacillus bifermentans DSM 20003
KRK40021.1 (1423726)
AZDA01000025.1 (genomic)
Lactobacillus backii
ANK59088.1 (375175)
CP014623.1 (chromosome)
Vagococcus penaei
AQP53909.1 (633807)
CP019609.1 (chromosome)
Vagococcus teuberi
APB30386.1 (519472)
CP017267.1 (chromosome)
Vagococcus fluvialis
RCX11314.1 (2738)
QPJV01000014.1 (genomic DNA)
Vagococcus acidifermentans
RSU09408.1 (564710)
NGKC01000019.1 (genomic DNA)
Vagococcus salmoninarum
RST93168.1 (2739)
NGJU01000019.1 (genomic DNA)
Vagococcus lutrae LBD1
EST89182.1 (1408226)
AYSH01000020.1 (genomic DNA)
Vagococcus elongatus
RSU11891.1 (180344)
NGKA01000009.1 (genomic DNA)
Enterococcus cecorum DSM 20682 ATCC 43198
KB903410.1_12 (1121864)
KB903410.1 ()
Enterococcus asini ATCC 700915
EOH83355.1 (1158606)
KB946296.1 (genomic)
Enterococcus phoeniculicola
OJG73831.1 (154621)
JXKY01000001.1 (genomic)
Enterococcus hirae ATCC 9790
AFM69984.1 (768486)
CP003504.1 (chromosome)
Enterococcus gallinarum EG2
EEV34145.1 (565653)
GG670291.1 (genomic DNA)
Enterococcus saccharolyticus subsp saccharolyticus ATCC 43076
EOT30379.1 (1139996)
KE136389.1 (genomic)
Enterococcus aquimarinus
OJG11008.1 (328396)
JXKD01000005.1 (genomic)
Enterococcus italicus DSM 15952
EFU74414.1 (888064)
GL622241.1 (genomic)
Tetragenococcus halophilus
LSFG01000002.1_39 (51669)
LSFG01000002.1 ()
Tetragenococcus osmophilus
AYW47619.1 (526944)
CP027783.1 (chromosome)
Enterococcus faecalis V583
AAO82924.1 (226185)
AE016830.1 (chromosome)
Enterococcus canis
OJG17809.1 (214095)
JXKH01000006.1 (genomic)
Enterococcus timonensis
FNVY01000003.1_56 (1852364)
FNVY01000003.1 ()
Enterococcus avium
JUZB01000328.1_13 (33945)
JUZB01000328.1 ()
Carnobacterium sp CP1
ALV22300.1 (1564681)
CP010796.1 (chromosome)

Carnobacterium inhibens subsp gilichinskyi
AGY82699.1 (1266845)
CP006812.1 (chromosome)

Alkalibacterium gilvum
SEI96117.1 (1130080)
FNYW01000038.1 (genomic DNA)

Lacticigenium naphtae DSM 19658
AUEG01000005.1_50 (1122143)
AUEG01000005.1 ()

Marinilactibacillus sp 15R
API87935.1 (1911586)
CP017761.1 (chromosome)

Pisciglobus halotolerans
SFH57752.1 (745365)
FOQE01000004.1 (genomic DNA)

Carnobacterium divergens
ANZ98969.1 (2748)
CP016843.1 (chromosome)

Carnobacterium gallinarum DSM 4847
JQLU01000005.1_1435 (1449338)
JQLU01000005.1 ()

Desemzia incerta
SFQ40180.1 (82801)
FOXW01000007.1 (genomic DNA)

Trichococcus collinsii
CZR01070.1 (157076)
FJNA01000002.1 (genomic DNA)

Jeotgalibaca sp H21T32
AZP04192.1 (2496265)
CP034465.1 (chromosome)

Jeotgalibaca dankookensis
BBYN01000039.1_4 (708126)
BBYN01000039.1 ()

Atopobacter phocae ATCC BAA 285
JAGN01000008.1_16 (1336234)
JAGN01000008.1 ()

Abiotrophia sp HMSC24B09
OFS29120.1 (1581061)
KV822203.1 (genomic)

Globicatella sp HMSC072A10
OFK60141.1 (1739315)
KV805690.1 (genomic)

Streptococcus phocae subsp salmonis
KGR73297.1 (1000562)
JSAP01000013.1 (genomic DNA)

Streptococcus suis 98HAH33
ABP92926.1 (391296)
CP000408.1 (chromosome)

Streptococcus sp UBA6071
DIXV01000029.1_17 (1947565)
DIXV01000029.1 ()

Streptococcus pluranimalium
AUW96634.1 (82348)
CP025536.1 (chromosome)

Streptococcus marimammalium DSM 18627
KB904339.1_225 (1123306)
KB904339.1 ()

Aerococcaceae bacterium
HBY90562.1 (2053495)
DOTN01000063.1 (genomic DNA)

Eremococcus coleocola DSM 15696
AUAT01000037.1_5 (1121871)
AUAT01000037.1 ()

Ignavigranum ruoffiae
SEQ18788.1 (89093)
FOEN01000006.1 (genomic DNA)

Isobaculum melis
SER84118.1 (142588)
FOHA01000007.1 (genomic DNA)

Listeria weihenstephanensis
AQY49883.1 (1006155)
CP011102.1 (chromosome)

Listeria monocytogenes EGD e
CAD00716.1 (169963)
AL591824.1 (genomic DNA)

Bacillus andreraoultii
CCFJ01000056.1_16 (1499685)
CCFJ01000056.1 ()

Bacillus thermoamylovorans
CEE01494.1 (35841)
CCRF01000047.1 (genomic DNA)

Bacillus kwashiorkori
CTDX01000015.1_119 (1522318)
CTDX01000015.1 ()

Saliterribacillus persicus
RCW71899.1 (930114)
QPJJ01000005.1 (genomic DNA)

Paraliobacillus ryukyuensis
RBO92315.1 (200904)
QNRI01000014.1 (genomic DNA)

Paraliobacillus quinghaiensis

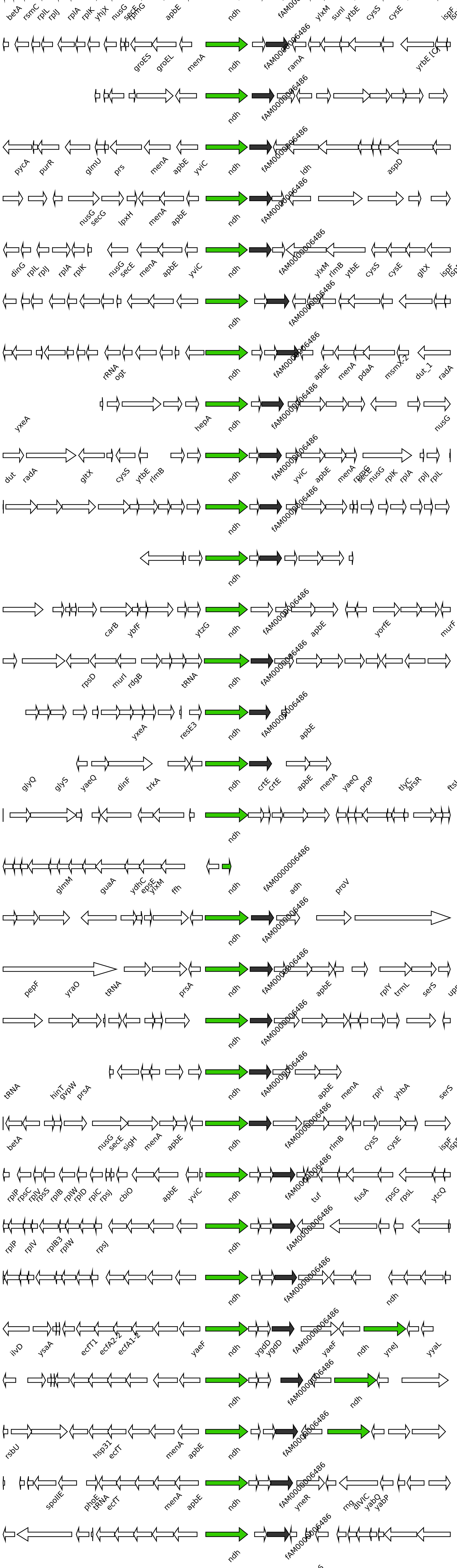

Paraliobacillus quinghaiensis
QJSH01000029.1_12 (470815)
QJSH01000029.1 ()

Paraliobacillus sp X 1125
CP029797.1_92 (2213194)
CP029797.1 ()

Bacillus circulans
KLV22524.1 (1397)
LDPH01000030.1 (genomic DNA)

Halolactibacillus alkaliphilus
SFP06824.1 (442899)
FOWN01000041.1 (genomic DNA)

Streptohalobacillus salinus
PXW88452.1 (621096)
QJJR01000012.1 (genomic DNA)

Amphibacillus jilinensis Y1
AMWI01000057.1_22 (562743)
AMWI01000057.1 ()

Amphibacillus sediminis NBRC 103570
BCQW01000012.1_37 (1220586)
BCQW01000012.1 ()

Amphibacillus marinus
SEO91730.1 (872970)
FODJ01000017.1 (genomic DNA)

Amphibacillus xylanus NBRC 15112
BAM46203.1 (698758)
AP012050.1 (genomic DNA)

Bacillus niameyensis
LN831197.1_739 (1522308)
LN831197.1 ()

Oceanobacillus sojae
LT727812.1_1234 (582851)
LT727812.1 ()

Bacillus thermoamylovorans
CEE01499.1 (35841)
CCRF01000047.1 (genomic DNA)

Bacillus andreraoultii
CCFJ01000056.1_10 (1499685)
CCFJ01000056.1 ()

Bacillus kwashiorkori
CTDX01000015.1_124 (1522318)
CTDX01000015.1 ()

Sporolactobacillus nakayamae
SFG16043.1 (269670)
FOOY01000005.1 (genomic DNA)

Bacillus cellulosilyticus DSM 2522
ADU29188.1 (649639)
CP002394.1 (chromosome)

Bacillus tuaregi
LT629731.1_159 (1816695)
LT629731.1 ()

Granulicatella elegans ATCC 700633
EEW92284.1 (626369)
KI391971.1 (genomic)

Aerococcus christensenii
AMB92293.1 (87541)
CP014159.1 (chromosome)

Pelagirhabdus alkalitolerans
SDC55049.1 (1612202)
FMYI01000011.1 (genomic DNA)

Pilibacter termitis
SJZ72687.1 (263852)
FUXI01000012.1 (genomic DNA)

Lactococcus lactis subsp lactis Il1403
AAK04926.1 (272623)
AE005176.1 (chromosome)

Lactococcus termiticola
GBG97386.1 (2169526)
BFFO01000011.1 (genomic DNA)

Lactococcus fujiensis JCM 16395
BBAL01000004.1_94 (1291764)
BBAL01000004.1 ()

Lactococcus lactis subsp lactis Il1403
AAK04927.1 (272623)
AE005176.1 (chromosome)

Lactococcus garvieae Lg2
BAK60175.1 (420890)
AP009333.1 (genomic DNA)

Lactobacillaceae bacterium UBA5802
DIDK01000042.1_2 (1952192)
DIDK01000042.1 ()

Lactobacillus mixtipabuli
GAW99241.1 (1435342)
BCMF01000005.1 (genomic DNA)

Lactobacillus paracollinoides
ANZ60256.1 (240427)
CP014912.1 (chromosome)

Lactobacillus paracollinoides
ANZ60302.1 (240427)
CP014912.1 (chromosome)

Lactobacillus curieae
AQW21692.1 (1138822)
CP018906.1 (chromosome)

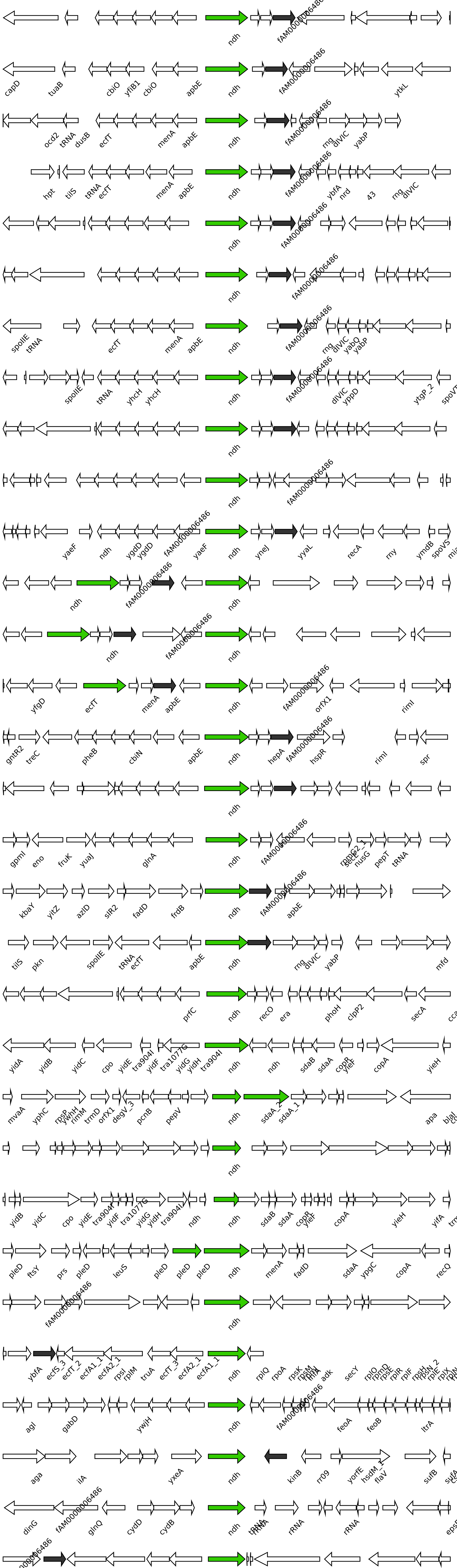

Lactobacillus buchneri ATCC 11577
EEI19756.1 (525318)
GG669719.1 (genomic)

Lactobacillus kisonensis F0435
EHO49554.1 (797516)
JH591051.1 (genomic)

Lactobacillus buchneri NRRL B 30929
AEB73867.1 (511437)
CP002652.1 (chromosome)

Lactobacillus kimchicus JCM 15530
BBFK01000001.1_622 (1302272)
BBFK01000001.1 ()

Firmicutes bacterium UBA6806
DKFC01000033.1_2 (1948099)
DKFC01000033.1 ()

Fusibacter sp 3D3
GAU77351.1 (1048380)
BDHH01000007.1 (genomic DNA)

Firmicutes bacterium UBA5201
DHVT01000020.1_46 (1948034)
DHVT01000020.1 ()

Clostridiales bacterium 38 18
OJV65694.1 (1895746)
MKTL01000004.1 (genomic DNA)

Dethiosulfatibacter aminovorans DSM 17477
SHI57491.1 (1121476)
FQZL01000005.1 (genomic DNA)

Alkalibaculum bacchi
RBP68977.1 (645887)
QNRX01000002.1 (genomic DNA)

Alkalibacter saccharofermentans DSM 14828
SHF10427.1 (1120975)
FQTU01000014.1 (genomic DNA)

Firmicutes bacterium HGW Firmicutes 9
PKM38834.1 (2013790)
PGZM01000009.1 (genomic DNA)

Spirochaeta sp UBA5339
DHQL01000013.1_152 (1947542)
DHQL01000013.1 ()

Sediminispirochaeta smaragdinae DSM 11293
ADK80725.1 (573413)
CP002116.1 (chromosome)

Marispirochaeta aestuarii
ORC35598.1 (1963862)
MWQY01000008.1 (genomic DNA)

Salinispira pacifica
AHC16808.1 (1307761)
CP006939.1 (chromosome)

Spirochaeta lutea
KGE71897.1 (1480694)
JNUP01000064.1 (genomic DNA)

Spirochaetes bacterium GWE1_60_18
OHD46595.1 (1802185)
MIAZ01000005.1 (genomic DNA)

Treponema caldarium DSM 7334
AEJ20684.1 (744872)
CP002868.1 (chromosome)

Treponema azotonutricium ZAS 9
AEF81317.1 (545695)
CP001841.1 (chromosome)

Spirochaetaceae bacterium UBA1318
DBTK01000038.1_33 (1952885)
DBTK01000038.1 ()

Spirochaetaceae bacterium UBA5200
DHVU01000009.1_1 (1952889)
DHVU01000009.1 ()

Spirochaetes bacterium RIFOXYC1_FULL_54_7
OHD81793.1 (1802198)
MIBM01000079.1 (genomic DNA)

Spirochaetae bacterium HGW Spirochaetae 3
PKL26038.1 (2013835)
PGXV01000002.1 (genomic DNA)

Spirochaetaceae bacterium UBA5630
DIKA01000006.1_1 (1952893)
DIKA01000006.1 ()

Spirochaetaceae bacterium UBA5556
DIMW01000011.1_34 (1952892)
DIMW01000011.1 ()

Spirochaetae bacterium HGW Spirochaetae 9
PKL05792.1 (2013841)
PGXP01000185.1 (genomic DNA)

Spirochaetaceae bacterium UBA2256
DDXQ01000185.1_7 (1952886)
DDXQ01000185.1 ()

Spirochaetaceae bacterium UBA5328
DHQW01000001.1_13 (1952890)
DHQW01000001.1 ()

Spirochaeta africana DSM 8902
AFG36189.1 (889378)
CP003282.1 (chromosome)

Spirochaeta sp UBA1101

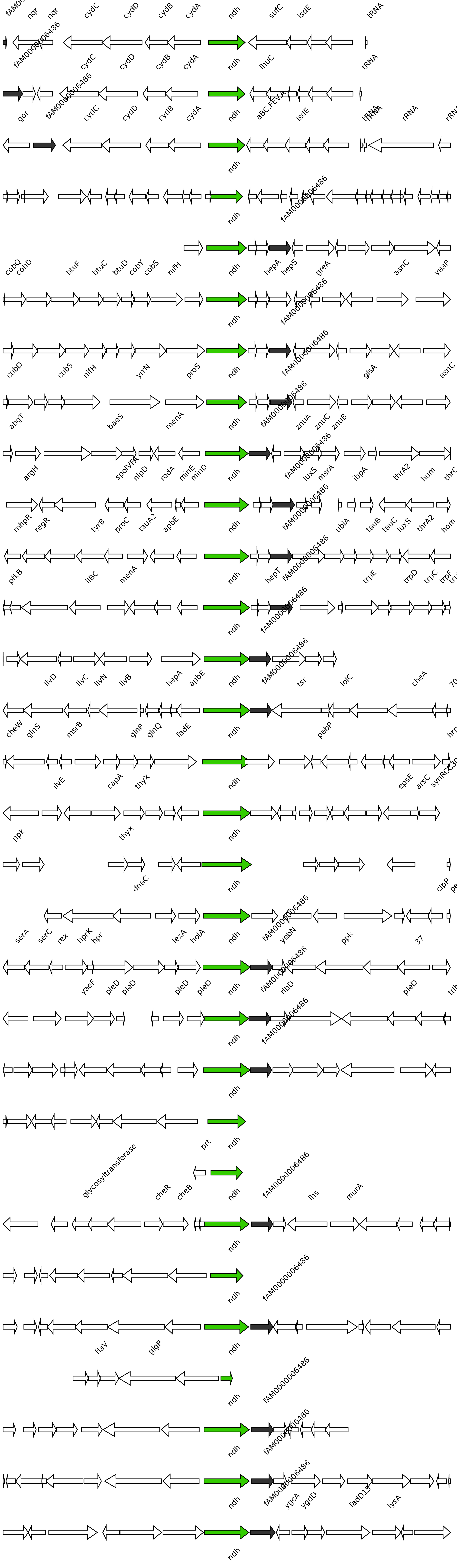

[illegible]
DCBS01000208.1_7 (1947540)
DCBS01000208.1 ()

Alkalispirochaeta americana
SIQ20904.1 (159291)
FTMS01000005.1 (genomic DNA)

Spirochaeta thermophila DSM 6192
ADN02919.1 (665571)
CP001698.1 (chromosome)

Caldanaerobius fijiensis DSM 17918
SHF56520.1 (1121256)
FQVH01000029.1 (genomic DNA)

Caldanaerobius polysaccharolyticus DSM 13641
KE386494.1_555 (935948)
KE386494.1 ()

Caldanaerobacter subterraneus subsp tengcongensis MB4
AAM25616.1 (273068)
AE008691.1 (chromosome)

Thermoanaerobacter italicus Ab9
ADD03370.1 (580331)
CP001936.1 (chromosome)

Clostridium homopropionicum
SFF67201.1 (36844)
FOOL01000001.1 (genomic DNA)

Clostridium sp USBA 49
SKA72808.1 (1881060)
FUYD01000001.1 (genomic DNA)

Clostridiales bacterium oral taxon 876 str F0540
ERI90117.1 (1321778)
KE993508.1 (genomic)

Clostridiales bacterium GWB2_37_7
OGO78099.1 (1797677)
MGOS01000063.1 (genomic DNA)

Caloramator australicus RC3
CCJ34172.1 (857293)
CAKP01000110.1 (genomic DNA)

Caloramator mitchellensis
KRQ86727.1 (908809)
LKHP01000007.1 (genomic DNA)

Lutispora thermophila DSM 19022
SHI73519.1 (1122184)
FQZS01000007.1 (genomic DNA)

Clostridiales bacterium GWB2_37_7
OGO78259.1 (1797677)
MGOS01000054.1 (genomic DNA)

Clostridium sp UBA701
DBMJ01000022.1_64 (1946375)
DBMJ01000022.1 ()

Oxobacter pfennigii
KPU42309.1 (36849)
LKET01000068.1 (genomic)

Clostridium amazonitimonense
LM994640.1_934 (1499689)
LM994640.1 ()

Clostridium polynesiense
CCXI01000014.1_21 (1325933)
CCXI01000014.1 ()

Clostridium amylolyticum
SHI43477.1 (1121298)
FQZO01000001.1 (genomic DNA)

Clostridium intestinale URNW
ERK32143.1 (1294142)
KI273145.1 (genomic)

Clostridium paraputrificum
CUN49611.1 (29363)
CYZW01000001.1 (genomic DNA)

Clostridium thermopalmarium DSM 5974
PRR72027.1 (1121340)
PVXN01000037.1 (genomic)

Clostridium sp HMP27
KGK86937.1 (1487921)
JMFY01000022.1 (genomic DNA)

Clostridium isatidis
ASW44096.1 (182773)
CP016786.1 (chromosome)

Clostridium gasigenes
SDP81563.1 (94869)
FNJM01000019.1 (genomic DNA)

Clostridium sp 7_2_43FAA
EEH99284.1 (457396)
JH815222.1 (genomic)

Clostridium hydrogeniformans DSM 21757
KK366005.1_204 (1121319)
KK366005.1 ()

Clostridium celatum DSM 1785
EKY23260.1 (545697)
KB291697.1 (genomic)

Clostridium colicanis 209318
ENZ02459.1 (999411)
KB850956.1 (genomic)

Clostridium sp Ade TY
AVSV01000007.1_68 (1391647)
AVSV01000007.1 ()

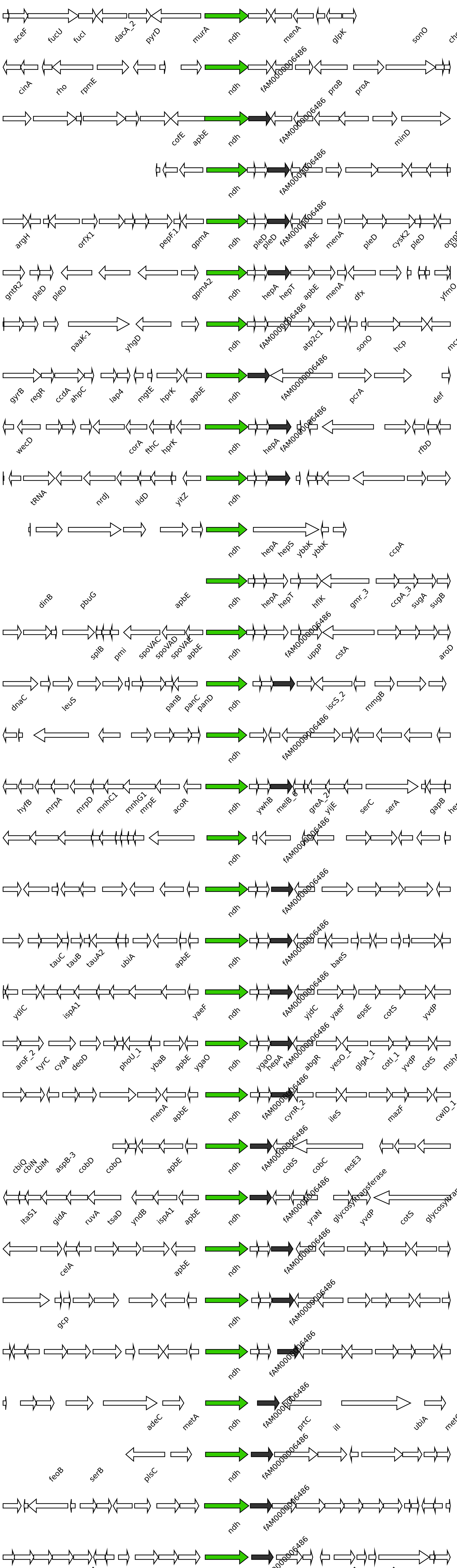

Clostridium baratii str Sullivan
AIY84998.1 (1415775)
CP006905.1 (chromosome)

Romboutsia weinsteinii
RDY25418.1 (2020949)
NOJY02000074.1 (genomic DNA)

Clostridium mediterraneense
LT594783.1_141 (1805472)
LT594783.1 ()

Clostridium dakarense
HG529376.1_2 (1301100)
HG529376.1 ()

Peptoclostridium sp UBA4559
DGKX01000012.1_134 (1947070)
DGKX01000012.1 ()

Niameybacter massiliensis
LN875034.1_522 (1658108)
LN875034.1 ()

Cellulosilyticum lentocellum DSM 5427
ADZ82627.1 (642492)
CP002582.1 (chromosome)

Cellulosilyticum ruminicola JCM 14822
BBCG01000095.1_5 (1294025)
BBCG01000095.1 ()

Firmicutes bacterium UBA3567
DFNM01000150.1_15 (1947964)
DFNM01000150.1 ()

Firmicutes bacterium HGW Firmicutes 7
PKM52536.1 (2013788)
PGZO01000002.1 (genomic DNA)

Firmicutes bacterium HGW Firmicutes 1
PKM93411.1 (2013770)
PHAG01000014.1 (genomic DNA)

Geotoga petraea
SDC76923.1 (28234)
FMYV01000007.1 (genomic DNA)

Oceanotoga teriensis
PWJ93289.1 (515440)
QGGI01000008.1 (genomic DNA)

Clostridiales bacterium 70B A
VDN49014.1 (2173034)
LR130778.1 (genomic DNA)

Clostridiales bacterium UBA1351
DBSD01000101.1_13 (1950818)
DBSD01000101.1 ()

Acidaminobacter hydrogenoformans DSM 2784
SCZ76319.1 (1120920)
FMWL01000001.1 (genomic DNA)

Caloramator quimbayensis
SKA79150.1 (1147123)
FUYH01000003.1 (genomic DNA)

Petroclostridium xylanilyticum
NPML01000019.1_228 (1792311)
NPML01000019.1 ()

Peptoclostridium litorale DSM 5388
KDR96531.1 (1121324)
JJMM01000002.1 (genomic)

Anaeromicrobium sediminis
PAB59069.1 (1478221)
NIBG01000009.1 (genomic DNA)

Proteiniborus ethanoligenes
SDZ28143.1 (415015)
FNQE01000030.1 (genomic DNA)

Firmicutes bacterium UBA6767
DKGP01000013.1_11 (1948097)
DKGP01000013.1 ()

Peptoclostridium litorale DSM 5388
KDR96530.1 (1121324)
JJMM01000002.1 (genomic)

Paenibacillus assamensis DSM 18201
AULU01000009.1_165 (1122916)
AULU01000009.1 ()

Paenibacillus popilliae ATCC 14706
GAC44211.1 (1212764)
BALG01000382.1 (genomic DNA)

Brevibacillus laterosporus
JNFS01000001.1_211 (1465)
JNFS01000001.1 ()

Paenibacillus larvae subsp larvae B 3650
PCK71670.1 (741161)
ADZY03000091.1 (genomic DNA)

Paenibacillus lentus
AZK48814.1 (1338368)
CP034248.1 (chromosome)

Paenibacillus sp IHBB 10380
AJS61052.1 (1566358)
CP010976.1 (chromosome)

Paenibacillus durus
AIQ10966.1 (44251)
CP009288.1 (chromosome)

Gorillibacterium timonense

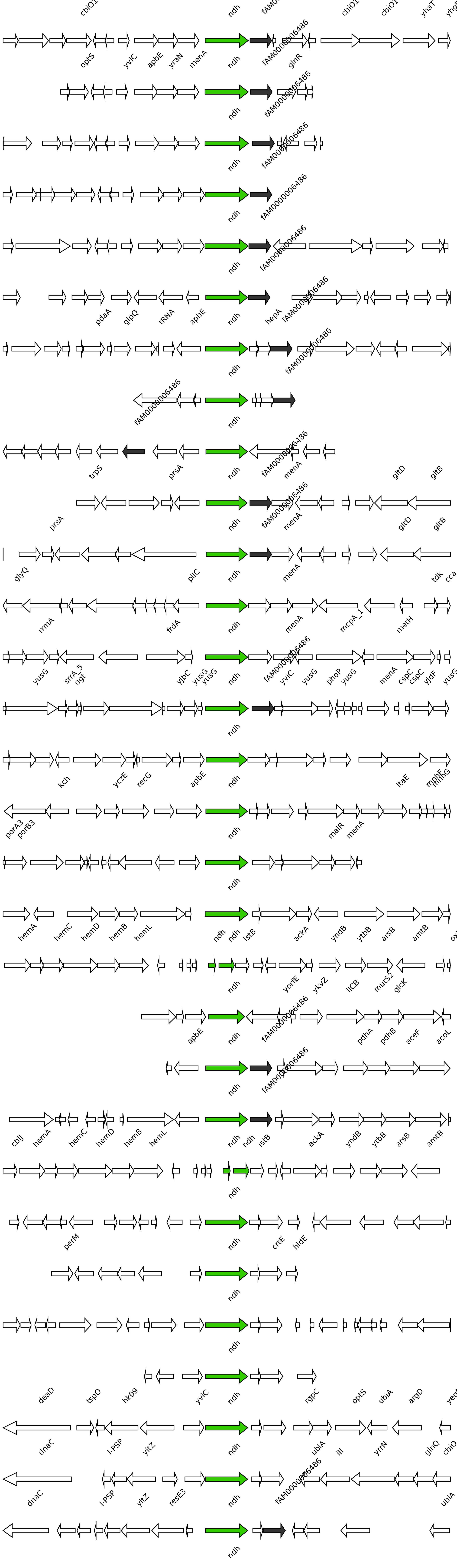

LN881721.1_314 (1689269)
LN881721.1 ()

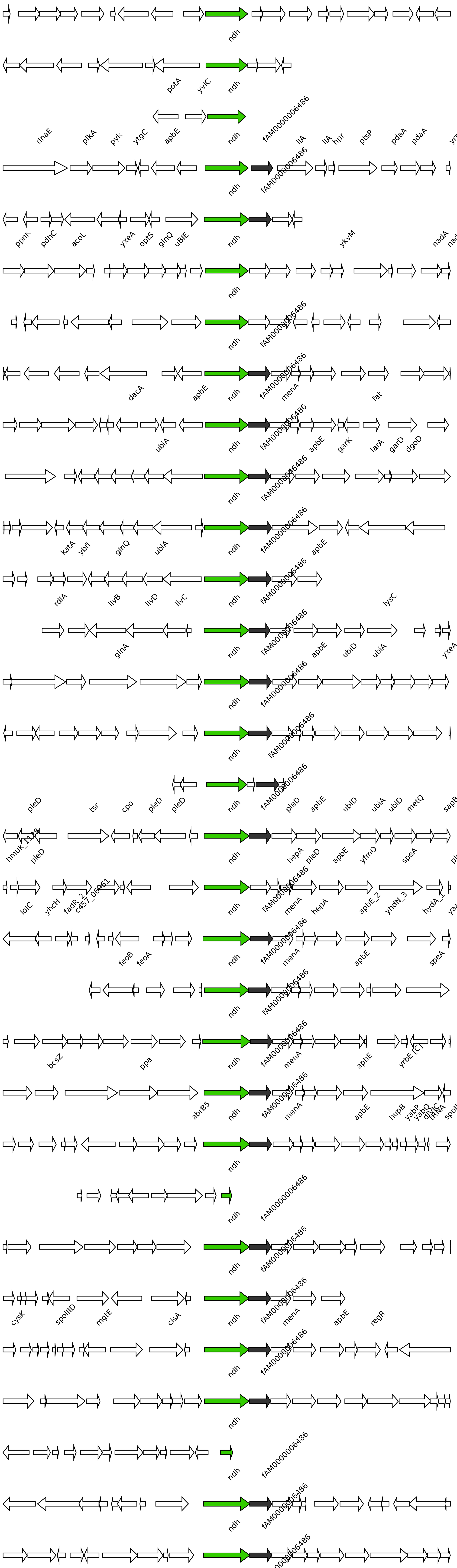

Bacillales bacterium UBA4045
DFZX01000082.1_4 (1950274)
DFZX01000082.1 ()

Paenibacillaceae bacterium ZCTH02 B3
OUM97134.1 (1861638)
LZRV01000146.1 (genomic DNA)

Turicibacter sp H121
AMC07973.1 (1712675)
CP013476.1 (chromosome)

Mageeibacillus sp UBA5734
DIGA01000118.1_13 (1946797)
DIGA01000118.1 ()

Acetobacterium bakii
KNZ43335.1 (52689)
LGYO01000004.1 (genomic DNA)

Clostridiales bacterium UBA6114
DIWE01000023.1_164 (1950909)
DIWE01000023.1 ()

Ruminococcaceae bacterium UBA5401
DHOB01000042.1_23 (1952391)
DHOB01000042.1 ()

Sporobacter termitidis DSM 10068
SHI17621.1 (1123282)
FQXV01000012.1 (genomic DNA)

Harryflintia acetispora
RGB66778.1 (1849041)
QVEO01000008.1 (genomic DNA)

Anaerotruncus sp AT3
LT962687.1_2073 (1673720)
LT962687.1 ()

Anaerotruncus sp AF02 27
RGX52590.1 (2292191)
QSBX01000044.1 (genomic DNA)

Acetoanaerobium sticklandii
CBH21539.1 (1511)
FP565809.1 (genomic DNA)

Acetivibrio ethanolgignens
KSV57452.1 (290052)
LNAM01000219.1 (genomic DNA)

Firmicutes bacterium UBA5010
DGYI01000061.1_20 (1948033)
DGYI01000061.1 ()

Firmicutes bacterium UBA1535
DCPV01000254.1_4 (1947938)
DCPV01000254.1 ()

Clostridium saccharolyticum WM1
ADL06751.1 (610130)
CP002109.1 (chromosome)

Ruminiclostridium cellulolyticum H10
ACL75470.1 (394503)
CP001348.1 (chromosome)

Ruminiclostridium hungatei
OPX44420.1 (48256)
MZGX01000009.1 (genomic)

Ruminiclostridium sufflavum DSM 19573
PYG87741.1 (1121337)
QKMR01000009.1 (genomic DNA)

Cellulosilyticum sp I15G10I2
FMMP01000009.1_15 (1892843)
FMMP01000009.1 ()

Anaerosporobacter mobilis DSM 15930
SHM09853.1 (1120996)
FRCP01000006.1 (genomic DNA)

Mobilisporobacter senegalensis
ROR25681.1 (1329262)
RJVG01000010.1 (genomic DNA)

Firmicutes bacterium UBA7709
DLFX01000389.1_9 (1948107)
DLFX01000389.1 ()

Lachnospiraceae bacterium Marseille P3773
LT860099.1_2010 (2002841)
LT860099.1 ()

Lachnoclostridium sp UBA6558
DJJW01000058.1_61 (1946697)
DJJW01000058.1 ()

Anaerocolumna xylanovorans DSM 12503
SHO49436.1 (1121345)
FRFD01000006.1 (genomic DNA)

Proteocatella sphenisci DSM 23131
AUID01000016.1_53 (1123009)
AUID01000016.1 ()

Firmicutes bacterium UBA6132
DIVM01000310.1_26 (1948088)
DIVM01000310.1 ()

Ruminococcaceae bacterium UBA1865
DDHX01000047.1_15 (1952297)
DDHX01000047.1 ()

bacterium MS4
CCFG01000014.1_150 (1120746)
CCFG01000014.1 ()

Ruminococcaceae bacterium UBA2656
DENA01000012.1_4 (1952299)
DENA01000012.1 ()

Ruminococcaceae bacterium UBA3451
DFRY01000053.1_77 (1952318)
DFRY01000053.1 ()

Clostridium sp W14A
OCN00795.1 (1849176)
MBSV01000072.1 (genomic DNA)

Clostridiaceae bacterium DTU054
LFRR01000073.1_17 (1671643)
LFRR01000073.1 ()

Clostridiales bacterium DTU013
LFSV01000091.1_9 (1671648)
LFSV01000091.1 ()

Hungateiclostridiaceae bacterium UBA1305
DBTX01000072.1_31 (1947398)
DBTX01000072.1 ()

Lachnoclostridium sp UBA1308
DBTU01000019.1_148 (1946684)
DBTU01000019.1 ()

Lachnoclostridium sp UBA1434
DCTS01000015.1_28 (1946685)
DCTS01000015.1 ()

Firmicutes bacterium HGW Firmicutes 16
PKM73083.1 (2013777)
PGZZ01000005.1 (genomic DNA)

Kineothrix alysoides
JPNB01000003.1_156 (1469948)
JPNB01000003.1 ()

Lachnospiraceae bacterium UBA5452
DIQW01000045.1_6 (1952103)
DIQW01000045.1 ()

Firmicutes bacterium HGW Firmicutes 11
PKM85626.1 (2013772)
PHAE01000002.1 (genomic DNA)

Firmicutes bacterium UBA1426
DCUA01000229.1_6 (1947936)
DCUA01000229.1 ()

Clostridiales bacterium UBA9856
HAF59712.1 (2060925)
DMDC01000032.1 (genomic DNA)

Peptococcaceae bacterium UBA5767
DIET01000029.1_55 (1952717)
DIET01000029.1 ()

Ruminococcaceae bacterium UBA5396
DHOG01000210.1_14 (1952388)
DHOG01000210.1 ()

Ruminococcaceae bacterium UBA5423
DHNF01000116.1_14 (1952395)
DHNF01000116.1 ()

Clostridiales bacterium DTU033
LFTH01000127.1_24 (1676153)
LFTH01000127.1 ()

Firmicutes bacterium UBA5559
DIMT01000036.1_55 (1948075)
DIMT01000036.1 ()

Clostridiales bacterium GWF2_36_10
OGO88507.1 (1797682)
MGOX01000013.1 (genomic DNA)

Clostridiales bacterium GWF2_38_85
OGO89273.1 (1797683)
MGOY01000042.1 (genomic DNA)

Catabacter sp UBA7707
DLFZ01000159.1_3 (1946301)
DLFZ01000159.1 ()

Sporobacter termitidis DSM 10068
SHI02251.1 (1123282)
FQXV01000006.1 (genomic DNA)

Clostridiales bacterium UBA1316
DBTM01000060.1_37 (1950814)
DBTM01000060.1 ()

Lachnospiraceae bacterium XBD2001
SFT32182.1 (1520820)
FPAN01000002.1 (genomic DNA)

Erysipelothrix larvae
AMC92937.1 (1514105)
CP013213.1 (chromosome)

Erysipelothrix sp UBA3383
DEPV01000042.1_19 (1946468)
DEPV01000042.1 ()

Firmicutes bacterium UBA7709
DLFX01000281.1_1 (1948107)
DLFX01000281.1 ()

Firmicutes bacterium UBA5774
DIEM01000001.1_32 (1948080)
DIEM01000001.1 ()

Spirochaeta cellobiosiphila DSM 17781
AUFW01000007.1_221 (1123275)
AUFW01000007.1 ()

Olavius algarvensis spirochete endosymbiont

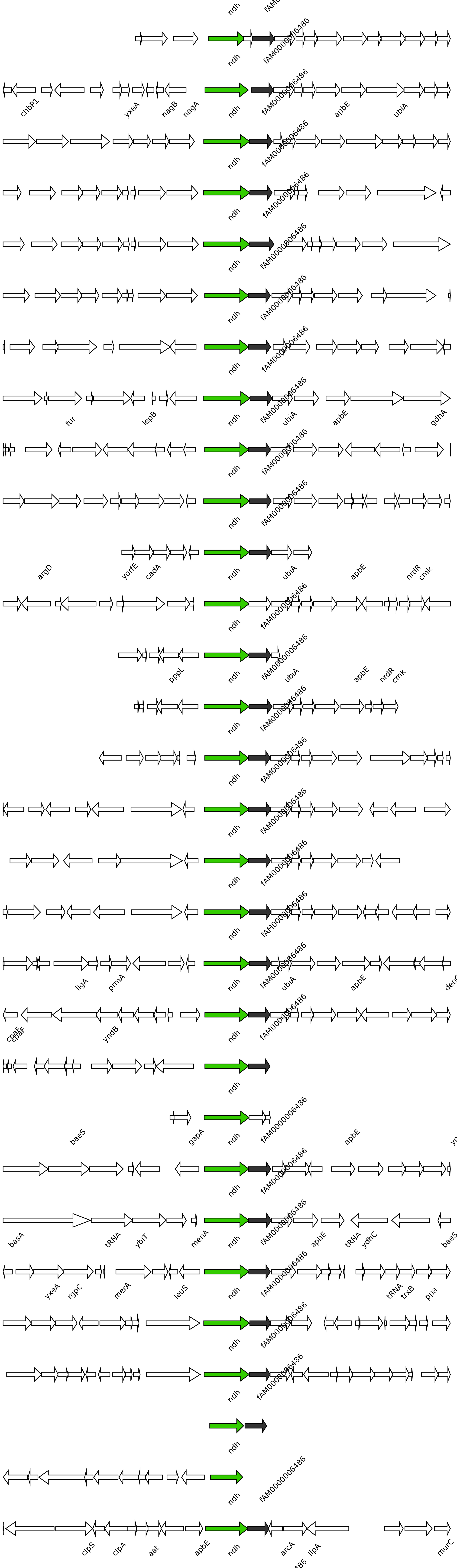

VDA99994.1 (260710)
UWYT01000178.1 (genomic DNA)

Sphaerochaeta halotolerans
RFU94931.1 (2293840)
QUWK01000006.1 (genomic DNA)

Sphaerochaeta globosa str Buddy
ADY12899.1 (158189)
CP002541.1 (chromosome)

Spirochaetales bacterium Spiro_03
LVBE01000176.1_5 (1674859)
LVBE01000176.1 ()

Sphaerochaeta pleomorpha str Grapes
AEV28649.1 (158190)
CP003155.1 (chromosome)

Spirochaetae bacterium HGW Spirochaetae 8
PKL14558.1 (2013840)
PGXQ01000010.1 (genomic DNA)

Spirochaetes bacterium GWF2_52_7
OHD66450.1 (1802192)
MIBG01000052.1 (genomic DNA)

Spirochaetae bacterium HGW Spirochaetae 2
PKL28448.1 (2013834)
PGXW01000013.1 (genomic DNA)

Halanaerobium praevalens DSM 2228
ADO76779.1 (572479)
CP002175.1 (chromosome)

Halanaerobium sp DL 01
RCW87999.1 (1653064)
QPJN01000005.1 (genomic DNA)

Halanaerobium hydrogeniformans
ADQ14332.1 (656519)
CP002304.1 (chromosome)

Halanaerobium salsuginis
SFL65106.1 (29563)
FOTI01000022.1 (genomic DNA)

Halothermothrix orenii H 168
ACL70235.1 (373903)
CP001098.1 (chromosome)

Halocella sp SP3 1
AZO94232.1 (2382161)
CP032760.1 (chromosome)

Halothermothrix sp UBA3952
DGDM01000124.1_4 (1946587)
DGDM01000124.1 ()

Thermotalea metallivorans
KXG77056.1 (520762)
LOEE01000019.1 (genomic DNA)

Geosporobacter subterraneus DSM 17957
SHJ89689.1 (1121919)
FQZV01000051.1 (genomic DNA)

Defluviitalea phaphyphila
JWID01000020.1_37 (1473580)
JWID01000020.1 ()

Thermoclostridium stercorarium subsp stercorarium DSM 8532
AGC69551.1 (1121335)
CP004044.1 (chromosome)

Clostridiales bacterium DTU070
LFSH01000052.1_11 (1671655)
LFSH01000052.1 ()

Anaerobacterium chartisolvens
RCX11165.1 (1297424)
QPJT01000027.1 (genomic DNA)

Pseudoclostridium thermosuccinogenes
AUS95841.1 (84032)
CP021850.1 (chromosome)

Hungateiclostridium thermocellum ATCC 27405
ABN51796.1 (203119)
CP000568.1 (chromosome)

Clostridiales bacterium UBA3578
DFNB01000083.1_105 (1950858)
DFNB01000083.1 ()

Ruminiclostridium hungatei
OPX42341.1 (48256)
MZGX01000030.1 (genomic)

Firmicutes bacterium HGW Firmicutes 12
PKM90844.1 (2013773)
PHAD01000004.1 (genomic DNA)

Clostridiales bacterium UBA3948
DGDQ01000114.1_7 (1950871)
DGDQ01000114.1 ()

Clostridiales bacterium UBA3582
DFMX01000110.1_8 (1950859)
DFMX01000110.1 ()

Orenia marismortui DSM 5156
KB900622.1_154 (926561)
KB900622.1 ()

Halobacteroides halobius DSM 5150
AGB41902.1 (748449)
CP003359.1 (chromosome)

Orenia metallireducens
OCL26034.1 (1413210)
LWDV01000009.1 (genomic DNA)

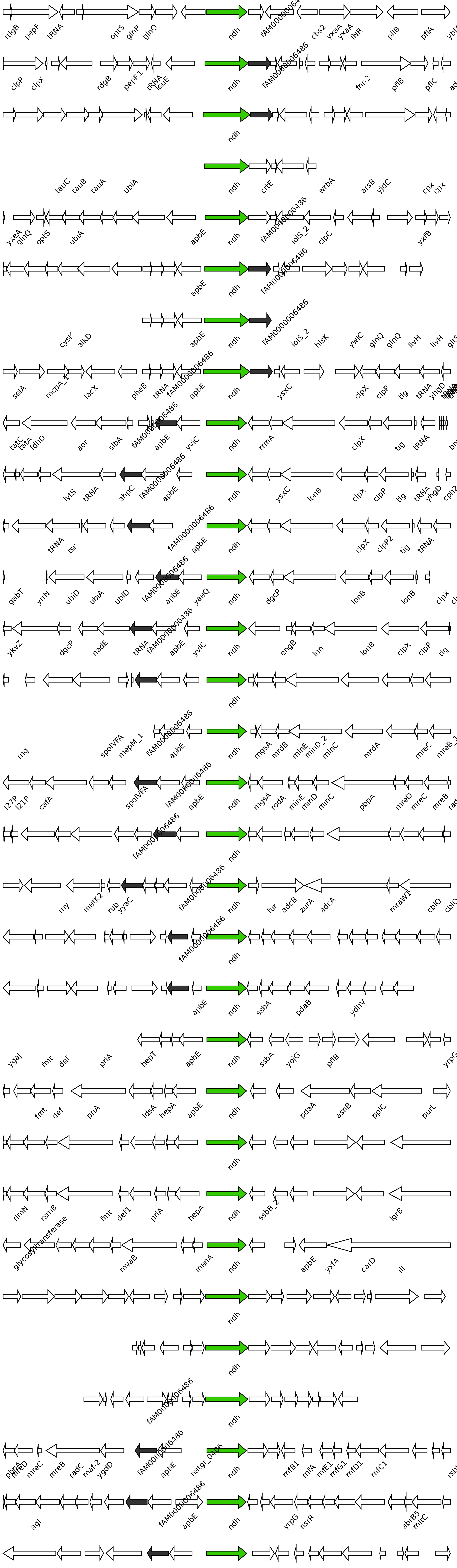

Firmicutes bacterium UBA3941
DGDX01000025.1_13 (1947994)
DGDX01000025.1 ()

Caldicoprobacter faecalis
SFQ10009.1 (937334)
FOXR01000012.1 (genomic DNA)

Clostridiales bacterium DTU074
LFSE01000111.1_1 (1671658)
LFSE01000111.1 ()

Firmicutes bacterium UBA3942
DGDW01000079.1_17 (1947995)
DGDW01000079.1 ()

Firmicutes bacterium UBA3941
DGDX01000025.1_12 (1947994)
DGDX01000025.1 ()

Mahella australiensis 50 1 BON
AEE97133.1 (697281)
CP002360.1 (chromosome)

Thermoanaerobacterium thermosaccharolyticum DSM 571
ADL68590.1 (580327)
CP002171.1 (chromosome)

Thermoanaerobacterales bacterium SK G1
AYO31041.1 (2316383)
CP033169.1 (chromosome)

Thermosediminibacter oceani DSM 16646
ADL08466.1 (555079)
CP002131.1 (chromosome)

Caldanaerovirga acetigignens
SHM59140.1 (447595)
FRCR01000007.1 (genomic DNA)

Thermovenabulum gondwanense
KYO66531.1 (520767)
LOHZ01000027.1 (genomic DNA)

Mesoaciditoga sp
PMP78668.1 (2060323)
PNIW01000097.1 (genomic DNA)

Clostridiales bacterium UBA6114
DIWE01000023.1_72 (1950909)
DIWE01000023.1 ()

Haloplasma contractile SSD 17B
ERJ12588.1 (1033810)
AFNU02000004.1 (genomic DNA)

Inordinaticella fortuita
NSKI01000006.1_68 (1274318)
NSKI01000006.1 ()

Hungateiclostridiaceae bacterium UBA1305
DBTX01000095.1_36 (1947398)
DBTX01000095.1 ()

Ruminococcaceae bacterium UBA3334
DERS01000064.1_12 (1952317)
DERS01000064.1 ()

Spirochaetes bacterium GWE2_31_10
OHD46137.1 (1802186)
MIBA01000093.1 (genomic DNA)

Firmicutes bacterium HGW Firmicutes 11
PKM85545.1 (2013772)
PHAE01000003.1 (genomic DNA)

Firmicutes bacterium UBA5559
DIMT01000029.1_20 (1948075)
DIMT01000029.1 ()

Methanocellaceae archaeon UBA148
DAHG01000033.1_41 (1915626)
DAHG01000033.1 ()

Weissella koreensis KACC 15510
AEJ23730.1 (1045854)
CP002899.1 (chromosome)

Weissella kandleri
KRN74874.1 (1616)
JQBP01000004.1 (genomic)

Arcobacter sp 31_11_sub10_T18
OUR74206.1 (1856277)
MAAG01000029.1 (genomic DNA)

Arcobacter sp UBA7394
DKNG01000067.1_6 (1946052)
DKNG01000067.1 ()

Anaerobacillus arseniciselenatis
OIJ09766.1 (85682)
MLQQ01000042.1 (genomic DNA)

Bacillus sp FJAT 18017
ALC91227.1 (1705566)
CP012602.1 (chromosome)

Bacillus sp AFS006103
PEQ94924.1 (2033483)
NTXX01000008.1 (genomic DNA)

Estrella lausannensis
CRX37669.1 (483423)
CWGJ01000005.1 (genomic DNA)

Prochlorococcus marinus subsp pastoris str CCMP1986
CAE18541.1 (59919)
BX548174.1 (genomic DNA)

Eggerthellaceae bacterium AT8
LN998085.1_93 (1720215)

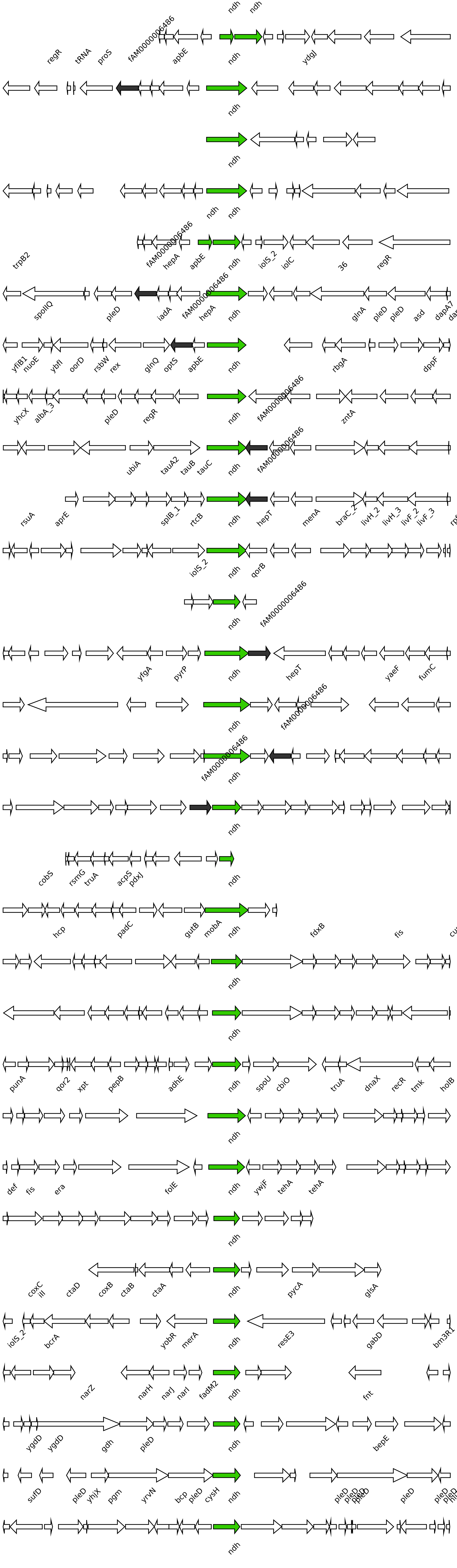

LN908985.1_93 (1720315)
LN908985.1 ()

Helicobacter sp MIT 99 5507
RDU57508.1 (152489)
NXLO01000003.1 (genomic DNA)

Helicobacter sp 16 1353
RAX51486.1 (2004996)
NHYM01000019.1 (genomic DNA)

Helicobacter sp MIT 14 3879
RDU65230.1 (2040649)
NXLP01000002.1 (genomic DNA)

Campylobacter geochelonis
CZE51325.1 (1780362)
FIZQ01000012.1 (genomic DNA)

Helicobacter mustelae 12198
CBG40183.1 (679897)
FN555004.1 (genomic DNA)

Helicobacter anseris
RDU72490.1 (375926)
NXLX01000019.1 (genomic DNA)

Helicobacter sp house sparrow 1
FZQY01000004.1_526 (2020247)
FZQY01000004.1 ()

Helicobacter pametensis ATCC 51478
JADE01000006.1_84 (1408442)
JADE01000006.1 ()

Helicobacter cholecystus
RDU69087.1 (45498)
NXLU01000004.1 (genomic DNA)

Helicobacter brantae
RDU72253.1 (375927)
NXLV01000001.1 (genomic DNA)

Campylobacter sp MIT 97 5078
KGI57298.1 (1548153)
JRMR01000001.1 (genomic DNA)

Campylobacter avium LMG 24591
ASQ30777.1 (522484)
CP022347.1 (chromosome)

Helicobacter sp MIT 17 337
RDU67605.1 (2040648)
NXLQ01000001.1 (genomic DNA)

Helicobacter macacae MIT 99 5501
ETD25192.1 (1357400)
KI669454.1 (genomic)

Helicobacter sp CLO 3
OBV29848.1 (211)
MAMQ01000042.1 (genomic DNA)

Helicobacter canis NCTC 12740
ETD26541.1 (1357399)
KI669458.1 (genomic)

Helicobacter sp UBA3407
DEOX01000065.1_4 (1946588)
DEOX01000065.1 ()

Helicobacter canadensis MIT 98 5491
EFR47949.1 (537970)
DS990368.1 (genomic)

Helicobacter sp MIT 11 5569
KGL10850.1 (1548151)
JRPB01000006.1 (genomic DNA)

Helicobacter sp CNRCH 2005 566H
QXJG01000002.1_173 (2315334)
QXJG01000002.1 ()

Helicobacter mesocricetorum
FZPJ01000024.1_39 (87012)
FZPJ01000024.1 ()

Helicobacter sp 15 1451
RAX58934.1 (2004995)
NHYN01000002.1 (genomic DNA)

Helicobacter saguini
KGI51546.1 (1548018)
JRMP01000178.1 (genomic DNA)

Helicobacter jaachi
KGI88185.1 (1677920)
JRPR01000003.1 (genomic DNA)

Helicobacter cinaedi PAGU611
BAM13145.1 (1172562)
AP012344.1 (genomic DNA)

Helicobacter marmotae
RDU60249.1 (152490)
NXLR01000004.1 (genomic DNA)

Helicobacter hepaticus ATCC 51449
AAP77840.1 (235279)
AE017125.1 (chromosome)

Helicobacter japonicus
KGI53938.1 (425400)
JRMQ01000002.1 (genomic DNA)

Helicobacter typhlonius
KGL18078.1 (76936)
JRPF01000005.1 (genomic DNA)

Helicobacter himalayensis
CP014991.1_599 (1591088)
CP014991.1 ()

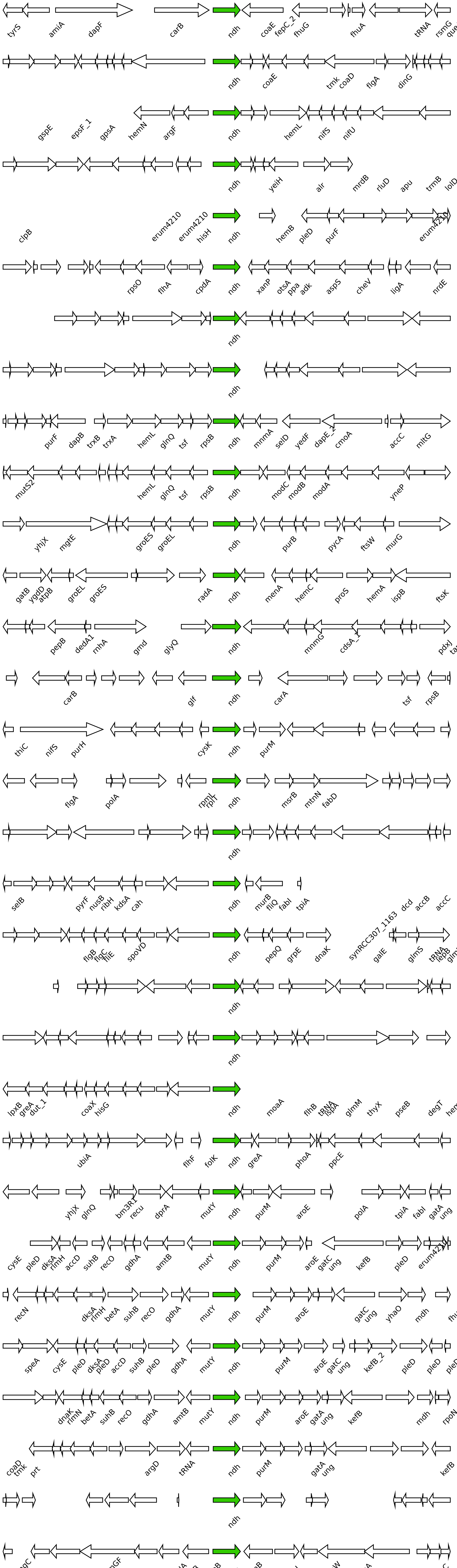

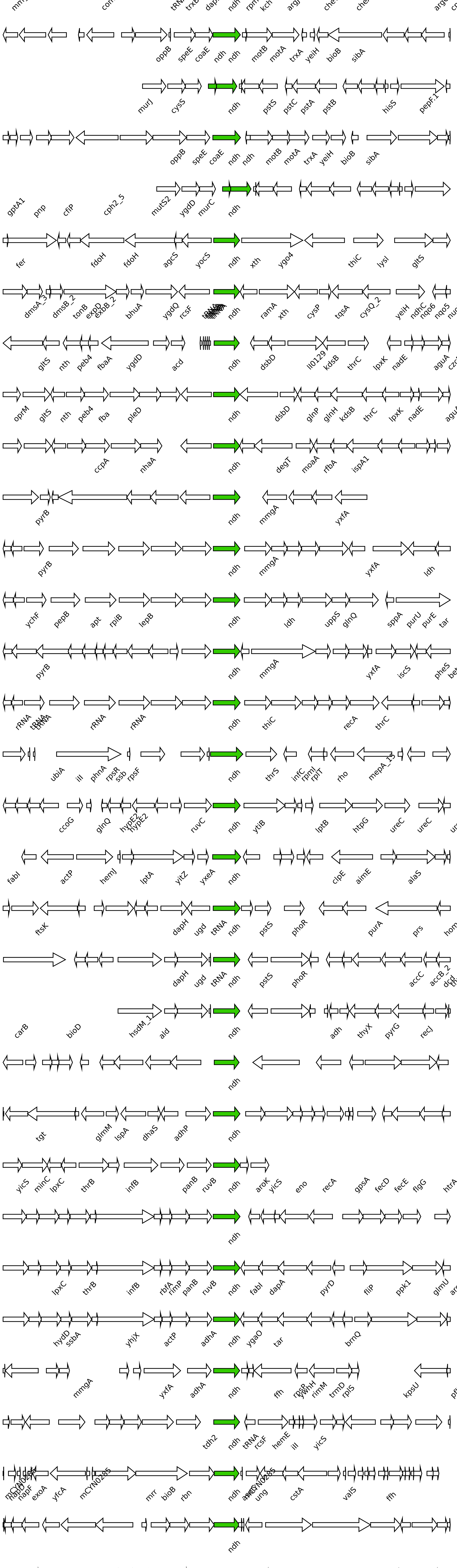

Helicobacter sp 10 6591
RAX55728.1 (2004998)
NHYK01000006.1 (genomic DNA)

Helicobacter fennelliae MRY12 0050
GAD20109.1 (1325130)
BASD01000034.1 (genomic DNA)

Helicobacter sp TUL
PAV00054.1 (1848928)
NESU01000007.1 (genomic DNA)

Helicobacter fennelliae MRY12 0050
GAD20108.1 (1325130)
BASD01000034.1 (genomic DNA)

Campylobacter sputorum bv faecalis CCUG 20703
ASM36883.1 (1031920)
CP019683.1 (chromosome)

Campylobacter blaseri
PSM53272.1 (2042961)
PDHH01000001.1 (genomic DNA)

Campylobacter geochelonis
CZE50426.1 (1780362)
FIZQ01000003.1 (genomic DNA)

Campylobacter insulaenigrae NCTC 12927
AJC87838.1 (1031564)
CP007770.1 (chromosome)

Campylobacter lari RM2100
ACM64233.1 (306263)
CP000932.1 (chromosome)

Helicobacter sp 13S00482 2
PAF53185.1 (1476200)
MLAT01000016.1 (genomic DNA)

Helicobacter sp 13S00477 4
PAF52773.1 (1905759)
MLAS01000001.1 (genomic DNA)

Helicobacter sp 12S02232 10
PAF49937.1 (1476197)
MLAQ01000001.1 (genomic DNA)

Helicobacter sp 12S02634 8
PAF48363.1 (1476199)
MLAP01000001.1 (genomic DNA)

Helicobacter sp 11S03491 1
PAF42549.1 (1476196)
MLAO01000004.1 (genomic DNA)

Helicobacter macacae MIT 99 5501
ETD22162.1 (1357400)
KI669456.1 (genomic)

Helicobacter bilis ATCC 43879
EEO23674.1 (613026)
KI392040.1 (genomic)

Helicobacter sp MIT 17 337
RDU66208.1 (2040648)
NXLQ01000006.1 (genomic DNA)

Helicobacter aurati
RDU71346.1 (137778)
NXLW01000012.1 (genomic DNA)

Helicobacter fennelliae MRY12 0050
GAD19826.1 (1325130)
BASD01000027.1 (genomic DNA)

Helicobacter fennelliae MRY12 0050
GAD20034.1 (1325130)
BASD01000031.1 (genomic DNA)

Campylobacter avium LMG 24591
ASQ31261.1 (522484)
CP022347.1 (chromosome)

Helicobacter mesocricetorum
FZPJ01000012.1_20 (87012)
FZPJ01000012.1 ()

Helicobacter sp MIT 14 3879
RDU63185.1 (2040649)
NXLP01000007.1 (genomic DNA)

Helicobacter mustelae 12198
CBG39444.1 (679897)
FN555004.1 (genomic DNA)

Helicobacter sp house sparrow 1
FZQY01000004.1_315 (2020247)
FZQY01000004.1 ()

Helicobacter anseris
RDU74611.1 (375926)
NXLX01000001.1 (genomic DNA)

Helicobacter saguini
KGI52887.1 (1548018)
JRMP01000177.1 (genomic DNA)

Helicobacter sp 16 1353
RAX52065.1 (2004996)
NHYM01000013.1 (genomic DNA)

Helicobacter heilmannii
CRF50759.1 (35817)
CDMM01000026.1 (genomic DNA)

Helicobacter felis ATCC 49179
CBY82537.1 (936155)
FQ670179.2 (genomic DNA)

Helicobacter suis
RDAO01000004.1_96 (104628)

[illegible]
BDAO01000004.1 ()

Helicobacter baculiformis
FZMF01000028.1_9 (427351)
FZMF01000028.1 ()

Helicobacter sp L2
QXQT01000001.1_76 (2316076)
QXQT01000001.1 ()

Helicobacter salomonis
FZMB01000047.1_29 (56878)
FZMB01000047.1 ()

Helicobacter bizzozeronii
FZEH01000031.1_8 (56877)
FZEH01000031.1 ()

Helicobacter sp CLO 3
OBV29123.1 (211)
MAMQ01000075.1 (genomic DNA)

Helicobacter canis NCTC 12740
ETD26572.1 (1357399)
KI669458.1 (genomic)

Helicobacter sp 11S02629 2
PAF45481.1 (1476195)
MLAN01000003.1 (genomic DNA)

Campylobacter mucosalis
KEA46491.1 (202)
JHQQ01000001.1 (genomic DNA)

Campylobacter pinnipediorum subsp caledonicus
OPA72163.1 (1874362)
MBGA01000025.1 (genomic DNA)

Campylobacter blaseri
PSM52016.1 (2042961)
PDHH01000004.1 (genomic DNA)

Blastocatellia bacterium
PLCD01000017.1_422 (2052146)
PLCD01000017.1 ()

Chloracidobacterium sp UBA4013
DGBD01000064.1_17 (1946318)
DGBD01000064.1 ()

Chloracidobacterium sp UBA6041
DIYZ01000227.1_12 (1946320)
DIYZ01000227.1 ()

Pyrinomonas methylaliphatogenes
CDM65704.1 (454194)
CBXV010000006.1 (genomic DNA)

Acidobacteria bacterium 28 1
MWYZ01000070.1_75 (1962177)
MWYZ01000070.1 ()

Cyanobium gracile PCC 6307
AFY28727.1 (292564)
CP003495.1 (chromosome)

Cyanobium sp Baikal G2
PHX67405.1 (2026889)
NSHT01000191.1 (genomic DNA)

Synechococcus sp MW101C3
NQKX01000006.1_31 (210768)
NQKX01000006.1 ()

Candidatus Synechococcus spongiarum 142
KKZ10820.1 (1608213)
JXUO01000305.1 (genomic DNA)

Synechococcales bacterium UBA8138
HBU26487.1 (2055796)
DOMQ01000094.1 (genomic DNA)

Cyanobacteria bacterium UBA7373
DKOB01000140.1_7 (1947895)
DKOB01000140.1 ()

Synechococcus sp RCC307
CAK27292.1 (316278)
CT978603.1 (genomic DNA)

Synechococcus sp WH 8102
CAE08680.1 (84588)
BX548020.1 (genomic DNA)

Synechococcus sp CPC35
MAB54741.1 (2024880)
NYUU01000035.1 (genomic DNA)

Prochlorococcus marinus str MIT 9313
CAE21800.1 (74547)
BX548175.1 (genomic DNA)

Synechococcus sp CC9311
ABI46485.1 (64471)
CP000435.1 (chromosome)

Prochlorococcus marinus subsp marinus str CCMP1375
AAP99142.1 (167539)
AE017126.1 (chromosome)

Prochlorococcus sp MIT 0601
KGG13299.1 (1499498)
JNAU01000004.1 (genomic DNA)

Prochlorococcus sp AG 363 J23
QCFN01000002.1_50 (2162190)
QCFN01000002.1 ()

Prochlorococcus marinus str NATL2A
AAZ58934.1 (59920)
CP000095.2 (chromosome)

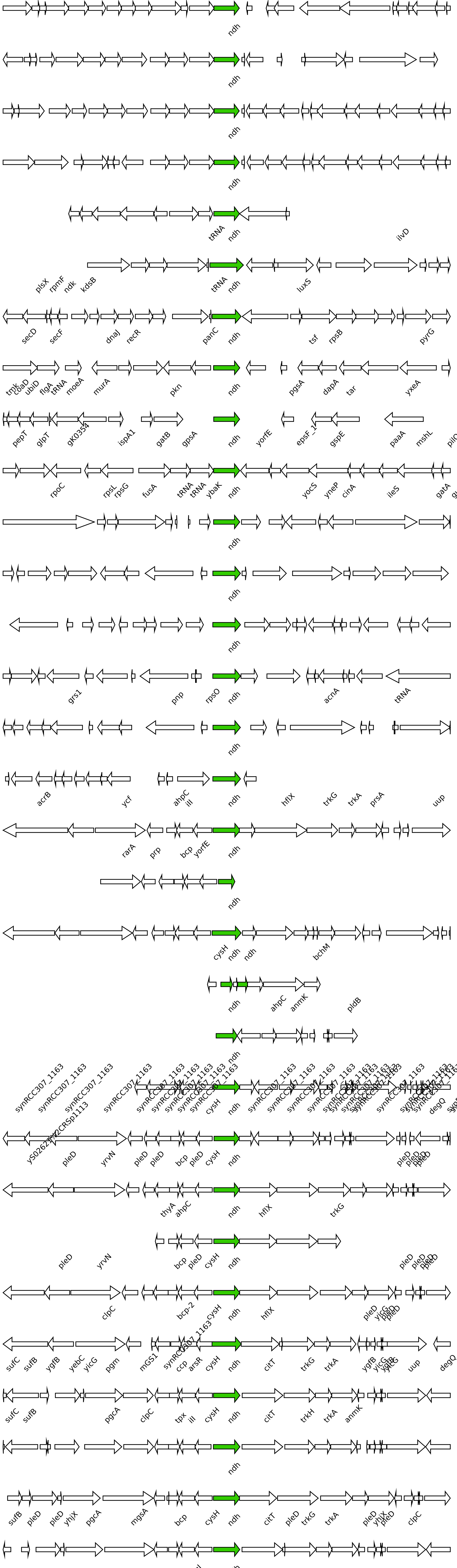

Cyanobacteria bacterium J083
RMF23784.1 (2420336)
RFIT01000541.1 (genomic DNA)

Stanieria cyanosphaera PCC 7437
AFZ36499.1 (111780)
CP003653.1 (chromosome)

Pleurocapsa sp PCC 7327
AFY79194.1 (118163)
CP003590.1 (chromosome)

Aphanothece sacrum FPU1
GBF82880.1 (1920663)
BDQK01000017.1 (genomic DNA)

Cyanothece sp PCC 8801
ACK64138.1 (41431)
CP001287.1 (chromosome)

Aphanothece hegewaldii CCALA 016
PSF39050.1 (2107694)
PXOH01000002.1 (genomic DNA)

Microcystis aeruginosa NIES 2549
AKE66153.1 (1641812)
CP011304.1 (chromosome)

Cyanobacteria bacterium UBA6050
DIYQ01000148.1_28 (1947889)
DIYQ01000148.1 ()

Hydrocoleum sp CS 953
OZH53261.1 (1671698)
LGSU01001201.1 (genomic)

Trichodesmium erythraeum IMS101
ABG53104.1 (203124)
CP000393.1 (chromosome)

Gloeomargarita lithophora Alchichica D10
APB32781.1 (1188229)
CP017675.1 (chromosome)

Synechococcus sp PCC 7002
ACB00102.1 (32049)
CP000951.1 (chromosome)

Thermosynechococcus elongatus BP 1
BAC08688.1 (197221)
BA000039.2 (genomic DNA)

Synechococcus sp PCC 6312
AFY62483.1 (195253)
CP003558.1 (chromosome)

Prochlorothrix hollandica PCC 9006   CALU 1027
KB235933.1_212 (317619)
KB235933.1 ()

Geitlerinema sp PCC 9228
LNDC01000137.1_14 (111611)
LNDC01000137.1 ()

Desertifilum sp IPPAS B 1220
OEJ73491.1 (1781255)
MJGC01000088.1 (genomic DNA)

Chroococcidiopsis sp TS 821
PPS39794.1 (1378066)
MVDI01000014.1 (genomic)

Aliterella atlantica CENA595
KJH69900.1 (1618023)
JYON01000032.1 (genomic DNA)

Chlorogloea sp CCALA 695
PSB33005.1 (2107693)
PVWN01000011.1 (genomic DNA)

Anabaena sp CA   ATCC 33047
LUHI01000044.1_481 (52271)
LUHI01000044.1 ()

Fischerella sp NIES 3754
BAU04249.1 (1752063)
AP017305.1 (genomic DNA)

Anabaena sp 90
AFW94452.1 (46234)
CP003284.1 (chromosome)

Nostoc azollae 0708
ADI65982.1 (551115)
CP002059.1 (chromosome)

Crinalium epipsammum PCC 9333
AFZ13703.1 (1173022)
CP003620.1 (chromosome)

Raphidiopsis curvata NIES 932
BAZ91535.1 (1973482)
AP018317.1 (genomic DNA)

Leptolyngbya sp
PZV12935.1 (47254)
QBMH01000074.1 (genomic DNA)

Geitlerinema sp PCC 7407
AFY66075.1 (1173025)
CP003591.1 (chromosome)

Oscillatoriales cyanobacterium JSC 12
EKQ70766.1 (864702)
CM001633.1 (chromosome)

Oscillatoriales cyanobacterium USR001
OCR01356.1 (1880991)
MBRE01000043.1 (genomic DNA)

Microcoleaceae bacterium UBA11344
HAT13172.1 (2055792)

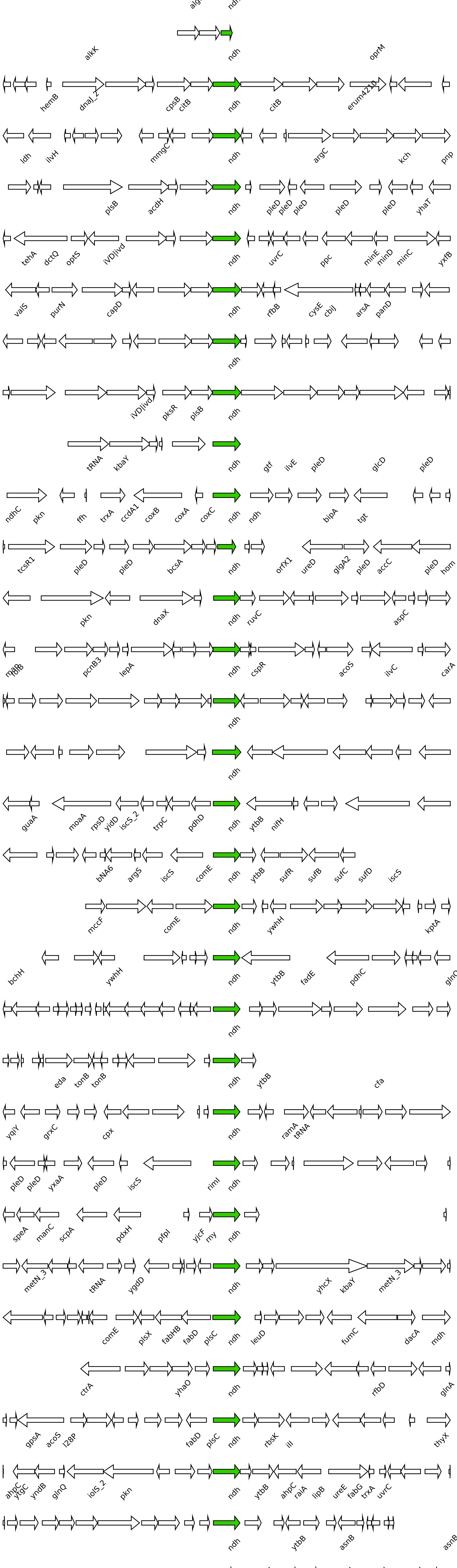

DMYK01000166.1 (genomic DNA)

Microcoleaceae bacterium UBA11344
HAT12238.1 (2055792)
DMYK01000007.1 (genomic DNA)

Cyanobacteria bacterium J007
RMH73976.1 (2420339)
RFFV01000202.1 (genomic DNA)

Hydrocoleum sp CS 953
OZH52162.1 (1671698)
LGSU01001393.1 (genomic)

Trichodesmium erythraeum IMS101
ABG52723.1 (203124)
CP000393.1 (chromosome)

Arthrospira sp O9 13F
RAQ38575.1 (1982223)
PKGD01000915.1 (genomic DNA)

filamentous cyanobacterium CCP1
PSB67865.1 (2107703)
PVWE01000097.1 (genomic DNA)

Cyanobacteria bacterium QS_9_48_30
PSO97371.1 (1919135)
PXPX01000063.1 (genomic DNA)

Cyanobacteria bacterium QS_8_64_29
PSP18474.1 (1919134)
PXQH01000017.1 (genomic DNA)

Pleurocapsa sp PCC 7327
AFY76164.1 (118163)
CP003590.1 (chromosome)

Stanieria cyanosphaera PCC 7437
AFZ37138.1 (111780)
CP003653.1 (chromosome)

Aphanothece sacrum FPU1
GBF78661.1 (1920663)
BDQK01000001.1 (genomic DNA)

Cyanothece sp PCC 8801
ACK64766.1 (41431)
CP001287.1 (chromosome)

Crocosphaera watsonii WH 8502
CCQ53051.1 (423474)
CAQK01000768.1 (genomic DNA)

cyanobacterium endosymbiont of Epithemia turgida isolate EtSB Lake Yunoko
BAP17434.1 (1228987)
AP012549.1 (genomic DNA)

Chondrocystis sp NIES 4102
BAZ44737.1 (2005460)
AP018281.1 (genomic DNA)

Snowella sp
PZV27946.1 (2164131)
QBMS01000004.1 (genomic DNA)

Planktothrix agardhii NIES 204
BBD54647.1 (282423)
AP017991.1 (genomic DNA)

Calothrix sp NIES 3974
BAZ07964.1 (2005462)
AP018254.1 (genomic DNA)

Merismopedia glauca CCAP 1448 3
PSB04209.1 (1296344)
PVWJ01000016.1 (genomic DNA)

Neosynechococcus sphagnicola sy1
KGF73910.1 (1497020)
JJML01000002.1 (genomic DNA)

Geitlerinema sp BBD 1991 9
LWRO01000022.1_57 (1643526)
LWRO01000022.1 ()

Phormidium sp OSCR
KPQ34802.1 (1666905)
LJZT01000088.1 (genomic DNA)

Cyanobacteria bacterium T3Sed10_304
PXCV01000601.1_4 (2099686)
PXCV01000601.1 ()

Cyanobacteria bacterium UBA6050
DIYQ01000012.1_3 (1947889)
DIYQ01000012.1 ()

filamentous cyanobacterium ESFC 1
KB904821.1_771 (1128427)
KB904821.1 ()

Aphanothece hegewaldii CCALA 016
PSF39504.1 (2107694)
PXOH01000001.1 (genomic DNA)

Dactylococcopsis salina PCC 8305
AFZ49943.1 (13035)
CP003944.1 (chromosome)

Halothece sp PCC 7418
AFZ44366.1 (65093)
CP003945.1 (chromosome)

Cyanobacteria bacterium SW_9_44_58
PSO50896.1 (1919144)
PXPI01000016.1 (genomic DNA)

Gloeocapsa sp PCC 73106
ELR98346.1 (102232)
ALVY01000185.1 (genomic DNA)

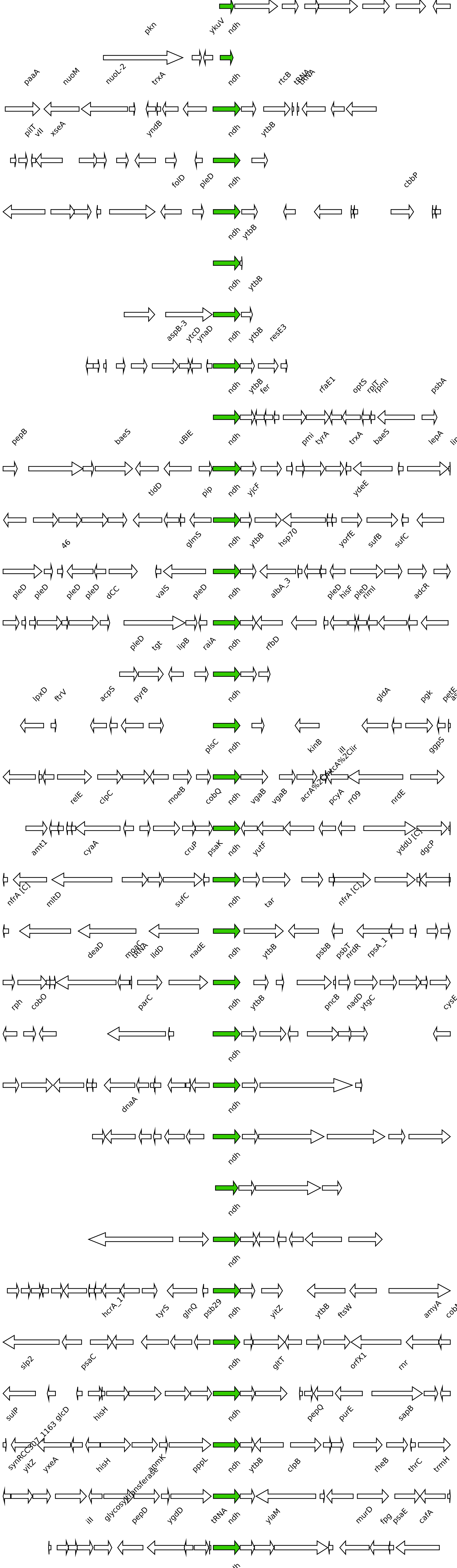

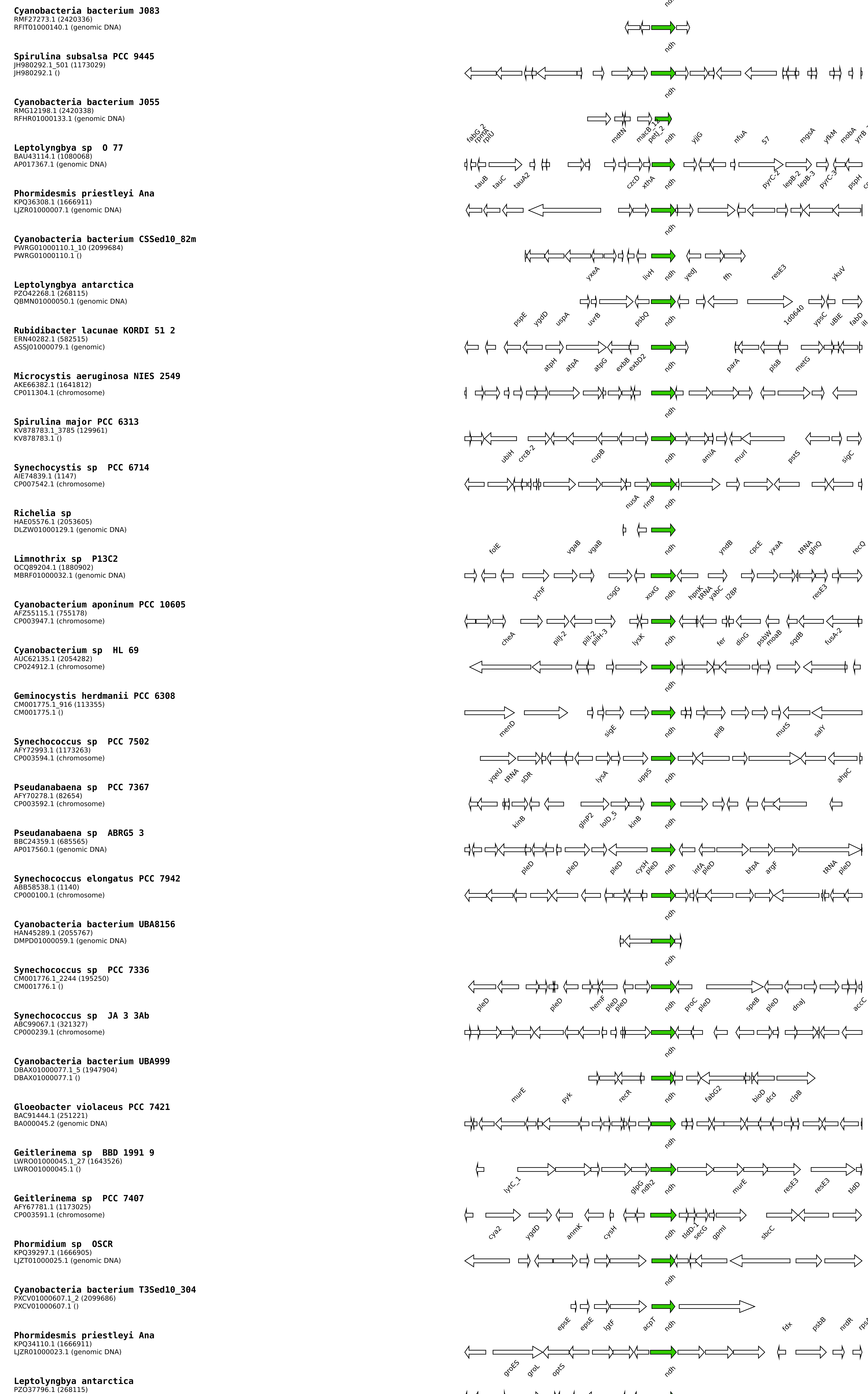
Cyanobacteria bacterium J083
RMF27273.1 (2420336)
RFIT01000140.1 (genomic DNA)
Spirulina subsalsa PCC 9445
JH980292.1_501 (1173029)
JH980292.1 ()
Cyanobacteria bacterium J055
RMG12198.1 (2420338)
RFHR01000133.1 (genomic DNA)
Leptolyngbya sp O 77
BAU43114.1 (1080068)
AP017367.1 (genomic DNA)
Phormidesmis priestleyi Ana
KPQ36308.1 (1666911)
LJZR01000007.1 (genomic DNA)
Cyanobacteria bacterium CSSed10_82m
PWRG01000110.1_10 (2099684)
PWRG01000110.1 ()
Leptolyngbya antarctica
PZO42268.1 (268115)
QBMN01000050.1 (genomic DNA)
Rubidibacter lacunae KORDI 51 2
ERN40282.1 (582515)
ASSJ01000079.1 (genomic)
Microcystis aeruginosa NIES 2549
AKE66382.1 (1641812)
CP011304.1 (chromosome)
Spirulina major PCC 6313
KV878783.1_3785 (129961)
KV878783.1 ()
Synechocystis sp PCC 6714
AIE74839.1 (1147)
CP007542.1 (chromosome)
Richelia sp
HAE05576.1 (2053605)
DLZW01000129.1 (genomic DNA)
Limnothrix sp P13C2
OCQ89204.1 (1880902)
MBRF01000032.1 (genomic DNA)
Cyanobacterium aponinum PCC 10605
AFZ55115.1 (755178)
CP003947.1 (chromosome)
Cyanobacterium sp HL 69
AUC62135.1 (2054282)
CP024912.1 (chromosome)
Geminocystis herdmanii PCC 6308
CM001775.1_916 (113355)
CM001775.1 ()
Synechococcus sp PCC 7502
AFY72993.1 (1173263)
CP003594.1 (chromosome)
Pseudanabaena sp PCC 7367
AFY70278.1 (82654)
CP003592.1 (chromosome)
Pseudanabaena sp ABRG5 3
BBC24359.1 (685565)
AP017560.1 (genomic DNA)
Synechococcus elongatus PCC 7942
ABB58538.1 (1140)
CP000100.1 (chromosome)
Cyanobacteria bacterium UBA8156
HAN45289.1 (2055767)
DMPD01000059.1 (genomic DNA)
Synechococcus sp PCC 7336
CM001776.1_2244 (195250)
CM001776.1 ()
Synechococcus sp JA 3 3Ab
ABC99067.1 (321327)
CP000239.1 (chromosome)
Cyanobacteria bacterium UBA999
DBAX01000077.1_5 (1947904)
DBAX01000077.1 ()
Gloeobacter violaceus PCC 7421
BAC91444.1 (251221)
BA000045.2 (genomic DNA)
Geitlerinema sp BBD 1991 9
LWRO01000045.1_27 (1643526)
LWRO01000045.1 ()
Geitlerinema sp PCC 7407
AFY67781.1 (1173025)
CP003591.1 (chromosome)
Phormidium sp OSCR
KPQ39297.1 (1666905)
LJZT01000025.1 (genomic DNA)
Cyanobacteria bacterium T3Sed10_304
PXCV01000607.1_2 (2099686)
PXCV01000607.1 ()
Phormidesmis priestleyi Ana
KPQ34110.1 (1666911)
LJZR01000023.1 (genomic DNA)
Leptolyngbya antarctica
PZO37796.1 (268115)
ndh

QBMN01000115.1 (genomic DNA)

Cyanobacteria bacterium CSSed10_82m
PWRG01000431.1_2 (2099684)
PWRG01000431.1 ()

Gloeobacter violaceus PCC 7421
BAC89112.1 (251221)
BA000045.2 (genomic DNA)

Oscillatoriales cyanobacterium JSC 12
EKQ66998.1 (864702)
CM001633.1 (chromosome)

Aliterella atlantica CENA595
KJH72111.1 (1618023)
JYON01000007.1 (genomic DNA)

Leptolyngbya sp
PZV09091.1 (47254)
QBMH01000178.1 (genomic DNA)

Cyanobacteria bacterium CSSed10_82m
PWRG01000061.1_9 (2099684)
PWRG01000061.1 ()

Cyanobacteria bacterium QS_9_48_30
PSO96368.1 (1919135)
PXPX01000094.1 (genomic DNA)

Nostoc azollae 0708
ADI64842.1 (551115)
CP002059.1 (chromosome)

Crocosphaera watsonii WH 8502
CCQ49716.1 (423474)
CAQK01000177.1 (genomic DNA)

Synechococcus elongatus PCC 7942
ABB56230.1 (1140)
CP000100.1 (chromosome)

Synechocystis sp PCC 6714
AIE72815.1 (1147)
CP007542.1 (chromosome)

Snowella sp
PZV27957.1 (2164131)
QBMS01000004.1 (genomic DNA)

Cyanobacteria bacterium CSSed10_82m
PWRG01000343.1_5 (2099684)
PWRG01000343.1 ()

Chloracidobacterium thermophilum B
AEP10912.1 (981222)
CP002514.1 (chromosome)

Chloracidobacterium sp CP2_5A
OYT70649.1 (2012633)
NKPT01000033.1 (genomic DNA)

Actinobacteria bacterium 13_1_20CM_3_68_9
OLE38968.1 (1803475)
MNJO01000006.1 (genomic DNA)

Desulfotomaculum ferrireducens
AQS59021.1 (1833852)
CP019698.1 (chromosome)

Desulfotomaculum aeronauticum DSM 10349
SHJ96690.1 (1121421)
FRAR01000004.1 (genomic DNA)

Desulfotomaculum reducens MI 1
ABO48980.1 (349161)
CP000612.1 (chromosome)

Desulfotomaculum aeronauticum DSM 10349
SHK40283.1 (1121421)
FRAR01000012.1 (genomic DNA)

Desulfotomaculum ferrireducens
AQS58171.1 (1833852)
CP019698.1 (chromosome)

Desulfohalotomaculum alkaliphilum DSM 12257
JONT01000012.1_24 (1121423)
JONT01000012.1 ()

Halodesulfurarchaeum formicicum
AOW80227.1 (1873524)
CP016070.1 (chromosome)

Halobacteriales archaeon SW_7_71_33
PSQ59507.1 (1919205)
PXSP01000048.1 (genomic DNA)

Halapricum salinum
BBMO01000001.1_93 (1457250)
BBMO01000001.1 ()

Halobacteriales archaeon QH_2_65_14
PSP28875.1 (1919151)
PXQO01000021.1 (genomic DNA)

Halobacteriales archaeon SW_7_65_23
PSQ47659.1 (1919203)
PXSQ01000079.1 (genomic DNA)

Halobacteriales archaeon QH_6_66_25
PSP45343.1 (1919155)
PXQS01000162.1 (genomic DNA)

Halobacteriales archaeon SW_9_67_25
PSQ60742.1 (1919210)
PXSU01000033.1 (genomic DNA)

Halovenus aranensis
SDJ22000.1 (890420)
FNFC01000001.1 (genomic DNA)

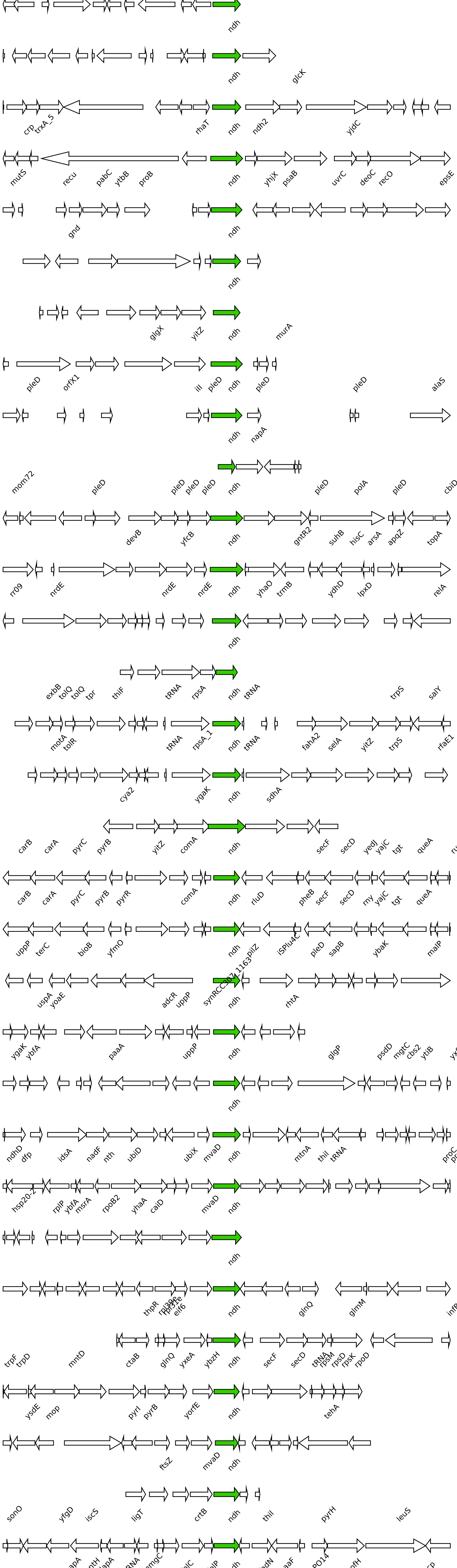

halophilic archaeon J07HX64
ERH09550.1 (1085028)
KE356579.1 (genomic)

Halobacteriales archaeon QH_10_67_13
PSP16805.1 (1919148)
PXQL01000076.1 (genomic DNA)

halophilic archaeon J07HX5
ERG89816.1 (1325472)
KE356559.1 (genomic)

Natronoarchaeum philippinense
SNZ02466.1 (558529)
OBEJ01000001.1 (genomic DNA)

Natrialbaceae archaeon Tc Br11_E2g8
LKNG01000037.1_8 (1729464)
LKNG01000037.1 ()

Saliphagus sp LR7
QPND01000010.1_21 (2282654)
QPND01000010.1 ()

Natronolimnobius aegyptiacus
ARS91427.1 (745377)
CP019893.1 (chromosome)

Natronolimnobius innermongolicus JCM 12255
ELY61066.1 (1227499)
AOHZ01000015.1 (genomic DNA)

Haloterrigena turkmenica DSM 5511
ADB59976.1 (543526)
CP001860.1 (chromosome)

Haloterrigena salina JCM 13891
ELZ23897.1 (1227488)
AOIS01000010.1 (genomic DNA)

Haloterrigena sp ZY19
RQWN01000002.1_603 (2491022)
RQWN01000002.1 ()

Halobiforma lacisalsi AJ5
APW99909.1 (358396)
CP019285.1 (chromosome)

Halobiforma nitratireducens JCM 10879
EMA39868.1 (1227454)
AOMA01000078.1 (genomic DNA)

Natronobacterium gregoryi SP2
AFZ71963.1 (797304)
CP003377.1 (chromosome)

Natronobacterium texcoconense
SDQ34521.1 (1095778)
FNLC01000001.1 (genomic DNA)

Halopiger djelfimassiliensis
HG315686.1_858 (1293047)
HG315686.1 ()

Natrialbaceae haloarchaeon AArcht7
RQG99634.1 (1679086)
REFZ01000008.1 (genomic DNA)

Natrialbaceae haloarchaeon AArcht4
RQG97101.1 (1679083)
REGA01000002.1 (genomic DNA)

Halopiger xanaduensis SH 6
AEH38333.1 (797210)
CP002839.1 (chromosome)

Halopiger aswanensis
RKD95599.1 (148449)
RAPO01000002.1 (genomic DNA)

Natrinema ejinorense
PCR89691.1 (373386)
NXNI01000001.1 (genomic DNA)

Natrinema salaciae
SEQ56667.1 (1186196)
FOFD01000002.1 (genomic DNA)

Halopiger salifodinae
JROF01000001.1_152 (1202768)
JROF01000001.1 ()

Halopiger goleimassiliensis
HG315690.1_179 (1293048)
HG315690.1 ()

Natrialbaceae archaeon B1 Br10_E2g2
LKMK01000148.1_5 (1729458)
LKMK01000148.1 ()

Natrialbaceae archaeon Tc Br11_E2g14
LKND01000112.1_6 (1729462)
LKND01000112.1 ()

Halostella salina
RCIH01000005.1_78 (1547897)
RCIH01000005.1 ()

Halostella sp LT12
RCDI01000002.1_29 (2448456)
RCDI01000002.1 ()

Halobacteriales archaeon QS_9_68_17
PSQ27149.1 (1919191)
PXSA01000021.1 (genomic DNA)

Halorussus rarus
QPMJ01000001.1_799 (660515)
QPMJ01000001.1 ()

Halorussus sp HD8 51
QLVG01000050.1_15 (1710536)

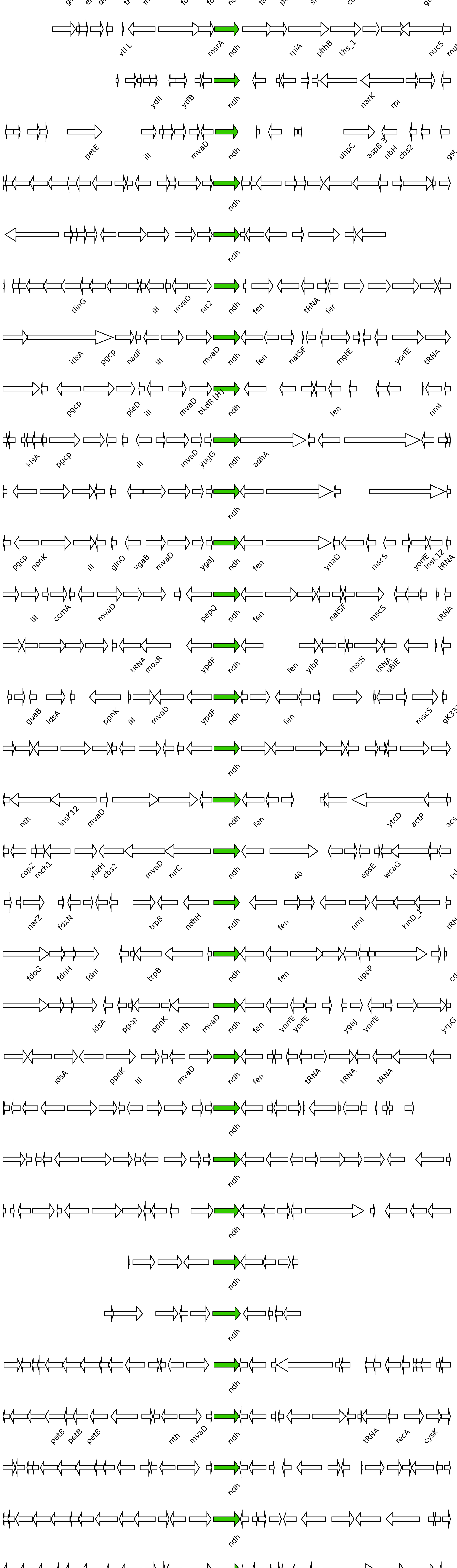

QLVG01000050.1 ()

halophilic archaeon
RBI61250.1 (29295)
QNGA01000004.1 (genomic DNA)

Haladaptatus paucihalophilus DX253
EFW92892.1 (797209)
AEMG01000006.1 (genomic DNA)

Haladaptatus litoreus
SIQ99980.1 (553468)
FTNO01000001.1 (genomic DNA)

Haladaptatus cibarius D43
JDTH01000002.1_958 (1455608)
JDTH01000002.1 ()

Halosimplex carlsbadense 2 9 1
ELZ22366.1 (797114)
AOIU01000036.1 (genomic DNA)

Halobacteriales archaeon QH_8_67_27
PSP71270.1 (1919163)
PXRC01000019.1 (genomic DNA)

Halosimplex sp YPL4
QWGG01000001.1_122 (1710538)
QWGG01000001.1 ()

Halobacteriales archaeon QS_5_68_33
PSQ08953.1 (1919179)
PXRS01000016.1 (genomic DNA)

Halobacteriales archaeon QH_1_68_42
PSP51805.1 (1919146)
PXQJ01000052.1 (genomic DNA)

Halobacteriales archaeon QH_10_67_22
PSP31957.1 (1919149)
PXQN01000059.1 (genomic DNA)

Halobacteriales archaeon QS_4_69_31
PSQ01195.1 (1919176)
PXRP01000048.1 (genomic DNA)

Halorientalis sp IM1011
AQL41730.1 (1932360)
CP019067.1 (chromosome)

Halorientalis persicus
SEN92573.1 (1367881)
FOCX01000006.1 (genomic DNA)

Halorientalis regularis
SDF85807.1 (660518)
FNBK01000010.1 (genomic DNA)

Halobacteriales archaeon QS_4_69_34
PSP87873.1 (1919177)
PXRN01000026.1 (genomic DNA)

Halococcus sediminicola
BBMP01000026.1_12 (1264579)
BBMP01000026.1 ()

Halococcus thailandensis JCM 13552
EMA50149.1 (1227457)
AOMF01000171.1 (genomic DNA)

Halococcus morrhuae DSM 1307
EMA40340.1 (931277)
AOMC01000152.1 (genomic DNA)

Halococcus agarilyticus
BAFM01000001.1_258 (1232219)
BAFM01000001.1 ()

Halobacteriales archaeon QH_9_66_26
PSP71082.1 (1919166)
PXRD01000087.1 (genomic DNA)

Halococcus salifodinae DSM 8989
EMA49788.1 (1227456)
AOME01000076.1 (genomic DNA)

Halococcus saccharolyticus DSM 5350
EMA43258.1 (1227455)
AOMD01000030.1 (genomic DNA)

Halococcus sp IIIV 5B
RJT06773.1 (2321230)
QZWE01000004.1 (genomic DNA)

Halococcus hamelinensis 100A6
AJRK01000218.1_2 (1132509)
AJRK01000218.1 ()

Haloprofundus marisrubri
KTG10333.1 (1514971)
LOPU01000018.1 (genomic DNA)

Haloprofundus sp NK23
QQRR01000001.1_342 (2283527)
QQRR01000001.1 ()

Haloferacaceae archaeon SYSU A9 0
PEND01000007.1_43 (1985678)
PEND01000007.1 ()

Halogranum gelatinilyticum
SDM73003.1 (660521)
FNHL01000003.1 (genomic DNA)

Halogranum salarium B 1
EJN61410.1 (1210908)
ALJD01000002.1 (genomic DNA)

Halogranum amylolyticum
SEO79735.1 (660520)
FODV01000005.1 (genomic DNA)

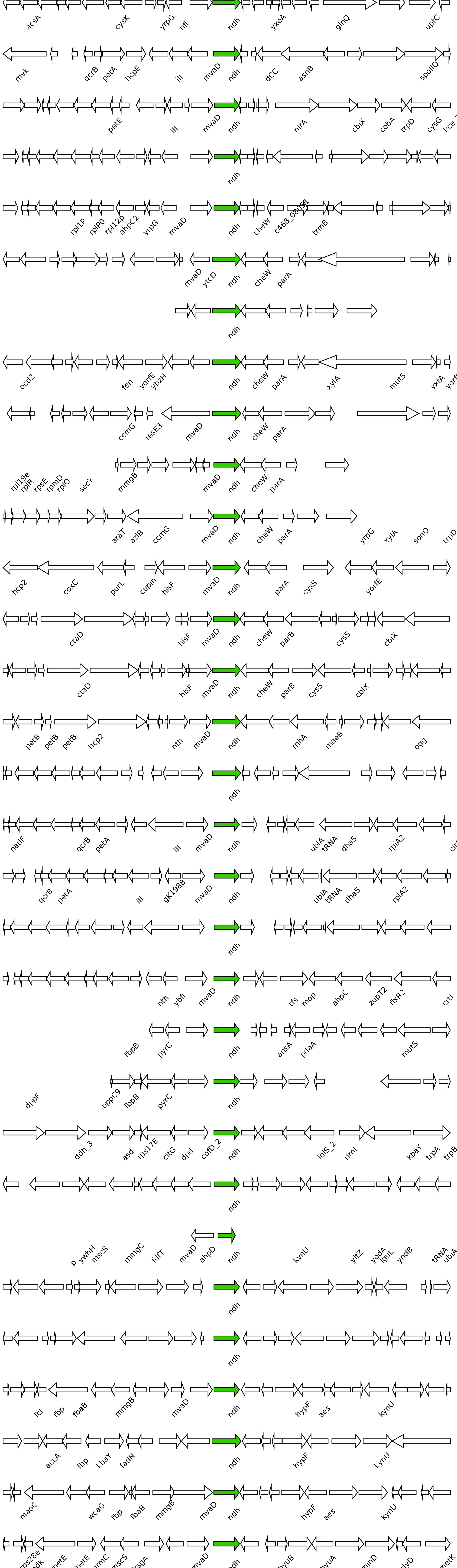

Haloplanus vescus
SDZ85005.1 (555874)
FNQT01000001.1 (genomic DNA)

Haloplanus aerogenes
RMB13549.1 (660522)
REFS01000004.1 (genomic DNA)

Haloplanus sp CBA1113
AXG06783.1 (1547899)
CP031150.1 (chromosome)

Haloplanus salinus
RCU46095.1 (1126245)
QPHM01000001.1 (genomic DNA)

Haloplanus natans DSM 17983
KE386573.1_2280 (926690)
KE386573.1 ()

Salinigranum rubrum
AUV81692.1 (755307)
CP026309.1 (chromosome)

Haloferax mediterranei ATCC 33500
AFK19195.1 (523841)
CP001868.2 (chromosome)

Haloferax mucosum ATCC BAA 1512
ELZ99005.1 (662479)
AOLN01000001.1 (genomic DNA)

Haloferax sulfurifontis ATCC BAA 897
ELZ97849.1 (662480)
AOLM01000004.1 (genomic DNA)

Haloferax sp SB3
POG54847.1 (1544719)
LOPW02000017.1 (genomic DNA)

Haloferax gibbonsii
AKU07488.1 (35746)
CP011947.1 (chromosome)

Haloferax volcanii DS2
ADE02821.1 (309800)
CP001956.1 (chromosome)

Haloferax sp SB29
KTG07610.1 (1544718)
LOPV01000712.1 (genomic DNA)

Haloferax elongans ATCC BAA 1513
ELZ86049.1 (1230453)
AOLK01000015.1 (genomic DNA)

Haloferax larsenii JCM 13917
ELZ82247.1 (1227460)
AOLI01000009.1 (genomic DNA)

Halogeometricum limi
SFR42183.1 (555875)
FOYS01000002.1 (genomic DNA)

Halogeometricum borinquense DSM 11551
ADQ67385.1 (469382)
CP001690.1 (chromosome)

Halogeometricum rufum
SFR47215.1 (553469)
FOYT01000001.1 (genomic DNA)

Halogeometricum pallidum JCM 14848
ELZ26494.1 (1227487)
AOIV01000044.1 (genomic DNA)

Halopelagius longus
RDI70558.1 (1236180)
QQST01000001.1 (genomic DNA)

Halopelagius inordinatus
SFG14038.1 (553467)
FOOQ01000001.1 (genomic DNA)

Halobellus sp Atlit 38R
RLM88384.1 (2282131)
QXIJ01000008.1 (genomic DNA)

Halobellus sp Atlit 31R
RLM53577.1 (2282130)
QPLR01000005.1 (genomic DNA)

Halobellus clavatus
SDY54475.1 (660517)
FNPB01000021.1 (genomic DNA)

Haloquadratum walsbyi J07HQW2
ERG96525.1 (1238425)
KE356561.1 (genomic)

Haloquadratum walsbyi C23
CCC39566.1 (768065)
FR746099.1 (genomic DNA)

Haloquadratum walsbyi J07HQW1
ERG90804.1 (1238424)
KE356560.1 (genomic)

Halorubraceae archaeon
PUNH01000394.1_12 (2093797)
PUNH01000394.1 ()

Haloferacaceae archaeon PL Br10_E2g29
LKMS01000007.1_22 (1727654)
LKMS01000007.1 ()

Halohasta litchfieldiae
ATW87389.1 (1073996)
CP024845.1 (chromosome)

Halonotius pteroides
RJX50128.1 (268735)
[illegible]

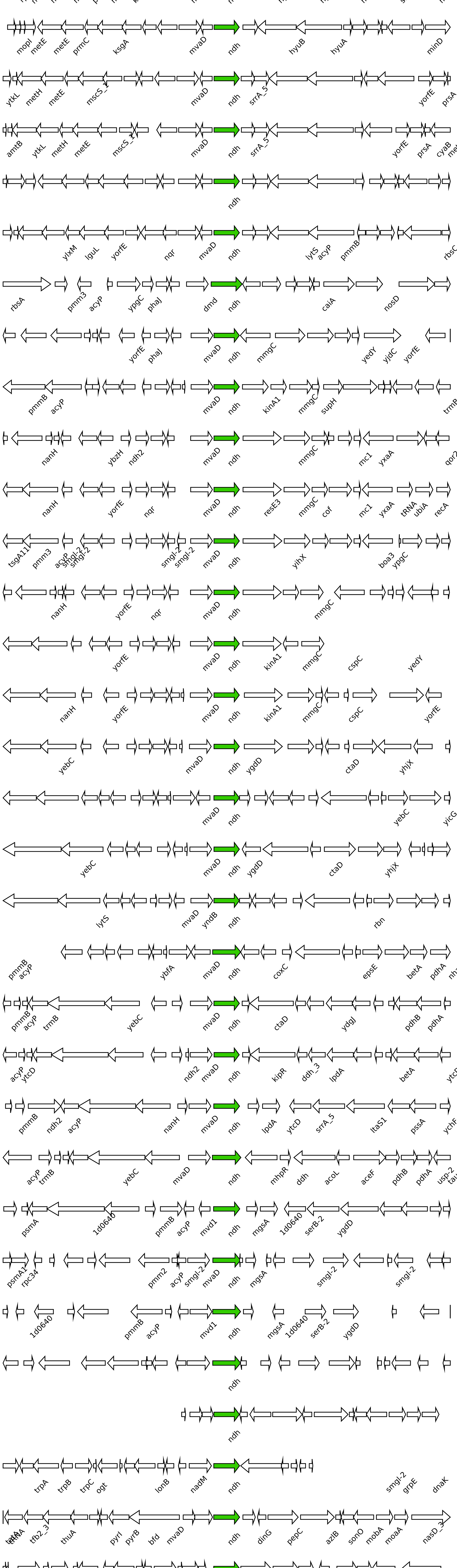

QMDW01000007.1 (genomic DNA)

Halonotius sp F13 13
RJX42669.1 (2216978)
QKNY01000013.1 (genomic DNA)

Halonotius sp J07HN4
ERH06314.1 (1070774)
KE356578.1 (genomic)

uncultured archaeon A07HN63
ESS08190.1 (1412873)
KI543219.1 (genomic)

Haloferacaceae archaeon B1 Br10_E2g22
LKML01000004.1_44 (1732021)
LKML01000004.1 ()

Candidatus Halobonum tyrrellensis G22
ESP88550.1 (1324957)
ASGZ01000028.1 (genomic DNA)

Halobacteriales archaeon QS_6_71_20
PSQ05558.1 (1919183)
PXRU01000019.1 (genomic DNA)

Halobaculum gomorrense
SHH60819.1 (43928)
FQWV01000010.1 (genomic DNA)

halophilic archaeon J07HB67
ERH13777.1 (1085029)
KE356582.1 (genomic)

Haloarchaeobius iranensis
SDM84046.1 (996166)
FNIA01000008.1 (genomic DNA)

Halalkalicoccus paucihalophilus
KYH25712.1 (1008153)
LTAZ01000005.1 (genomic)

Halalkalicoccus jeotgali B3
ADJ15615.1 (795797)
CP002062.1 (chromosome)

Halomicrobium zhouii
SFR94247.1 (767519)
FOZK01000001.1 (genomic DNA)

Halomicrobium mukohataei DSM 12286
ACV48235.1 (485914)
CP001688.1 (chromosome)

Halobacteriales archaeon QS_4_62_28
PSP94387.1 (1919173)
PXRO01000027.1 (genomic DNA)

Halobacteriales archaeon QH_8_67_36
PSP60863.1 (1919164)
PXQZ01000105.1 (genomic DNA)

Halobacteriales archaeon QS_6_64_34
PSP85792.1 (1919182)
PXRT01000002.1 (genomic DNA)

Haloarcula sp CBA1127
BCNB01000006.1_1714 (1765055)
BCNB01000006.1 ()

Haloarcula marismortui ATCC 43049
AAV46410.1 (272569)
AY596297.1 (genomic)

Haloarcula japonica DSM 6131
EMA32811.1 (1227453)
AOLY01000009.1 (genomic DNA)

Haloarcula argentinensis DSM 12282
EMA18525.1 (1230451)
AOLX01000027.1 (genomic DNA)

Haloarcula vallismortis ATCC 29715
EMA01416.1 (662477)
AOLQ01000067.1 (genomic DNA)

Haloarcula hispanica ATCC 33960
AEM57655.1 (634497)
CP002921.1 (chromosome)

Haloarcula taiwanensis
AUG47840.1 (1932004)
CP019154.1 (chromosome)

Haloarcula rubripromontorii
KOX91936.1 (1705562)
LIUF01000005.1 (genomic DNA)

Halobacteriales archaeon QS_5_70_15
PSQ10527.1 (1919180)
PXRQ01000078.1 (genomic DNA)

Halobacteriales archaeon QS_4_70_19
PSP97626.1 (1919178)
PXRM01000029.1 (genomic DNA)

Halobacteriales archaeon QH_10_70_21
PSP44306.1 (1919150)
PXQM01000048.1 (genomic DNA)

Halobacteriales archaeon QS_7_69_60
PSQ17796.1 (1919185)
PXRW01000012.1 (genomic DNA)

Halobacteriales archaeon SW_5_68_122
PSQ40470.1 (1919199)
PXSL01000134.1 (genomic DNA)

Halobacteriales archaeon QS_9_68_42
PSQ43018.1 (1919192)
PXSE01000040.1 (genomic DNA)

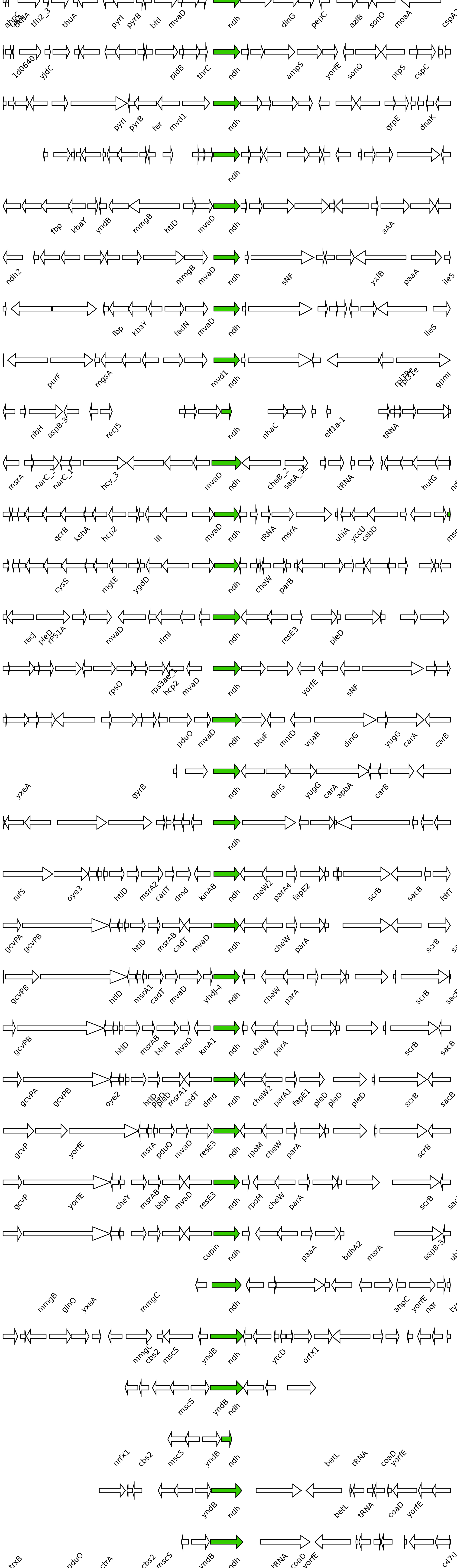

Halobacteriales archaeon QH_7_69_31
PSP49252.1 (1919161)
PXQY01000056.1 (genomic DNA)

Halobacteriales archaeon QS_1_69_70
PSP67846.1 (1919171)
PXRI01000015.1 (genomic DNA)

Halobacteriales archaeon QH_7_66_36
PSP57249.1 (1919158)
PXQV01000014.1 (genomic DNA)

Halobacteriales archaeon QH_7_65_31
PSP39074.1 (1919157)
PXQU01000007.1 (genomic DNA)

Natronomonas sp CBA1133
RNJ26035.1 (2487041)
RJJC01000001.1 (genomic DNA)

Halobacterium jilantaiense
SEW01857.1 (355548)
FOJA01000001.1 (genomic DNA)

Halobacterium sp DL1
AHG03390.1 (751944)
CP007060.1 (chromosome)

Halobacterium sp CBA1132
BCMZ01000001.1_652 (1765057)
BCMZ01000001.1 ()

Halobacterium hubeiense
CQH51431.1 (1407499)
LN831302.1 (genomic DNA)

uncultured archaeon A07HB70
ESS07096.1 (1412872)
KI543174.1 (genomic)

Halobacteriales archaeon QH_6_64_20
PSP43687.1 (1919154)
PXQR01000040.1 (genomic DNA)

Halobacteriales archaeon QH_8_64_26
PSP62763.1 (1919162)
PXRA01000069.1 (genomic DNA)

Halobacteriales archaeon QS_3_64_16
PSP76707.1 (1919172)
PXRJ01000001.1 (genomic DNA)

Halarchaeum sp CBA1220
RNH92182.1 (1853682)
RIZB01000001.1 (genomic DNA)

Halarchaeum acidiphilum MH1 52 1
BANO01000137.1_8 (1261545)
BANO01000137.1 ()

halophilic archaeon DL31
AEN06245.1 (756883)
CP002988.1 (chromosome)

Halolamina rubra
BBJN01000035.1_44 (1380430)
BBJN01000035.1 ()

Halolamina pelagica
KPN31517.1 (699431)
LGUC01000001.1 (genomic DNA)

Halolamina sp CBA1230
OPF21972.1 (1853690)
MWUK01000003.1 (genomic DNA)

Halolamina sediminis
CVUA01000001.1_1777 (1480675)
CVUA01000001.1 ()

archaeon AArc Sl
AUX08423.1 (2055893)
CP025066.1 (chromosome)

Natrialba hulunbeirensis JCM 10989
ELY87701.1 (1227493)
AOIM01000041.1 (genomic DNA)

Natrialba magadii ATCC 43099
ADD06783.1 (547559)
CP001932.1 (chromosome)

Natrialba sp SSL1
OIB56998.1 (1869245)
MASN01000035.1 (genomic DNA)

Natrialba chahannaoensis JCM 10990
ELY99663.1 (1227492)
AOIN01000056.1 (genomic DNA)

Halovivax asiaticus JCM 14624
ELZ12681.1 (1227490)
AOIQ01000008.1 (genomic DNA)

Halovivax ruber XH 70
AGB15398.1 (797302)
CP003050.1 (chromosome)

Salinarchaeum sp Harcht Bsk1
AGN02706.1 (1333523)
CP005962.1 (chromosome)

Halobacteriales archaeon SW_7_68_16
PSQ42007.1 (1919204)
PXSO01000101.1 (genomic DNA)

Halorhabdus tiamatea SARL4B
ERJ07122.1 (1033806)
AFNT02000006.1 (genomic DNA)

Halorhabdus utahensis DSM 12940
ACV10876.1 (519442)
CP001687.1 (chromosome)

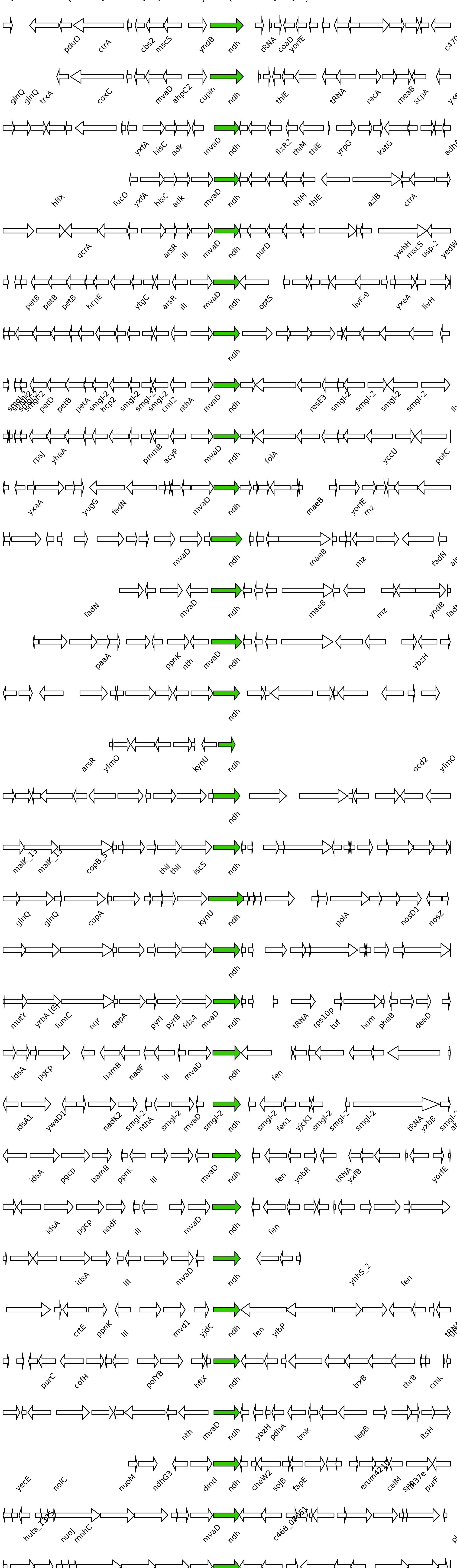

[illegible] (chromosome)

Halobacteriales archaeon QS_7_69_60
PSQ15729.1 (1919185)
PXRW01000043.1 (genomic DNA)

Natrialbaceae archaeon Tc Br11_E2g8
LKNG01000035.1_7 (1729464)
LKNG01000035.1 ()

Halobacteriales archaeon SW_7_68_16
PSQ42885.1 (1919204)
PXSO01000075.1 (genomic DNA)

Halobacteriales archaeon QH_8_64_26
PSP65595.1 (1919162)
PXRA01000007.1 (genomic DNA)

Halobacteriales archaeon QH_6_64_20
PSP43156.1 (1919154)
PXQR01000061.1 (genomic DNA)

Haladaptatus sp R4
KZN22691.1 (1679489)
LWHG01000032.1 (genomic DNA)

Halogeometricum limi
SFR64969.1 (555875)
FOYS01000005.1 (genomic DNA)

Halogeometricum rufum
SFR66240.1 (553469)
FOYT01000003.1 (genomic DNA)

Halogeometricum pallidum JCM 14848
ELZ33874.1 (1227487)
AOIV01000006.1 (genomic DNA)

Halogranum salarium B 1
EJN59459.1 (1210908)
ALJD01000004.1 (genomic DNA)

Halobacteriales archaeon QS_7_68_65
PSQ13147.1 (1919184)
PXRV01000056.1 (genomic DNA)

Halococcus sediminicola
BBMP01000006.1_208 (1264579)
BBMP01000006.1 ()

Halococcus morrhuae DSM 1307
EMA42391.1 (931277)
AOMC01000131.1 (genomic DNA)

Halococcus thailandensis JCM 13552
EMA49475.1 (1227457)
AOMF01000174.1 (genomic DNA)

Halobacteriales archaeon QS_1_68_20
PSP80030.1 (1919169)
PXRE01000006.1 (genomic DNA)

Halobacteriales archaeon QS_8_69_26
PSQ17350.1 (1919187)
PXRX01000024.1 (genomic DNA)

Halobacteriales archaeon SW_7_71_33
PSQ56586.1 (1919205)
PXSP01000133.1 (genomic DNA)

Salinarchaeum sp Harcht Bsk1
AGM99964.1 (1333523)
CP005962.1 (chromosome)

Halostella salina
RCIH01000003.1_69 (1547897)
RCIH01000003.1 ()

Halostella sp LT12
RCDI01000003.1_188 (2448456)
RCDI01000003.1 ()

Halobacteriales archaeon QS_9_68_17
PSQ26377.1 (1919191)
PXSA01000032.1 (genomic DNA)

Halorussus sp HD8 51
KZ859503.1_50 (1710536)
KZ859503.1 ()

Halobacteriales archaeon QS_1_67_19
PSP55224.1 (1919167)
PXRF01000018.1 (genomic DNA)

Halorussus rarus
QPMJ01000001.1_933 (660515)
QPMJ01000001.1 ()

Halobacteriales archaeon SW_6_65_15
PSQ49114.1 (1919201)
PXSM01000059.1 (genomic DNA)

halophilic archaeon
RBI61209.1 (29295)
QNGA01000004.1 (genomic DNA)

Haladaptatus litoreus
SIR01226.1 (553468)
FTNO01000001.1 (genomic DNA)

Haladaptatus cibarius D43
JDTH01000002.1_914 (1455608)
JDTH01000002.1 ()

Haladaptatus paucihalophilus DX253
EFW92859.1 (797209)
AEMG01000006.1 (genomic DNA)

Haladaptatus sp R4
KZN24045.1 (1679489)
LWHG01000021.1 (genomic DNA)

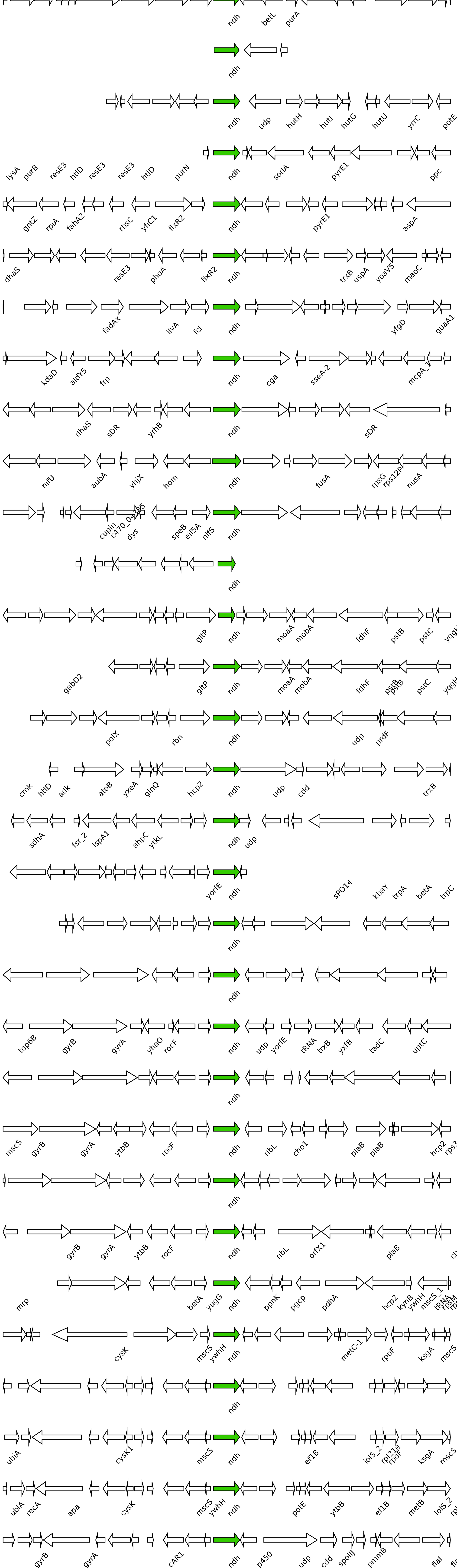

Natronoarchaeum philippinense
SNZ04424.1 (558529)
OBEJ01000001.1 (genomic DNA)

Halalkalicoccus sp GSM28
RDQG01000007.1_2 (2480581)
RDQG01000007.1 ()

Halalkalicoccus jeotgali B3
ADJ15596.1 (795797)
CP002062.1 (chromosome)

Halalkalicoccus paucihalophilus
KYH25700.1 (1008153)
LTAZ01000005.1 (genomic)

Halobacteriales archaeon QH_6_64_20
PSP45064.1 (1919154)
PXQR01000004.1 (genomic DNA)

Halobacteriales archaeon QS_3_64_16
PSP73868.1 (1919172)
PXRJ01000034.1 (genomic DNA)

Halobacteriales archaeon QH_8_64_26
PSP60721.1 (1919162)
PXRA01000110.1 (genomic DNA)

Halobacteriales archaeon QS_8_65_32
PSQ19756.1 (1919186)
PXRY01000122.1 (genomic DNA)

Halococcus sp IIIV 5B
RJT02526.1 (2321230)
QZWE01000019.1 (genomic DNA)

Halococcus hamelinensis 100A6
AJRK01000156.1_1 (1132509)
AJRK01000156.1 ()

Halococcus sediminicola
BBMP01000011.1_104 (1264579)
BBMP01000011.1 ()

Halococcus thailandensis JCM 13552
EMA51168.1 (1227457)
AOMF01000166.1 (genomic DNA)

Halococcus morrhuae DSM 1307
EMA39371.1 (931277)
AOMC01000161.1 (genomic DNA)

Halobacteriales archaeon QS_7_68_65
PSQ12433.1 (1919184)
PXRV01000087.1 (genomic DNA)

Halococcus agarilyticus
BAFM01000002.1_270 (1232219)
BAFM01000002.1 ()

Halococcus salifodinae DSM 8989
EMA49320.1 (1227456)
AOME01000078.1 (genomic DNA)

Halococcus saccharolyticus DSM 5350
EMA44186.1 (1227455)
AOMD01000025.1 (genomic DNA)

Halobacteriales archaeon QH_9_66_26
PSP73468.1 (1919166)
PXRD01000023.1 (genomic DNA)

Halovivax ruber XH 70
AGB16060.1 (797302)
CP003050.1 (chromosome)

Halovivax asiaticus JCM 14624
ELZ13454.1 (1227490)
AOIQ01000006.1 (genomic DNA)

Natronococcus amylolyticus DSM 10524
ELY54319.1 (1227497)
AOIB01000037.1 (genomic DNA)

Natronococcus occultus SP4
AGB39170.1 (694430)
CP003929.1 (chromosome)

Natronococcus jeotgali DSM 18795
ELY58477.1 (1227498)
AOIA01000114.1 (genomic DNA)

Natronolimnobius innermongolicus JCM 12255
ELY57517.1 (1227499)
AOHZ01000041.1 (genomic DNA)

Haloterrigena turkmenica DSM 5511
ADB61145.1 (543526)
CP001860.1 (chromosome)

Haloterrigena sp ZY19
RQWN01000001.1_402 (2491022)
RQWN01000001.1 ()

Haloterrigena salina JCM 13891
ELZ18067.1 (1227488)
AOIS01000037.1 (genomic DNA)

Haloterrigena saccharevitans
OLZ40625.1 (301967)
LWLN01000001.1 (genomic DNA)

Haloterrigena mahii
OAQ54217.1 (1416969)
JHUT02000001.1 (genomic DNA)

Natrinema pellirubrum DSM 15624
AGB31405.1 (797303)
CP003372.1 (chromosome)

Natrinema sp CBA1119
PGF17467.1 (1608465)
PDBS01000001.1 (genomic DNA)

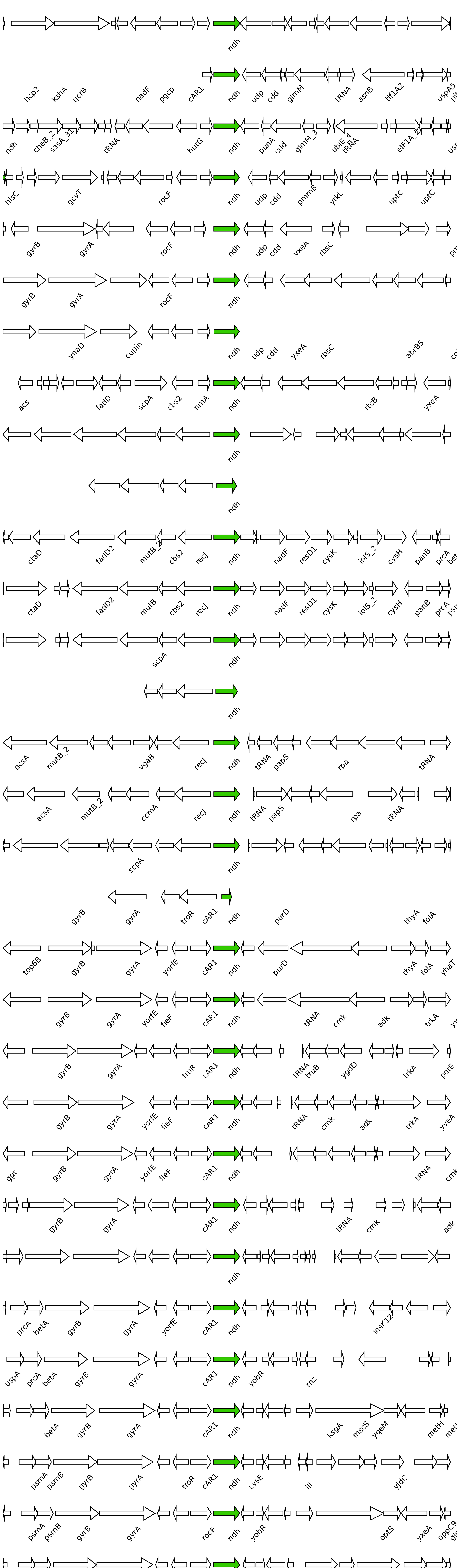

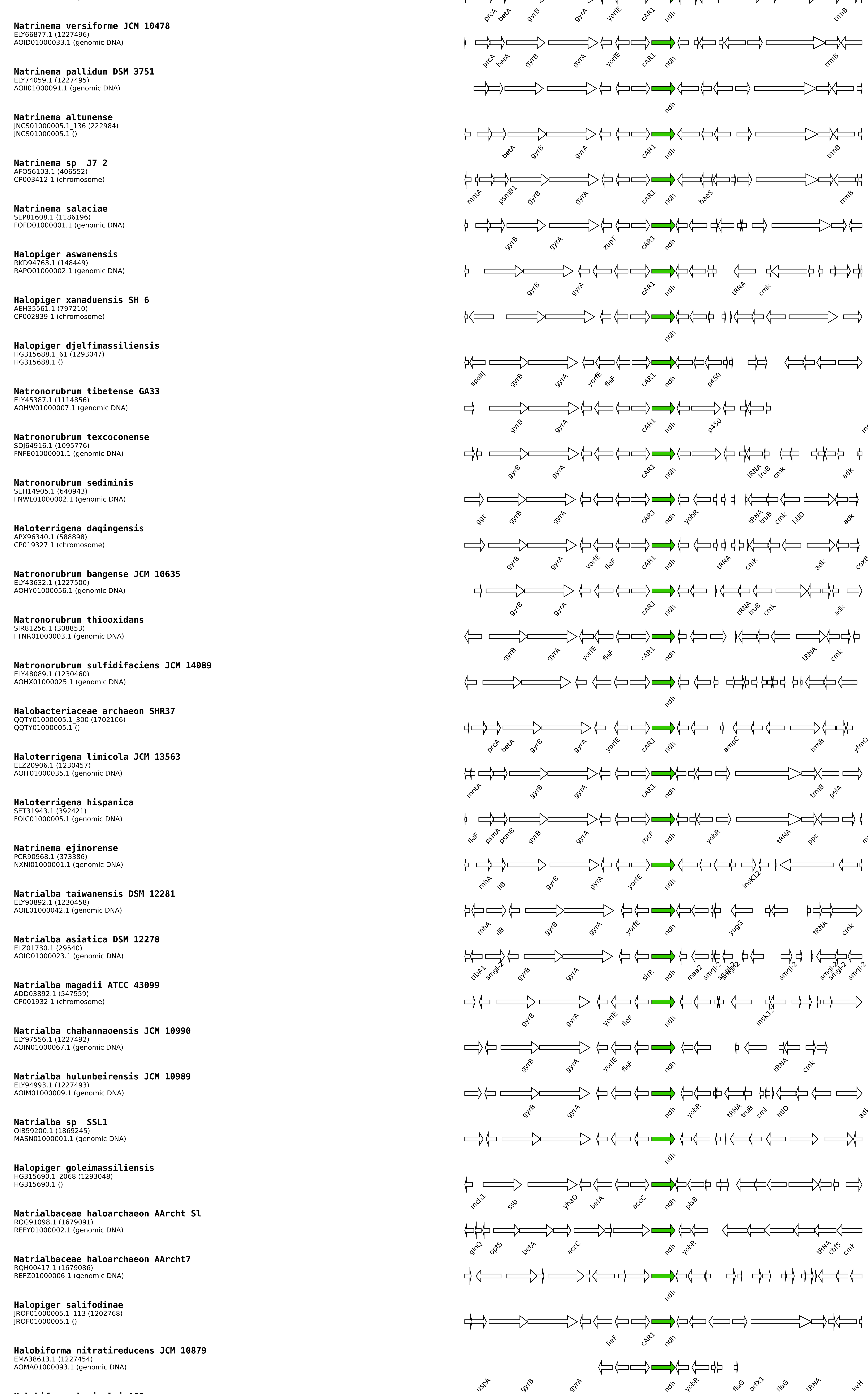

Natrinema versiforme JCM 10478
ELY66877.1 (1227496)
AOID01000033.1 (genomic DNA)
Natrinema pallidum DSM 3751
ELY74059.1 (1227495)
AOII01000091.1 (genomic DNA)
Natrinema altunense
JNCS01000005.1_136 (222984)
JNCS01000005.1 ()
Natrinema sp J7 2
AFO56103.1 (406552)
CP003412.1 (chromosome)
Natrinema salaciae
SEP81608.1 (1186196)
FOFD01000001.1 (genomic DNA)
Halopiger aswanensis
RKD94763.1 (148449)
RAPO01000002.1 (genomic DNA)
Halopiger xanaduensis SH 6
AEH35561.1 (797210)
CP002839.1 (chromosome)
Halopiger djelfimassiliensis
HG315688.1_61 (1293047)
HG315688.1 ()
Natronorubrum tibetense GA33
ELY45387.1 (1114856)
AOHW01000007.1 (genomic DNA)
Natronorubrum texcoconense
SDJ64916.1 (1095776)
FNFE01000001.1 (genomic DNA)
Natronorubrum sediminis
SEH14905.1 (640943)
FNWL01000002.1 (genomic DNA)
Haloterrigena daqingensis
APX96340.1 (588898)
CP019327.1 (chromosome)
Natronorubrum bangense JCM 10635
ELY43632.1 (1227500)
AOHY01000056.1 (genomic DNA)
Natronorubrum thiooxidans
SIR81256.1 (308853)
FTNR01000003.1 (genomic DNA)
Natronorubrum sulfidifaciens JCM 14089
ELY48089.1 (1230460)
AOHX01000025.1 (genomic DNA)
Halobacteriaceae archaeon SHR37
QQTY01000005.1_300 (1702106)
QQTY01000005.1 ()
Haloterrigena limicola JCM 13563
ELZ20906.1 (1230457)
AOIT01000035.1 (genomic DNA)
Haloterrigena hispanica
SET31943.1 (392421)
FOIC01000005.1 (genomic DNA)
Natrinema ejinorense
PCR90968.1 (373386)
NXNI01000001.1 (genomic DNA)
Natrialba taiwanensis DSM 12281
ELY90892.1 (1230458)
AOIL01000042.1 (genomic DNA)
Natrialba asiatica DSM 12278
ELZ01730.1 (29540)
AOIO01000023.1 (genomic DNA)
Natrialba magadii ATCC 43099
ADD03892.1 (547559)
CP001932.1 (chromosome)
Natrialba chahannaoensis JCM 10990
ELY97556.1 (1227492)
AOIN01000067.1 (genomic DNA)
Natrialba hulunbeirensis JCM 10989
ELY94993.1 (1227493)
AOIM01000009.1 (genomic DNA)
Natrialba sp SSL1
OIB59200.1 (1869245)
MASN01000001.1 (genomic DNA)
Halopiger goleimassiliensis
HG315690.1_2068 (1293048)
HG315690.1 ()
Natrialbaceae haloarchaeon AArcht Sl
RQG91098.1 (1679091)
REFY01000002.1 (genomic DNA)
Natrialbaceae haloarchaeon AArcht7
RQH00417.1 (1679086)
REFZ01000006.1 (genomic DNA)
Halopiger salifodinae
JROF01000005.1_113 (1202768)
JROF01000005.1 ()
Halobiforma nitratireducens JCM 10879
EMA38613.1 (1227454)
AOMA01000093.1 (genomic DNA)

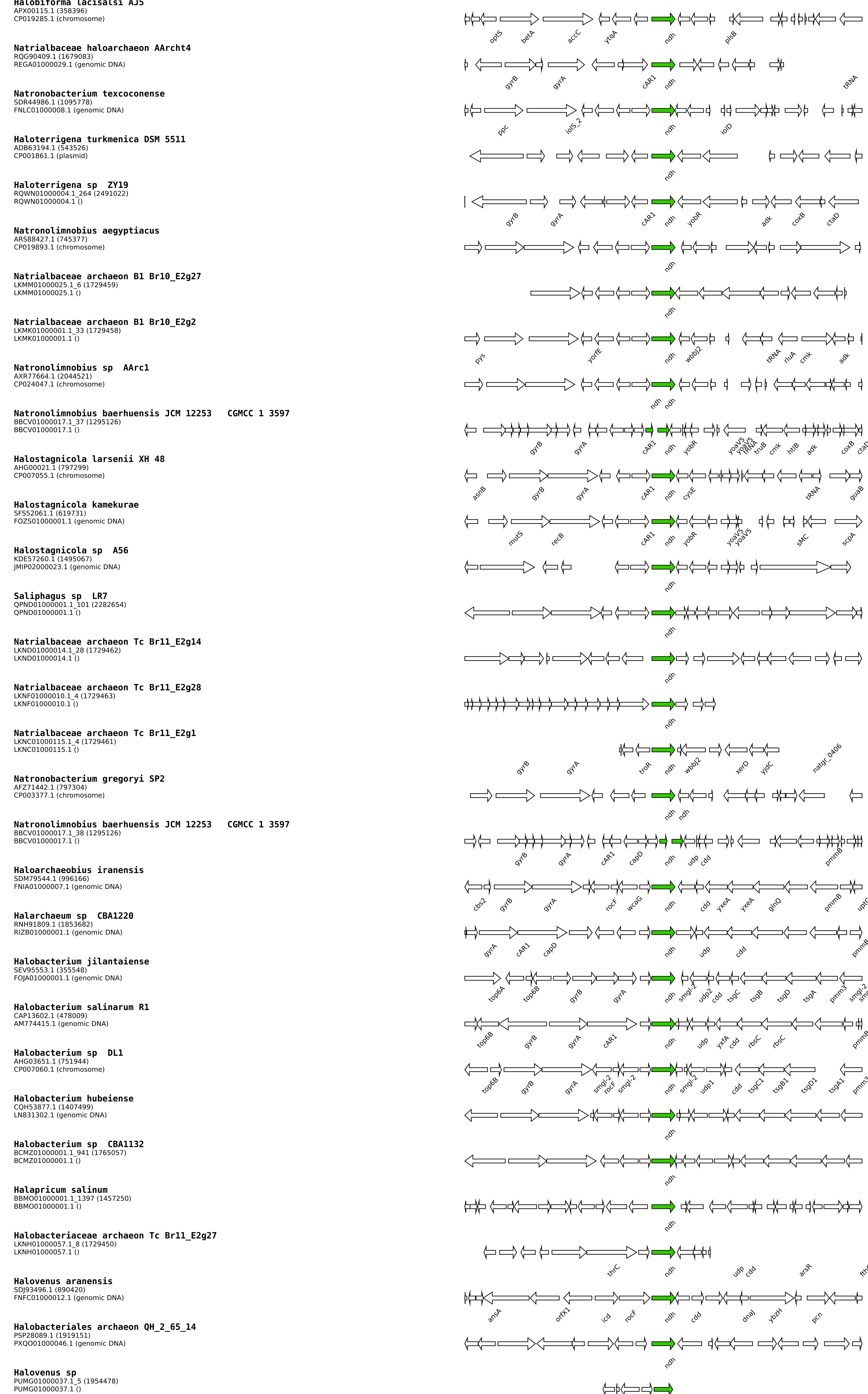
Halobiforma lacisalsi AJ5
APX00115.1 (358396)
CP019285.1 (chromosome)
optS betA accC ytqA ndh plsB
Natrialbaceae haloarchaeon AArcht4
RQG90409.1 (1679083)
REGA01000029.1 (genomic DNA)
gyrB gyrA cAR1 ndh tRNA
Natronobacterium texcoconense
SDR44986.1 (1095778)
FNLC01000008.1 (genomic DNA)
ppc iolS_2 ndh iolD
Haloterrigena turkmenica DSM 5511
ADB63194.1 (543526)
CP001861.1 (plasmid)
ndh
Haloterrigena sp ZY19
RQWN01000004.1_264 (2491022)
RQWN01000004.1 ()
gyrB gyrA cAR1 ndh yobR adk coxB ctaD
Natronolimnobius aegyptiacus
ARS88427.1 (745377)
CP019893.1 (chromosome)
ndh
Natrialbaceae archaeon B1 Br10_E2g27
LKMM01000025.1_6 (1729459)
LKMM01000025.1 ()
ndh
Natrialbaceae archaeon B1 Br10_E2g2
LKMK01000001.1_33 (1729458)
LKMK01000001.1 ()
pys yorfE ndh wbbJ2 tRNA rluA cmk adk
Natronolimnobius sp AArc1
AXR77664.1 (2044521)
CP024047.1 (chromosome)
ndh ndh
Natronolimnobius baerhuensis JCM 12253 CGMCC 1 3597
BBCV01000017.1_37 (1295126)
BBCV01000017.1 ()
gyrB gyrA cAR1 ndh yobR yoaV5 yoaV5 tRNA truB cmk htlB adk coxB ctaD
Halostagnicola larsenii XH 48
AHG00021.1 (797299)
CP007055.1 (chromosome)
asnB gyrB gyrA cAR1 ndh cysE tRNA guaB
Halostagnicola kamekurae
SFS52061.1 (619731)
FOZS01000001.1 (genomic DNA)
mutS recB cAR1 ndh yobR yoaV5 yoaV5 sMC scpA
Halostagnicola sp A56
KDE57260.1 (1495067)
JMIP02000023.1 (genomic DNA)
ndh
Saliphagus sp LR7
QPND01000001.1_101 (2282654)
QPND01000001.1 ()
ndh
Natrialbaceae archaeon Tc Br11_E2g14
LKND01000014.1_28 (1729462)
LKND01000014.1 ()
ndh
Natrialbaceae archaeon Tc Br11_E2g28
LKNF01000010.1_4 (1729463)
LKNF01000010.1 ()
ndh
Natrialbaceae archaeon Tc Br11_E2g1
LKNC01000115.1_4 (1729461)
LKNC01000115.1 ()
gyrB gyrA troR ndh wbbJ2 xerD yjdC natgr_0406
Natronobacterium gregoryi SP2
AFZ71442.1 (797304)
CP003377.1 (chromosome)
ndh ndh
Natronolimnobius baerhuensis JCM 12253 CGMCC 1 3597
BBCV01000017.1_38 (1295126)
BBCV01000017.1 ()
gyrB gyrA cAR1 capD ndh udp cdd pmmB
Haloarchaeobius iranensis
SDM79544.1 (996166)
FNIA01000007.1 (genomic DNA)
cbs2 gyrB gyrA rocF wcaG ndh cdd yxeA yxeA glnQ pmmB uptC
Halarchaeum sp CBA1220
RNH91809.1 (1853682)
RIZB01000001.1 (genomic DNA)
gyrA cAR1 capD ndh udp cdd pmmB
Halobacterium jilantaiense
SEV95553.1 (355548)
FOJA01000001.1 (genomic DNA)
top6A top6B gyrB gyrA ndh smgl-2 udp2 cdd tsgC tsgB tsgD tsgA pmm3 smgl-2
Halobacterium salinarum R1
CAP13602.1 (478009)
AM774415.1 (genomic DNA)
top6B gyrB gyrA cAR1 ndh udp yxfA cdd rbsC rbsC pmmB
Halobacterium sp DL1
AHG03651.1 (751944)
CP007060.1 (chromosome)
top6B gyrB gyrA smgl-2 rocF smgl-2 ndh smgl-2 udp1 cdd tsgC1 tsgB1 tsgD1 tsgA1 pmm3
Halobacterium hubeiense
CQH53877.1 (1407499)
LN831302.1 (genomic DNA)
ndh
Halobacterium sp CBA1132
BCMZ01000001.1_941 (1765057)
BCMZ01000001.1 ()
ndh
Halapricum salinum
BBMO01000001.1_1397 (1457250)
BBMO01000001.1 ()
ndh
Halobacteriaceae archaeon Tc Br11_E2g27
LKNH01000057.1_8 (1729450)
LKNH01000057.1 ()
thrC ndh udp cdd arsR
Halovenus aranensis
SDJ93496.1 (890420)
FNFC01000012.1 (genomic DNA)
ansA orfX1 icd rocF ndh cdd dnaJ ybzH pcn
Halobacteriales archaeon QH_2_65_14
PSP28089.1 (1919151)
PXQO01000046.1 (genomic DNA)
ndh
Halovenus sp
PUMG01000037.1_5 (1954478)
PUMG01000037.1 ()

Halobacteriales archaeon QH_10_65_19
PSP24669.1 (1919147)
PXQK01000038.1 (genomic DNA)

Halobacteriales archaeon QH_6_66_25
PSP48067.1 (1919155)
PXQS01000004.1 (genomic DNA)

Halobacteriales archaeon SW_7_65_23
PSQ47956.1 (1919203)
PXSQ01000069.1 (genomic DNA)

Halobacteriales archaeon QS_1_68_17
PSP82884.1 (1919168)
PXRG01000030.1 (genomic DNA)

Halorientalis sp IM1011
AQL41335.1 (1932360)
CP019067.1 (chromosome)

Halorientalis persicus
SEO13911.1 (1367881)
FOCX01000008.1 (genomic DNA)

Halorientalis regularis
SDE78893.1 (660518)
FNBK01000001.1 (genomic DNA)

Halobacteriales archaeon QS_6_64_34
PSP85608.1 (1919182)
PXRT01000004.1 (genomic DNA)

Halobacteriales archaeon QH_8_67_36
PSP60541.1 (1919164)
PXQZ01000114.1 (genomic DNA)

Haloarcula vallismortis ATCC 29715
EMA10183.1 (662477)
AOLQ01000012.1 (genomic DNA)

Haloarcula vallismortis ATCC 29715
EMA09092.1 (662477)
AOLQ01000025.1 (genomic DNA)

Haloarcula taiwanensis
AUG47056.1 (1932004)
CP019154.1 (chromosome)

Haloarcula taiwanensis
AUG47673.1 (1932004)
CP019154.1 (chromosome)

Haloarcula argentinensis DSM 12282
EMA23372.1 (1230451)
AOLX01000015.1 (genomic DNA)

Haloarcula sp CBA1127
BCNB01000006.1_1886 (1765055)
BCNB01000006.1 ()

Haloarcula marismortui ATCC 43049
AAV45481.1 (272569)
AY596297.1 (genomic)

Haloarcula marismortui ATCC 43049
AAV46237.1 (272569)
AY596297.1 (genomic)

Haloarcula sp CBA1127
BCNB01000006.1_2599 (1765055)
BCNB01000006.1 ()

Haloarcula argentinensis DSM 12282
EMA18713.1 (1230451)
AOLX01000027.1 (genomic DNA)

Haloarcula rubripromontorii
KOX91548.1 (1705562)
LIUF01000009.1 (genomic DNA)

Haloarcula rubripromontorii
KOX92106.1 (1705562)
LIUF01000005.1 (genomic DNA)

Haloarcula hispanica ATCC 33960
AEM57492.1 (634497)
CP002921.1 (chromosome)

Haloarcula hispanica ATCC 33960
AEM56800.1 (634497)
CP002921.1 (chromosome)

Haloarcula japonica DSM 6131
EMA29574.1 (1227453)
AOLY01000037.1 (genomic DNA)

Halomicrobium zhouii
SFS06406.1 (767519)
FOZK01000003.1 (genomic DNA)

Halomicrobium mukohataei DSM 12286
ACV48842.1 (485914)
CP001688.1 (chromosome)

Halobacteriales archaeon QS_4_62_28
PSP95651.1 (1919173)
PXRO01000006.1 (genomic DNA)

Halosimplex sp YPL4
QWGG01000001.1_1022 (1710538)
QWGG01000001.1 ()

Halobacteriales archaeon QH_8_67_27
PSP71512.1 (1919163)
PXRC01000009.1 (genomic DNA)

Halosimplex carlsbadense 2 9 1
ELZ24539.1 (797114)
AOIU01000029.1 (genomic DNA)

Halobacteriales archaeon QS_4_69_31

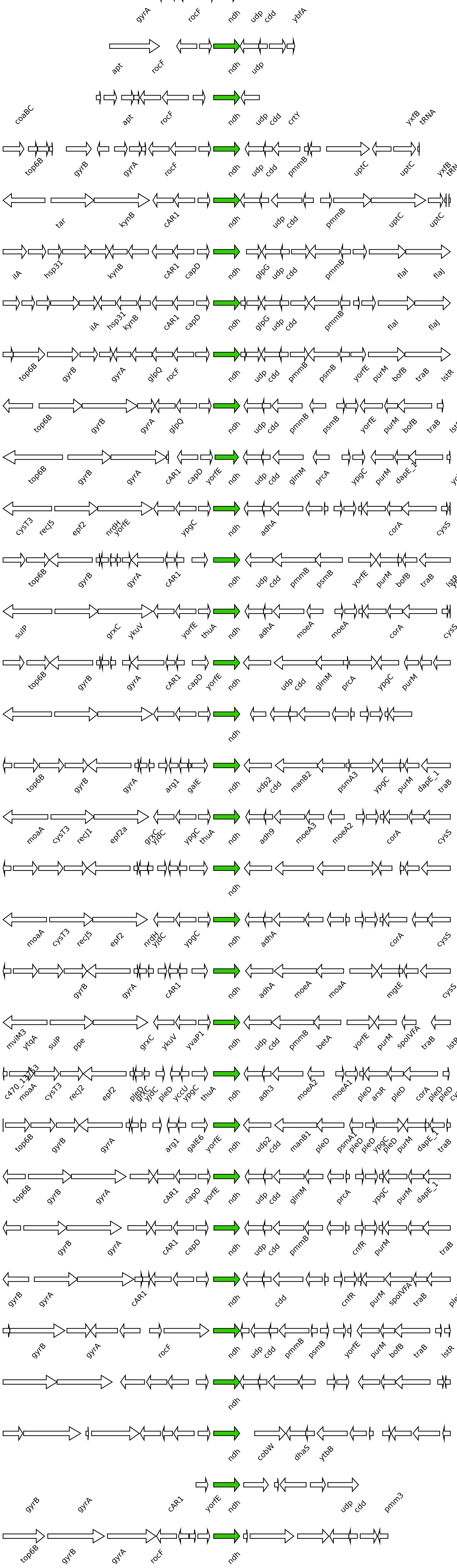

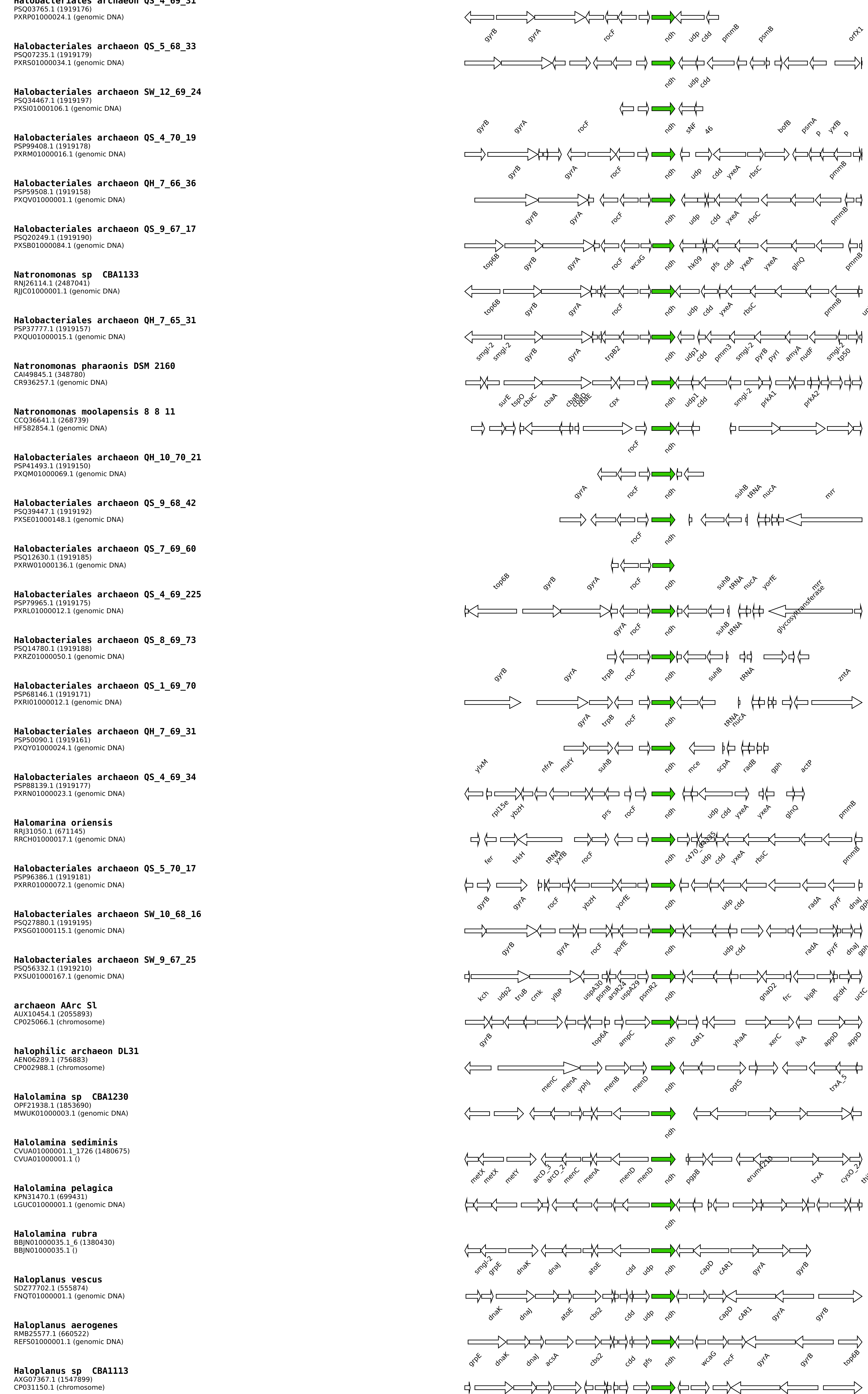

Halobacteriales archaeon QS_4_69_31
PSQ03765.1 (1919176)
PXRP01000024.1 (genomic DNA)
Halobacteriales archaeon QS_5_68_33
PSQ07235.1 (1919179)
PXRS01000034.1 (genomic DNA)
Halobacteriales archaeon SW_12_69_24
PSQ34467.1 (1919197)
PXSI01000106.1 (genomic DNA)
Halobacteriales archaeon QS_4_70_19
PSP99408.1 (1919178)
PXRM01000016.1 (genomic DNA)
Halobacteriales archaeon QH_7_66_36
PSP59508.1 (1919158)
PXQV01000001.1 (genomic DNA)
Halobacteriales archaeon QS_9_67_17
PSQ20249.1 (1919190)
PXSB01000084.1 (genomic DNA)
Natronomonas sp CBA1133
RNJ26114.1 (2487041)
RJJC01000001.1 (genomic DNA)
Halobacteriales archaeon QH_7_65_31
PSP37777.1 (1919157)
PXQU01000015.1 (genomic DNA)
Natronomonas pharaonis DSM 2160
CAI49845.1 (348780)
CR936257.1 (genomic DNA)
Natronomonas moolapensis 8 8 11
CCQ36641.1 (268739)
HF582854.1 (genomic DNA)
Halobacteriales archaeon QH_10_70_21
PSP41493.1 (1919150)
PXQM01000069.1 (genomic DNA)
Halobacteriales archaeon QS_9_68_42
PSQ39447.1 (1919192)
PXSE01000148.1 (genomic DNA)
Halobacteriales archaeon QS_7_69_60
PSQ12630.1 (1919185)
PXRW01000136.1 (genomic DNA)
Halobacteriales archaeon QS_4_69_225
PSP79965.1 (1919175)
PXRL01000012.1 (genomic DNA)
Halobacteriales archaeon QS_8_69_73
PSQ14780.1 (1919188)
PXRZ01000050.1 (genomic DNA)
Halobacteriales archaeon QS_1_69_70
PSP68146.1 (1919171)
PXRI01000012.1 (genomic DNA)
Halobacteriales archaeon QH_7_69_31
PSP50090.1 (1919161)
PXQY01000024.1 (genomic DNA)
Halobacteriales archaeon QS_4_69_34
PSP88139.1 (1919177)
PXRN01000023.1 (genomic DNA)
Halomarina oriensis
RRJ31050.1 (671145)
RRCH01000017.1 (genomic DNA)
Halobacteriales archaeon QS_5_70_17
PSP96386.1 (1919181)
PXRR01000072.1 (genomic DNA)
Halobacteriales archaeon SW_10_68_16
PSQ27880.1 (1919195)
PXSG01000115.1 (genomic DNA)
Halobacteriales archaeon SW_9_67_25
PSQ56332.1 (1919210)
PXSU01000167.1 (genomic DNA)
archaeon AArc Sl
AUX10454.1 (2055893)
CP025066.1 (chromosome)
halophilic archaeon DL31
AEN06289.1 (756883)
CP002988.1 (chromosome)
Halolamina sp CBA1230
OPF21938.1 (1853690)
MWUK01000003.1 (genomic DNA)
Halolamina sediminis
CVUA01000001.1_1726 (1480675)
CVUA01000001.1 ()
Halolamina pelagica
KPN31470.1 (699431)
LGUC01000001.1 (genomic DNA)
Halolamina rubra
BBJN01000035.1_6 (1380430)
BBJN01000035.1 ()
Haloplanus vescus
SDZ77702.1 (555874)
FNQT01000001.1 (genomic DNA)
Haloplanus aerogenes
RMB25577.1 (660522)
REFS01000001.1 (genomic DNA)
Haloplanus sp CBA1113
AXG07367.1 (1547899)
CP031150.1 (chromosome)

Haloplanus salinus
RCU48305.1 (1126245)
QPHM01000001.1 (genomic DNA)

Haloplanus natans DSM 17983
KE386573.1_2077 (926690)
KE386573.1 ()

Haloprofundus marisrubri
KTG11140.1 (1514971)
LOPU01000013.1 (genomic DNA)

Haloprofundus sp NK23
QQRR01000001.1_170 (2283527)
QQRR01000001.1 ()

Haloferacaceae archaeon SYSU A9 0
PEND01000006.1_204 (1985678)
PEND01000006.1 ()

Halogranum salarium B 1
EJN61180.1 (1210908)
ALJD01000002.1 (genomic DNA)

Halogranum gelatinilyticum
SDM79820.1 (660521)
FNHL01000003.1 (genomic DNA)

Halogranum amylolyticum
SEO83824.1 (660520)
FODV01000005.1 (genomic DNA)

Halorubraceae archaeon
PUNH01000329.1_16 (2093797)
PUNH01000329.1 ()

Halohasta litchfieldiae
ATW87274.1 (1073996)
CP024845.1 (chromosome)

Haloferacaceae archaeon B1 Br10_E2g22
LKML01000011.1_20 (1732021)
LKML01000011.1 ()

Halonotius sp J07HN4
ERH05873.1 (1070774)
KE356578.1 (genomic)

Halonotius pteroides
RJX47602.1 (268735)
QMDW01000040.1 (genomic DNA)

Halonotius sp F13 13
RJX42731.1 (2216978)
QKNY01000013.1 (genomic DNA)

Haloparvum sedimenti
LKIR01000089.1_13 (1678448)
LKIR01000089.1 ()

Halopenitus malekzadehii
SEH60035.1 (1267564)
FNWU01000011.1 (genomic DNA)

haloarchaeon 3A1_DGR
KK033115.1_10 (1071085)
KK033115.1 ()

Halopenitus persicus
AP017558.1_150 (1048396)
AP017558.1 ()

Halorubrum vacuolatum
SNR29954.1 (63740)
FZNQ01000002.1 (genomic DNA)

Halorubrum halodurans
OYR56422.1 (1383851)
NHPJ01000089.1 (genomic DNA)

Halobellus sp ZY21
RDQE01000009.1_20 (2480582)
RDQE01000009.1 ()

Halorubrum aethiopicum
LOAJ01000001.1_2769 (1758255)
LOAJ01000001.1 ()

Halorubrum sp 48 1 W
RAW45524.1 (2249761)
QMIM01000016.1 (genomic DNA)

Halorubrum sp BV1
JQKV01000001.1_482 (1498500)
JQKV01000001.1 ()

Halorubrum sp SD626R
CVRT01000006.1_3 (1419722)
CVRT01000006.1 ()

Halorubrum sodomense
SFR39373.1 (35743)
FOYN01000002.1 (genomic DNA)

Halorubrum sp T3
JH815329.1_415 (1194088)
JH815329.1 ()

Halorubrum trapanicum
AP017569.1_901 (29284)
AP017569.1 ()

Halorubrum sp Atlit 26R
RLM70401.1 (2282128)
QPLT01000006.1 (genomic DNA)

Halorubrum arcis JCM 13916
EMA71615.1 (1230455)
AOJJ01000049.1 (genomic DNA)

Halorubrum coriense DSM 10284

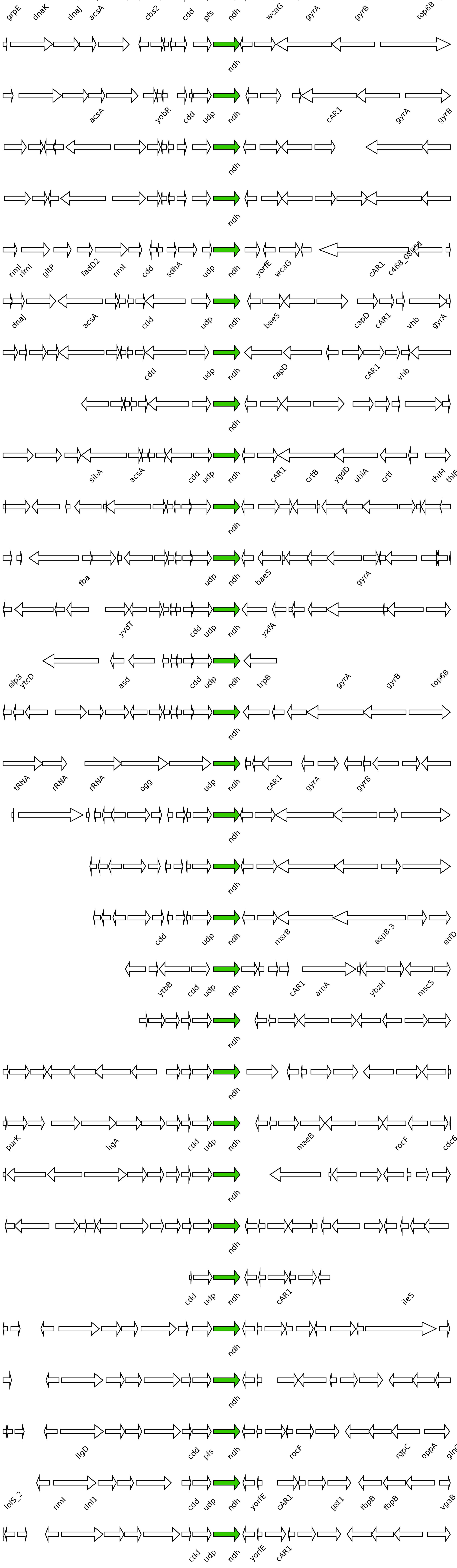

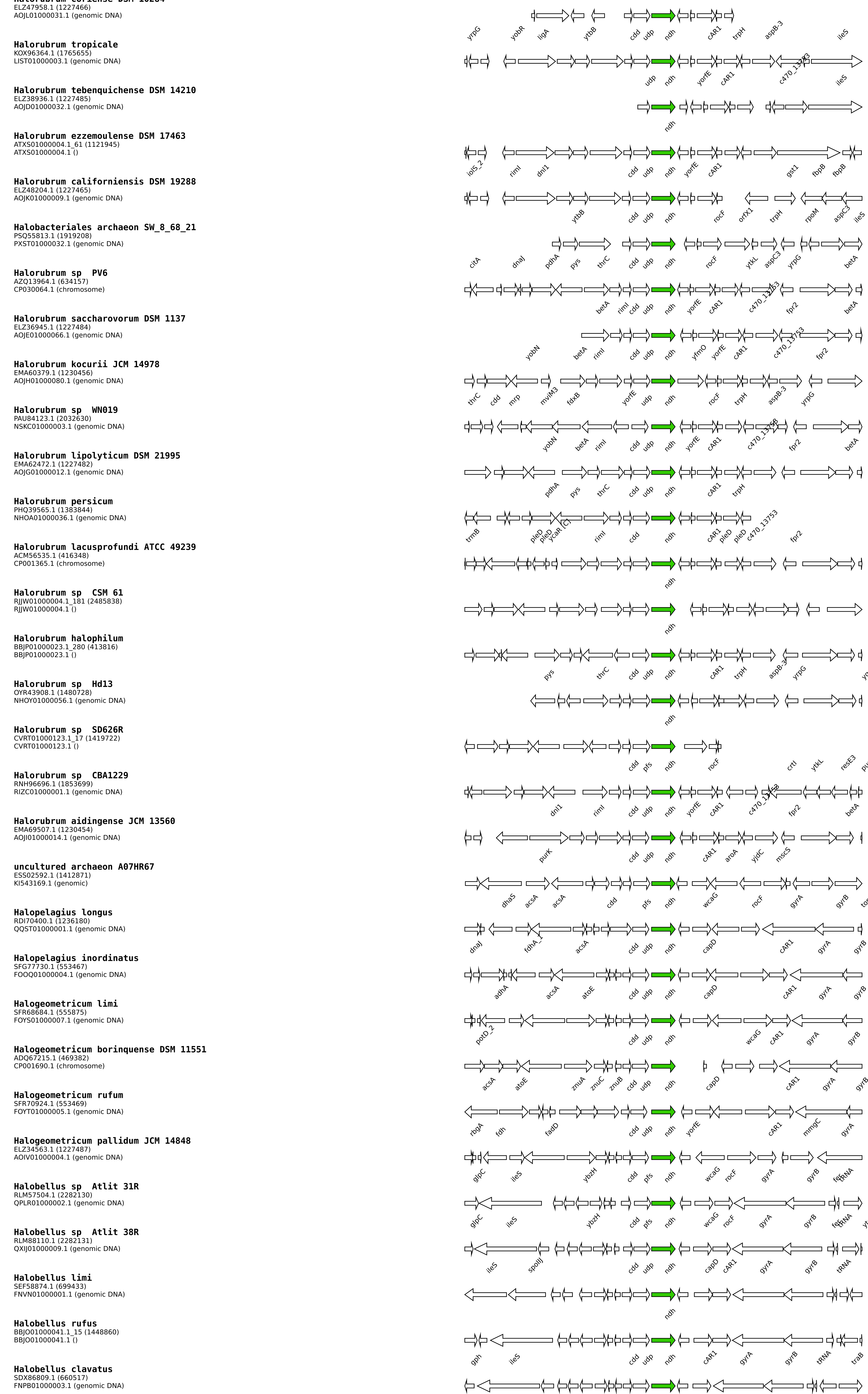

Halorubrum coriense DSM 10284
ELZ47958.1 (1227466)
AOJL01000031.1 (genomic DNA)
Halorubrum tropicale
KOX96364.1 (1765655)
LIST01000003.1 (genomic DNA)
Halorubrum tebenquichense DSM 14210
ELZ38936.1 (1227485)
AOJD01000032.1 (genomic DNA)
Halorubrum ezzemoulense DSM 17463
ATXS01000004.1_61 (1121945)
ATXS01000004.1 ()
Halorubrum californiensis DSM 19288
ELZ48204.1 (1227465)
AOJK01000009.1 (genomic DNA)
Halobacteriales archaeon SW_8_68_21
PSQ55813.1 (1919208)
PXST01000032.1 (genomic DNA)
Halorubrum sp PV6
AZQ13964.1 (634157)
CP030064.1 (chromosome)
Halorubrum saccharovorum DSM 1137
ELZ36945.1 (1227484)
AOJE01000066.1 (genomic DNA)
Halorubrum kocurii JCM 14978
EMA60379.1 (1230456)
AOJH01000080.1 (genomic DNA)
Halorubrum sp WN019
PAU84123.1 (2032630)
NSKC01000003.1 (genomic DNA)
Halorubrum lipolyticum DSM 21995
EMA62472.1 (1227482)
AOJG01000012.1 (genomic DNA)
Halorubrum persicum
PHQ39565.1 (1383844)
NHOA01000036.1 (genomic DNA)
Halorubrum lacusprofundi ATCC 49239
ACM56535.1 (416348)
CP001365.1 (chromosome)
Halorubrum sp CSM 61
RJJW01000004.1_181 (2485838)
RJJW01000004.1 ()
Halorubrum halophilum
BBJP01000023.1_280 (413816)
BBJP01000023.1 ()
Halorubrum sp Hd13
OYR43908.1 (1480728)
NHOY01000056.1 (genomic DNA)
Halorubrum sp SD626R
CVRT01000123.1_17 (1419722)
CVRT01000123.1 ()
Halorubrum sp CBA1229
RNH96696.1 (1853699)
RIZC01000001.1 (genomic DNA)
Halorubrum aidingense JCM 13560
EMA69507.1 (1230454)
AOJI01000014.1 (genomic DNA)
uncultured archaeon A07HR67
ESS02592.1 (1412871)
KI543169.1 (genomic)
Halopelagius longus
RDI70400.1 (1236180)
QQST01000001.1 (genomic DNA)
Halopelagius inordinatus
SFG77730.1 (553467)
FOOQ01000004.1 (genomic DNA)
Halogeometricum limi
SFR68684.1 (555875)
FOYS01000007.1 (genomic DNA)
Halogeometricum borinquense DSM 11551
ADQ67215.1 (469382)
CP001690.1 (chromosome)
Halogeometricum rufum
SFR70924.1 (553469)
FOYT01000005.1 (genomic DNA)
Halogeometricum pallidum JCM 14848
ELZ34563.1 (1227487)
AOIV01000004.1 (genomic DNA)
Halobellus sp Atlit 31R
RLM57504.1 (2282130)
QPLR01000002.1 (genomic DNA)
Halobellus sp Atlit 38R
RLM88110.1 (2282131)
QXIJ01000009.1 (genomic DNA)
Halobellus limi
SEF58874.1 (699433)
FNVN01000001.1 (genomic DNA)
Halobellus rufus
BBJO01000041.1_15 (1448860)
BBJO01000041.1 ()
Halobellus clavatus
SDX86809.1 (660517)
FNPB01000003.1 (genomic DNA)

Haloquadratum walsbyi J07HQW2
ERG94839.1 (1238425)
KE356561.1 (genomic)

Haloquadratum walsbyi J07HQW1
ERG92531.1 (1238424)
KE356560.1 (genomic)

Haloquadratum walsbyi C23
CCC40774.1 (768065)
FR746099.1 (genomic DNA)

Haloferax elongans ATCC BAA 1513
ELZ85132.1 (1230453)
AOLK01000017.1 (genomic DNA)

Haloferax larsenii JCM 13917
ELZ81393.1 (1227460)
AOLI01000012.1 (genomic DNA)

Haloferax sp SB29
KTG30864.1 (1544718)
LOPV01000017.1 (genomic DNA)

Haloferax mucosum ATCC BAA 1512
ELZ98844.1 (662479)
AOLN01000001.1 (genomic DNA)

Haloferax mediterranei ATCC 33500
AFK19343.1 (523841)
CP001868.2 (chromosome)

Haloferax gibbonsii
AKU07631.1 (35746)
CP011947.1 (chromosome)

Haloferax sp SB3
POG54991.1 (1544719)
LOPW02000017.1 (genomic DNA)

Haloferax sulfurifontis ATCC BAA 897
ELZ97673.1 (662480)
AOLM01000004.1 (genomic DNA)

Haloferax volcanii DS2
ADE03682.1 (309800)
CP001956.1 (chromosome)

Halobacteriales archaeon QS_6_71_20
PSQ08089.1 (1919183)
PXRU01000002.1 (genomic DNA)

Halobaculum gomorrense
SHH64275.1 (43928)
FQWV01000011.1 (genomic DNA)

Salinigranum rubrum
AUV81857.1 (755307)
CP026309.1 (chromosome)

Candidatus Halobonum tyrrellensis G22
ESP88730.1 (1324957)
ASGZ01000026.1 (genomic DNA)

halophilic archaeon J07HB67
ERH11577.1 (1085029)
KE356582.1 (genomic)

Haloquadratum sp J07HQX50
ERG97990.1 (1238426)
KE356563.1 (genomic)

Halorhabdus tiamatea SARL4B
ERJ06321.1 (1033806)
AFNT02000017.1 (genomic DNA)

Halorhabdus utahensis DSM 12940
ACV12933.1 (519442)
CP001687.1 (chromosome)

uncultured archaeon A07HB70
ESS06197.1 (1412872)
KI543181.1 (genomic)

halophilic archaeon J07HX64
ERH08301.1 (1085028)
KE356579.1 (genomic)

Haloferax gibbonsii
AKU09344.1 (35746)
CP011948.1 (plasmid)

Haladaptatus paucihalophilus DX253
EFW91163.1 (797209)
AEMG01000018.1 (genomic DNA)

Halococcus thailandensis JCM 13552
EMA56711.1 (1227457)
AOMF01000022.1 (genomic DNA)

Halococcus salifodinae DSM 8989
EMA48139.1 (1227456)
AOME01000102.1 (genomic DNA)

uncultured archaeon A07HN63
ESS09070.1 (1412873)
KI543214.1 (genomic)

halophilic archaeon
RBI59719.1 (29295)
QNGA01000008.1 (genomic DNA)

Haloarculaceae archaeon HArcel1
AWB27011.1 (1679096)
CP028858.1 (chromosome)

Halarchaeum acidiphilum MH1 52 1
BANO01000167.1_3 (1261545)
BANO01000167.1 ()

Candidatus Synechococcus spongiarum 142

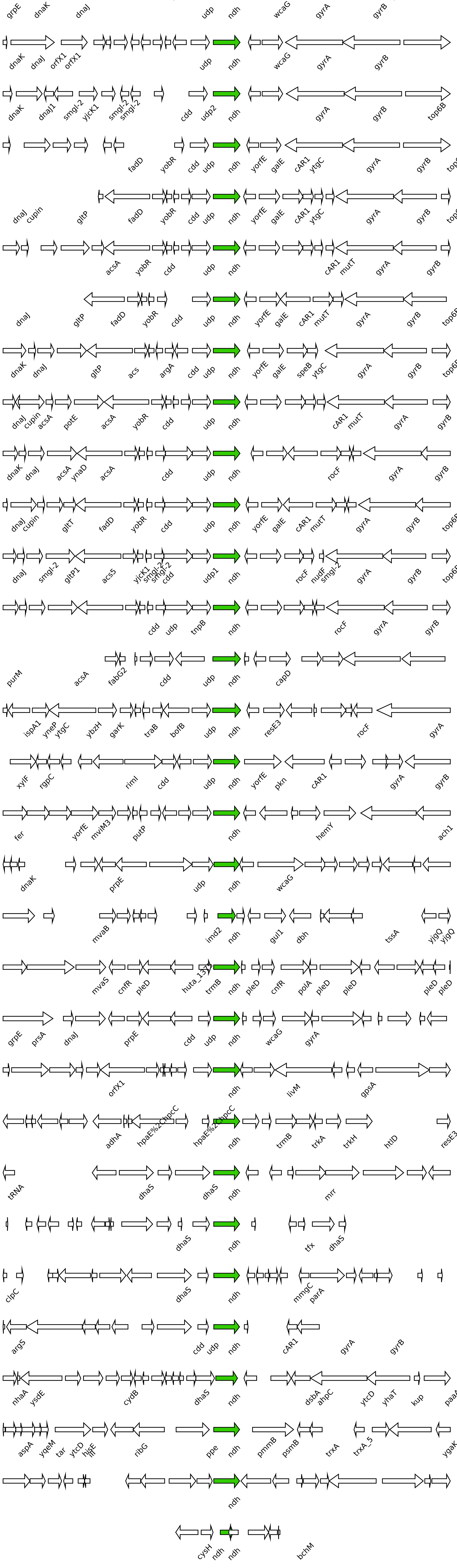

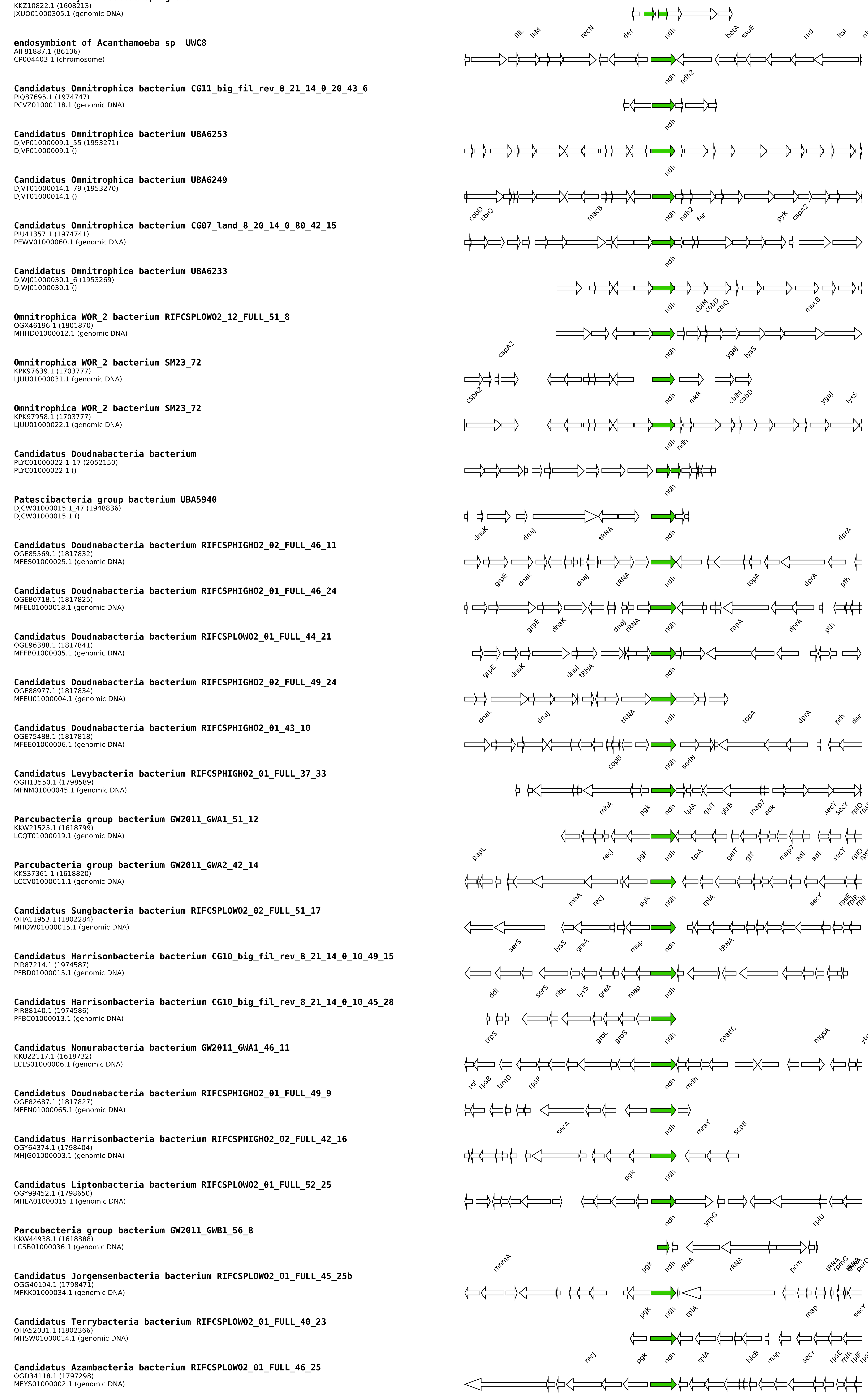
KKZ10822.1 (1608213)
JXUO01000305.1 (genomic DNA)
endosymbiont of Acanthamoeba sp UWC8
AIF81887.1 (86106)
CP004403.1 (chromosome)
Candidatus Omnitrophica bacterium CG11_big_fil_rev_8_21_14_0_20_43_6
PIQ87695.1 (1974747)
PCVZ01000118.1 (genomic DNA)
Candidatus Omnitrophica bacterium UBA6253
DJVP01000009.1_55 (1953271)
DJVP01000009.1 ()
Candidatus Omnitrophica bacterium UBA6249
DJVT01000014.1_79 (1953270)
DJVT01000014.1 ()
Candidatus Omnitrophica bacterium CG07_land_8_20_14_0_80_42_15
PIU41357.1 (1974741)
PEWV01000060.1 (genomic DNA)
Candidatus Omnitrophica bacterium UBA6233
DJWJ01000030.1_6 (1953269)
DJWJ01000030.1 ()
Omnitrophica WOR_2 bacterium RIFCSPLOWO2_12_FULL_51_8
OGX46196.1 (1801870)
MHHD01000012.1 (genomic DNA)
Omnitrophica WOR_2 bacterium SM23_72
KPK97639.1 (1703777)
LJUU01000031.1 (genomic DNA)
Omnitrophica WOR_2 bacterium SM23_72
KPK97958.1 (1703777)
LJUU01000022.1 (genomic DNA)
Candidatus Doudnabacteria bacterium
PLYC01000022.1_17 (2052150)
PLYC01000022.1 ()
Patescibacteria group bacterium UBA5940
DJCW01000015.1_47 (1948836)
DJCW01000015.1 ()
Candidatus Doudnabacteria bacterium RIFCSPHIGHO2_02_FULL_46_11
OGE85569.1 (1817832)
MFES01000025.1 (genomic DNA)
Candidatus Doudnabacteria bacterium RIFCSPHIGHO2_01_FULL_46_24
OGE80718.1 (1817825)
MFEL01000018.1 (genomic DNA)
Candidatus Doudnabacteria bacterium RIFCSPLOWO2_01_FULL_44_21
OGE96388.1 (1817841)
MFFB01000005.1 (genomic DNA)
Candidatus Doudnabacteria bacterium RIFCSPHIGHO2_02_FULL_49_24
OGE88977.1 (1817834)
MFEU01000004.1 (genomic DNA)
Candidatus Doudnabacteria bacterium RIFCSPHIGHO2_01_43_10
OGE75488.1 (1817818)
MFEE01000006.1 (genomic DNA)
Candidatus Levybacteria bacterium RIFCSPHIGHO2_01_FULL_37_33
OGH13550.1 (1798589)
MFNM01000045.1 (genomic DNA)
Parcubacteria group bacterium GW2011_GWA1_51_12
KKW21525.1 (1618799)
LCQT01000019.1 (genomic DNA)
Parcubacteria group bacterium GW2011_GWA2_42_14
KKS37361.1 (1618820)
LCCV01000011.1 (genomic DNA)
Candidatus Sungbacteria bacterium RIFCSPLOWO2_02_FULL_51_17
OHA11953.1 (1802284)
MHQW01000015.1 (genomic DNA)
Candidatus Harrisonbacteria bacterium CG10_big_fil_rev_8_21_14_0_10_49_15
PIR87214.1 (1974587)
PFBD01000015.1 (genomic DNA)
Candidatus Harrisonbacteria bacterium CG10_big_fil_rev_8_21_14_0_10_45_28
PIR88140.1 (1974586)
PFBC01000013.1 (genomic DNA)
Candidatus Nomurabacteria bacterium GW2011_GWA1_46_11
KKU22117.1 (1618732)
LCLS01000006.1 (genomic DNA)
Candidatus Doudnabacteria bacterium RIFCSPHIGHO2_01_FULL_49_9
OGE82687.1 (1817827)
MFEN01000065.1 (genomic DNA)
Candidatus Harrisonbacteria bacterium RIFCSPHIGHO2_02_FULL_42_16
OGY64374.1 (1798404)
MHJG01000003.1 (genomic DNA)
Candidatus Liptonbacteria bacterium RIFCSPLOWO2_01_FULL_52_25
OGY99452.1 (1798650)
MHLA01000015.1 (genomic DNA)
Parcubacteria group bacterium GW2011_GWB1_56_8
KKW44938.1 (1618888)
LCSB01000036.1 (genomic DNA)
Candidatus Jorgensenbacteria bacterium RIFCSPLOWO2_01_FULL_45_25b
OGG40104.1 (1798471)
MFKK01000034.1 (genomic DNA)
Candidatus Terrybacteria bacterium RIFCSPLOWO2_01_FULL_40_23
OHA52031.1 (1802366)
MHSW01000014.1 (genomic DNA)
Candidatus Azambacteria bacterium RIFCSPLOWO2_01_FULL_46_25
OGD34118.1 (1797298)
MEYS01000002.1 (genomic DNA)

Parcubacteria group bacterium M6 OD1 1
LSNK01000002.1_140 (1795559)
LSNK01000002.1 ()

Candidatus Yanofskybacteria bacterium
PLYL01000009.1_31 (2052154)
PLYL01000009.1 ()

Candidatus Yanofskybacteria bacterium RIFCSPHIGHO2_02_FULL_38_22b
OGN07316.1 (1802673)
MGJN01000007.1 (genomic DNA)

Candidatus Yanofskybacteria bacterium RIFCSPLOWO2_02_FULL_41_13
OGN30175.1 (1802702)
MGKQ01000007.1 (genomic DNA)

Candidatus Yanofskybacteria bacterium RIFCSPHIGHO2_01_FULL_43_32
OGN01778.1 (1802665)
MGJF01000002.1 (genomic DNA)

Candidatus Yanofskybacteria bacterium RIFCSPLOWO2_01_FULL_42_49
OGN22807.1 (1802694)
MGKI01000008.1 (genomic DNA)

Candidatus Yanofskybacteria bacterium RIFCSPHIGHO2_02_FULL_41_11
OGN08179.1 (1802675)
MGJP01000067.1 (genomic DNA)

Candidatus Yanofskybacteria bacterium RIFCSPHIGHO2_01_FULL_43_42
OGN03703.1 (1802666)
MGJG01000002.1 (genomic DNA)

Candidatus Peregrinibacteria bacterium CG11_big_fil_rev_8_21_14_0_20_46_8
PIQ77999.1 (1974793)
PCVQ01000017.1 (genomic DNA)

Candidatus Peregrinibacteria bacterium CG10_big_fil_rev_8_21_14_0_10_36_19
PIR54692.1 (1974784)
PFDP01000015.1 (genomic DNA)

Candidatus Peregrinibacteria bacterium GW2011_GWA2_44_7
KKT76680.1 (1619057)
LCJI01000015.1 (genomic DNA)

Candidatus Uhrbacteria bacterium GW2011_GWD2_52_7
KKW30413.1 (1618989)
LCRD01000013.1 (genomic DNA)

Candidatus Uhrbacteria bacterium RIFOXYC12_FULL_57_11
OGM00064.1 (1802423)
MGFF01000010.1 (genomic DNA)

Candidatus Uhrbacteria bacterium RIFCSPHIGHO2_01_FULL_63_20
OGL66739.1 (1802385)
MGDT01000006.1 (genomic DNA)

Parcubacteria group bacterium GW2011_GWA2_47_26
KKU73941.1 (1618844)
LCOG01000003.1 (genomic DNA)

Candidatus Uhrbacteria bacterium RIFCSPHIGHO2_02_FULL_57_19
OGL73977.1 (1802391)
MGDZ01000012.1 (genomic DNA)

Candidatus Uhrbacteria bacterium RIFCSPHIGHO2_02_FULL_60_44
OGL73238.1 (1802393)
MGEB01000046.1 (genomic DNA)

Chlorobi bacterium OLB7
KXK57604.1 (1619898)
LMYZ01000041.1 (genomic DNA)

Ignavibacteria bacterium UBA5590
DILO01000036.1_6 (1948678)
DILO01000036.1 ()

Chlorobi bacterium OLB5
KXK56779.1 (1617412)
LLZO01000003.1 (genomic DNA)

Ignavibacteria bacterium GWC2_56_12
OGU46195.1 (1798429)
MHAB01000052.1 (genomic DNA)

Ignavibacteria bacterium UBA6688
DKJQ01000392.1_7 (1948685)
DKJQ01000392.1 ()

Candidatus Thermochlorobacteriaceae bacterium GBChlB
KER10097.1 (1519464)
JPGV01000020.1 (genomic DNA)

Candidatus Thermochlorobacter aerophilum
RFM23800.1 (1868324)
PHFL01000058.1 (genomic DNA)

Candidatus Hydrogenedentes bacterium
PCJ64426.1 (2030809)
NVWG01000017.1 (genomic DNA)

Candidatus Uhrbacteria bacterium CG_4_9_14_3_um_filter_41_35
PJA46681.1 (1975034)
PFWT01000009.1 (genomic DNA)

Rubrobacter xylanophilus DSM 9941
ABG04065.1 (266117)
CP000386.1 (chromosome)

Rubrobacter radiotolerans
AHY46368.1 (42256)
CP007514.1 (chromosome)

Rubrobacter indicoceani
CP031115.1_1109 (2051957)
CP031115.1 ()

Rubrobacter aplysinae
LEKH01000021.1_7 (909625)
LEKH01000021.1 ()

Rubrobacter xylanophilus DSM 9941

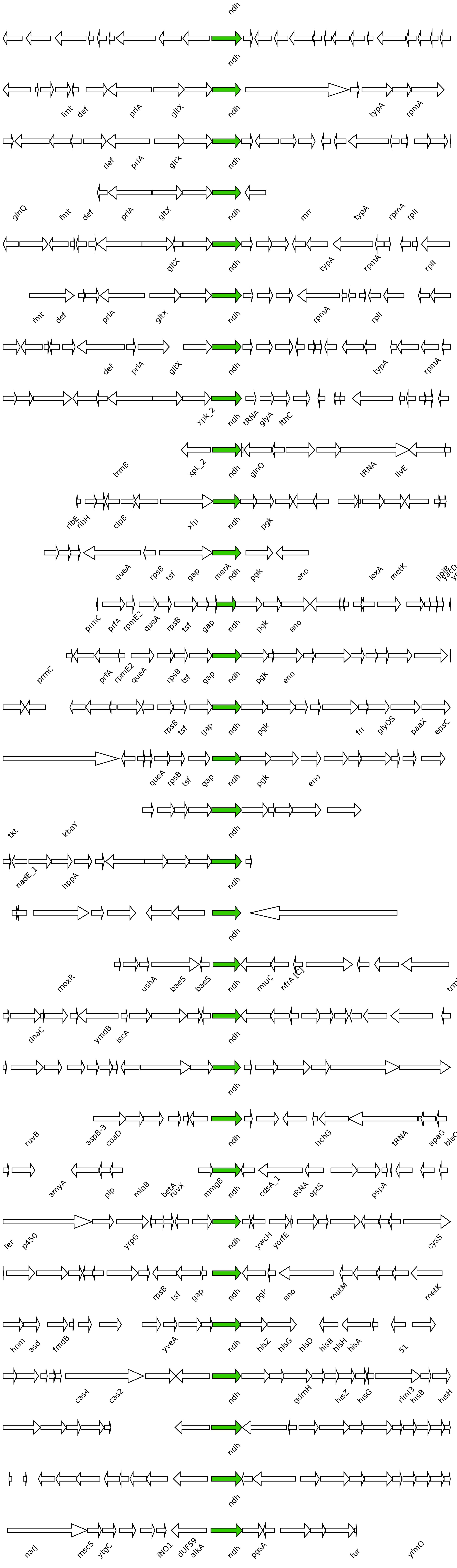

ABG04183.1 (266117)
CP000386.1 (chromosome)

Rubrobacter radiotolerans
AHY46464.1 (42256)
CP007514.1 (chromosome)

Rubrobacter indicoceani
CP031115.1_1205 (2051957)
CP031115.1 ()

Rubrobacter aplysinae
LEKH01000008.1_107 (909625)
LEKH01000008.1 ()

Leptospirillum sp Group IV UBA BS
EQD23939.1 (1260983)
KI543143.1 (genomic)

Leptospirillum ferriphilum ML 04
AFS54301.1 (1048260)
CP002919.1 (chromosome)

Leptospirillum ferrooxidans C2 3
BAM07590.1 (1162668)
AP012342.1 (genomic DNA)

Sulfobacillus acidophilus DSM 10332
AEW05430.1 (679936)
CP003179.1 (chromosome)

Sulfobacillus benefaciens
PSR33449.1 (453960)
PXYW01000020.1 (genomic DNA)

Sulfobacillus thermosulfidooxidans DSM 9293
SMC07278.1 (929705)
FWWY01000001.1 (genomic DNA)

Sulfobacillus sp hq2
POB09126.1 (2039167)
NWUL01000048.1 (genomic DNA)

Rubrobacter radiotolerans
AHY46386.1 (42256)
CP007514.1 (chromosome)

Rubrobacter indicoceani
CP031115.1_1126 (2051957)
CP031115.1 ()

Rubrobacter xylanophilus DSM 9941
ABG04078.1 (266117)
CP000386.1 (chromosome)

Rubrobacter aplysinae
LEKH01000020.1_15 (909625)
LEKH01000020.1 ()

Candidatus Daviesbacteria bacterium GW2011_GWA1_38_7
KKQ80378.1 (1618418)
LBVE01000013.1 (genomic DNA)

Candidatus Daviesbacteria bacterium RIFCSPHIGHO2_12_FULL_47_45
OGE40671.1 (1797781)
MFDI01000009.1 (genomic DNA)

Candidatus Blackburnbacteria bacterium RIFCSPHIGHO2_02_FULL_39_13
OGY09867.1 (1797515)
MHBY01000004.1 (genomic DNA)

Parcubacteria group bacterium CG2_30_48_51
OIP79346.1 (1805320)
MNZI01000036.1 (genomic DNA)

Candidatus Niyogibacteria bacterium CG10_big_fil_rev_8_21_14_0_10_46_36
PIR69513.1 (1974726)
PFCO01000006.1 (genomic DNA)

Candidatus Niyogibacteria bacterium RIFCSPLOWO2_02_FULL_45_13
OGZ31605.1 (1801725)
MHMR01000004.1 (genomic DNA)

Candidatus Ryanbacteria bacterium RIFCSPHIGHO2_02_FULL_48_12
OGZ50057.1 (1802121)
MHNR01000009.1 (genomic DNA)

Candidatus Giovannonibacteria bacterium RIFCSPHIGHO2_01_FULL_45_23
OGF62107.1 (1798325)
MFHD01000023.1 (genomic DNA)

Candidatus Giovannonibacteria bacterium RIFCSPHIGHO2_01_FULL_44_100
OGF60599.1 (1798324)
MFHC01000022.1 (genomic DNA)

Candidatus Giovannonibacteria bacterium GW2011_GWA1_44_29
KKT63688.1 (1618646)
LCIV01000005.1 (genomic DNA)

Candidatus Giovannonibacteria bacterium RIFCSPHIGHO2_01_FULL_45_33
OGF61186.1 (1798327)
MFHF01000038.1 (genomic DNA)

Candidatus Giovannonibacteria bacterium RIFCSPLOWO2_01_FULL_44_16
OGF83072.1 (1798348)
MFIA01000008.1 (genomic DNA)

Candidatus Giovannonibacteria bacterium RIFCSPLOWO2_12_FULL_44_32
OGF96913.1 (1798366)
MFIS01000025.1 (genomic DNA)

Candidatus Giovannonibacteria bacterium GW2011_GWB1_47_6b
KKU75969.1 (1618655)
LCOK01000038.1 (genomic DNA)

Candidatus Wildermuthbacteria bacterium RIFCSPHIGHO2_02_FULL_47_17
OHA68233.1 (1802452)
MHTX01000020.1 (genomic DNA)

Candidatus Giovannonibacteria bacterium RIFCSPLOWO2_12_FULL_44_15
OGF93717.1 (1798364)
MFIQ01000007.1 (genomic DNA)

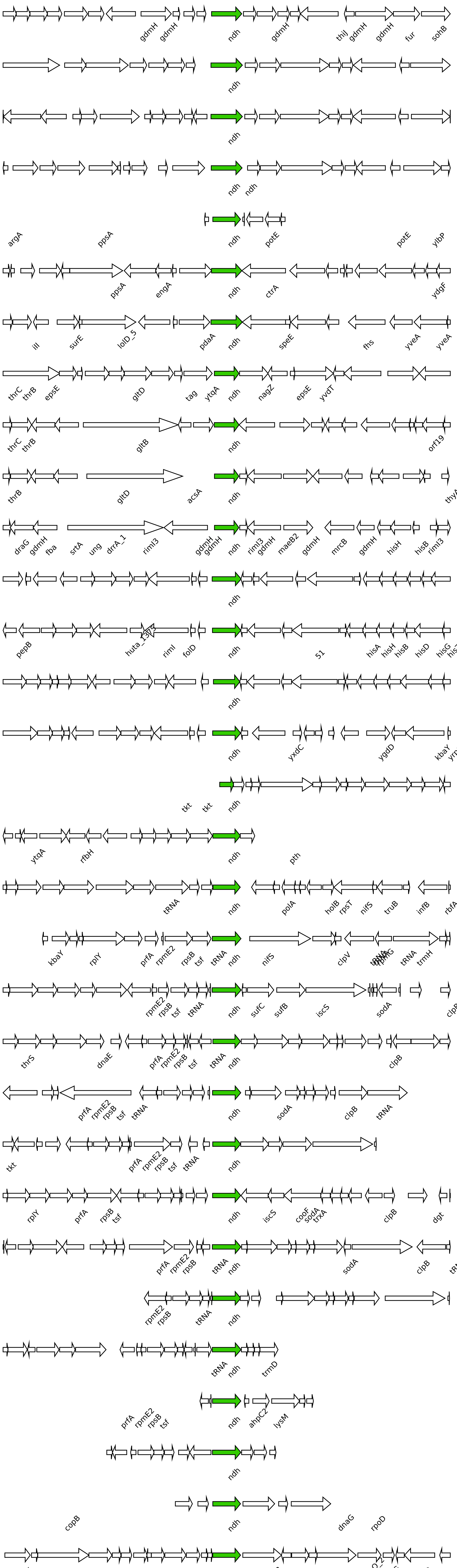

Candidatus Giovannonibacteria bacterium RIFCSPHIGHO2_12_FULL_43_15
OGF77345.1 (1798341)
MFHT01000021.1 (genomic DNA)

Candidatus Giovannonibacteria bacterium
HBT81526.1 (2053557)
DOMN01000012.1 (genomic DNA)

Candidatus Doudnabacteria bacterium RIFCSPHIGHO2_01_FULL_49_9
OGE82943.1 (1817827)
MFEN01000058.1 (genomic DNA)

Candidatus Nomurabacteria bacterium RIFCSPHIGHO2_02_FULL_36_29
OGI75820.1 (1801749)
MFTZ01000006.1 (genomic DNA)

Parcubacteria group bacterium RIFCSPLOWO2_01_FULL_48_18
OHB23403.1 (1817743)
MHXH01000001.1 (genomic DNA)

Candidatus Harrisonbacteria bacterium CG10_big_fil_rev_8_21_14_0_10_40_38
PIR89137.1 (1974583)
PFAZ01000007.1 (genomic DNA)

Candidatus Curtissbacteria bacterium RIFCSPLOWO2_12_FULL_41_16
OGE03457.1 (1797736)
MFBZ01000006.1 (genomic DNA)

Candidatus Peribacteria bacterium RIFCSPHIGHO2_01_FULL_51_9
OGJ59132.1 (1801915)
MFXQ01000022.1 (genomic DNA)

Candidatus Lloydbacteria bacterium RIFCSPHIGHO2_02_FULL_50_11
OGZ08710.1 (1798660)
MHLK01000001.1 (genomic DNA)

Candidatus Lloydbacteria bacterium RIFCSPLOWO2_01_FULL_54_18
OGZ12906.1 (1798666)
MHLQ01000044.1 (genomic DNA)

Parcubacteria group bacterium GW2011_GWC2_44_17
KKT46051.1 (1618929)
LCIC01000032.1 (genomic DNA)

Candidatus Saccharibacteria bacterium UBA4663
DHLR01000014.1_25 (1953343)
DHLR01000014.1 ()

bacterium UBA6025
DIZP01000043.1_30 (1947813)
DIZP01000043.1 ()

Patescibacteria group bacterium UBA6028
DIZM01000022.1_16 (1948839)
DIZM01000022.1 ()

Candidatus Saccharibacteria bacterium YM_S32_TM7_50_20
CP025011.1_1086 (2056494)
CP025011.1 ()

Candidatus Saccharibacteria bacterium UBA4723
DHJJ01000040.1_7 (1953346)
DHJJ01000040.1 ()

Candidatus Saccharibacteria bacterium UBA5148
DHXU01000003.1_212 (1953351)
DHXU01000003.1 ()

Candidatus Saccharibacteria bacterium UBA4729
DHJD01000020.1_1 (1953349)
DHJD01000020.1 ()

Candidatus Saccharibacteria bacterium CG_4_10_14_0_2_um_filter_52_9
PIZ62949.1 (1974866)
PFOA01000035.1 (genomic DNA)

Candidatus Saccharibacteria bacterium UBA5150
DHXS01000016.1_36 (1953352)
DHXS01000016.1 ()

Candidatus Saccharibacteria bacterium UBA6224
DJWS01000009.1_204 (1953366)
DJWS01000009.1 ()

Candidatus Saccharibacteria bacterium UBA5946
DJCQ01000003.1_82 (1953358)
DJCQ01000003.1 ()

Candidatus Saccharibacteria bacterium RAAC3_TM7_1
AHB42640.1 (1394711)
CP006915.1 (chromosome)

Candidatus Saccharibacteria bacterium UBA6579
DJJB01000006.1_16 (1953373)
DJJB01000006.1 ()

Candidatus Saccharibacteria bacterium UBA6575
DJJF01000044.1_12 (1953372)
DJJF01000044.1 ()

Candidatus Saccharibacteria bacterium UBA1020
DBAC01000005.1_9 (1953310)
DBAC01000005.1 ()

Candidatus Saccharibacteria bacterium UBA1050
DCDR01000001.1_188 (1953313)
DCDR01000001.1 ()

Candidatus Saccharibacteria bacterium GW2011_GWC2_48_9
KKW03140.1 (1619071)
LCPR01000008.1 (genomic DNA)

Candidatus Saccharibacteria bacterium UBA6039
DIZB01000008.1_25 (1953361)
DIZB01000008.1 ()

Candidatus Saccharibacteria bacterium 49 20
OJU87773.1 (1895829)
MKSN01000001.1 (genomic DNA)

Candidatus Saccharibacteria bacterium UBA6040

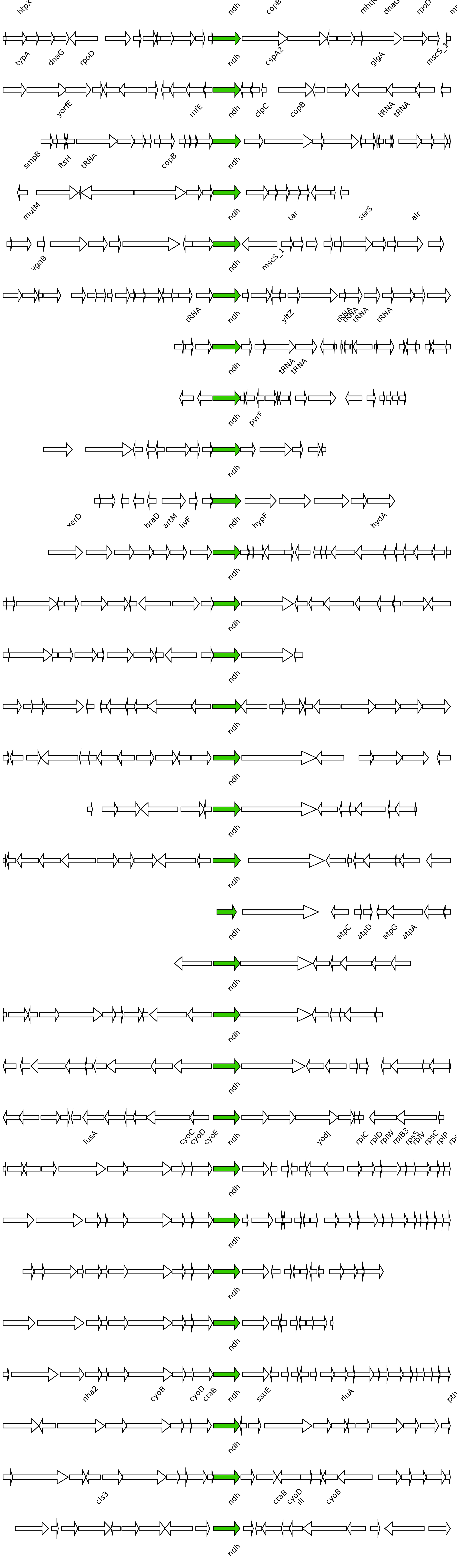

DIZA01000007.1_27 (1953362)
DIZA01000007.1 ()

Candidatus Saccharibacteria bacterium RIFCSPHIGHO2_12_FULL_44_22
OGL31660.1 (1802140)
MGCS01000013.1 (genomic DNA)

Candidatus Saccharibacteria bacterium UBA4724
DHJI01000054.1_17 (1953347)
DHJI01000054.1 ()

Candidatus Saccharibacteria bacterium UBA2112
DCYK01000029.1_46 (1953324)
DCYK01000029.1 ()

candidate division TM7 bacterium JGI 0001002 L20
AUNW02000009.1_10 (1094954)
AUNW02000009.1 ()

Candidatus Saccharimonas aalborgensis
AGL61812.1 (1332188)
CP005957.1 (chromosome)

Candidatus Saccharibacteria bacterium UBA6258
DJVK01000007.1_50 (1953367)
DJVK01000007.1 ()

Candidatus Saccharibacteria bacterium 32 50 10
OYX39565.1 (1970480)
NCEC01000003.1 (genomic DNA)

Candidatus Saccharibacteria bacterium UBA1944
DDEW01000019.1_24 (1953321)
DDEW01000019.1 ()

Candidatus Saccharibacteria bacterium GW2011_GWC2_48_9
KKW05173.1 (1619071)
LCPR01000001.1 (genomic DNA)

Candidatus Saccharibacteria bacterium 32 49 12
OYX42031.1 (1970479)
NCEI01000029.1 (genomic DNA)

Candidatus Saccharibacteria bacterium 32 50 13
OYX53205.1 (1970481)
NCEK01000022.1 (genomic DNA)

Candidatus Saccharibacteria bacterium
MAU33901.1 (2026720)
PABZ01000011.1 (genomic DNA)

Candidatus Saccharibacteria bacterium UBA4215
DFTJ01000017.1_84 (1953341)
DFTJ01000017.1 ()

Candidatus Saccharibacteria bacterium UBA6175
DITV01000002.1_227 (1953363)
DITV01000002.1 ()

Candidatus Saccharibacteria bacterium 32 49 10
OYW85750.1 (1970478)
NCDL01000094.1 (genomic DNA)

Candidatus Saccharibacteria bacterium 49 20
OJU87709.1 (1895829)
MKSN01000001.1 (genomic DNA)

Candidatus Saccharibacteria bacterium GW2011_GWC2_44_17
AKM80020.1 (1619070)
CP011211.1 (chromosome)

Candidatus Saccharibacteria bacterium RIFCSPHIGHO2_12_FULL_44_22
OGL32910.1 (1802140)
MGCS01000001.1 (genomic DNA)

Glaciecola sp KUL10
GBL04092.1 (2161813)
BGNG01000003.1 (genomic DNA)

Calothrix sp NIES 3974
BAZ05167.1 (2005462)
AP018254.1 (genomic DNA)

Candidatus Scalindua sp husup a2
GAX62525.1 (1284222)
BAOS01000034.1 (genomic DNA)

Candidatus Scalindua brodae
KHE90712.1 (237368)
JRYO01000244.1 (genomic DNA)

marine gamma proteobacterium HTCC2080
EAW40333.1 (247639)
AAVV01000009.1 (genomic DNA)

Congregibacter litoralis KT71
EAQ98248.1 (314285)
CM002299.1 (chromosome)

Halieaceae bacterium UBA6060
DIYG01000172.1_9 (1951921)
DIYG01000172.1 ()

Pseudohaliea rubra DSM 19751
KGE05119.1 (1265313)
KN234748.1 (genomic)

Luminiphilus syltensis NOR5 1B
EED34367.1 (565045)
DS999411.1 (genomic)

Chromatocurvus halotolerans
QQSW01000020.1_6 (1132028)
QQSW01000020.1 ()

gamma proteobacterium HdN1
CBL43828.1 (83406)
FP929140.1 (genomic DNA)

Ketobacter alkanivorans
AUM11992.1 (1917421)
CP022684.1 (chromosome)

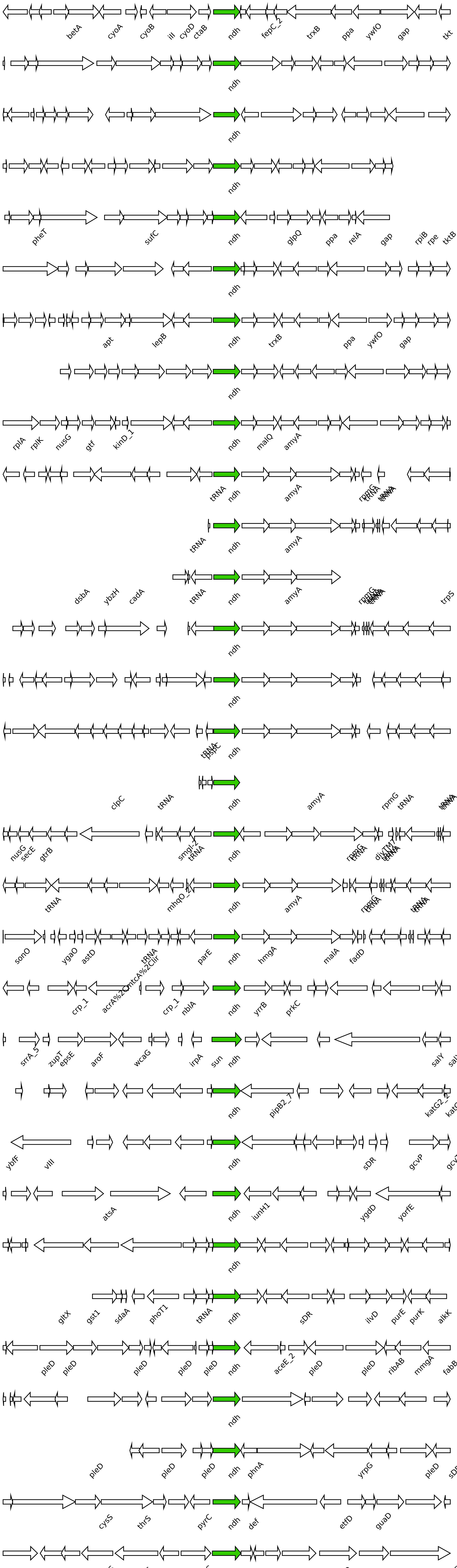

Ketobacter sp
RLP52420.1 (2083498)
RAHY01000034.1 (genomic DNA)

Candidatus Saccharibacteria bacterium 32 49 12
OYX42240.1 (1970479)
NCEI01000025.1 (genomic DNA)

Coriobacteriia bacterium
PKWR01000019.1_7 (2052159)
PKWR01000019.1 ()

Actinobacteria bacterium UBA3086
DFBG01000142.1_6 (1953123)
DFBG01000142.1 ()

Coriobacteriaceae bacterium EMTCatB1
GAV31759.1 (1927122)
BDLO01000001.1 (genomic DNA)

Actinobacteria bacterium HGW Actinobacteria 1
PKQ37924.1 (2013644)
PHFB01000003.1 (genomic DNA)

Actinobacteria bacterium UBA2279
DDWT01000030.1_4 (1953119)
DDWT01000030.1 ()

Actinobacteria bacterium HGW Actinobacteria 7
PKQ16269.1 (2013652)
PHET01000008.1 (genomic DNA)

Brockia lithotrophica
PTQ52577.1 (933949)
PEBW01000002.1 (genomic DNA)

Thermoplasmatales archaeon SW_10_69_26
PSG98161.1 (1919232)
PXUB01000004.1 (genomic DNA)

Bradymonas sediminis
AWV88360.1 (1548548)
CP030032.1 (chromosome)

Bradymonadaceae bacterium TMQ3
RDV37015.1 (2283320)
QRGZ01000007.1 (genomic DNA)

Nitrospiraceae bacterium UBA6905
DKBH01000040.1_16 (1952984)
DKBH01000040.1 ()

Candidatus Sulfobium mesophilum
SPP99654.1 (2016548)
OUUY01000007.1 (genomic DNA)

Deltaproteobacteria bacterium RBG_16_49_23
OGP78021.1 (1797840)
MGQQ01000045.1 (genomic DNA)

Candidatus Uhrbacteria bacterium CG22_combo_CG10 13_8_21_14_all_47_17
PIP60150.1 (1975041)
PCSZ01000079.1 (genomic DNA)

Oceanicoccus sagamiensis
ARN75059.1 (716816)
CP019343.1 (chromosome)

Parcubacteria group bacterium GW2011_GWA2_38_13b
OWK26862.1 (1618780)
LBUF02000017.1 (genomic DNA)

Ignatzschineria larvae DSM 13226
AZOD01000015.1_40 (1111732)
AZOD01000015.1 ()

Commensalibacter intestini A911
EHD13452.1 (1088868)
AGFR01000009.1 (genomic DNA)

Commensalibacter sp AMU001
AYN86493.1 (2478912)
CP033087.1 (chromosome)

Neoasaia chiangmaiensis
AQS87401.1 (320497)
CP014691.1 (chromosome)

Asaia bogorensis NBRC 16594
BAT20218.1 (1231624)
AP014690.1 (genomic DNA)

Saccharibacter floricola DSM 15669
KB899343.1_34 (1123227)
KB899343.1 ()

Parasaccharibacter apium
CP020554.1_1043 (1510841)
CP020554.1 ()

Neokomagataea thailandica NBRC 106555
BCZB01000011.1_1 (1223520)
BCZB01000011.1 ()

Gluconobacter japonicus
KXV21250.1 (376620)
LHZH01000121.1 (genomic DNA)

Gluconobacter oxydans 621H
AAW61415.1 (290633)
CP000009.1 (chromosome)

Gluconobacter morbifer G707
EHH69059.1 (1088869)
AGQV01000001.1 (genomic DNA)

Tanticharoenia sakaeratensis NBRC 103193
GAN53073.1 (1231623)
BALE01000004.1 (genomic DNA)

Sphingomonas sp MM 1

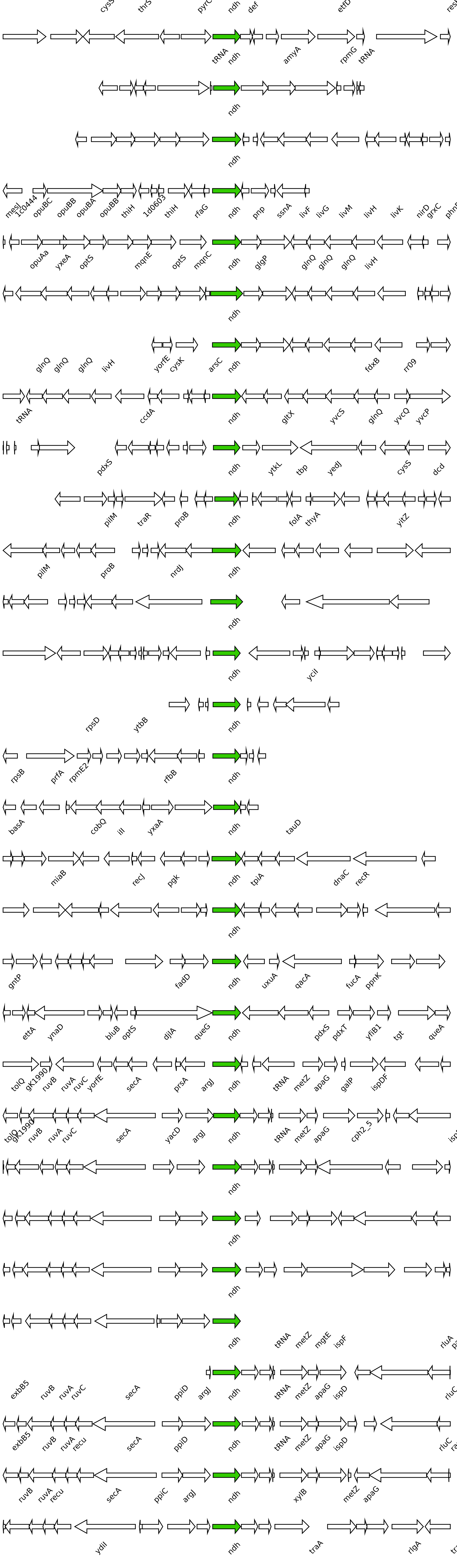

AGH52028.1 (745310)
CP004040.1 (plasmid)

Sphingobium baderi
ALR22968.1 (1332080)
CP013265.1 (plasmid)

Robbsia andropogonis
KKB63276.1 (28092)
LAQU01000011.1 (genomic DNA)

Azospirillum humicireducens
ANC90550.1 (1226968)
CP015285.1 (chromosome)

Confluentimicrobium sp EMB200 NS6
ALG91964.1 (1609966)
CP010869.1 (chromosome)

Burkholderia sp JS23
SDV50391.1 (1770053)
FNLO01000011.1 (genomic DNA)

Pandoraea apista
AJE98332.1 (93218)
CP010518.4 (chromosome)

Amantichitinum ursilacus
KPC50729.1 (857265)
LAQT01000027.1 (genomic DNA)

Comamonas sp BIGb0124
ROR20228.1 (2485130)
RJVF01000012.1 (genomic DNA)

Nitrosovibrio sp Nv17
SFW21195.1 (1855339)
FPIQ01000006.1 (genomic DNA)

Altererythrobacter sp HN Y73
RKF17668.1 (2320269)
RAPF01000013.1 (genomic DNA)

Nitrosospira sp 56 18
OJY12651.1 (1895901)
MKVR01000017.1 (genomic DNA)

Novosphingobium tardaugens NBRC 16725
GAD50390.1 (1219035)
BASZ01000008.1 (genomic DNA)

Arboricoccus pini
SNB71040.1 (1963835)
FYEH01000008.1 (genomic DNA)

Bordetella sp 3d 2 2
PWF23994.1 (2175229)
QETA01000002.1 (genomic DNA)

Luteimonas sp JM171
AOH35794.1 (1896164)
CP017074.1 (chromosome)

Xanthomonadaceae bacterium NML91 0213
PJJ97620.1 (2032582)
NWQL01000004.1 (genomic DNA)

Acidocella aminolytica 101 DSM 11237
GAN80347.1 (1120923)
BANC01000044.1 (genomic DNA)

Comamonas terrigena
DDHF01000082.1_138 (32013)
DDHF01000082.1 ()

Comamonas testosteroni
BBJZ01000015.1_61 (285)
BBJZ01000015.1 ()

Roseomonas aestuarii
PWC29407.1 (568898)
PDOA01000003.1 (genomic DNA)

Roseomonas gilardii
APT59249.1 (257708)
CP015583.1 (chromosome)

Acetobacter senegalensis
CEF42045.1 (446692)
LN606600.1 (genomic DNA)

Acetobacter pasteurianus IFO 3283 01
BAH98749.1 (634452)
AP011121.1 (genomic DNA)

Acetobacter syzygii 9H 2
GAN70884.1 (1231343)
BAMZ01000009.1 (genomic DNA)

Neoasaia chiangmaiensis
AQS87644.1 (320497)
CP014691.1 (chromosome)

Komagataeibacter xylinus
ATU71812.1 (28448)
CP024644.1 (chromosome)

Komagataeibacter hansenii
LUCI01000014.1_29 (436)
LUCI01000014.1 ()

Komagataeibacter hansenii
LUCI01000016.1_41 (436)
LUCI01000016.1 ()

Komagataeibacter xylinus
ATU73135.1 (28448)
CP024644.1 (chromosome)

Acidomonas methanolica NBRC 104435
GAJ29927.1 (1231351)
BAND01000085.1 (genomic DNA)

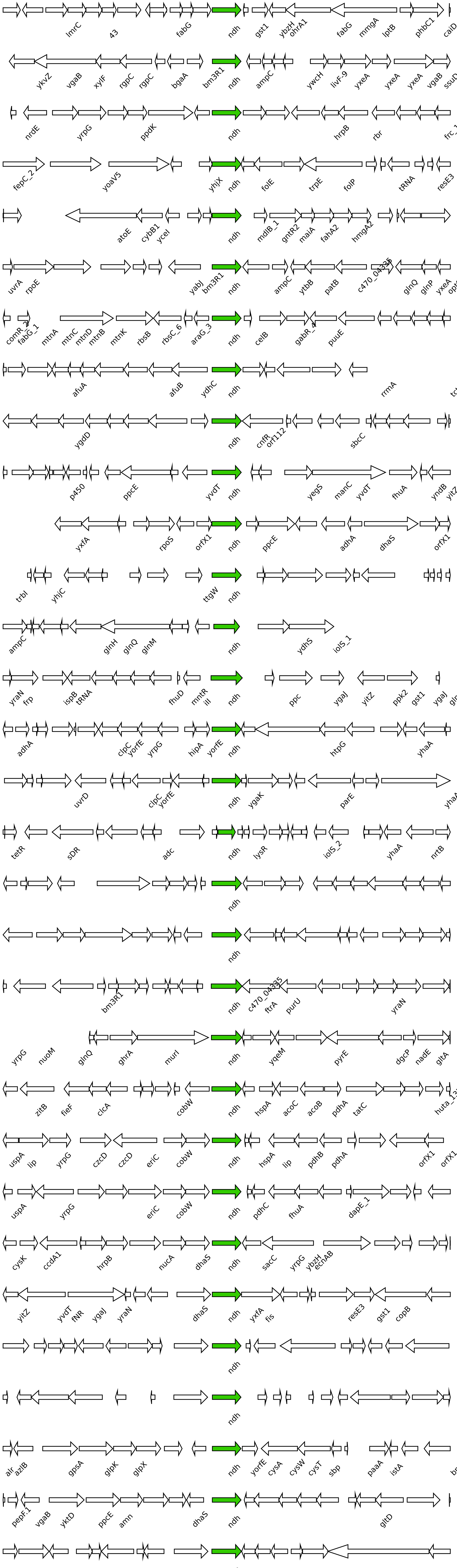

Acetobacter nitrogenifigens DSM 23921   LMG 23498
AUBI01000016.1_11 (1120919)
AUBI01000016.1 ()

Gluconacetobacter diazotrophicus PA1 5
ACI52123.1 (272568)
CP001189.1 (chromosome)

Acetobacter aceti
AQS85454.1 (435)
CP014692.1 (chromosome)

Acetobacter sp DsW_059
OUJ11384.1 (1670661)
JOPI01000011.1 (genomic DNA)

Acetobacter papayae JCM 25143
BAIN01000003.1_104 (1234671)
BAIN01000003.1 ()

Gluconobacter japonicus
KXV19976.1 (376620)
LHZH01000134.1 (genomic DNA)

Gluconobacter morbifer G707
EHH67517.1 (1088869)
AGQV01000010.1 (genomic DNA)

Acetobacter syzygii 9H 2
GAN71987.1 (1231343)
BAMZ01000027.1 (genomic DNA)

Acetobacter pasteurianus IFO 3283 01
BAI00175.1 (634452)
AP011121.1 (genomic DNA)

Acetobacter senegalensis
CEF41475.1 (446692)
LN606600.1 (genomic DNA)

Acetobacter persici
AQT05361.1 (1076596)
CP014687.1 (chromosome)

Neokomagataea thailandica NBRC 106555
BCZB01000004.1_9 (1223520)
BCZB01000004.1 ()

Parvularcula oceani
JPHU01000001.1_178 (1247963)
JPHU01000001.1 ()

Sphingomonas aeria Xue et al 2018 non Park et al 2015
PGFU01000019.1_22 (2052949)
PGFU01000019.1 ()

Halofilum ochraceum
LVEG02000004.1_81 (1611323)
LVEG02000004.1 ()

Frateuria aurantia DSM 6220
AFC87347.1 (767434)
CP003350.1 (chromosome)

Burkholderiales bacterium
PLEY01000036.1_11 (1891238)
PLEY01000036.1 ()

Methyloversatilis universalis FAM5
EGK70712.1 (1000565)
AFHG01000053.1 (genomic DNA)

Methyloversatilis sp RAC08
AOF83000.1 (1842540)
CP016448.1 (chromosome)

Burkholderiales bacterium
PLEY01000036.1_12 (1891238)
PLEY01000036.1 ()

Burkholderiales bacterium RIFCSPLOWO2_02_FULL_57_36
OGB22914.1 (1797562)
MESE01000098.1 (genomic DNA)

Noviherbaspirillum humi
SNS63613.1 (1688639)
FZOT01000004.1 (genomic DNA)

Cupriavidus gilardii CR3
ALD91182.1 (1267562)
CP010516.1 (chromosome)

Ralstonia pickettii 12J
ACD27568.1 (402626)
CP001068.1 (chromosome)

Ralstonia sp PBA
EIZ04491.1 (795666)
AKCV01000019.1 (genomic DNA)

Pandoraea thiooxydans
AKJ70507.1 (445709)
CP011568.3 (chromosome)

Pandoraea apista
AJF01311.1 (93218)
CP010518.4 (chromosome)

Burkholderia mallei
AIP74784.1 (13373)
CP009147.1 (chromosome)

Mycoavidus cysteinexigens
BBE09133.1 (1553431)
AP018150.1 (genomic DNA)

Candidatus Glomeribacter gigasporarum BEG34
CCD29034.1 (1070319)
CAFB01000037.1 (genomic DNA)

Candidatus Glomeribacter gigasporarum
LXBI01000088.1_5 (132144)

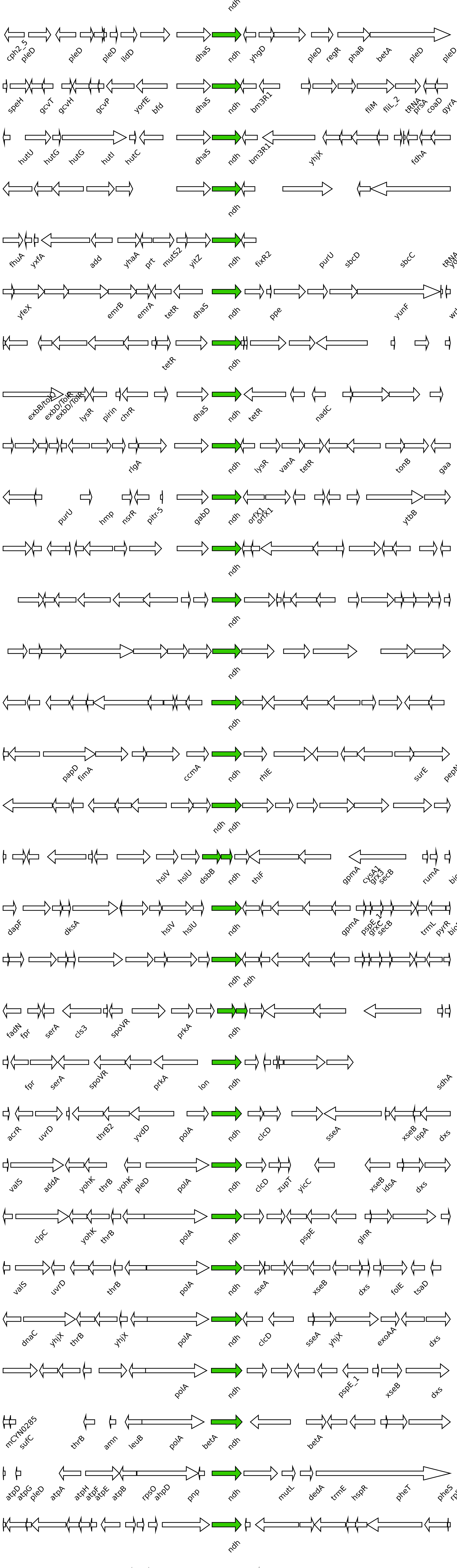

LXRJ01000088.1 ()

Paraburkholderia rhizoxinica HKI 454
CBW74495.1 (882378)
FR687359.1 (genomic DNA)

Janthinobacterium sp Marseille
ABR88937.1 (375286)
CP000269.1 (chromosome)

Lysobacter silvestris
PNS09469.1 (1645665)
NPZB01000001.1 (genomic DNA)

Robbsia andropogonis
KKB65431.1 (28092)
LAQU01000001.1 (genomic DNA)

Thioalbus denitrificans
RCX33196.1 (547122)
QPJY01000001.1 (genomic DNA)

Gallionellaceae bacterium UBA2239
DDYH01000041.1_75 (1953084)
DDYH01000041.1 ()

Betaproteobacteria bacterium UBA6910
DKBC01000010.1_2 (1948462)
DKBC01000010.1 ()

Thiomonas intermedia K12
ADG29562.1 (75379)
CP002021.1 (chromosome)

Thiomonas delicata
SBP88414.1 (364030)
LT592171.1 (genomic DNA)

Thiomonas sp FB 6
KB898514.1_24 (1158291)
KB898514.1 ()

Betaproteobacteria bacterium RIFCSPLOWO2_02_FULL_67_26
OGA25909.1 (1797492)
MERD01000016.1 (genomic DNA)

Proteobacteria bacterium CG1_02_64_396
OIO54185.1 (1805333)
MNWR01000103.1 (genomic DNA)

Methylotenera mobilis JLW8
ACT48946.1 (583345)
CP001672.1 (chromosome)

Methylotenera versatilis 301
ADI30820.1 (666681)
CP002056.1 (chromosome)

Betaproteobacteria bacterium HGW Betaproteobacteria 2
PKO53000.1 (2013711)
PHCM01000115.1 (genomic DNA)

Methylovorus glucosetrophus SIP3 4
ACT51586.1 (582744)
CP001674.1 (chromosome)

Methylobacillus glycogenes JCM 2850
BAMT01000002.1_280 (1236959)
BAMT01000002.1 ()

Methylobacillus flagellatus KT
ABE50578.1 (265072)
CP000284.1 (chromosome)

Proteobacteria bacterium ST_bin12
OQW70420.1 (1978760)
MSXP01000001.1 (genomic)

Candidatus Methylopumilus turicensis
CEN56607.1 (1581680)
LN794158.1 (genomic DNA)

Methylomonas clara
CP033953.1_2319 (417)
CP033953.1 ()

Methylophilaceae bacterium UBA1604
DCNE01000055.1_13 (1952234)
DCNE01000055.1 ()

Methylophilales bacterium 16 45 7
OYY98561.1 (1970536)
NCGS01000355.1 (genomic DNA)

Methylophilaceae bacterium
PCI52991.1 (2030816)
NVTX01000132.1 (genomic DNA)

Candidatus Methylopumilus planktonicus
CEZ20085.1 (1581557)
LN827929.1 (genomic DNA)

Proteobacteria bacterium UBA1858
DDIE01000044.1_38 (1948140)
DDIE01000044.1 ()

Methylophilales bacterium MBRSF5
AKO64846.1 (1623448)
CP011001.1 (chromosome)

Methylophilales bacterium HTCC2181
EAV46787.1 (383631)
AAUX01000001.1 (genomic DNA)

Methylobacillus sp MM3
OAJ70092.1 (1848039)
LXTQ01000040.1 (genomic DNA)

Novimethylophilus kurashikiensis
GBG15303.1 (1825523)
BDOQ01000018.1 (genomic DNA)

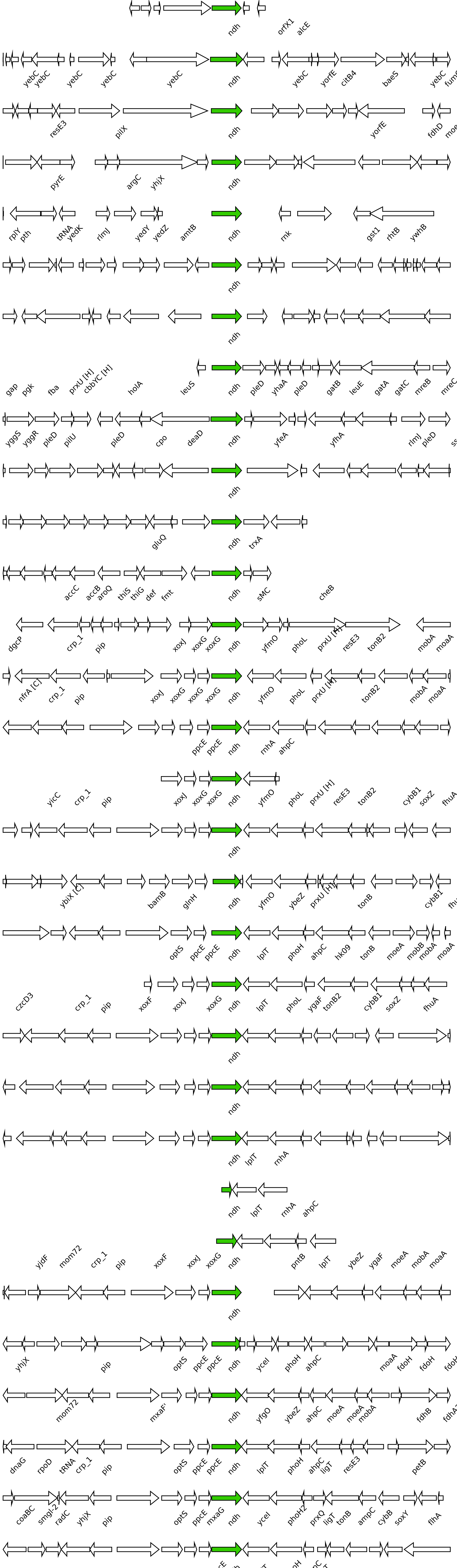

Methylophilales bacterium RIFCSPHIGHO2_02_FULL_57_10
OGV71287.1 (1801614)
MHBT01000133.1 (genomic DNA)

Variibacter gotjawalensis
BAT58891.1 (1333996)
AP014946.1 (genomic DNA)

Bradyrhizobium sp JGI 0001002 A22
AUOJ02000004.1_20 (1094929)
AUOJ02000004.1 ()

Rhodoblastus acidophilus
PPQ40617.1 (1074)
NRRJ01000001.1 (genomic DNA)

Hydromonas sp F02
AXF86209.1 (2268024)
CP031124.1 (chromosome)

beta proteobacterium AAP65
KPF47990.1 (1523424)
LJHW01000022.1 (genomic DNA)

Thauera phenolivorans
MBFM01000001.1_282 (1792543)
MBFM01000001.1 ()

Thauera sp MZ1T
ACR02311.1 (85643)
CP001281.2 (chromosome)

Polaromonas sp C04
OOG53177.1 (1945857)
MUNS01000031.1 (genomic)

Polynucleobacter acidiphobus
CP023277.1_667 (556053)
CP023277.1 ()

Polynucleobacter rarus
NTGB01000001.1_1399 (556055)
NTGB01000001.1 ()

Polynucleobacter asymbioticus QLW P1DMWA 1
ABP34207.1 (312153)
CP000655.1 (chromosome)

Betaproteobacteria bacterium HGW Betaproteobacteria 12
PKO86174.1 (2013703)
PHCU01000118.1 (genomic DNA)

Burkholderiales bacterium GJ E10
BAP87582.1 (1469502)
AP014683.1 (genomic DNA)

Ottowia oryzae
AVO33971.1 (2109914)
CP027666.1 (chromosome)

Granulibacter bethesdensis CGDNIH1
ABI62550.1 (391165)
CP000394.2 (chromosome)

Acidisphaera sp G45 3
CP029176.1_3446 (1969806)
CP029176.1 ()

Acidicaldus organivorans
KN173879.1_20 (209650)
KN173879.1 ()

Humitalea rosea
PZW48307.1 (990373)
QKYU01000005.1 (genomic DNA)

Rhodopseudomonas palustris CGA009
CAE26547.1 (258594)
BX571963.1 (genomic DNA)

Mongoliimonas terrestris
MSIG01000009.1_201 (1709001)
MSIG01000009.1 ()

Methylovirgula ligni
REF89667.1 (569860)
QUMO01000001.1 (genomic DNA)

Hyphomicrobium nitrativorans NL23
AHB47676.1 (1029756)
CP006912.1 (chromosome)

Hyphomicrobium sp 32 62 53
OYY01111.1 (1970390)
NCEW01000003.1 (genomic DNA)

Hyphomicrobium denitrificans 1NES1
AGK56568.1 (670307)
CP005587.1 (chromosome)

Hyphomicrobium sp NDB2Meth4
FPEJ01000001.1_247 (1892846)
FPEJ01000001.1 ()

Hyphomicrobium sulfonivorans
KWT64315.1 (121290)
LMTR01000093.1 (genomic DNA)

Methylorubrum extorquens PA1
ABY31032.1 (419610)
CP000908.1 (chromosome)

Methylobacterium sp Leaf456
KQT57188.1 (1736382)
LMQV01000012.1 (genomic DNA)

Methylobacterium gossipiicola
SFG26921.1 (582675)
FOPM01000001.1 (genomic DNA)

Arboricoccus pini
SNB64787.1 (1963835)

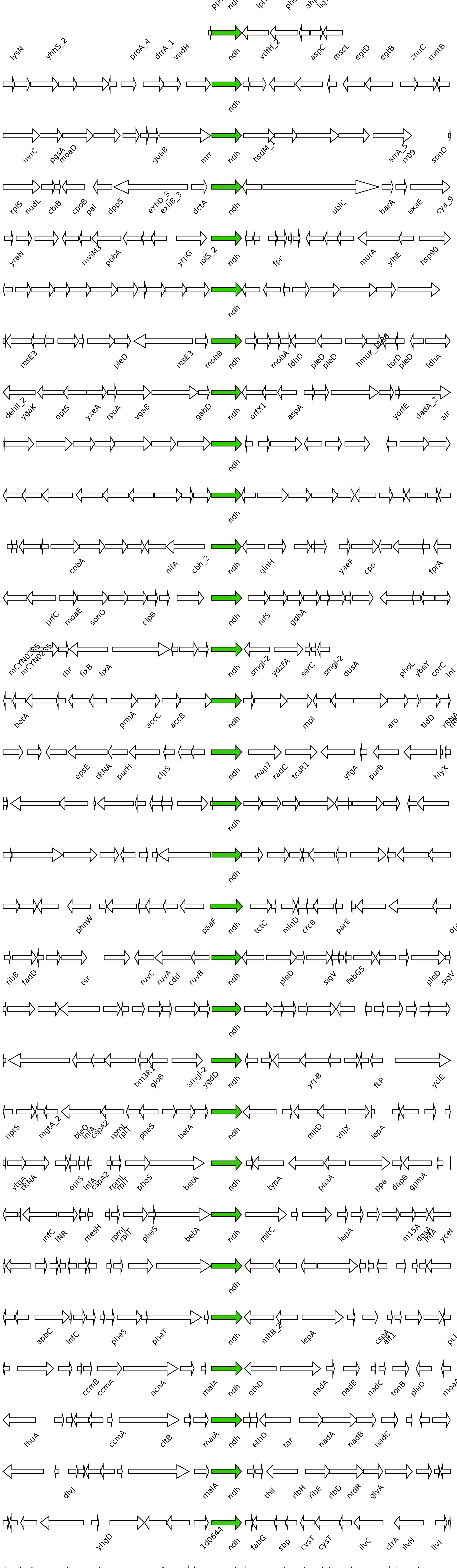

FYEH01000004.1 (genomic DNA)

Azospira oryzae
PZP52814.1 (146939)
QFOK01000612.1 (genomic DNA)

Pseudogulbenkiania sp NH8B
BAK77532.1 (748280)
AP012224.1 (genomic DNA)

Leeia oryzae DSM 17879
AQXU01000017.1_76 (1122158)
AQXU01000017.1 ()

Thiofilum flexile DSM 14609
KB904813.1_23 (1123400)
KB904813.1 ()

Thiothrix eikelboomii
SKA94954.1 (92487)
FUYB01000028.1 (genomic DNA)

Thiothrix nivea DSM 5205
EIJ34890.1 (870187)
JH651384.1 (genomic)

Methylovirgula sp 4M Z18
RFB80251.1 (2293567)
QUOY01000001.1 (genomic DNA)

Parendozoicomonas haliclonae
SMA34483.1 (1960125)
FWPT01000001.1 (genomic DNA)

Endozoicomonas montiporae CL 33
AMO57966.1 (570277)
CP013251.1 (chromosome)

Endozoicomonas elysicola
KEI72945.1 (305900)
JOJP01000001.1 (genomic DNA)

Endozoicomonas numazuensis
KEQ18970.1 (1137799)
JOKH01000001.1 (genomic DNA)

Pseudomonas sp LTJR 52
AYN95613.1 (2479392)
CP033105.1 (chromosome)

Pseudomonas thermotolerans DSM 14292
KB822620.1_58 (1123021)
KB822620.1 ()

Pseudomonas cremoricolorata
AIR88140.1 (157783)
CP009455.1 (chromosome)

Pseudomonas fragi
AMB78238.1 (296)
CP013861.1 (chromosome)

Pseudomonadaceae bacterium
MAB96462.1 (1914538)
NYTR01000002.1 (genomic DNA)

Pseudomonas mendocina ymp
ABP83533.1 (399739)
CP000680.1 (chromosome)

Pseudomonas fulva 12 X
AEF23753.1 (743720)
CP002727.1 (chromosome)

Pseudomonas guangdongensis
SDT89659.1 (1245526)
LT629780.1 (genomic DNA)

Pseudomonas stutzeri A1501
ABP81263.1 (379731)
CP000304.1 (chromosome)

Pseudomonas stutzeri SDM LAC
AGSX01000050.1_7 (1109445)
AGSX01000050.1 ()

Pseudomonadales bacterium 32 61 5
OYW95879.1 (1970544)
NCDF01000083.1 (genomic DNA)

Pseudomonas flexibilis
KHL70754.1 (706570)
JRUD01000002.1 (genomic DNA)

Pseudomonas hussainii
MPIO01000009.1_43 (1429083)
MPIO01000009.1 ()

Azotobacter chroococcum NCIMB 8003
AJE22470.1 (1328314)
CP010415.1 (chromosome)

Pseudomonas salegens
SDU24804.1 (1434072)
LT629787.1 (genomic DNA)

Pseudomonas sp S 6 2
AQZ96754.1 (1931241)
CP020100.1 (chromosome)

Pseudomonas xinjiangensis
SDR73124.1 (487184)
LT629736.1 (genomic DNA)

Pseudomonas pelagia
PCC98928.1 (553151)
NWMT01000175.1 (genomic DNA)

Pseudomonas sabulinigri
SDS36830.1 (472181)
LT629763.1 (genomic DNA)

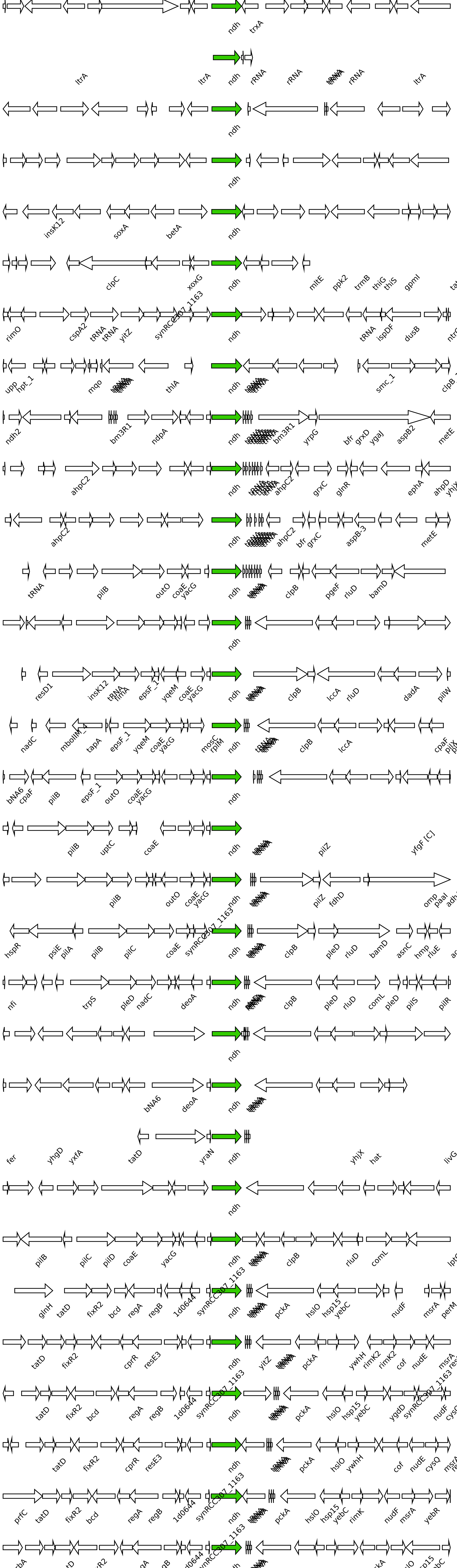

Pseudomonas litoralis
SDS74546.1 (797277)
LT629748.1 (genomic DNA)

Thiopseudomonas denitrificans
LNJZ01000008.1_166 (1501432)
LNJZ01000008.1 ()

Gammaproteobacteria bacterium DTU037
LFRZ01000068.1_4 (1671662)
LFRZ01000068.1 ()

Oblitimonas alkaliphila
AKX43900.1 (1697053)
CP012358.1 (chromosome)

Paludibacterium yongneupense DSM 18731
AUGZ01000018.1_108 (1122928)
AUGZ01000018.1 ()

Sulfurifustis variabilis
BAU47665.1 (1675686)
AP014936.1 (genomic DNA)

Sulfuricaulis limicola
BAV32616.1 (1620215)
AP014879.1 (genomic DNA)

Candidatus Muproteobacteria bacterium RBG_16_65_31
OGI45042.1 (1817759)
MFST01000023.1 (genomic DNA)

Gammaproteobacteria bacterium RIFCSPLOWO2_02_FULL_61_13
OGT80304.1 (1798300)
MGYT01000155.1 (genomic DNA)

Gammaproteobacteria bacterium RIFCSPLOWO2_12_47_11
OGT73334.1 (1798301)
MGYU01000075.1 (genomic DNA)

Thiotrichaceae bacterium UBA7359
DKOP01000030.1_1 (1953017)
DKOP01000030.1 ()

Thiotrichaceae bacterium UBA4486
DGNS01000005.1_396 (1953014)
DGNS01000005.1 ()

Sulfuriferula multivorans
GBL44353.1 (1559896)
BGOW01000001.1 (genomic DNA)

Hydrogenophilales bacterium 12 64 13
OYW63555.1 (1970517)
NCCW01000009.1 (genomic DNA)

Hydrogenophilales bacterium 12 61 10
OYW40712.1 (1970515)
NCCT01000002.1 (genomic DNA)

Hydrogenophilales bacterium 17 64 65
OZA35074.1 (1970528)
NCIQ01000003.1 (genomic DNA)

Thiobacillus denitrificans ATCC 25259
AAZ98173.1 (292415)
CP000116.1 (chromosome)

Thiobacillus sp UBA2186
DCVO01000116.1_15 (1947685)
DCVO01000116.1 ()

Proteobacteria bacterium SPGG2
LXTK01000241.1_11 (1842525)
LXTK01000241.1 ()

Gammaproteobacteria bacterium HGW Gammaproteobacteria 1
PKM44855.1 (2013791)
PGZL01000006.1 (genomic DNA)

Proteobacteria bacterium UBA3588
DFMR01000062.1_9 (1948171)
DFMR01000062.1 ()

Chromatiaceae bacterium 2141T STBD 0c 01a
CP019936.1_3062 (1978339)
CP019936.1 ()

Thiohalobacter thiocyanaticus
BAZ94344.1 (585455)
AP018052.1 (genomic DNA)

Nitrosococcus halophilus Nc 4
ADE13227.1 (472759)
CP001798.1 (chromosome)

Methylophaga lonarensis MPL
EMR13794.1 (1286106)
APHR01000014.1 (genomic DNA)

Methylophaga sulfidovorans
SFK05035.1 (45496)
FOSH01000004.1 (genomic DNA)

Methylophaga thiooxydans DMS010
EEF80018.1 (637616)
GG657897.1 (genomic)

Methylophaga frappieri
AFJ03136.1 (754477)
CP003380.1 (chromosome)

Methylophaga nitratireducenticrescens
AFI83174.1 (754476)
CP003390.3 (chromosome)

Methylophaga sp 42_8_T64
OUR86345.1 (1856286)
MAAW01000090.1 (genomic DNA)

Methylophaga sp 41_12_T18
OUR73037.1 (1856284)

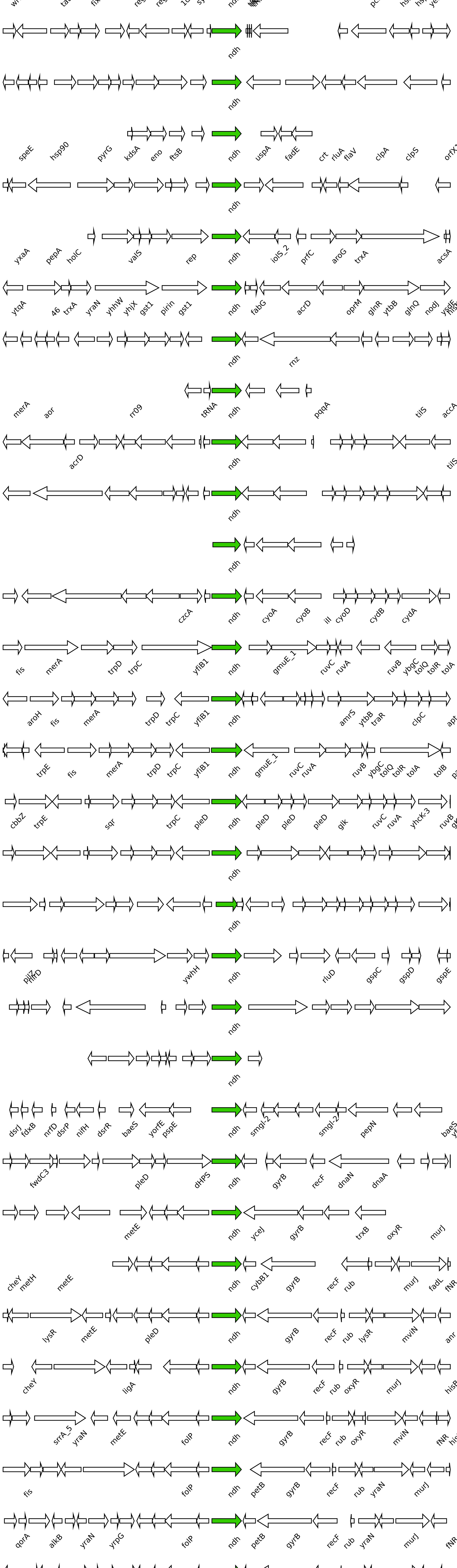

MAAI01000035.1 (genomic DNA)

Proteobacteria bacterium UBA651
DBOH01000086.1_1 (1948194)
DBOH01000086.1 ()

Methylocaldum szegediense O 12
KE386489.1_266 (675524)
KE386489.1 ()

Methylomagnum ishizawai
SMF96298.1 (1760988)
FXAM01000001.1 (genomic DNA)

Methyloterricola oryzae
JYNS01000002.1_378 (1495050)
JYNS01000002.1 ()

Methylococcus capsulatus str Bath
AAU92100.1 (243233)
AE017282.2 (chromosome)

Candidatus Thiodiazotropha endoloripes
ODB87277.1 (1818881)
LVJY01000005.1 (genomic DNA)

Candidatus Thiodiazotropha endolucinida
ODJ87952.1 (1655433)
MARB01000008.1 (genomic)

Methylococcaceae bacterium UBA1147
DBZZ01000017.1_11 (1952219)
DBZZ01000017.1 ()

Methylococcales bacterium OPU3_GD_OMZ
MPSY01000017.1_1 (1920888)
MPSY01000017.1 ()

Methylomarinum vadi
JPON01000001.1_2045 (438855)
JPON01000001.1 ()

Gammaproteobacteria bacterium HGW Gammaproteobacteria 3
PKM11362.1 (2013799)
PGZD01000040.1 (genomic DNA)

Methylomicrobium alcaliphilum 20Z
CCE22976.1 (1091494)
FO082060.1 (genomic DNA)

Methylobacter tundripaludum SV96
EGW22848.1 (697282)
JH109152.1 (genomic)

Methylobacter luteus IMV B 3098
KE386569.1_3296 (1095552)
KE386569.1 ()

Methylococcaceae bacterium NSP1 2
OYV20944.1 (1917481)
NQJH01000017.1 (genomic DNA)

Methylococcaceae bacterium NSP1 1
OYV16543.1 (1917480)
NQJG01000253.1 (genomic DNA)

Methylococcaceae bacterium UBA6146
DIUY01000001.1_139 (1952225)
DIUY01000001.1 ()

Methylovulum psychrotolerans
ASF45428.1 (1704499)
CP022129.1 (chromosome)

Methylosarcina fibrata AML C10
KB889965.1_127 (1132443)
KB889965.1 ()

Methylococcaceae bacterium UBA7658
DLHW01000071.1_16 (1952228)
DLHW01000071.1 ()

Methylococcaceae bacterium
PMDM01000134.1_6 (1933926)
PMDM01000134.1 ()

Methyloglobulus morosus KoM1
ESS66224.1 (1116472)
AYLO01000175.1 (genomic)

Methylomicrobium album BG8
EIC30717.1 (686340)
CM001475.1 (chromosome)

Crenothrix polyspora
SJM94501.1 (360316)
FUKJ01000335.1 (genomic DNA)

Methylococcaceae bacterium UBA662
DBNW01000079.1_14 (1952227)
DBNW01000079.1 ()

Methylococcaceae bacterium UBA659
DBNZ01000185.1_18 (1952226)
DBNZ01000185.1 ()

Methylomonas methanica
OAH96448.1 (421)
LUUG01000133.1 (genomic DNA)

Methylomonas methanica MC09
AEF98812.1 (857087)
CP002738.1 (chromosome)

Methylomonas lenta
OAI21566.1 (980561)
LUUI01000008.1 (genomic DNA)

Methylococcaceae bacterium
PMDM01000093.1_1 (1933926)
PMDM01000093.1 ()

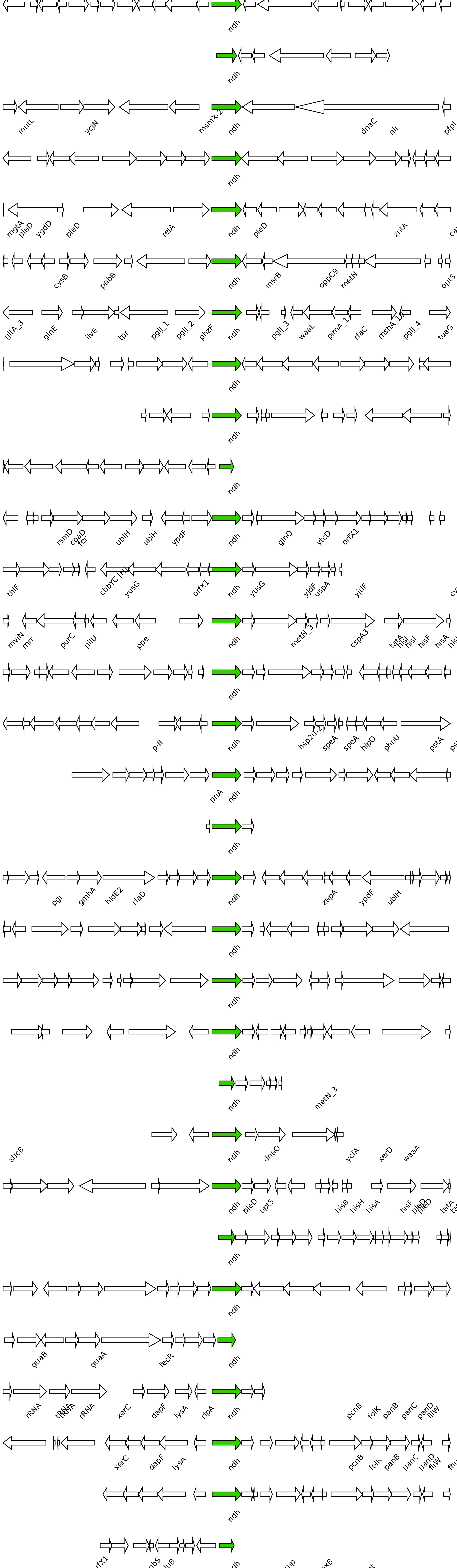

Methyloprofundus sedimenti
OQK17198.1 (1420851)
LPUF01000001.1 (genomic DNA)

Proteobacteria bacterium UBA664
DBNU01000078.1_8 (1948198)
DBNU01000078.1 ()

Gammaproteobacteria bacterium UBA3977
DGCN01000036.1_35 (1948612)
DGCN01000036.1 ()

Mangrovitalea sediminis
NTLB01000002.1_121 (1982043)
NTLB01000002.1 ()

Tamilnaduibacter salinus
PAV26131.1 (1484056)
NMPM01000037.1 (genomic DNA)

Marinobacter sp X15 166B
OEY66198.1 (1897620)
MEIY01000001.1 (genomic DNA)

Marinobacter mobilis
SDW73837.1 (488533)
FNNE01000004.1 (genomic DNA)

Marinobacter mobilis
SDX51499.1 (488533)
FNNE01000010.1 (genomic DNA)

Marinobacter sp PWS21
PHQ15263.1 (2033727)
KZ319370.1 (genomic)

Marinobacter persicus
PPK56198.1 (930118)
PTIU01000002.1 (genomic DNA)

Marinobacter hydrocarbonoclasticus VT8
ABM17247.1 (351348)
CP000514.1 (chromosome)

Marinobacter fuscus
PSF13419.1 (2109942)
PXNP01000009.1 (genomic DNA)

Marinobacter halophilus
PSF10079.1 (1323740)
PXNN01000003.1 (genomic DNA)

Marinobacter nanhaiticus D15 8W
ENO15090.1 (626887)
KB822693.1 (genomic)

Marinobacter sp UBA2698
DELK01000026.1_47 (1946817)
DELK01000026.1 ()

Marinobacter sp BSs20148
AFP29075.1 (490759)
CP003735.1 (chromosome)

Marinobacter daqiaonensis
SFR66212.1 (650891)
FOYW01000001.1 (genomic DNA)

Oleiphilus sp HI0071
KZY82629.1 (1822246)
LWFL01001547.1 (genomic DNA)

Oleiphilus sp HI0125
KZZ63177.1 (1822266)
LWGF01000057.1 (genomic DNA)

Acidihalobacter ferrooxidans
APZ42010.1 (1765967)
CP019434.1 (chromosome)

Metallibacterium scheffleri
LDOS01000002.1_1461 (993689)
LDOS01000002.1 ()

Betaproteobacteria bacterium GR16 43
APV51863.1 (1904640)
CP019169.1 (chromosome)

Proteobacteria bacterium UBA964
DBCG01000141.1_15 (1948225)
DBCG01000141.1 ()

Polaromonas naphthalenivorans CJ2
ABM36595.1 (365044)
CP000529.1 (chromosome)

Polaromonas sp SP1
AYQ28868.1 (2268087)
CP031013.1 (chromosome)

Polaromonas sp C04
OOG56027.1 (1945857)
MUNS01000025.1 (genomic)

Gulbenkiania indica
LION01000006.1_93 (375574)
LION01000006.1 ()

Chitinimonas koreensis DSM 17726
KE386750.1_164 (1121278)
KE386750.1 ()

Chitinimonas taiwanensis DSM 18899
SFZ79211.1 (1121279)
FPKR01000015.1 (genomic DNA)

Candidatus Dactylopiibacterium carminicum
PAS98259.1 (857335)
NMRM01000003.1 (genomic DNA)

Parvibium lacunae
RCS59523.1 (1888893)

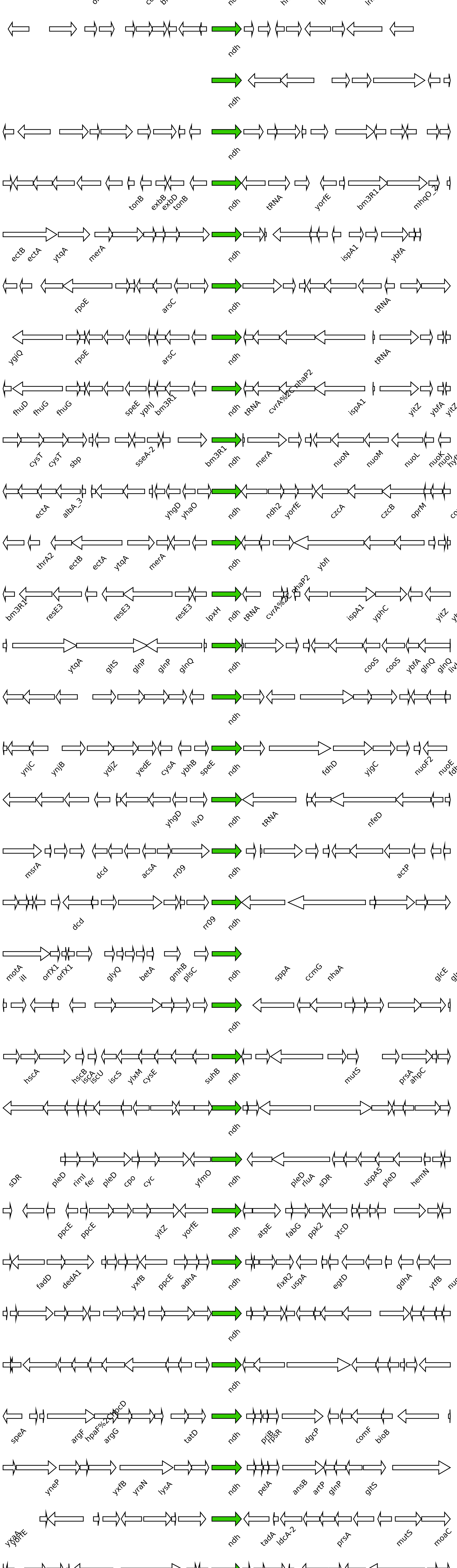

QPGB01000001.1 (genomic DNA)

Noviherbaspirillum autotrophicum
KIF81433.1 (709839)
JWJG01000028.1 (genomic DNA)

Solimicrobium silvestre
PRC94607.1 (2099400)
PUGF01000002.1 (genomic DNA)

Undibacterium parvum
AZP14560.1 (401471)
CP034464.1 (chromosome)

Undibacterium parvum
AZP14672.1 (401471)
CP034464.1 (chromosome)

Iodobacter sp H11R3
AZN35043.1 (2496266)
CP034433.1 (chromosome)

Burkholderiales bacterium RIFCSPLOWO2_12_FULL_61_40
OGB29284.1 (1797566)
MESI01000111.1 (genomic DNA)

Ketobacter alkanivorans
AUM12095.1 (1917421)
CP022684.1 (chromosome)

Gammaproteobacteria bacterium BRH_c0
KJS06472.1 (1629722)
LADM01000087.1 (genomic DNA)

Ketobacter sp
RLP53915.1 (2083498)
RAHY01000011.1 (genomic DNA)

Agitococcus lubricus
PTQ88489.1 (1077255)
QAON01000011.1 (genomic DNA)

Acinetobacter sp WCHAc060096
RKG30988.1 (2419602)
RAXU01000025.1 (genomic DNA)

Acinetobacter sp ADP1
CAG70265.1 (62977)
CR543861.1 (genomic DNA)

Alkanindiges illinoisensis DSM 15370
JHUX01000003.1_1047 (1120977)
JHUX01000003.1 ()

Moraxellaceae bacterium HYN0046
AXI02515.1 (2283318)
CP031222.1 (chromosome)

Acinetobacter puyangensis
SNX43977.1 (1096779)
OANT01000002.1 (genomic DNA)

Moraxellaceae bacterium REDSEA S42_B15
LUOU01000205.1_2 (1811615)
LUOU01000205.1 ()

Acinetobacter larvae
AOA58068.1 (1789224)
CP016895.1 (chromosome)

Acinetobacter sp UBA5927
DJDJ01000036.1_48 (1945945)
DJDJ01000036.1 ()

Acinetobacter guillouiae
BBRY01000022.1_33 (106649)
BBRY01000022.1 ()

Acinetobacter lwoffii
BBSQ01000022.1_51 (28090)
BBSQ01000022.1 ()

Acinetobacter pittii PHEA 2
ADY82539.1 (871585)
CP002177.1 (chromosome)

Acinetobacter nectaris CIP 110549
ESK37790.1 (1392540)
KI530735.1 (genomic)

Acinetobacter boissieri
SDB89523.1 (1219383)
FMYL01000004.1 (genomic DNA)

Acinetobacter apis
SNQ28978.1 (1229165)
FZLN01000001.1 (genomic DNA)

gamma proteobacterium HTCC5015
EDY85976.1 (391615)
DS990607.1 (genomic)

Thiotrichaceae bacterium UBA5859
DJFZ01000016.1_17 (1953015)
DJFZ01000016.1 ()

Oceanospirillales bacterium WDS2A16A
QPEP01000011.1_122 (1853216)
QPEP01000011.1 ()

Halomonas utahensis
LT727794.1_1785 (86177)
LT727794.1 ()

Parahaliea mediterranea
QEWO01000001.1_459 (651086)
QEWO01000001.1 ()

Cellvibrionaceae bacterium UBA677
DBNH01000083.1_70 (1951542)
DBNH01000083.1 ()

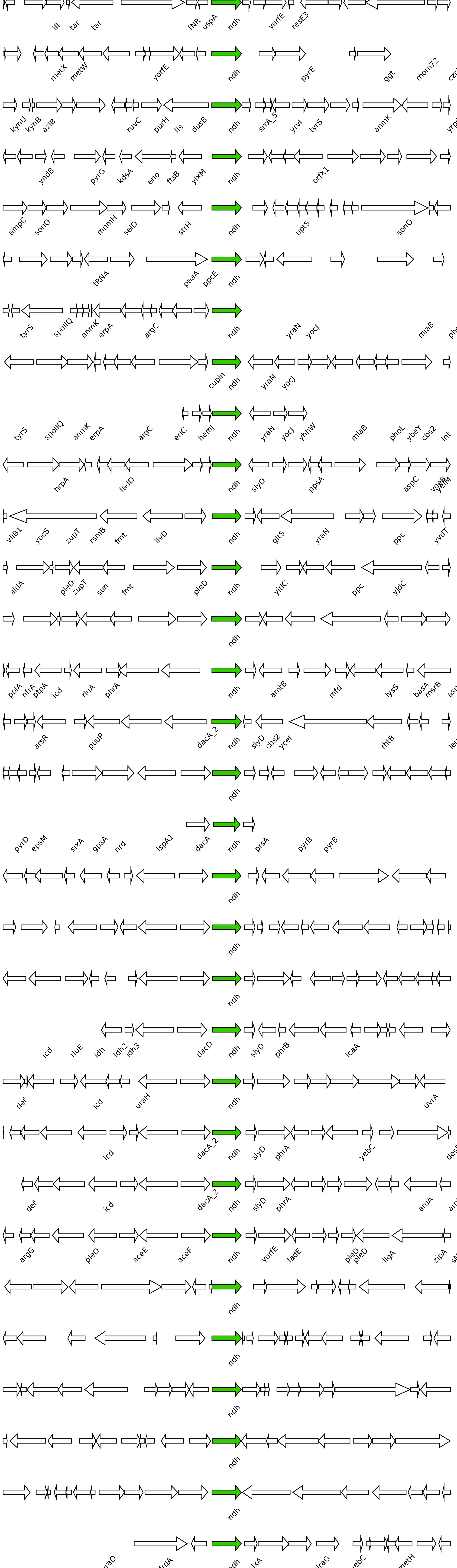

Ferrimonas marina
SHH78350.1 (299255)
FQXG01000004.1 (genomic DNA)

Oceanicoccus sp KOV_DT_Chl
FQLF01000007.1_252 (1904639)
FQLF01000007.1 ()

Pokkaliibacter plantistimulans
PXF30020.1 (1635171)
LAPT01000088.1 (genomic DNA)

Balneatrix alpica DSM 16621
KK211040.1_361 (1121103)
KK211040.1 ()

Rheinheimera sp LHK132
CP034683.1_112 (2498451)
CP034683.1 ()

Rheinheimera salexigens
OEY68243.1 (1628148)
MKEK01000001.1 (genomic DNA)

Arsukibacterium sp MJ3
KKO48451.1 (1632859)
LAHP01000014.1 (genomic DNA)

Simiduia agarivorans SA1 DSM 21679
AFU98309.2 (1117647)
CP003746.2 (chromosome)

Vespertiliibacter pulmonis
RPE85641.1 (1443036)
RKQP01000001.1 (genomic DNA)

Glaesserella parasuis SH0165
ACL33209.1 (557723)
CP001321.1 (chromosome)

Mannheimia haemolytica
KIX28792.1 (75985)
JPIZ01000031.1 (genomic DNA)

Haemophilus ducreyi 35000HP
AAP95760.1 (233412)
AE017143.1 (chromosome)

Actinobacillus pleuropneumoniae serovar 5b str L20
ABN74357.1 (416269)
CP000569.1 (chromosome)

Pasteurella testudinis DSM 23072
SMB80183.1 (1122938)
FWWV01000002.1 (genomic DNA)

Aggregatibacter actinomycetemcomitans
AMQ91682.1 (714)
CP012958.1 (chromosome)

Mannheimia succiniciproducens MBEL55E
AAU36788.1 (221988)
AE016827.1 (chromosome)

Actinobacillus succinogenes 130Z
ABR75327.1 (339671)
CP000746.1 (chromosome)

Conservatibacter flavescens
PJG84372.1 (28161)
PHHA01000034.1 (genomic DNA)

Haemophilus influenzae Rd KW20
AAC22405.1 (71421)
L42023.1 (chromosome)

Motilimonas sp PLHSC7 2
RJG41989.1 (2303987)
QZCH01000022.1 (genomic DNA)

Psychromonas sp B3M02
RBW47168.1 (2267226)
QOCB01000032.1 (genomic DNA)

Psychromonas ingrahamii 37
ABM03753.1 (357804)
CP000510.1 (chromosome)

Psychromonas aquimarina ATCC BAA 1526
KE386791.1_238 (1278312)
KE386791.1 ()

Psychromonas hadalis ATCC BAA 638
ATUO01000026.1_9 (1278302)
ATUO01000026.1 ()

Psychromonas sp psych 6C06
PKF63827.1 (2058089)
PIZM01000001.1 (genomic DNA)

Psychromonas sp CNPT3
AGH81124.1 (314282)
CP004404.1 (chromosome)

Tolumonas auensis DSM 9187
ACQ93151.1 (595494)
CP001616.1 (chromosome)

Aeromonas hydrophila subsp hydrophila ATCC 7966
ABK37387.1 (380703)
CP000462.1 (chromosome)

Aeromonas sp RU39B
SIQ19884.1 (1907416)
FTMJ01000002.1 (genomic DNA)

Aliidiomarina taiwanensis
RUO37145.1 (946228)
PIPQ01000012.1 (genomic)

Zobellella maritima
QCZE01000001.1_496 (2059725)

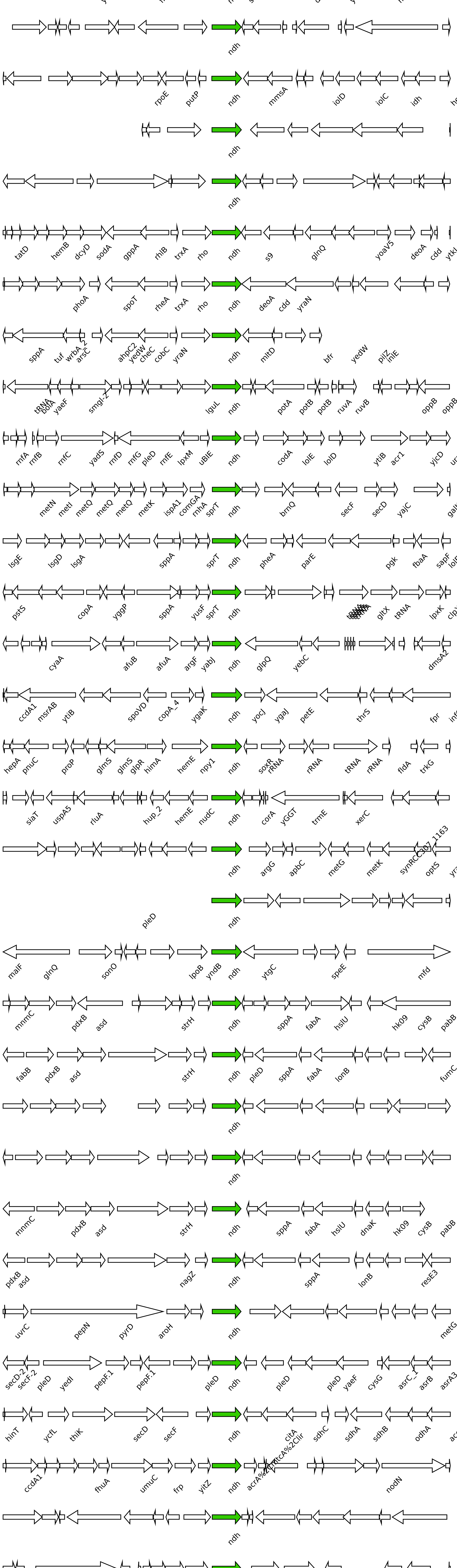

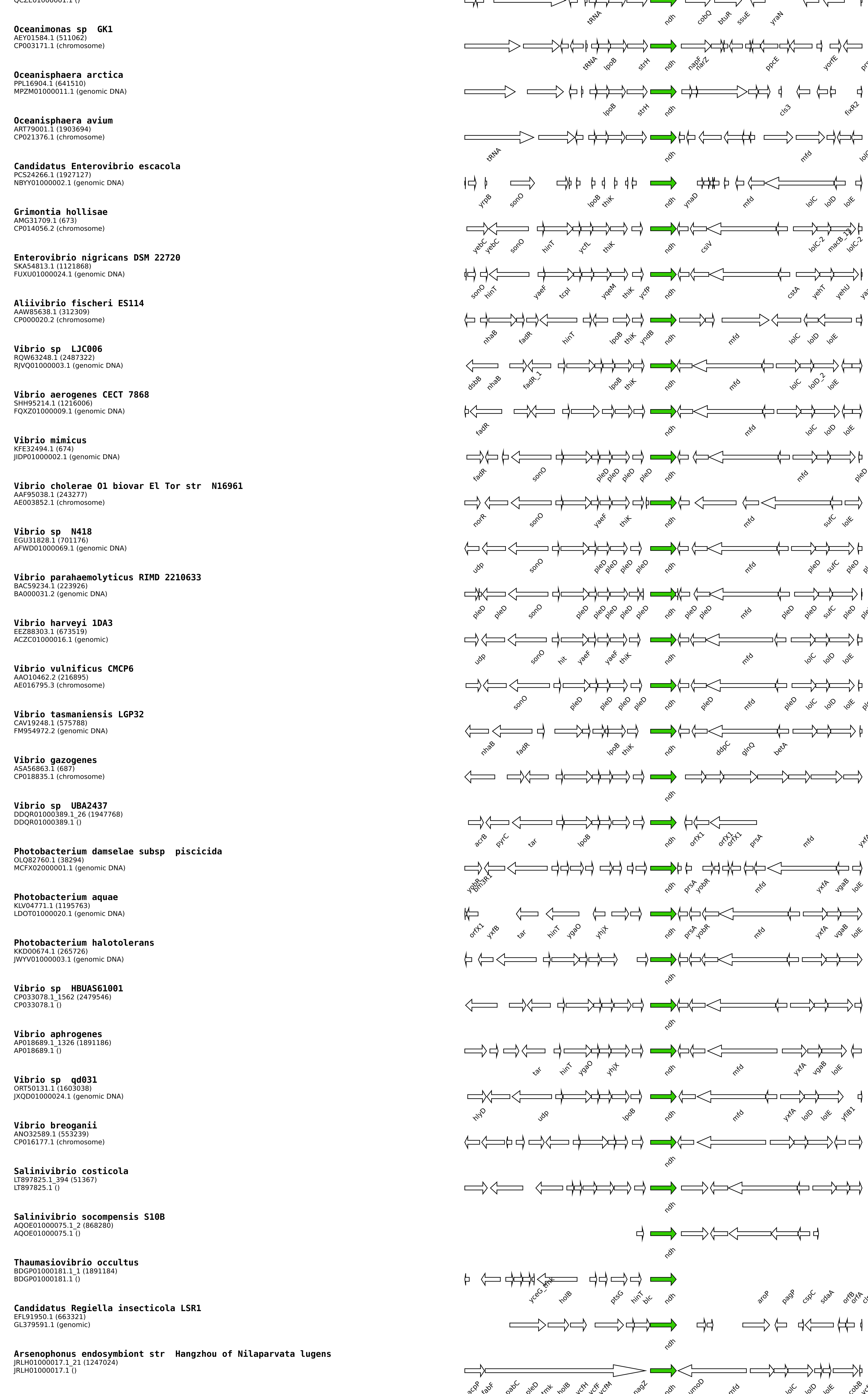

QCZE01000001.1 ()
tRNA ndh cobQ btuR ssuE yraN
Oceanimonas sp GK1
AEY01584.1 (511062)
CP003171.1 (chromosome)
tRNA lpoB strH ndh napF flaZ ppcE yorfE
Oceanisphaera arctica
PPL16904.1 (641510)
MPZM01000011.1 (genomic DNA)
lpoB strH ndh cls3 fixR2
Oceanisphaera avium
ART79001.1 (1903694)
CP021376.1 (chromosome)
tRNA ndh mfd
Candidatus Enterovibrio escacola
PCS24266.1 (1927127)
NBYY01000002.1 (genomic DNA)
yrpB sonO lpoB thiK ndh ynaD mfd lolC lolD lolE
Grimontia hollisae
AMG31709.1 (673)
CP014056.2 (chromosome)
yebC yebC sonO hinT ycfL thiK ndh csiV lolC-2 macB_12 lolC-2
Enterovibrio nigricans DSM 22720
SKA54813.1 (1121868)
FUXU01000024.1 (genomic DNA)
sonO hinT yaeF tcpI yqeM thiK ycfP ndh cstA yehT yehU
Aliivibrio fischeri ES114
AAW85638.1 (312309)
CP000020.2 (chromosome)
nhaB fadR hinT lpoB thiK yndB ndh mfd lolC lolD lolE
Vibrio sp LJC006
RQW63248.1 (2487322)
RJVQ01000003.1 (genomic DNA)
dsbB nhaB fadR_1 lpoB thiK ndh mfd lolC lolD_2 lolE
Vibrio aerogenes CECT 7868
SHH95214.1 (1216006)
FQXZ01000009.1 (genomic DNA)
fadR ndh mfd lolC lolD lolE
Vibrio mimicus
KFE32494.1 (674)
JIDP01000002.1 (genomic DNA)
fadR sonO pleD pleD pleD pleD ndh mfd pleD
Vibrio cholerae O1 biovar El Tor str N16961
AAF95038.1 (243277)
AE003852.1 (chromosome)
norR sonO yaeF thiK ndh mfd sufC lolE
Vibrio sp N418
EGU31828.1 (701176)
AFWD01000069.1 (genomic DNA)
udp sonO pleD pleD pleD pleD ndh mfd pleD sufC pleD
Vibrio parahaemolyticus RIMD 2210633
BAC59234.1 (223926)
BA000031.2 (genomic DNA)
pleD pleD sonO pleD pleD pleD pleD pleD ndh pleD pleD mfd pleD pleD sufC pleD
Vibrio harveyi 1DA3
EEZ88303.1 (673519)
ACZC01000016.1 (genomic)
udp sonO hit yaeF yaeF thiK ndh mfd lolC lolD lolE
Vibrio vulnificus CMCP6
AAO10462.2 (216895)
AE016795.3 (chromosome)
sonO pleD pleD pleD pleD ndh pleD mfd pleD lolC lolD lolE
Vibrio tasmaniensis LGP32
CAV19248.1 (575788)
FM954972.2 (genomic DNA)
nhaB fadR lpoB thiK ndh ddpC glnQ betA
Vibrio gazogenes
ASA56863.1 (687)
CP018835.1 (chromosome)
ndh
Vibrio sp UBA2437
DDQR01000389.1_26 (1947768)
DDQR01000389.1 ()
acrB pyrC tar lpoB ndh orfX1 orfX1 orfX1 prsA mfd
Photobacterium damselae subsp piscicida
OLQ82760.1 (38294)
MCFX02000001.1 (genomic DNA)
yobR bm3R1 ndh prsA yobR mfd yxfA vgaB lolE
Photobacterium aquae
KLV04771.1 (1195763)
LDOT01000020.1 (genomic DNA)
orfX1 yxfB tar hinT ygaO yhjX ndh prsA yobR mfd yxfA vgaB lolE
Photobacterium halotolerans
KKD00674.1 (265726)
JWYV01000003.1 (genomic DNA)
ndh
Vibrio sp HBUAS61001
CP033078.1_1562 (2479546)
CP033078.1 ()
ndh
Vibrio aphrogenes
AP018689.1_1326 (1891186)
AP018689.1 ()
tar hinT ygaO yhjX ndh mfd yxfA vgaB lolE
Vibrio sp qd031
ORT50131.1 (1603038)
JXQD01000024.1 (genomic DNA)
hlyD udp lpoB ndh mfd yxfA lolD lolE yfiB1
Vibrio breoganii
ANO32589.1 (553239)
CP016177.1 (chromosome)
ndh
Salinivibrio costicola
LT897825.1_394 (51367)
LT897825.1 ()
ndh
Salinivibrio socompensis S10B
AQOE01000075.1_2 (868280)
AQOE01000075.1 ()
ndh
Thaumasiovibrio occultus
BDGP01000181.1_1 (1891184)
BDGP01000181.1 ()
yceG_tmk holB ptsG hinT blc ndh aroP pagP cspC sdaA orfB orfA
Candidatus Regiella insecticola LSR1
EFL91950.1 (663321)
GL379591.1 (genomic)
ndh
Arsenophonus endosymbiont str Hangzhou of Nilaparvata lugens
JRLH01000017.1_21 (1247024)
JRLH01000017.1 ()
acpP fabF pabC pleD tmk holB ycfH ycfF ycfM nagZ ndh umoD mfd lolC lolD lolE cobB

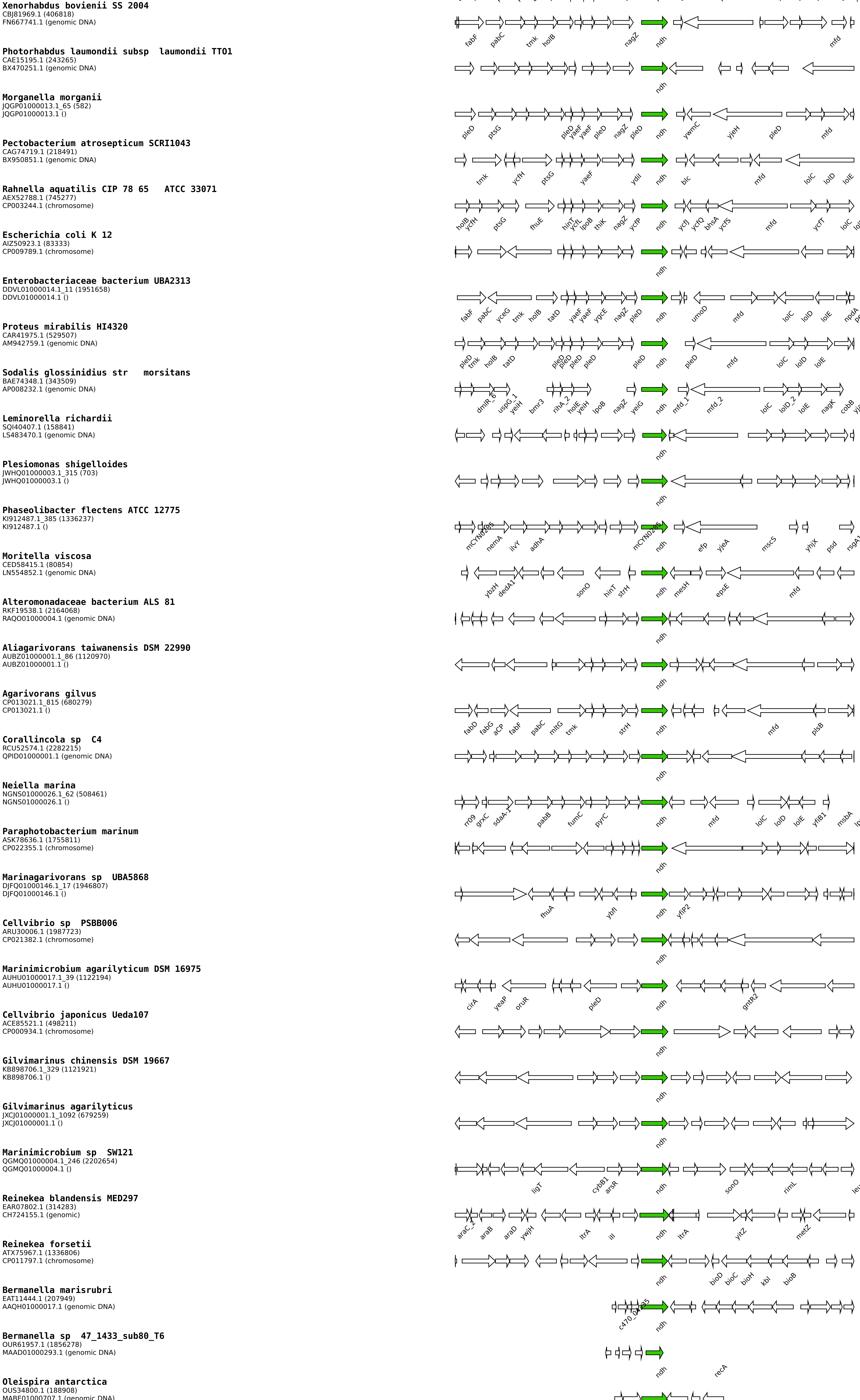
Xenorhabdus bovienii SS 2004
CBJ81969.1 (406818)
FN667741.1 (genomic DNA)
Photorhabdus laumondii subsp laumondii TTO1
CAE15195.1 (243265)
BX470251.1 (genomic DNA)
Morganella morganii
JQGP01000013.1_65 (582)
JQGP01000013.1 ()
Pectobacterium atrosepticum SCRI1043
CAG74719.1 (218491)
BX950851.1 (genomic DNA)
Rahnella aquatilis CIP 78 65 ATCC 33071
AEX52788.1 (745277)
CP003244.1 (chromosome)
Escherichia coli K 12
AIZ50923.1 (83333)
CP009789.1 (chromosome)
Enterobacteriaceae bacterium UBA2313
DDVL01000014.1_11 (1951658)
DDVL01000014.1 ()
Proteus mirabilis HI4320
CAR41975.1 (529507)
AM942759.1 (genomic DNA)
Sodalis glossinidius str morsitans
BAE74348.1 (343509)
AP008232.1 (genomic DNA)
Leminorella richardii
SQI40407.1 (158841)
LS483470.1 (genomic DNA)
Plesiomonas shigelloides
JWHQ01000003.1_315 (703)
JWHQ01000003.1 ()
Phaseolibacter flectens ATCC 12775
KI912487.1_385 (1336237)
KI912487.1 ()
Moritella viscosa
CED58415.1 (80854)
LN554852.1 (genomic DNA)
Alteromonadaceae bacterium ALS 81
RKF19538.1 (2164068)
RAQO01000004.1 (genomic DNA)
Aliagarivorans taiwanensis DSM 22990
AUBZ01000001.1_86 (1120970)
AUBZ01000001.1 ()
Agarivorans gilvus
CP013021.1_815 (680279)
CP013021.1 ()
Corallincola sp C4
RCU52574.1 (2282215)
QPID01000001.1 (genomic DNA)
Neiella marina
NGNS01000026.1_62 (508461)
NGNS01000026.1 ()
Paraphotobacterium marinum
ASK78636.1 (1755811)
CP022355.1 (chromosome)
Marinagarivorans sp UBA5868
DJFQ01000146.1_17 (1946807)
DJFQ01000146.1 ()
Cellvibrio sp PSBB006
ARU30006.1 (1987723)
CP021382.1 (chromosome)
Marinimicrobium agarilyticum DSM 16975
AUHU01000017.1_39 (1122194)
AUHU01000017.1 ()
Cellvibrio japonicus Ueda107
ACE85521.1 (498211)
CP000934.1 (chromosome)
Gilvimarinus chinensis DSM 19667
KB898706.1_329 (1121921)
KB898706.1 ()
Gilvimarinus agarilyticus
JXCJ01000001.1_1092 (679259)
JXCJ01000001.1 ()
Marinimicrobium sp SW121
QGMQ01000004.1_246 (2202654)
QGMQ01000004.1 ()
Reinekea blandensis MED297
EAR07802.1 (314283)
CH724155.1 (genomic)
Reinekea forsetii
ATX75967.1 (1336806)
CP011797.1 (chromosome)
Bermanella marisrubri
EAT11444.1 (207949)
AAQH01000017.1 (genomic DNA)
Bermanella sp 47_1433_sub80_T6
OUR61957.1 (1856278)
MAAD01000293.1 (genomic DNA)
Oleispira antarctica
OUS34800.1 (188908)
MABE01000707.1 (genomic DNA)

Oleibacter marinus
SIS86028.1 (484498)
FTOH01000005.1 (genomic DNA)

Oceanobacter sp
PHR96949.1 (2030817)
NVWL01000049.1 (genomic DNA)

Bacterioplanes sanyensis
ASP38777.1 (1249553)
CP022530.1 (chromosome)

Thalassolituus sp HI0120
KZZ44621.1 (1822263)
LWGC01001441.1 (genomic DNA)

Thalassolituus sp HI0120
KZZ43401.1 (1822263)
LWGC01001587.1 (genomic DNA)

Thalassolituus oleivorans R6 15
AHK16712.1 (1208320)
CP006829.1 (chromosome)

Saccharospirillum impatiens DSM 12546
AUIH01000038.1_15 (1123228)
AUIH01000038.1 ()

Saccharospirillum mangrovi
QAPE01000011.1_85 (2161747)
QAPE01000011.1 ()

Marinobacterium aestuarii
ANG62282.1 (1821621)
CP015839.1 (chromosome)

Marinobacterium stanieri
SIQ22096.1 (49186)
FTMN01000003.1 (genomic DNA)

Marinobacterium georgiense DSM 11526
SEA37293.1 (1122198)
FNRJ01000003.1 (genomic DNA)

Marinobacterium sp
QKGH01000190.1_9 (1902815)
QKGH01000190.1 ()

Marinobacterium litorale DSM 23545
AUAZ01000017.1_15 (1122201)
AUAZ01000017.1 ()

Nitrincola nitratireducens
EXJ10812.1 (1229521)
AONB01000010.1 (genomic DNA)

Pseudoalteromonas sp T1lg22
PQBY01000016.1_384 (2077096)
PQBY01000016.1 ()

Neptunomonas phycophila
MRCJ01000003.1_99 (1572645)
MRCJ01000003.1 ()

Leucothrix pacifica
PWQ98243.1 (1247513)
QGKM01000017.1 (genomic DNA)

Leucothrix sp IMCC9719
PWQ97542.1 (1481894)
QGKL01000019.1 (genomic DNA)

Leucothrix mucor DSM 2157
ATTE01000001.1_1509 (998674)
ATTE01000001.1 ()

Neptunomonas qingdaonensis
SFG38849.1 (1045558)
FOOU01000006.1 (genomic DNA)

Neptunomonas antarctica
LJSI01000050.1_95 (619304)
LJSI01000050.1 ()

Amphritea japonica ATCC BAA 1530
KB907103.1_179 (1278309)
KB907103.1 ()

Amphritea atlantica
SEQ69638.1 (355243)
FOGB01000006.1 (genomic DNA)

Amphritea balenae
RRC97717.1 (452629)
RQXV01000010.1 (genomic)

gamma proteobacterium IMCC2047
EGG99665.1 (434085)
AEGL01000196.1 (genomic DNA)

Marinomonas sp QM202
NHNK01000001.1_131 (1902503)
NHNK01000001.1 ()

Marinomonas fungiae
LIQF01000003.1_409 (1137284)
LIQF01000003.1 ()

Marinomonas mediterranea MMB 1
ADZ91521.1 (717774)
CP002583.1 (chromosome)

Marinomonas sp MWYL1
ABR71179.1 (400668)
CP000749.1 (chromosome)

Marinobacterium jannaschii DSM 6295
JHVJ01000019.1_79 (1122200)
JHVJ01000019.1 ()

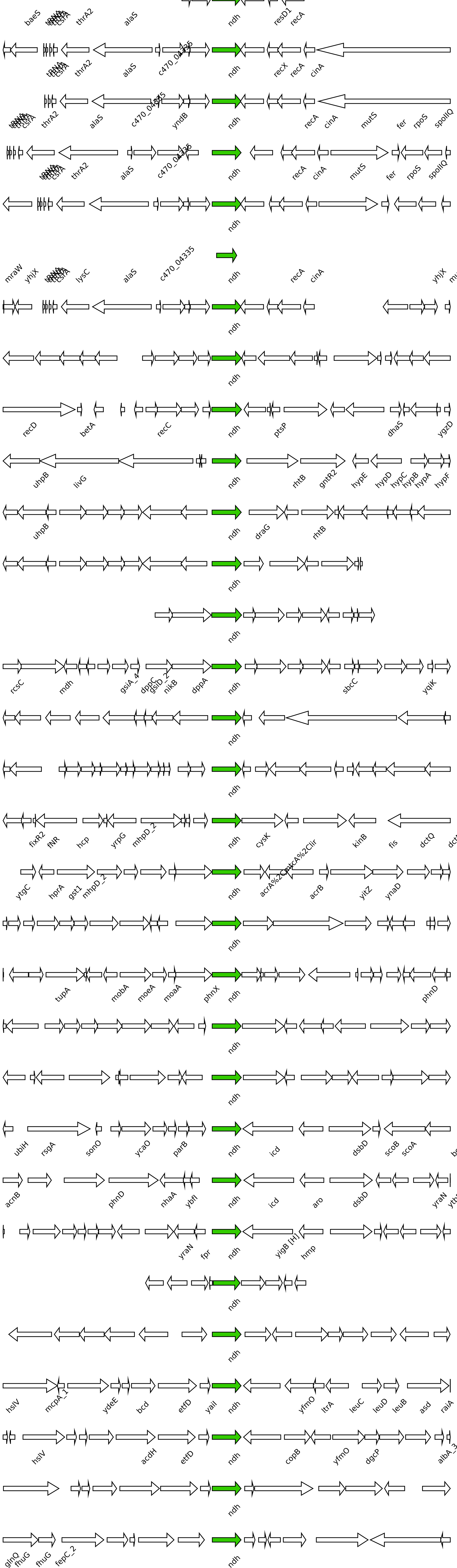

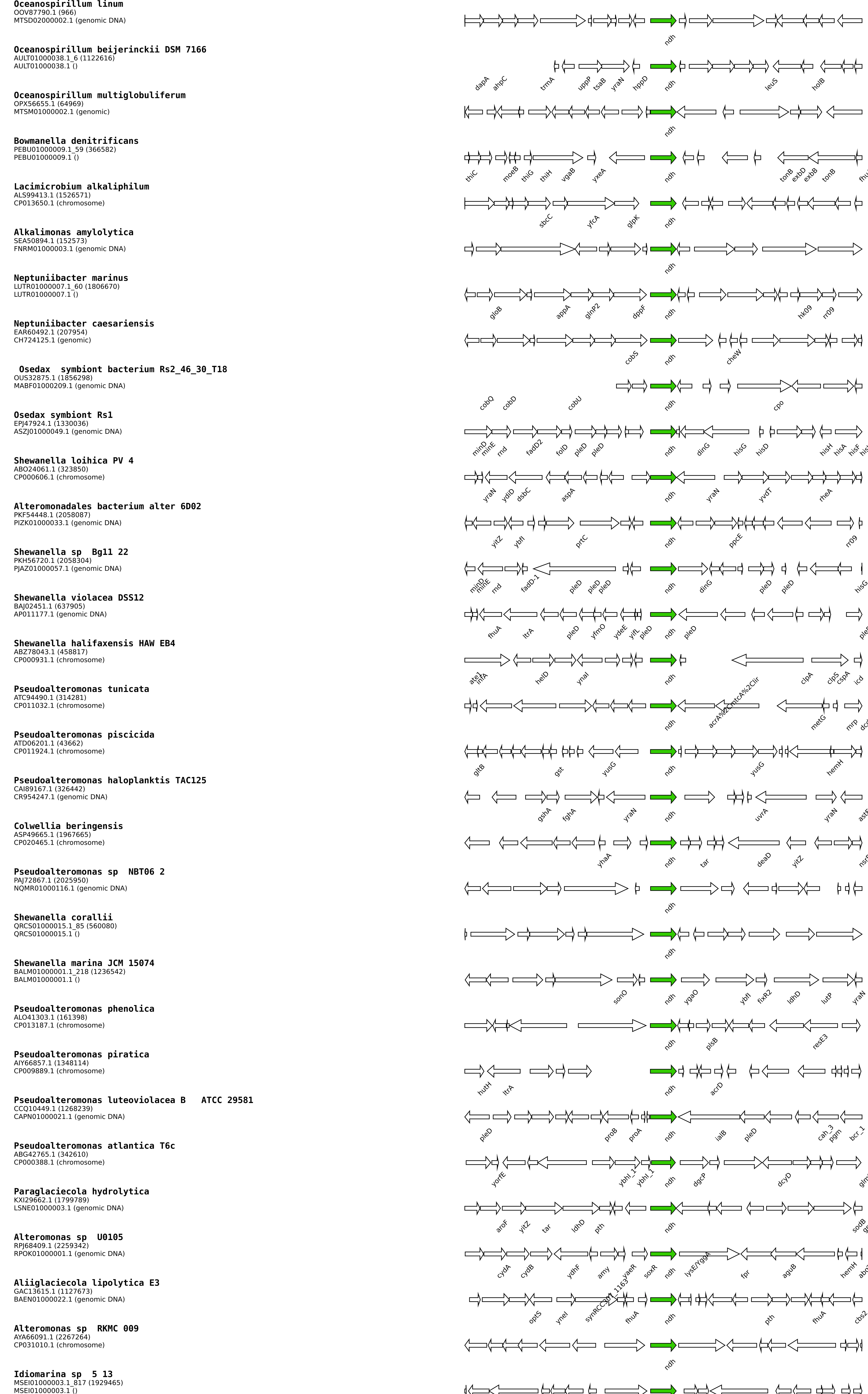
Oceanospirillum linum
OOV87790.1 (966)
MTSD02000002.1 (genomic DNA)
ndh
Oceanospirillum beijerinckii DSM 7166
AULT01000038.1_6 (1122616)
AULT01000038.1 ()
dapA ahpC trmA uppP tsaB yraN hppD ndh leuS holB
Oceanospirillum multiglobuliferum
OPX56655.1 (64969)
MTSM01000002.1 (genomic)
ndh
Bowmanella denitrificans
PEBU01000009.1_59 (366582)
PEBU01000009.1 ()
thiC moeB thiG thiH vgaB yxeA ndh tonB exbD exbB tonB
Lacimicrobium alkaliphilum
ALS99413.1 (1526571)
CP013650.1 (chromosome)
sbcC yfcA glpK ndh
Alkalimonas amylolytica
SEA50894.1 (152573)
FNRM01000003.1 (genomic DNA)
ndh
Neptuniibacter marinus
LUTR01000007.1_60 (1806670)
LUTR01000007.1 ()
gloB appA glnP2 dppF ndh hk09 rr09
Neptuniibacter caesariensis
EAR60492.1 (207954)
CH724125.1 (genomic)
cobS ndh cheW
Osedax symbiont bacterium Rs2_46_30_T18
OUS32875.1 (1856298)
MABF01000209.1 (genomic DNA)
cobQ cobD cobU ndh cpo
Osedax symbiont Rs1
EPJ47924.1 (1330036)
ASZJ01000049.1 (genomic DNA)
minD minE rnd fadD2 folD pleD pleD ndh dinG hisG hisD hisH hisA hisF
Shewanella loihica PV 4
ABO24061.1 (323850)
CP000606.1 (chromosome)
yraN ydiD dsbC aspA ndh yraN yvdT rheA
Alteromonadales bacterium alter 6D02
PKF54448.1 (2058087)
PIZK01000033.1 (genomic DNA)
yitZ ybfI prtC ndh ppcE rr09
Shewanella sp Bg11 22
PKH56720.1 (2058304)
PJAZ01000057.1 (genomic DNA)
minD minE rnd fadD-1 pleD pleD pleD ndh dinG pleD pleD hisG
Shewanella violacea DSS12
BAJ02451.1 (637905)
AP011177.1 (genomic DNA)
fhuA ltrA pleD yfmO ydeE yifL pleD ndh pleD
Shewanella halifaxensis HAW EB4
ABZ78043.1 (458817)
CP000931.1 (chromosome)
ate1 infA helD ynaI ndh clpA clpS cspA icd
Pseudoalteromonas tunicata
ATC94490.1 (314281)
CP011032.1 (chromosome)
ndh acrA%2CmtcA%2Clir metG mrp
Pseudoalteromonas piscicida
ATD06201.1 (43662)
CP011924.1 (chromosome)
gltB gst yusG ndh yusG hemH
Pseudoalteromonas haloplanktis TAC125
CAI89167.1 (326442)
CR954247.1 (genomic DNA)
gshA fghA yraN ndh uvrA yraN
Colwellia beringensis
ASP49665.1 (1967665)
CP020465.1 (chromosome)
yhaA ndh tar deaD yitZ
Pseudoalteromonas sp NBT06 2
PAJ72867.1 (2025950)
NQMR01000116.1 (genomic DNA)
ndh
Shewanella corallii
QRCS01000015.1_85 (560080)
QRCS01000015.1 ()
ndh
Shewanella marina JCM 15074
BALM01000001.1_218 (1236542)
BALM01000001.1 ()
sonO ndh ygaO ybfI fixR2 ldhD lutP yraN
Pseudoalteromonas phenolica
ALO41303.1 (161398)
CP013187.1 (chromosome)
ndh plsB resE3
Pseudoalteromonas piratica
AIY66857.1 (1348114)
CP009889.1 (chromosome)
hutH ltrA ndh acrD
Pseudoalteromonas luteoviolacea B ATCC 29581
CCQ10449.1 (1268239)
CAPN01000021.1 (genomic DNA)
pleD proB proA ndh ialB pleD cah_3 pgm bcr_1
Pseudoalteromonas atlantica T6c
ABG42765.1 (342610)
CP000388.1 (chromosome)
yorfE ybhI_1 ybhI_1 ndh dgcP dcyD
Paraglaciecola hydrolytica
KXI29662.1 (1799789)
LSNE01000003.1 (genomic DNA)
aroF yitZ tar ldhD pth ndh sodB
Alteromonas sp U0105
RPJ68409.1 (2259342)
RPOK01000001.1 (genomic DNA)
cydA cydB ydhF amy yaeR soxR ndh lysE/YggA fpr aguB hemH
Aliiglaciecola lipolytica E3
GAC13615.1 (1127673)
BAEN01000022.1 (genomic DNA)
optS yneI synRCC307_1163 fhuA ndh pth fhuA
Alteromonas sp RKMC 009
AYA66091.1 (2267264)
CP031010.1 (chromosome)
ndh
Idiomarina sp 5 13
MSEI01000003.1_817 (1929465)
MSEI01000003.1 ()

Salinimonas sp N102
AXR08347.1 (2303538)
CP031769.1 (chromosome)

Aestuariibacter aggregatus
SHF83168.1 (634436)
FQWD01000001.1 (genomic DNA)

Alteromonadaceae bacterium XY R5
LXWE01000005.1_5 (1737567)
LXWE01000005.1 ()

Catenovulum sp RQJ05
QMED01000057.1_5 (2234133)
QMED01000057.1 ()

Oceanobacter kriegii DSM 6294
AUGV01000003.1_387 (1122615)
AUGV01000003.1 ()

Arenicella xantha
RBP48457.1 (644221)
QNRT01000007.1 (genomic DNA)

Idiomarina indica
SDB49902.1 (1159017)
FMXN01000014.1 (genomic DNA)

Idiomarina maritima
PWW13659.1 (519453)
QGTT01000005.1 (genomic DNA)

Idiomarina sediminum DSM 21906
AUHL01000007.1_80 (1122124)
AUHL01000007.1 ()

Idiomarina sp UBA6937
DKAB01000151.1_1 (1946650)
DKAB01000151.1 ()

Idiomarina atlantica
KFZ28464.1 (1517416)
JPIN01000008.1 (genomic)

Idiomarina planktonica
RUO64026.1 (1323738)
PIQB01000002.1 (genomic)

Idiomarina salinarum
KFZ30423.1 (435908)
JPER01000005.1 (genomic)

Halomonas alkaliphila
AYF32330.1 (272774)
CP024811.1 (chromosome)

Halomonas subglaciescola
SHM41557.1 (29571)
LT670847.1 (genomic DNA)

Zymobacter palmae
BBG29442.1 (33074)
AP018933.1 (genomic DNA)

Halotalea alkalilenta
ANF58770.1 (376489)
CP015243.1 (chromosome)

Carnimonas nigrificans ATCC BAA 78
JAGO01000011.1_257 (1336245)
JAGO01000011.1 ()

Halomonadaceae bacterium R4HLG17
PVRV01000023.1_460 (2081032)
PVRV01000023.1 ()

Kushneria sinocarnis
RKR02650.1 (595502)
RBIN01000006.1 (genomic DNA)

Kushneria konosiri
ARS54316.1 (698828)
CP021323.1 (chromosome)

Salinisphaera halophila
QJOD01000001.1_628 (1304158)
QJOD01000001.1 ()

Salinisphaeraceae bacterium UBA4764
DHHU01000072.1_27 (1953075)
DHHU01000072.1 ()

Salinisphaera sp UBA1666
DCKU01000046.1_6 (1947454)
DCKU01000046.1 ()

Salinisphaera sp LB1
AWN15227.1 (2183911)
CP029488.1 (chromosome)

Salinisphaera sp Q1T1 3
RJS92851.1 (2321229)
QZWD01000009.1 (genomic DNA)

Halomonas aestuarii
APE29989.1 (1897729)
CP018139.1 (chromosome)

Halomonas heilongjiangensis
PMR71537.1 (1387883)
PNRE01000014.1 (genomic DNA)

Halomonas elongata DSM 2581
CBV43261.1 (768066)
FN869568.2 (genomic DNA)

Salinicola tamaricis
CP023559.1_4304 (1771309)
CP023559.1 ()

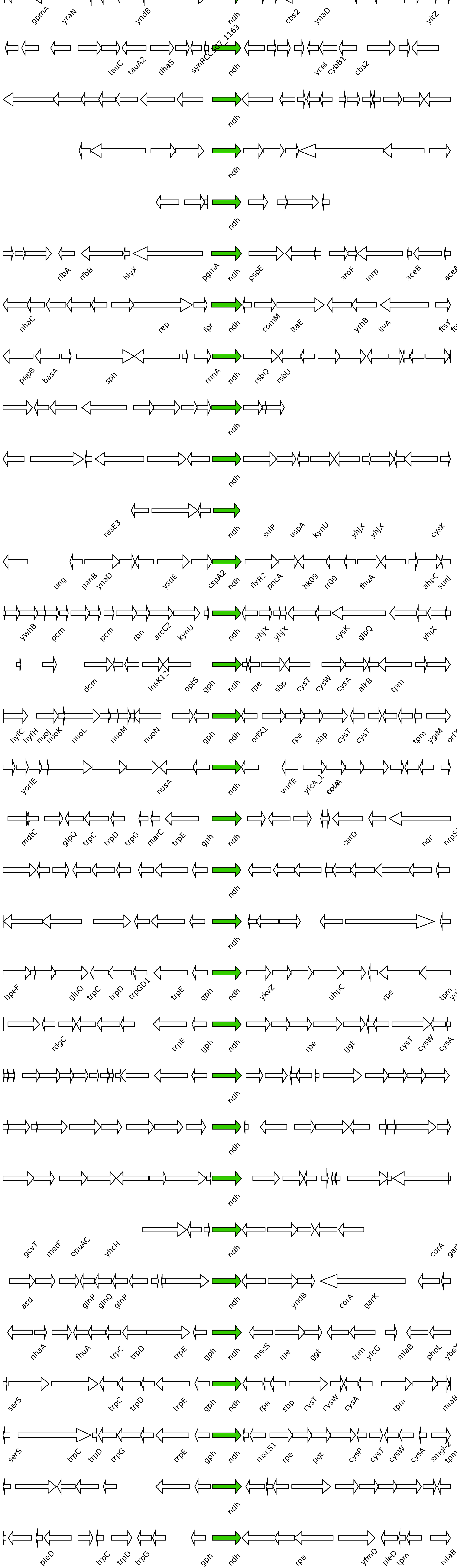

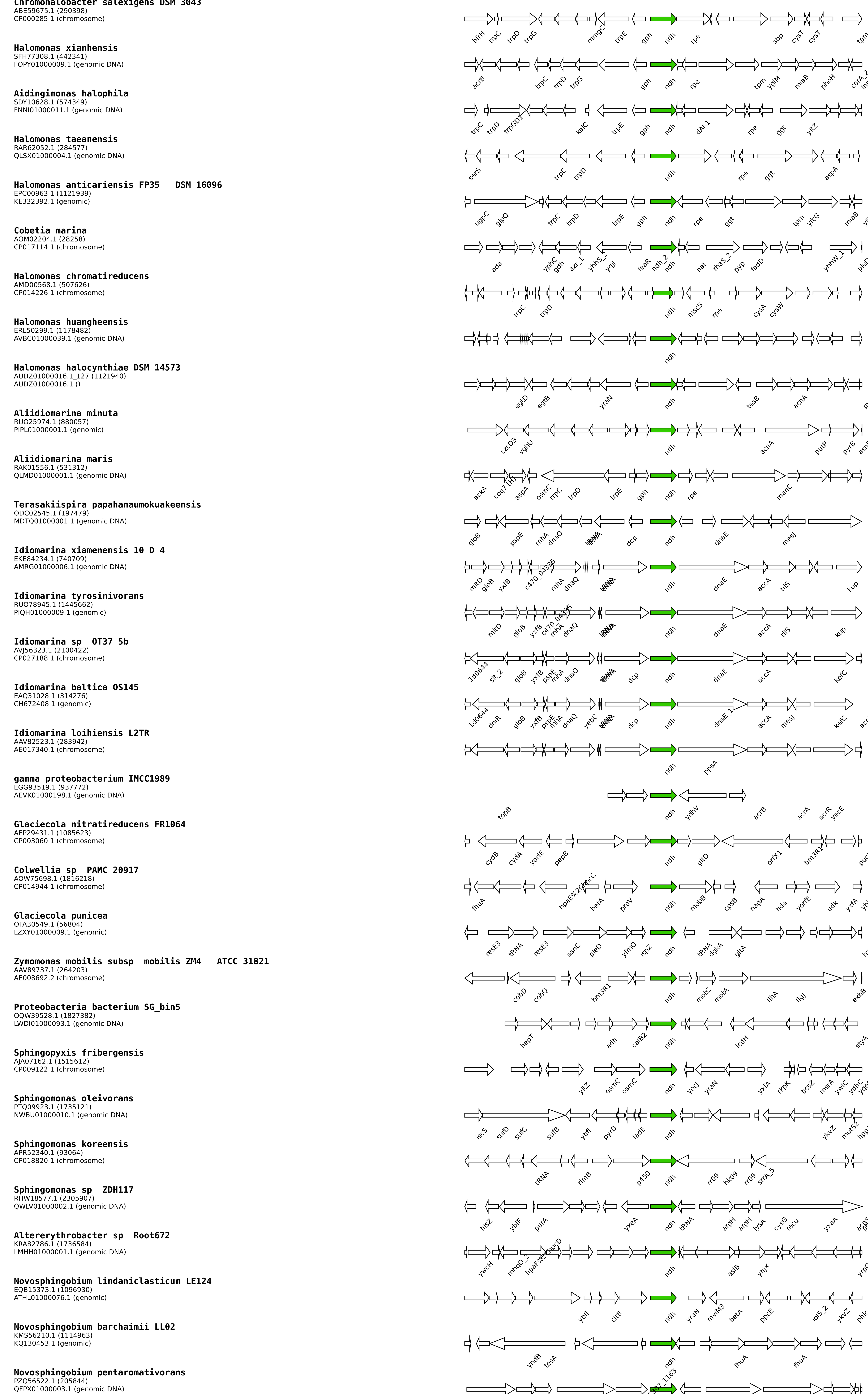
Chromohalobacter salexigens DSM 3043
ABE59675.1 (290398)
CP000285.1 (chromosome)
Halomonas xianhensis
SFH77308.1 (442341)
FOPY01000009.1 (genomic DNA)
Aidingimonas halophila
SDY10628.1 (574349)
FNNI01000011.1 (genomic DNA)
Halomonas taeanensis
RAR62052.1 (284577)
QLSX01000004.1 (genomic DNA)
Halomonas anticariensis FP35 DSM 16096
EPC00963.1 (1121939)
KE332392.1 (genomic)
Cobetia marina
AOM02204.1 (28258)
CP017114.1 (chromosome)
Halomonas chromatireducens
AMD00568.1 (507626)
CP014226.1 (chromosome)
Halomonas huangheensis
ERL50299.1 (1178482)
AVBC01000039.1 (genomic DNA)
Halomonas halocynthiae DSM 14573
AUDZ01000016.1_127 (1121940)
AUDZ01000016.1 ()
Aliidiomarina minuta
RUO25974.1 (880057)
PIPL01000001.1 (genomic)
Aliidiomarina maris
RAK01556.1 (531312)
QLMD01000001.1 (genomic DNA)
Terasakiispira papahanaumokuakeensis
ODC02545.1 (197479)
MDTQ01000001.1 (genomic DNA)
Idiomarina xiamenensis 10 D 4
EKE84234.1 (740709)
AMRG01000006.1 (genomic DNA)
Idiomarina tyrosinivorans
RUO78945.1 (1445662)
PIQH01000009.1 (genomic)
Idiomarina sp OT37 5b
AVJ56323.1 (2100422)
CP027188.1 (chromosome)
Idiomarina baltica OS145
EAQ31028.1 (314276)
CH672408.1 (genomic)
Idiomarina loihiensis L2TR
AAV82523.1 (283942)
AE017340.1 (chromosome)
gamma proteobacterium IMCC1989
EGG93519.1 (937772)
AEVK01000198.1 (genomic DNA)
Glaciecola nitratireducens FR1064
AEP29431.1 (1085623)
CP003060.1 (chromosome)
Colwellia sp PAMC 20917
AOW75698.1 (1816218)
CP014944.1 (chromosome)
Glaciecola punicea
OFA30549.1 (56804)
LZXY01000009.1 (genomic)
Zymomonas mobilis subsp mobilis ZM4 ATCC 31821
AAV89737.1 (264203)
AE008692.2 (chromosome)
Proteobacteria bacterium SG_bin5
OQW39528.1 (1827382)
LWDI01000093.1 (genomic DNA)
Sphingopyxis fribergensis
AJA07162.1 (1515612)
CP009122.1 (chromosome)
Sphingomonas oleivorans
PTQ09923.1 (1735121)
NWBU01000010.1 (genomic DNA)
Sphingomonas koreensis
APR52340.1 (93064)
CP018820.1 (chromosome)
Sphingomonas sp ZDH117
RHW18577.1 (2305907)
QWLV01000002.1 (genomic DNA)
Altererythrobacter sp Root672
KRA82786.1 (1736584)
LMHH01000001.1 (genomic DNA)
Novosphingobium lindaniclasticum LE124
EQB15373.1 (1096930)
ATHL01000076.1 (genomic)
Novosphingobium barchaimii LL02
KMS56210.1 (1114963)
KQ130453.1 (genomic)
Novosphingobium pentaromativorans
PZQ56522.1 (205844)
QFPX01000003.1 (genomic DNA)

Sphingobium sp YG1
BBD02372.1 (2082188)
AP018519.1 (genomic DNA)

Sphingobium sp LF 16
CP029335.1_1689 (2185111)
CP029335.1 ()

Proteobacteria bacterium ST_bin13
OQW76283.1 (1978761)
MSXQ01000005.1 (genomic)

alpha proteobacterium AAP81b
KPF74737.1 (1523432)
LJHX01000073.1 (genomic DNA)

Sphingomonas sp IBVSS1
OSZ69285.1 (1985171)
NFUS01000003.1 (genomic DNA)

Sphingomonadales bacterium 32 65 25
OYX77940.1 (1970594)
NCEU01000003.1 (genomic DNA)

Alphaproteobacteria bacterium Ga0077535
LNEK01000043.1_15 (1752192)
LNEK01000043.1 ()

Erythrobacter sp YT30
KWV93400.1 (1735012)
LMAF01000001.1 (genomic DNA)

Erythrobacter sp HL 111
SDR75557.1 (1798193)
LT629743.1 (genomic DNA)

Porphyrobacter cryptus DSM 12079
AUHC01000009.1_136 (1122970)
AUHC01000009.1 ()

Porphyrobacter HT 58 2
AUX69464.1 (2023229)
CP022600.1 (chromosome)

Erythrobacter sp JL475
KEO88403.1 (383381)
JMIV01000001.1 (genomic DNA)

Erythrobacter sp KY5
AWW74287.1 (2011159)
CP021912.1 (chromosome)

Erythrobacter longus
KEO90748.1 (1044)
JMIW01000002.1 (genomic DNA)

Hyphomonadaceae bacterium UKL13 1
AMS30040.1 (1690484)
CP012156.1 (chromosome)

Sphingomonas sp HMWF008
PTS76530.1 (2056845)
QAIS01000519.1 (genomic DNA)

Novosphingobium fuchskuhlense
KUR70812.1 (1117702)
KQ954245.1 (genomic)

Sphingomonas sp HMWF008
PTS76993.1 (2056845)
QAIS01000502.1 (genomic DNA)

Blastomonas sp RAC04
AOG02080.1 (1842535)
CP016460.1 (chromosome)

Acidovorax delafieldii
LSHP01000045.1_16 (47920)
LSHP01000045.1 ()

Rhodobacteraceae bacterium UBA996
DBBA01000406.1_18 (1952817)
DBBA01000406.1 ()

Maribius salinus
SHJ05846.1 (313368)
FQZA01000004.1 (genomic DNA)

Roseivivax lentus
SIT11483.1 (633194)
FTOQ01000017.1 (genomic DNA)

Roseivivax halotolerans
SFQ40050.1 (93684)
FOXV01000005.1 (genomic DNA)

Palleronia abyssalis
SPJ25114.1 (1501240)
ONZF01000007.1 (genomic DNA)

Rhodobacteraceae bacterium THAF1
VDC25585.1 (2483814)
UXAS01000024.1 (genomic DNA)

Maribius sp MOLA 401
JQEY01000004.1_112 (1530186)
JQEY01000004.1 ()

Hwanghaeicola aestuarii
PZX18521.1 (568105)
QKZL01000003.1 (genomic DNA)

Thalassococcus halodurans
SEG43062.1 (373675)
FNUZ01000004.1 (genomic DNA)

Roseivivax marinus
ETW14035.1 (1379903)
AQQW01000002.1 (genomic DNA)

Roseivivax halodurans JCM 10272

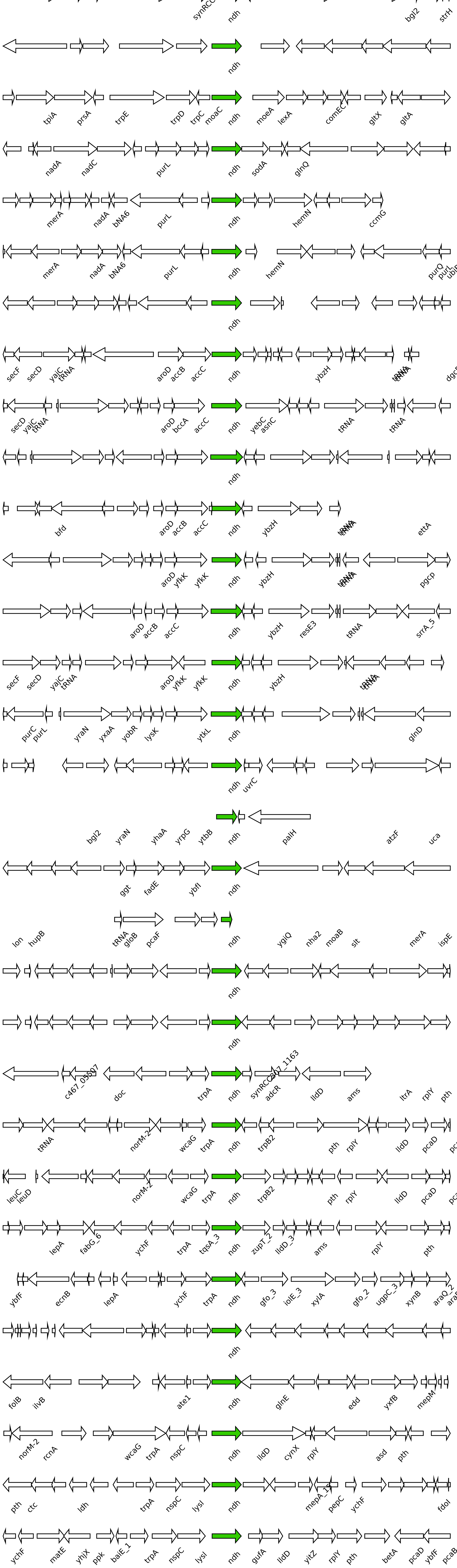

Roseivivax halodurans JCM 10272
ETX16211.1 (1449350)
JALZ01000002.1 (genomic)

Sinobacterium caligoides
ROS04861.1 (933926)
RKHR01000003.1 (genomic DNA)

Spongiibacter sp IMCC21906
AKH70243.1 (1620392)
CP011477.1 (chromosome)

Spongiibacter marinus DSM 19753
AULP01000003.1_228 (1122224)
AULP01000003.1 ()

Zhongshania aliphaticivorans
AMO67879.1 (1470434)
CP014544.1 (chromosome)

Gammaproteobacteria bacterium BRH_c0
KJS08369.1 (1629722)
LADM01000049.1 (genomic DNA)

Alcanivorax pacificus W11 5
AJD46856.1 (391936)
CP004387.1 (chromosome)

Alcanivorax indicus
QGMP01000001.1_278 (2202653)
QGMP01000001.1 ()

Alcanivorax borkumensis SK2
CAL15722.1 (393595)
AM286690.1 (genomic DNA)

Alcanivorax dieselolei B5
AFT68812.1 (930169)
CP003466.1 (chromosome)

Alcanivorax sp N3 2A
ASK36243.1 (2014542)
CP022307.1 (chromosome)

Gammaproteobacteria bacterium HGW Gammaproteobacteria 14
PKM22112.1 (2013796)
PGZG01000096.1 (genomic DNA)

Microbulbifer pacificus
PREV01000026.1_769 (407164)
PREV01000026.1 ()

Microbulbifer agarilyticus
AQQ68815.1 (260552)
CP019650.1 (chromosome)

Microbulbifer sp A4B17
AWF82831.1 (359370)
CP029064.1 (chromosome)

Microbulbifer thermotolerans
AMX03381.1 (252514)
CP014864.1 (chromosome)

Microbulbifer donghaiensis
SHF19797.1 (494016)
FQVA01000001.1 (genomic DNA)

Microbulbifer aggregans
AOS95848.1 (1769779)
CP014143.1 (chromosome)

Gayadomonas joobiniege G7
AMRX01000003.1_524 (1046714)
AMRX01000003.1 ()

Alteromonadales bacterium BS08
MRUG01000078.1_126 (1895005)
MRUG01000078.1 ()

Oceanobacter sp
PHR96013.1 (2030817)
NVWL01000059.1 (genomic DNA)

Agaribacterium haliotis
NKQJ01000005.1_163 (2013869)
NKQJ01000005.1 ()

Cellvibrionales bacterium UBA1483
DCRV01000029.1_11 (1950783)
DCRV01000029.1 ()

Ketobacter alkanivorans
AUM11081.1 (1917421)
CP022684.1 (chromosome)

Kistimonas sp bin40
MPMQ01000052.1_21 (1919249)
MPMQ01000052.1 ()

Aquisalimonas asiatica
SEP11093.1 (406100)
FOEG01000010.1 (genomic DNA)

Spiribacter sp E85
PWG61916.1 (2182432)
QFFI01000025.1 (genomic DNA)

Legionella longbeachae
ARB93861.1 (450)
CP020412.2 (chromosome)

Legionella hackeliae
CEK10538.1 (449)
LN681225.1 (genomic DNA)

Legionella tunisiensis
CALJ01000137.1_12 (1034944)
CALJ01000137.1 ()

Legionella feeleii
KTD03630.1 (453)
LNYB01000014.1 (genomic DNA)

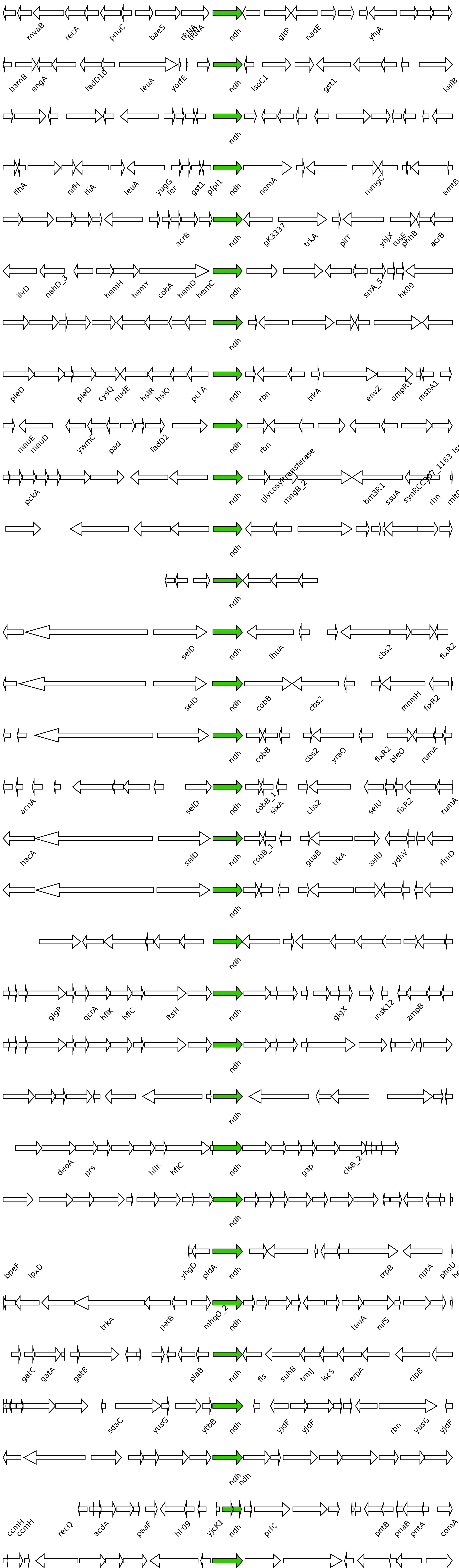

Legionella tunisiensis
CALJ01000137.1_13 (1034944)
CALJ01000137.1 ()

Algicola sagamiensis DSM 14643
KB894498.1_41 (1120963)
KB894498.1 ()

Shewanella spongiae
RJY07502.1 (342950)
ML064703.1 (genomic)

Shewanellaceae bacterium C51
RLV59620.1 (2338552)
ML014779.1 (genomic)

Shewanella mangrovi
KFZ39246.1 (1515746)
JPEO01000001.1 (genomic DNA)

Shewanella amazonensis SB2B
ABL99143.1 (326297)
CP000507.1 (chromosome)

Shewanella oneidensis MR 1
AAN56508.1 (211586)
AE014299.2 (chromosome)

Shewanella frigidimarina NCIMB 400
ABI72717.1 (318167)
CP000447.1 (chromosome)

Hydrogenovibrio sp UBA653
DBOF01000117.1_6 (1946607)
DBOF01000117.1 ()

Thiomicrospira sp S5
AZR82676.1 (1803865)
CP014470.1 (chromosome)

Hydrogenovibrio sp Milos T1
JQMT01000001.1_513 (90813)
JQMT01000001.1 ()

Hydrogenovibrio sp Milos T1
JQMT01000001.1_514 (90813)
JQMT01000001.1 ()

Hydrogenovibrio kuenenii DSM 12350
JAGP01000001.1_541 (1168067)
JAGP01000001.1 ()

Hydrogenovibrio marinus
KDN96779.1 (28885)
JMIU01000001.1 (genomic DNA)

Paraferrimonas sedimenticola
NRIR01000005.1_225 (375674)
NRIR01000005.1 ()

Paraferrimonas haliotis
NRRC01000003.1_270 (2013866)
NRRC01000003.1 ()

Shewanella marina JCM 15074
BALM01000039.1_25 (1236542)
BALM01000039.1 ()

Shewanella corallii
QRCS01000014.1_158 (560080)
QRCS01000014.1 ()

Shewanella japonica
ARD23180.1 (93973)
CP020472.1 (chromosome)

Shewanella loihica PV 4
ABO23031.1 (323850)
CP000606.1 (chromosome)

Shewanella halifaxensis HAW EB4
ABZ75760.1 (458817)
CP000931.1 (chromosome)

Ferrimonas balearica DSM 9799
ADN77130.1 (550540)
CP002209.1 (chromosome)

Ferrimonas sediminum
SDK11772.1 (718193)
FNEM01000019.1 (genomic DNA)

Ferrimonas futtsuensis DSM 18154
AUGL01000026.1_31 (1121878)
AUGL01000026.1 ()

Ferrimonas marina
SHI09814.1 (299255)
FQXG01000007.1 (genomic DNA)

Shewanella frigidimarina NCIMB 400
ABI73265.1 (318167)
CP000447.1 (chromosome)

Ferrimonas sediminum
SDI63694.1 (718193)
FNEM01000002.1 (genomic DNA)

Ferrimonas senticii DSM 18821
AUGM01000008.1_80 (1121880)
AUGM01000008.1 ()

Ferrimonas senticii DSM 18821
AUGM01000001.1_121 (1121880)
AUGM01000001.1 ()

Gammaproteobacteria bacterium 28 57 27
OYY74837.1 (1970513)
NCGK01000003.1 (genomic DNA)

Proteobacteria bacterium UBA2462

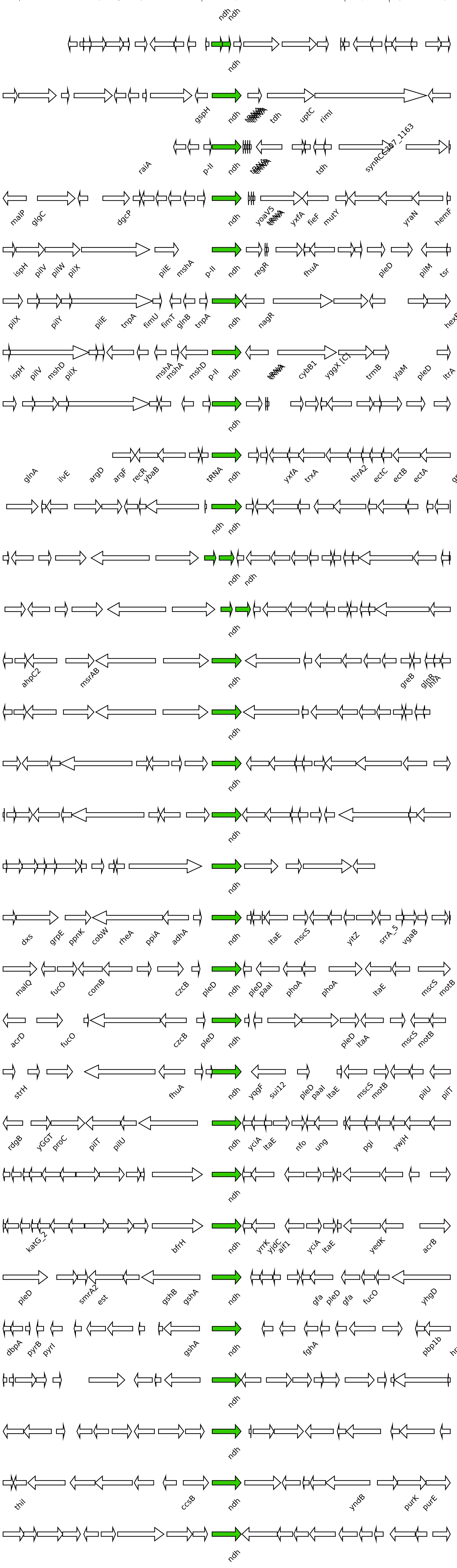

Proteobacteria bacterium UBA2462
DDPS01000018.1_7 (1948151)
DDPS01000018.1 ()

Simplicispira suum
AVO42374.1 (2109915)
CP027669.1 (chromosome)

Gammaproteobacteria bacterium UBA5338
DHQM01000065.1_10 (1948629)
DHQM01000065.1 ()

Candidatus Schmidhempelia bombi str Bimp
AWGA01000055.1_17 (1387197)
AWGA01000055.1 ()

Frischella perrara
AJA45797.1 (1267021)
CP009056.1 (chromosome)

Gilliamella apicola
AHN27330.1 (1196095)
CP007445.1 (chromosome)

Orbus hercynius
RKS87577.1 (593135)
RBWY01000001.1 (genomic DNA)

Coxiellaceae bacterium UBA6002
DJAM01000148.1_22 (1951632)
DJAM01000148.1 ()

Legionellales bacterium RIFCSPHIGHO2_12_FULL_42_9
OGV50088.1 (1798569)
MHBD01000040.1 (genomic DNA)

Hyphomicrobiaceae bacterium
PKEY01000113.1_38 (2015799)
PKEY01000113.1 ()

Legionella fallonii LLAP 10
CEG56160.1 (1212491)
LN614827.1 (genomic DNA)

Legionella quateirensis
KTD54759.1 (45072)
LNYR01000002.1 (genomic DNA)

Jeongeupia sp USM3
AOY01140.1 (1906741)
CP017668.1 (chromosome)

Crenobacter sp K1W11S 77
AXK40904.1 (2290923)
CP031337.1 (chromosome)

Chromobacterium violaceum ATCC 12472
AAQ58666.1 (243365)
AE016825.1 (chromosome)

Vogesella sp LIG4
SCK07217.1 (1192162)
LT607802.1 (genomic DNA)

Chitiniphilus shinanonensis DSM 23277
KB895334.1_203 (1121280)
KB895334.1 ()

Chitinibacter sp ZOR0017
JTLG01000042.1_29 (1339254)
JTLG01000042.1 ()

Chitinilyticum aquatile DSM 21506
AUMS01000004.1_207 (1121275)
AUMS01000004.1 ()

Andreprevotia lacus DSM 23236
SMC21786.1 (1121001)
FWXD01000006.1 (genomic DNA)

Deefgea rivuli DSM 18356
JHVM01000008.1_93 (1121375)
JHVM01000008.1 ()

Amantichitinum ursilacus
KPC52242.1 (857265)
LAQT01000010.1 (genomic DNA)

Azoarcus sp BH72
CAL94383.1 (62928)
AM406670.1 (genomic DNA)

Aromatoleum aromaticum EbN1
CAI10087.1 (76114)
CR555306.1 (genomic DNA)

Brevundimonas sp UBA5713
DIGV01000027.1_154 (1946130)
DIGV01000027.1 ()

Brevundimonas abyssalis TAR 001
GAD59038.1 (1391729)
BATC01000017.1 (genomic DNA)

Dokdonella koreensis DS 123
ANB19639.1 (1300342)
CP015249.1 (chromosome)

Thauera sp K11
ATE61227.1 (2005884)
CP023439.1 (chromosome)

Azoarcus sp BH72
CAL94451.1 (62928)
AM406670.1 (genomic DNA)

Acidovorax citrulli AAC00 1
ABM33694.1 (397945)
CP000512.1 (chromosome)

Comamonadaceae bacterium UBA2121
DCYB01000010.1_142 (1951599)
DCYB01000010.1 ()

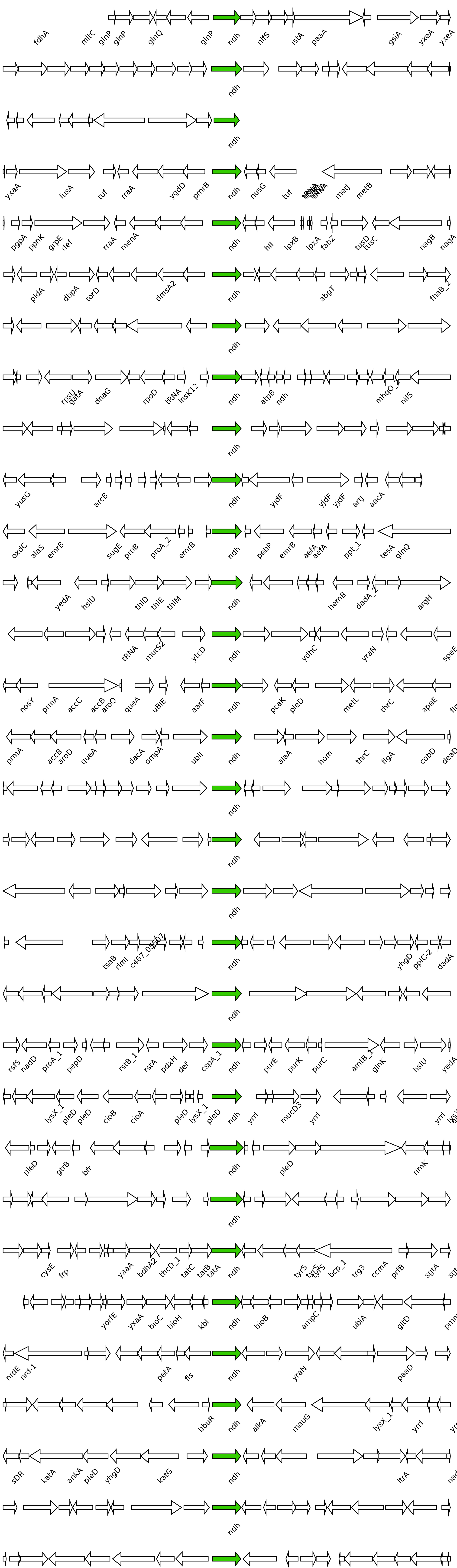

Comamonas serinivorans
ARU05756.1 (1082851)
CP021455.1 (chromosome)

Comamonadaceae bacterium UBA7039
DLAU01000167.1_13 (1951612)
DLAU01000167.1 ()

Comamonadaceae bacterium UBA3515
DFPM01000257.1_4 (1951605)
DFPM01000257.1 ()

Caulobacter mirabilis
ATQ44263.1 (69666)
CP024201.1 (chromosome)

Ideonella sp B508 1
BADL01000359.1_8 (137716)
BADL01000359.1 ()

Dokdonella immobilis
SFN29151.1 (578942)
FOVF01000012.1 (genomic DNA)

Derxia gummosa DSM 723
AXWS01000007.1_383 (1121388)
AXWS01000007.1 ()

Neisseria animaloris
OSI08771.1 (326522)
MTBN01000001.1 (genomic)

Vitreoscilla massiliensis
LN898219.1_371 (1689272)
LN898219.1 ()

Vitreoscilla sp C1
AUZ04022.1 (96942)
CP019644.1 (chromosome)

Snodgrassella sp CFCC 13594
LQXR01000001.1_301 (1775559)
LQXR01000001.1 ()

Brevundimonas diminuta ATCC 11568
EGF93913.1 (751586)
GL883086.1 (genomic)

Kingella negevensis
CCNJ01000058.1_46 (1522312)
CCNJ01000058.1 ()

Alysiella crassa DSM 2578
JQKH01000074.1_9 (1120980)
JQKH01000074.1 ()

Alysiella filiformis DSM 16848
SOD66049.1 (1120981)
OCNF01000003.1 (genomic DNA)

Dichelobacter nodosus VCS1703A
ABQ14076.1 (246195)
CP000513.1 (chromosome)

Brevundimonas sp NS26
QUOQ01000004.1_65 (2293980)
QUOQ01000004.1 ()

Candidimonas nitroreducens
OWT65478.1 (683354)
NJIH01000002.1 (genomic DNA)

Burkholderiales bacterium Beta_01
LUZQ01000036.1_11 (1674887)
LUZQ01000036.1 ()

Alcaligenes faecalis
KGP01250.1 (511)
JQCV01000005.1 (genomic DNA)

Candidimonas sp SYP B2681
RTZ47961.1 (2497686)
RYDV01000001.1 (genomic DNA)

Pusillimonas sp T7 7
AEC19588.1 (1007105)
CP002663.1 (chromosome)

Pusillimonas noertemannii
PVY61186.1 (305977)
QEKO01000004.1 (genomic DNA)

Eoetvoesia caeni
RBP42996.1 (645616)
QNRQ01000001.1 (genomic DNA)

Advenella mimigardefordensis DPN7
AHG65746.1 (1247726)
CP003915.1 (chromosome)

Kerstersia gyiorum
KKO70454.1 (206506)
LBNE01000015.1 (genomic DNA)

Orrella dioscoreae
SOE51733.1 (1851544)
LT907988.1 (genomic DNA)

Bordetella genomosp 9
ARP85244.1 (1416803)
CP021109.1 (chromosome)

Bordetella sp H567
AOB29813.1 (1697043)
CP012334.1 (chromosome)

Bordetella sp FB 8
KB907784.1_1840 (1159870)
KB907784.1 ()

Bordetella bronchiseptica RB50

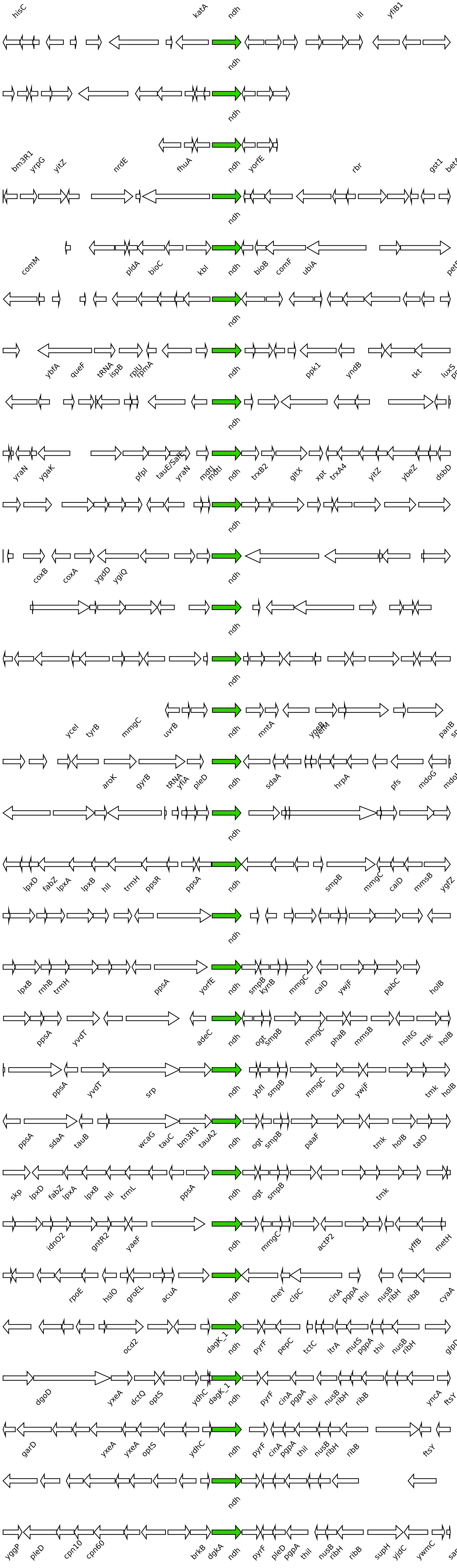

CAE31457.1 (257310)
BX470250.1 (genomic DNA)

Bordetella genomosp 9
ARP85763.1 (1416803)
CP021109.1 (chromosome)

Bordetella sp H567
AOB33845.1 (1697043)
CP012334.1 (chromosome)

Hydrogenophaga sp PBC
AOS81519.1 (795665)
CP017311.1 (chromosome)

Hydrogenophaga sp RAC07
AOF84966.1 (1842537)
CP016449.1 (chromosome)

Noviherbaspirillum sp K1S02 23
RJG01064.1 (2320865)
QYUQ01000002.1 (genomic DNA)

Massilia psychrophila
PIL38778.1 (1603353)
PDOB01000029.1 (genomic DNA)

Massilia sp WF1
KLU38220.1 (1406431)
LELH02000003.1 (genomic DNA)

Massilia oculi
AWL03500.1 (945844)
CP029343.1 (chromosome)

Duganella sp Leaf126
KQQ36205.1 (1736266)
LMNW01000023.1 (genomic DNA)

Collimonas fungivorans Ter331
AEK63245.1 (1005048)
CP002745.1 (chromosome)

Limnobacter sp CACIAM 66H1
KYP12014.1 (1813033)
LUJK01000009.1 (genomic DNA)

Thauera butanivorans NBRC 103042
BCUG01000003.1_133 (1219356)
BCUG01000003.1 ()

Gallaecimonas sp HK 28
CP031416.1_2827 (2291597)
CP031416.1 ()

Paraburkholderia sp DCR13
CP029641.1_171 (2211211)
CP029641.1 ()

Burkholderiales bacterium RIFCSPLOWO2_02_FULL_57_36
OGB25897.1 (1797562)
MESE01000036.1 (genomic DNA)

Noviherbaspirillum autotrophicum
KIF82918.1 (709839)
JWJG01000028.1 (genomic DNA)

Yangia pacifica
AWI83197.1 (311180)
CP022189.1 (chromosome)

Sulfitobacter sp HI0054
KZY52345.1 (1822238)
LWFD01000118.1 (genomic DNA)

Moritella viscosa
CED59659.1 (80854)
LN554852.1 (genomic DNA)

Vibrio sp N418
EGU33845.1 (701176)
AFWD01000051.1 (genomic DNA)

Vibrio harveyi 1DA3
EEZ86562.1 (673519)
ACZC01000045.1 (genomic)

Photobacterium aquae
KLV05555.1 (1195763)
LDOT01000013.1 (genomic DNA)

Shewanella marina JCM 15074
BALM01000033.1_39 (1236542)
BALM01000033.1 ()

Shewanella halifaxensis HAW EB4
ABZ78578.1 (458817)
CP000931.1 (chromosome)

Photobacterium damselae subsp piscicida
OLQ83204.1 (38294)
MCFX02000001.1 (genomic DNA)

Aliivibrio fischeri ES114
AAW86859.1 (312309)
CP000020.2 (chromosome)

Photobacterium damselae subsp piscicida
OLQ83205.1 (38294)
MCFX02000001.1 (genomic DNA)

Pseudomonas pohangensis
SDU06573.1 (364197)
LT629785.1 (genomic DNA)

Gammaproteobacteria bacterium UBA6940
DJZY01000020.1_12 (1948640)
DJZY01000020.1 ()

Roseovarius albus
SLN69859.1 (1247867)
FWFX01000016.1 (genomic DNA)

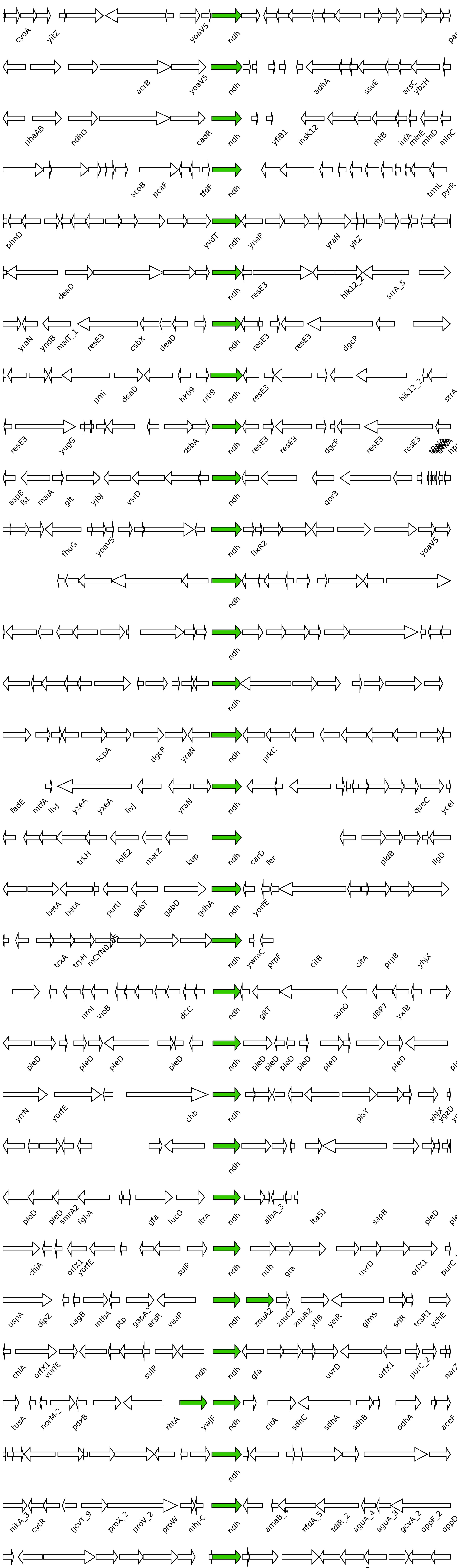

Dyella sp 333MFSha
SDF56547.1 (1798240)
FNBR01000001.1 (genomic DNA)

Rhodobacter sp 140A
RBP77752.1 (2183910)
QNSD01000054.1 (genomic DNA)

Candidatus Peregrinibacteria bacterium CG_4_9_14_0_2_um_filter_53_11
PJC36511.1 (1974780)
PFRX01000047.1 (genomic DNA)

Candidatus Brocadiaceae bacterium
PUMJ01000061.1_21 (2094028)
PUMJ01000061.1 ()

Planctomycetes bacterium SM23_32
KPK58690.1 (1704029)
LJUE01000194.1 (genomic DNA)

Chloroflexi bacterium UBA5177
DHWR01000117.1_5 (1947833)
DHWR01000117.1 ()

Candidatus Kerfeldbacteria bacterium CG_4_9_14_3_um_filter_45_8
PJA92965.1 (2014243)
PFVE01000033.1 (genomic DNA)

Nanohaloarchaea archaeon PL Br10_U2g16
LKMV01000043.1_1 (1727665)
LKMV01000043.1 ()

Natronorubrum thiooxidans
SIS21189.1 (308853)
FTNR01000033.1 (genomic DNA)

Natronoarchaeum philippinense
SNZ03633.1 (558529)
OBEJ01000001.1 (genomic DNA)

Methanonatronarchaeum thermophilum
OUJ18654.1 (1927129)
MRZU01000003.1 (genomic)

Methanonatronarchaeia archaeon
PWHS01000009.1_2 (2099682)
PWHS01000009.1 ()

Candidatus Haloredivivus sp G17
EHK02366.1 (1072681)
AGNT01000064.1 (genomic DNA)

Nanohaloarchaea archaeon B1 Br10_U2g21
LKMP01000001.1_90 (1729455)
LKMP01000001.1 ()

Nanohaloarchaea archaeon PL Br10_U2g27
LKMX01000048.1_6 (1727666)
LKMX01000048.1 ()

Nanohaloarchaea archaeon PL Br10_U2g19
LKMW01000001.1_87 (1727664)
LKMW01000001.1 ()

Nanohaloarchaea archaeon SW_7_43_1
PSG99373.1 (1919221)
PXPE01000001.1 (genomic DNA)

Nanohaloarchaea archaeon SW_4_43_9
PSH00146.1 (1919220)
PXPD01000012.1 (genomic DNA)

Haloquadratum walsbyi J07HQW1
ERG91948.1 (1238424)
KE356560.1 (genomic)

Candidatus Nanosalinarum sp J07AB56
EGQ39839.1 (889962)
GL982569.1 (genomic)

Haloquadratum sp J07HQX50
ERG97793.1 (1238426)
KE356563.1 (genomic)

Syntrophobacter sp SbD1
SPF34117.1 (2043167)
OMOE01000024.1 (genomic DNA)

Verrucomicrobia bacterium UBA5540
DINM01000081.1_16 (1948267)
DINM01000081.1 ()

Verrucomicrobia bacterium UBA5207
DHVN01000084.1_4 (1948266)
DHVN01000084.1 ()

Clostridiales bacterium oral taxon 876 str F0540
ERI90934.1 (1321778)
KE993501.1 (genomic)

Calditerrivibrio nitroreducens DSM 19672
ADR18466.1 (768670)
CP002347.1 (chromosome)

Denitrovibrio acetiphilus DSM 12809
ADD69507.1 (522772)
CP001968.1 (chromosome)

Nanohaloarchaea archaeon B1 Br10_U2g29
LKMQ01000017.1_8 (1729456)
LKMQ01000017.1 ()

Chlamydiia bacterium
PMZB01000275.1_10 (2052156)
PMZB01000275.1 ()

Candidatus Syntrophoarchaeum caldarius
OFV68592.1 (1838285)
LYOS01000001.1 (genomic DNA)

Methanomicrobiales archaeon

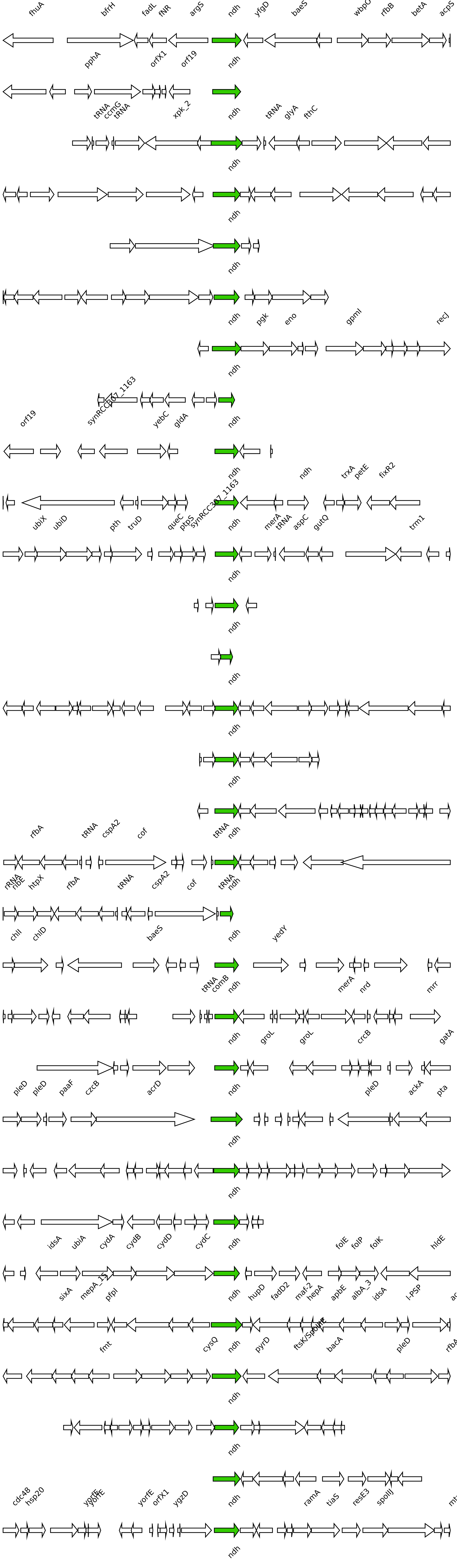

QEXZ01000088.1_2 (2184052)
QEXZ01000088.1 ()

ANME 1 cluster archaeon ex4572_4
OYT67157.1 (2011131)
NJEM01000017.1 (genomic DNA)

Candidatus Syntrophoarchaeum butanivorans
OFV65926.1 (1839936)
LYOR01000005.1 (genomic DNA)

Archaeoglobus profundus DSM 5631
ADB57179.1 (572546)
CP001857.1 (chromosome)

Methanomicrobia archaeon JdFR 19
MTMG01000018.1_78 (1935140)
MTMG01000018.1 ()

Methermicoccus shengliensis DSM 18856
JONQ01000008.1_332 (1122233)
JONQ01000008.1 ()

Methanosarcinales archaeon Methan_01
LUYE01000015.1_52 (1674798)
LUYE01000015.1 ()

Methanosaeta harundinacea 6Ac
AET63775.1 (1110509)
CP003117.1 (chromosome)

Methanosaeta sp UBA114
DAXJ01000089.1_13 (1915555)
DAXJ01000089.1 ()

Methanothrix sp
PMIM01000099.1_1 (90426)
PMIM01000099.1 ()

Methanosarcinales archaeon Methan_03
LUYG01000576.1_2 (1674800)
LUYG01000576.1 ()

Methanothrix soehngenii GP6
AEB68148.1 (990316)
CP002565.1 (chromosome)

Methanosaeta sp NSM2
OYV13931.1 (1917473)
NOLD01000123.1 (genomic DNA)

Methanosaeta sp UBA80
DAWB01000087.1_2 (1915564)
DAWB01000087.1 ()

Methanothrix thermoacetophila PT
ABK15315.1 (349307)
CP000477.1 (chromosome)

Archaeoglobus sp
RUM34262.1 (1872626)
QNXS01000035.1 (genomic DNA)

Arcobacter sp LA11
BDIR01000001.1_50 (1898176)
BDIR01000001.1 ()

Arcobacter sp 31_11_sub10_T18
OUR72848.1 (1856277)
MAAG01000069.1 (genomic DNA)

Arcobacter nitrofigilis DSM 7299
ADG92238.1 (572480)
CP001999.1 (chromosome)

Sulfurospirillum sp UBA6810
DKEY01000054.1_14 (1947600)
DKEY01000054.1 ()

Parcubacteria group bacterium GW2011_GWA2_47_12
KKU57133.1 (1618841)
LCNP01000044.1 (genomic DNA)

Chloroflexus aurantiacus J 10 fl
ABY33395.1 (324602)
CP000909.1 (chromosome)

Candidatus Chloroploca asiatica
PDW01315.1 (1506545)
LYXE01000009.1 (genomic DNA)

Candidatus Viridilinea sp Chok 6
RRR73335.1 (2491704)
RSAS01000345.1 (genomic DNA)

Candidatus Viridilinea mediisalina
PDW04106.1 (2024553)
NQWI01000015.1 (genomic DNA)

Oscillochloris trichoides DG 6
EFO81042.1 (765420)
GL501403.1 (genomic)

Gloeobacter violaceus PCC 7421
BAC88466.1 (251221)
BA000045.2 (genomic DNA)

Acidimicrobiia bacterium UBA4461
DGOR01000174.1_14 (1948318)
DGOR01000174.1 ()

Candidatus Abyssubacteria bacterium SURF_17
RJP69602.1 (2093361)
QZKI01000080.1 (genomic DNA)

Candidatus Nitrosocosmicus exaquare
CP017922.1_434 (1826872)
CP017922.1 ()

Candidatus Nitrosocosmicus oleophilus
ALI34719.1 (1353260)
CP012850.1 (chromosome)

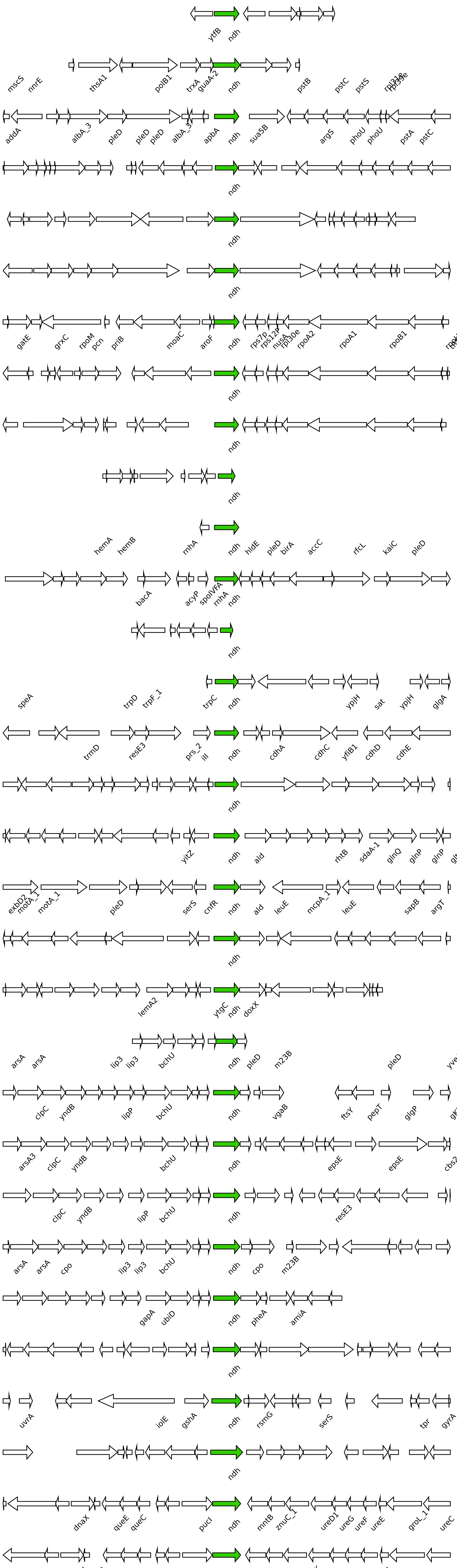

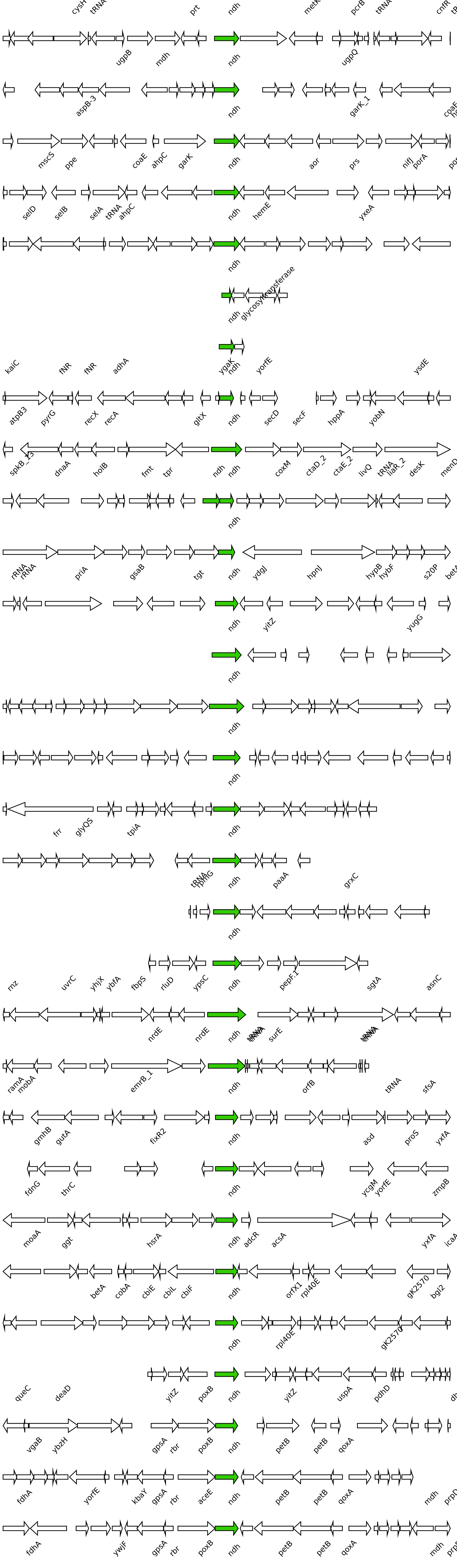

Pyrolobus fumarii 1A
AEM38021.1 (694429)
CP002838.1 (chromosome)

Hyperthermus butylicus DSM 5456
ABM80189.1 (415426)
CP000493.1 (chromosome)

Pyrodictium delaneyi
ALL00377.1 (1273541)
CP013011.1 (chromosome)

Pyrodictium occultum
KSW11543.1 (2309)
LNTB01000001.1 (genomic DNA)

Marispirochaeta aestuarii
ORC37011.1 (1963862)
MWQY01000004.1 (genomic DNA)

Opitutae bacterium UBA1513
DCQR01000069.1_1 (1948708)
DCQR01000069.1 ()

Cyanobacteria bacterium J083
RMF22282.1 (2420336)
RFIT01000708.1 (genomic DNA)

Alsobacter sp SH9
PSC05952.1 (2109933)
PVZS01000005.1 (genomic DNA)

Thermobaculum terrenum ATCC BAA 798
ACZ41000.1 (525904)
CP001825.1 (chromosome)

Leptolyngbya sp O 77
BAU44991.1 (1080068)
AP017367.1 (genomic DNA)

Bacteroides propionicifaciens DSM 19291   JCM 14649
AQWS01000011.1_22 (1121099)
AQWS01000011.1 ()

Candidatus Atelocyanobacterium thalassa isolate ALOHA
ADB95303.1 (1453429)
CP001842.1 (chromosome)

Thaumarchaeota archaeon 13_1_40CM_4_48_7
OLC22436.1 (1805396)
MNFA01000068.1 (genomic DNA)

Gammaproteobacteria bacterium Gsub
JYIN01000010.1_58 (1615892)
JYIN01000010.1 ()

Oceanobacillus sojae
LT727810.1_676 (582851)
LT727810.1 ()

Candidatus Falkowbacteria bacterium UBA2372
DDTE01000008.1_32 (1953278)
DDTE01000008.1 ()

Candidatus Uhrbacteria bacterium GW2011_GWF2_39_13
KKR04862.1 (1618995)
LBWG01000003.1 (genomic DNA)

Candidatus Uhrbacteria bacterium CG10_big_fil_rev_8_21_14_0_10_48_16
PJE76773.1 (1975038)
PFEU01000013.1 (genomic DNA)

Gemmatimonas sp
PKET01000029.1_6 (1962908)
PKET01000029.1 ()

Oenococcus kitaharae DSM 17330
EHN59459.1 (1045004)
CM001398.1 (chromosome)

Chlamydiae bacterium RIFCSPHIGHO2_12_FULL_49_11
OGN61347.1 (1797603)
MGLW01000066.1 (genomic DNA)

Ferroplasma acidarmanus fer1
AGO61780.1 (333146)
CP004145.1 (chromosome)

Acidiplasma sp MBA 1
KJE49886.1 (1293648)
JYHS01000001.1 (genomic DNA)

Picrophilus torridus DSM 9790
AAT43633.1 (263820)
AE017261.1 (chromosome)

Picrophilus torridus DSM 9790
AAT43762.1 (263820)
AE017261.1 (chromosome)

Ferroplasma acidarmanus fer1
AGO61301.1 (333146)
CP004145.1 (chromosome)

Ferroplasma sp Type II
EQB74165.1 (261388)
KI542692.1 (genomic)

Sulfodiicoccus acidiphilus
BBD72227.1 (1670455)
AP018553.1 (genomic DNA)

Acidianus brierleyi
AWR93239.1 (41673)
CP029289.1 (chromosome)

Acidianus manzaensis
ARM74789.1 (282676)
CP020477.1 (chromosome)

Acidianus sulfidivorans JP7

AWR96309.1 (619593)
CP029288.1 (chromosome)

Saccharolobus solfataricus P2
AAK43064.1 (273057)
AE006641.1 (chromosome)

Sulfolobus sp UBA167
DAHF01000081.1_3 (1915604)
DAHF01000081.1 ()

Sulfolobus sp JCM 16833
BBCL01000012.1_16 (1294262)
BBCL01000012.1 ()

Candidatus Acidianus copahuensis
EZQ07065.1 (1160895)
JFZT01000039.1 (genomic DNA)

Sulfolobus acidocaldarius DSM 639
AAY81237.1 (330779)
CP000077.1 (chromosome)

Sulfolobus acidocaldarius SUSAZ
AHC52041.1 (1435377)
CP006977.1 (chromosome)

Sulfolobus sp A20
AOL16408.1 (1891280)
CP017006.1 (chromosome)

Saccharolobus solfataricus P2
AAK41279.1 (273057)
AE006641.1 (chromosome)

Sulfolobus islandicus L S 2 15
ACP35313.1 (429572)
CP001399.1 (chromosome)

Sulfodiicoccus acidiphilus
BBD73428.1 (1670455)
AP018553.1 (genomic DNA)

Candidatus Aramenus sulfurataquae
EWG07964.1 (1326980)
ASRH01000002.1 (genomic DNA)

Acidianus brierleyi
AWR94120.1 (41673)
CP029289.1 (chromosome)

Metallosphaera yellowstonensis MK1
EHP68189.1 (671065)
JH597770.1 (genomic)

Metallosphaera cuprina Ar 4
AEB94833.1 (1006006)
CP002656.1 (chromosome)

Metallosphaera sedula DSM 5348
ABP95666.1 (399549)
CP000682.1 (chromosome)

Metallosphaera hakonensis JCM 8857 DSM 7519
BBBA01000006.1_54 (1293036)
BBBA01000006.1 ()

Sulfolobus acidocaldarius DSM 639
AAY81008.1 (330779)
CP000077.1 (chromosome)

Sulfolobus acidocaldarius SUSAZ
AHC51899.1 (1435377)
CP006977.1 (chromosome)

Sulfolobus sp A20
AOL16601.1 (1891280)
CP017006.1 (chromosome)

Acidianus hospitalis W1
AEE94424.1 (933801)
CP002535.1 (chromosome)

Acidianus sulfidivorans JP7
AWR98335.1 (619593)
CP029288.1 (chromosome)

Acidianus manzaensis
ARM76714.1 (282676)
CP020477.1 (chromosome)

Candidatus Acidianus copahuensis
EZQ01895.1 (1160895)
JFZT01000059.1 (genomic DNA)

Sulfolobus metallicus DSM 6482 JCM 9184
BBBY01000003.1_29 (523847)
BBBY01000003.1 ()

Sulfolobus sp UBA167
DAHF01000113.1_1 (1915604)
DAHF01000113.1 ()

Sulfolobus sp JCM 16833
BBCL01000004.1_59 (1294262)
BBCL01000004.1 ()

Sulfurisphaera tokodaii str 7
BAB67684.1 (273063)
BA000023.2 (genomic DNA)

Sulfolobus islandicus L S 2 15
ACP36361.1 (429572)
CP001399.1 (chromosome)

Saccharolobus solfataricus P2
AAK43249.1 (273057)
AE006641.1 (chromosome)

Desulfobacterales bacterium SG8_35
KPK00203.1 (1703400)
LJTJ01000014.1 (genomic DNA)

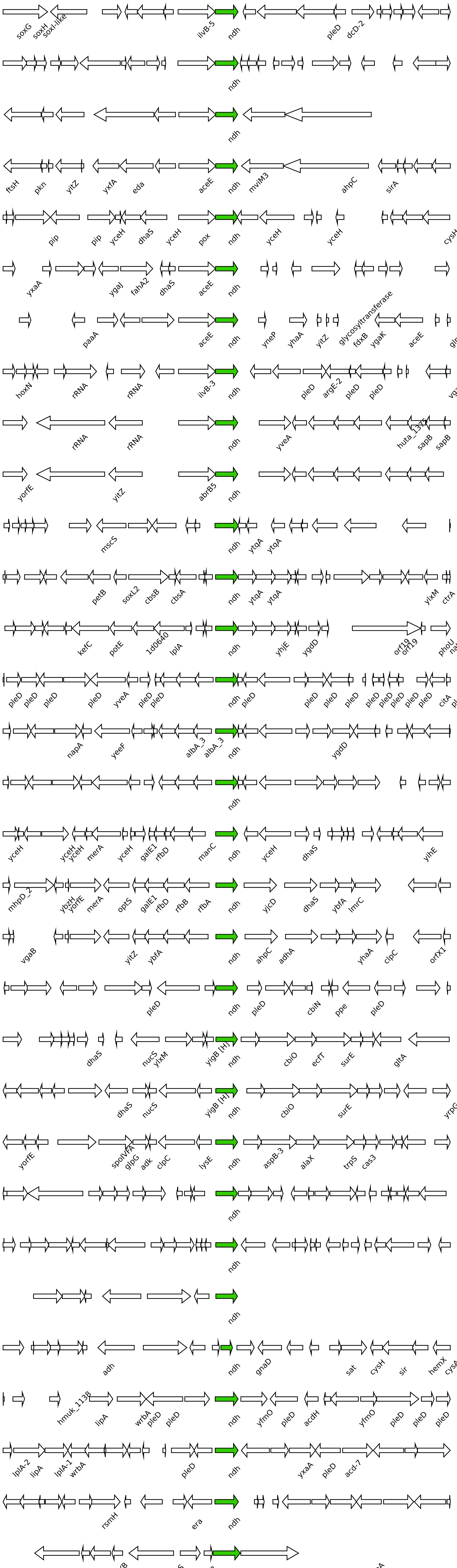

Bacillus solimangrovi
OEH94529.1 (1305675)
MJEH01000001.1 (genomic DNA)

Bacillus acanthi
QBBX01000028.1_8 (2171554)
QBBX01000028.1 ()

Jeotgalibacillus campisalis
KIL52823.1 (220754)
JXRR01000001.1 (genomic DNA)

Bacillus kribbensis DSM 17871
AUMQ01000016.1_307 (1121092)
AUMQ01000016.1 ()

Planifilum fimeticola
PRX39685.1 (201975)
PVNE01000021.1 (genomic DNA)

Paucisalibacillus globulus
LT855399.1_188 (351095)
LT855399.1 ()

Thermoactinomyces sp Gus2 1
KFZ41502.1 (1535750)
JPZM01000001.1 (genomic DNA)

Fictibacillus aquaticus
OYD57760.1 (2021314)
NOII01000003.1 (genomic DNA)

Bacillales bacterium UBA6768
DKGO01000148.1_58 (1950334)
DKGO01000148.1 ()

Aneurinibacillus soli
BAU28870.1 (1500254)
AP017312.1 (genomic DNA)

Paenibacillus swuensis
ANE47739.1 (1178515)
CP011388.1 (chromosome)

Gracilibacillus dipsosauri
PWU70427.1 (178340)
QGTD01000001.1 (genomic DNA)

Salibacterium halotolerans
SFP65414.1 (1884432)
FOXD01000008.1 (genomic DNA)

Bacillus gobiensis
ALC84375.1 (1441095)
CP012600.1 (chromosome)

Virgibacillus salinus
SDQ92435.1 (553311)
FNKD01000003.1 (genomic DNA)

Paenibacillus curdlanolyticus YK9
EFM12553.1 (717606)
AEDD01000001.1 (genomic DNA)

Paenibacillus assamensis DSM 18201
AULU01000011.1_178 (1122916)
AULU01000011.1 ()

Bacillus fastidiosus NBRC 101226
BCVG01000009.1_24 (1348628)
BCVG01000009.1 ()

Calditerricola satsumensis JCM 14719
BBCF01000370.1_1 (1294024)
BBCF01000370.1 ()

Peptococcus niger
SDD55248.1 (2741)
FNAF01000004.1 (genomic DNA)

Candidatus Riflebacteria bacterium HGW Riflebacteria 2
PKL47030.1 (2013830)
PGYA01000040.1 (genomic DNA)

Candidatus Omnitrophica bacterium CG1_02_44_16
OIO35218.1 (1805287)
MNVR01000035.1 (genomic DNA)

Candidatus Omnitrophica bacterium UBA6210
DISM01000004.1_281 (1953267)
DISM01000004.1 ()

Candidatus Eisenbacteria bacterium RBG_19FT_COMBO_70_11
OGF23807.1 (1817857)
MFFR01000093.1 (genomic DNA)

Candidatus Uhrbacteria bacterium RIFCSPLOWO2_02_FULL_53_10
OGL89211.1 (1802411)
MGET01000048.1 (genomic DNA)

Dissulfuribacter thermophilus
OCC16022.1 (1156395)
MAGO01000002.1 (genomic DNA)

archaeon GW2011_AR3
KHO45085.1 (1579365)
JWKO01000013.1 (genomic DNA)

Azotobacter chroococcum NCIMB 8003
AJE21600.1 (1328314)
CP010415.1 (chromosome)

Chitinivibrio alkaliphilus ACht1
ERP31951.1 (1313304)
ASJR01000008.1 (genomic DNA)

Agitococcus lubricus
PTQ88157.1 (1077255)
QAON01000014.1 (genomic DNA)

Parcubacteria group bacterium GW2011_GWA2_43_13

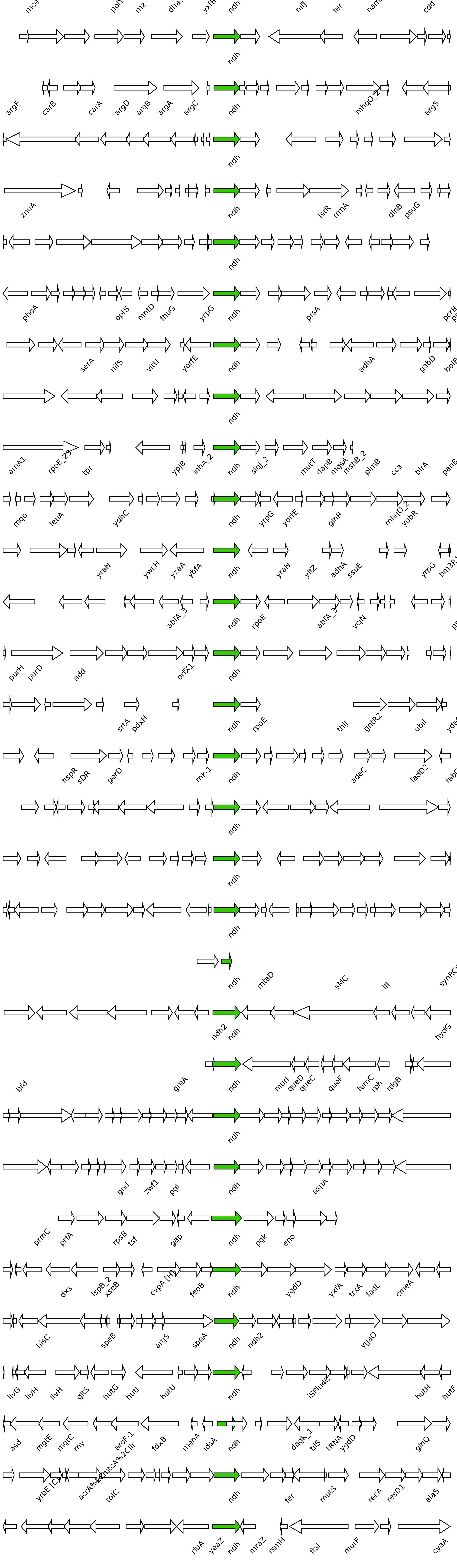

KKS93638.1 (1618826)
LCFM01000008.1 (genomic DNA)

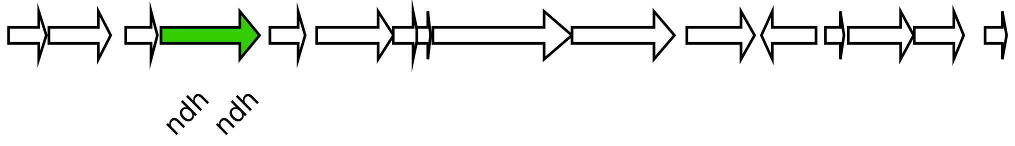

Candidatus Doudnabacteria bacterium
PLYC01000022.1_18 (2052150)
PLYC01000022.1 ()

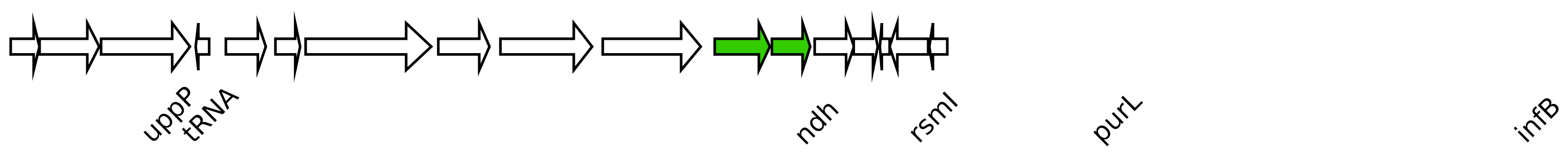

Candidatus Abawacabacteria bacterium RBG_16_42_10
OGC81655.1 (1817814)
MEWR01000023.1 (genomic DNA)

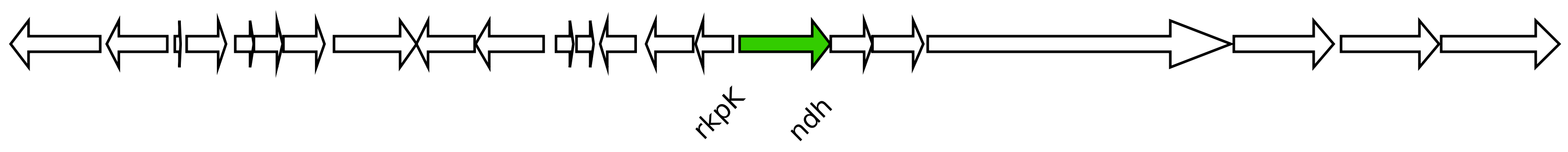

Acidocella sp 20 61 6
OYV35655.1 (1970303)
NCAK01000399.1 (genomic DNA)

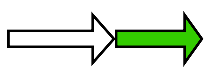

Supplement: Supplementary file 3 [file 41467_2026_74385_MOESM3_ESM.zip › source data/Bioinformatics data/NDH-II_genomic_context.pdf]
