# Supplementary material for: A Bacillales-specific tubular scaffold essential for NADH dehydrogenase activity
Source: Nat Commun. 2026 Jun 18;17:7717. doi: 10.1038/s41467-026-74385-2 (PMC13433803; doi:10.1038/s41467-026-74385-2)
Supplement: Supplementary file 3 [file 41467_2026_74385_MOESM3_ESM.zip › source data/Bioinformatics data/Supplemental data Bioinformatics.pdf]

Supplemental data Bioinformatics

NDH-II_alignment_specific_residues.fasta. Alignment of NDH-II protein sequences used to compute Cm score.

NDH-II.fasta. full dataset of Ndh protein sequences.

HMP.fasta. full dataset of HMP protein sequences.

NDH-II_trimmed_alignment.phy. Sampled and trimmed alignment of NDH-II protein sequences.

NDH-II_tree.treefile. ML phylogeny of NDH-II.

NDH-II_alignment.fasta. Sampled alignment of NDH-II protein sequences.

HMP_trimmed_alignment.phy. Sampled and trimmed alignment of HMP protein sequences.

HMP_tree.treefile. ML phylogeny of HMP.

HMP_alignment.fasta. Sampled alignment of HMP protein sequences.

NDH-II_genomic_context.pdf. Genomic context of NDH-II. NDH-II: green, FAM0000006486 (polyprenyl synthetase): black, FAM0000055849 (peptidase S8): pink, and FAM0000057223 (HMP): blue.
