# Supplementary material for: A Bacillales-specific tubular scaffold essential for NADH dehydrogenase activity
Source: Nat Commun. 2026 Jun 18;17:7717. doi: 10.1038/s41467-026-74385-2 (PMC13433803; doi:10.1038/s41467-026-74385-2)
Supplement: Supplementary file 3 [file 41467_2026_74385_MOESM3_ESM.zip › source data/Cryo-EM data/Global/D_1292149434_val-report-full-annotate_P1.pdf]

Full wwPDB EM Validation Report (i)

Sep 11, 2025 – 10:12 am BST

PDB ID	:	9SNK / pdb_00009snk
EMDB ID	:	EMD-55048
Title	:	CryoEM structure of NADH:quinone oxidoreductases YjlCD from Bacillus subtilis
Deposited on	:	2025-09-11
Resolution	:	2.59 Å(reported)

This wwPDB validation report is for manuscript review

This is a Full wwPDB EM Validation Report.

This report is produced by the wwPDB biocuration pipeline after annotation of the structure.

We welcome your comments at *validation@mail.wwpdb.org*
A user guide is available at
https://www.wwpdb.org/validation/2017/EMValidationReportHelp
with specific help available everywhere you see the (i) symbol.

The types of validation reports are described at
http://www.wwpdb.org/validation/2017/FAQs#types.

The following versions of software and data (see references (i)) were used in the production of this report:

EMDB validation analysis	:	0.0.1.dev126
Mogul	:	1.8.4, CSD as541be (2020)
MolProbity	:	FAILED
buster-report	:	1.1.7 (2018)
Percentile statistics	:	20231227.v01 (using entries in the PDB archive December 27th 2023)
MapQ	:	1.9.13
Ideal geometry (proteins)	:	Engh & Huber (2001)
Ideal geometry (DNA, RNA)	:	Parkinson et al. (1996)

For Manuscript Review

1 Overall quality at a glance (i)

The following experimental techniques were used to determine the structure:
ELECTRON MICROSCOPY

The reported resolution of this entry is 2.59 Å.

There are no overall percentile quality scores available for this entry.

MolProbity failed to run properly - the sequence quality summary graphics cannot be shown.

For Manuscript Review

Validation Pipeline (wwPDB-VP) : 2.45.1

2 Entry composition (i)

There are 10 unique types of molecules in this entry. The entry contains 17888 atoms, of which 0 are hydrogens and 0 are deuteriums.

In the tables below, the AltConf column contains the number of residues with at least one atom in alternate conformation and the Trace column contains the number of residues modelled with at most 2 atoms.

- Molecule 1 is a protein called NADH dehydrogenase-like protein YjlD.

Mol	Chain	Residues	Atoms					AltConf	Trace
			Total	C	N	O	S		
1	A	391	2979	1906	502	566	5	5	0
1	C	391	2979	1906	502	566	5	5	0
1	F	391	2979	1906	502	566	5	5	0
1	I	391	2979	1906	502	566	5	5	0

- Molecule 2 is a protein called YjlC.

Mol	Chain	Residues	Atoms					AltConf	Trace
			Total	C	N	O	S		
2	B	122	961	607	156	196	2	1	0
2	D	122	961	607	156	196	2	1	0
2	G	122	961	607	156	196	2	1	0
2	J	122	961	607	156	196	2	1	0

- Molecule 3 is FLAVIN-ADENINE DINUCLEOTIDE (CCD ID: FAD) (formula: $C_{27}H_{33}N_9O_{15}P_2$) (labeled as "Ligand of Interest" by depositor).

For Manuscript Review

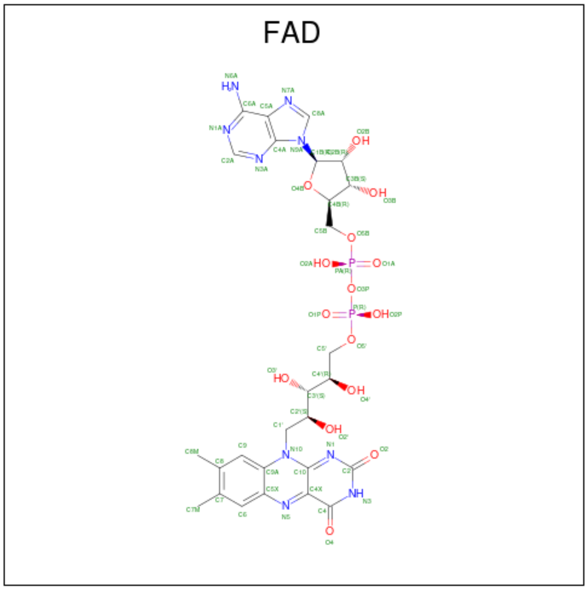

Mol	Chain	Residues	Atoms	AltConf
3	A	1	Total C N O P<br>53 27 9 15 2	0
3	C	1	Total C N O P<br>53 27 9 15 2	0
3	F	1	Total C N O P<br>53 27 9 15 2	0
3	I	1	Total C N O P<br>53 27 9 15 2	0

- Molecule 4 is HEPTANOIC ACID (CCD ID: SHV) (formula: $C_7H_{14}O_2$).

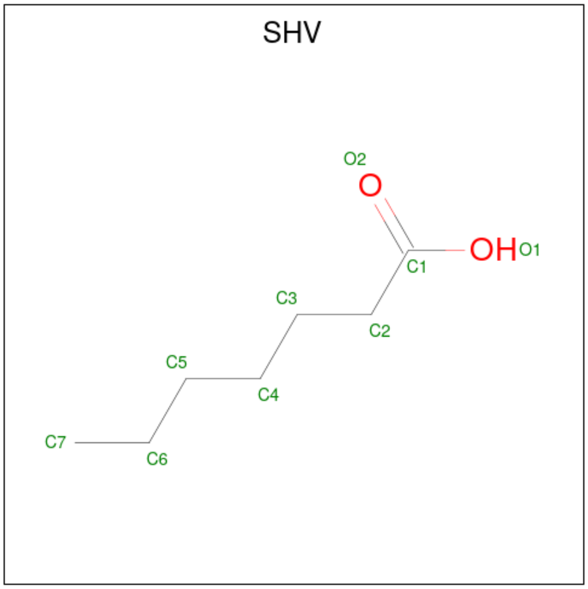

For Manuscript Review

Mol	Chain	Residues	Atoms	AltConf
4	A	1	Total C O<br>9 7 2	0
4	A	1	Total C O<br>9 7 2	0
4	A	1	Total C O<br>9 7 2	0
4	B	1	Total C O<br>9 7 2	0
4	B	1	Total C O<br>9 7 2	0
4	C	1	Total C O<br>9 7 2	0
4	C	1	Total C O<br>9 7 2	0
4	C	1	Total C O<br>9 7 2	0
4	D	1	Total C O<br>9 7 2	0
4	D	1	Total C O<br>9 7 2	0
4	F	1	Total C O<br>9 7 2	0
4	F	1	Total C O<br>9 7 2	0
4	F	1	Total C O<br>9 7 2	0
4	G	1	Total C O<br>9 7 2	0
4	G	1	Total C O<br>9 7 2	0
4	I	1	Total C O<br>9 7 2	0
4	I	1	Total C O<br>9 7 2	0
4	I	1	Total C O<br>9 7 2	0
4	J	1	Total C O<br>9 7 2	0
4	J	1	Total C O<br>9 7 2	0

- Molecule 5 is HEXANOIC ACID (CCD ID: 6NA) (formula: $C_6H_{12}O_2$).

For Manuscript Review

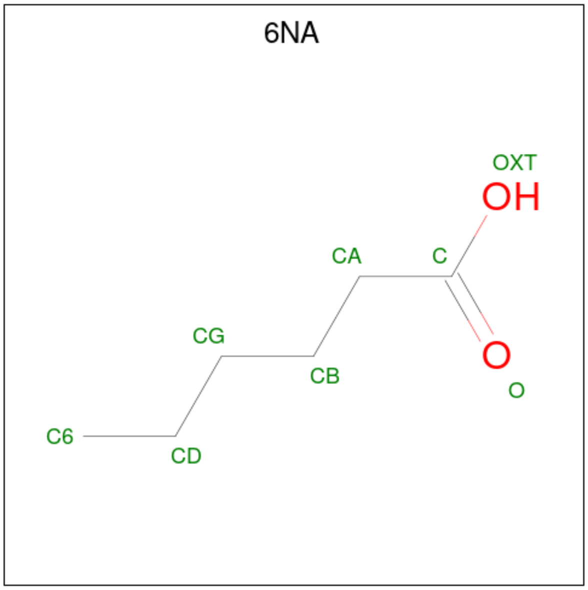

Mol	Chain	Residues	Atoms	AltConf
5	A	1	Total C O<br>8 6 2	0
5	A	1	Total C O<br>8 6 2	0
5	A	1	Total C O<br>8 6 2	0
5	B	1	Total C O<br>8 6 2	0
5	C	1	Total C O<br>8 6 2	0
5	C	1	Total C O<br>8 6 2	0
5	C	1	Total C O<br>8 6 2	0
5	D	1	Total C O<br>8 6 2	0
5	F	1	Total C O<br>8 6 2	0
5	F	1	Total C O<br>8 6 2	0
5	F	1	Total C O<br>8 6 2	0
5	G	1	Total C O<br>8 6 2	0
5	I	1	Total C O<br>8 6 2	0
5	I	1	Total C O<br>8 6 2	0

Continued on next page...

For Manuscript Review

Continued from previous page...

Mol	Chain	Residues	Atoms	AltConf
5	I	1	Total C O<br>8 6 2	0
5	J	1	Total C O<br>8 6 2	0

- Molecule 6 is 1,2-Distearoyl-sn-glycerophosphoethanolamine (CCD ID: 3PE) (formula: $C_{41}H_{82}NO_8P$).

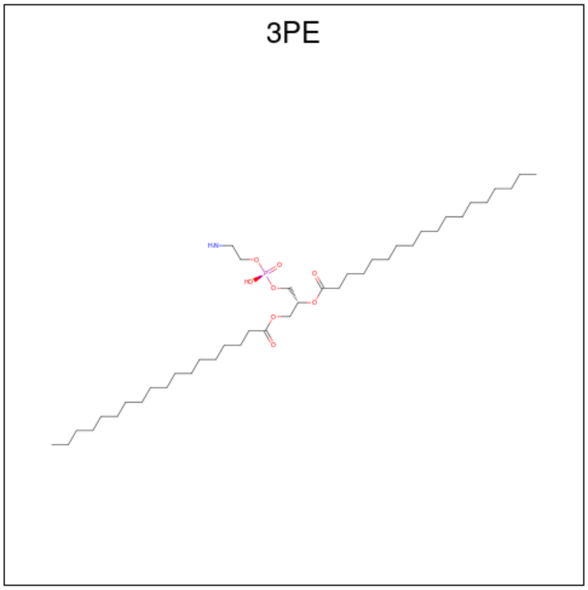

Mol	Chain	Residues	Atoms	AltConf
6	A	1	Total C N O P<br>32 22 1 8 1	0
6	B	1	Total C N O P<br>22 12 1 8 1	0
6	B	1	Total C N O P<br>22 12 1 8 1	0
6	C	1	Total C N O P<br>32 22 1 8 1	0
6	D	1	Total C N O P<br>22 12 1 8 1	0
6	F	1	Total C N O P<br>32 22 1 8 1	0
6	G	1	Total C N O P<br>22 12 1 8 1	0
6	I	1	Total C N O P<br>32 22 1 8 1	0

- Molecule 7 is PENTANOIC ACID (CCD ID: LEA) (formula: $C_5H_{10}O_2$).

For Manuscript Review

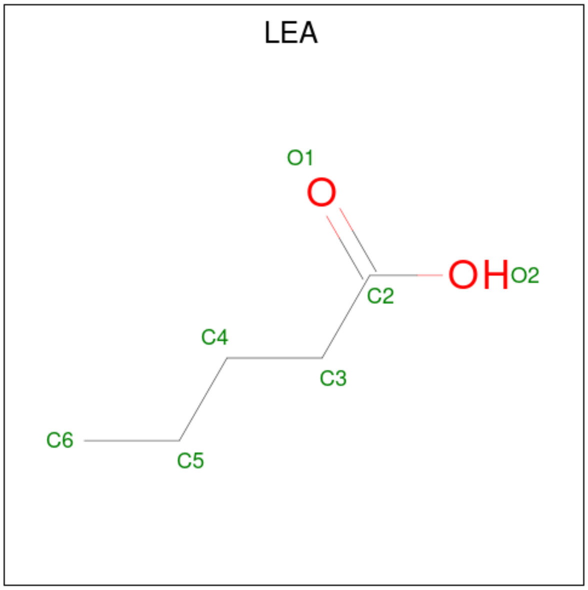

Mol	Chain	Residues	Atoms	AltConf
7	A	1	Total C O<br>7 5 2	0
7	A	1	Total C O<br>7 5 2	0
7	B	1	Total C O<br>7 5 2	0
7	B	1	Total C O<br>7 5 2	0
7	B	1	Total C O<br>7 5 2	0
7	C	1	Total C O<br>7 5 2	0
7	C	1	Total C O<br>7 5 2	0
7	D	1	Total C O<br>7 5 2	0
7	D	1	Total C O<br>7 5 2	0
7	D	1	Total C O<br>7 5 2	0
7	F	1	Total C O<br>7 5 2	0
7	F	1	Total C O<br>7 5 2	0
7	G	1	Total C O<br>7 5 2	0
7	G	1	Total C O<br>7 5 2	0

Continued on next page...

For Manuscript Review

Continued from previous page...

Mol	Chain	Residues	Atoms	AltConf
7	G	1	Total C O<br>7 5 2	0
7	I	1	Total C O<br>7 5 2	0
7	I	1	Total C O<br>7 5 2	0
7	J	1	Total C O<br>7 5 2	0
7	J	1	Total C O<br>7 5 2	0
7	J	1	Total C O<br>7 5 2	0

- Molecule 8 is GLYCEROL (CCD ID: GOL) (formula: $C_3H_8O_3$).

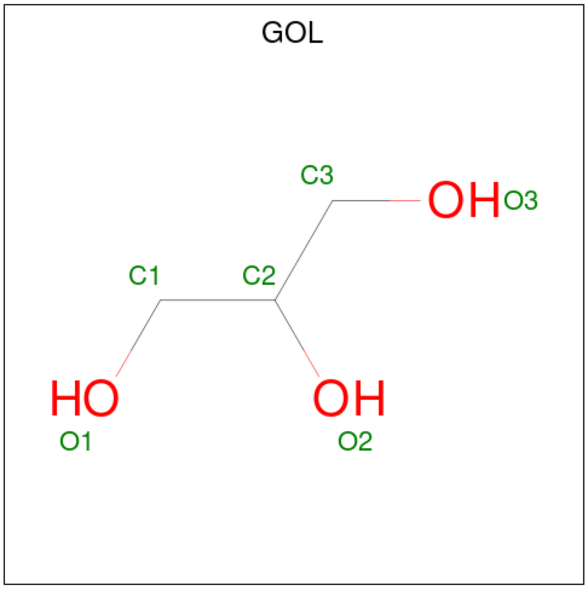

Mol	Chain	Residues	Atoms	AltConf
8	A	1	Total C O<br>6 3 3	0
8	A	1	Total C O<br>6 3 3	0
8	A	1	Total C O<br>6 3 3	0
8	A	1	Total C O<br>6 3 3	0
8	A	1	Total C O<br>6 3 3	0

Continued on next page...

For Manuscript Review

Continued from previous page...

Mol	Chain	Residues	Atoms	AltConf
8	A	1	Total C O<br>6 3 3	0
8	A	1	Total C O<br>6 3 3	0
8	C	1	Total C O<br>6 3 3	0
8	C	1	Total C O<br>6 3 3	0
8	C	1	Total C O<br>6 3 3	0
8	C	1	Total C O<br>6 3 3	0
8	C	1	Total C O<br>6 3 3	0
8	C	1	Total C O<br>6 3 3	0
8	C	1	Total C O<br>6 3 3	0
8	F	1	Total C O<br>6 3 3	0
8	F	1	Total C O<br>6 3 3	0
8	F	1	Total C O<br>6 3 3	0
8	F	1	Total C O<br>6 3 3	0
8	F	1	Total C O<br>6 3 3	0
8	F	1	Total C O<br>6 3 3	0
8	F	1	Total C O<br>6 3 3	0
8	I	1	Total C O<br>6 3 3	0
8	I	1	Total C O<br>6 3 3	0
8	I	1	Total C O<br>6 3 3	0
8	I	1	Total C O<br>6 3 3	0
8	I	1	Total C O<br>6 3 3	0

Continued on next page...

For Manuscript Review

Continued from previous page...

Mol	Chain	Residues	Atoms	AltConf
8	I	1	Total C O<br>6 3 3	0
8	I	1	Total C O<br>6 3 3	0

- Molecule 9 is DECANOIC ACID (CCD ID: DKA) (formula: $C_{10}H_{20}O_2$).

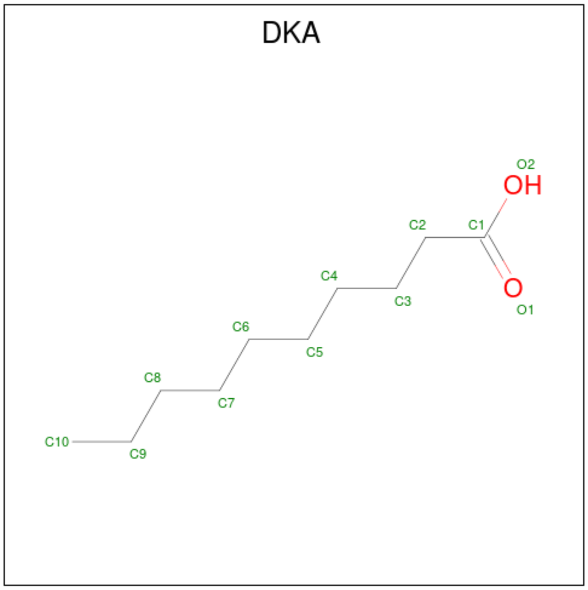

Mol	Chain	Residues	Atoms	AltConf
9	B	1	Total C O<br>12 10 2	0
9	B	1	Total C O<br>12 10 2	0
9	D	1	Total C O<br>12 10 2	0
9	D	1	Total C O<br>12 10 2	0
9	G	1	Total C O<br>12 10 2	0
9	G	1	Total C O<br>12 10 2	0
9	J	1	Total C O<br>12 10 2	0
9	J	1	Total C O<br>12 10 2	0

- Molecule 10 is water.

For Manuscript Review

Mol	Chain	Residues	Atoms		AltConf
10	A	215	Total 215	O 215	0
10	B	34	Total 34	O 34	0
10	C	215	Total 215	O 215	0
10	D	32	Total 32	O 32	0
10	F	215	Total 215	O 215	0
10	G	32	Total 32	O 32	0
10	I	215	Total 215	O 215	0
10	J	30	Total 30	O 30	0

MolProbity failed to run properly - this section is therefore empty.

For Manuscript Review

3 Experimental information (i)

Property	Value	Source
EM reconstruction method	SINGLE PARTICLE	Depositor
Imposed symmetry	POINT, C4	Depositor
Number of particles used	691400	Depositor
Resolution determination method	FSC 0.143 CUT-OFF	Depositor
CTF correction method	PHASE FLIPPING AND AMPLITUDE CORRECTION	Depositor
Microscope	TFS KRIOS	Depositor
Voltage (kV)	300	Depositor
Electron dose ($e^-/\text{Å}^2$)	40	Depositor
Minimum defocus (nm)	500	Depositor
Maximum defocus (nm)	2900	Depositor
Magnification	215000	Depositor
Image detector	FEI FALCON IV (4k x 4k)	Depositor
Maximum map value	0.905	Depositor
Minimum map value	-0.541	Depositor
Average map value	0.000	Depositor
Map value standard deviation	0.018	Depositor
Recommended contour level	0.068	Depositor
Map size (Å)	290.0, 290.0, 290.0	wwPDB
Map dimensions	500, 500, 500	wwPDB
Map angles (°)	90.0, 90.0, 90.0	wwPDB
Pixel spacing (Å)	0.58, 0.58, 0.58	Depositor

For Manuscript Review

4 Model quality (i)

4.1 Standard geometry (i)

MolProbity failed to run properly - this section is therefore empty.

4.2 Too-close contacts (i)

MolProbity failed to run properly - this section is therefore empty.

4.3 Torsion angles (i)

4.3.1 Protein backbone (i)

MolProbity failed to run properly - this section is therefore empty.

4.3.2 Protein sidechains (i)

MolProbity failed to run properly - this section is therefore empty.

4.3.3 RNA (i)

MolProbity failed to run properly - this section is therefore empty.

4.4 Non-standard residues in protein, DNA, RNA chains (i)

There are no non-standard protein/DNA/RNA residues in this entry.

4.5 Carbohydrates (i)

There are no oligosaccharides in this entry.

4.6 Ligand geometry (i)

104 ligands are modelled in this entry.

In the following table, the Counts columns list the number of bonds (or angles) for which Mogul statistics could be retrieved, the number of bonds (or angles) that are observed in the model and the number of bonds (or angles) that are defined in the Chemical Component Dictionary. The Link column lists molecule types, if any, to which the group is linked. The Z score for a bond

For Manuscript Review

length (or angle) is the number of standard deviations the observed value is removed from the expected value. A bond length (or angle) with $|Z| > 2$ is considered an outlier worth inspection. RMSZ is the root-mean-square of all Z scores of the bond lengths (or angles).

Mol	Type	Chain	Res	Link	Bond lengths			Bond angles		
					Counts	RMSZ	$\#\|Z\| > 2$	Counts	RMSZ	$\#\|Z\| > 2$
9	DKA	B	202	-	11,11,11	0.75	0	11,11,11	0.66	0
5	6NA	G	209	-	7,7,7	0.93	0	7,7,7	0.81	0
4	SHV	C	405	-	8,8,8	0.88	0	8,8,8	0.75	0
7	LEA	G	204	-	6,6,6	1.00	0	6,6,6	0.88	0
7	LEA	B	201	-	6,6,6	1.00	0	6,6,6	0.88	0
9	DKA	D	202	-	11,11,11	0.75	0	11,11,11	0.66	0
8	GOL	I	412	-	5,5,5	0.36	0	5,5,5	0.37	0
6	3PE	F	407	-	31,31,50	0.60	0	34,36,55	0.54	0
7	LEA	A	409	-	6,6,6	1.00	0	6,6,6	0.88	0
5	6NA	A	403	-	7,7,7	0.92	0	7,7,7	0.89	0
4	SHV	B	206	-	8,8,8	0.88	0	8,8,8	0.80	0
6	3PE	I	407	-	31,31,50	0.60	0	34,36,55	0.54	0
7	LEA	I	408	-	6,6,6	1.01	0	6,6,6	0.88	0
8	GOL	F	410	-	5,5,5	0.38	0	5,5,5	0.35	0
5	6NA	F	406	-	7,7,7	0.92	0	7,7,7	0.87	0
5	6NA	C	403	-	7,7,7	0.92	0	7,7,7	0.89	0
4	SHV	G	203	-	8,8,8	0.87	0	8,8,8	0.77	0
7	LEA	D	204	-	6,6,6	1.00	0	6,6,6	0.88	0
8	GOL	I	410	-	5,5,5	0.38	0	5,5,5	0.35	0
6	3PE	G	205	-	21,21,50	0.71	0	24,26,55	0.62	0
6	3PE	A	407	-	31,31,50	0.60	0	34,36,55	0.54	0
8	GOL	C	410	-	5,5,5	0.38	0	5,5,5	0.35	0
4	SHV	D	203	-	8,8,8	0.87	0	8,8,8	0.77	0
8	GOL	C	412	-	5,5,5	0.36	0	5,5,5	0.37	0
8	GOL	C	411	-	5,5,5	0.35	0	5,5,5	0.40	0
8	GOL	I	414	-	5,5,5	0.35	0	5,5,5	0.35	0
8	GOL	C	414	-	5,5,5	0.35	0	5,5,5	0.35	0
4	SHV	G	206	-	8,8,8	0.88	0	8,8,8	0.80	0
9	DKA	G	208	-	11,11,11	0.76	0	11,11,11	0.68	0
6	3PE	D	205	-	21,21,50	0.71	0	24,26,55	0.62	0
9	DKA	J	203	-	11,11,11	0.75	0	11,11,11	0.66	0
7	LEA	C	408	-	6,6,6	1.01	0	6,6,6	0.88	0
5	6NA	A	406	-	7,7,7	0.92	0	7,7,7	0.87	0
8	GOL	A	417	-	5,5,5	0.35	0	5,5,5	0.39	0
4	SHV	A	405	-	8,8,8	0.88	0	8,8,8	0.75	0
8	GOL	C	416	-	5,5,5	0.34	0	5,5,5	0.37	0
7	LEA	B	204	-	6,6,6	1.00	0	6,6,6	0.88	0
7	LEA	G	207	-	6,6,6	1.00	0	6,6,6	0.90	0
4	SHV	A	404	-	8,8,8	0.88	0	8,8,8	0.75	0

For Manuscript Review

Mol	Type	Chain	Res	Link	Bond lengths			Bond angles		
					Counts	RMSZ	#|Z| > 2	Counts	RMSZ	#|Z| > 2
7	LEA	F	409	-	6,6,6	1.00	0	6,6,6	0.88	0
7	LEA	J	207	-	6,6,6	1.00	0	6,6,6	0.90	0
5	6NA	F	403	-	7,7,7	0.92	0	7,7,7	0.89	0
4	SHV	B	203	-	8,8,8	0.87	0	8,8,8	0.77	0
8	GOL	I	411	-	5,5,5	0.35	0	5,5,5	0.40	0
5	6NA	F	413	-	7,7,7	0.92	0	7,7,7	0.81	0
8	GOL	F	416	-	5,5,5	0.34	0	5,5,5	0.37	0
9	DKA	G	202	-	11,11,11	0.75	0	11,11,11	0.66	0
3	FAD	I	401	-	53,58,58	0.96	2 (3%)	68,89,89	0.81	0
8	GOL	F	414	-	5,5,5	0.35	0	5,5,5	0.35	0
8	GOL	C	417	-	5,5,5	0.35	0	5,5,5	0.39	0
7	LEA	A	408	-	6,6,6	1.01	0	6,6,6	0.88	0
7	LEA	J	205	-	6,6,6	1.00	0	6,6,6	0.88	0
8	GOL	A	416	-	5,5,5	0.34	0	5,5,5	0.37	0
9	DKA	B	208	-	11,11,11	0.76	0	11,11,11	0.68	0
6	3PE	B	210	-	21,21,50	0.71	0	24,26,55	0.62	0
4	SHV	C	402	-	8,8,8	0.88	0	8,8,8	0.78	0
4	SHV	I	404	-	8,8,8	0.88	0	8,8,8	0.75	0
5	6NA	I	413	-	7,7,7	0.92	0	7,7,7	0.81	0
8	GOL	F	415	-	5,5,5	0.35	0	5,5,5	0.38	0
8	GOL	I	417	-	5,5,5	0.35	0	5,5,5	0.39	0
9	DKA	J	208	-	11,11,11	0.76	0	11,11,11	0.68	0
8	GOL	I	415	-	5,5,5	0.35	0	5,5,5	0.38	0
8	GOL	C	415	-	5,5,5	0.35	0	5,5,5	0.38	0
3	FAD	A	401	-	53,58,58	0.96	2 (3%)	68,89,89	0.81	0
5	6NA	C	413	-	7,7,7	0.92	0	7,7,7	0.81	0
8	GOL	F	417	-	5,5,5	0.35	0	5,5,5	0.39	0
5	6NA	I	406	-	7,7,7	0.92	0	7,7,7	0.87	0
7	LEA	D	207	-	6,6,6	1.00	0	6,6,6	0.90	0
8	GOL	F	411	-	5,5,5	0.35	0	5,5,5	0.40	0
8	GOL	A	414	-	5,5,5	0.35	0	5,5,5	0.35	0
3	FAD	C	401	-	53,58,58	0.96	2 (3%)	68,89,89	0.81	0
8	GOL	F	412	-	5,5,5	0.36	0	5,5,5	0.37	0
5	6NA	J	201	-	7,7,7	0.93	0	7,7,7	0.81	0
7	LEA	J	202	-	6,6,6	1.00	0	6,6,6	0.88	0
4	SHV	J	206	-	8,8,8	0.88	0	8,8,8	0.80	0
8	GOL	A	415	-	5,5,5	0.35	0	5,5,5	0.38	0
4	SHV	F	402	-	8,8,8	0.88	0	8,8,8	0.78	0
8	GOL	A	411	-	5,5,5	0.35	0	5,5,5	0.40	0
9	DKA	D	208	-	11,11,11	0.76	0	11,11,11	0.68	0
7	LEA	B	207	-	6,6,6	1.00	0	6,6,6	0.90	0
4	SHV	J	204	-	8,8,8	0.87	0	8,8,8	0.77	0

For Manuscript Review

Mol	Type	Chain	Res	Link	Bond lengths			Bond angles		
					Counts	RMSZ	#|Z| > 2	Counts	RMSZ	#|Z| > 2
7	LEA	G	201	-	6,6,6	1.00	0	6,6,6	0.88	0
5	6NA	B	209	-	7,7,7	0.93	0	7,7,7	0.81	0
4	SHV	F	404	-	8,8,8	0.88	0	8,8,8	0.75	0
7	LEA	F	408	-	6,6,6	1.01	0	6,6,6	0.88	0
4	SHV	F	405	-	8,8,8	0.88	0	8,8,8	0.75	0
8	GOL	A	412	-	5,5,5	0.36	0	5,5,5	0.37	0
7	LEA	C	409	-	6,6,6	1.00	0	6,6,6	0.88	0
8	GOL	I	416	-	5,5,5	0.34	0	5,5,5	0.37	0
4	SHV	I	402	-	8,8,8	0.88	0	8,8,8	0.78	0
6	3PE	C	407	-	31,31,50	0.60	0	34,36,55	0.54	0
4	SHV	A	402	-	8,8,8	0.88	0	8,8,8	0.78	0
3	FAD	F	401	-	53,58,58	0.96	2 (3%)	68,89,89	0.81	0
5	6NA	C	406	-	7,7,7	0.92	0	7,7,7	0.87	0
4	SHV	D	206	-	8,8,8	0.88	0	8,8,8	0.80	0
8	GOL	A	410	-	5,5,5	0.38	0	5,5,5	0.35	0
6	3PE	B	205	-	21,21,50	0.71	0	24,26,55	0.62	0
7	LEA	I	409	-	6,6,6	1.00	0	6,6,6	0.88	0
7	LEA	D	201	-	6,6,6	1.00	0	6,6,6	0.88	0
4	SHV	I	405	-	8,8,8	0.88	0	8,8,8	0.75	0
5	6NA	D	209	-	7,7,7	0.93	0	7,7,7	0.81	0
5	6NA	I	403	-	7,7,7	0.92	0	7,7,7	0.89	0
5	6NA	A	413	-	7,7,7	0.92	0	7,7,7	0.81	0
4	SHV	C	404	-	8,8,8	0.88	0	8,8,8	0.75	0

In the following table, the Chirals column lists the number of chiral outliers, the number of chiral centers analysed, the number of these observed in the model and the number defined in the Chemical Component Dictionary. Similar counts are reported in the Torsion and Rings columns. '-' means no outliers of that kind were identified.

Mol	Type	Chain	Res	Link	Chirals	Torsions	Rings
9	DKA	B	202	-	-	1/9/9/9	-
5	6NA	G	209	-	-	0/5/5/5	-
4	SHV	C	405	-	-	4/6/6/6	-
7	LEA	G	204	-	-	2/4/4/4	-
7	LEA	B	201	-	-	2/4/4/4	-
9	DKA	D	202	-	-	1/9/9/9	-
8	GOL	I	412	-	-	2/4/4/4	-
6	3PE	F	407	-	-	14/35/35/54	-
7	LEA	A	409	-	-	1/4/4/4	-
5	6NA	A	403	-	-	1/5/5/5	-
4	SHV	B	206	-	-	2/6/6/6	-

Continued on next page...

For Manuscript Review

Continued from previous page...

Mol	Type	Chain	Res	Link	Chirals	Torsions	Rings
6	3PE	I	407	-	-	14/35/35/54	-
7	LEA	I	408	-	-	0/4/4/4	-
8	GOL	F	410	-	-	0/4/4/4	-
5	6NA	F	406	-	-	0/5/5/5	-
5	6NA	C	403	-	-	1/5/5/5	-
4	SHV	G	203	-	-	3/6/6/6	-
7	LEA	D	204	-	-	2/4/4/4	-
8	GOL	I	410	-	-	0/4/4/4	-
6	3PE	G	205	-	-	8/25/25/54	-
6	3PE	A	407	-	-	14/35/35/54	-
8	GOL	C	410	-	-	0/4/4/4	-
4	SHV	D	203	-	-	3/6/6/6	-
8	GOL	C	412	-	-	2/4/4/4	-
8	GOL	C	411	-	-	0/4/4/4	-
8	GOL	I	414	-	-	0/4/4/4	-
8	GOL	C	414	-	-	0/4/4/4	-
4	SHV	G	206	-	-	2/6/6/6	-
9	DKA	G	208	-	-	2/9/9/9	-
6	3PE	D	205	-	-	8/25/25/54	-
9	DKA	J	203	-	-	1/9/9/9	-
7	LEA	C	408	-	-	0/4/4/4	-
5	6NA	A	406	-	-	0/5/5/5	-
8	GOL	A	417	-	-	0/4/4/4	-
4	SHV	A	405	-	-	4/6/6/6	-
8	GOL	C	416	-	-	0/4/4/4	-
7	LEA	B	204	-	-	2/4/4/4	-
7	LEA	G	207	-	-	3/4/4/4	-
4	SHV	A	404	-	-	3/6/6/6	-
7	LEA	F	409	-	-	1/4/4/4	-
7	LEA	J	207	-	-	3/4/4/4	-
5	6NA	F	403	-	-	1/5/5/5	-
4	SHV	B	203	-	-	3/6/6/6	-
8	GOL	I	411	-	-	0/4/4/4	-
5	6NA	F	413	-	-	2/5/5/5	-
8	GOL	F	416	-	-	0/4/4/4	-
9	DKA	G	202	-	-	1/9/9/9	-
3	FAD	I	401	-	-	1/30/50/50	0/6/6/6
8	GOL	F	414	-	-	0/4/4/4	-

Continued on next page...

For Manuscript Review

Continued from previous page...

Mol	Type	Chain	Res	Link	Chirals	Torsions	Rings
8	GOL	C	417	-	-	0/4/4/4	-
7	LEA	A	408	-	-	0/4/4/4	-
7	LEA	J	205	-	-	2/4/4/4	-
8	GOL	A	416	-	-	0/4/4/4	-
9	DKA	B	208	-	-	2/9/9/9	-
6	3PE	B	210	-	-	8/25/25/54	-
4	SHV	C	402	-	-	2/6/6/6	-
4	SHV	I	404	-	-	3/6/6/6	-
5	6NA	I	413	-	-	2/5/5/5	-
8	GOL	F	415	-	-	0/4/4/4	-
8	GOL	I	417	-	-	0/4/4/4	-
9	DKA	J	208	-	-	2/9/9/9	-
8	GOL	I	415	-	-	0/4/4/4	-
8	GOL	C	415	-	-	0/4/4/4	-
3	FAD	A	401	-	-	1/30/50/50	0/6/6/6
5	6NA	C	413	-	-	2/5/5/5	-
8	GOL	F	417	-	-	0/4/4/4	-
5	6NA	I	406	-	-	0/5/5/5	-
7	LEA	D	207	-	-	3/4/4/4	-
8	GOL	F	411	-	-	0/4/4/4	-
8	GOL	A	414	-	-	0/4/4/4	-
3	FAD	C	401	-	-	1/30/50/50	0/6/6/6
8	GOL	F	412	-	-	2/4/4/4	-
5	6NA	J	201	-	-	0/5/5/5	-
7	LEA	J	202	-	-	2/4/4/4	-
4	SHV	J	206	-	-	2/6/6/6	-
8	GOL	A	415	-	-	0/4/4/4	-
4	SHV	F	402	-	-	2/6/6/6	-
8	GOL	A	411	-	-	0/4/4/4	-
9	DKA	D	208	-	-	2/9/9/9	-
7	LEA	B	207	-	-	3/4/4/4	-
4	SHV	J	204	-	-	3/6/6/6	-
7	LEA	G	201	-	-	2/4/4/4	-
5	6NA	B	209	-	-	0/5/5/5	-
4	SHV	F	404	-	-	3/6/6/6	-
7	LEA	F	408	-	-	0/4/4/4	-
4	SHV	F	405	-	-	4/6/6/6	-
8	GOL	A	412	-	-	2/4/4/4	-

Continued on next page...

For Manuscript Review

Continued from previous page...

Mol	Type	Chain	Res	Link	Chirals	Torsions	Rings
7	LEA	C	409	-	-	1/4/4/4	-
8	GOL	I	416	-	-	0/4/4/4	-
4	SHV	I	402	-	-	2/6/6/6	-
6	3PE	C	407	-	-	14/35/35/54	-
4	SHV	A	402	-	-	2/6/6/6	-
3	FAD	F	401	-	-	1/30/50/50	0/6/6/6
5	6NA	C	406	-	-	0/5/5/5	-
4	SHV	D	206	-	-	2/6/6/6	-
8	GOL	A	410	-	-	0/4/4/4	-
6	3PE	B	205	-	-	8/25/25/54	-
7	LEA	I	409	-	-	1/4/4/4	-
7	LEA	D	201	-	-	2/4/4/4	-
4	SHV	I	405	-	-	4/6/6/6	-
5	6NA	D	209	-	-	0/5/5/5	-
5	6NA	I	403	-	-	1/5/5/5	-
5	6NA	A	413	-	-	2/5/5/5	-
4	SHV	C	404	-	-	3/6/6/6	-

All (8) bond length outliers are listed below:

Mol	Chain	Res	Type	Atoms	Z	Observed(Å)	Ideal(Å)
3	A	401	FAD	C8A-N7A	-2.57	1.30	1.34
3	C	401	FAD	C8A-N7A	-2.57	1.30	1.34
3	F	401	FAD	C8A-N7A	-2.57	1.30	1.34
3	I	401	FAD	C8A-N7A	-2.57	1.30	1.34
3	A	401	FAD	C5X-N5	-2.27	1.35	1.39
3	C	401	FAD	C5X-N5	-2.27	1.35	1.39
3	F	401	FAD	C5X-N5	-2.27	1.35	1.39
3	I	401	FAD	C5X-N5	-2.27	1.35	1.39

There are no bond angle outliers.

There are no chirality outliers.

All (212) torsion outliers are listed below:

Mol	Chain	Res	Type	Atoms
6	A	407	3PE	C11-O13-P-O12
6	A	407	3PE	C22-C21-O21-C2
6	B	205	3PE	C1-O11-P-O14
6	B	205	3PE	C11-O13-P-O12

Continued on next page...

For Manuscript Review

Continued from previous page...

Mol	Chain	Res	Type	Atoms
6	B	205	3PE	C11-O13-P-O14
6	B	210	3PE	C1-O11-P-O14
6	B	210	3PE	C11-O13-P-O12
6	B	210	3PE	C11-O13-P-O14
6	C	407	3PE	C11-O13-P-O12
6	C	407	3PE	C22-C21-O21-C2
6	D	205	3PE	C1-O11-P-O14
6	D	205	3PE	C11-O13-P-O12
6	D	205	3PE	C11-O13-P-O14
6	F	407	3PE	C11-O13-P-O12
6	F	407	3PE	C22-C21-O21-C2
6	G	205	3PE	C1-O11-P-O14
6	G	205	3PE	C11-O13-P-O12
6	G	205	3PE	C11-O13-P-O14
6	I	407	3PE	C11-O13-P-O12
6	I	407	3PE	C22-C21-O21-C2
8	A	412	GOL	O1-C1-C2-C3
8	C	412	GOL	O1-C1-C2-C3
8	F	412	GOL	O1-C1-C2-C3
8	I	412	GOL	O1-C1-C2-C3
6	B	205	3PE	C32-C31-O31-C3
6	B	210	3PE	C32-C31-O31-C3
6	D	205	3PE	C32-C31-O31-C3
6	G	205	3PE	C32-C31-O31-C3
6	B	205	3PE	O32-C31-O31-C3
6	B	210	3PE	O32-C31-O31-C3
6	D	205	3PE	O32-C31-O31-C3
6	G	205	3PE	O32-C31-O31-C3
6	A	407	3PE	O22-C21-O21-C2
6	C	407	3PE	O22-C21-O21-C2
6	F	407	3PE	O22-C21-O21-C2
6	I	407	3PE	O22-C21-O21-C2
5	A	413	6NA	C-CA-CB-CG
5	C	413	6NA	C-CA-CB-CG
5	F	413	6NA	C-CA-CB-CG
5	I	413	6NA	C-CA-CB-CG
6	A	407	3PE	C11-O13-P-O11
6	B	205	3PE	C1-O11-P-O13
6	B	205	3PE	C11-O13-P-O11
6	B	210	3PE	C1-O11-P-O13
6	B	210	3PE	C11-O13-P-O11
6	C	407	3PE	C11-O13-P-O11

Continued on next page...

For Manuscript Review

Continued from previous page...

Mol	Chain	Res	Type	Atoms
6	D	205	3PE	C1-O11-P-O13
6	D	205	3PE	C11-O13-P-O11
6	F	407	3PE	C11-O13-P-O11
6	G	205	3PE	C1-O11-P-O13
6	G	205	3PE	C11-O13-P-O11
6	I	407	3PE	C11-O13-P-O11
7	B	207	LEA	C2-C3-C4-C5
7	D	207	LEA	C2-C3-C4-C5
7	G	207	LEA	C2-C3-C4-C5
7	J	207	LEA	C2-C3-C4-C5
6	A	407	3PE	C32-C31-O31-C3
6	C	407	3PE	C32-C31-O31-C3
6	F	407	3PE	C32-C31-O31-C3
6	I	407	3PE	C32-C31-O31-C3
9	B	208	DKA	C3-C4-C5-C6
9	D	208	DKA	C3-C4-C5-C6
9	G	208	DKA	C3-C4-C5-C6
9	J	208	DKA	C3-C4-C5-C6
6	A	407	3PE	O32-C31-O31-C3
6	C	407	3PE	O32-C31-O31-C3
6	F	407	3PE	O32-C31-O31-C3
6	I	407	3PE	O32-C31-O31-C3
6	B	205	3PE	C1-O11-P-O12
6	B	210	3PE	C1-O11-P-O12
6	D	205	3PE	C1-O11-P-O12
6	G	205	3PE	C1-O11-P-O12
7	A	409	LEA	C2-C3-C4-C5
7	C	409	LEA	C2-C3-C4-C5
7	F	409	LEA	C2-C3-C4-C5
7	I	409	LEA	C2-C3-C4-C5
6	A	407	3PE	O11-C1-C2-O21
6	C	407	3PE	O11-C1-C2-O21
6	F	407	3PE	O11-C1-C2-O21
6	I	407	3PE	O11-C1-C2-O21
4	A	405	SHV	O2-C1-C2-C3
4	C	405	SHV	O2-C1-C2-C3
4	F	405	SHV	O2-C1-C2-C3
4	I	405	SHV	O2-C1-C2-C3
6	A	407	3PE	C2-C1-O11-P
6	C	407	3PE	C2-C1-O11-P
6	F	407	3PE	C2-C1-O11-P
6	I	407	3PE	C2-C1-O11-P

Continued on next page...

For Manuscript Review

Continued from previous page...

Mol	Chain	Res	Type	Atoms
7	B	201	LEA	O1-C2-C3-C4
7	D	201	LEA	O1-C2-C3-C4
7	G	201	LEA	O1-C2-C3-C4
7	J	202	LEA	O1-C2-C3-C4
7	B	201	LEA	O2-C2-C3-C4
7	B	204	LEA	O1-C2-C3-C4
7	D	201	LEA	O2-C2-C3-C4
7	D	204	LEA	O1-C2-C3-C4
7	G	201	LEA	O2-C2-C3-C4
7	G	204	LEA	O1-C2-C3-C4
7	J	202	LEA	O2-C2-C3-C4
7	J	205	LEA	O1-C2-C3-C4
4	B	203	SHV	C2-C3-C4-C5
4	D	203	SHV	C2-C3-C4-C5
4	G	203	SHV	C2-C3-C4-C5
4	J	204	SHV	C2-C3-C4-C5
4	B	206	SHV	O1-C1-C2-C3
4	D	206	SHV	O1-C1-C2-C3
4	G	206	SHV	O1-C1-C2-C3
4	J	206	SHV	O1-C1-C2-C3
7	B	207	LEA	O1-C2-C3-C4
7	D	207	LEA	O1-C2-C3-C4
7	G	207	LEA	O1-C2-C3-C4
7	J	207	LEA	O1-C2-C3-C4
4	A	404	SHV	O2-C1-C2-C3
4	B	206	SHV	O2-C1-C2-C3
4	C	404	SHV	O2-C1-C2-C3
4	D	206	SHV	O2-C1-C2-C3
4	F	404	SHV	O2-C1-C2-C3
4	G	206	SHV	O2-C1-C2-C3
4	I	404	SHV	O2-C1-C2-C3
4	J	206	SHV	O2-C1-C2-C3
7	B	204	LEA	O2-C2-C3-C4
7	D	204	LEA	O2-C2-C3-C4
7	G	204	LEA	O2-C2-C3-C4
7	J	205	LEA	O2-C2-C3-C4
4	A	405	SHV	O1-C1-C2-C3
4	C	405	SHV	O1-C1-C2-C3
4	F	405	SHV	O1-C1-C2-C3
4	I	405	SHV	O1-C1-C2-C3
7	B	207	LEA	O2-C2-C3-C4
7	D	207	LEA	O2-C2-C3-C4

Continued on next page...

For Manuscript Review

Continued from previous page...

Mol	Chain	Res	Type	Atoms
7	G	207	LEA	O2-C2-C3-C4
7	J	207	LEA	O2-C2-C3-C4
3	A	401	FAD	O4B-C4B-C5B-O5B
3	C	401	FAD	O4B-C4B-C5B-O5B
3	F	401	FAD	O4B-C4B-C5B-O5B
3	I	401	FAD	O4B-C4B-C5B-O5B
9	B	202	DKA	C3-C4-C5-C6
9	D	202	DKA	C3-C4-C5-C6
9	G	202	DKA	C3-C4-C5-C6
9	J	203	DKA	C3-C4-C5-C6
8	A	412	GOL	O1-C1-C2-O2
8	C	412	GOL	O1-C1-C2-O2
8	F	412	GOL	O1-C1-C2-O2
8	I	412	GOL	O1-C1-C2-O2
4	B	203	SHV	O2-C1-C2-C3
4	D	203	SHV	O2-C1-C2-C3
4	G	203	SHV	O2-C1-C2-C3
4	J	204	SHV	O2-C1-C2-C3
4	A	404	SHV	O1-C1-C2-C3
4	C	404	SHV	O1-C1-C2-C3
4	F	404	SHV	O1-C1-C2-C3
4	I	404	SHV	O1-C1-C2-C3
5	A	413	6NA	CA-CB-CG-CD
5	C	413	6NA	CA-CB-CG-CD
5	F	413	6NA	CA-CB-CG-CD
5	I	413	6NA	CA-CB-CG-CD
6	A	407	3PE	O11-C1-C2-C3
6	C	407	3PE	O11-C1-C2-C3
6	F	407	3PE	O11-C1-C2-C3
6	I	407	3PE	O11-C1-C2-C3
4	A	402	SHV	O2-C1-C2-C3
4	C	402	SHV	O2-C1-C2-C3
4	F	402	SHV	O2-C1-C2-C3
4	I	402	SHV	O2-C1-C2-C3
4	A	405	SHV	C2-C3-C4-C5
4	C	405	SHV	C2-C3-C4-C5
4	F	405	SHV	C2-C3-C4-C5
4	I	405	SHV	C2-C3-C4-C5
4	B	203	SHV	O1-C1-C2-C3
4	D	203	SHV	O1-C1-C2-C3
4	G	203	SHV	O1-C1-C2-C3
4	J	204	SHV	O1-C1-C2-C3

Continued on next page...

For Manuscript Review

Continued from previous page...

Mol	Chain	Res	Type	Atoms
4	A	405	SHV	C4-C5-C6-C7
4	C	405	SHV	C4-C5-C6-C7
4	F	405	SHV	C4-C5-C6-C7
4	I	405	SHV	C4-C5-C6-C7
6	A	407	3PE	C33-C34-C35-C36
6	C	407	3PE	C33-C34-C35-C36
6	F	407	3PE	C33-C34-C35-C36
6	I	407	3PE	C33-C34-C35-C36
4	A	402	SHV	O1-C1-C2-C3
4	C	402	SHV	O1-C1-C2-C3
4	F	402	SHV	O1-C1-C2-C3
4	I	402	SHV	O1-C1-C2-C3
5	A	403	6NA	CA-CB-CG-CD
5	C	403	6NA	CA-CB-CG-CD
5	F	403	6NA	CA-CB-CG-CD
5	I	403	6NA	CA-CB-CG-CD
6	A	407	3PE	O21-C21-C22-C23
6	C	407	3PE	O21-C21-C22-C23
6	F	407	3PE	O21-C21-C22-C23
6	I	407	3PE	O21-C21-C22-C23
6	A	407	3PE	C21-C22-C23-C24
6	C	407	3PE	C21-C22-C23-C24
6	F	407	3PE	C21-C22-C23-C24
6	I	407	3PE	C21-C22-C23-C24
4	A	404	SHV	C2-C3-C4-C5
4	C	404	SHV	C2-C3-C4-C5
4	F	404	SHV	C2-C3-C4-C5
4	I	404	SHV	C2-C3-C4-C5
6	A	407	3PE	C32-C33-C34-C35
6	C	407	3PE	C32-C33-C34-C35
6	F	407	3PE	C32-C33-C34-C35
6	I	407	3PE	C32-C33-C34-C35
9	B	208	DKA	C5-C6-C7-C8
9	D	208	DKA	C5-C6-C7-C8
9	G	208	DKA	C5-C6-C7-C8
9	J	208	DKA	C5-C6-C7-C8
6	A	407	3PE	C26-C27-C28-C29
6	C	407	3PE	C26-C27-C28-C29
6	F	407	3PE	C26-C27-C28-C29
6	I	407	3PE	C26-C27-C28-C29

There are no ring outliers.

For Manuscript Review

No monomer is involved in short contacts.

The following is a two-dimensional graphical depiction of Mogul quality analysis of bond lengths, bond angles, torsion angles, and ring geometry for all instances of the Ligand of Interest. In addition, ligands with molecular weight > 250 and outliers as shown on the validation Tables will also be included. For torsion angles, if less then 5% of the Mogul distribution of torsion angles is within 10 degrees of the torsion angle in question, then that torsion angle is considered an outlier. Any bond that is central to one or more torsion angles identified as an outlier by Mogul will be highlighted in the graph. For rings, the root-mean-square deviation (RMSD) between the ring in question and similar rings identified by Mogul is calculated over all ring torsion angles. If the average RMSD is greater than 60 degrees and the minimal RMSD between the ring in question and any Mogul-identified rings is also greater than 60 degrees, then that ring is considered an outlier. The outliers are highlighted in purple. The color gray indicates Mogul did not find sufficient equivalents in the CSD to analyse the geometry.

Ligand 3PE F 407

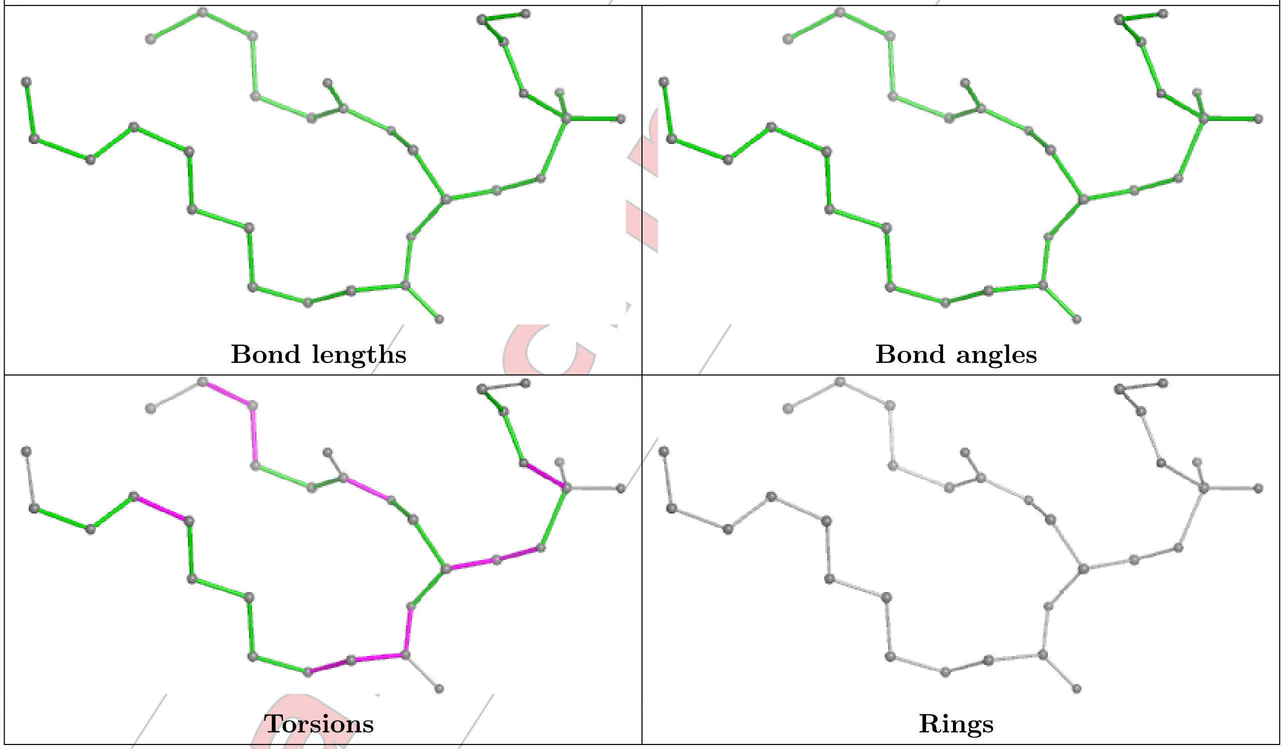

For Manuscript Review

Ligand 3PE I 407

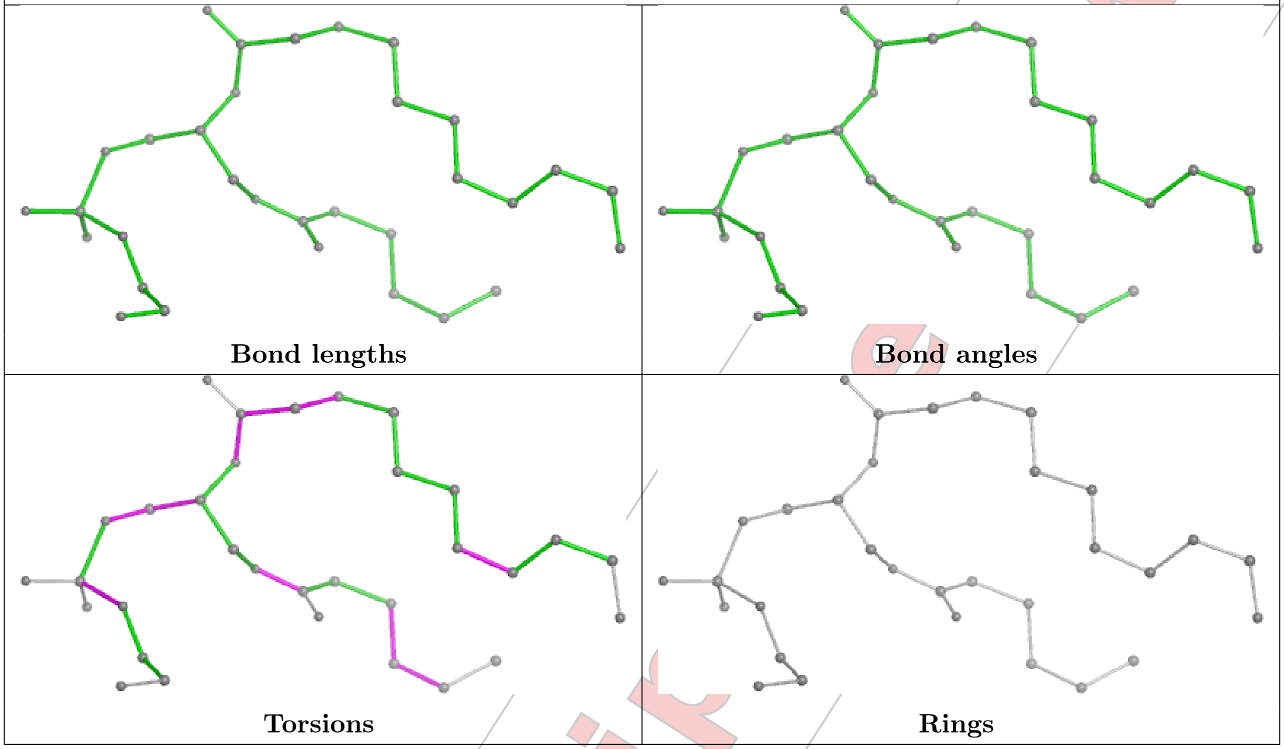

Bond lengths	Bond angles
Torsions	Rings

For Manuscript Review

Ligand 3PE G 205

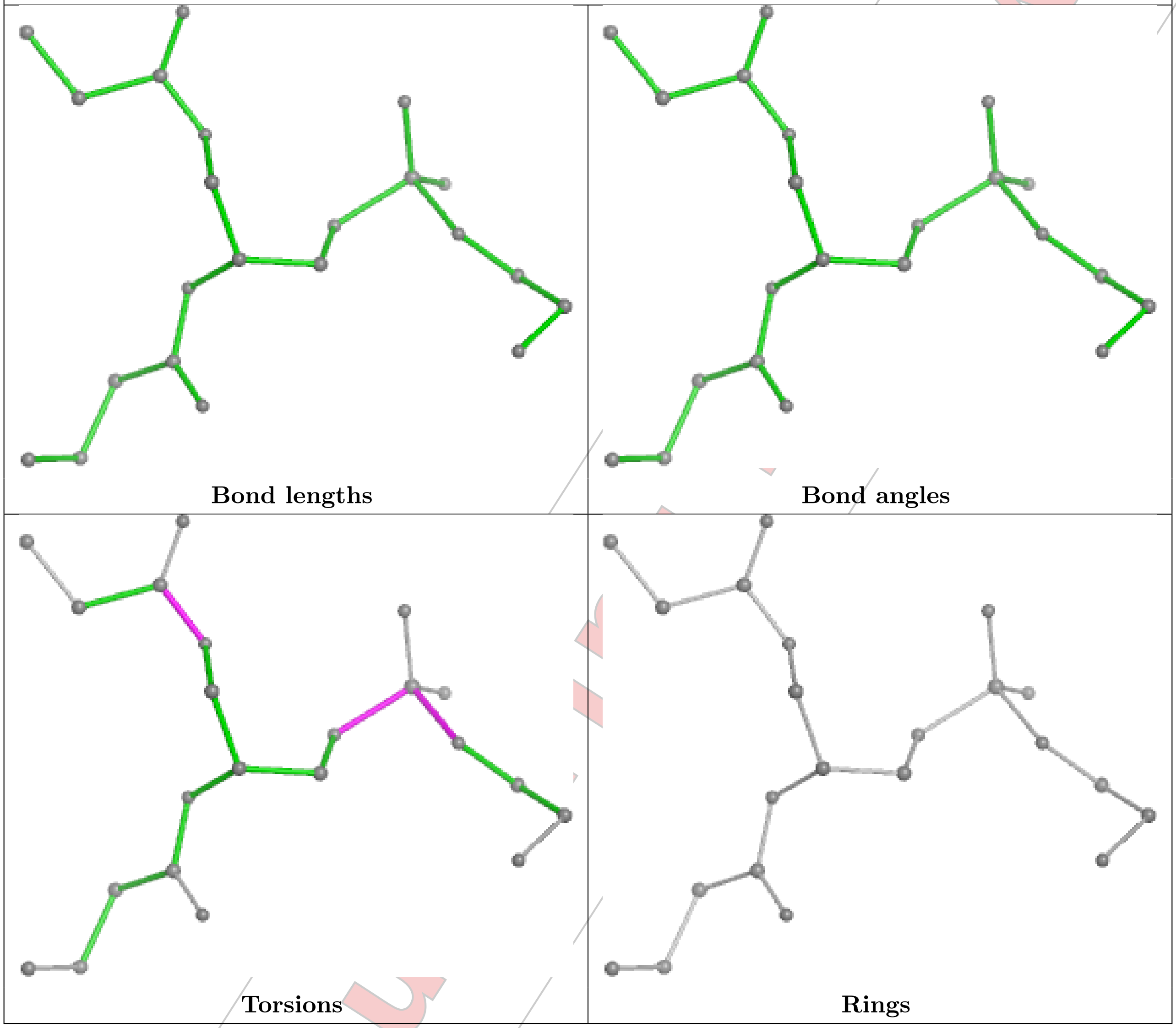

For Manuscript Review

Ligand 3PE A 407

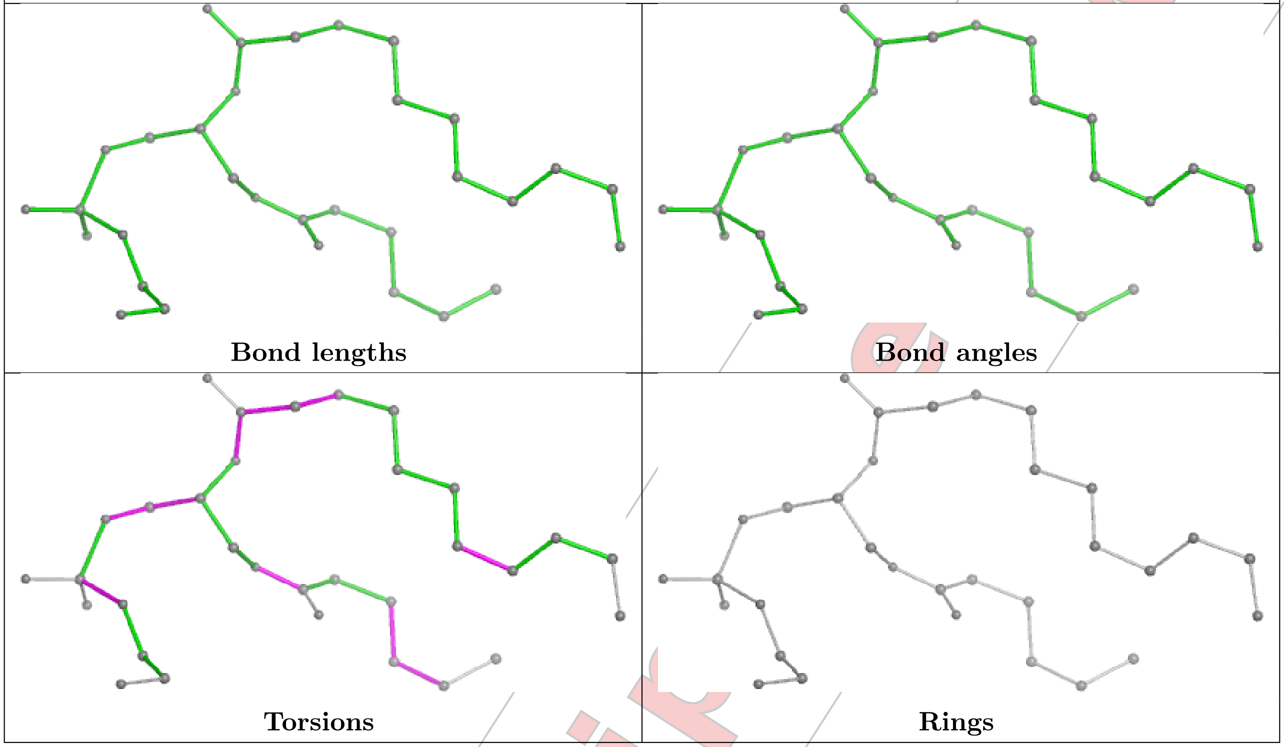

For Manuscript Review

Ligand 3PE D 205

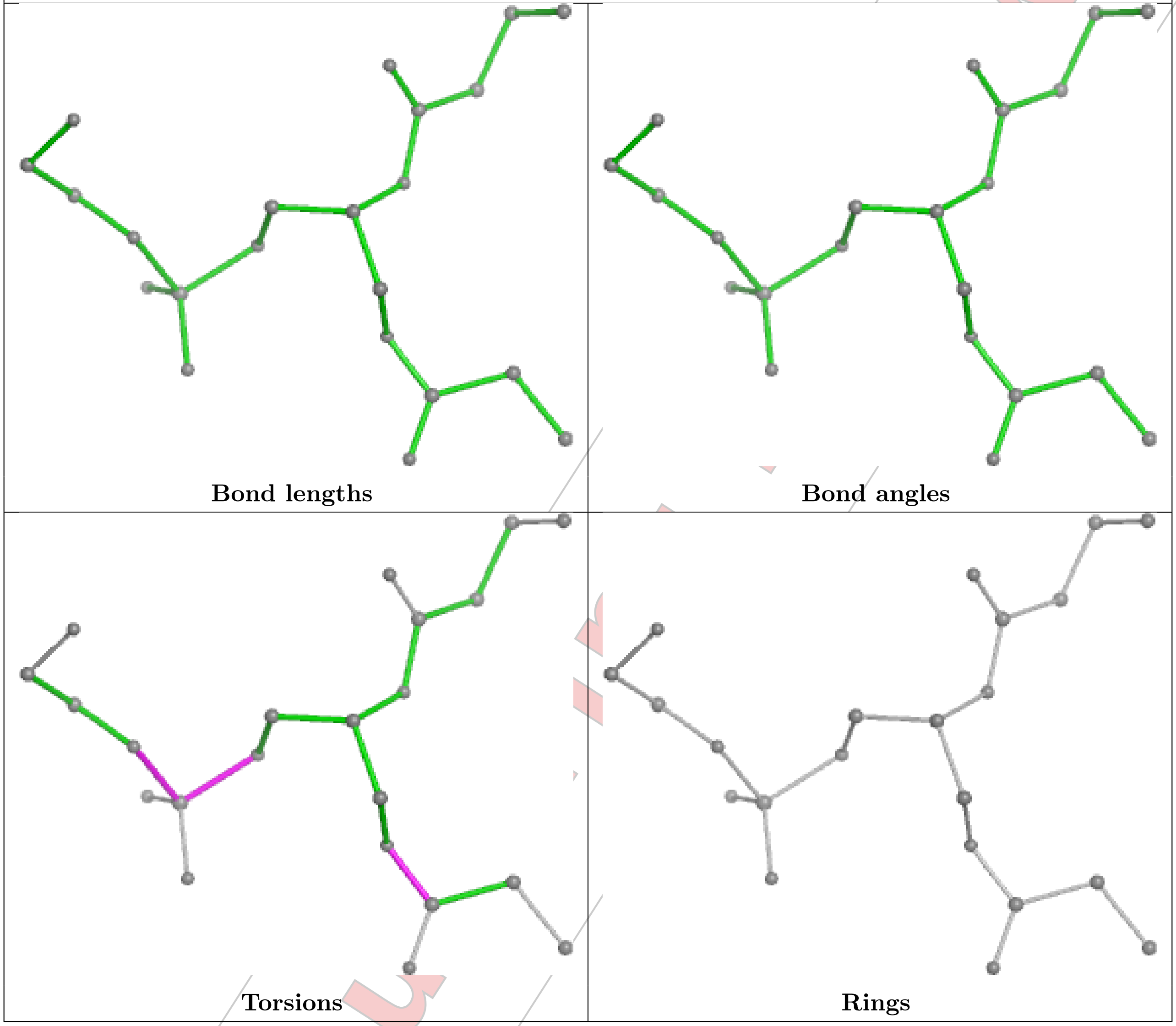

Ligand FAD I 401

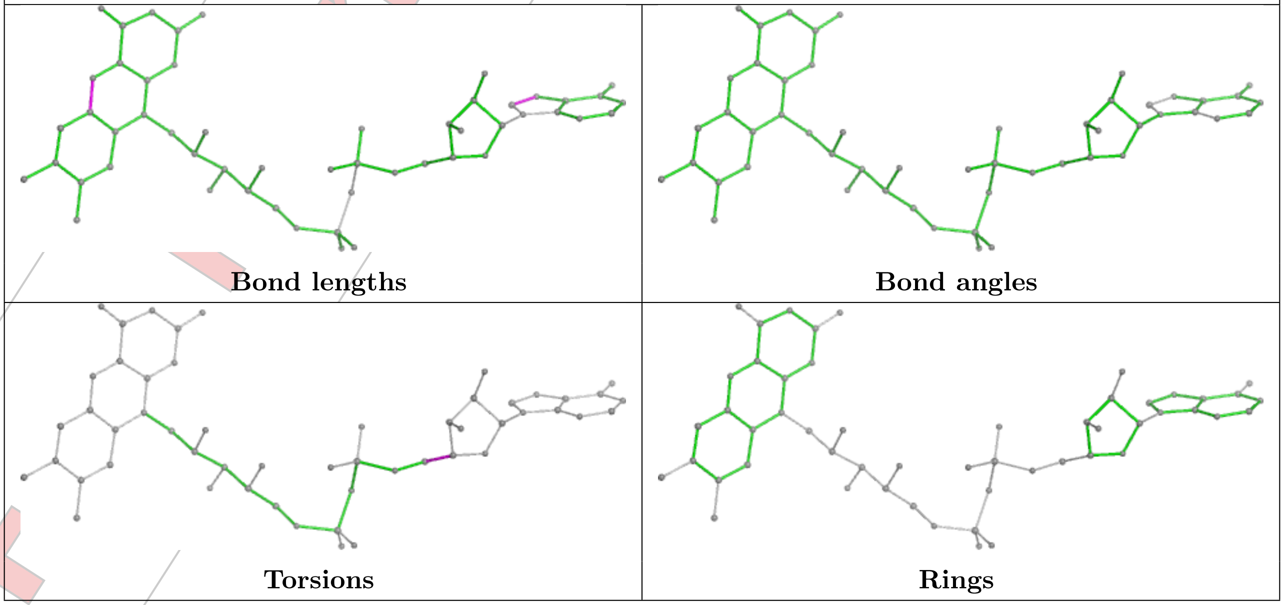

Ligand 3PE B 210

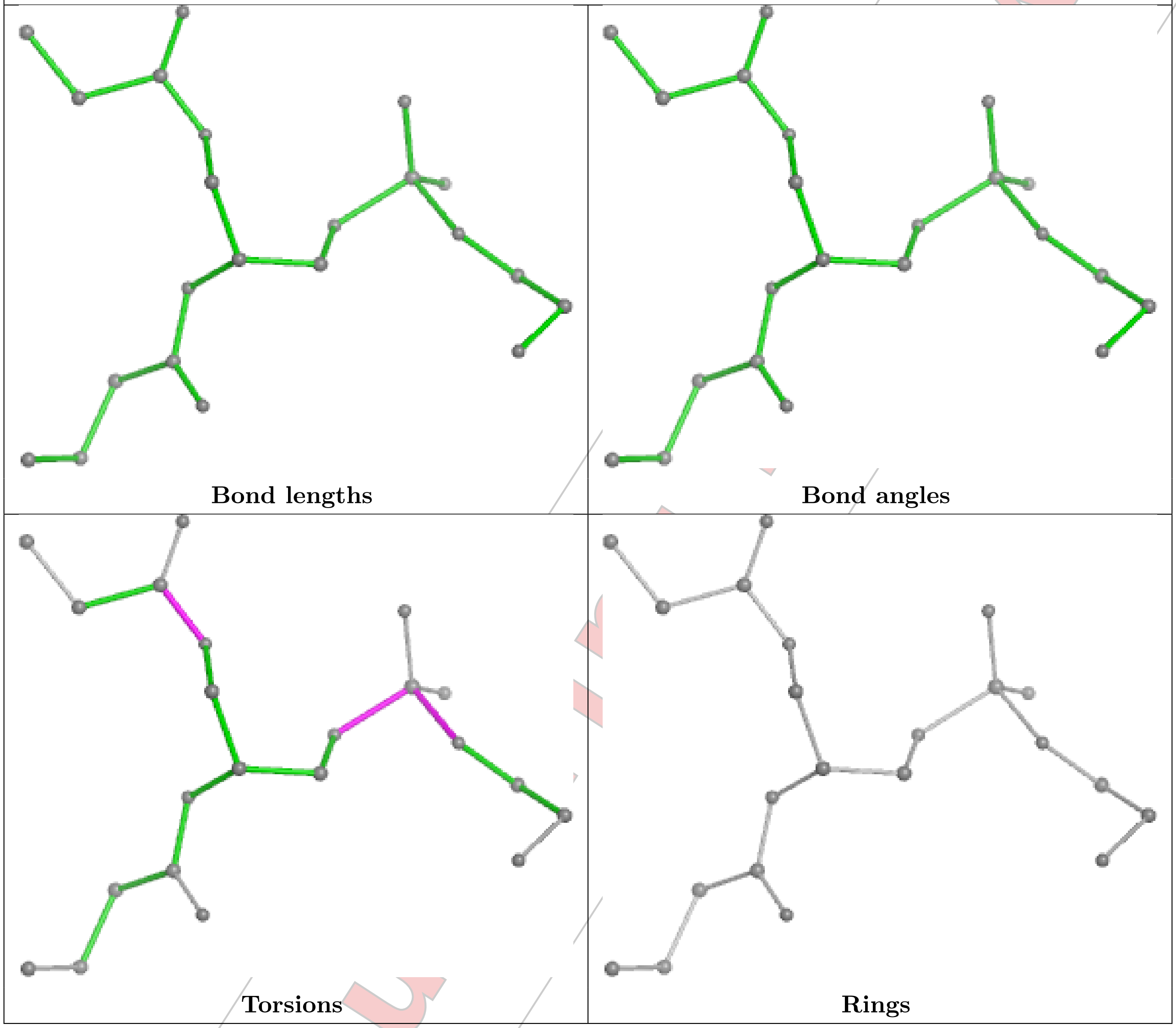

Ligand FAD A 401

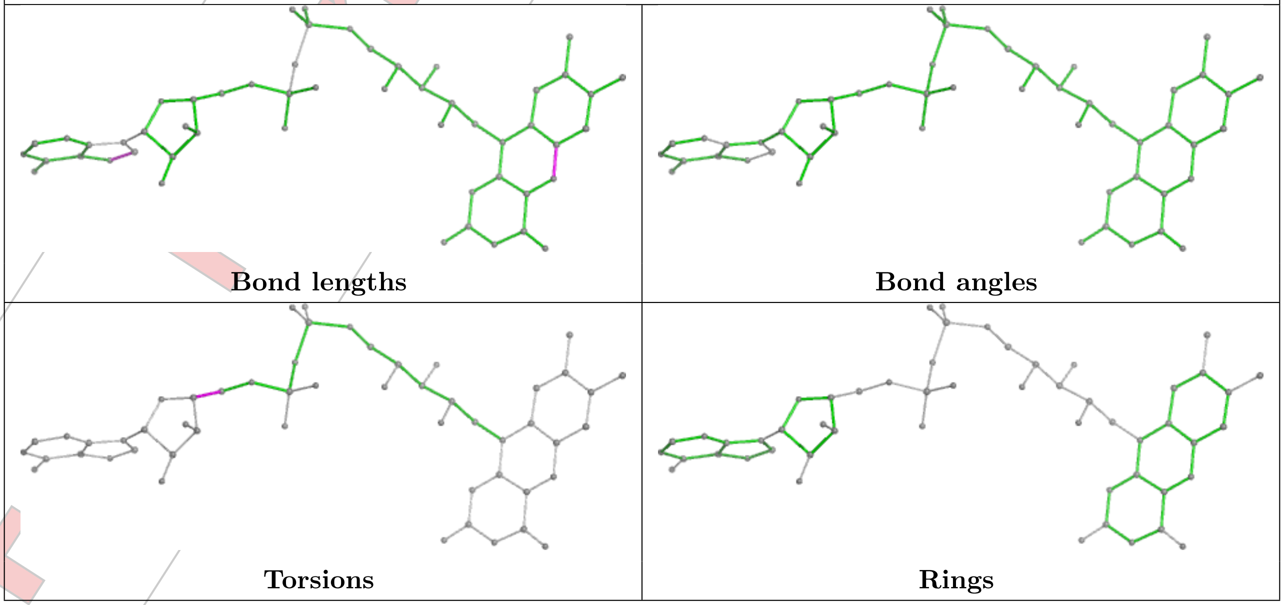

Ligand FAD C 401

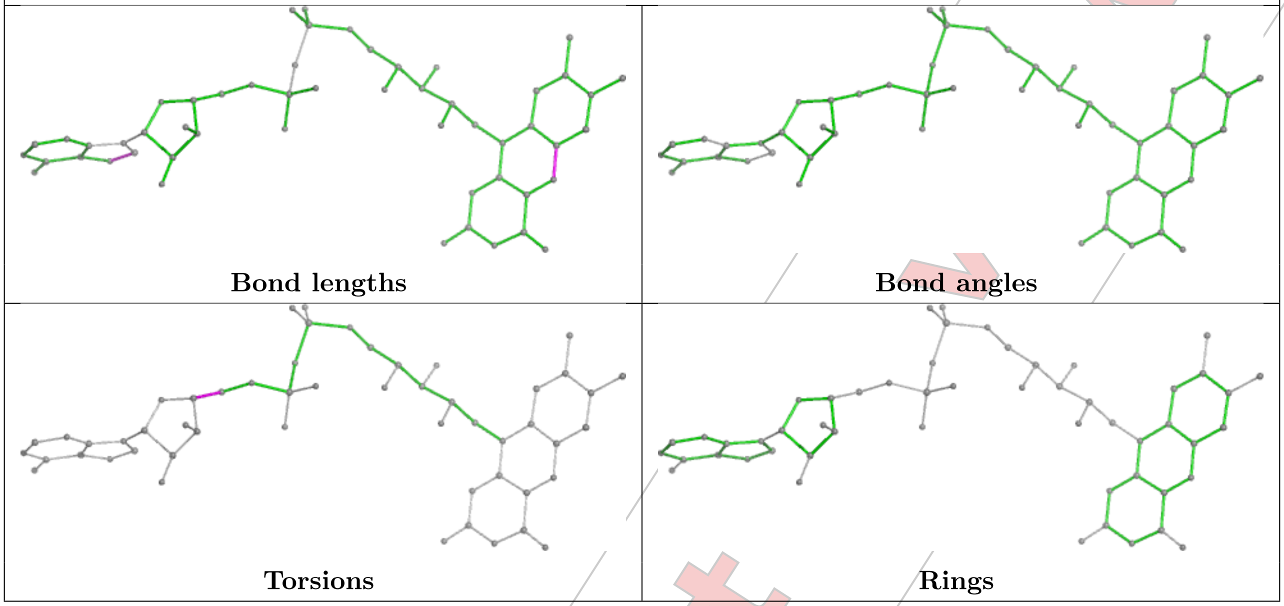

Ligand 3PE C 407

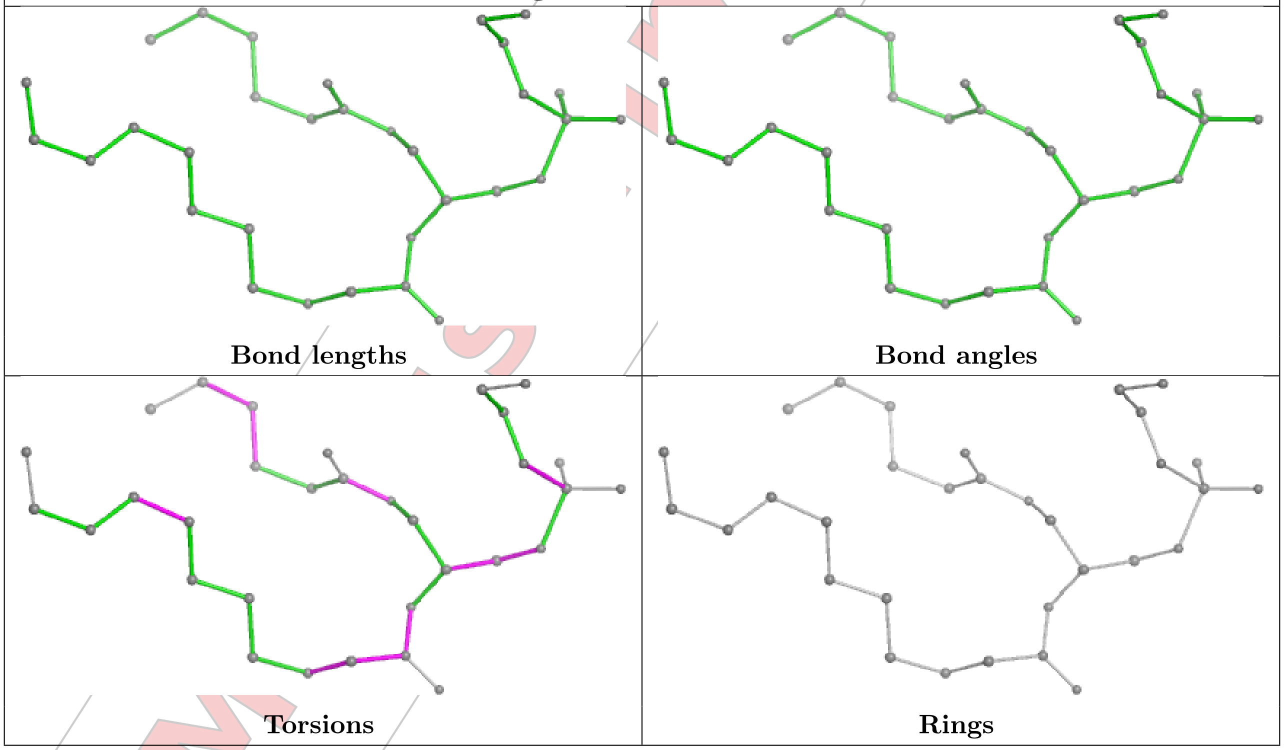

For Manuscript Review

Ligand FAD F 401

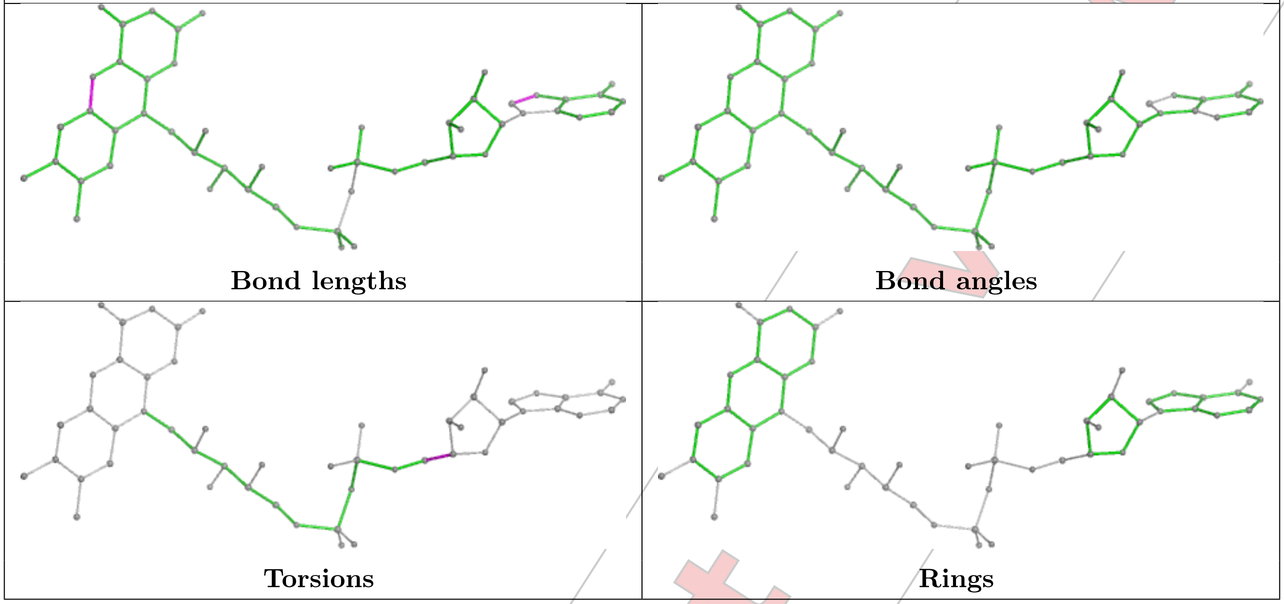

Ligand 3PE B 205

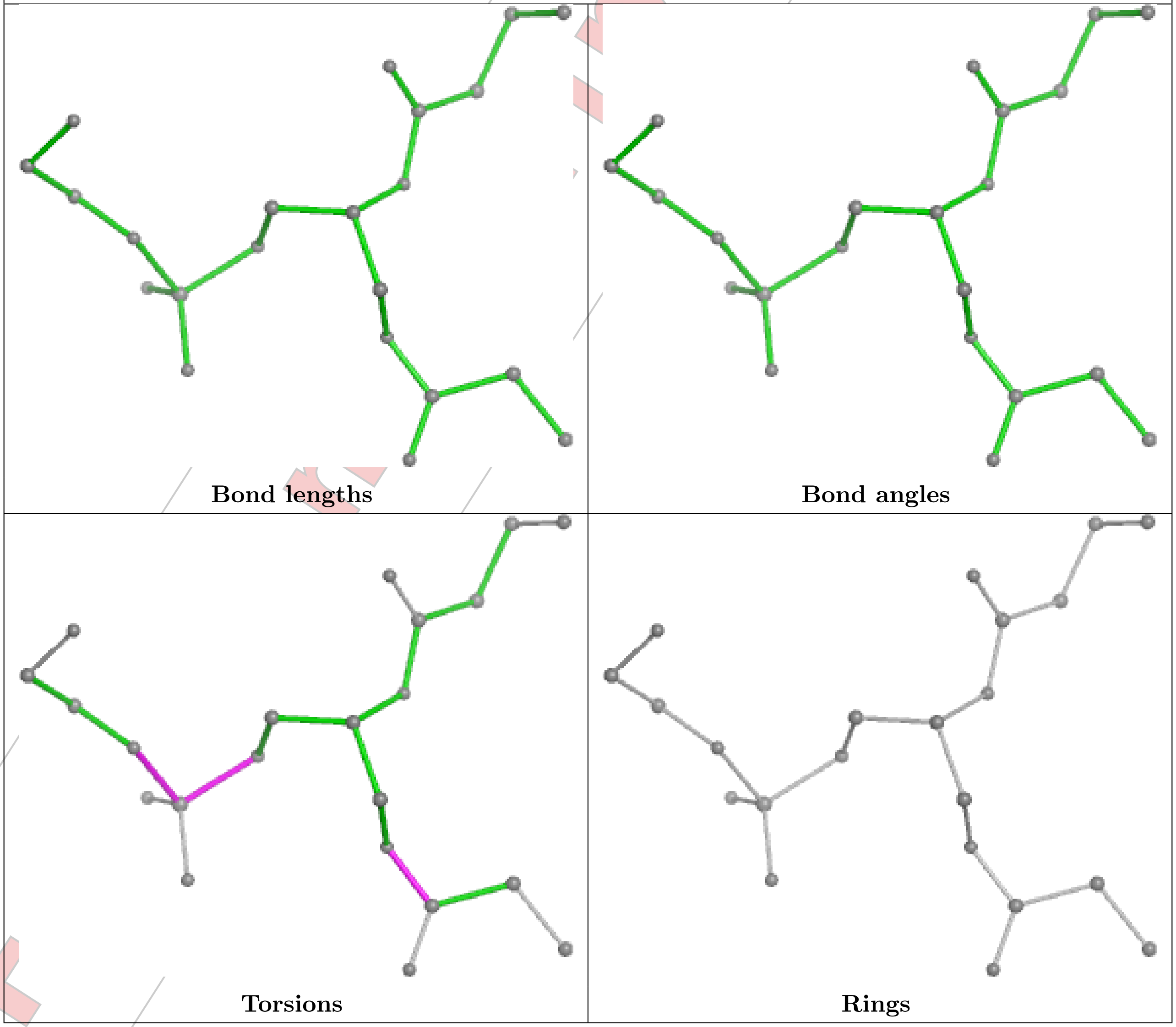

4.7 Other polymers (i)

There are no such residues in this entry.

4.8 Polymer linkage issues (i)

There are no chain breaks in this entry.

For Manuscript Review

5 Map visualisation (i)

This section contains visualisations of the EMDB entry EMD-55048. These allow visual inspection of the internal detail of the map and identification of artifacts.

Images derived from a raw map, generated by summing the deposited half-maps, are presented below the corresponding image components of the primary map to allow further visual inspection and comparison with those of the primary map.

5.1 Orthogonal projections (i)

5.1.1 Primary map

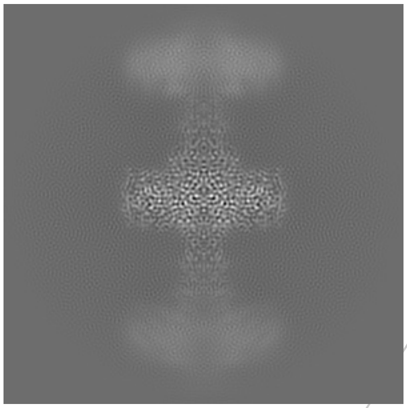
X

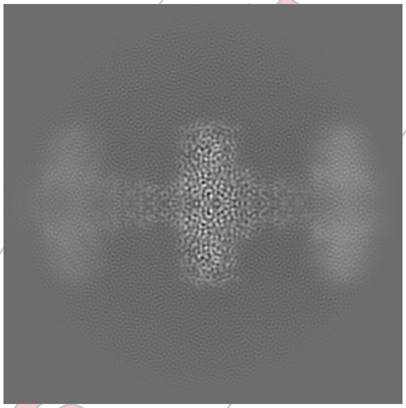
Y

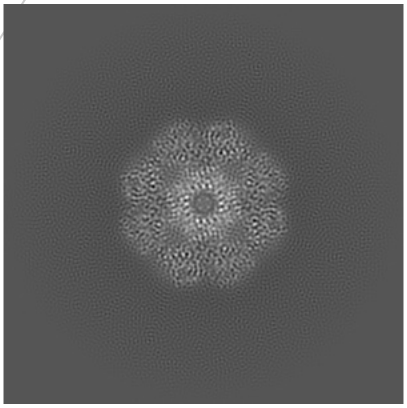
Z

5.1.2 Raw map

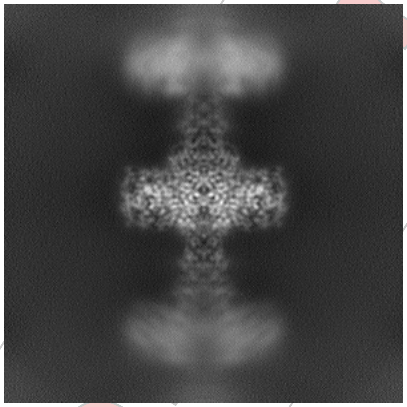
X

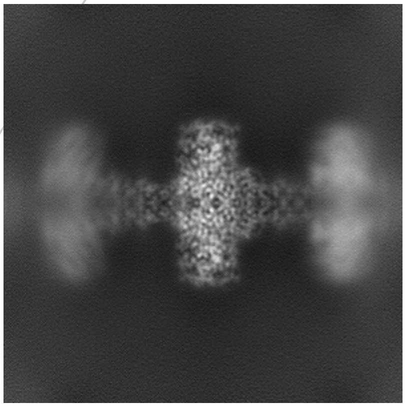
Y

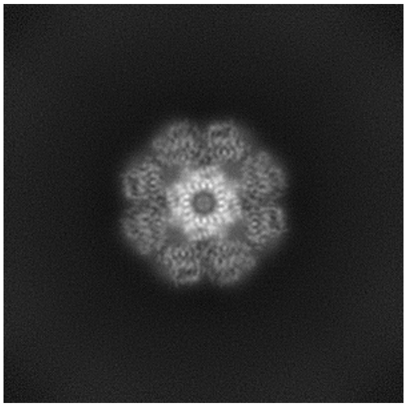
Z

The images above show the map projected in three orthogonal directions.

For Manuscript Review

5.2 Central slices (i)

5.2.1 Primary map

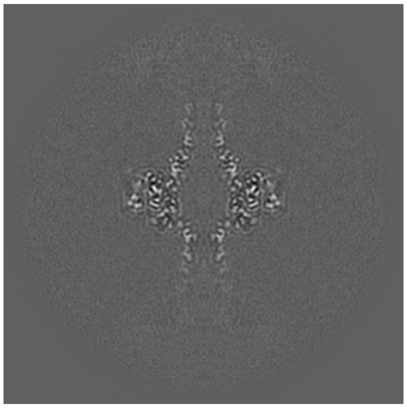

X Index: 250

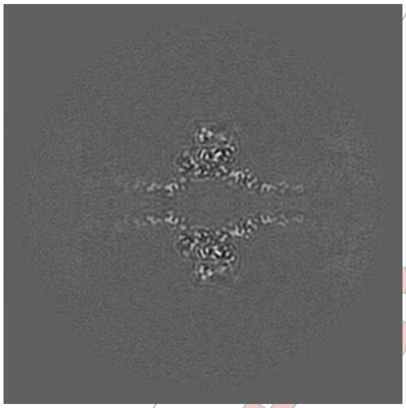

Y Index: 250

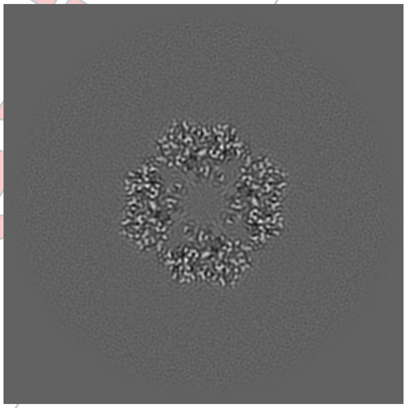

Z Index: 250

5.2.2 Raw map

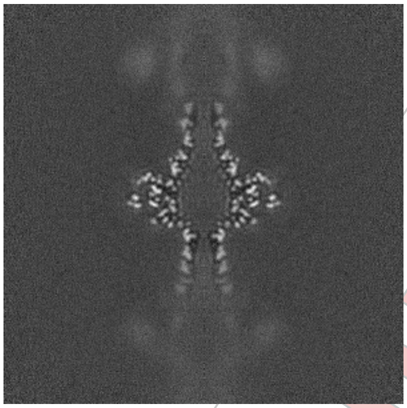

X Index: 250

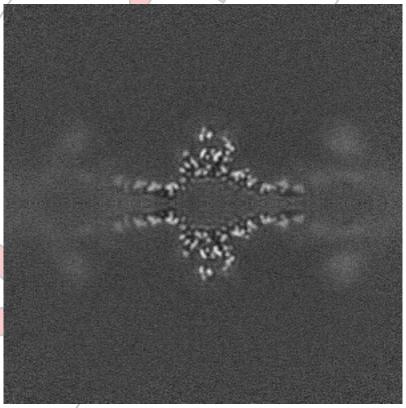

Y Index: 250

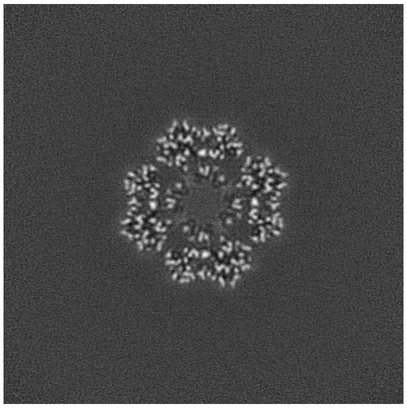

Z Index: 250

The images above show central slices of the map in three orthogonal directions.

For Manuscript Review

5.3 Largest variance slices (i)

5.3.1 Primary map

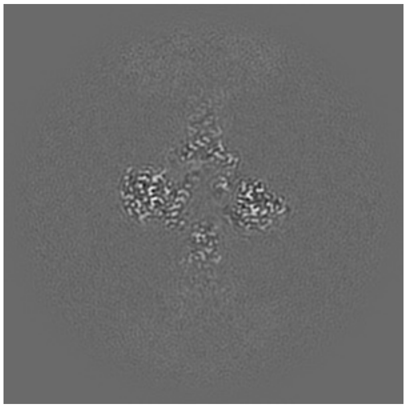
X Index: 232

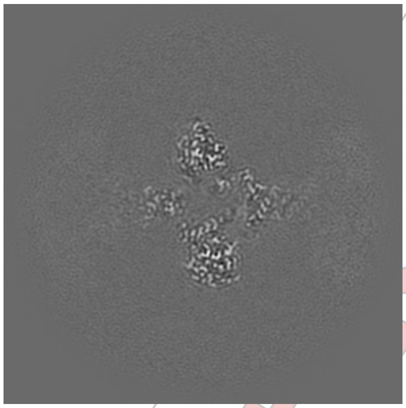
Y Index: 268

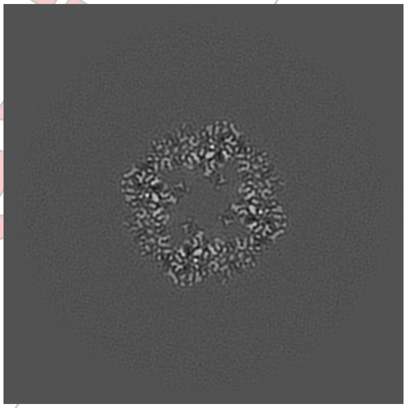
Z Index: 260

5.3.2 Raw map

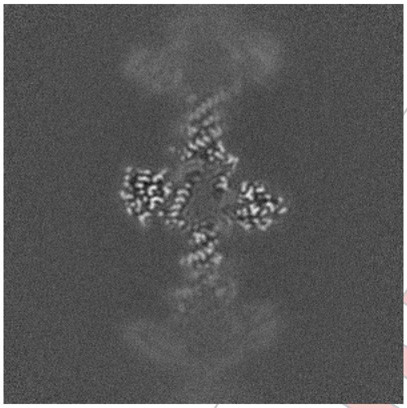
X Index: 232

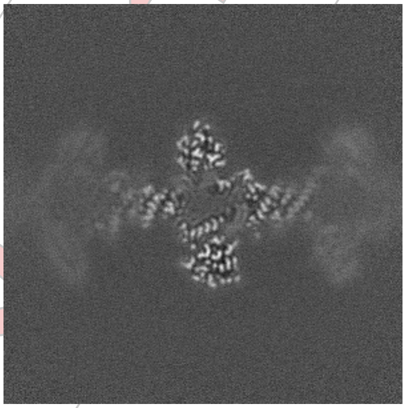
Y Index: 268

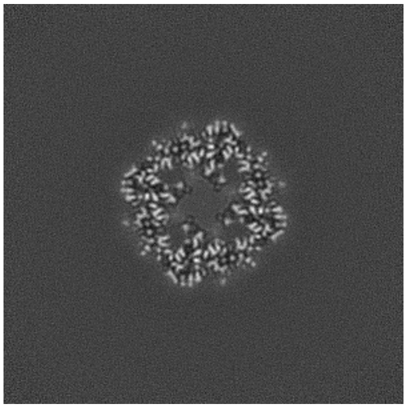
Z Index: 260

The images above show the largest variance slices of the map in three orthogonal directions.

For Manuscript Review

5.4 Orthogonal standard-deviation projections (False-color) ⓘ

5.4.1 Primary map

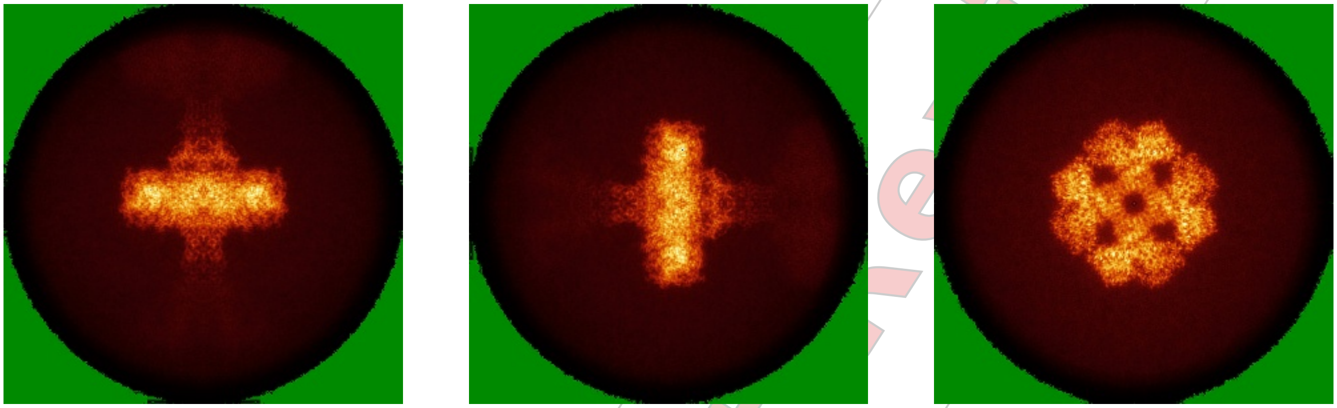

X Y Z

5.4.2 Raw map

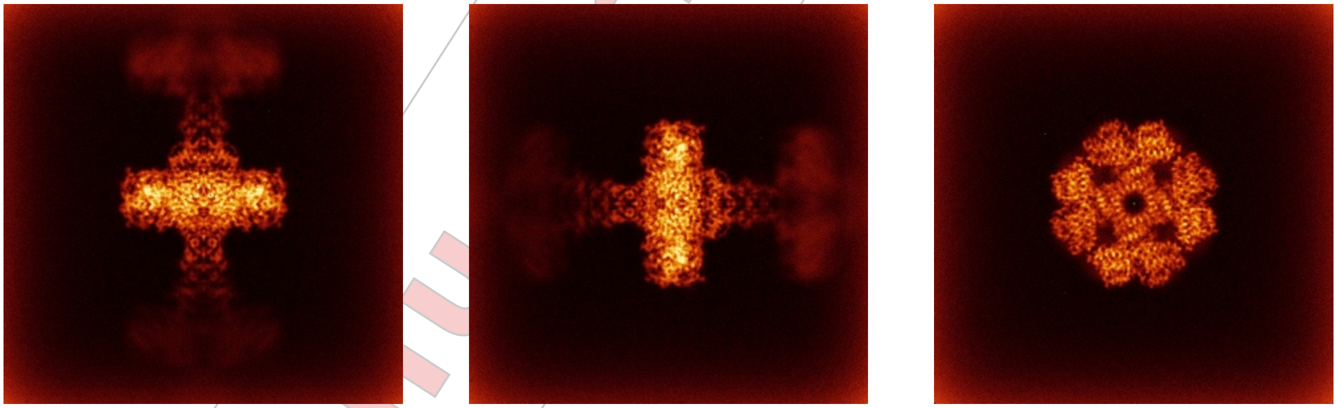

X Y Z

The images above show the map standard deviation projections with false color in three orthogonal directions. Minimum values are shown in green, max in blue, and dark to light orange shades represent small to large values respectively.

For Manuscript Review

5.5 Orthogonal surface views (i)

5.5.1 Primary map

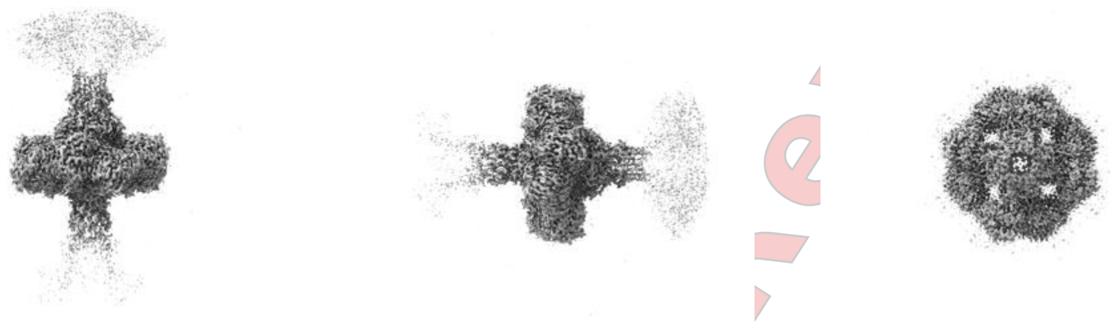

X Y Z

The images above show the 3D surface view of the map at the recommended contour level 0.068. These images, in conjunction with the slice images, may facilitate assessment of whether an appropriate contour level has been provided.

5.5.2 Raw map

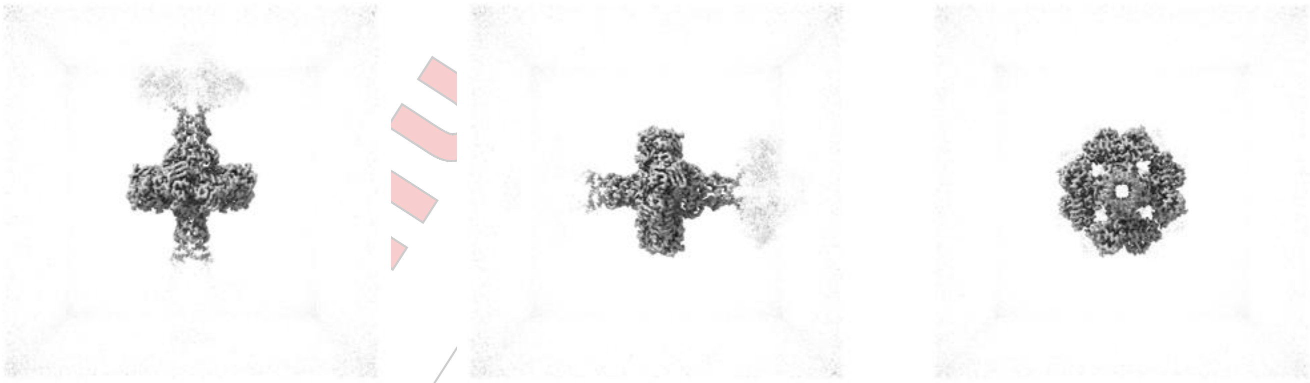

X Y Z

These images show the 3D surface of the raw map. The raw map's contour level was selected so that its surface encloses the same volume as the primary map does at its recommended contour level.

5.6 Mask visualisation (i)

This section was not generated. No masks/segmentation were deposited.

For Manuscript Review

6 Map analysis (i)

This section contains the results of statistical analysis of the map.

6.1 Map-value distribution (i)

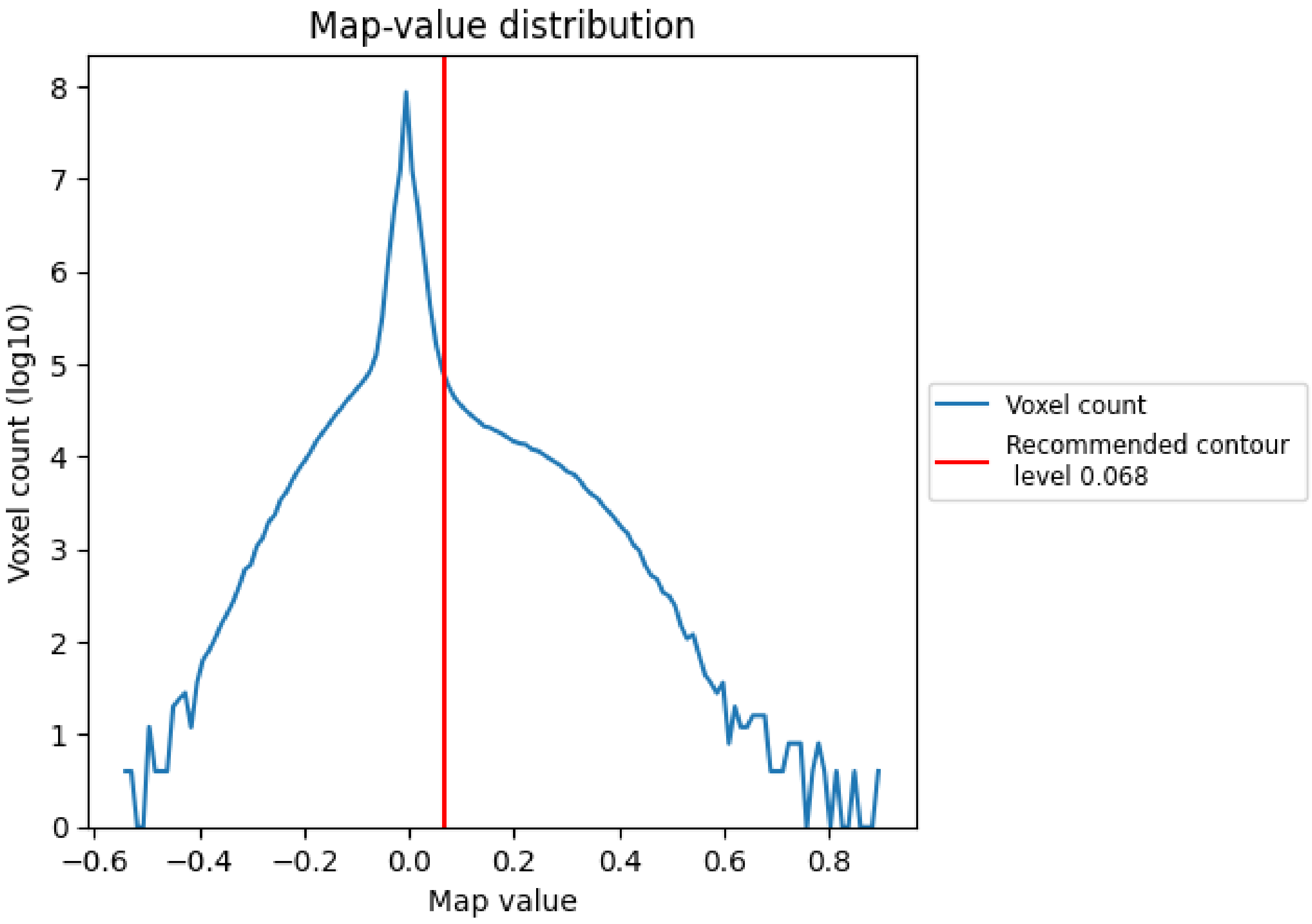

The map-value distribution is plotted in 128 intervals along the x-axis. The y-axis is logarithmic. A spike in this graph at zero usually indicates that the volume has been masked.

For Manuscript Review

6.2 Volume estimate (i)

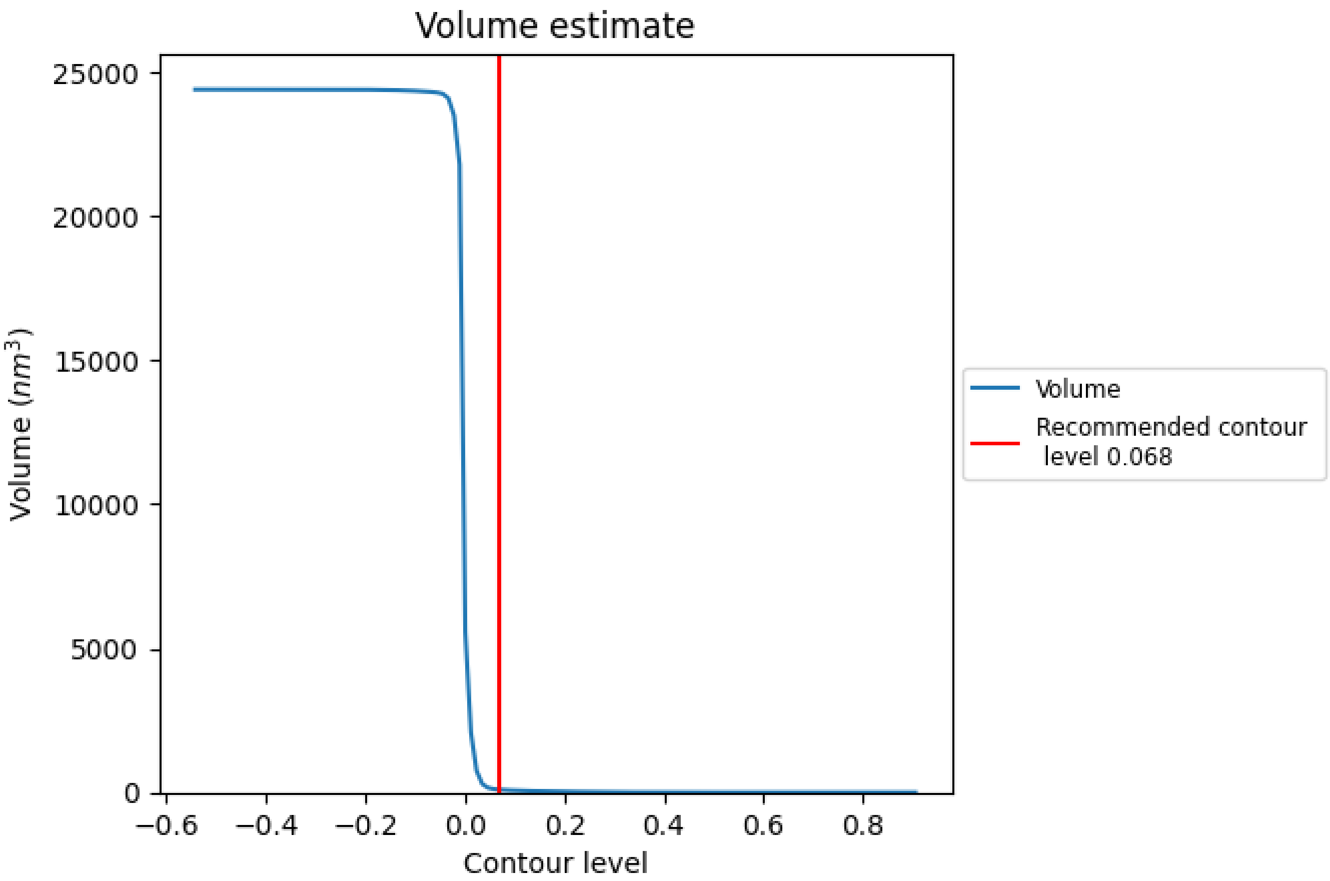

The volume at the recommended contour level is 100 nm^3; this corresponds to an approximate mass of 90 kDa.

The volume estimate graph shows how the enclosed volume varies with the contour level. The recommended contour level is shown as a vertical line and the intersection between the line and the curve gives the volume of the enclosed surface at the given level.

For Manus

6.3 Rotationally averaged power spectrum (i)

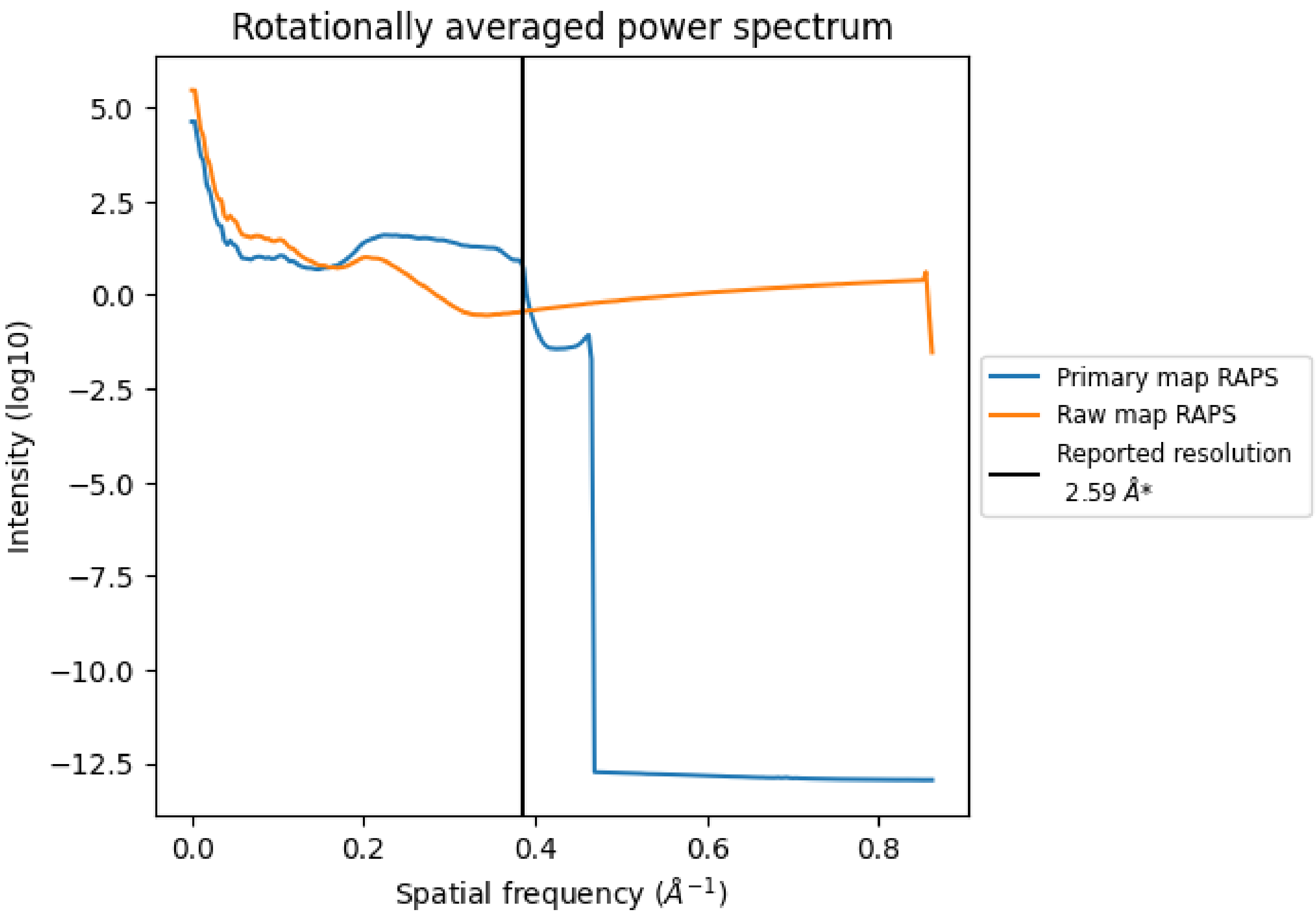

*Reported resolution corresponds to spatial frequency of 0.386 Å^{-1}

For Manuscript Review

7 Fourier-Shell correlation (i)

Fourier-Shell Correlation (FSC) is the most commonly used method to estimate the resolution of single-particle and subtomogram-averaged maps. The shape of the curve depends on the imposed symmetry, mask and whether or not the two 3D reconstructions used were processed from a common reference. The reported resolution is shown as a black line. A curve is displayed for the half-bit criterion in addition to lines showing the 0.143 gold standard cut-off and 0.5 cut-off.

7.1 FSC (i)

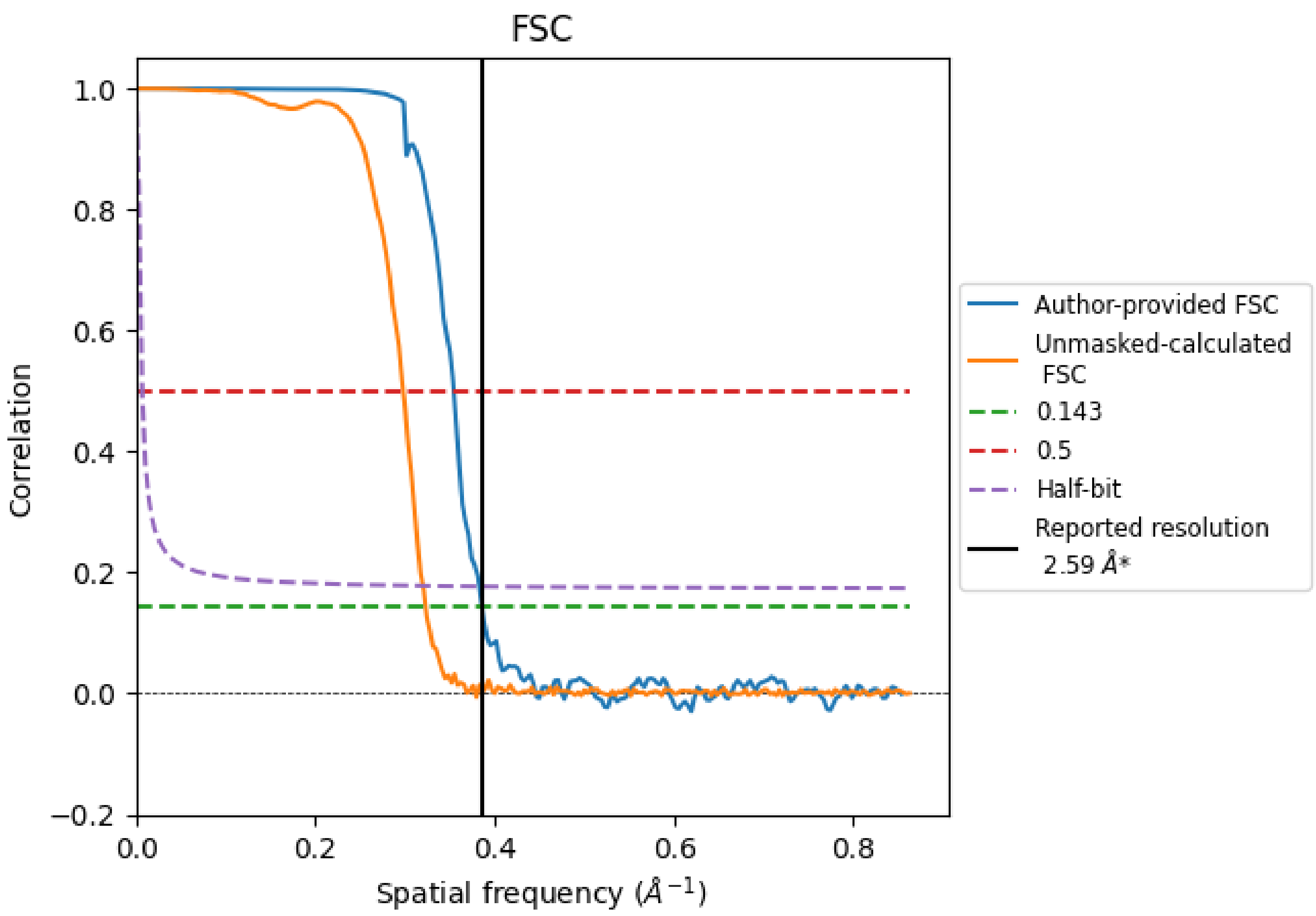

*Reported resolution corresponds to spatial frequency of 0.386 Å^{-1}

For Manuscript Review

7.2 Resolution estimates (i)

Resolution estimate (Å)	Estimation criterion (FSC cut-off)		
	0.143	0.5	Half-bit
Reported by author	2.59	-	-
Author-provided FSC curve	2.59	2.82	2.61
Unmasked-calculated*	3.10	3.36	3.13

*Resolution estimate based on FSC curve calculated by comparison of deposited half-maps. The value from deposited half-maps intersecting FSC 0.143 CUT-OFF 3.10 differs from the reported value 2.59 by more than 10 %

For Manuscript Review

8 Map-model fit (i)

This section contains information regarding the fit between EMDB map EMD-55048 and PDB model 9SNK. Per-residue inclusion information can be found in section ?? on page ??.

8.1 Map-model overlay (i)

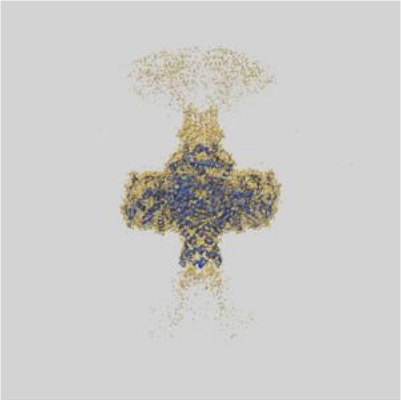

X

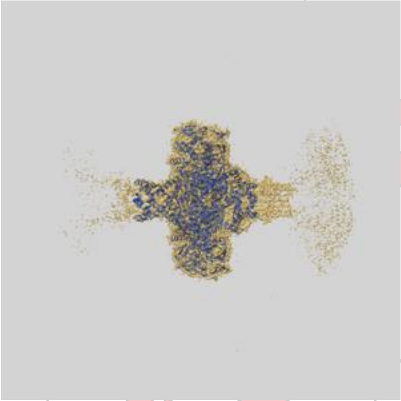

Y

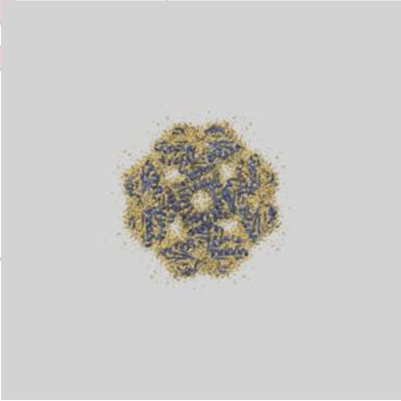

Z

The images above show the 3D surface view of the map at the recommended contour level 0.068 at 50% transparency in yellow overlaid with a ribbon representation of the model coloured in blue. These images allow for the visual assessment of the quality of fit between the atomic model and the map.

For Manuscript Review

8.2 Q-score mapped to coordinate model (i)

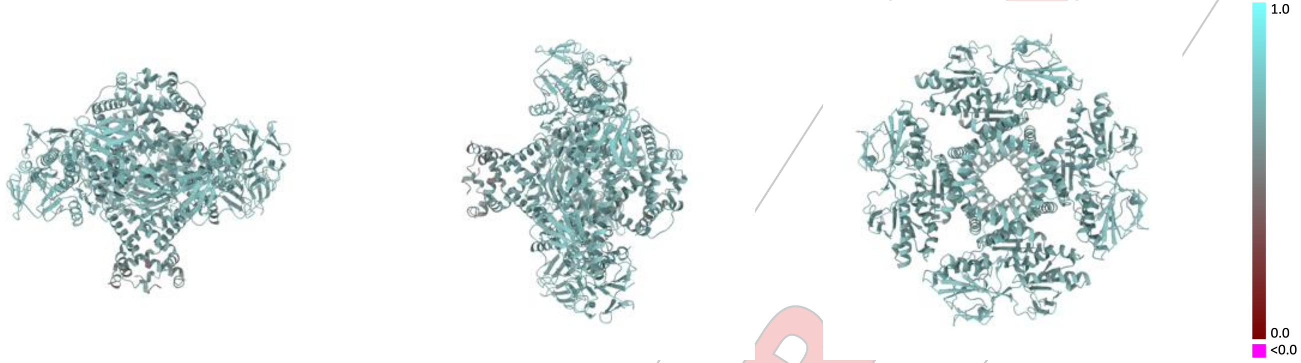

X Y Z

The images above show the model with each residue coloured according its Q-score. This shows their resolvability in the map with higher Q-score values reflecting better resolvability. Please note: Q-score is calculating the resolvability of atoms, and thus high values are only expected at resolutions at which atoms can be resolved. Low Q-score values may therefore be expected for many entries.

8.3 Atom inclusion mapped to coordinate model (i)

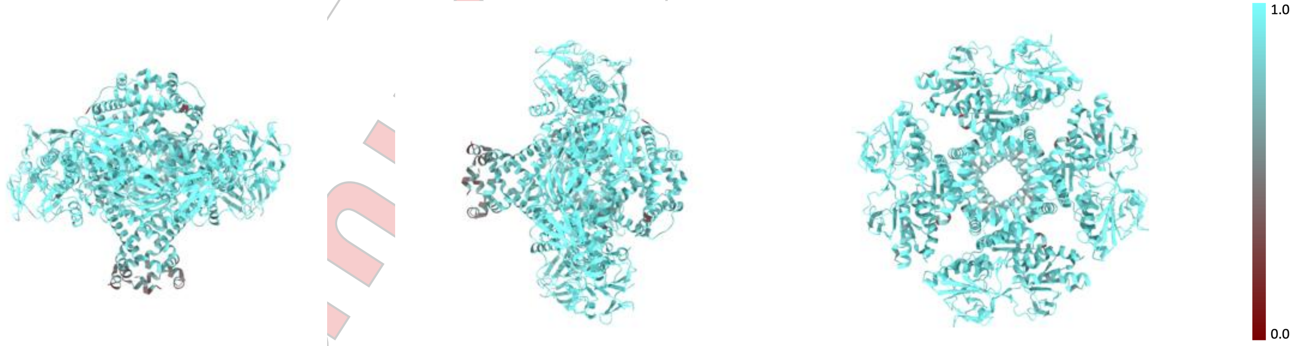

X Y Z

The images above show the model with each residue coloured according to its atom inclusion. This shows to what extent they are inside the map at the recommended contour level (0.068).

For Manuscript Review

8.4 Atom inclusion ⓘ

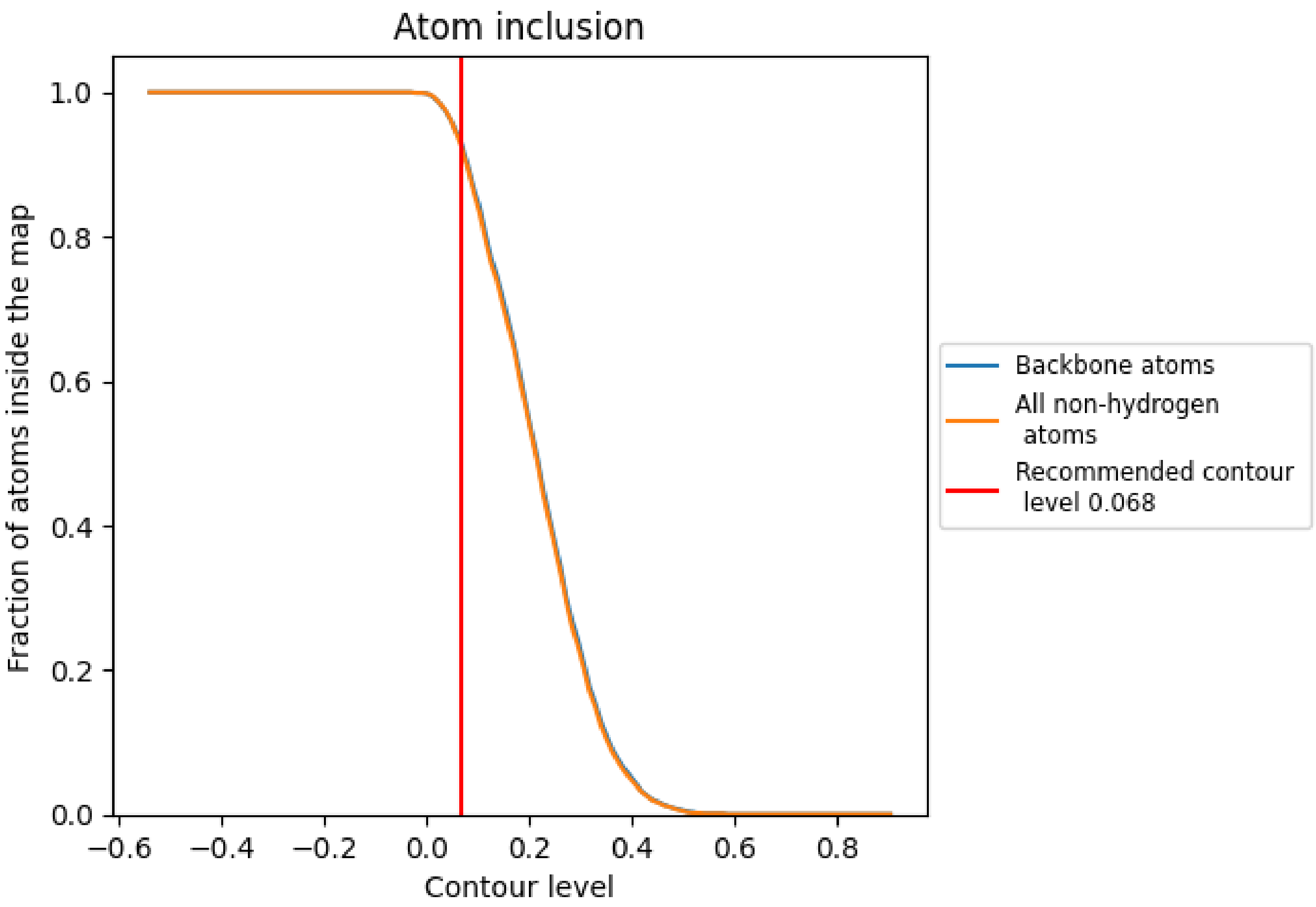

At the recommended contour level, 93% of all backbone atoms, 92% of all non-hydrogen atoms, are inside the map.

For Manuscript Review

8.5 Map-model fit summary (i)

The table lists the average atom inclusion at the recommended contour level (0.068) and Q-score for the entire model and for each chain.

Chain	Atom inclusion	Q-score
All	0.9240	0.6550
A	0.9510	0.6640
B	0.8360	0.6230
C	0.9510	0.6640
D	0.8430	0.6240
F	0.9510	0.6650
G	0.8430	0.6250
I	0.9510	0.6650
J	0.8500	0.6260

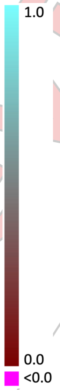

For Manuscript Review
